# Role of chromosome ends in meiotic stability, recombination and wheat evolution in the context of breeding

**DOI:** 10.1186/s12870-025-08020-5

**Published:** 2025-12-29

**Authors:** A. Gálvez-Galván, M. Aguilar, P. Prieto

**Affiliations:** 1 Plant Breeding Department, Institute for Sustainable Agriculture, Agencia Estatal Consejo Superior de Investigaciones Científicas (CSIC), Avda. Menéndez Pidal s/n, Campus Alameda del Obispo s/n, Apartado, Córdoba, 4084, 14004 Spain; 2 Programa de Doctorado Biociencias y Ciencias Agroalimentarias, Universidad de Córdoba, Córdoba, Spain; 3 Área de Fisiología Vegetal, Universidad de Córdoba, Campus Rabanales, edif. C4, 3ª planta, Córdoba, Spain

**Keywords:** Telomeres, Subtelomeres, Chromosome pairing, Homologous pairing, Chromosome dynamics, G4, Triticum aestivum, Bread wheat, Triticum turgidum, Durum wheat, Triticum dicoccoides, Aegilops tauschii, Wheat evolution and domestication, Meiosis, Genome evolution, Genome organisation, DNA-binding protein, Chromatin structure, Chromosome ends

## Abstract

Wheat is one of the most important crops worldwide, and understanding its genome organisation is crucial for geneticists and breeders. In this study, we examined the dynamic roles of telomeric and subtelomeric regions in wheat, focusing on their influence on homologous chromosome pairing during meiosis, the process that produces gametes. We analysed various *Triticum* species and modern cultivars, uncovering a complex “barcode” at chromosome ends that rules homologous recognition. Phylogenetic analysis of the *ZIP4-5B* gene highlighted the evolutionary relationships among wheat species, emphasising the contribution of wild relatives to genetic diversity, especially in terminal chromosomal regions. Our findings suggest that telomeric regions, although traditionally seen as conserved, display significant variability and structural complexity influenced by genetic background and chromosomal context. We observed a strong link between telomere position and variant accumulation, with subtelomeric regions acting as hot spots for instability and chromatin remodelling. G-quadruplex (G4) structures were found to be distributed unevenly, with their density affected by telomere distance and genomic context. Subtelomeric regions were identified as key sites for genetic improvement, harbouring rapidly evolving sequences and transposable elements that may impact meiotic pairing accuracy. Our results indicate that telomeres and subtelomeres serve as dynamic genomic centres, encoding chromosomal identity and regulating homologous pairing through a balance of sequence diversity and structural motifs. This research enhances our understanding of wheat genome stability and provides insights for breeding strategies aimed at increasing genetic diversity.

**Supplementary Information:**

The online version contains supplementary material available at 10.1186/s12870-025-08020-5.

## Key Message

Telomeric and subtelomeric regions in wheat play a dynamic and critical role in meiotic chromosome pairing by acting as genomic “barcodes” that guide homologous recognition. These regions, once considered stable, show high variability and structural complexity influenced by genetic and evolutionary factors, especially through contributions from wild wheat relatives. The findings highlight subtelomeres as hotspots for genetic improvement and genome evolution, offering valuable insights for enhancing wheat breeding and genetic diversity.

**Supplementary Information:**

The online version contains supplementary material available at 10.1186/s12870-025-08020-5.

## Introduction

Wheat is a cornerstone staple crop for global civilisations, delivering critical nutrients including proteins, dietary fibre, B vitamins, minerals, and phytochemicals. Beyond human consumption, it serves as a vital component of animal feed. The predominant cultivated species are hexaploid bread wheat (*Triticum aestivum* L.), primarily utilized for flour production, and tetraploid durum wheat (*T. turgidum* subsp. durum (Desf.) Husn.), mainly employed in pasta manufacturing. As one of the earliest domesticated crops, wheat’s cultivation originated in the Middle East approximately 10,000 years ago, marking a key development in agricultural history.

A hybridisation event between a species related to *Triticum urartu* (2n = 2x = 14; genome AA) [1, 2] and a species similar to *Aegilops speltoides* (2n = 2x = 14; genome BB) [3] gave rise to the tetraploid wild emmer wheat, *Triticum turgidum* subsp. dicoccoides (Körn. ex Asch. & Graebn.) Thell. (2n = 4x = 28; genome AABB). Subsequent domestication events led to the development of domesticated emmer wheat, *T. turgidum* L. subsp. dicoccon (Schrank) Thell. (2n = 4x = 28; genome AABB) [3–5], and later to cultivated durum wheat, *T. turgidum* subsp. durum (2n = 4x = 28; genome AABB) [6]. Spontaneous hybridisation between cultivated durum wheat and the wild species *Aegilops tauschii* (2n = 2x = 14; genome DD) resulted in the formation of bread wheat, *Triticum aestivum* L. (2n = 6x = 42; genome AABBDD) [7–13].

Despite the critical importance of wheat, with global production approximating 800 million tonnes across roughly 220 million hectares (FAOSTAT, https://www.fao.org/faostat/es/#data/QCL/visualize; consulted 20/07/2025), its productivity faces several challenges from climate change. Rising temperatures, erratic precipitation, and increasingly frequent extreme weather events, such as droughts and heatwaves, adversely impact yield, grain quality, plant development, and disease prevalence. With the global population continuing to increase, there is an urgent demand for wheat cultivars tailored to specific agro-climatic conditions and capable of delivering enhanced yields. Breeding programmes must therefore prioritise supporting wheat’s resilience to climatic extremes to ensure food security and promote sustainable agriculture, while simultaneously improving both yield and quality. These efforts need significant investment and interdisciplinary collaboration across scientific fields. Advancements in plant breeding, complemented by the emergence of ‘omics technologies and computational tools, provide robust opportunities to advance wheat research. These innovations empower researchers and breeders with sophisticated resources to access, analyse, and manipulate the complex architecture of the wheat genome. Integrating these capabilities is vital for developing better cultivars adapted to diverse agro-climatic environments. The efficient generation and application of this extensive data will be pivotal in maximising the impact of research efforts.

In this context, elucidating the molecular mechanisms governing early developmental stages is crucial, as increasing temperatures are estimated to profoundly influence vernalisation [14], thereby affecting flowering, particularly meiosis, the cellular process responsible for gamete formation in sexually reproducing organisms, and impacting fertility and grain yield in major crops such as wheat. Chromosome recognition and pairing, which occur at the onset of meiosis, are crucial processes, particularly in polyploid species like wheat. These events begin between homologous chromosomes (equivalent within the same subgenome) at terminal regions, encompassing telomeres and subtelomeres, and may differ from interactions between homoeologous chromosomes (equivalent across different subgenomes) [15]. Following recognition and pairing, chromosomes align and undergo synapsis, facilitating crossing-over, recombination, and the exchange of genetic material between homologous chromosomes [16]. This genetic exchange enhances diversity and generates novel allelic combinations, which are essential for adaptation and crop improvement.

Chromosome recognition, pairing, and recombination between homologous chromosomes in wheat are meticulously regulated processes. Despite its intricate polyploid genome, wheat exhibits diploid-like behaviour during meiosis. It is well-documented that the suppression of homoeologous recombination is controlled by the *Ph1* (Pairing homoeologous 1) locus, situated on chromosome 5B [17–19], which has been recently named *ZIP4-5B* [20]. This locus inhibits recombination between homoeologous chromosomes while facilitating precise recognition and pairing of true homologues. Mutants of *Ph1* have been widely employed as genetic tools in wheat breeding programmes to enable interspecific recombination between wheat chromosomes and those of related species [21, 22]. At the onset of meiosis, during prophase I, homologous chromosomes initiate recognition and association at the chromosome ends [15, 23]. During this phase, chromosomes adopt a spatial arrangement termed the Rabl orientation, characterised by centromeres clustering at one nuclear pole and telomeres at the opposite pole. This configuration promotes the formation of the “telomere bouquet,” a structure wherein chromosome ends aggregate, thereby enhancing the alignment and pairing of homologous chromosomes [23, 24]. Moreover, previous interactions between homologous chromosomes may occur during premeiotic stages, further optimising the efficiency of chromosome recognition and association [25].

The telomere is a sophisticated DNA-protein complex characterised by tandemly repeated DNA sequences that differ across species. The conserved telomeric repeat sequence in plants is 5′-TTTAGGG-3′, initially identified in *Arabidopsis thaliana* [26]. Nonetheless, research in species such as rice has revealed variations in both the sequence and distribution of telomeric repeats among chromosomes, as well as heterogeneity in telomere length [27]. These telomeric repeats are synthesised by telomerase, an enzyme that is critical in averting chromosome degradation and ensuring genome stability. However, telomerase activity is not infallible and may introduce mutations during repeat synthesis, demanding further investigation in the context of telomere dynamics during meiosis and genome evolution. Beyond structural DNA components, telomere-associated proteins play crucial roles in processes such as nuclear architecture, mitosis, and meiosis [28]. This highlights the dynamic nature of plant telomeres, where structural and regulatory elements interact to maintain chromosome integrity across cell divisions. Telomeres may also feature a G-overhang, a single-stranded, guanine-rich 3′ extension at the chromosome terminus, though its presence is not universal. This overhang can facilitate the formation of a protective looped structure known as the t-loop, which safeguards chromosome ends from being mistaken for DNA damage [29, 30]. In addition, telomeric DNA is enriched in guanine nucleotides (nt), promoting the formation of G-quadruplexes (G4), four-stranded secondary DNA structures that deviate from the conventional double helix. These G4 structures are increasingly recognised for their roles in gene regulation and the preservation of DNA stability [31, 32].

Adjacent to the telomeric region lies the subtelomere, a transitional zone distinguished by pronounced genetic variability and intricate sequence composition. Subtelomeric regions exhibit lower conservation than telomeres and are typically enriched with genes, transposable elements (TEs), tandem repeats, and low-complexity sequences [33, 34]. Particularly, these regions frequently contain recombination hot spots, making them crucial for ensuring accurate chromosome pairing and promoting genetic diversity. Beyond their role in recombination, subtelomeres are also critical for genome stability, replication, chromosome dynamics, and transcriptional regulation. Previous research has demonstrated that the absence of subtelomeres can impair chromosome recognition processes, underscoring their indispensable role [35]. Consequently, a thorough investigation of homologous chromosome recognition and pairing in polyploid species such as wheat needs a comprehensive understanding of the chromosomal regions involved, encompassing both telomeres and subtelomeres, as well as their spatial dynamics, DNA sequence specificity, structural characteristics, and the proteins that potentially mediate these processes.

A diverse array of meiotic proteins interacts with chromatin to ensure precise alignment of homologous chromosomes and successful recombination. Although fewer meiosis-specific proteins have been characterised in plants compared to other organisms, research has focused on proteins directly involved in recombination, such as SPO11, MLH1/MLH3, and RAD51 [36–40]. In contrast, the proteins and molecular mechanisms underlying homologous chromosome recognition and pairing remain underexplored. Among the proteins involved in early meiotic events, cohesins, such as REC8, are crucial for maintaining sister chromatid cohesion and facilitating homologous chromosome pairing [41]. ZYP1, a critical component of the synaptonemal complex, promotes synapsis between homologous chromosomes [42], while ASY1 contributes to the initial stages of synaptonemal complex formation, aiding initial homologue alignment [43]. Furthermore, *ZIP4-5B* has been identified as a key gene associated with the *ph1* mutant genotype, influencing homologous pairing in wheat [20]. Another essential element of the cohesin complex is SMC1β, a member of the structural maintenance of chromosomes (SMC) protein family. SMC1β protein is crucial for sustaining stable associations between homologous chromosomes during meiosis I, a prerequisite for accurate chromosome alignment, synapsis, and subsequent recombination events [44]. Given the critical functions of the above-mentioned proteins, investigating their distribution within terminal chromosome regions, especially in polyploid plants, could provide significant insights into meiotic processes, particularly during chromosome recognition and pairing. In wheat, meiosis is specifically intricate due to the interplay of multiple homologous and homoeologous chromosome sets, which complicates recognition, synapsis, and recombination. This complexity presents substantial challenges for the comprehensive analysis of meiotic proteins and their underlying mechanisms.

The main objective of this study is to conduct a detailed molecular characterisation of the terminal chromosomal regions, telomeres and subtelomeres, in both hexaploid and tetraploid wheat. The diploid ancestor *A. tauschii* was also added to this work. Thus, a thorough analysis of multiple sequence-based features encompassing a diverse range of wheat cultivars, focusing on hexaploid wheat, will facilitate a comparative molecular evaluation of these regions at the DNA level. Additionally, an in-silico analysis of the protein targets interacting with DNA sequences in these chromosomal regions was undertaken. This approach will aid in identifying conserved elements and divergent features potentially implicated in the intricate processes of homologous chromosome recognition and pairing within a polyploid genomic framework. The findings from this research could guide the development of advanced genetic tools for wheat breeding programmes, particularly by enabling precise chromosome manipulation to enhance the introgression of agronomically beneficial traits from related species into cultivated wheat cultivars.

## Materials and methods

### Plant material and growing conditions

The bread and durum wheat cultivars used in this study are detailed in Table 1. These wheat lines were employed for both molecular and cytogenetic analyses. Seeds were germinated on moist filter paper in Petri dishes and incubated in darkness at 4 °C for 4–5 days. Subsequently, they were transferred to 25 °C for 1–2 days to promote germination. Seedlings were then cultivated in a greenhouse under semi-controlled conditions, maintaining day/night temperatures of 25 °C/15°C and a relative humidity of 40%.

**Table 1:** Source of the biological material used in the study

Ploidy level	Species	Accession nº	Source
Hexaploid (2n = 6x = 42; AABBDD)	*Triticum aestivum* subsp. *aestivum* cv. LongReach Lancer	PANG0009	JIC^1^
	*T. aestivum* subsp. *aestivum* cv. CDC Landmark	PANG0003	JIC^1^
	*T. aestivum* subsp. *aestivum* cv. SY Mattis	PANG0015	JIC^1^
	*T. aestivum* subsp. *aestivum* cv. CDC Stanley	PANG0004	JIC^1^
	*T. aestivum* subsp. *aestivum* cv. ArinaLrFor	PANG0001	JIC^1^
	*T. aestivum* subsp. *aestivum* cv. Alchemy	W10040	JIC^1^
	*T. aestivum* subsp. *aestivum* cv. Chinese Spring	W9493	JIC^1^
	*T. aestivum* subsp. *aestivum* cv. Norin-61	TRI 7199	IPK^2^
	*T. aestivum* subsp. *aestivum* cv. Spelt	PI190962	CIMMYT^3^
	*T. aestivum* subsp. *aestivum* cv. Jagger	PANG000	JIC^1^
	*T. aestivum* subsp. *aestivum* cv. Fielder	W10146	JIC^1^
	*T. aestivum* subsp. *aestivum* cv. Mace	PANG0011	JIC^1^
	*T. aestivum* subsp. *aestivum* cv. Julius	PANG0007	JIC^1^
	*T. aestivum* subsp. *aestivum* cv. Renan	BW 52,170	CIMMYT^3^
Tetraploid (2n = 4x = 28; AABB)	*Triticum turgidum* subsp. *durum* cv. Capelli	WAT1180351	JIC^1^
	*Triticum dicoccoides*	TRI 1902	IPK^2^
Diploid (2n = 2x = 14; DD)	*Aegilops tauschii* (squarrosa)	T2220051	JIC^1^

^1^John Innes Centre (JIC); https://www.jic.ac.uk/; Norwich, England

^2^Leibniz Institute of Plant Genetics and Crop Plant Research (IPK); https://www.ipk-gatersleben.de/en/; Gatersleben, Germany

^3^Centro Internacional de Mejoramiento de Maíz y Trigo (CIMMYT); https://www.cimmyt.org/es/; México

## Chromosome preparations of root tip cells in somatic metaphase

Emerging seedling roots, measuring 1–2 cm in length, were excised and treated with 0.05% w/v colchicine at 25 °C for 4 h. Subsequently, the roots were fixed in a solution of 100% ethanol and acetic acid (3:1, v/v) and stored at 4 °C until required for further processing. Chromosome spreads were prepared according to the protocol outlined by [45, 46], with minor modifications. Roots were washed three times, each for 5 min, in 1× enzyme buffer (4 mM citric acid and 6 mM sodium citrate). The meristems were then excised and incubated for 1.5 h in an enzyme mixture comprising 0.5% pectolyase Y23 (Kyowa Chemical Products Co., Ltd.), 1% cellulose “Onozuka” RS (Yakult Pharmaceutical Ind. Co., Ltd.), and 20% pectinase (Sigma) in sterile, nuclease-free water (VWR Life Science).

## Fluorescence in situ hybridization (FISH)

 In situ hybridisation was conducted as previously described [45, 46]. To visualise telomeres, the highly conserved telomeric repeat pAt74, derived from *A. thaliana* [26], was employed. DNA telomeric probe was labelled via nick translation using biotin-11-dUTP (Boehringer Mannheim Biochemicals, Germany), following the manufacturers’ guidelines. The reaction was performed at 15 °C for 90 min in a ThermoBrite Leica thermocycler. Detection of biotin- and digoxigenin-labeled probes was achieved using streptavidin-Cy3 conjugates (Sigma, St. Louis, MO, USA) and FITC-conjugated. Chromosomal DNA was counterstained with 4′,6-diamidino-2-phenylindole (DAPI) and mounted in Vectashield (Vector Laboratories, Burlingame, CA, USA).

Fluorescence signals were observed using a Nikon Eclipse 80i epifluorescence microscope, with images captured by a Nikon CCD camera controlled via Nikon 3.0 software (Nikon Instruments Europe BV, Amstelveen, The Netherlands). Image processing, including adjustments to brightness and contrast, was performed using Photoshop 11.0.2 (Adobe Systems Inc., San Jose, CA, USA).

### DNA sequences analysis and prediction tools

All genome assemblies used in this study are listed in Table 2. In total, 17 hexaploid (bread) wheat cultivars from *Triticum aestivum* were included, encompassing Chinese Spring [47] and cultivars characterised within the wheat pan-genome as part of the 10 + Wheat Genomes Project, namely LongReach Lancer, CDC Landmark, SY Mattis, CDC Stanley, ArinaLrFor, Norin-61, PI190962 (spelt wheat; *Triticum aestivum* subsp. spelta), Jagger, Mace, and Julius [48]. Additionally, the hexaploid wheat cultivars Alchemy (https://www.ncbi.nlm.nih.gov/datasets/genome/GCA_951799155.1/), Aikang58 [49], Fielder [50], Attraktion [51], Kariega and Renan [52], each with their genomes sequenced and assembled into chromosomes, were incorporated into the molecular analysis.

**Table 2:** Genome assemblies used in the study

Species/Cultivar		Ploidy level	Assembly		High confident protein coding genes^1^
			Method	Reference	
*Triticum aestivum* subsp. *aestivum*	LongReach Lancer	Hexaploid (2n = 6x = 42; AABBDD)	NRgene DeNovo Magic 3.0	10wheat_assembly_lancer^2^	High confidence de novo predictions called by PSGB and EI under the frame of the 10 + genome project
	CDC Landmark			10wheat_assembly_landmark1^3^	
	SY Mattis			10wheat_assembly_sy_mattis^4^	
	CDC Stanley			10wheat_assembly_stanley^5^	
	ArinaLrFor			10wheat_assembly_arinaLrFor^6^	
	Alchemy		W2rap and a modified TRITEX pipeline	NIAB Elite MAGIC Alchemy^7^	Not available on Ensembl Plants
	Aikang58		DeNovoMAGICYM v. 2.0	ASM2589588v1^8^	Not available on Ensembl Plants
	Chinese Spring		FALCON v. 0.2.2; DenovoMAGIC v. 2	IWGSC CS RefSeq v2.1; NCBI RefSeq^9^	IWGSC
	Norin-61		NRgene DeNovo Magic 3.0	10wheat_assembly_norin61^10^	High confidence de novo predictions called by PSGB and EI under the frame of the 10 + genome project
	Spelt			10wheat_assembly_spelt^11^	Not available on Ensembl Plants
	Jagger			10wheat_assembly_jagger^12^	High confidence de novo predictions called by PSGB and EI under the frame of the 10 + genome project
	Fielder		Hifiasm (v.0.12) (r304); with a DELL PowerEdge R730 followed by Bandage analysis. Sequence reads from Omni-C were mapped using the TRITEX pipeline.	wheat_cv_fielder_v1_assembly^13^	Not available on Ensembl Plants
	Attraktion		hifiasm v0.14 to generate a primary contig assembly. The pseudomolecule construction was carried out using the TRITEX pipeline.	wheat_cv_attraktion_v1^14^	Not available on Ensembl Plants
	Mace		NRgene DeNovo Magic 3.0	10wheat_assembly_mace^15^	High confidence de novo predictions called by PSGB and EI under the frame of the 10 + genome project
	Julius			10wheat_assembly_julius^16^	
	Kariega		Hifiasm12 (v.0.11); hybridScaffold pipeline (Bionano Solve 3.6); Omni-C reads were incorporated using Juicer tools38 (v.1.6) and 3D-DNA39 (v.180114).	Tae_Kariega_v1^17^	High confidence genes imported from the community GFF3.
	Renan		Long-read sequencing with Oxford Nanopore Technology (ONT) (PromethION platform); Optical mapping with Bionano Genomics and Hi-C data.	Triticum_aestivum_Renan_v2.1^18^	
*T. turgidum* subsp. *durum*; Svevo		Tetraploid (2n = 4x = 28; AABB)	NR-Gene DeNovoMAGICTM pipeline	Svevo.v1; NCBI RefSeq^19^	High confidence genes imported from the community GFF3.
*T. dicoccoides;* Ecotype Zavitan			DenovoMAGIC v. 2	WEW_v2.1; NCBI RefSeq^20^	WEWSeq
*A. tauschii* subsp. *strangulate*; Cultivar AL8/78		Diploid (2n = 2x = 14; DD)	FALCON v. 2	Aet v5.0; NCBI RefSeq^21^	Aet v4

^1^ http://plants.ensembl.org/index.html/^2^ https://www.ncbi.nlm.nih.gov/datasets/genome/GCA_903993975.1/

^3^ https://www.ncbi.nlm.nih.gov/datasets/genome/GCA_903995565.1/^4^ https://www.ncbi.nlm.nih.gov/datasets/genome/GCA_903994185.1/

^5^ https://www.ncbi.nlm.nih.gov/datasets/genome/GCA_903994155.1/^6^ https://www.ncbi.nlm.nih.gov/datasets/genome/GCA_903993985.1/

^7^ https://www.ncbi.nlm.nih.gov/datasets/genome/GCA_951799155.1/^8^ https://www.ncbi.nlm.nih.gov/datasets/genome/GCA_025895885.1/

^9^ https://www.ncbi.nlm.nih.gov/datasets/genome/GCF_018294505.1/^10^ https://www.ncbi.nlm.nih.gov/datasets/genome/GCA_904066035.1/

^11^ https://www.ncbi.nlm.nih.gov/datasets/genome/GCA_903994165.1/^12^ https://www.ncbi.nlm.nih.gov/datasets/genome/GCA_903993795.1/

^13^ https://www.ncbi.nlm.nih.gov/datasets/genome/GCA_907166925.1/^14^ https://www.ncbi.nlm.nih.gov/datasets/genome/GCA_918797515.1/

^15^ https://www.ncbi.nlm.nih.gov/datasets/genome/GCA_903994175.1/^16^ https://www.ncbi.nlm.nih.gov/datasets/genome/GCA_903994195.1/

^17^ https://www.ncbi.nlm.nih.gov/datasets/genome/GCA_910594105.1/^18^ https://www.ncbi.nlm.nih.gov/datasets/genome/GCA_937894285.1/

^19^ https://www.ncbi.nlm.nih.gov/datasets/genome/GCA_900231445.1/^20^ https://www.ncbi.nlm.nih.gov/datasets/genome/GCF_002162155.2/

^21^ https://www.ncbi.nlm.nih.gov/datasets/genome/GCF_002575655.2/

Evolutionary analyses were performed using MEGA11 (https://www.megasoftware.net/) [53]. Phylogenetic relationships among the cultivars and species included in this study were inferred employing the Maximum Likelihood method and the Tamura-Nei model [54]. The tree exhibiting the highest log likelihood (−8479.56) is presented. Initial trees for the heuristic search were automatically generated by applying the Neighbor-Join and BioNJ algorithms to a matrix of pairwise distances estimated using the Tamura-Nei model, followed by selection of the topology with the superior log likelihood value. The tree is drawn to scale, with branch lengths expressed as the number of substitutions per site (indicated above the branches). The proportion of sites with at least one unambiguous base present in at least one sequence for each descendant clade is displayed adjacent to each internal node in the tree. This analysis encompassed twenty genomic sequences of the ZIP4-5B gene, incorporating codon positions 1 st, 2nd, 3rd, and noncoding regions. The final dataset comprised a total of 5261 positions.

All telomeric and subtelomeric sequences examined in this study were obtained from the National Center for Biotechnology Information (NCBI, https://www.ncbi.nlm.nih.gov) (see Table 2). To enable comparisons across species and cultivars, the sequences were oriented from the centromere to the telomere. Furthermore, all short-arm sequences were transformed into their reverse complement strand using an online tool (https://www.bioinformatics.org/sms/rev_comp.html).

Variations of the standard telomeric repeat (TTTAGGG) were detected and visualised using a bespoke Python script developed specifically for this purpose. Furthermore, G4Hunter (https://bioinformatics.ibp.cz/#/analyse/quadruplex) [55, 56] was used to assess the frequency of G-quadruplex-forming sequences. The analysis was conducted using default parameters, specifically a window size of 25 and a threshold value of 1.2.

High-confidence predictions of protein-coding genes were generated using EnsemblPlants (https://plants.ensembl.org) [57]. The resulting gene annotations are summarised in Table 2.

Repeat-associated parameters—including satellite sequences, SSRs, and low-complexity regions (such as A-, AG-, and G-rich domains), along with transposable elements, comprising DNA transposons and retroelements (long terminal repeats [LTRs], short interspersed nuclear elements [SINEs], and long interspersed nuclear elements [LINEs]), were identified using RepeatMasker (Interspersed Repeat Masking Based on Protein Similarity, https://www.repeatmasker.org/cgi-bin/RepeatProteinMaskRequest) and Censor (https://www.girinst.org/censor/index.php). The reference sequence database was restricted to Triticeae species (wheat, barley, and rye).

GC content and the distribution of CpG islands were calculated using EMBOSS Cpgplot (https://www.bioinformatics.nl/cgi-bin/emboss/cpgplot) [58], using the tool’s default parameters. Recombination hot spots, both hot and cold, were predicted using iRSpot-EL (http://bliulab.net/iRSpot-EL/) [59], with all settings left at their default values.

The distribution of sequence motifs associated with hot recombination hot spots, specifically the simple repeat CCGCCGCCG [60], and transposable elements (CTCCCTCC; TTAGTCCCGGTT) was assessed using the MAST (Motif Alignment & Search Tool) algorithm from the MEME Suite v5.5.7 (https://meme-suite.org/meme/tools/mast) [61, 62].

Additionally, putative DNA-binding sites were identified for predicted diploid, tetraploid, and hexaploid wheat proteins homologous to SMC1β cohesin (CCACCAGGTGGC). The analysis was configured to include both direct and reverse complement sequences, with results subsequently merged. An E-value threshold of ≤ 10 was applied, whereby MAST reports all exact or degenerate matches to the query sequence with E-values below this cut-off. Furthermore, only hits with a p-value < 0.0001 were retained in the final output.

Statistical analyses were performed in Python with the appropriate packages (panda, scipy.stats, statsmodels).

## Results

The aim of this study was to investigate sequence variability at the chromosome ends of hexaploid bread wheat across multiple cultivars within a breeding context, to elucidate how chromosomes initiate interactions at the onset of meiosis. The study also included two ancestor tetraploid species (*T. turgidum* subsp. *durum* and *T. diccocoides*) plus the donor of the subgenome D, the diploid *A. tauschii*.

**Fig. 1:**
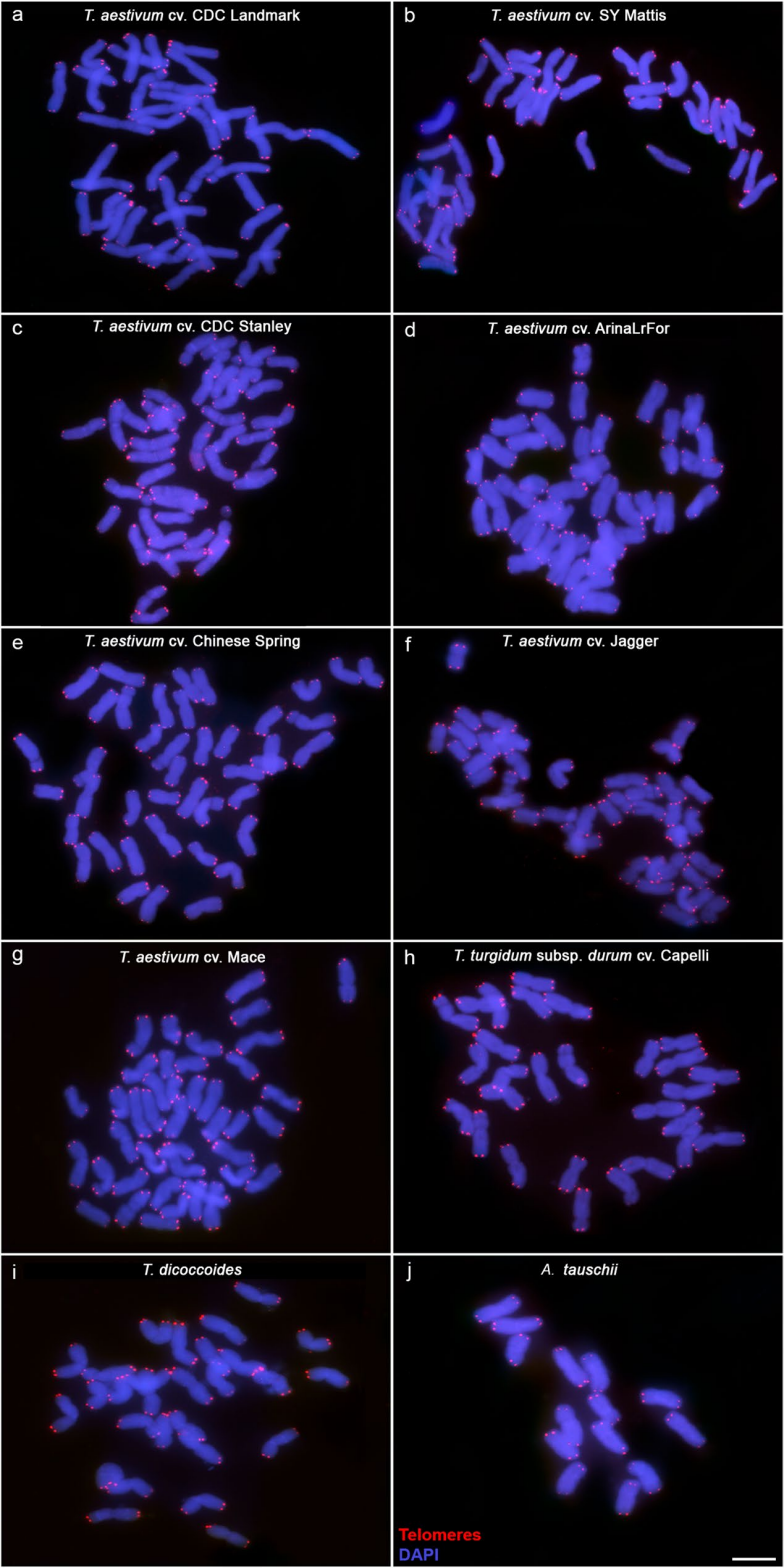
Cytogenetic visualization of telomeres in metaphase chromosomes of hexaploid, tetraploid, and diploid wheat species using fluorescence *in situ* hybridization (FISH). Telomeres were labelled in red; DNA was counterstained with DAPI (blue). Scale bar = 10 μm

Terminal sequences of all chromosomes were examined using the sequence data available. In situ hybridization experiments were performed in somatic metaphase chromosome spreads to visualize telomeres using the conserved telomeric repeat from *A. thaliana*. As expected, all chromosome ends visualized in all the species and cultivars studied contained the telomeric repeat (Fig. 1). However, we found that only some of the chromosome assemblies used in this study contained a stretch of terminal telomeric repeats 5’-TTTAGGG-3’ (Table 3; Additional file 1). For this reason, to the molecular analysis of the telomeric sequences was focused on the chromosome ends whose sequence includes the telomeric repeat.

**Table 3:** Identification of telomere sequences in the chromosome ends of the multiple species and cultivars studied

	Hexaploid (2n = 6x = 42; AABBDD); Triticum aestivum																	Tetraploid (2n = 4x = 28; AABB)		Diploid (2n = 2x = 14; DD)
	LongReach Lancer	CDC Landmark	SY Mattis	CDC Stanley	ArinaLrFor	Alchemy	Aikang58	Chinese Spring	Norin-61	Spelt	Jagger	Fielder	Attraktion	Mace	Julius	Kariega	Renan	T. turgidum	T. dicoccoides	A. tauschii
1AS	108	121						88		107		1390				12,143				
1AL										82						3657				
2AS		89	106	109							50	2684	1015			9292				
2AL																				
3AS			97							82			11,495			9870			69	
3AL																18,882				
4AS					160			85	33	77			4958	66	48				95	
4AL																				
5AS																				
5AL													11,488			12,639				
6AS								64											132	
6AL							364			132			4176			9035				
7AS			124				3256	133		68			3854				2109		86	
7AL						33	135						3665			4519	594			
1BS	100		129							90			1756			9484				
1BL																				
2BS																6397				
2BL																				
3BS			76							48										
3BL													4478			2000				
4BS	91			82										69		9657			94	
4BL																				
5BS																				
5BL																				
6BS								147												
6BL																				
7BS						178		111		118				116	69			131	116	
7BL																				
1DS										82			5398			9906				106
1DL						90														
2DS																18,858				
2DL													1280			14,043				
3DS																				
3DL													12,322			6235				
4DS																				
4DL							5430			88										
5DS																3714				
5DL													16,898			11,335				59
6DS					67															
6DL													6405			11,108				86
7DS	51							85								11,560				
7DL																7783				

Figures represent the length (number of nucleotides) of the telomeric stretch found in some chromosome ends in the diploid (2n = 2x = 14), tetraploid (2n = 4x = 28) and hexaploid (2n = 6x = 42) species and cultivars analyzed. The different genomes/subgenomes are marked in different colours: subgenome A (blue); subgenome B (orange); subgenome D (green).)

**Fig. 2:**
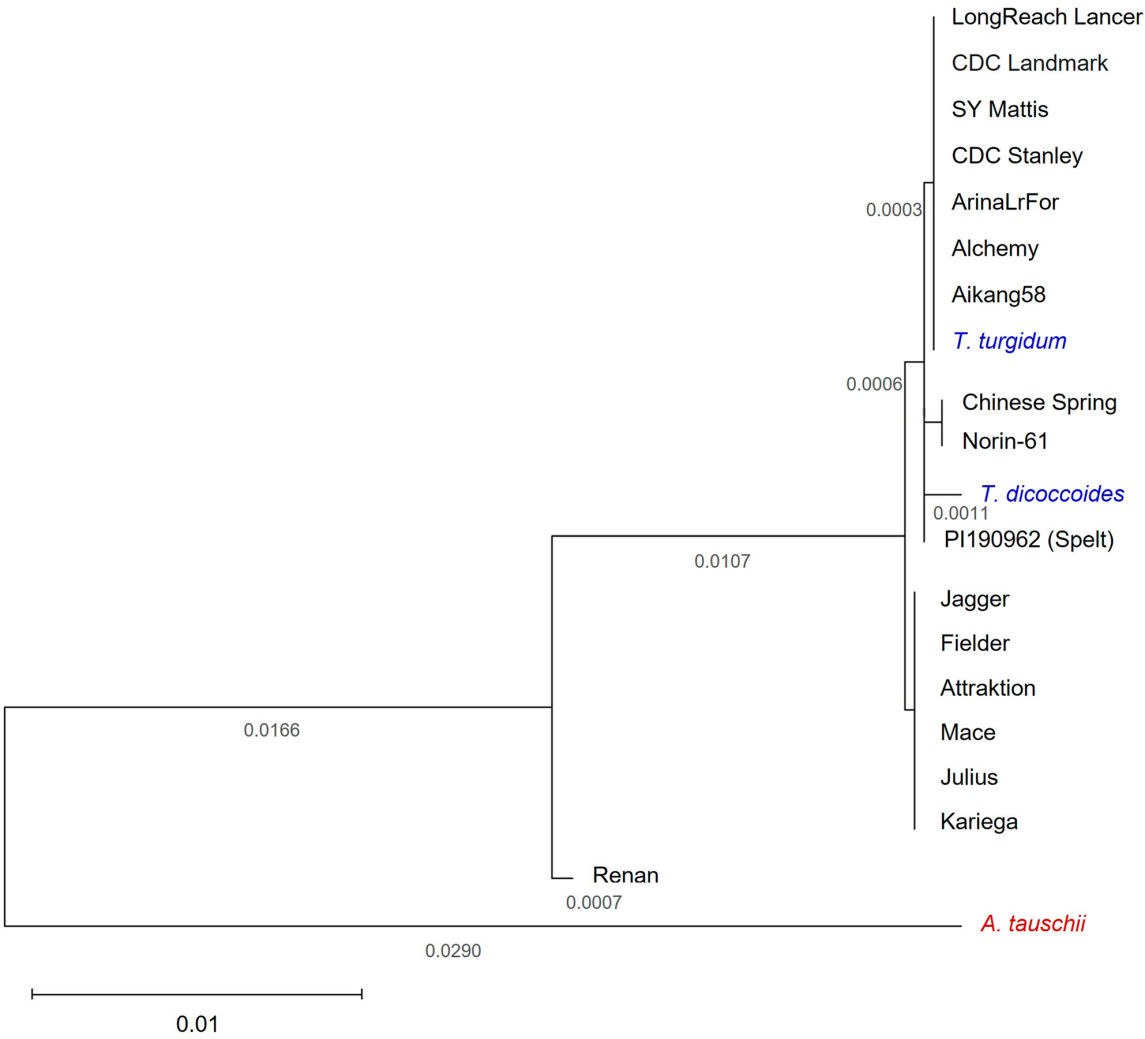
Phylogenetic tree showing the relationships among the wheat species and cultivars included in this study. Hexaploid cultivars are shown in black, tetraploid in blue, and diploid in red. This analysis encompassed twenty genomic sequences of the *ZIP4-B2* gene, incorporating codon positions 1 st, 2nd, 3rd, and noncoding regions. The final dataset comprised a total of 5261 positions

All the species and cultivars studied were phylogenetically ordered (Fig. 2). The phylogenetic analysis presented here is based solely on the ZIP4-5B gene, which plays a central role in meiosis and homologous chromosome pairing in wheat. The diploid species *A. tauschi* represents the most distant species. *Triticum aestivum* cv. Renan diverges as an independent branch, and a different branch includes the rest of tetraploid and hexaploid species and cultivars, which are organized in several branches. The first one includes the *T. aestivum* cultivars LongReach Lancer, CDC Landmark, SY Mattis, CDC Stanley, ArinaLrFor, Alchemy, Aikang58 and the tetraploid *T. turgidum* (both domesticated species share A and B subgenomes). The next branch includes the cultivars Chinese Spring and Norin-61. A third branch is shown below for *T. dicoccoides*, and *T. aestivum* cv. PI190962 (Spelt) represents an independent fourth branch. Finally, a branch is shared by the *T. aestivum* cultivars Jagger, Fielder, Attraktion, Mace, Julius and Kariega.

### Telomere analysis

We continued our study by analysing the sequences of all chromosome ends in the different cultivars and species whose genomes were sequenced and assembled into chromosomes. The 17 hexaploid bread wheat cultivars, together with 2 tetraploid species and the diploid species, make up a total of 784 different chromosome arms, but only 96 chromosome arms contained telomeric sequences (Table 3). We focused on the telomere and subtelomeric regions of these 96 chromosome ends, and we discarded the analysis of the chromosome ends lacking telomeric sequences. Subgenomes A, B and D were represented by 49, 23 and 24 chromosome arms, respectively.

Analysis of the assembled telomeric region of the chromosomes in the different cultivars and species revealed a high variability both in the telomere length and its sequence. The length ranged from 18.882 base pairs (bp) in chromosome 3AL of *T. aestivum* cv. Kariega to just 33 bp in chromosome 4AS of *T. aestivum* cv. Norin-61 or chromosome 7AL of *T. aestivum* cv. Alchemy (Table 3).

We performed an exhaustive analysis of the telomere nucleotide sequence in the multiple species and cultivars. This analysis revealed changes and variations in the typical plant telomeric sequence repeat TTTAGGG. These telomeric repeat variants ranged from around 3.81 (subgenome D of hexaploid species) to 8.63% (subgenome B of tetraploid species) of the total number of telomeric repeats, depending on the species and the subgenome (Table 4), being the highest occurrence in subgenome B. Regarding the type of variant, substitutions (0.17–1.44%) were generally less frequent, despite some exceptions (for example, 6.25% in 4AS Julius; Additional file 2). Additions (0.87–2.88%) contribute more significantly than substitutions, and deletions (2.22–4.32%) are the dominant type of variant. Differences among subgenomes and species/cultivars were statistically significant (*p* < 0.00001).

**Table 4:**
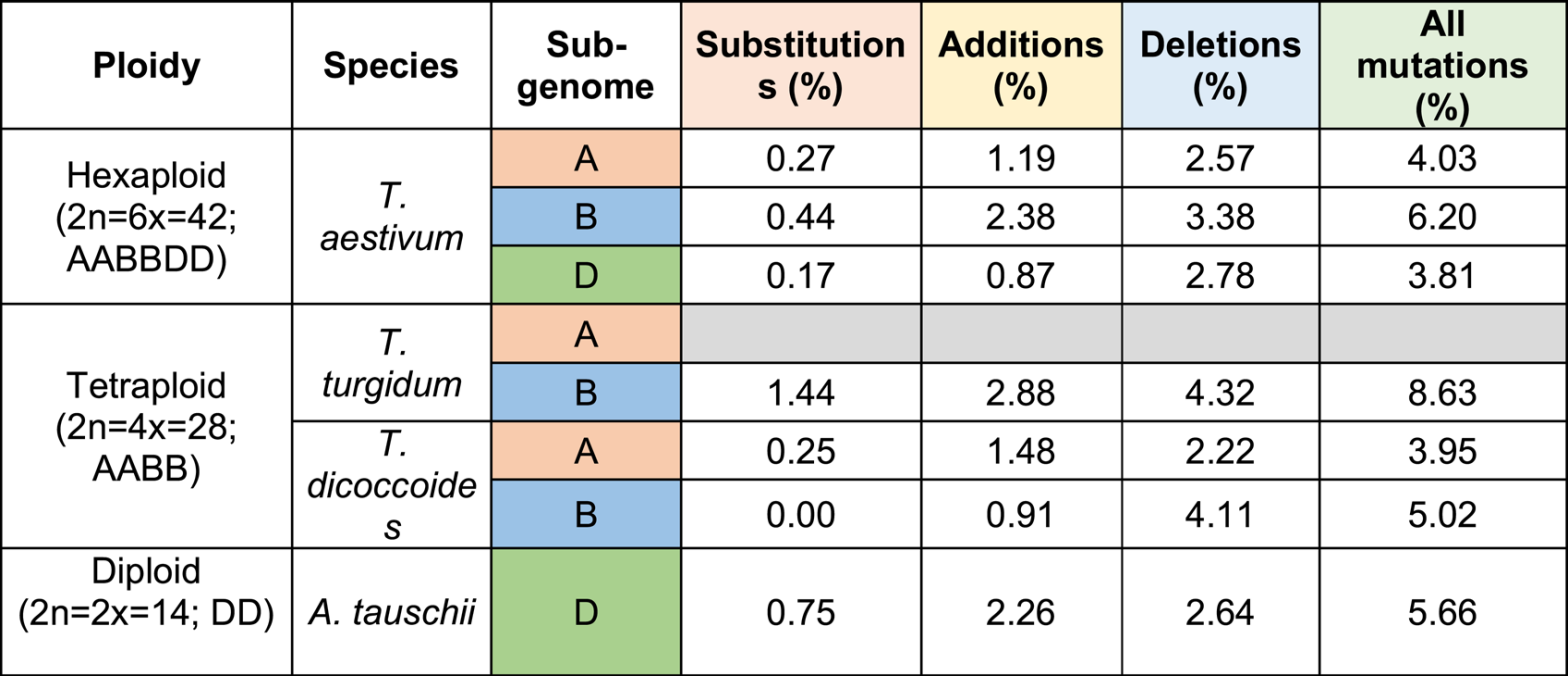
Abundance of mutations of the telomere sequence repeats

Figures represent the percentage of total telomere repeats that contain a mutation

The differential effects of cultivar/species, subgenome, and chromosome on telomere variant density in wheat are robustly supported by a dataset of 96 observations spanning 20 cultivars/species, 3 subgenomes (A, B, D), and 14 chromosome ends, via 3-way ANOVA (Additional file 2). The analysis revealed that species/cultivar exerted the most significant influence, accounting for 26.3% of the variance (*p* < 0.01), with notable differences among cultivars. Chromosome followed, explaining 17.7% of the variance (*p* < 0.01). Subgenome had the smallest main effect, contributing 7.8% of the variance (*p* < 0.01).

We found multiple variants of the standard telomeric repeat, including simple deletions, additions and substitutions, double additions, double deletions, substitutions, and other more complex variants. Table 5 shows all the different variants and their respective abundances. The most abundant variants include single deletions and single insertions of a T or a G, representing nearly 85% of all the mutations detected. With a 60.2%, TTAGGG is the most abundant single variant of the telomeric repeat. Rare variants, those with relative frequencies below 1%, are numerous (31 out of 40) but have a small cumulative contribution to the total. For instance, TTTGTG, TGTGTAGGG and TTTAGGGC have a minimum frequency of only 0.01%.

**Table 5:** Relative abundance of telomere sequence variants

Telomere variant	Genome (%)	Subgenome A (%)	Subgenome B (%)	Subgenome D (%)
gTTAGGGt	60.198	59.272	61.125	60.744
gTTTAGGt	9.491	10.096	8.610	9.203
gTTTTAGGGt	8.858	7.864	7.161	10.106
gTTTAGGGGt	6.144	6.323	5.115	6.253
gTTAGGt	2.967	2.869	3.836	2.818
gTTTGGGt	1.890	2.524	1.449	1.475
gTTTTTAGGGt	1.351	1.461	2.131	1.057
gTTGGGt	1.130	1.382	1.194	0.903
gTTAGGGGt	1.119	1.010	1.449	1.123
gTTTTGGGt	0.950	0.956	0.938	0.947
gTTTAAGGt	0.855	0.584	0.767	1.145
gTTTAAGGGt	0.697	0.584	0.597	0.815
gTGTAGGGt	0.644	0.717	1.194	0.440
gTAGGt	0.443	0.452	0.597	0.396
gTTTGGGGt	0.412	0.478	0.597	0.308
gTGTTAGGGt	0.370	0.345	0.767	0.286
gTTTAGGGGGt	0.243	0.266	0.256	0.220
gTTGGGGt	0.243	0.345	0.171	0.176
gTTAAGGGt	0.232	0.266	0.171	0.220
gTTAGt	0.232	0.319	0.171	0.176
gTTTTTTAGGGt	0.201	0.266	0.171	0.154
gTTTAGGAt	0.179	0.266	0.000	0.154
gTTTTGGt	0.158	0.292	0.085	0.066
gTATGGt	0.148	0.080	0.085	0.220
gTTTTGGGGt	0.127	0.159	0.256	0.066
gTTGAGGGt	0.095	0.106	0.171	0.066
gTTTACGGt	0.084	0.053	0.341	0.044
gTTTAGGCt	0.084	0.106	0.000	0.088
gTTTATGt	0.074	0.133	0.000	0.044
gTAAGGGt	0.063	0.106	0.000	0.044
gTTTTTAGGCt	0.053	0.000	0.426	0.000
gTTTCGGt	0.053	0.000	0.085	0.088
gTTTAGAGt	0.053	0.133	0.000	0.000
gTTTAGGAGt	0.042	0.080	0.000	0.022
gTTTTGt	0.032	0.027	0.000	0.044
gTTAAGGGGt	0.032	0.000	0.000	0.066
gTTATGGt	0.021	0.000	0.085	0.022
gTTTGTGt	0.011	0.027	0.000	0.000
gTGTGTAGGGt	0.011	0.027	0.000	0.000
gTTTAGGGCt	0.011	0.027	0.000	0.000

The relative frequencies of all the variants in the telomeric repeat sequence were similar when comparing the whole genome and A, B, D subgenomes, and among different cultivars and chromosomes, though some differences were detected. In subgenome A, the relative abundance of variants is similar to the whole genome, with the dominant variant being TTAGGG (59.27%). As a notable variant, TTTAGG (10.10%) is slightly more frequent in A subgenome than in the others. Rare variants such as TTTAGGA (0.27%) have a higher representation in subgenome A than in subgenomes B and D. Subgenome B exhibits hot spots for some less frequent variants. It has the highest proportion of the dominant variant (TTAGGG, 61.13%). It is relevant that rare and more complex variants such as TGTAGGG (1.19%) and TTTTTAGGC (0.43%), and TTTACGG (0.34%) are more representative in this subgenome compared to A and D. Some variants are absent (for example, TTTAGGA; TTTAGGC; TTTATG; TAAGGG; TTTAGAG; TTTAGGAG; TTTTG; TTAAGGGG; TTTGTG; TGTGTAGGG; TTTAGGGC, 0%). Finally, subgenome D shows greater relative diversity. The dominant variant (TTAGGG, 60.74%) has a similar frequency than in the other subgenomes. The variant TTTTAGGG (10.11%) is more frequent in this subgenome than in A and B, indicating a possible differential accumulation of mutations. Rare variants such as TATGG (0.22%) have a slightly higher representation.

When analysing the distribution of telomere repeat variants in the telomere sequence, we found them in all telomeres analysed. Taking all the telomere sequences and all the cultivars and subgenomes, a linear correlation was found between the distance from the telomere start and the number of variants (Number = 0,0387×Distance, R^2^ = 0,885), the slope representing the average frequency of variants. When specific subgenomes were analysed, we obtained the following correlations: subgenome A (Number = 0,0390xDistance, R^2^ = 0,927), subgenome B (Number = 0,0590×Distance, R^2^ = 0,859, and subgenome D (Number = 0,0351×Distance, R^2^ = 0,917).

The density of variants along the telomere evolves inversely. We found a heterogeneous distribution, with regions of higher and lower densities, but an overall higher abundance in the proximal region near the subtelomere when considering the complete set of chromosomes, cultivars and subgenomes. The overall regression equation is Density = − 0.00032×Distance + 7.2 (R^2^ = 0.41, *p* < 0.001). When analysing proximal (< 2000 nt) and distal regions (> 2000 nt) of the telomere separately, we found that the density of variants had a different evolution. For proximal regions, Density = − 0.0043×Distance + 10.1 (R^2^ = 0.71). For terminal regions, Density = − 0.0012×Distance + 4.5 (R^2^ = 0.54). This means that the density of mutations is four times higher at the proximal region. An identical overall behaviour was found in all the subgenomes in hexaploid wheat, and in tetraploid and diploid species. However, specific behaviours were found in certain chromosomes and cultivars (i.e., in chromosome 1AS, the density ranges from 2.81% in Kariega to an anomalous 8.26% in Landmark in the proximal region). Some cultivars have relatively constant densities between proximal and distal telomere segments, or even higher density in distal telomere when compared to proximal telomere. As an example, Kariega 3AS shows densities of 2.10 and 2.76% for proximal and distal regions, respectively. In general, differences between subgenomes are more pronounced than differences between cultivars, indicating that genomic structure has a greater impact on telomeric mutation accumulation than cultivar-specific adaptations.

Regarding the evolution of the diverse types of variants along the telomere, we found a general decreasing density towards the distal telomere. Substitutions (*p* < 0.001) and additions (*p* = 0.002) are more frequent in proximal regions of the analysed telomeric sequences. Additions are more frequent in specific subgenomes (notably in subgenome B) and have a more uniform distribution than substitutions, though their density decreases more abruptly towards the distal region. Deletions are the most frequent and dominant type of mutation along the telomere, and their density decreases significantly towards the distal region.

**Fig. 3:**
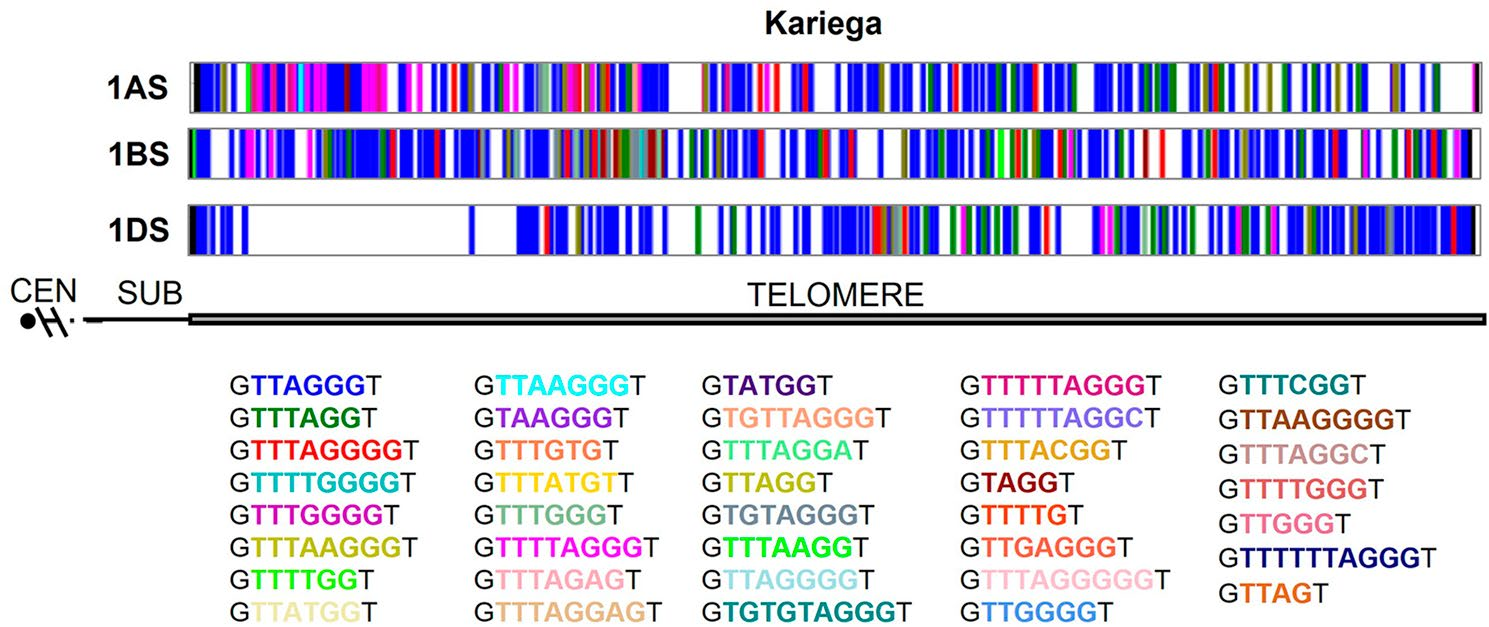
Distribution of the telomeric sequence repeat variants along the telomere of homoeologous group 1 S in *Triticum aestivum* cv. Kariega. The x-axis indicates a position in base pairs (bp) within the telomeric sequence. Variants are shown using a colour bar as defined in the legend. Chromosome arms are oriented from the centromere toward the telomere

The distribution of variants along the telomere is very heterogeneous. Mutations tend to cluster in certain regions. There seem to be periodic bursts of mutations followed by relatively stable regions and even regions (gaps) that are free of mutations (Fig. 3). The overall average size and density of gaps larger than 28 nt were determined. The overall average gap was 37.31 ± 20.55 nt, and the overall density was 11.13 ± 1.39 gaps/kb. For subgenome A, the average gap was 38.34 ± 19.92 nt, with a density of 10.92 ± 1.49 gaps/kb, showing high variability in gap size due to outliers like Renan (150.6 kb). Subgenome B had the highest average gap at 40.57 ± 16.84 nt and a density of 10.50 ± 2.17 gaps/kb, with the greatest variability in density among subgenomes. Subgenome D exhibited the lowest average gap at 34.45 ± 10.03 nt, indicating more consistency, and the highest density at 11.69 ± 1.34 gaps/kb, with the least variability in density. These results suggest subgenome B tends toward larger, more variable gaps, while D has smaller, more uniform gaps and slightly higher, stable gap densities, with A being intermediate but influenced by extreme values.

Two 4-way ANOVA analyses were conducted to evaluate the effects of cultivar, chromosome, subgenome, and distance (distance from the telomere start) on average gap size and density of gaps larger than 28 (density). For average gap, distance was the dominant factor, explaining 28.4% of the variance (*p* < 0.01), with shorter distance showing larger gaps (e.g., Short: 44.27 vs. Long: 33.27), followed by cultivar at 20.0% (*p* < 0.01) (e.g., Renan: 150.6 vs. Aikang58: 16.24), chromosome at 13.0% (*p* < 0.01), and subgenome at 6.2% (*p* < 0.01) (B: 40.66 > A: 37.52 > D: 31.36). For density, distance led with 23.7% (*p* < 0.01), increasing with distance (distal telomere: 12.04 vs. proximal: 9.58), followed by cultivar at 18.8% (*p* < 0.01) (e.g., Kariega: 11.55 vs. Renan: 5.5), chromosome at 13.8% (*p* < 0.05), and subgenome at 10.7% (*p* < 0.01) (D: 11.39 > A: 10.92 > B: 10.58. These results underscore distance and cultivar as primary drivers, with average gap decreasing and density of gaps larger than 28 nt increasing with distance, reflecting structural influences on gap distribution in wheat telomeres.

The distribution of these large gaps was also analysed. We quantified the average separation between gaps (mutant variant-free regions). The average separation between gaps is ~ 214–257 nt. A 4-way ANOVA was performed to assess the effects of telomere distance, cultivar, chromosome, and subgenome on average separation between gaps (variants free regions larger than 28 nt). Subgenome was the most influential factor, explaining 28.5% of the variance (*p* < 0.01), with subgenome B showing the largest separation (340.59 nt) compared to D (267.96) and A (171.99), followed by telomere distance at 22.0% (*p* < 0.01), distal telomere regions averaged 552.79 nt *versus* 132.95 for proximal ones. Cultivar contributed 19.9% (*p* < 0.01), with Kariega at 270.98 nt *versus* Fielder at 131.97, and chromosome accounted for 13.0% (*p* < 0.01), with chromosome 6 at 283.61 nt *versus* chromosome 1 at 222.38. These findings highlight subgenome and distance as primary drivers of gap separation variation, with cultivar and chromosome also playing substantial roles in wheat telomeres.

**Fig. 4:**
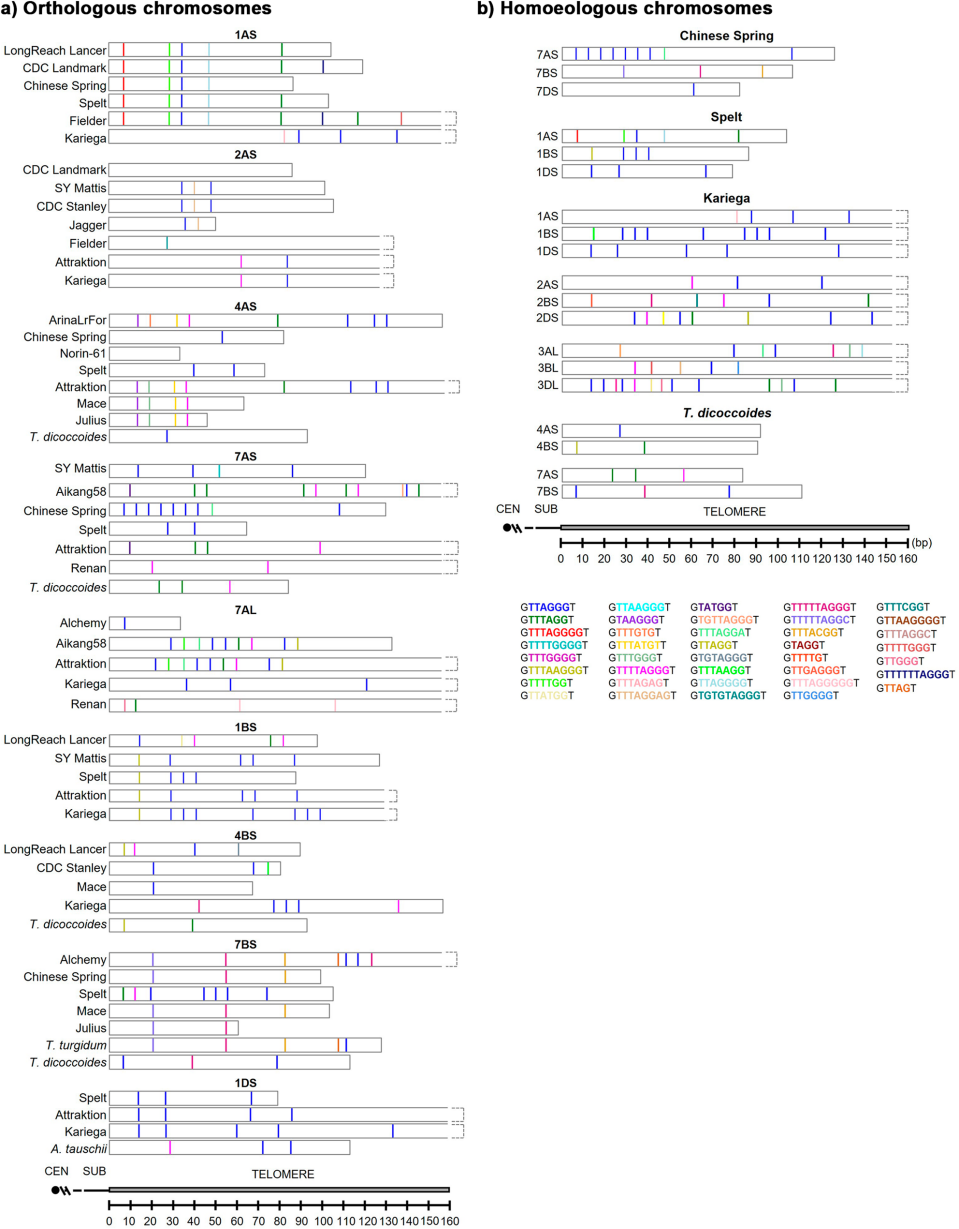
Distribution of telomeric sequence repeat variants along the telomere: (**a**) Orthologous chromosomes; (**b**) Homoeologous chromosomes X-axis: Position in base pairs (bp); Colour bar: Variant types. Chromosome arms are oriented from the centromere toward the telomere end

When comparing the distribution pattern of telomere repeat variants among bread wheat cultivars in the same chromosome, we found a high similarity in phylogenetically closer bread wheat cultivars. Some of these variants are fairly conserved among bread wheat cultivars for the same chromosome arm (Fig. 4a) and between phylogenetically related species *T. aestivum* and *T. turgidum*. Variants are more conserved among phylogenetically closer cultivars. However, the pattern of variations among homoeologous chromosomes from the same cultivar was completely different (Fig. 4b), suggesting a putative contribution of telomeres in chromosome specificity.

### G-Quadruplexes in telomeres

The density and distribution of predicted G-Quadruplexes (G4) were also analysed in all the telomeres (Additional file 3). The average density of G4s is 76.6 ± 23.6 (per kb). We found a change in the density of predicted G4s along the telomere from proximal to distal regions. There is an overall moderate negative correlation, indicating that proximal regions of the telomere tend to have higher G4 densities, while terminal regions tend to have lower G4 densities (*r*=−0.45). This trend is consistent in all three subgenomes, being more relevant in subgenome A and less in D, though differences among them are not statistically significant (*p* = 0.82). We also compared the density of G4s among heterologous chromosomes, subgenomes and species/cultivars. A 4-way ANOVA was conducted to evaluate the effects of telomere distance, species/cultivar, chromosome, and subgenome on the density of G4s. Telomere distance emerged as the dominant factor, explaining 31.3% of the variance (*p* < 0.01), with distal telomere regions showing higher G4 density (84.43 G4/kb) compared to proximal regions (67.07 G4/kb), followed by species/cultivar at 22.3% (*p* < 0.01), with notable differences (e.g., Renan: 121.55 vs. Mace: 56.13). Chromosome contributed 16.9% (*p* < 0.01), varying from 106.3 G4/kb (6AS) to 51.76 (4AS), and subgenome accounted for 5.8% (*p* < 0.01), with B (79.61) slightly higher than A (73.56) and D (74.42). These results highlight distance and species/cultivar as the primary drivers of G4 density, with chromosome and subgenome playing lesser but significant roles, reflecting structural and genetic influences on G4 distribution in wheat telomeres.

**Fig. 5:**
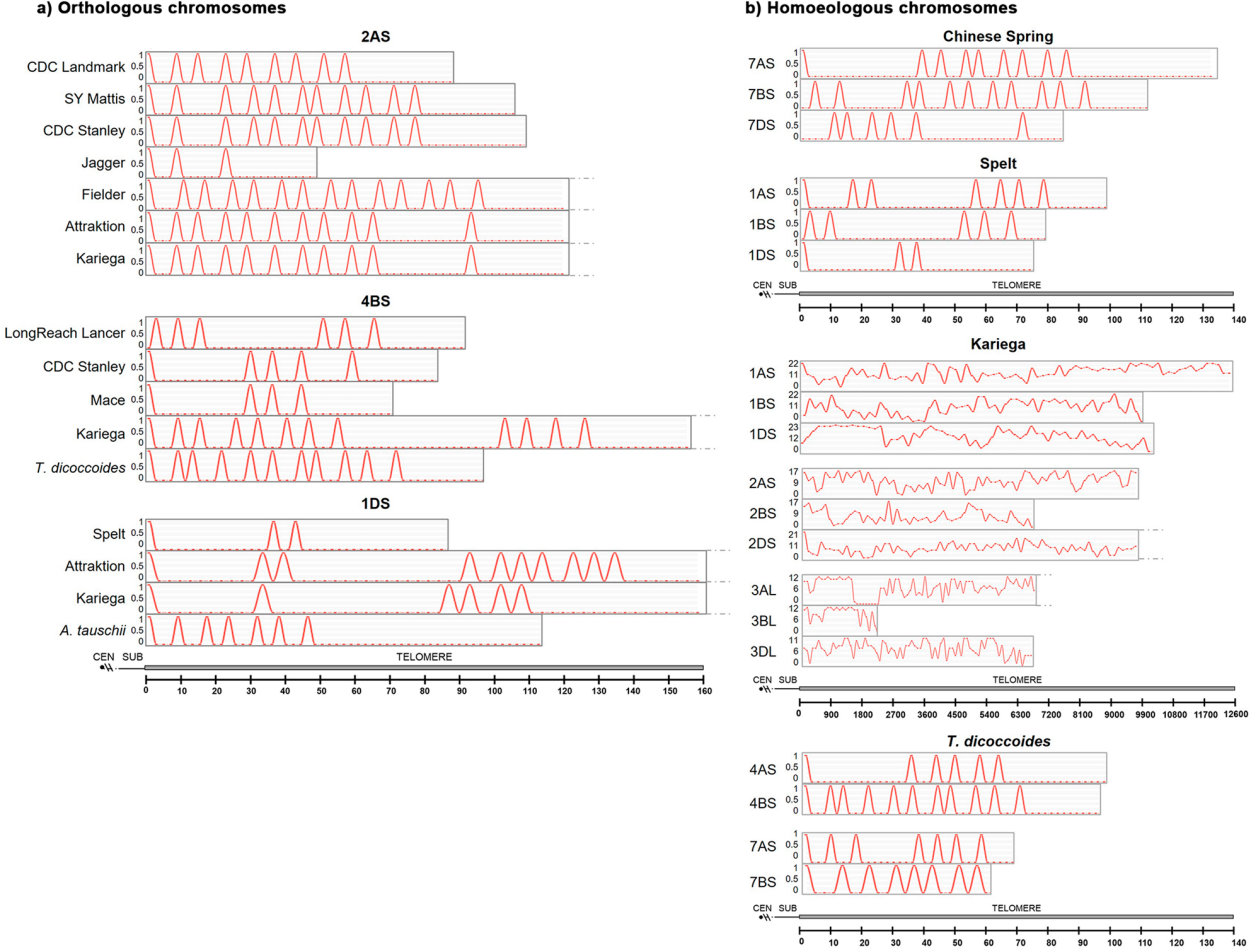
Distribution of G-quadruplexes (G4s) within telomeric sequence repeats: (**a**) Orthologous chromosomes; (**b**) Homoeologous chromosomes Y-axis: Number of quadruplexes (count); X-axis: Position along the telomere (bp). Chromosome arms are oriented from the centromere toward the telomere end

Particularly relevant were the comparisons in the G4s distribution patterns among species/cultivars and among subgenomes (Fig. 5; Additional file 4). As expected, we found similarities in the G4s pattern among some of the hexaploid cultivars. The pattern in the tetraploid *T. turgidum* was more similar to the hexaploid cultivars than to its ancestor *T. dicoccoides*. In addition, *T. dicoccoides* and *A. tauschii* have a species-specific distribution pattern not shared with the hexaploid cultivars analysed. As for the analysis of homoeologous chromosomes, we found no similarities among them in all the chromosomes analysed (Fig. 5).

As an exceptional feature, at the end of the long arm of chromosome 3B of the hexaploid cultivar Attraktion, we have identified an extremely degenerate G-rich terminal sequence with a length of 743 nucleotides. In this region, we found no TTTAGGG repeats, and no G4s were detected. Although internal and shorter, a similar region was found in Kariega 1BS.

### Subtelomere analysis

We also analysed the subtelomeres of the same chromosome arms and species/cultivars whose telomeric sequences were studied. Specifically, we focused on the distal 500 Kb of the subtelomeres, investigating key features potentially involved in homologous recognition and pairing. These included G-Quadruplexes (G4), distribution of coding genes, transposable elements (TEs), repeat DNA elements, GC-content and CpG islands, crossover and recombination hot-cold spots and predicted protein-binding sites related to homologous pairing and recombination. For the most relevant features, the analysis was extended to the 5 M distal part of the subtelomere.

#### G-Quadruplexes (G4)

The abundance and distribution of G-Quadruplexes (G4) were analysed in the distal 500 Kb of the subtelomere bordering the telomere (Additional file 5). We examined potential correlations with species/cultivar, chromosome, and subgenome. The average abundance (1,87 ± 0.61 G4s/kb) substantially lower than the observed in the telomere, though the variability remained comparable, about 30%. Slight differences in average density were found among subgenomes, being 1.97 ± 0.55, 1.82 ± 0.39, and 1.76 ± 0.52 G4s/kb for A, B, and D subgenomes.

A 3-way ANOVA with effect size analysis revealed striking differences in how biological factors influence G4s density. Chromosome arm emerged as the dominant factor, since it explains 59% of the total variance (*p* < 0.001), indicating that local genomic architecture plays a crucial role in G4 density. Species/cultivar accounted for 24% of variance (*p* = 0.002), demonstrating that genetic background significantly modulates G4 density patterns. In contrast, subgenome origin (A, B, or D) showed minimal explanatory power, just 1% and was not statistically significant (*p* = 0.56). According to Additional file 5, the density of G4s for most chromosomes between the different hexaploid cultivars, the tetraploid species and the diploid species is similar. However, some hexaploid cultivars with none or very few predicted G4s stand out: Kariega 1AS; 2BS; 2DS; 5DS and Attraktion 6AL.

We analysed the G4s distribution and found a non-random distribution, volatile clustering, a higher density in the latter half of the 500 kb sequence near the telomere, and a relevant periodic fluctuation of G4 density with local maxima and minima. We calculated the number of minima and maxima for each of the 96 chromosome arms. On average, the 500 kb distal subtelomeric regions contained 20.35 ± 2.10 minima/maxima, with an average spacing of 22.0 ± 0.2 kb between adjacent minima. Comparable results were obtained when the analysis was extended to 5 Mb of distal subtelomere (number of G4s: 237.60 ± 14.09; average separation: 21.19 ± 2.04 kb). This number of minima/maxima and separation between minima was almost invariant across chromosomes, subgenomes and species/cultivars, except for those cases where very few or no G4s were predicted: Kariega 1AS, 2BS, 2DS, 5DS and Attraktion 6AL. A 3-way ANOVA analysis showed that all parameters have significant effects on the number of minima/maxima, with chromosome variation dominant: chromosome (*p* < 0.01, 45.2%), species/cultivar (*p* < 0.01, 26.2%), and subgenome (*p* ≈ 0.04, 3.6%).

**Fig. 6:**
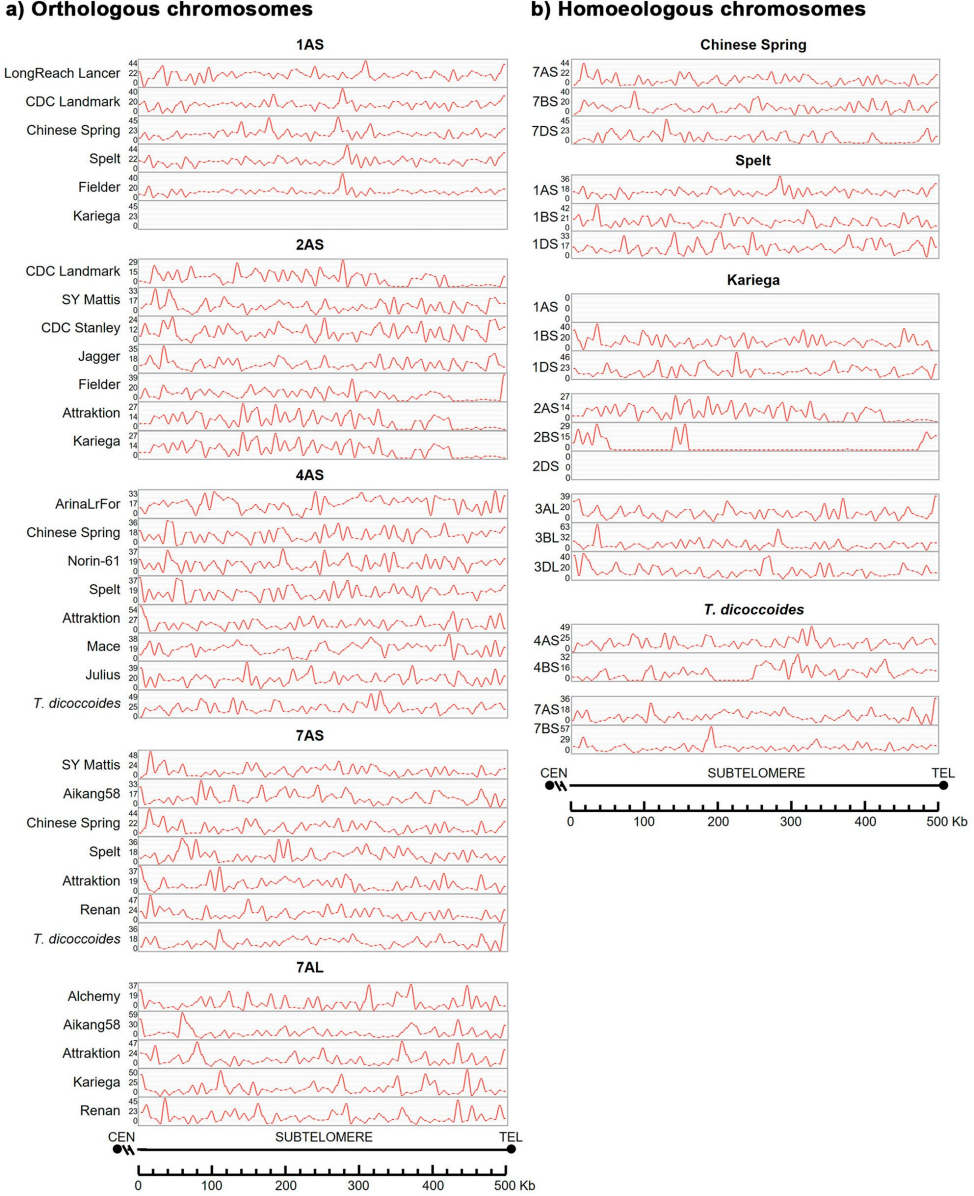
Distribution of G-quadruplexes (G4s) in the distal 500 kb of the subtelomeric region adjacent to the telomere: (**a**) Orthologous chromosomes; (**b**) Homoeologous chromosomes. Y-axis: G4 count; X-axis: Position in base pairs (bp). Chromosome arms are oriented from the centromere toward the telomere

When comparing the distribution of G4s along the subtelomere, we found similar patterns among multiple hexaploid cultivars in several chromosomes. (Fig. 6; Additional file 6). When we compared the hexaploid cultivars and the tetraploid species (*T. dicoccoides* and *T. turgidum*), we found a conservation of the G4 pattern. The pattern of durum wheat is more similar to the hexaploid cultivars than to its tetraploid ancestor (*T. dicoccoides*). For the diploid ancestor analysed, *A. tauschii*, the G4 distribution pattern in all the chromosomes analysed is similar to the hexaploid cultivars. However, the distribution pattern of G4s among homoeologous chromosomes was different.

### Coding genes

The number, density and distribution of genes were also analysed. Highly confident protein coding gene annotation for all the species and cultivars is listed in Additional file 7. For some hexaploid cultivars (Alchemy, Aikang58, PI190962, Fielder and Attraktion) gene annotation was not available in Ensembl Plants (https://plants.ensembl.org/index.html). Due to the importance of this feature the analysis was performed on 500Kb and extended to 5 M of the distal region of subtelomeres.

The overall average and standard deviation of raw gene counts, and their densities were calculated for the distal 500 kb subtelomere and for the distal 5 Mb subtelomere. For the distal 500 kb, the raw average was 7.29 ± 5.35 genes, corresponding to a density of 14.59 ± 10.70 genes/Mb when normalized to the 0.5 Mb region, with the density being exactly twice the raw mean and variability due to the scaling factor, reflecting high heterogeneity (e.g., 0 to 35 genes) in this smaller region. For the distal 5 Mb, the raw average was 89.64 ± 22.73 genes over 5 Mb, yielding a density of 17.93 ± 4.56 genes/Mb, where the density is one-fifth the raw mean and variability, indicating greater consistency across the larger region. Comparing these, densities are very similar in shorter and longer regions, though density in distal 500 kb shows greater variability relative to its mean (CV ≈ 73%) than density in distal 5 Mb (CV ≈ 25%), highlighting that gene distribution is more uniform over the 5 Mb subtelomere than the 500 kb region, likely due to averaging effects over a larger genomic span. Some exceptions with none or just 1 gene in the 500 kb distal region are noticeable: Kariega 1AS, Kariega 1AL, CDC Stanley 2AS; *T. dicoccoides* 4AS, Kariega 1BS, Kariega 2DS, and Kariega 5DS. It should be noted that in chromosome 1AS the number and density of genes is higher both in the distal 500Kb and in the distal 5 M, compared to the rest of the chromosome arms studied. The number and density of genes are different for each chromosome. The phylogenetically closest hexaploid cultivars have a similar number and density of genes (1AS: LongReach Lancer and CDC Landmark; 4AS: Chinese Spring and Norin-61). It should be noticed that *T. turgidum* has several genes more similar to the hexaploid cultivars than to *T. dicoccoides* (Additional file 7).

Gene density varied among chromosomes, subgenomes and cultivars/species. The variability in the density of genes in the distal 500 kb is consistently higher than in the distal 5 Mb across all analysed levels (chromosomes, subgenomes, and cultivars/species). A 3-way ANOVA assessed the effects of chromosome, cultivar/species, and subgenome on gene densities in the distal 500 kb and distal 5 Mb subtelomeric regions. For gene density in distal 500 kb, chromosome dominated, explaining 51.6% of the variance (*p* < 0.01), followed by cultivar/species at 8.45% (*p* < 0.01) and subgenome at 0.97% (*p* < 0.01), with high variability. In contrast, gene density in distal 5 Mb showed lower variability, with chromosome contributing 26.7% (*p* < 0.01), and subgenome 1.74% (*p* < 0.05) to this variability. The greater variability in distal 500 kb gene density (driven by chromosome) *versus* the more uniform gene density in distal 5 Mb (with stronger cultivar/species influence) suggests that gene distribution is highly heterogeneous near telomeres, likely due to localized structural differences, while the broader 5 Mb region reflects more consistent, cultivar-specific patterns, implying different evolutionary or functional constraints across these subtelomeric scales.

**Fig. 7:**
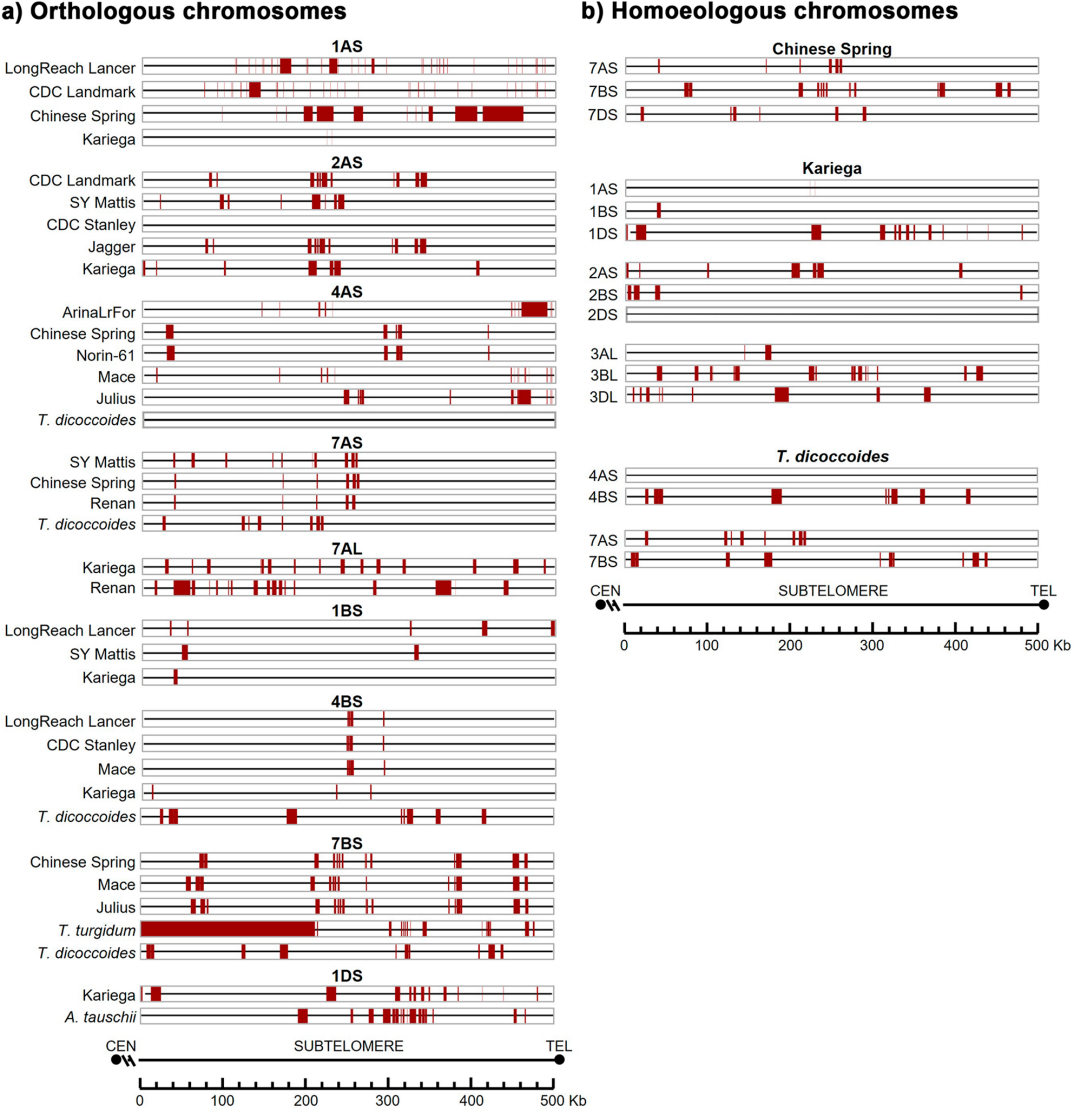
Distribution of high-confidence protein-coding genes in the distal 500 kb of the subtelomeric region adjacent to the telomere ends: (**a**) Orthologous chromosomes; (**b**) Homoeologous chromosomes. Chromosome arms are oriented from the centromere toward the telomere. See Table 2 for annotated gene details

Regarding gene distribution (Fig. 7), the pattern was highly conserved among phylogenetically closely related hexaploid cultivars. For example, in region 4AS between Chinese Spring and Norin-61, and in 7BS between Mace, Julius, and Chinese Spring. This conservation was also observed in more distantly related cultivars, such as in 7AS across SY Mattis, Chinese Spring, and Renan, and in 4BS across Lancer, Stanley, and Mace, in both the 500 Kb and 5 Mb regions analyzed. Conversely, other chromosome arms showed more variable gene distribution patterns in both size windows, particularly among more distantly related cultivars or across different species (data not shown). In sharp contrast, gene distribution patterns were completely different among homoeologous chromosomes, suggesting that these differences may contribute to homolog recognition specificity during the initial stages of meiosis.

As to the nature of the genes located in this region, we found a great diversity, in those cultivars where genes are already tagged. As an example, for the 1AS orthologs, the genes identified across all cultivars encode proteins such as TAR1, uncharacterized proteins, or proteins absent from UniProt. For the 7AS orthologs, the identified proteins include the SnTox1 sensitivity protein, a guanine nucleotide-binding protein alpha subunit, a NAC domain-containing protein, plastocyanin, a protein kinase domain-containing protein, and several uncharacterized proteins. In contrast, homoeologous group 7 shows greater divergence among the three chromosomes. 7AS includes protein kinase domain-containing protein, SnTox1 sensitivity protein, guanine nucleotide-binding protein alpha subunit, NAC domain-containing protein, plastocyanin, uncharacterized protein. 7BS includes Rab-GAP TBC domain-containing protein, ABC transporter domain-containing protein, uncharacterized protein, SLH domain-containing protein, leucine-rich repeat-containing N-terminal plant-type domain-containing proteins (multiple), late embryogenesis abundant protein LEA-2 subgroup domain-containing protein, SnoaL-like domain-containing proteins, F-box domain-containing protein, DEUBAD domain-containing protein, RRM domain-containing protein. And 7DS includes guanine nucleotide-binding protein alpha subunit, NAC047_7D.3, plastocyanin, RING-type E3 ubiquitin transferase, protein N-lysine methyltransferase METTL21A, VWFA domain-containing protein.

### Transposable elements

The presence and distribution of transposable elements (TEs), both retrotransposons and DNA transposons, was also analysed in the distal subtelomeric region adjacent to the telomere in all chromosome arms subject to the study (500 Kb). The retrotransposons or retroelements were grouped according to class into SINEs (Short Interspersed Nuclear Elements), LINEs (Long Interspersed Nuclear Elements) and LTR (Long Terminal Repeat) elements, where we included Copia and Gypsy (Additional file 8). The table shows the total number of TEs, the cumulative length they occupy in base pairs (bp) and their density, expressed as the percentage of the analysed sequence (500 Kb). Across all chromosome arms from the species and cultivars studied, retroelements are consistently more abundant than DNA transposons. The highest TE density was observed in chromosome arm 7BS of *T. dicoccoides*, where TEs account for 52.79% of the 500 kb sequence. In contrast, the lowest TEs content was recorded in chromosome arm 2BS of the hexaploid cultivar Kariega, with a value of 5.49%.

The overall average total number (Nr), total length and density of TE were 252 ± 370, 146 ± 77 kb, and 26.3 ± 12.8%, respectively. The high standard deviation for “Nr” reflects the presence of outliers (e.g., 3661, 2004), indicating significant variability across some cultivars/species. The density (%) of TEs in subgenomes A, B, and D were 26.8 ± 12.6, 31.4 ± 12.8, and 24.0 ± 7.9, respectively. DNA transposons contributed modestly, with an overall density of 3.9 ± 3.8%, and the respective densities in subgenomes A, B, and D were 4.1 ± 4.1, 4.9 ± 4.6, and 3.0 ± 1.7%. In contrast, retroelements were more abundant, with a total density of 22.9 ± 10.6%, distributed as follows: 23.6 ± 11.1% in A, 29.85 ± 9.9% in B, and 16.9 ± 6.0% in D. These results confirm that retroelements are the major contributors to TE content, with subgenome B showing the highest levels and subgenome D the lowest and most consistent. Among retroelements, Gypsy were the most abundant class, with an overall density of 12.3 ± 8.2%. Their distribution across subgenomes was 11.2 ± 7.9% in A, 14.8 ± 8.5% in B, and 10.9 ± 8.0% in D. Again, subgenome B has the highest mean Gypsy content. The overall standard deviation (8.21%) suggests significant variation in Gypsy density across all subgenomes. Copia shows an overall density of 9.0 ± 5.7%. In subgenomes A, B, and D, densities are 9.2 ± 5.8, 9.6 ± 5.7, and 8.0 ± 5.4%, respectively. Subgenome B has the highest Copia content, but the differences among subgenomes are much lower. SINEs are extremely rare, with most entries showing 0%. In contrast, LINES exhibited a different pattern, with an average density of 2.4 ± 3.0% and respective values of 2.9 ± 3.4% in A, 1.9 ± 2.62% in B and 2.3 ± 2.9% in D. Unlike Gypsy elements, LINEs were most abundant in subgenome A, and least in B. Notably, subgenome A showed the highest variability in LINE content, possibly driven by local expansions (e.g., in chromosome 7AS), and Copia dominates in subgenome B. Thus, key patterns emerged: SINEs are negligible; LINEs are most variable in subgenome A; and Copia elements are slightly enriched in subgenome B, suggesting differential retrotransposon activity.

A three-way ANOVA, to assess the effects of chromosome, subgenome, and cultivar/species on TE densities, revealed that the density of most TEs and categories of TEs were affected by chromosome, subgenome and cultivar/species. Chromosome was highly significant for all categories (*p* < 0.001), indicating TE density varies with chromosome, with the strongest effect on LTRs and Gypsy. Subgenome is significant for Total TEs, Retroelements, LINEs, LTRs, Copia, and Gypsy (*p* < 0.001). Subgenome B has higher Gypsy and Copia than A/D (consistent with earlier means). And cultivar/species is significant for Retroelements, LTRs, Copia, Gypsy, DNA transposons, and Total TEs (*p* < 0.001) (example: Kariega has higher Retroelements than Lancer). This analysis of TEs also revealed significant effects on density variance. Three-way ANOVA demonstrated that chromosome identity had the strongest effect on density variance (explaining 38–50% of variance, particularly for Gypsy and LTR elements), while the effect of subgenome was also relevant (it matters most for Gypsy (25%, B > A/D), and LTRs (18%) and Copia content). However, wheat cultivar/species played a modest effect (5–19%), largest for Copia, with Kariega and SY Mattis showing elevated retroelements and LINEs, respectively. Tukey’s tests confirmed subgenome B’s dominance in Gypsy abundance. Gypsy explains up to 85% of the total variance in TEs density among chromosomes, subgenomes and cultivars/species. These findings suggest the pivotal role of chromosomal context and subgenome ancestry in shaping TE dynamics in wheat.

**Fig. 8:**
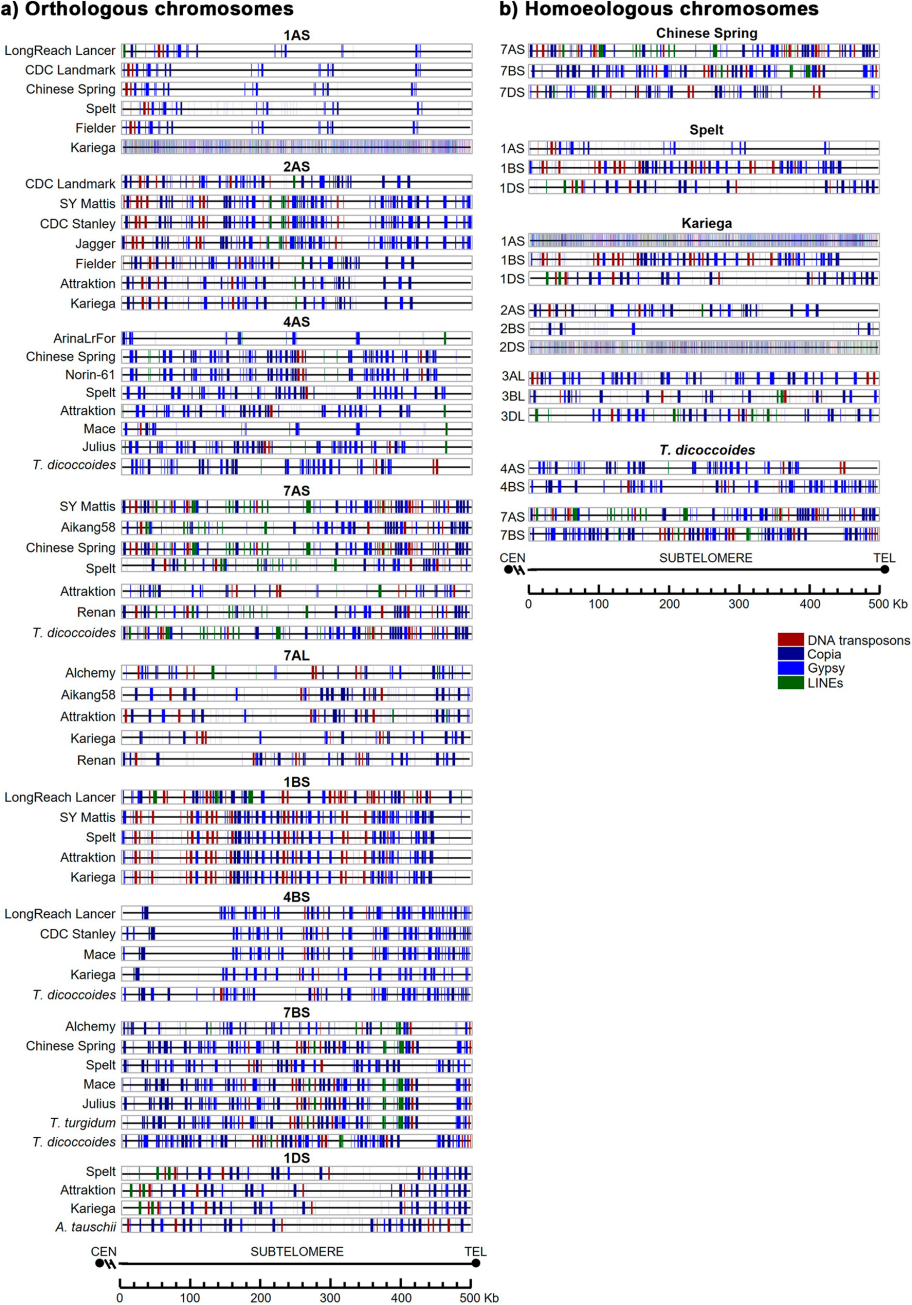
Distribution of transposable elements (TEs) in the distal 500 kb of the subtelomeric region: (**a**) Orthologous chromosomes; (**b**) Homoeologous chromosomes Colour code: Dark red, DNA transposons; Dark blue, Copia; Blue, Gypsy; Green, LINEs. No SINEs were detected in any species analyzed. Chromosome arms are oriented from the centromere toward the telomere

In general, the distribution pattern of TEs within the same chromosome is more similar among closely related cultivars according to the phylogeny, although it is also highly conserved even in more distant cultivars and among hexaploid and tetraploid species (Fig. 8). For example, on 4AS, Chinese Spring and Norin-61 showed equivalent pattern. Similarly, on 1AS, Fielder, CS and Landmark have the same pattern, even though they occupy different branches of the phylogenetic tree. The tetraploid species also have a similar distribution pattern to the hexaploid cultivars. For example, on 7AS, SY Mattis, Chinese Spring, Renan, and *T. dicoccoides* as do *T. turgidum*, Mace, Julius, and Chinese Spring on 7BS. Notably, for most TEs, *T. turgidum* was more similar to the hexaploid cultivars than to *T. dicoccoides* (for example on chromosome 7BS). In addition, *A. tauschii* showed strong similarities in TE distribution pattern with the hexaploid cultivars, particularly on chromosomes 1DS, 5DL, and 6DL. On chromosome 1BS, a common TE distribution pattern is evident among most cultivars, with LongReach Lancer standing out due to its distinctive profile. In some cases, shifts in the TE pattern are observed between species, potentially due to insertions or deletions in the sequence, as seen on 1AS for Lancer and PI190962 (Spelt). To complement the cultivar-focused panel, we also analyzed the TE distribution across the homoeologous group 7 chromosomes (7AS, 7BS, and 7DS) in the hexaploid cultivar *Chinese Spring*. The results reveal striking differences in TE patterns among these chromosomes, highlighting the distinct evolutionary paths of each subgenome and their putative contribution to chromosome specificity at the onset of meiosis (Fig. 8).

### Repeat DNA sequences

We conducted a comprehensive analysis of repetitive DNA elements within distal 500 Kb subtelomeric region close to the telomere across all chromosome arms (Additional file 9). The study included three classes of repeats: satellite repeats, Simple Sequence Repeats (SSRs), and low complexity DNA. Satellite repeats are tandem arrays of short DNA sequence ranging from a single base to several thousand bases. SSRs are short motifs (1–5 bp) repeated in tandem like A, CA, CGG, etc. Low complexity regions are poly-purine or poly-pyrimidine stretches, with extremely high AT or GC content. Remarkably, chromosome 6AL of the *T. aestivum* cultivar Attraktion lacked any identifiable repeated sequences, whereas chromosomes 1AS, 2BS, 2DS and 5DS of the Kariega cultivar have a remarkably high satellite DNA content.

A thorough statistical analysis of repeat sequences revealed the following figures. Across all chromosomes, the total number of repeat elements averaged 280.6 ± 349.2 (mean ± SD), with a mean cumulative length of 62,914 ± 97,123 bp and a mean density of 12.6% ± 19.4%. The density of total repeats was 9.0 ± 20.5, 17.2 ± 23.9, and 11.6 ± 22.6% for subgenomes A, B, D, respectively, indicating greater repetitive element accumulation in subgenome B. Subgenome A shows the lowest mean density, while B has nearly double. Subgenome D was intermediate but more closely aligned with A, suggesting shared evolutionary constraints. Standard deviations are large, reflecting outliers (e.g., Kariega 1AS, with 494,918 bp satellites). Extreme cases included Kariega 1AS (A subgenome) and 2DS (D subgenome), both exhibiting near-total repeat coverage (99.0% and 99.1%, respectively), and Kariega 2BS (B subgenome), which also demonstrated high satellite density (81.9%).

When performing a specific statistical analysis for each type of repeat, we found the following results. For satellites, the overall mean number, length, and density were 35.2 ± 123.5, 8,987 ± 31,421 bp, and 1.8 ± 6,3%, respectively. Satellite density by subgenome was 2.0 ± 7.1% in subgenome A, 1.9 ± 6.8% in subgenome B and 1.4 ± 4.9%, for subgenome D. Subgenome A had the highest satellite content, likely due to residual non-Kariega outliers (e.g., ArinaLrFor 4AS: 41.61%). Subgenome D has the lowest satellite density, consistent with its overall lower repeat content. High SDs indicate variability even after removing Kariega, driven by other high-satellite cultivars.

For SSRs, the overall mean number, length and density were 124.7 ± 35.8, 6,213 ± 1,892 bp, and 1.2 ± 0.4%, respectively. Subgenome B showed slightly higher SSRs density (1.3 ± 0.4%) compared to A (1.2 ± 0.4%) and D (1.2 ± 0.4%), consistent with elevated transposable element activity in this subgenome. Low SDs suggest stable simple repeat distributions across cultivars.

Regarding low Complexity DNA, the overall mean number, length, and density were 24.5 ± 8.9, 1,452 ± 532 bp, and 0.3 ± 0.1%, respectively. Densities were nearly identical across subgenomes A, B, and D (0.3% ± 0.1), exhibiting minimal variation (SD < 0.01%).

Overall, satellites are highest in A (2.0%), lowest in D (1.4%), with extreme variability (high SDs) due to non-Kariega outliers (e.g., ArinaLrFor). SSRs showed differences, having around 10% higher density than A/D, and highly consistent across cultivars (low SDs). Regarding low Complexity, nearly identical across subgenomes (0.28–0.30%), being the least variable repeat class.

We also performed a 3-way ANOVA analysis of the effects of chromosome, subgenome, and cultivar/species on repeat densities. All three factors revealed statistically significant effect on total repeat density (*p* < 0.001). Subgenome explained 32% of variance, chromosome 41% and cultivar/species 7%, demonstrating that chromosomal location and subgenome ancestry are primary determinants of repeat distribution. Satellites exhibited the strongest subgenome effect (47%), particularly in subgenome A due to high-density outliers, while chromosome location accounted for even more variance (52%), while the effect of cultivar was only 18%. SSRs showed a moderate subgenome effect (14%, B > A ≈ D) but no significant cultivar influence. In contrast, low complexity repeats displayed minimal variation across chromosomes, subgenomes and cultivars, with only one chromosome showing a marginal effect (5%). These results suggest that satellite and simple repeat distributions are shaped primarily by chromosomal context and subgenome ancestry, while low complexity repeats are uniformly distributed and largely invariant.

**Fig. 9:**
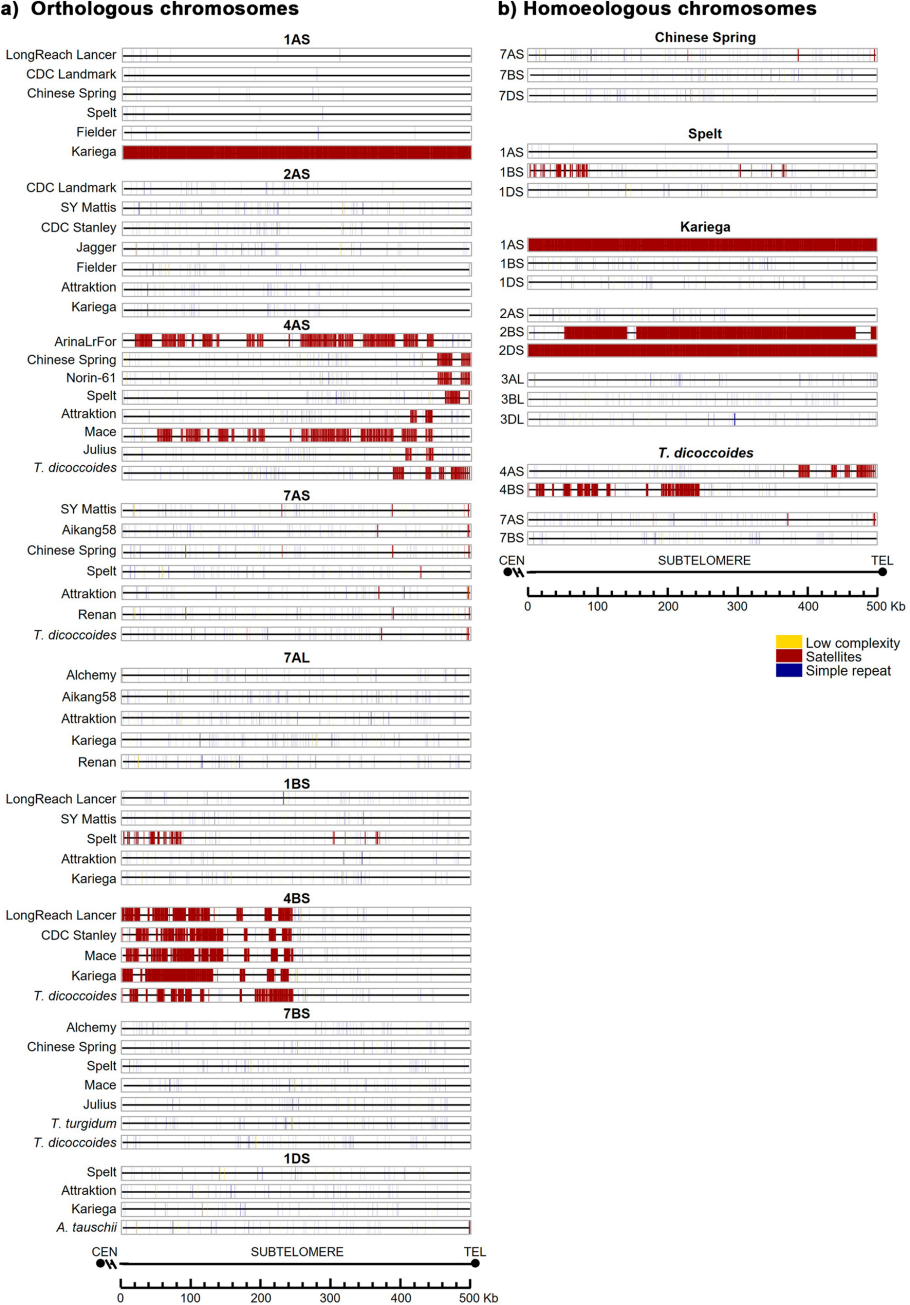
Distribution of repeat elements in the distal 500 kb of the subtelomeric region: (**a**) Orthologous chromosomes; (**b**) Homoeologous chromosomes Colour code: Yellow, low complexity sequences; Dark red, satellite sequences; Dark blue, SSRs. Definitions: *Satellite sequences*: Repeats with unit sizes ranging from 1 bp to several kb. *SSRs*: Simple Sequence Repeats of 2–5 bp motifs (e.g., A, CA, CGG). *Low complexity*: Regions enriched in poly-purine/pyrimidine or high AT/CG content. Chromosome arms are oriented from the centromere toward the telomere

Differences in the distribution pattern of the three types of repeat sequences analysed, especially SSRs and low complexity, were relevant between hexaploid cultivars and between hexaploid/tetraploid species (Fig. 9). The D-genome donor, *A. tauschii*, exhibited unique repeat distribution patterns, particularly on chromosome 1DS. In contrast, satellite DNA distribution showed synteny among closely related hexaploid cultivars (e.g., 4BS), while homoeologues showed divergent repeat organisation (Fig. 9).

### GC and CpG Islands

Distal subtelomere sequences (500 Kb) of all chromosome ends were analysed for the GC content and the identification of predicted CpG islands. The overall mean GC content was 46.02 ± 3.08%. Per chromosome, it ranges from 41.73% (3AS) to 52.88% (1AS), SD varies (0.02–1.22). Per cultivar/species, it goes from 44.71% (SY Mattis) to 48.98% (Fielder), SD varies (0.71–4.32). Per subgenome: A = 46.66 ± 3.68%, B = 46.38 ± 1.22%, D = 45.25 ± 1.81%. A 3-way ANOVA analysis showed that chromosome, cultivar/species, and subgenome all significantly affect GC content, with chromosome arm having the strongest effect (84.09%, *p* < 0.001), while subgenome (3.97%, *p* < 0.001) and cultivar (7.84%, *p* < 0.05) had a milder but still significant impact.

**Fig. 10:**
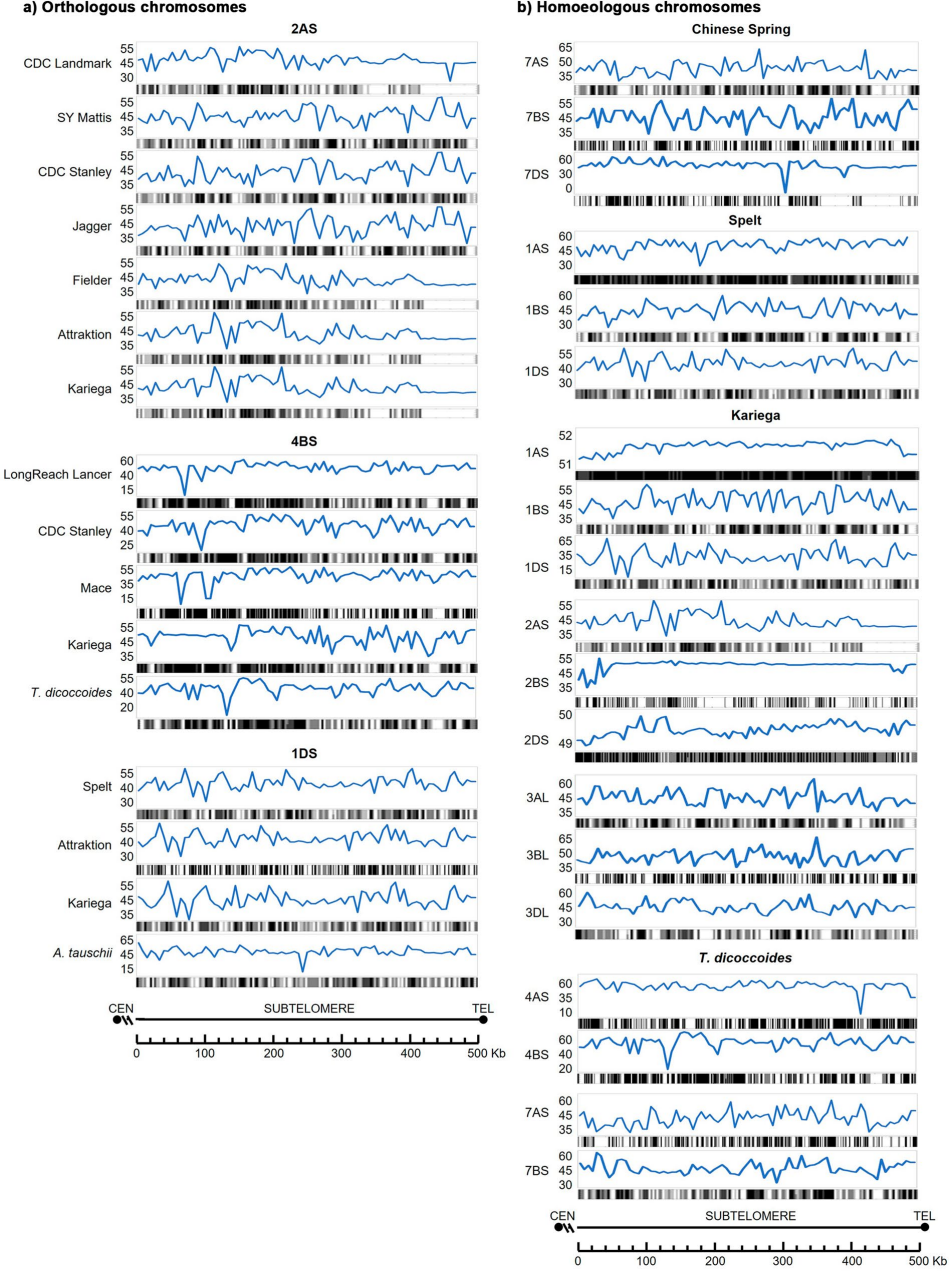
CG content and predicted CpG islands in the distal 500 kb of the subtelomeric region: (**a**) Orthologous chromosomes; (**b**) Homoeologous chromosomes. Y-axis: CG content (%); black-and-white bars: Predicted CpG islands. Chromosome arms are oriented from the centromere toward the telomere

Both GC content and the predicted CpG islands varied significantly among all the chromosome arms analysed. As observed for other features, phylogenetically closer wheat cultivars exhibited more similarities while homoelogues displayed marked differences (Fig. 10, Additional file 10). In all the analyzed hexaploid cultivars, particularly in *Kariega*, chromosome 1AS stands out for its high GC content and a dense concentration of CpG islands in the distal region adjacent to the telomere. Notable differences in GC content and CpG island distribution were found between tetraploid *T. dicoccoides* and the hexaploid cultivars on chromosomes 4BS and some others (3AS, 6AS, data not shown). In contrast, the pattern in tetraploid *T. turgidum* (7BS) more closely resembles that of the hexaploids than its progenitor *T. dicoccoides*. The diploid *A. tauschii* shows a distinct pattern for all the chromosome arms analyzed, compared to the hexaploids (for example, chromosomes 1DS, Fig. 10). The presence or absence of GC- and CpG-rich regions varies depending on the chromosome arm. For example, gaps in GC content and predicted CpG islands are observed in the proximal region of chromosome 6AS, and the most distal region of chromosomes 2AS, 3AS, 5AL, 4DL, and 7DS. Conversely, a higher GC and CpG island density is observed in the proximal region of 4BS and the central subtelomeric region of chromosomes 7AS and 1BS. On chromosomes 2DL and 3DL, the subtelomeric region shows an interspersed pattern with alternating GC- and CpG-rich segments and CpG-depleted regions. Comparison among homoeologous chromosomes 7AS, 7BS, and 7DS in *T. aestivum* cv. Chinese Spring reveals divergent patterns (Fig. 10). Chromosome 7BS shows a more homogeneous CpG island distribution, while 7AS and 7DS exhibit a noticeable reduction in CpG content near the telomeric end of the subtelomeric region.

### Crossover and recombination hot spots

Given the importance of recombination hot spots and crossovers during meiosis for genetic exchange, and the known recombination-rich nature of subtelomeric regions, we have studied recombination cold and hot spots for all chromosome arms in the distal subtelomeric region (500 Kb) adjacent to the telomere. In addition, the distribution of three short sequence motifs (CCGCCGCCGCCG; CTCCCCCTCC; TTAGTCCCGGTT) associated with hot recombination regions in wheat were also analysis in 500 Kb distal subtelomere and within a larger region of 5 Mb.

**Fig. 11:**
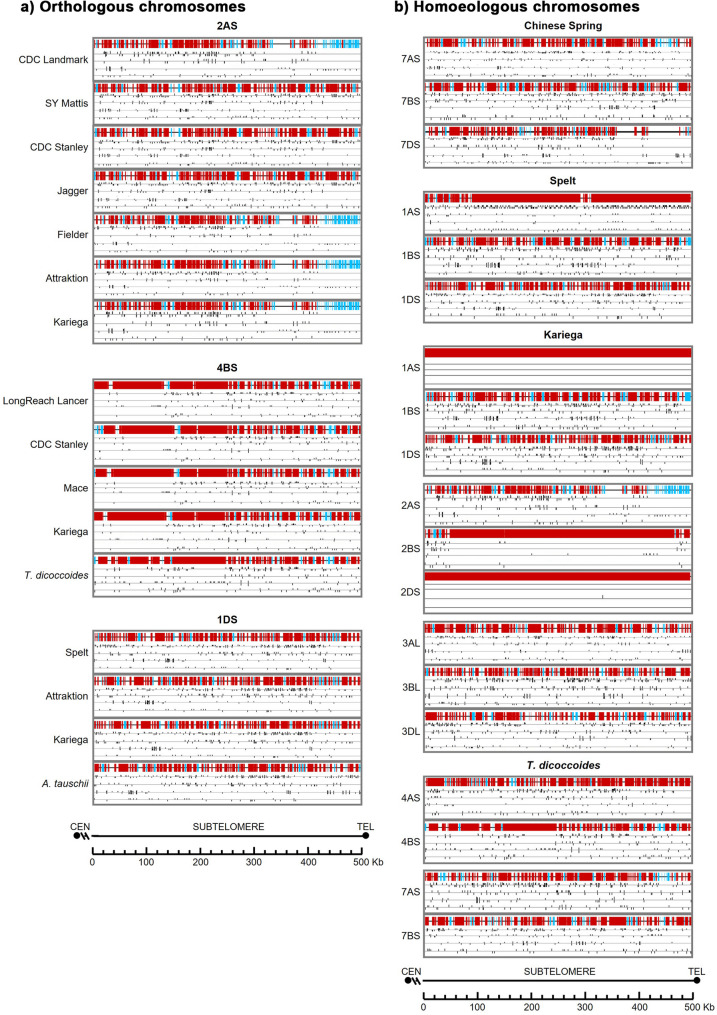
Predicted recombination hot spots and cold spots, and associated sequence motifs in the distal 500 kb of the subtelomeric region: (**a**) Orthologous chromosomes; (**b**) Homoeologous chromosomes. Red: Hot spots; blue: Cold spots. Additional panels show distributions of the motifs associated with hot spots (CCGCCGCCG, CTCCCTCC, TTAGTCCCGGTT), followed by predicted binding sites of wheat SMC1β homologs. Chromosome arms are oriented from the centromere toward the telomere

Among all chromosome arms, 1AS exhibits the highest density in recombination hot-spots, with a conserved distribution pattern among all the cultivars analysed. While the distribution and content of hot and cold spots are consistent among cultivars for the same chromosome, they differ significantly between homoeologous chromosomes (Fig. 11, Additional file 11). Hexaploid wheat cultivars share a similar recombination landscape, particularly among phylogenetically related lines (4AS; 7AS; 1BS). However, there are clear differences in the distribution patterns between the tetraploid *T. dicoccoides* (3AS, not shown) and the diploid *A. tauschii* (1DS, 5DL), when compared to hexaploid cultivars, although 6DL from *A. tauschii* resembles hexaploid profiles. Interestingly, the distribution pattern of *T. turgidum* (7BS) aligns more closely with hexaploid patterns.

A strong relationship was observed between the GC content, the distribution of CpG islands and the presence of recombination cold spots or absence of recombination hot spots, particularly for chromosomes 2AS, 3AS, and 7DS. A consistent correlation between -CCGCCGCCG- sequence motifs and hot recombination spots was also found in all chromosome arms in all species and cultivars. However, the -TTAGTCCCGGTT- motif seems to be more related to the presence of cold spots, such as in chromosome 2AS, where the other two motifs are absent. Interestingly, the motifs related to hot spots in chromosome 1AS from Kariega are absent.

### DNA-binding proteins

The distribution of predicted binding sites for the cohesin protein SMC1β was also analysed. The distribution of SMC1β binding motifs is chromosome-specific, showing clear variation in both density and distribution among chromosomes. However, for the same chromosome, patterns tend to be conserved among cultivars and species. For instance, hexaploid cultivars and *T. turgidum* share similar patterns on chromosome 7BS, while slight differences are observed in *T. dicoccoides* (4BS) and *A. tauschii* (1DS) (Fig. 11, Additional file 11). As occurred for most of the parameters studied before, the distribution patterns for the same chromosome among cultivars and species were more similar than those one among homoeologous chromosomes.

**Fig. 12:**
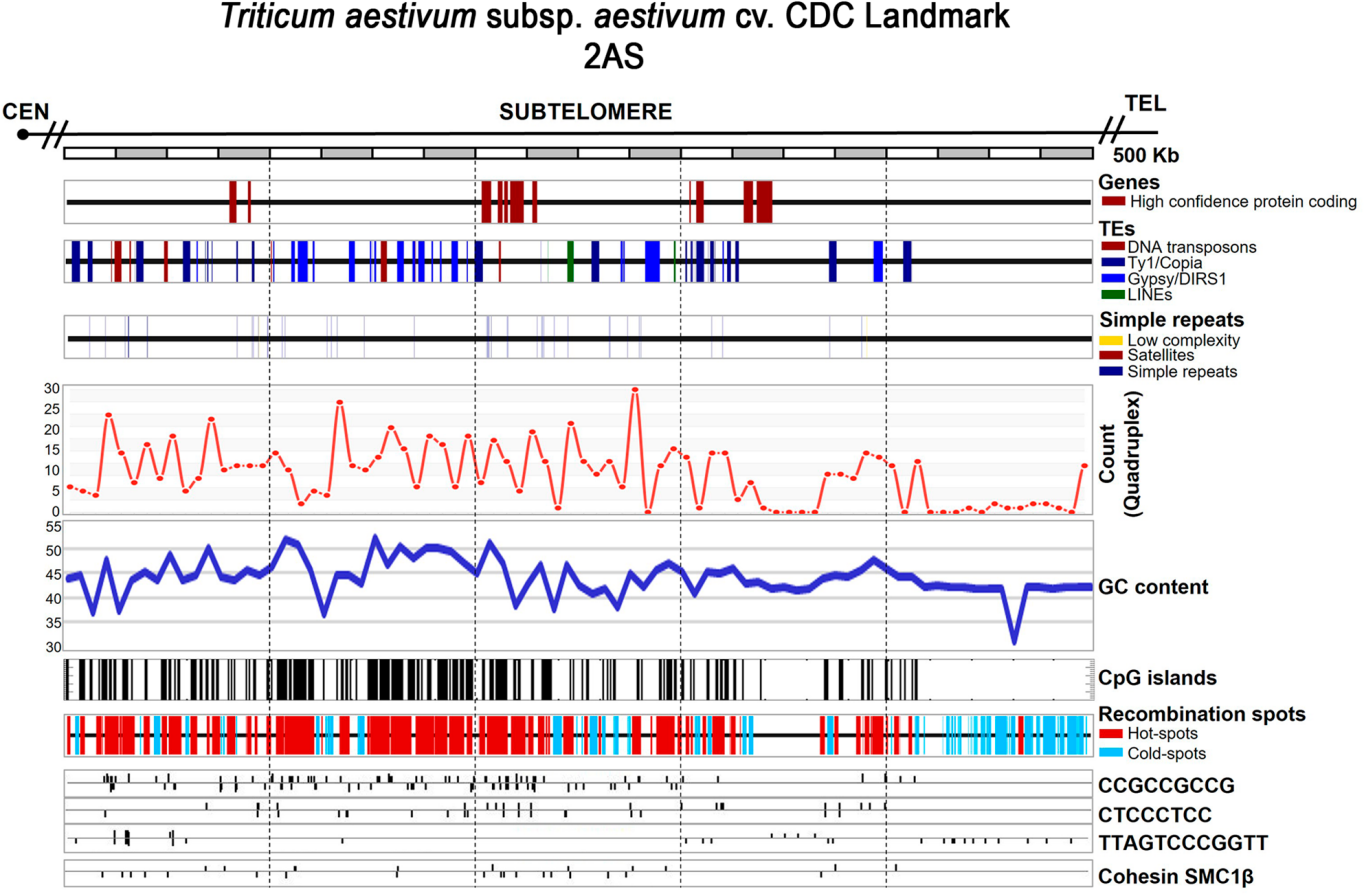
Integrated map of all the features analyzed in the distal 500 kb subtelomeric region adjacent to the telomere of chromosome arm 2AS in *Triticum aestivum* cv. CDC Landmark. Chromosome arm is oriented from the centromere toward the telomere

Finally, we assessed potential correlations among other genomic features. Strong correlations were observed between GC content, CpG islands, G4s, recombination spots, and recombination-associated sequence motifs, as illustrated in Fig. 12.

## Discussion

Plant chromosome terminal regions, including telomeres and subtelomeres, are vital for stability, recombination, and meiotic behaviour [63]. In polyploid wheat, precise homologous chromosome pairing during meiosis is crucial for genetic stability and diversity, even with potential homoeologous pairing [64]. This pairing is strictly controlled to favour homologues, ensuring correct chromosome segregation, preventing aneuploidy, and supporting recombination vital for plant fertility, adaptation, and breeding. In our study we deeply analysed telomeric and subtelomeric regions across various *Triticum* species and modern cultivars, uncovering a complex, dynamic “barcode” at chromosome ends that could contribute to the regulation of homologous recognition during meiosis.

A phylogenetic analysis clarified relationships among diverse wheat species and cultivars Although the phylogenetic analysis was based on a single gene (ZIP4-5B), it provides a useful approximation of the relative similarity among the analysed cultivars and species. Given that chromosomal rearrangements and gene-specific evolution can influence phylogenetic patterns, the results should be interpreted as illustrative rather than as a comprehensive reconstruction of evolutionary relationships. As anticipated, *A. tauschii* (the D genome donor) formed a distinct clade, reflecting its evolutionary distance [65]. Notably, *T. aestivum* ‘Renan’ clustered closer to *Aegilops* species, due to known disease resistance introgressions from *A. ventricosa* [52]. This result highlights the contribution of wild relatives to cultivated wheat’s genetic diversity, especially in terminal chromosome regions. For example, other *Aegilops*-derived segments carrying the Lr37-Yr17-Sr38 gene cluster were found in the terminal region of chromosome 2 A in cultivars like Jagger. Similarly, LongReach Lancer grouped with known introgressions from *T. timopheevii* and *T. ponticum* [48], and a 5B-7B chromosome translocation was previously observed between ArinaLrFor and SY Mattis [48]. These examples confirm that chromosome ends are frequent targets for artificial selection, recombination, and genomic rearrangements, impacting chromosome structure, recombination, and gene expression [66]. The independent clustering of PI190962 (Spelt wheat) aligns with its ancient divergence from modern bread wheat, retaining unique allelic and structural variants often near chromosome ends, making it a valuable genetic resource [48, 67]. Conversely, closely related cultivars, such as Chinese Spring and Norin-61, exhibited conserved gene distribution patterns, suggesting that phylogenetic proximity supports more consistent homologous chromosome pairing and recombination during meiosis.

Telomeric regions serve as protective chromosome caps and are critical for initiating pairing during the meiotic bouquet, where chromosome ends cluster to facilitate homolog alignment. In situ hybridization confirmed this motif across all metaphase chromosome ends in our work, consistent with its near-universal presence in land plants [68]. Assembling telomeric regions remains challenging, clearly revealed in our study showing highly variable reconstructed telomere lengths across wheat cultivars, due to their repetitive, GC-rich nature [69]. We found a strong correlation between telomere position and variant accumulation, with proximal telomeric regions (those closer to the subtelomere) having four times higher variant density than distal ends. This suggests recombination or replication stress at the subtelomeric interface, confirming that these adjacent regions are hot spots for instability and chromatin remodelling [70]. The higher variant bias, especially in subgenome B, implies that these processes preferentially introduce mutations near the subtelomeric interface. This localized polymorphism could create unique sequence signatures, contributing to homologous discrimination during meiosis.

We also observed consistent non-canonical telomeric variants of the plant-specific TTTACGG consensus repeat across independent wheat lines, ruling out technical errors and suggesting biological mechanisms like telomerase errors or replication slippage [34, 71]. While common mutations (T or G deletions) reflect telomerase errors, plants seem to tolerate some mutations while maintaining protective function. These mutations were not uniformly distributed. For example, subgenome D had the highest deletion frequency, including unique motifs like TTAAGGG, while subgenome B showed the most insertions and complex variants such as GTAGGG. This subgenome-specific bias aligns with previous findings on differing recombination and mutation rates in polyploid homoeologous genomes [72]. These variants, especially in subgenome B, could impact telomere-binding protein affinity, influencing chromatin structure and pairing fidelity [73]. The presence of these non-canonical variants confirms that plant telomeres are dynamic and diverge in a lineage-specific manner [68]. The prevalence of deletions in subgenome D mirrors observations in synthetic wheat, where D-genome telomeres show accelerated divergence after polyploidization [74]. ANOVA revealed that the genetic background accounts for the largest share of telomeric sequence variability. This suggests that breeding history, environmental stress, and introgression events significantly influence telomere evolution, aligning with findings that telomeric regions are integration points for alien chromatin and breeding targets in wheat [48, 75]. Furthermore, the higher sequence divergence of subgenome D, despite shorter canonical arrays, could stem from its more recent integration via hybridisation with *A. tauschii* and potentially less efficient telomere maintenance. This is consistent with reports indicating the *A. tauschii*-derived D genome experiences lower recombination and a higher mutational load [67]. Interestingly, while telomeric variant profiles were conserved among closely related wheat cultivars and between *T. aestivum* and *T. turgidum*, they were highly divergent among homoeologous chromosomes within the same genome. This suggests that specific variant profiles are inherited and preserved through domestication and speciation. However, the lack of conservation among homoeologous chromosomes within a cultivar indicates that telomere evolution is more strongly tied to chromosomal lineage than to species or cultivar identity [76].

While telomeres are often considered neutral, our results suggest that telomere sequence variants could impact function by modulating telomere-binding protein affinities, telomerase activity, or chromatin dynamics. In yeast and mammals, non-canonical repeats can reduce protein affinity, potentially destabilizing chromosome ends or altering telomerase function [73]. Furthermore, subtelomeric regions frequently contain gene-rich domains tied to disease resistance and environmental response [77], suggesting that telomere-proximal structural variants might influence gene expression via position effects or epigenetic mechanisms. Contrary to the traditional view of telomeres as highly conserved, our analysis clearly shows them to be extremely dynamic regions with significant sequence variation and structural complexity. This aligns with evidence that plant telomeres exhibit both sequence and structural polymorphisms influenced by genomic, chromosomal, and varietal factors [78]. Thus, telomeres may function as dynamic “identity elements”, like subtelomeric domains, and might also contribute to chromosome specificity at the onset of meiosis.

Telomeric variants are not randomly distributed. They cluster in specific zones interspersed with mutation-free gaps. These gaps vary significantly by subgenome and telomere position, being larger and more variable in subgenome B, and shorter and more uniform in subgenome D, aligning with its conserved telomeric architecture [74]. High mutation density regions showed fewer, shorter gaps, suggesting a spatial correlation between mutation accumulation and sequence conservation [79]. These mutation-free zones might be protected by protein-DNA interactions or chromatin compaction, acting as preferential targets for telomere-binding proteins. Multifactorial ANOVA revealed that telomere distance and genomic architecture (cultivar, chromosome, subgenome) significantly influence gap size and variant distribution. Telomere distance explained 28.4% of gap size variance and 23.7% of gap density, with subgenome and cultivar/species also having substantial effects. This confirms that both genetic background and chromosomal context shape telomere structure, consistent with telomeric evolution correlating with chromosomal architecture, replication timing, and local chromatin organization in other species [80]. The observed periodicity and clustering might indicate local chromatin structures or replication timing domains affecting mutations. The divergence of telomeric variant profiles among homoeologous chromosomes, despite conservation among related cultivars, suggests evolutionary selection against homoeologous pairing, a key mechanism for fertility in polyploids [81]. This “pairing affinity control” model can modulate homolog recognition thresholds, supported by *ZIP4-5B* and *Ph1* mutant analyses [19]. Our study’s correspondence between *ZIP4-5B* phylogenies and telomeric variant patterns reinforces that telomeric features contribute to meiotic pairing fidelity. Different patterns of mutation clusters and gaps among chromosomes and homoeologs could also impact homologous recognition and pairing, confirming that telomeres may function as dynamic “identity elements,” like subtelomeric domains, to modulate chromosome-specific recognition.

Overall, our results challenge the long-standing concept that telomeric regions are highly conserved. Instead, we propose a model in which plant telomeres are dynamic, structurally diverse domains shaped by mutation, recombination, and chromosomal context. This dynamic nature may be evolutionarily advantageous, facilitating adaptation through subtle genomic variability at chromosomal termini and contributing to chromosome recognition and pairing during early meiosis.

In addition, our analysis of G-quadruplexes (G4s) showed a highly dynamic and structured distribution in wheat telomeric regions, varying significantly along the telomere itself, across chromosomes, subgenomes, and cultivars. G4s, non-canonical nucleic acid structures, are known to influence genome stability, gene expression, and chromosome end maintenance [82, 83]. The G4 density that we found in our work (76.6 ± 23.6 G4s per kilobase) was consistent with previous studies [84]. While G4 density trends were similar across subgenomes A, B, and D, a 4-way ANOVA highlighted telomere distance as the strongest factor influencing G4 density, followed by cultivar/species, chromosome and subgenome. This suggests that structural characteristics like recombination frequency, chromatin accessibility, and epigenetic marks likely drive G4 formation and retention more than simple sequence divergence between subgenomes. Interestingly, we found a higher G4 density in distal telomeric regions, contrasting with the higher mutation density typically observed in proximal regions. This suggests that while proximal telomeres are mutation hot spots, G4-forming motifs might be selected against or disrupted by the higher mutation rates in these areas, indicating G4 structures are extremely sensitive to sequence integrity [85]. Conversely, distal telomeres may better preserve G4 structures due to lower mutation rates and selective pressure to maintain telomere function.

Comparing G4 patterns, we found conserved motifs across hexaploid wheat lines, especially between closely related cultivars, consistent with general telomere sequence conservation in *T. aestivum*. Interestingly, tetraploid wheat (*T. turgidum*) showed more G4 similarity to hexaploids than to its wild ancestor (*T. dicoccoides*), supporting genomic convergence during polyploidization and domestication [86]. The distinct G4 patterns in *T. dicoccoides* and *A. tauschii* might reflect species-specific telomere evolution or varying recombination dynamics in wild genomes [67]. Crucially, the lack of G4 conservation among homoeologous chromosomes indicates independent structural evolution of telomeres after polyploidization. This aligns with prior findings that telomeric and subtelomeric regions are highly prone to lineage and chromosome-specific structural rearrangements [77]. We observed a notable exception: a 743 nt-long, highly degenerate G-rich region at the distal telomere of chromosome 3BL in the Attraktion cultivar, which lacked both canonical telomeric repeats and G4 motifs. A similar internal region was found in Kariega 1BS. These anomalous regions may be degenerated telomeres, resulting from past recombination events and telomere sequence instability [73]. Given that subgenome B exhibits higher mutation rates and structural rearrangements [72], these findings are not surprising. Further sequencing in more cultivars is needed to determine the prevalence and functional implications of such degenerate telomeric regions.

Our results confirm that G4 distribution in wheat telomeres is non-uniform, shaped by complex interactions between telomere length, genomic background, and cultivar-specific features. These findings reinforced the view of the telomere as a dynamic genomic region, with G4 motifs playing distinct structural or regulatory roles depending on their position and context. Further experimental validation, including G4-binding assays and chromatin accessibility profiling, would help to clarify the biological implications of the observed G4 distribution patterns.

Regarding subtelomeres, these chromosome regions are key sites of genetic innovation, hosting rapidly evolving sequences, gene families, and transposable elements (TEs). In wheat, these regions also show structural differences between homoeologous chromosomes, potentially affecting meiotic pairing accuracy [77]. Our study mapped genomic features involved in homologous recognition, identifying elements that may influence pairing specificity in these distal regions. After analyzing subtelomeres, we found a unique G4 landscape crucial for pairing regulation. G4 density in subtelomeres was significantly lower than in telomeres but highly variable. Chromosome arm identity explained most variance, while subgenome origin had minimal impact, indicating that G4 formation depends on both local sequence composition and broader genomic context. Our findings align with studies in *A. thaliana* and *Oryza sativa*, where G4s are enriched in telomeres and certain gene regulatory regions but scattered in intergenic or repetitive subtelomeric areas [87]. The chromosome-specific variation in G4 density underscores the role of local genomic architecture, such as base composition and structural motifs, rather than lineage-wide effects. This matches barley data, where G4 density correlated more with chromosomal domain features than genome-wide trends [34]. Periodic G4 clustering was remarkably consistent across cultivars, suggesting an evolutionary constraint linked to chromatin organisation or nucleosome positioning [88]. G4s associate with recombination hot spots by inducing replication stress and double-strand breaks (DSBs), particularly in open chromatin and gene regulatory regions [82, 89]. While they can enhance genome instability and recruit recombination machinery to promote genetic diversity [90], G4s may also function as cold spots when their structure blocks helicases/recombination factors or occurs in heterochromatin [82]. This dual role depends on chromatin context and epigenetic regulation. In wheat, G4s may initiate homologous pairing by forming specific chromatin states, like in *Arabidopsis* [91], and influence transcription/replication timing [92]. However, their absence on arms like Kariega 1AS, 2BS, and Attraktion 6AL suggests recombination suppression or structural rearrangements may impair pairing efficiency. Conserved G4 patterns in hexaploid cultivars (e.g., Chinese Spring and Norin-61) indicate polyploidization-driven convergence in *T. turgidum* and *T. aestivum* [67]. The different D subgenome profile of *A. tauschii* suggests lineage-specific evolution, potentially altering pairing in wild vs. domesticated genomes. Subgenome origin (A/B/D) showed minimal impact on G4 abundance, confirming local structural factors and evolutionary rearrangements dominate G4 dynamics, which is consistent with the structural plasticity of the B subgenome, not correlating with G4 abundance [63, 65]. Our findings demonstrate that wheat subtelomeric G4 distribution is chromosome-specific rather than subgenome-dependent. The conserved periodic patterns across cultivars suggest functional roles in subtelomeric regulation, potentially affecting recombination, chromatin organization, and homologous pairing.

GC content and CpG islands critically influence wheat genome structure and function, affecting gene regulation, chromatin organization, and stability. Our data showed distinct patterns across chromosomes, cultivars, and subgenomes, reflecting chromosomal context, evolutionary history, and species-specific variation. GC/CpG content strongly correlates with gene density, recombination hot spots, and chromatin accessibility [93]. GC variation may stem from repetitive element accumulation, particularly in GC-poor regions. G4 motifs frequently co-localize with GC-rich genic regions and CpG islands [82], a pattern confirmed in wheat. The marginally higher GC content in subgenome A than in B and D, support evolutionary divergence [74]. CpG islands clustered in GC-rich regions (e.g., 1AS) and near telomeres, potentially promoting open chromatin for homologous pairing. A three-way ANOVA showed weaker effects of species/cultivar and subgenome, underscoring the dominance of local chromosomal features over lineage-wide trends. GC and CpG profiles differed significantly between *T. dicoccoides* (particularly 3AS) and hexaploids, highlighting polyploidization’s chromatin effects [94]. Among homoeologues (7AS/BS/DS), 7BS showed uniform CpG distribution while 7AS and 7DS had telomere-proximal depletion, suggesting differential evolutionary constraints on pairing. These epigenetic patterns may interact with the *Ph1* locus, known to regulate chromatin accessibility in polyploids [20]. G4-GC-CpG correlations were conserved in related cultivars (e.g., Lancer/Landmark 1AS) but divergent in homoeologues, potentially creating pairing-specific chromatin signatures. The extensive GC/CpG variation across chromosomes, cultivars and subgenomes reflects wheat’s complex genome architecture. These features likely influence gene regulation, chromatin organization, and meiotic pairing dynamics.

Gene density in subtelomeres showed significant variation, consistent with barley’s variable distal gene content and rearrangement susceptibility [34]. Chromosome identity explained most of the variance, with 1AS consistently gene-rich, matching its known role as a recombination anchor [95]. Conserved gene-rich zones in related cultivars (e.g., Chinese Spring/Norin-61 4AS; Mace/Julius 7BS) reflect shared ancestry and selection pressures maintaining synapsis-critical regions [48]. Conversely, homoeologues showed divergence through subfunctionalization/silencing [86]. Low-density regions (Kariega 1AS/2DS; *T. dicoccoides* 4AS) suggest gene deserts/TE expansions that may impair pairing. Gene-recombination hotspot correlations indicate accessible chromatin facilitates homolog alignment, as seen in maize/*Arabidopsis* [96, 97]. Subtelomeric genes often control agronomic traits and may influence pairing via position/epigenetic effects [98].

Transposable elements (TEs), particularly retrotransposons, significantly influence genome structure, chromosome behaviour, and subgenome evolution [99]. In wheat, subtelomeric TEs may affect meiotic pairing through chromatin modulation, with subgenome B showing highest density via Gypsy and Copia elements. These elements can alter chromatin architecture [100] and promote rearrangements impacting pairing. The lower TE content in subgenome D and its compact structure enhance homolog recognition, while B’s plasticity facilitates adaptive introgressions like the Lr37-Yr17-Sr38 cluster [75]. Conserved TE patterns in related cultivars (e.g., Chinese Spring/Norin-61 4AS) contrast with divergence among homoeologous chromosomes, suggesting TE-mediated pairing regulation through chromatin changes, as in maize [101]. DNA transposons showed minimal subgenome bias. TE enrichment near recombination cold spots may prevent improper homoeologous pairing [72]. Cultivars like Kariega and SY Mattis with higher retrotransposon loads may exhibit distinct pairing behaviour. Chromosomal TE variation contributes to wheat’s evolutionary adaptability, with TE-rich regions predisposed to structural changes affecting pairing stability and genome remodelling potential.

Wheat subtelomeres show significant variation in satellite repeats, SSRs, and low-complexity DNA across chromosomes, subgenomes, and cultivars. Satellite repeats dominate subgenome A and reach extreme densities in some chromosomes (99.0% in Kariega 1AS), potentially causing localized heterochromatinization linked to meiotic suppression [102]. Diploid relatives like *A. tauschii* showed divergent profiles, reflecting post-polyploidization divergence [65]. Chromosome identity and subgenome primarily drive repeat distribution, with minor cultivar effects, indicating structural constraints. SSRs are more uniform but slightly enriched in subgenome B, likely contributing to chromatin stability [103]. Low-complexity DNA maintains consistent density across subgenomes, suggesting conserved structural roles [104]. Satellite-rich regions in subgenome B may either facilitate (homologous landmarks) or hinder (chromatin compaction) pairing, depending on interactions with G4s/TEs. These findings highlighted how subgenome lineage, chromosome identity, and repeat type collectively shape subtelomeric structure and potentially influence pairing dynamics.

Our study reveals striking variability in hotspot distribution across chromosomes and cultivars. While 1AS consistently showed hotspot richness, tetraploid (*T. dicoccoides* 3AS) and diploid (*A. tauschii* 1DS) divergence highlights polyploidization’s impact [13]. Cold spots, enriched in TEs and lacking recombination motifs (e.g., TTAGTCCCGGTT in 2AS), prevail in regions like Kariega 2DS, suppressing crossovers to maintain subgenome specificity. Hotspot patterns are conserved across cultivars per chromosome arm but differ between homoeologues, reflecting distinct evolutionary histories. This aligns with findings that recombination is shaped by chromosomal structure, epigenetics, and evolution [105]. Hot spots strongly correlate with GC/CpG islands, known for transcriptional and meiotic activity [106], making them predictive markers. G4s show context-dependent effects: moderate density near hot spots may promote recombination, while high density (common near gene promoters/telomeres) can inhibit it by stabilizing chromatin [89], balancing genome accessibility and stability. Key findings reveal substantial recombination variability influenced by chromosomal, cultivar, and species-specific factors, with polyploidization playing a key role in shaping wheat’s recombination landscape. Strong correlations between recombination, GC/CpG content, and open chromatin suggest a coordinated genomic architecture facilitating homologous pairing and crossover formation. These results advance our understanding of wheat recombination regulation and inform breeding strategies for enhancing genetic diversity. Hot spots associate with GC-rich regions, G4s, genes, and open chromatin, collectively promoting synapsis through accessible pairing sites, a mechanism observed in *Arabidopsis* [107]. The *Ph1* locus, crucial for recombination fidelity, likely uses these epigenetic/sequence cues, as its loss leads to aberrant homoeologous pairing [20].

Finally, related to proteins interacting with DNA, SMC1β, is a key cohesin subunit that shows chromosome-specific binding patterns in wheat. While conserved on arms like 4AS and 7BS, it diverged in *T. dicoccoides* 4BS and *A. tauschii* 1DS. These SMC1β-rich regions, typically gene-dense and GC-rich, stabilize homologous pairing during synapsis [108]. Conserved SMC1β sites maintain chromatin signatures within cultivars, while homoeologous divergence prevents mispairing. Cohesins likely enhance pairing fidelity by interacting with G4s and recombination hot spots to form chromatin loops [109]. Minor cultivar-specific cohesion differences may reflect breeding history and influence meiosis outcomes.

Collectively, our findings reveal that wheat telomeres and subtelomeres function as dynamic genomic hubs where sequence diversity, structural motifs (e.g., G4s, TEs), and epigenetic states converge to regulate homologous pairing. Subtelomeric “barcodes”, marked by G4 periodicity, TE patterns, and CpG islands, encode chromosomal identity, enabling precise homolog discrimination in polyploids while suppressing non-homologous interactions. This aligns with the telomere-led bouquet configuration, which facilitates efficient homology searches despite homoeologous sequence similarity. The meiotic genome organizes into a loop-axis architecture: chromatin loops (GC-rich, G4-dense, and enriched in active genes/TEs) promote recombination diversity, while the cohesin-anchored axis (AT-rich, TE-dense, and G4-sparse) ensures stability. This balance is critical, open loops allow SPO11-mediated DSBs and RAD51/DMC1 repair, whereas the axis restricts aberrant recombination. While subgenome ancestry provides an evolutionary framework, local features (TE activity, G4 density, recombination motifs) dictate pairing specificity. Our model redefines telomeres as active regulators of meiotic orchestration, explaining both high-fidelity homolog pairing and rare homoeologous mismatches across wheat cultivars. These insights advance understanding of polyploid genome stability and offer new avenues for breeding strategies aimed at manipulating recombination landscapes.

## Supplementary Information

Supplementary Material 1

Supplementary Material 2

Supplementary Material 3

Supplementary Material 4

Supplementary Material 5

Supplementary Material 6

Supplementary Material 7

Supplementary Material 8

Supplementary Material 9

Supplementary Material 10

Supplementary Material 11

## Data Availability

All raw data underlying this article are available in the article.
