# Supplementary material for: Role of chromosome ends in meiotic stability, recombination and wheat evolution in the context of breeding
Source: BMC Plant Biol. 2025 Dec 29;26:187. doi: 10.1186/s12870-025-08020-5 (PMC12859859; doi:10.1186/s12870-025-08020-5)
Supplement: Supplementary file 1 — Supplementary Material 1 [file 12870_2025_8020_MOESM1_ESM.docx]

**[Supporting Information Figure S1](https://www.dropbox.com/home/An%C3%A1lisis%20Tel%C3%B3meros%20y%20Subtel%C3%B3meros%20Trigos/ARTICULO%20para%20enviar/TABLAS%20Y%20FIGURAS%20DEFINITIVAS/FIGURAS).** Chromosome end sequences of the different species and cultivars analyzed in this study. All the sequences are oriented from centromere to telomere end. The beginning of the telomere is identified by the first complete telomeric sequence marked in bold. Sequences were obtained from the NCBI webpage: <https://www.ncbi.nlm.nih.gov/datasets/genome/>.

***T. aestivum* cv. Renan_v2.1**

**7AS**

AGGG**TTTAGGG**TTTAGGGTTTAGGGTTTTAGGGTTTAGGGTTTAGGGTTTAGGGTTTAGGGTTTAGGGTTTAGGGTTTAGGGTTTTAGGGTTTAGGGTTTAGGGTTTAGGGTTTAGGGTTTAGGGTTTAGGGTTTAGGGTTTAGGGTTTAGGGTTTAGGGTTTAGGGTTAGGGTTTAGGGTTTAGGGTTTAGGGTTTAGGGTTTAGGTTTAGGGTTTAGGGTTTAGGGTTTAGGGTTTAGGGTTTAGGGTTTAGGGTTTAGGGTTTAGGGTTTAGGGTTTAGGGTTTAGGGTTTAGGGTTTAGGGTTTAGGGTTTAGGGTTTAGGGTTTAGGGTTTAGGGTTTAGGGTTTAGGGTTTAGGGTTTAGGGTTTAGGGTTTAGGGTTTAGGGTTTAGGGTTTTAGGGTTTAGGGTTTAGGGTTTAGGGTTTAGGGTTTAGGGTTTAGGGTTTAGGGTTTAGGGTTTAGGGTTTAGGGTTTAGGGTTTAGGGTTTAGGGTTTAGGGTTTAGGGTTTAGGGTTTAGGGTTTAGGGTTTAGGGTTTAGGGTTTAGGGTTTAGGGTTTAGGGTTTAGGGTTTAGGGGGTTTAGGGTTTAGGGTTTAGGGTTTAGGGTTTAGGGTTTAGGGTTTAGGGTTTAGGGTTTAGGGTTTAGGGTTTAGGGTTTAGGGTTTAGGGTTTAGGGTTTAGGGTTTAGGGTTTAGGGTTTAGGGTTTAGGGTTTAGGGTTTAGGGTTTAGGGTTTAGGGTTTAGGGTTTAGGGTTTAGGGTTTTAGGGTTTAGGGTTTAGGGTTTAGGGTTTAGGGTTTAGGGTTTAGGGTTTAGGGTTTAGGGTTTAGGGTTTAGGGTTTAGGGTTTAGGGTTTAGGGTTTAGGGTTTAGGGTTTTAGGGTTTAGGGTTTAGGGTTTAGGGTTTAGGGTTTAGGGTTTAGGGTTTTAGGGTTTAGGGTTTAGGGTTTAGGGTTTAGGGTTTAGGGTTTAGGGTTTAGGGTTTAGGGTTTAGGGTTTAGGGTTTAGGGTTTAGGGTTTAGGGTTTAGGGTTTAGGGTTTAGGGTTTAGGGTTTAGGGTTTAGGGTTTAGGGTTTAGGGTTTAGGGTTTAGGGTTTAGGGTTTAGGGTTTAGGGTTTAGGGTTTAGGGTTTAGGGTTTAGGGTTTAGGGTTTAGGGTTTAGGGTTTAGGGTTTAGGGTTTAGGGTTTAGGGTTTAGGGTTTAGGGTTTAGGGTTTAGGGTTTAGGGTTTAGGGTTTAGGGTTTAGGGTTTAGGGTTTAGGGTTTAGGGGTTTAGGGTTTAGGGTTTAGGGTTTAGGGTTTAGGGTTTAGGGTTTAGGGTTTAGGGTTTAGGGTTTAGGGTTTAGGGTTTAGGGTTTAGGGTTTAGGGTTTAGGGTTTAGGGTTTAGGGTTTAGGGTTTAGGGTTTAGGGTTTAGGGTTTAGGGTTTAGGGTTTAGGGTTTAGGGTTTAGGGTTTAGGGTTTAGGGTTTAGGGTTTAGGGTTTAGGGTTTAGGGTTTAGGGTTTAGGGTTTAGGGTTTTAGGGTTTAGGGTTTAGGGTTTAGGGTTTAGGGTTTAGGGTTTAGGGTTTAGGGTTTAGGGTTTAGGGTTTAGGGTTTAGGGTTTAGGGTTTAGGGTTTAGGGTTTAGGGTTTAGGGTTTAGGGTTTAGGGTTTAGGGTTTAGGGTTTAGGGTTTAGGGTTTAGGGTTTAGGGTTTAGGGTTTAGGGTTTAGGGTTTAGGGTTTAGGGTTTAGGGTTTAGGGTTTAGGGTTTAGGGTTTAGGGTTTAGGGTTTAGGGTTTAGGGTTTAGGGTTTAGGGTTTAGGGTTTAGGGTTTAGGGTTTAGGTTTTAGGGTTTAGGTTTAGGGTTTAGGGTTTAGGGTTTAGGGTTTAGGGTTTAGGGTTTAGGGTTTAGGGTTTAGGGTTTAGGGTTTAGGGTTTAGGGTTTAGGGTTTAGGGTTTAGGGTTTAGGGTTTAGGGTTTAGGGTTTAGGGTTTAGGGTTTAGGGTTTAGGGTTTAGGGTTTAGGGTTTAGGGTTTAGGGTTTAGGGTTTAGGGTTTAGGGTTTAGGGTTTAGGGTTTAGGGTTTAGGGTTTAGGGTTTAGGGTTTAGGGTTTAGGGTTTAGGGTTTAGGGTT

**7AL**

TTTGACTG**TTTAGGG**TTGGGTTTAGGTTTAGGGTTTAGGGTTTAGGGTTTAGGGTTTAGGGTTTAGGGTTTAGGGGGTTTAGGGTTTAGGGTTTAGGGTTTAGGGTTTAGGGTTTAGGGGGTTTAGGGTTTAGGGTTTAGGGTTTAGGGTTTAGGGTTTAGGGTTTAGGGTTTAGGGTTTAGGGTTTAGGGTTTAGGGTTTAGGGGTTTAGGGTTTAGGGTTTAGGGTTTAGGGTTTAGGGTTTAGGGTTTAGGGTTTAGGTTTTGGGTTTAGGGTTTAGGGTTTAGGGTTTAGGGTTTAGGGTTTAGGGTGGTTTAGGGTTTAGGGTTTAGGGTTTAGGGTTTAGGGTTTAGGGTTTAGGTTAGGGTTTAGGGTTTAGGGTTTAGGGTTTAGGGTTTAGGGTTTAGGGTTTAGGGTTTAGGGTTTAGGGTTTAGGGTTTAGGGTTTAGGGTTTAGGGTTTAGGGTTTGGGTTTAGGGTTTAGGGTTTAGGGTTTAGGGTTTAGGGTTTAGGGTTTAGGGTTTAGGGTTTAGGGTTTATTGGGTTTAGGGTTTAGGGTTTAGGGTTTAGGGTTTAGGGTTTAGGGTTTAGGGTTTAGGGTTT

***T. aestivum* cv. Spelt**

**1AS**

TTTAGGTAGGTTTAGGTTAGGG**TTTAGGG**TTTAGGGGTTTAGGGTTTAGGGTTTTGGTTAGGGTTTAGGGTTAGGGGTTTAGGGTTTAGGGTTTAGGGTTTAGGGTTTAGGTTTAGGGTTTAGGGTTTT

**1AL**

GGG**TTTAGGG**TTAGGGTTTTAGGGTTTAGGGTTTAGGGTTTAGGGTTTAGGGTTAGGGTTTAGGGTTTAGGGTTTAGGGTTTAGG

**3AS**

T**TTTAGGG**TTTAGGGTTTAGGGTTTAGGGTTTAGGGTTAGGGTTTAGGGTTAGGGTTTAGGGTTTAGGGTTTAGGGTTTAGGG

**4AS**

AGGG**TTTAGGG**TTTAGGGTTTAGGGTTTAGGGTTTAGGGTTTAGGGTTAGGGTTTAGGGTTTAGGGTTAGGGTTTAGGGTT

**6AL**

TAGGG**TTTAGGG**TTTAGGGTTTAGGGTTTAGGGTTAGGGTTTAGGGTTTAGGGTTTTAGGGTTTAGGGTTTGGGGTTTTTAGGGTTTAGGGTTTAGGGTTTAGGGTTTAGGGTTAGGGTTTAGGGTTTAGGGTTTAG

**7AS**

TTAGGGTTT**TTTAGGG**TTTAGGGTTTAGGGTTTAGGGTTAGGGTTTAGGGTTAGGGTTTAGGGTTTAGGGTTTAGGG

**1BS**

AGGG**TTTAGGG**TTTAGGGTTTAAGGGTTTAGGGTTAGGGTTAGGGTTAGGGTTTAGGGTTTAGGGTTTAGGGTTTAGGGTTTAGGGTTTAGGGT

**3BS**

AGGG**TTTAGGG**TTTAGGGTTTAGGGTTTAGGGTTTAGGGTTTAGGGTTTAGG

**7BS**

AGG**TTTAGGG**TTTAGGTTTTAGGGTTAGGGTTTAGGGTTTAGGGTTTAGGGTTAGGGTTAGGGTTAGGGTTTAGGGTTTAGGGTTAGGGTTTAGGGTTTAGGGTTTAGGGTTTAGGGTTTA

**1DS**

AAGGG**TTTAGGG**TTTAGGGTTAGGGTTTAGGGTTAGGGTTTAGGGTTTAGGGTTTAGGGTTTAGGGTTTAGGGTTAGGGTTTAGGGT

**4DL**

GGAGTGA**TTTAGGG**TTTAGGGTTTAGGGTTAGGGTTTAGGGTTTAGGGTTAGGGTTTAGGGTTTAGGGTTTAGGGTTAGGGTTTAGGGTTTAGGG

***T. aestivum* cv. Alchemy**

**7AL**

TACCTTAGGG**TTTAGGG**TTAGGGTTTAGGGTTTAGGGTTTAGG

**7BS**

GGAATAGG**TTTAGGG**TTTAGGGTTTAGGGTTTTTAGGCTTTAGGGTTTAGGGTTTAGGGTTTAGGGTTTTTAGGGTTTAGGGTTTAGGGTTTAGGGTTTACGGTTTAGGGTTTAGGGTTTAGGGTTAGTTAGGGTTAGGGTTTTTAGGGTTTAGGGTTTAGGGTTTAGGGTTTAGGGTTTAGGGTT

**1DL**

TTTTAGGTTTTAGGTTTGGGG**TTTAGGG**TTTAGGGTTTAGGGTTTAGGGTTTAGGGTTTAGGGTTTAGGGTTTAGGGTTTAGGGTTTAGGGTTTAGGGTTTAGGGTTTAGG

***T. aestivum* cv. Aikang58**

**6AL**

TAGGG**TTTAGGG**TTTAGGGTTTAGGGTTTAGGGTTTAGGGTTTAGGGTTAGGGTTTAGGGTTTAGGGTTTACCCCGCGCCCTTAGGGTTTAGGGTTTAGGGTTTAGGGGTTAGGGTTTAGGGTTGTACCGGGTTTAGAGGTTTAGGTTTCTGAGCGGTTAGGTTAGGCAGTTTTTAGGTTAGGGTGTTAGGGTTTAGGAAGTTTAGGTTAGGGGTTTAGGGAATTTAGAGTTTATTTGTTGGTTAGGGTTTAGGGTTAGGGTTTAGGGTTAGGGTTAAGGTTTAGGTTTAGGGTTTAGGGTTTAGGGTTTTAGGGTTTAGGGTTTAGGGTTTAGGGTTGGGTTTAGGTAGGTTATGGGTTAGTTAGGGT

**7AS**

TTAAGGGTTAAGGG**TTTAGGG**TTTATGGTTTAGGGTTTAGGGTTTAGGGTTTAGGGTTTAGGTTTAGGTTTAGGGTTTAGGGTTTAGGGTTTAGGGTTTAGGGTTTAGGGTTTAGGTTTTAGGGTTTAGGGTTTAGGTTTTAGGGTTTAGGGTTTAGGGTGTTAGGGTTTAGGTAGGTTTTGTTTAGGGTTTAGGTTTAGGTTTTAGGGTTTAGTTAGGGTTTAGGGTTTAGGGTTTAGGGTTAGGGTTTGGGTTTAGGGTTAGGGTTAGGTTAGGGTTTAGGTTTTTAGGGTTTAGGGTTTAGGGTTAGGGTTTAGGGTTTAGGGTTTAAGGTAGGGTTTAGGGTTAGGGTTTTTAGGGTTTAGGGTTTTAGGGTTTAGGGTTTAGTGGTTTGGGTTTAGGGTTTAGGTTTTTAGGGTTAGGGTTTAGGGTTTAGGGTTAGGGTTAGGGTTTAGGGTTTGGGTTAGGGTTTAGGGTTTAGGGTTAGGGTTTAGGGTTAGGGTTTAGGGTTGGTTTAGGGTTTAGGGTTAGGGTTTAGGGTTTAGGGTTTAGGGTTTTAGGGTTAGGGTTAGGTTTAGGGTTTAGGGTTGTGTAGGGTTTAGGGTTTAGGGTTTAGGGTTTAGGGTTTAGGGTTTAGGGTTTGGGTTAGGGTTTAGGGTTTAGGGTTTAGGGTTAGGTAGGGGTTTGGGTTAGGGTTTTTTAGGGTTATTGAGGGTTTAGGGTTTTAGGGTTTAGGGTTAGGGTTTAGGGTTTAGGGTTTAGGGTTTAGGGTTTAGGGTTTAGGGGTTTTTAGGGTTTAGGGTTGGGTTTAGGGTTTAGGGTTTAGGGTTTAGGGTTTAGGGTTTAGGGTTTAGGTTTAGGGTTTAGGGTTTTAGGTTTTTAGGGTTTAGGGTTTAGGGTTTTTAGGGTTTAGGGTTTTAGGGTTTAGGGTTTTAGGGTTAGGGTTTAGGGTTTAGGGTAGGGTTTAGGGTTTTAGGGTTTAGGGTTAGGGTTTTAGGGTTTAGGGTTTAGGGTTAGGTTTAGGGTTAGGGTTAGGGTTTAGGGTTTAGGGTTTTAGGGGTTTAGGGTTTAGGGTTTAGGGTTTAGGGTTTAGGGTTTAGGGTTTAGGGTTTAGGGTTTTTAGGGTTTAGGGTTTAGGGTTTAGGGTTTAGGGTTTTAAGGGTTTAGGGTTTAGGGTTAGGGTTTAGGGTTTAGGGTTTAGGGTTTAGGGTTTAGGGTTTAGGGTTTAGGGTTTAGGGGTTTAGGGTTTAGGTAGGGTTTAGGGTTTAGGGTTAGGGTTTAGGGTTTAGGGTTTAGGGTTTAGGGTTTTTTTAGGGTTTAGGTTTAGGTTTTAGGTAGGGTTTAGGGTTAGGGATTTAGGGTTAGGGTTAGGGTTAGGGTTTAGGGTTTTAGGGTTTAGGGTTTAGGGTTTAGGGTTTAGGGGTTTTTTAGGGTTTTAGGGTTTTTAGGGTTTTAGGGTTTAGGGTTTAGGGTTAGGGTTTTTTTAGGGTTTAGGGTTTAGGGTTTTAGGGTTTTAGGGTTTAGGGGTTTAGGGTTTAGGGTTTAGGGTTAAGGGTAGGGTTTAGGGTTTAGGGTTTAGGGTTTAGGGTTTTTAGGGTTTAGGGTTAGGGTTAGGGTTAGGGTTTAGGGTTTAGGGTTTAGGGTTAGGGTTTAGGGTTTAGGGTTTAAGGGTTTAGGGTTTAGGGTTAGGGTTTTAGGGTTTTAGGGTTTAGGGTTTGGGTTAGGTTTAGGGTTTAGGGTTTGGTTAGGTTAGGGTTTAGGGTTTAGGGTTTAGGGTTTTGGGTTTAGGGTTAGGGTTTAGGGTTAGGGTTAGGTTTAGGGTTTAGGGTTTAGGGTTAGGGTTAGGGTTTAGGGTTTAGGGTTAGGGTTTAGGGTTTAGGGTTTTAGGGTTTAGGGTTTAGGGTTAGGGTTAGGTTAGGGGTTAGGGTTAGGGTTTTAGGGTTTAGGGTTAGGGTTTAGGGTGGTTTGGGGTTTAGGTTTAGGGTTTAGGGTTTAGGGGTTTAGGGTTTATGGGTTTAGGGTTTAGGGTTTTTAGGGTTAGGGTTTAGGGTTTAGGTTTAGGGTTTAGGGTTAAGGTTTAGGGTTTAGGGTTAGGGTTAGGGTTAGGGTTTAGGGTTTAGGGTTAGGGTTAGGGTTTAGGGTTTTTAGGGTTTAGGGTTTAGGGGTTTTAAGGGTTTTAGGGTTTAGGGTTATAGGGTTTGGGTTAGGGTTTAGGGTATTAGGGTTTAGGTTAGGGTTTAGGGTTTAGGGTTAGGGTTTTAGGTTGGTTTAAGGTTTAGGTGGTTTAAGGGTTTAGGGTTTAGGTTTAGGGTTTTAGGGTTTAGGTTAGGGTTTAGGGTTAGGGTTAGGGTTTAGGTTTAGGGTTTAGGGTTTAGGGTTTAGGGTTTAGGGTTTAGGGTTTAGGGTTTAGGGTTTAGGGTTTAGGGTTTACGTTAGGTTAAGGTTTAGGGTTAGGGTTTAGGGTTTAGGGTTTAGGGTTTAGGGGGTTTAGGGTTTAGGGGTATTAGGGTTTAGGGTTTAGGGTTAGGGTTAGGGTTTTAGGGTTTTAGGGTTTAGGGTTTGGGTTTAGGGTTTAAGGGTTTAGGGTTTTAGGGTTTAGGGGTTTAGGGTTTTAGGGTTTAGGGTTTAGGGTTTTTAGAAGGGTTTAGGTTTTAGGGTTTAGGGTTTAGGGTTTAGGGTTTTTAGGGTTTAGGGTTTAAGGGTTTTTAGGGTTTAGGGTTTAGGGTTTAGGGTTTAGGTTTAGGGTTTAGGGGGTTAGGGTTTAGGGTTTAGGGTTAGGGTTAGGGTTTAGGGTTTAGGGTTTAGGGTTTAGGGTTTAGGGTTTAGGGTTTAGGGTTTAGGGTAGGGGTTAGGGTTAGGGGTTTAGGGTTAGGGTTAAGGGTTTGGGTTAGGGTTTAGGGTTTAGGGTTTAGGGGTTATAGGTTTAGGGTTAGGGTTAGGGTTTAGGGGTTTAGGGTTTAGGGTTTAGGGTTTAGGGTTTAGGGTTAGGGTTTAGGGTTTAGGGTTTAAGGGTTTTTAGGGTTTAGGGTTTAGGTTTAGGGTTTTAGGGTTTAGGGTTTAGGGTTAGGGTTTAGGGTTTAGGGTTTAGGGTTTAGGGTTTAGGGTTAGTTTAGGGTTTAGGGTAGGGTTTAGGGTTAGGTTAGGGTTTAGGTTTAGCTTTAGGGTTTAGGGTTTAGGGTTTTAAGGGTTTAGGGTTTTAGGGTTAGGGTTTAGGGTTAGTTAGGGTTTAGGGTTTAGTGTGGGTGTAGGAGTAGGTAAGGTAAGGTGTAA

**7AL**

GGG**TTTAGGG**TTTAGGGTTTAGGGTTTAGGGTTAGGGTTTTGGTTTAGGATTAGGGTTAGGGTTTAGGTTTTAGGGTTTAGGGTTAGGGTTAGGTTTAGGGTTTAGGGTTTAGGGTTTAGGGTTTAGGGTTTAGGGTT

**4DL**

GGAGTGA**TTTAGGG**TTTAGGGTTTAGGGTTAGGGTTTAGGTTTAGGGTTAGGGTTTAGGGTTTAGGGTTTAGGGTTAAGGGGTTTAGGGTTAGGGTTTAGGGTTTAGGGTTTAGGTTAGGGTTAGGGTTTAGGGTTAGGGTTTTAAGGGTTTAGGTTTTAGGGTTTAGGGTTTTAGGGTTTAGGGTTAGGGGTTTAGGGTTTAGGGTGTTAGGGTTTAGGGTTAGGTTAGGGTTTTAGGGTTTAGGGTTTAGGGTTTAGGGTTAGGGTTTAGGGTTTAGGGTTTAGGGTTTAGGGTTTAGGGTTTAGGTTAGGGTTTAGGGTTTAGGGGTTTAGGGTTTAGGGTTTAAGGGTTTAGGGTTTAGGGTTTAGGGTTTAGGGTTTAGGGTTTAGGGTTTTAGGGTTAGGGTTTAGGGTTTTAGGGTTTAGGGTTTAGGGTTTAAGGGTTTAGGGTTTAGGGTAGGGTTAGGGTTTAGGGTTTAGGGTTTAGGGTTTAGGGTTTTAGGGTTTTAGGTTTAGGGTTTAGGGTTTAGGGTTTAGGGTTAAGGGTTAGGGTTTAGGGTTTTAGGGTTTAGGGTTAGGGTTTAGGTTTAGGGTTTAGGGTTAGGGTTTTAGGGTTTTAGGTTTTAGGGTTTTAAGGGTTTTATGGGGTTTAGGTTTTAGGGTTTAGGTAGGTTTAGGGTTTAGGGTTTAGGGTTTTAGGGTTTGGGTTTAGGGTTTAGGGTTTAGGGTTTAGGGTTTAGGGTTTAGGTTAGGGTTAGGGTTTTTAGGGTTTAGGGTTTAGGGTTTAGGTTTAGGGTTTTTAGGGTTAGGGTTTTAGGGTTAGGGTTTAAGGTTTAGGGTTAGGGTTTAGGGTTTAGGGTTTAGGGTTTTTGGGTTAGGAGTTTAGGGTTTTAGGTTTTAGGTTAGGGTTTAGGGTAGTTTTAGGGTTTAGGGGTTTAGGGTTTAAAGGGTTTAGGGTTTAGGGTTTAGGGTTTAGGTTAGGGTTTAGGGTTAGGGTTTTAGGGTTTAGGGTTAGGGTTTAGGGTTTAGGTTTAAGGGTTTAGGTTTAGGGTTAGGGTTTAGGTTTAAGGGTTTAGTGGGTTTAGGTTTAGGGTTTAGGTTTTAGGGTTTAGGGTTAAGGGTTTAGTTTAGGGTTTAGGTTTTAGTTTAGGGGTAGGGTTTAGGGGTTAGGGTTTTAGGGTTTAGGGTTTAGGGTTTAGTTAGGGGTTTAGGGTTAGGGTTAGGGTTTAGGGTTTAGGGTTTGGGTTAGGGTTAGGGTTAAGGGGTTTAGGGTTTAGGGTTTAGTTTAGGGTTTTTTAGGGTTAGGGTTTTAGGGTTTAGGGTTTAGGGTTTAGGTTAGGGTTTAGGGTTTGGGTTTAGGGTTAAGGGTTGGTTATAGTTTAGGGTTAGGTTTAGGGTTAGGGTTTAGGGTTTAGGGTTTAGGGTTTAGGGGTTTAGGGTTTTAGGGTTTAGGGTTTAGGGTTAGGGTTTAGGGTTTGGGGTTTAGGGTTTAGGGTTTAGGGTTTTGGGTTTAGGGTTAGGGTTTAGGGTTTAAGGGTTTAGGGTTTAGGGTTAGGGTTTAGGGTTTTAGGGTTTTTTTTTTTAGGGTTTAGGTTTTAGGGTTTAGGTTAGGGGTTTAGGGTTAGGGTTTAGGGTTTAGGGTTAGGGTTTAGGGTTAGGGTTAGGGTTTAGGGTTTAGGGTTTAGGGTTAGGGGTAGGGTTTAGGGTTTAGGGTTTAGGGTTTAGGGTTTAGGTTTAGGGTTAGGGTTTTTAGGGTTTAGGGTTTAGGGTTTTAGGTTTAGGTTTAAGTGTTTAGGGTTTTAGGGTTAGGGTTTAGGGTTAGGGTTTAGGGTTTTAGGGTTTAGGGTTTAGGGTTTAGGGTTTAGGGTTTTAGGGTTTAGGGTTTTAGGGTTTAGGGTTTAGGGTTTAGGGTTTAGGGTTTAGGGTTTAGGGTTAGGGTTTAGGGTTTAGGGTTAGGTTTAGGGTTTAGGGTTTAGGGTTTAGGGTTAGGTTAGGGTTTAGGGTTTAGGGTTTAGGGTTTAGGGTTTAGGTTTAGGGTTTGGGTTTTTAGGGTTTAGGGTTTTTAGGGTTTAGGTTAGGGTTTAGGGTTTAGGGGGCTTTTAAGGGTTTAGGGTTAGGGTTTGGGTTAGGTTTAGGGTTTAGGTTAGGGTTTAAGGTTTAGGGTTTAGGGTTTAGGGTTTAGGGTTTAGTGTGTTTAGGGTTAGGGTTTAGGGTTTAGGGTTTAAGGGTTGTTTAGGGTTTAGGGTTTAGGGGTTTAGGGTTTAGGGTTTAGGGTTTAGGGTTTAGGTTTAGGGTTTAGGGTTTAGGGTTTAGGGGTTTAGGGTTTAGGGTTTAGGGTTAGGGTTTAGGGTTAGGGTTTAGGGTTTAGGGTTTAGGGTTTAGGGTTTAGGGTTTAGGGTTTAGGGTTTAGGGTTTAGGGGTTTAGGGTTTTAGGGTTTTAGGGTTTAGGTTAGGGTTTAGGGTTTTAGGTTTAGGGTTTAAGGGTTTAGGGTTTAGGGTTTAAGGGTTTAAGGGTTTAGGGTTTAGGTTTAGGGTTAGGGTTAGGGTTTAGGGTTTAGGGTTAGGGTTTAGGGTTTAGGGTTTTAGGGTTGGGTTTAGGGTTTAGGTTTAGGGTTTAGGGTTTAGGTTTAGGGATTAGGGGTTTAGGGTTTAGGTTAGGTTAGGTTAGGGTTTAGGTTTAGGGTTTAGGGTTTAGGTTTAGGGTTGGGGTTAAGGGTTTAAGGGTTTAGGGTTTTAGGGTTTTAGGTTAGGGTTTAGGGTTTAGGGTTTAGGGTTAGGGTTTTAGGGTTTTAAGGGTTTAGGGTTTAGGGTTTAGGGTTTAGGGTTTAGGGTTTAGGGTTAGGGTTTAGGGTTGGGGTTTAGGGTTAGGGTTTAGGTTTAGGGTTTAGGGTTTAGGGTTAGGGTTTAGGGTTTAGGGTTTAGGGTTAGGGTTAGGGTTAGGGTTGGGTTTAGGGTTTAGGGTTAGGGGTTTAGGGTTAGGGTTAGGGTTTAGGGTTAGGGTTAGGGTTTAGGGTTAGGGTTTAGGGTTTAGGGTTAGGGTTAGTTTAAGGGTTTAGGTTAGGTTTAGGTTTAGGTTTAGGGTTTAGGGTTAGGGTTTAGGGTTAGGGTTTAGGGTTTAGGGTTTAGGTTAGGGTTAGGGTTTAGGGTTTAGGGTTAGGGTTTTTAGGTTAGGGTTAGGTTTAGGGTTTAGGGTTTGGCGTTTAGGGTTAGGGTTTACGGGTTTAGGGGTTTAGGGTTTAGGGTTTAGGTTTAGGGTTTTAGGGTTAGGGTTTAGGGGTTTAGGGTTTAGGGGTTTAGGGTTTAGGTTTAGGTTTAGGGTTTAGGGTTTAGGGTTAGGGTTGGGTTGGGTTAGGGTTTTAAGGGTTTAGGGTTAGGTTTTAAGGGTTTTAGGGTTAGGGTTTTAAGGGTTAGGGTTTAGGGTTAGGGTTTTAGGGTAGGGTTTTGTTTAGGGTTAGGGTTAGGGTTTTAGGGTTTTAGGGTTTAGGGTTTAGGGTTTAGGGTTTAGGGGTTTAGGGTTTAGGGTTTAAGGGTTTAGGGTTTAGGTTAGGGTTTAGGGGTTTAGGGTTTAGGGTTTAGGTTTAGGGTTTAGGGTTTAGGGTTTAGGGTTTAGGGTTTAGGGTTTAGGGTTTAGGGTTTAGGGTTAGTTAGGGTTTTAGGGTTTTAGGGTTAGGGTTTAGGGTTTAGGGTTTAGGGTTAGGGTTTTAGGGTTTAGGGTTTAGGGTTTAAGGGTTTTAGTTAGGGTTTAGGGTTTAGGGTTAGGGTTAGGGTTTTAGGGTTTAGGGTTAGGGTTAGGGTTTAGGGTTAGGGTTAGGTAGGGTTTAGGGTTTAGGGTTTAGGGTTTAGGGTTAGGGTTTAGGGTTAGGGTTTGTTTAGGGTTTAGGGTTTAGGGTTTAGGGTTTAGGGTTTAGGGTTTAGGGTTTAAGGTAAGGGTTAGGTTAGGTTAGGGTTAGGGTTTTAGGGTTTATAGGGTTTTAGGTTTAGGGTTTAGGGTTTAGGGTTTAGGGTTAGGGTTAGGGTTAGGGTTAGGGTTTAGGGTTAGGGTTAAGGGTTTTAGGGTTTAGGGTTTAGGGTTTAGGGTTTAGGTTTAGGGTTTAGGGTTTAGGGGTTTAGGGTTTAGGGTTTAGGGTTTAGGGTTTAGGGTTAGGGGTTTAGGGTTTAGGGGTTTAGGTTTAGGGTTTAGGGTTTAGGGTTAGGGTTTAGGGTTTAAGGGTTTAGGGTTTAGGGTTTAAGGGTTTTAGGGTTTAGGGTTTAGGGTTTTAGGGTTTAGGGTTTAGGTTTAGGGTTTAGGGTTTAGGGTTTAGGGTTAGGGTTAGGGTTTAAGGGTTTTTAGGGTTTAGGGTTAGGTTTTAGGGTTTAGGGTAGGGTTAGGGTTTAGGGTTTTAGGGTTAGGGTTTAGGGTTAGGGTTTAGGGTTTAGGGTTTAGGGTTTAGGGTTAGGGTTTAGGGTTTAGGGTTAGGGTTTTAGGGTTTAGGGTTTAGGGTTAGGGTTAGGGTTTAAGGGTTTGGGGTTAGGGTTTAGGGTTAGGGTTTAGGGTTTAGGGTTTTAGGGTTTAGGGTTTAGGTTTAGGGGTTAGGGTTTAGGGTTGGGGTTTAGGGTTAGGGTTTAGGGTTTTAGGGTTTTAGGGTTTAGGGTTAGGGTTTTAGGGTTAGGGTTTAGGGTTTTAGGGTTTAGGGTTAGGGTTTAGGGTTTTAGGGTTAGGGTTTTAGGGTTTTTAGGGTTTAGGGTTTAGGGTTAGGGTTTAGGGTTTAGGGTTTAGGGTTTTAGGGTTTTAGGTTTAGGGTTTAGGGTTTAGGGTTAGGGTTTAGGGTTTTGGGTTAGGTTTTTAGGGTTTAGGGTTTAGGTTTAGGGTTTAGGGTTGGTTAGGGTTAGGGTTTAGGGTTTAGGGTTTAGGGTTTTAGGTTTAGGGTTTAGGGTTAGGTTTAGGGTTTTAGGTTTAGGGTTAGGTTTAGGTTTAGGGTTAGGGTTTGGGTTTAGGTTTAGGTTTAAGGTTTAGGGTTTAGGGTTTTAGGGTTAGGGTTTTAGGGTTAGGGAGTTTAGGGTTTAGGGTTAGGTTTAGGTTTTAGGTTTAGGGTTTAGGGTTAGGGTTTAGGGTTTAGGGTTAGGGTTTAGGTTTAGGTTTAGGGGTTTAGGGTTTAGGGTTTAAGGGTTTAGGGTTAGGGTTAGTTTAGGGTTAGGGTTTAGGGTTTAGGGTTTAGGGTTTTAGGGTTTTAGGGTTTAGGTTTAAGGGTTTAGGGTTTTAGGGTTTAGGGTTTATGTTTAGGGTTTAGGGTTTAGGTTTAGGGTTTAGGGTTTTAGGGTTTAGGGTTAGGTTAAGGTTTAGGGTTAGGGTTAGGGTTTAGGGTTTTAGGTTTAGGGTTTAGGGTTTAGGATTTAGGGTTTAGGGTTTAGGTTAGGGTTTAGGGTTTAGGTTTAGGGGTTTAGGGTTTAGGGTTTAGG

***T. aestivum* cv. ArinaLrFor**

**4AS**

GGG**TTTAGGG**TTTAGGGTAAGGGTTTGTGTTTAGGGTTTATGTTTTAGGGTTTAGGGTTTAGGGTTTAGGGTTTAGGGTTTAGGGTTTAGGTTTAGGGTTTAGGGTTTAGGGTTTAGGGTTAGGGTTTAGGGTTAGGGTTAGGGTTTAGGGTTTAGGGTTTAG

**6DS**

ATGGTTGGGTTAGGG**TTTAGGG**TTTAGGGTTTAGGGTTTAGGGTTTAGGGTTTAGGGTTTAGGGTTTAGGGTTTAGGGTTTA

***T. aestivum* cv. LongReach Lancer**

**1AS**

TATGGTTTAGGTAGGTTTAGGTTAGGG**TTTAGGG**TTTAGGGGTTTAGGGTTTAGGGTTTTGGTTAGGGTTTAGGGTTAGGGGTTTAGGGTTTAGGGTTTAGGGTTTAGGGTTTAGGTTTAGGGTTTAGGGTTTTT

**1BS**

GGG**TTTAGGG**TTTAGGGTTAGGGTTTAGGGTTTAGGGTTATGGTTTTAGGGTTTAGGGTTTAGGGTTTAGGGTTTAGGGTTTAGGTTTTAGGGTTTAGGGTTA

**4BS**

**TTTAGGG**TTAGGTTTTAGGGTTTAGGGTTTAGGGTTTAGGGTTAGGGTTTAGGGTTTAGGGTGTAGGGTTTAGGGTTTAGGGTTTAGGGTT

**7DS**

CCCGGG**TTTAGGG**TTTAGGGTTTAGGGTTTAGGGTTTAGGGTTTAGGGTTAGGGTTT

***T. aestivum* cv. CDC Landmark**

**1AS**

TATGGTTTAGGTAGGTTTAGGTTAGGG**TTTAGGG**TTTAGGGGTTTAGGGTTTAGGGTTTTGGTTAGGGTTTAGGGTTAGGGGTTTAGGGTTTAGGGTTTAGGGTTTAGGGTTTAGGTTTAGGGTTTAGGGTTTTTTAGGGTTTAGGGT

**2AS**

GG**TTTAGGG**TTTAGGGTTTAGGGTTTAGGGTTTAGGGTTTAGGGTTTAGGGTTTAGGGTTTAGGGTTTAGGGTTTAGGGTTTAGGGTTTAG

***T. aestivum* cv. Mattis**

**2AS**

GGAGGG**TTTAGGG**TTTAGGGTTTAGGGTTTAGGGTTTAGGGTTAGGGTTTAGGAGTTAGGGTTTAGGGTTTAGGGTTTAGGGTTTAGGGTTTAGGGTTTAGGGTTTAGGGTT

**3AS**

TTTGAGGG**TTTAGGG**TTTAGGGCTTAGGGTTTAGGGTTTAGGGTTAGGGTTAGGGTTAGTGTTAGGGTTTAGGTTTAGGGTTTAGGGTTAGGGTTTAGGGTTTAG

**7AS**

TTAAGGG**TTTAGGG**TTTAGGGTTAGGGTTTAGGGTTTAGGGTTTAGGGTTAGGGTTTAGGGTTTTGGGGTTTAGGGTTTAGGGTTTAGGGTTTAGGGTTAGGGTTTAGGGTTTAGGGTTTAGGGTTTAGGG

**1BS**

AGGG**TTTAGGG**TTTAGGGTTTAAGGGTTTAGGGTTAGGGTTTAGGGTTTAGGGTTTAGGGTTTAGGGTTAGGGTTAGGGTTTAGGGTTTAGGGTTAGGGTTTAGGGTTTAGGGTTTAGGGTTTAGGGTTTAGG

**3BS**

GGG**TTTAGGG**TTTAGGGTTTAGGGTTTAGGGTTTAGGGTTTAGGGTTTAGGGTTTTTAGGGTTTAGGGTTTAGGGTTTA

***T. aestivum* cv. Stanley**

**2AS**

GGAGGG**TTTAGGG**TTTAGGGTTTAGGGTTTAGGGTTTAGGGTTAGGGTTTAGGAGTTAGGGTTTAGGGTTTAGGGTTTAGGGTTTAGGGTTTAGGGTTTAGGGTTTAGGGTTTAG

**4BS**

AG**TTTAGGG**TTTAGGGTTTAGGGTTAGGGTTTAGGGTTTAGGGTTTAGGGTTTAGGGTTTAGGGTTTAGGGTTAGGGTTTAAGG

***T. aestivum* cv. Chinese Spring**

**1AS**

GGTTTAGGTAGGTTTAGGTTAGGG**TTTAGGG**TTTAGGGGTTTAGGGTTTAGGGTTTTGGTTAGGGTTTAGGGTTAGGGGTTTAGGGTTTAGGGTTTAGGGTTTAGGGTTTAG

**4AS**

TTTTGGG**TTTAGGG**TTTAGGGTTTAGGGTTTAGGGTTTAGGGTTTAGGGTTTAGGGTTTAGGGTTAGGGTTTAGGGTTTAGGGTTTAGGGTT

**6AS**

G**TTTAGGG**TTTAGGGTTTAGGGTTTAGGGTTTAGGGTTTAGGGTTTAGGGTTTTTAGGGTTTAGG

**7AS**

GTTAAGGG**TTTAGGG**TTAGGGTTAGGGTTAGGGTTAGGGTTAGGGTTAGGGTTAGGGTTTAGGATTTAGGGTTTAGGGTTTAGGGTTTAGGGTTTAGGGTTTAGGGTTTAGGGTTTAGGGTTAGGGTTTAGGGTTTAGGGT

**6BS**

CCG**TTTAGGG**TTTAGGGTTTAGGGTTTAGGGTTTAGGGTTAGGGTTAGGGTTTAGGGTTTAGGGTTTAGGGTTTTAGGGTTTAGGGTTTAGCGTTTAGGGTTTAGGGTTTAGGGTTAGGGTTTAGGGTTTAGGGTTTAGGGTTTAGGGTT

**7BS**

GGAATAGG**TTTAGGG**TTTAGGGTTTAGGGTTTTTAGGCTTTAGGGTTTAGGGTTTAGGGTTTAGGGTTTTTAGGGTTTAGGGTTTAGGGTTTAGGGTTTACGGTTTAGGGTTTAGGGTT

**7DS**

TTTGTGAACCCGGG**TTTAGGG**TTTAGGGTTTAGGGTTTAGGGTTTAGGGTTTAGGGTTTAGGGTTAGGGTTTAGGGTTTAGGGTTTAGGGTTTAGGGTT

***T. aestivum* cv. Norin-61**

**4AS**

TTTTGGG**TTTAGGG**TTTAGGGTTTAGGGTTTAGGGTTTAG

***T. aestivum* cv. Fielder**

**1AS**

GGTTTAGGTAGGTTTAGGTTAGGG**TTTAGGG**TTTAGGGGTTTAGGGTTTAGGGTTTTGGTTAGGGTTTAGGGTTAGGGGTTTAGGGTTTAGGGTTTAGGGTTTAGGGTTTAGGTTTAGGGTTTAGGGTTTTTTAGGGTTTAGGGTTTAGGTTTAGGGTTTAGGGTTTTGGGTTTAGGGTTTAGGTTTTAGGGTTTAAGGTTTAGGGTTTAGGGTTTAGGGTTAGGGTTTAGGGTTTAGGGTTTAGGGTTTAGGGTTTAGGGTTAGGGTTTAGGGTTTAGGGTTTAGGTTTAGGGTTTAGGGTTTAGGGTTTAGGGGTTTAGGGTTTAGGGTTTAGGGTTGGGTTTAGGGTTTAGGGTTTAGGGTTTAGGGTTTAGGGTTTAGGGTTTAGGGTTAGGGTTTAGGGTTTAGGGTTTAGGGTTTAGGGTTTAGGTTTAGGGTTTAGGGTTTAGGGTTTAGGGGTTTAGGTTTAGGGTTTTAGGGTTTAGGGTTAGGTTTAGGGTTTAGGGTTTAGGGTTTAGGGTTTAGGGTTTAGGGTTTAGGGTTTAGGGTTTAGGGTTTAGGGTTGTAGGGTTTAGGGTTTAGGGTTTAGGGTTTAGGGTTTAGGGTTTAGGGTAGGGTTAGGGTTTAGGGTTTAGGGTTTAGGGTTTAGGGTTTTAGGGTTTAGGGTTTAGGGTTTAGGGTTTAGGGTTTAGGGTTTAGGGTTAGGGTTAGGGTTTAGGGTTTAGGGTTTAGGGTTTAGGGTTTAGGGTTTAGGGTTTAGGGTTTAGGGTTTAGGGTTTAAGGGTTTAGGGTTTAGGGTTTAGGGTTTAGGGTTTAGGGTTTAGGGTTTAGGGTTTAGGGGTTTAGGGTTTAGGGTTTAGGGTTTAGGGTTTAGGGTTTAGGGTTTAGGGTTTAGGGTTAGGGTTTAGGGTTTAGGGTTAGGGTTTAGGGTTTAGGGGTTTAGGGTTAGGGTTAGGGTTTAGGGTTTAGGGTTTTAGGGTTAGGGTTTAGGGTTTAGGGTTTAGGGTTTAGGGTTTAGGGTTTAGGGTTTAGGGTTTAGGTTTAGGGTTAGGGTTTAGGGGTTTAGGGTTTAGGGTGTTAAGGGTTTAGGGTTTAGGGTTTAGGGTTTAGGGTTTAGGGTTTAGGGTTTAGGGTTAGGGTTTAGGGTTAGGGTTTAGGGTTTAGGGTTTAGGGGTTAGGTTTAGGGTTTAGGGTTTAGGGTTTAGGGTGGGTTTAGGGTTTAGGGTTTAGGGTTTAGGGTTTAGGGTTTAGGGGTTTAGGGTTTAGGGTTTAGGGTTTAGGGTAGGGTTAGGGTTTGGGTTTAGGGTTTAGGGTTTAGGGTTTAGGGTTTAGGGGTTAGGGTTTAGGGTTTAGGGTTTAGGGTTAGGGTTAGGGTTTTAGGGTTTAGGGTTTAGGGGTTTAGGGTTAGGGTTG

**2AS**

GG**TTTAGGG**TTTAGGGTTTAGGGTTTAGGGTGTGTAGGGTTTAGGGTTTAGGGTTTAGGGTTTAGGGTTTAGGGTTTAGGGTTTAGGGTTTAGGGTTTAGGGTTTAGGGTTTAGGGTTTAGGGTTTAGGGTTTAGGGTTTAGGGTTAGGGTTTAGGGTTTAGGGTTTAGGGTTTAGGGTTTAGGGTTTAGGGTTTAGGGTTTAGGGTTTAGGGTTTAGGGTTTAGGGTTTAGGGTTTAGGGTTTAGGGTTTAGGGTTTAGGGTTTAGGGTTTAGGGTTTAGGGTTTAGGGTTTAGGGTTTAGGGTTTAGGGTTAGGGTTTAGGGTTTAGGGTTTAGGGTTAGGGTTTGGGTTAGGGTTTAGGGTTAGGGTTTAGGGTTGGGTTTAGGGTTAGGGTTAGGGTTAGGGTTTAGGGTTAGGGTTTAGGGTTTAGGGTTTAGGGTTTAGGGTTTAGGGTTTAGGGTTTAGGGTTTAGGGTTTGGGTTTAGGGTTTAGGGTTTAGGGTTTAGGGTTTAGGGTTTAGGGTTTAGGGTTTAGGGTTTAGGGTTTAGGGTTTAGGGTTTAGGGTTTAGGGTTTAGGGTTTAGGGTTTAGGGTTTAGGGTTTAGGGTTTAGGGTTTAGGGTTTAGGGTTTAGGGTTTAGGGTTTAGGGTTTAGGGTTTAGGGTTTAGGGTTTAGGGTTTAGGGTTGGGTTTAGGGTTGTTAGGGTTTAGGGTTAGGGTTTAGGGTTTAGGGTTTAGGGTTTAGGGTTAGGGTTAGGGTTAGGGTTTAGGGTTTAGGGTTTAGGGTTTAGGGTTTAGGGTTTAGGGTTTAGGGTTAGGGTTTAGGGTTAGGGTTTAGGATTAGGGTTTAGGGTTTAGGGTTTAGGGTTTAGGGTTTAGGGTTTAGGGTTTAGGGTTTAGGGTTTAGGGTTTAGGGTTTAGGGTTAGGGTTAGGGTTTAGGGTTTAGGGTTTAGGGTTTAGGGTTAGGGTTTAGGGTTAGGGTTTAGGGTTGTTAGGGTTTAGGGTTTAGGGTTTAGGGTTTAGGGTTTAGGGTTTAGGGTTAGGGTTTAGGGTTTAGGGTTTAGGGTTTAGGGTTTAGGGTTTAGGGTTTAGGGTTTAGGGTTAGGGTTTAGGGTTTAGGGTTTAGGGTTTAGGGTTTAGGGTTTAGGGTTTAGGGTTAGGGTTTAGGGTTTAGGGTTTAGGGTTTGGGTTAGGGTTTAGGGTTTAGGGTTTAGGGTTTAGGGTTTAGGGTTTAGGGTTAGGGTTTAGGGTTTAGGGTTTAGGGTTTAGGGTTTAGGGTTTAGGGTTTAGGGTTTAGGGTTTAGGGTTTAGGGTTTAGGGTTTAGGGTTTAGGGTTTAGGGTTTAGGGTTTAGGGTTTAGGGTTTAGGGTTAGGGTTTAGGGTTTAGGGTTTAGGGTTTAGGGTTTAGGGTTTAGGGTTTAGGGTTTAGGGTTTAGGGTTTAGGGTTTAGGGTTTAGGGTTTAGGGTTTAGGGTTTAGGGTTTAGGGTTTAGGTTTAGGGTTTAGGGTTTAGGGTTTAGGGTTTAGGGTTTAGGGTTTAGGGTTTAGGGTTTAGGGTTTAGGGTTTAGGGTTTAGGGTTTAGGGTTTAGGGTTTAGGGTTTAGGGTTTAGGGTTTAGGGTTTAGGGTTTAGGGTTTAGGGTTTAGGGTTTAGGGTTTAGGGTTTAGGGTTTAGGGTTTAGGGTTTAGGGTTTAGGGTTTAGGGTTTAGGGTTTAGGGTTTAGGGTTTAGGGTTTAGGGTTTAGGGTTTAGGGTTTAGGGTTTAGGGTTAGGGTTTAGGGTTTAGGGTTTAGGGTTTAGGGTTTAGGGTTTAGGGTTTAGGGTTTAGGGTTTAGGGTTAGGGTGGTGATAGGGTTAGGGTTTAGGGTTAGGGTTTAGGGTTTAGGGTTTAGGGTTTAGGGTTTAGGGTTTTAGGTTTAGGGTTAGGGTTTAGGGTTTGGGTTTAGGGTTTAGGGTTTAGGGTTTAGGGTTAGGGTAGGGGTTTGGGTTAGGGTGGTTTAGGGTTTAGGGTTTAGGGTTTAGGGTTTAGGGTTTAGGGTTTAGGGTTTAGGGTTTAGTGGTTTAGGGTTTTAGGGTTTAGGGTTTAGGGTTTTTAGGGTTTAGGGTTTAGGGTTTAGGGTTTGGTTTAGGGTTAGGGTTAGGTAGGGTTTGGGTTTAGGGTTGTAGGGTTTAGGGTTTAGGGTTAGGGTTTAGGGTTAGGGTTTAGGGTGGTTAGGGGTTTGGGTAGGGTTTGGTTTTAGGGTTTAGGGTTTAGGGTTTAGGGTTTAGGGTGGTTTAGGGTTGGGTTGGGGTTAGGGTAGGGTGAGGGTTTAGAGGGTTAGGGGTGGGTTTAGGGTTTAGGGTTTTGGGTGGGTTTAGGGTTAGCGCCTGCTACATCGAGGGTGTAGGGGTGTGTGAGTACCAGTTATCAACAGTTTCCAAGTTACCACGTTTAAAGTTCAACGTTTAACGTTCAAAGTTTAAGTCAGTTAAGATTAAAGTTTTAAGTTTAAGTTTAAAGGTTTAAAGTTTAAAGTTTAAGTAAGTTTAAAGTTAAAGTTAACCGTTTAAAGTTTTTAAGTTAAGTTTAAACGTCTACCCGTAAAGTTCTAACAGTTTAACGTTTAAAGGATTACAGTTTAAAGTTTACAGTTTTTCAAGTTTAAGTTCTAAAGTTAAAGTTTTAAAGTTTAAAGTTTAAAGTTTAAA

***T. aestivum* cv. Attraktion**

**2AS**

ACTGGAGCGG**TTTAGGG**TTTAGGGTTTAGGGTTTAGGGTTTAGGGTTTAGGGTTTAGGGTTTAGGGTTTAGGGTTTTAGGGTTTAGGGTTTAGGGTTAGGGTTTAGGGTTTAGGGTTTAGGGTTTAGGGTTTAGGGTTAGGGTTTAGGGTTTAGGGTTTAGGGTTTAGGGTTTAGGGTTTAGGGTTTAGGGTTTAGGGTTAGGGTTTAGGGTTTAGGGTTTAGGGTTTAGGGTTTAGGGTTTAGGGTTTAGGGTTTAGGGTTTAGGGTTAGGGTTTAGGGTTTAGGGTTTAGGGTTTAGGGTTTAGGGTTTAGGGTTTAGGGTTAGGGTTTAGGGTTTAGGGTTTAGGGTTTAGGGTTTAGGGTTTAGGGTTTAGGGTTAGGGTTTAGGGTTAGGGTTTAGGGTTTAGGGTTAGGGTTAGGGTTTAGGGTTTAGGGTTTAGGGGTTAGGGTTTAGGGTTTAGGGTTAGGGTTAGGGTTTAGGGTTTAGGGTTTAGGGTTAGGGTTTAGGGTTTAGGGTTTAGGGTTTAGGGTTTAGGGTTAGGGTTAGGGTTAGGGTTTAGGGTTAGGGTTAGGGTTTAGGGTTTAGGGTTTAGGGTTAGGGTTTAGGGTTTAGGGTTTAGGGTTTAGGGTTTAGGGTTTAGGGTTTAGGGTTTAGGGTTTAGGGTTTAGGGTTTAGGGTTTAGGGTTTAGGGTTTAGGGTTTAGGGTTTAGGGTTTAGGGTTTAGGGTTTAGGGTTTAGGGTTTAGGGTTTAGGGTTTAGGGTTTAGGGTTTAGGGTTAGGGTTTAGGGTTTAGGGTTTAGGGTTTAGGGTTAGGGTTTAGGGTTTAGGGTTTAGGGTTTAGGGTTTAGGGTTAGGGTTTAGGGTTAGGGTTTAGGGTTTAGGGTTTAGGGTTTAGGGTTTAGGGTTTAGGGTTTAGGGTTAGGGTTAGGGTTTAGGGTTTAGGGTTTAGGGTTTAGGGTTTAGGGTTTAGGGTTTAGGGTTTAGGGTTAGGGGGTTTAGGGGTTTAGGGTTAGGGTTGGGTGA

**3AS**

TTGGGTTGGG**TTTAGGG**TTAGGGGTAGGGTTTAGGGTTTAGGGTTAGGGTTGGGTTTAGGTTAGGGTTAGGGTTTAGGGTTAGGGTTGAGGGTTTAGGGTTTAGGGTTAGGTTTAGGGTTGAGGGGTTTAGGGTTGAGGGTTGGGAGGGTTTAGGGTTGGTTAGGGTTTAGGGTTAGGTTAGGGTTGAGGGTTTAGGGTTAGGTTAGGGTTTAGGGTTAGGGTTAGGTTTAGGGTTGGTTAGGGTTTTAGGTTTAGGGTTTAGGGTTAGGGTTTAGGGTTAGGGTTGGTTTAGGGTGGTTTAGGGTTTAGTTGGGTTTAGGGTTGGTTAGGGTTAGGGTTTAGGGTTAGGTTTAGGGTTTAGGGTTTAGGGTTTAGGGTTTAGGGTTTAGGGTTTAGGGTTTAGGGTTAGGGGTTTAGGGTTTAGGGTTAGGGTTGTAGGGTTTTAGGGTTTAGGGTTTAGGGTTAGGGTTTTAGGGTTTAGGGTTTAGGGTTTAGGGTTTGAGGGTTAGGGGTTTAGGGTTTAGGGTTTAGGGTTTAGGGTTTAGGGTTAGGGTTAGGGTTTAGGGTTTAGGGTTTGTGGTTTAGGGTTTAGGGTTGAGGTTTAGGGTTTAGGGTTCTTTAGGGTTTAGGGTTTAGGGTTTAGGTTAGGGTTTAGGGTTTAGGGTTTAGGGTTAGGGTTTAGGGTTTAGGGTTAGGGCTTAGGGTAGTTAGGGTATAGGGTTTTAGGGATTAGGGTTTAGGGTTTAGGGTTTAGGGTTTAGGGTTTAGGGTTTAGGGTTTAGGGTTTAGGGTTTAGGTTTAGGGTTTAGGGTTTTGGTTTAGGGTTTAGGGTTTAGGGTTTGGGTTTAGGGTTTAGGGTTAGGGTTTAGGTAGGTTTAGGGTTTAGGGTTGTTTAGGGTTTAGGGTTTAGGGTTTAGGGTTTAGGGTTTAGGGTTTAGGGTTTAGGGTTTAGGGTTAGGGTTTAGGGTTTAGGGTTTAGGGTTAGGGTTTAGGGTTTAGGGTTTAGGGTTTAGGGTTTAGGGTTTAGGGTTAGGGTTTAGGGTTTAGGGTTTAGGGTTTAGGGTTTAGGGTTTAGGGTTTAGGGTTTAGGTTTAGGGTTTAGGTTTAGGGTTTAGGGTTTAGGGTTTAGGTTTAGGGTTAGGGTTTAGGGTTTAGGGTTTAGGGTTAGGGTTAGGGTTTAGGGTTTAGGGTTTAGGGTTAGGGTTTAGGGTTTAGGGTTTAGGGTTAGGGTTTAGGGTTAGGGTTTAGGGTTAGGGTTTAGGGTTTAGGGTTTAGGGTTTAGGGTTTAGGGTTTAGGGTTAGGGTTTAGGGTTTAGGGTTTAGGTTTAGGGTTTAGGGTTTAGGGTTTAGGGTTTAGGGTTAGGGTTTAGGGTTTAGGGTTTAGGGTTTAGGGTTAGGGTTTAGGGTTTTAGGGTTTAGGGTTTAGGGTTTAGGGTTTAGGGTTTAGGGTTTAGGGTTTAGGGTTTAGGGTTTAGGGTTTAGGGTTTAGGTTTAGGGTTTAGGGTTTAGGGTTTAGGGTTTAGGGTTTAGGTTTAGGGTTTAGGGTTTAGGGTTTAGGGTTTAGGGTTTAGGGTTTAGGGTTTAGGGTTTAGGGTTTTAGGGTTTAGGGTTTAGGGTTTTAGGGTTTAGGGTTTAGGGTTAGGGTTTAGGGTTTAGGGTTTAGGGTTTAGGGTTTAGGGTTTAGGGTTTAGGGTTTAGGGTTTAGGGTTAGGGTTTAGGGTTTTAGGGTTTTAGGGTTTTAGGGTTAGGTTTAGGGTTTAGGGTTTAGGGTTTAGGGTTTTAGGGTTTAGGGTTAGGGTTTAGGGTTTAGGTTTAGGGTTTAGGGTTTAGGTTTAGGGTTTAGGGTTTAGGGTTTAGGGTTTAGGGTTTAGGGTTTAGGGTTTAGGGTTTAGGGTTTAGGGTTTAGGGTTTAGGGTTTAGGGTTTAGGGTTTAGGGTTTAGGGTTTAGGGTTTAGGGTTTAGGGTTTAGGTTTAGGGTTTAGGGTTTAGGGTTTAGGGTTTAGGGTTTAGGGTTTAGGGTTTAGGGTTAGGGTTTAGGGTTTAGGGTTTAGGGTTAGGGTTTGGGTTTAGGGTTTAGGGTTAGGGTTTAGGGTTTAGGGTTTAGGTTTAGGGTTTAGGGTTTAGGGTTAGGGTTTAGGGTTTAGGGTTTAGGGTTTAGGGTTAGGTTTAGGGTTTAGGGTTTAGGGTTTAGGGTTTAGGGTTTAGGGTTTAGGGTTAGGGTTTAGGGTTAGGGTTTAGGGTTTAGGGTTTAGGGTTTAGGGTTTAGGGTTTAGGGTTTAGGGTTTAGGGTTTAGGGTTTAGGGTTTAGGGTTTAGGGTTTAGGGTTTAGGGTTTAGGGTTTAGGGTTTAGGGTTAGGGTTTTAGGGTTTAGGGTTTAGGGTTTAGGGTTTAGGGTTTAGGGTTTAGGGTTTAGGGTTTAGGGTTTAGGGTTTAGGGTTTAGGGTTTAGGTTTAGGGTTTAGGGTTTAGGGTTAGGGTTTAGGGTTTAGGGTTTAGGGTTAGGGTTTAGGGTTTAGGGGTTAGGGTTTAGGGTTTAGGGTTTAGGTTTAGGGTTAGGGTTTAGGTTTAGGGTTTAGGGTTTGGGTTTAGGGTTTAGGGTTTAGGTTAGGGTTAGGGTTTAGGGTTTAGGGTTTAGGGTTTAGGGTTTAGGGTTTAGGGTTTAGGGTTTAGGGTTAGGGTTAGGGTTAGGGTTTAGGGTTAGGGTTTAGGGTTAGGGTTTAGGGTTTAGGGTTAGGGTTTAGGTTTAGGGTTTAGGGTTTAGGGTTTAGGGTTTGGGTTTAGGGTTAGGGTTTAGGGTTAGGGTTTAGGGTTAGGGTTTAGGGTTTAGGGTTAGGGTTTAGGGTTTAGGGTTTAGGGTTAGGGTTAGGGTTTAGGGTTTAGGGTTTAGGGTTTAGGGTTTAGGGTTTAGGGTTTTAGGGTTTAGGGTTAGGGTTTAGGGTTTAGGGTTTAGGGTTTAGGGTTTAGGGTTTAGGGTTAGGGTTTAGGGTTTGGGTTTAGGGTTTAGGGTTTAGGGTTAGGGTTTAGGGTTTAGGGTTTAGGGTTTAGGGTTTAGGGTTTAGGGTTAGGGTTTAGGGTTTAGGGTTGTTAGGGTTTAGGGTTTAGGGTTTAGGGTTAGGGGTTTAGGGTTTAGGGTTTAGGGTTTAGGGTTTAGGTTAGGGTTTAGGGTTGGTAGGGTTTAGGGTTTAGGGTTTAGGGTTGTAGGGGTTAGAGGTTTAGGGTTTAGGGTTTAGGGTTTAGGGTTAGGGTTTAGGGTTAGGGTTAGGGTTTAGGGTTTAGGGTTTAGGTTTAGGGTTTAGGGTTTAGGGTTTAGGGTTTAGGGTTAGGGTTTAGGGTTTAGGGTTTAGGGTTTAGGGTTTAGGGTTAGGGTTTAGGGTTAGGGTTTAGGGTTAGGGTAGGGTTTAGGGTTTAGGGTTGGGTAGGGTTTAGGGTTTAGGGTTAGGGTTTAGGGTTTAGGGTTTAGGGTTTAAGGGTTTAGGGTTTAGGTTTAGGGTTAGGGTTAGGGTTTAGGGGTTTAGGGTTTAGGGTTTAGGGGTTTAGGGTTTAGGGTTTGGGTTTAGGGGTTAGGGTTAGGGTTTAGGGTTTAGGGTTTAGGGTTTAGGGTTTAGGGTTTAGGGTTAGGGTTTAGGGTTAGGGTTTGGGGTTTAGGGTTAGGGTTTAGGGGTTTAGGGTTTAGGGTTAGGGGTTTAGGGTGTTTAGGGTTTAGGGTTTAGGGTGTTAAGGGTTTAGGGTTTAGGGTTAGGGTTTAGGGGTTTAGGGTTAGGGGTTAGGGTTTAGGGTTTAGGGTTTAGGGTTTAGGGTTAGGGTTTAGGGTTTAGGGTTTAGGGTTTAGGGTTAGGGTTAGGGTTAGGGTTTAGGGTTAGGGTTTAGGGTTTAGGGTTAGGGTTTAGGGTTTAGGGTTGGTTTAGGGTTTAGGGTTTAGGGTTTAGGGTTAGGGTTTTAGGGTTTAGGGTTTAGGGTTTAGGGTTTAGGGTTTAGGGTTAGGGTTTAGGGTTTAGGGTTTAGGGTTTAGGGTTTAGGGTTAGGGTTTAGGGTTTAGGGTTTAGGGTTTAGGGTTTGGGTTTAGGGGTTTGGGGTTTGGGTTTTAGGGTTAGGGTTTAGGGTTTAGGGTTTAGGGTTTAGGGTTTAGGGTTTAGGGTTTAGGGTTTAGGGTTAGGGTTTAGGGTTAGGGTTTTGGGTTTAGGGTTTAGGGTTTAGGGTTTAGGGTTTAGGGTTTAGGGTTTAGGGTTTAGGGTTTAGGGTTTAGGGTTAGGGTTTAGGGTTTAGGGTTTAGGGTTTAGGGTTTAGGGTTAGGGTTTAGGGTTAGGGTTTAGGGTTAGGGTTTAGGGTTTAGGGTTTAGGGTTTAGGGTTTTAGGTGGGTTTAGGGTTTAGGGTTTAGGGTTTAGGGTTTAGGGTTTAGGGTTTAGGGTTAGGGTTTAGGGTTAGGGTTTAGGGTTTAGGGTTTAGGGTTTAGGGTTTAGGGTTTAGGGTTTAGGGTTTAGGGTTTAGGGTTAGGGTTTAGGGTTTAGGGTTTAGGGTTTAGGGTTTAGGTTTTAGGGTTTAGGGTTTAGGGTTTAGGGTTTGGGTTTAGGGTTTAGGGTTAGGGTTTAGGGTTTAGGGTTTAGGGTTTAGGGTTAGGGTTTAGGGTTTAGGGTTGTAGGGTTTAGGGTTTAGGGTTTAGGGTTTTAGGGTTTAGGGTTTAGGGTTAGGGTTTAGGGTTTAGGGTTTAGGGTTTAGGGTTTAGGGTTAGGGTTTAGGGTTTAGGGTTAGGGTTTAGGGTTTAGGGTTGGGTTAGGGTTTAGGGTTTAGGGTTAGGGTTTAGGGTTTAGGGTTTAGGGTTTAGGGTTTAGGGTTTAGGGTTAGGGTTTAGGGTTTAGGGTTAGGGTTTAGGGTTTAGGGTTTAGGGTTTAGGGTTTAGGGTTAGGGTTTAGGGTTTAGGGTTTAGGGTTGGGTTTAGGGTTTAGGGTTTAGGGTTAGGGTTTAGGGTTTAGGGTTTAGGGTTTAGGGTTTAGGGTTTAGGGTTTAGGGTTTTGGGTTTAGGGTTTAGGGTTTAGGGTTTAGGGTTTAGGGTTTAGGGTTTAGGTTTAGGGTTTAGGGTTTAGGGTTTAGGGTTTAGGGTTTAGGGTTTAGGGTTTAGGGTTTAGGGTTTAGGGTTTAGGGTTTAGGGTTTAGGGTTTAGGGTTTAGGGTTTGGGTTTAGGGTTTAGGGTTTAGGGTTTAGGGTTTAGGGTTTAGGGTTAGGGTTTAGGGTTTAGGGTTTAGGGTTTAGGGTTTAGGGTTTAGGGTTTAGGGTTTTGGGTTTAGGGTTTAGGTTTAGGGTTTAGGGTTTAGGGTTTAGGGTTAGGGTTTAGGGTTTAGGGTTTAGGGTTTAGGGTTTAGGGTTTAGGGTTTAGGGTTTAGGGTTTAGGGTTTAGGGTTAGGGGTTTAGGGTTTAGGGTTAGGGTTAGGGTTTAGGGTTTAGGGTTTAGGGTTTAGGGTTTAGGGTTAGGGTTTAGGGTTTAGGGTTTAGGGTTTAGGGTTTAGGGTTTAGGGTTTAGGGTTTAGGGTTTAGGGTTTAGGGTTAGGGTTTAGGGTTTAGGGTTTAGGGTTTAGGGTTTAGGGTTTAGGGTTTAGGGTTTAGGGTTTAGGGTTTAGGGTTTTAGGGTTTGGGTTTAGGGTTTAGGGTTTAGGGTTTAGGGTTTAGGGTTTAGGGTTAGGGTTTAGGGTTTAGGGTTTAGGGTTTAGGGTTTAGGGTGGGTTTAGGGTTTAGGGTTTAGGGTTTAGGGTTTAGGGTTTAGGGTTTAGGGTTAGGGTTTAGGGTTTAGGGTTTAGGGTTTAGGGTTTAGGGTTTAGGGTTTAGGGTTTAGGGTTTAGGGTTAGGTTTAGGGTTTAGGGTTTAGGGTTAGGGTTTAGGGTTTAGGGTTTAGGGTTTAGGGTTTGGGTTTAGGGTTTAGGGTTTAGGGTTTAGGGTTTAGGGTTTTGGGTTTAGGGTTTAGGGTTAGGGTTTAGGGTTTAGGGTTTAGGGTTTGGTTTAGGGTTTAGGGTTAGGGTTTAGGGTTTAGGGTTTAGGGTTTAGGGTTAGGGTTTAGGGTTTAGGGTTTGGGTTTAGGGTTTAGGGTTTAGGGTTTAGGGTTTAGGGTTAGGGTTTAGGGTTTAGGGTTTAGGGTTTAGGGTTTAGGGTTTGGGTTAGGGTTTAGGGTTTAGGGTTTTAGGGTTTAGGGTTTAGGGTTTAGGGTTTAGGGTTTAGGGTTTAGGGTTTAGGGTTTAGGGTTTAGGGTTTAGGGTTTAGGGTTTAGGGTTTAGGGTTTAGGGTTTAGGGTTTAGGGTTTAGGGTTTAGGGTTTAGGGTTTAGGGTTTAGGGTTTAGGGTTTAGGGTTTAGGGTTTAGGGTTTAGGGTTTAGGGTTTAGGGTTTAGGGTTTTAGGGGTTTAGGGTTAGGGTTTAGGGTTTAGGGTTTAGGGTTTAGGGTTTAGGGTTTAGGGTTTAGGGTTTAGGGTTTAGGGGTTAGGGTTTAGGGTTTAGGGTTAGGGTTTAGGGTTAGGGTTTAGGGTTTAGGGTTTAGGGTTTAGGGTTTAGGGTTTAGGGTTAGGTTTAGGGTTTAGGGTTTAGGGTTTAGGGTTTAGGGTTTAGGGTTTAGGGTTTAGGGTTTAGGGTTTAGGGTTTAGGGTTTGGGTAGGGTTTAGGGTTTAGGGTTTAGGGTTTAGGGTTTAGGGTTTAGGGTTAGGGTTAGGGTTTAGGGTTTAGGGTTTAGGGTTTAGGGTTTAGGGTTTAGGGTTTAGGGTTAGGGTTTAGGGTTTAGGGTTAGGGTTTAGGGGTGGGTTTAGGGTTAGGGTTTAGGGGTTTAGGGTTTAGGGTTTGGGTTTAGGGTTGGTTAGGGTTTAGGGTTTAGGGTTTAGGGTTTAGGGTTTAGGGTTTAGGGTTTTGGGTTTAGGGTTTGGGTTTAGGGTTGTTAGGGTTTAGGGTTTAGGGTTTAGGGTTTAGGGTTTAGGGTTTAGGGTTTAGGGTTTAGGGGTTTAGGGTTTAGGGTTTAGGGTTTAGGGTTTAGGGTTAGGTTTAGGGTTTAGGGTTTAGGGTTTAGGGTTTAGGGTTTAGGGTTTAGGGTTTAGGGTTTAGGGTTTAGGGTTGGGTTTAGGGTTTAGGGTTTAGGGTTTAGGGTTTAGGGTTTAGGGTTTAGGGTTAGGGTTTAGGGTTTAGGGTTTAGGGTTTAGGGTTTAAGGGTTTAGGGTTTAGGGTTTAGGGTTTAGGGTTTAGGGTTAGGGTTTAGGGTTTAGGGTTTAGGGTTTAGGGTTTAGGGTTTAGGGTTTAGGGTTAGGGTTTAGGGTTTAGGGTTTAGGGTTTAGGGTTTAGGGTTTAGGGTTTAGGGTTTAGGGTTTAGGGTTTAGGGTTTAGGGTTAGGGTTTAGGGTTTAGGGTTTAGGGTTTAGGGTTTAGGGTTTAGGGTTTAGGGTTTAGGGTTAGGGTTTAGGGTTTAGGGTTTAGGGTTAGGGTTTAGGGTTTAGGGTTTAGGGTTTAGGGTTTAGGGTTTAGGGTTTAGGGTTTAGGGTTAGGGTTTAGGGTTTAGGGTTTAGGGTTAGGGTTTAGGGTTTAGGGTTTAGGGGTTTAGGGTTTAGGGTTTAGGGTTTAGGGTTTAGGGTTTAGGGTTTAGGGTTTAGGGTTTAGGGTTTAGGGTTTAGGGTTAGGGTTTAGGGTTTAGGGTTTAGGGTTTAGGGTTGGTTAGGGTTTAGGGTTTAGGGTTTAGGGTTTAGGGTTTAGGGTTTAGGGTTTAGGGTTTAGGGTTTAGGGTTTAGGGTTTAGGGTTTAGGGTTTAGGGTTTAGGTTAGGGTTTAGGGTTTAGGGTTTAGGGTTTTAGGGTTTAGGGTTTAGGGTTAGGGTTTAGGGTTTAGGGTTTAGGGTTTAGGGTTTAGGGTTTAGGGTTTAGGGTTAGGGTTTAGGGTTTAGGGTTTAGGGTTAGGGTTTAGGGTTTAGGGTTTAGGGTTAGGGTTTAGGGTTTAGGGTTTAGGGTTTAGGGTTTAGGGTTTAGGGTTTAGGGTTAGGGTTTAGGGTTTAGGGTTAGGGTTTAGGGTTTAGGGTTAGGGTTTAGGGTTTAGGGTTTAGGGTTTAGGGTTTAGGGTTTAGGGTTTAGGGTTTAGGGTTAGGTTAGGGTTTAGGGTTAGGGTTTAGGGTTTAGGGTTTAGGGTTTAGGGTTTAGGGTTTAGGGTTTAGGGTTTAGGGTTTAGGGTTTAGGGTTTAGGGTTTAGGGTTTAGGGTTTAGGGTTTAGGGTTTAGGGTTTAGGGTTTAGGGTTTAGGGTTTAGGGTTTAGGGTTTAGGGTTAGGGTTTAGGGTTTAGGGTTAGGGTTAGGGTTTAGGGTTTAGGGTTTAGGGTTTAGGGTTTAGGGTTTAGGGTTTAGGGTTTAGGGTTTAGGGTTTAGGGTTTAGGGTTTAGGGTTAGGGTTTAGGGTTTAGGGTTTAGGGGTTTAGGGTTTAGGGTTTAGGGTTTAGGGTTTAGGGTTTAGGGTTTAGGGTTTAGGGTTTAGGGTTTAGGGTTTAGGGTTTAGGGTTTAGGGTTAGGGTTTAGGGTTTAGGGTTTAGGGTTAGGGTTTAGGGTTAGGGTTTAGGGTTTAGGGTTGTAGGGTTTAGGGTTTAGGGGTTTAGGGTTTAGGGTTAGGGTTTAGGGTTTAGGGTTTAGGGTTTAGGGTTTAGGGTTTAGGGTTGGTTAGGGTTTAGGGTTTAGGGTTTAGGGTTTGGGTTTAGGGTTTAGGGTTTAGGGTTTAGGGTTTAGGGTTTAGGGGTTTAGGGTTTAGGGTTTAGGGTTTAGGGTTTAGGGTTTAGGGTTTAGGGTTTAGGGTTTAGGGTTGGGTTTAGGGTTTAGGGTTTAGGGTTAGGGTTTAGGGTTTAGGGTTTAGGGTTTAGGGTTTAGGGTTTAGGGTTTAGGGTTAGGGTTAGGGTTTAGGGTTTAGGGTTTAGGGTTTAGGGTTTAGGGTTTAGGGTTTAGGGTTGGGTTTAGGGTTTAGGGTTTAGGGTTTAGGGTTTAGGGTTTAGGGGTTTAGGGTTTAGGGTTTTAGGGTTTAGGGTTTAGGGTTTAGGGTTTAGGGTTTAGGGTTTAGGGTTTAGGGTTTAGGGTTTAGGGTTTAGGGTTTAGGGTTTAGGGTTTAGGGTTTAGGGTTTAGGGTTTAGGGTTAGGGTTTAGGGTTTAGGGTTTAGGGTTTAGGGTTTAGGGTTTAGGGTTTAGGGTTTAGGGGTTAGGGTTTAGGGTTTAGGGTTTAGGGTTTAGGGTTTAGGGTTTAGGGTTTAGGGTTTAGGGTTTAGGGTTTAGGGTTTAGGGTTTAGGGTTTAGGGTTTAGGGTTTAGGGTTTAGGGTTTAGGGTTTAGGGTTTAGGGTTTAGGGTTTAGGGTTTAGGGTTTAGGGTTTAGGGTTTAGGGTTTAGGGTTTAGGGTTTAGGGTTTAGGGTTTAGGGTTTAGGGTTTAGGGTTTAGGGTTTAGGGTTTAGGGTTAGGGTTTAGGGTTTAGGGTTTAGGGTTTAGGGTTTAGGGTTTAGGGTTTAGGGTTTAGGGTTTAGGGTTTAGGGTTAGGGTTTAGGGTTTAGGGTTTAGGGTTTAGGGTTTAGGGTTAGGGTTTAGGGTTTAGGGTTTAGGGTTTAGGGTTTAGGGTTTAGGGTTTAGGGTTTAGGGTTTAGGGTTTAGGGTTTAGGGTTTAGGGTTTAGGGTTTAGGGTTTAGGGTTTAGGGTTTAGGGTTTAGGGTTTAGGGTTTAGGGTTTAGGGTTTAGGGTTTAGGGTTTAGGGTTTAGGGTTTAGGGTTTAGGGTTTAGGGTTAGGGTTTAGGGTTTAGGGTTTAGGGTTAGGGTTTAGGGTTTAGGGTTTAGGGTTTAGGGTTTAGGGTTTAGGGTTTAGGGTTTAGGGTTTAGGGTTTAGGGTTTAGGGTTTAGGGTTTAGGGTTTAGGGTTAGGGTTTAGGGTTTAGGGTTTAGGGTTTAGGGTTTAGGGTTTAGGGTTTAGGGTTAGGGTTTAGGGTTTAGGGTTTAGGGTGGGTTTAGGGTTTAGGGTTTTAGGGTTTAGGGTTTAGGGTTTAGGGTTTAGGGTTAGGGTTTAGGGTTTAGGGTTTAGGGTTTAGGGTTTAGGGTTTAGGGTTTAGGGTTTAGGGTTAGGGTTTAGGGTTTAGGGTTTAGGTTTAGGGTTTAGGGTTTAGGGTTTAGGGTTTAGGGTTTAGGGTTTAGGGTTTAGGGTTTAGGGTTTAGGGTTTAGGGTTTAGGGTTTAGGGTTTAGGGTTTAGGGTTTAGGGTTTAGGGTTTAGGGTTTAGGGTTTAGGGTTTAGGGTTTAGGGTTAGGGTTTAGGGTTTAGGGTTTAGGGTTTAGGGTTTAGGGTTTAGGGTTTAGGGTTTAGGGTTTTAGGGGTTTAGGGTTTTGGGTTTAGGGTTTAGGGTTTAGGGTTTAGGGTTAGGGTTTAGGGTTTAGGGTTAGGGTTTAGGGTTTAGGGTTTAGGGTTAGGGTTTAGGGTTTGGGTTAGGGTTTAGGGTTTAGGGTTTAGGGTTTGGGTTAGGGTTTAGGGTTTAGGGTTTAGGGTTTAGGGTTTAGGGTTTAGGGTTAGGGTTTAGGGTTTAGGGTTTAGGGTTTAGGGTTTAGGGTTTAGGGTTTAGGGTTTAGGGTTTAGGGTTTAGGGTTTAGGGTTTAGGGTTTAGGGTTTAGGGTTTAGGGTTTAGGGTTTAGGGTTTAGGGTTAGGGTTTAGGGTTTAGGGTTTAGGGTTTAGGGTTTAGGGTTTAGGGTTTAGGGTTTAGGGTTTAGGGTTTAGGGTTTAGGGTTTAGGGGTTTAGGGTTTAGGGTTTAGGGTTTAGGGTTTAGGGTTTAGGGTTAGGGTTTAGGGTTTAGGGTTTTAGGGTTTAGGGTTTAGGGTTTAGGGTTTAGGGTTTAGGGTTTAGGGTTTAGGGTTTAGGGTTTAGGGTTTAGGGTTTAGGGTTTAGGGTTTAGGGTTTAGGGTTTAGGGTTTAGGGTTTAGGGTTTAGGGTTTAGGGTTTAGGGTTTAGGGTTTAGGGTTAGGGTTTAGGGTTTAGGGTTTAGGGTTTAGGGTTTAGGGTTTAGGGTTTAGGGTTTAGGGTTTAGGGTTTAGGGTTTAGGGTTTAGGGTTTAGGGTTTAGGGTTAGGGTTTAGGGTTTAGGGTTTAGGGTTTAGGGTTTAGGGTTTAGGGTTTAGGGTTTAGGGTTTAGGGTTTAGGGTTTAGGGTTTAGGGTTTAGGGGTTAGGGTTTAGGGTTTAGGGTTTAGGGTTTAGGGTTTAGGGTTTAGGGTTTAGGGTTTAGGGTTTAGGGTTTAGGGTTTAGGGTTTAGGGTTTAGGGTTTAGGGTTTAGGGTTTAGGGTTTAGGGTTTAGGGTTTAGGGTTTAGGGTTTAGGGTTTAGGGTTTAGGGTTTAGGGTTTAGGGTTTAGGGTTTAGGGTTTAGGGTTTAGGGTTTAGGGTTTAGGGTTTAGGGTTTAGGGTTTAGGGTTTAGGGTTTAGGGTTTAGGGTTTAGGGTTTAGGGTTAGGGTTTAGGGTTTAGGGTTTAGGGTTTAGGGTTTAGGGTTTAGGGTTTAGGGTTTAGGGTTTA

**4AS**

GGG**TTTAGGG**TTTAGGGTAAGGGTTTGGGTTTAGGGTTTATGTTTTAGGGTTTAGGGTTTAGGGTTTAGGGTTTAGGGTTTAGGGTTTAGGGTTTAGGTTTAGGGTTTAGGGTTTAGGGTTTAGGGTTAGGGTTTAGGGTTAGGGTTAGGGTTTAGGGTTTAGGGTTTAGGGTTTAGGGTTAGGGTTTGGTTTAGGGTTTTAGGGTTTTAGGGTTTTAGGGTTAGGGTTTAGGGTTTAGGGTTTAGGGTTAGGGTTAGGGTTTAGGGTTTAGGGTTTAGGGTTTAGGGTTAGGGTTTAGGGTTAGGGTTTAGGGTTTAGGGTTTAGGGTTTAGGGTTAGGGTTTAGGGTTAGGGTTAGGGTTTAGGGTTTAGGGTTAGGGTTAGGGTTAGGGTTTAGGGTTAGGGTTTAGGGTTTAGGGTTAGGGTTAGGGTTTAGGGTTTAGGGTTTAGGGTTTAGGGTTTAGGGTTTAGGGTTTAGGGTTTAGGGTTTAGGGTTTAGGGTTTAGGGTTTAGGGTTTAGGGTTTAGGGTTTAGGGTTTAGGGTTTAGGGTTTAGGGTTAGGGTTTAGGGTTTAGGGTTTAGGGTTAGGGTTTAGGGTTTAGGGTTTAGGGTTTAGGGTTTAGGGTTTAGGGTTAGAGGGTTAGGGTTAGGGTTAGGGTTAGGGTTTAGGGTTAGGGTTTAGGGTTTAGGGTTTAGGGTTTAGGGTTTAGGGTTAGGGTTTAGGGTTTAGGGTTTAGGGTTTAGGGTTTAGGGTTTAGGGTTTAGGGTTTAGGGTTTAGGGTTTAGGGTTTAGGGTTTAGGGTTTAGGGTTTAGGGTTTAGGGTTTAGGGTTTAGGGTTTAGGGTTTAGGGTTTAGGGTTTAGGGTTTAGGGTTAGGGTTTAGGGTTTAGGGTTTAGGGTTTAGGGTTTAGGGTTAGGGTTTAGGGTTTAGGGTTTAGGGTTTAGGGTTAGGGTTTAGGGTTTAGGGTTTAGGGTTTAGGGTTTAAGGGTTTAGGGTTTAGGGTTTAGGGTTTAGGGTTTAGGGTTTAGGGTTAGGGTTTAGGGTTTAGGGTTTAGGGTTTAGGGTTAGGGTTTAGGGTTTAGGGTTTAGGGTTTAGGGTTAGGGTTTAGGGTTTAGGGTTTAGGGTTTAGGGTTTAAGGGTTTAGGGTTTAGGGTTTAGGGTTTAGGGTTTAGGGTTTAGGGTTTAGGGTTTAGGGTTTAGGGTTTAGGGTTTAGGGTTAGGGTTTAGGGTTTTTAGGGTTTAGGGTTTAGGGTTAGGGTTAGGGTTAGGGTTTAGGGTTTAGGGTTTAGGGTTTAGGGTTTAGGGTTTAGGGTTTAGGGTTTAGGGTTTAGGGTTTAGGGTTTAGGGTTTAGGGTTTAGGGTTTAGGGTTTAGGGTTTAGGGTTTAGGGTTTAGGGTTTAGGGTTTAGGGTTTAGGGTTTAGGGTTTAGGGTTTAGGGTTTAGGGTTTAGGGTTTAGGGTTTAGGGTTTAGGGTTTAGGGTTTAGGGTTTAGGGTTTAGGGTTTAGGGTTAGGGTTTAGGGTTTAGGGTTTAGGGTTTAGGGTTTAGGGTTTAGGGTTTAGGGTTTAGGGTTTAGGGTTTAGGGTTTAGGGTTTAGGGTTTAGGGTTTAGGGTTTAGGGTTTAGGGTTTAGGGTTTAGGGTTTAGGGTTTAGGGTTTAGGGTTTAGGGTTTAGGGTTTAGGGTTTAGGGTTTAGGGTTTAGGGTTTAGGGTTTAGGGTTTAGGGTTTAGGGTTTAGGGTTTAGGGTTTAGGGTTTAGGGTTTAGGGTTTAGGGTTTAGGGTTTAGGGTTTAGGGTTTAGGGTTTAGGGTTTAGGGTTTAGGGTTTAGGGTTTAGGGTTTAGGGTTTAGGGTTTAGGGTTTAGGGTTTAGGGTTTAGGGTTTAGGGTTTAGGGTTTAGGGTTTAGGGTTTAGGGTTTAGGGTTTAGGGTTTAGGGTTTAGGGTTTAGGGTTTAGGGTTTAGGGTTTAGGGTTTAGGGTTTAGGGTTTAGGGTTTAGGGTTTAGGGTTTAGGGTTTAGGGTTTAGGGTTTAGGGTTTAGGGTTTAGGGTTTAGGGTTTAGGGTTTAGGGTTTAGGGTTTAGGGTTTAGGGTTTAGGGTTTAGGGTTTAGGGTTTAGGGTTTAGGGTTTAGGGTTTAGGGTTTAGGGTTTAGGGTTTAGGGTTTAGGGTTTAGGGTTTAGGGTTTAGGGTTTAGGGTTTAGGGTTTAGGGTTTAGGGTTTAGGGTTTAGGGTTTAGGGTTTAGGGTTTAGGGTTTAGGGTTTAGGGTTTAGGGTTTAGGGTTTAGGGTTTAGGGTTTAGGGTTTAGGGTTTAGGGTTTAGGGTTTAGGGTTTAGGGTTTAGGGTTTAGGGTTTAGGGTTTAGGGTTTAGGGTTTAGGGTTTAGGGTTTAGGGTTTAGGGTTTAGGGTTTAGGGTTTAGGGTTTAGGGTTTAGGGTTAGGGTTTAGGGTTTAGGGTTTAGGGTTTAGGGTTTAGGGTTTAGGGTTTAGGGTTTAGGGTTTAGGGTTTAGGGTTTAGGGTTTAGGGTTTAGGGTTTAGGGTTTAGGGTTTAGGGTTTAGGGTTTAGGGTTTAGGGTTTAGGGTTTAGGGTTTAGGGTTTAGGGTTTAGGGTTTAGGGTTTAGGGTTTAGGGTTTAGGGTTTAGGGTTTAGGGTTTAGGGTTAGGGTTTAGGGTTAGGGTTTAGGGTTTAGGGTTTAGGGTTTAGGGTTTAGGGTTTAGGGTTTAGGGTTTAGGGTTTAGGGTTTAGGGTTTAGGGTTAGGGTTTAGGGTTAGGGTTTAGGGTTTAGGGTTTAGGGTTTAGGGTTTAGGGTTTAGGGTTAGGGTTTAGGGTTTAGGGTTTAGGGTTTAGGGTTTAGGGTTTAGGGTTTAGGGTTTAGGGTTTAGGGTTTAGGGTTTAGGGTTTAGGGTTTAGGGTTTAGGGTTTAGGGTTTAGGGTTTAGGGTTTAGGGTTTAGGGTTTAGGGTTTAGGGTTTAGGGTTTAGGGTTTAGGGTTTAGGGTTTAGGGTTTAGGGTTTAGGGTTTAGGGTTTAGGGTTTAGGGTTTAGGGTTTAGGGTTTAGGGTTTAGGGTTAGGGTTTAGGGTTTAGGGTTAGGGTTTAGGGTTTAGGGTTTAGGGTTTAGGGTTTAGGGTTTAGGGTTTAGGGTTTAGGGTTTAGGGTTTAGGGTTTAGGGTTTTTTAGGGTTTAGGGTTTAGGGTTTAGGGTTTAGGGTTTAGGGTTTAGGGTTTAGGGTTAGGGTTTAGGGTTTAGGGTTTAGGGTTTAGGGTTTAGGGTTTAGGGGTTTAGGGTTTAGGGTTTAGGGTTTAGGGTTTAGGGTTAGGGTTTAGGGTTTAGGGTTTAGGGTTTAGGGTTTAGGGTTTAGGGTTTAGGGTTTAGGGTTTAGGGTTTAGGGTTTAGGGTTTAGGGTTTAGGGTTTAGGGTTTAGGGTTTAGGGTTTAGGGTTTAGGGTTTAGGGTTTAGGGTTTAGGGTTTAGGGTTTAGGGTTTAGGGTTTAGGGTTTAGGGTTTAGGGTTTAGGGTTAGGGTTTAGGGTTTAGGGTTTAGGGTTTAGGGTTTAGGGTTTAGGGTTTAGGGTTTAGGGTTTAGGGTTTAGGGTTTAGGGTTTAGGGTTTAGGGTTAGGGTTTAGGGTTTAGGGTTTAGGGTTTAGGGTTTAGGGTTTAGGGTTAGGGTTTAGGGTTTAGGGTTTAGGGTTTAGGGTTAGGGTTTAGGGTTTAGGGTTTAGGGTTAGGGTTTAGGGTTTAGGGTTTAGGGTTTAGGGTTTAGGGTTTAGGGTTTAGGGTTTAGGGGTTTAGGGTTTAGGGTTTAGGGTTTAGGGTTTAGGGTTTAGGGTTTAGGGTTTAGGGTTTAGGGTTTAGGGTTTAGGGTTTAGGGTTAGGGTTAGGGTTTAGGGTTTAGGGTTAGGGTTTAGGGTTTAGGGTTTAGGGTTTAGGGTTTAGGGTTTAGGGTTTAGGGTTTAGGGTTTAGGGTTAGGGTTTAGGGTTTAGGGTTTAGGGTTTAGGGTTTAGGGTTTAGGGTTAGGGTTTAGGGTTTAGGGTTTAGGGTTTAGGGTTTAGGGTTTAGGGTTTAGGGTTTAGGGTTTAGGGTTTAGGGTTTAGGGTTTAGGGTTTAGGGTTTAGGGTTTTTTGGTTTAGGGTTTAGGGTTAGGGTTTAGGGTTAGGGTTAGGGTTTAGGGTTTAGGGTTAGGGTGGTTAGGGTTTAGGGTTTAGGGTTTAGGGTTTAGGGTTTTAGGGTTTAGGGTTTAGGGTTTAGGGTTTAGGGTTTAGGGTTTAGGGTTTAGGGTTTAGGGTTTAGGGTTTAGGGTTTAGGGTTTAGGGTTTAGGGTTTAGGGTTTAGGGTTTAGGGTTTAGGGTTTAGGGTTTAGGGTTTAGGGTTTGGGGTTAGGGTTTAGGGTTTAGGGTTTAGGGTTTAGGGTTTAGGGTTTAGGGTTTAGGGTTTAGGGTTTAGGGTTTAGGGTTAGGGTTTAGGGTTTAGGGTTTAGGGTTTAGGGTTTAGGGTTTAGGGTTTAGGGGTTTAGGGTTTAGGGTTTAGGGTTTAGGGTTTAGGGTTTAGGGTTTAGGGTTTAGGGTTAGGGTTTAGGGTTTAGGGTTTAGGGTTAGGGTTTAGGGTTTAGGGTTTAGGGTTAGGGTTTAGGGTTTAGGGTTTAGGGTTTAGGGTTTAGGGTTTAGGGTTTAGGGTTTAGGGTTAGGGTTTAGGTTTAGGGTTTAGGGTTTAGGGTTTAGGGTTAGGGTTAGGGTTTAGGGTTTAGGGTTTAGGGTTTAGGGTTTAGGGTTAGGGTTTAGGGTTTAGGGTTTTAGGGTTTAGGGTTTAGGGTTTAGGGTTTAGGGTTTAGGGTTTAGGGTTTAGGGTTTAGGGTTTAGGGTTAGGGTTTAGGGTTTAGGGTTTAGGGTTTAGGGTTTAGGGTTTAGGGTTTAGGGTTTAGGGTTTAGGGTTTAGGGTTTAGGGTTTAGGGTTTAGGGTTTAGGGTTTA

**5AL**

TTTGG**TTTAGGG**TTTAGGGTTTAGGCTTTAGGTTTAGGGTTTAGGTTTAGGGTTTAGGCTTTAGGTTTAGGGTTTAGGTTTAGGGTTTAGGGTTTAGGGTTTAGGGTTTAGGGTTTAGGGTTTAGGGTTTAGGGTTTAGGGTTTAGGGTTTAGGGTTTAGGGTTTAGGGTTTAGGGTTTAGGGTTTAGGGTTTAGGGTTTAGGGTTTAGGGTTTAGGGTTTAGGGTTAGGGTTTAGGGTTAGGGTTAGGGTTTAGGGTTTAGGGTTTAGGGTTTTAGGGTTTAGGGTTTAGGGTTAGGGTTTAGGGTTTAGGGTTTAGGGTTAGGGTTAGGGTTTAGGGTTTAGGGTTTAGGGTTTAGGGTTTAGGGTTTAGGGTTTAGGGTTTAGGGTTTAGGGTTTAGGGTTTAGGGTTAGGGTTTAGGGTTTAGGGTTTAGGGTTTAGGGTTTAGGGTTTAGGGTTTAGGGTTTAGGGTTTAGGGTTTAGGGTTTAGGGTTTAGGGTTAGGGTTTAGGGTTTAGGGTTAGGGTTTAGGGTTTAGGGTTTAGGGTTAGGGTTTAGGGTTTAGGGTTTAGGGTTTAGGGTTAGGGTTTAGGGTTTAGGGTTTAGGGTTTAGGGTTTAGGGTTAGGGTTTAGGGTTTAGGGTTAGGGTTTAGGGTTTAGGGTTAGGGTTTAGGGTTAAGGGTTTAGGGTTTAGGGTTTAGGGTTTAGGGTTTAGGGTTTAGGGTTTAGGGTTTAGGGTTTAGGGTTTAGGGTTAGGGTTTAGGGTTTAGGGTTTAGGGTTAGGGTTTAGGGTTTAGGGTTAGGGTTTAGGGTTAGGGTTTAGGGTTTAGGGTTTAGGGTTTAGGGTTAGGGTTTAGGGTTTAGGGTTTAGGGTTTAGGGTTTAGGGTTTAGGGTTTAGGGTTAGGGTTTAGGGTTTAGGGTTTAGGGTTAGGGTTTAGGGTTTAGGGTTTAGGGTTTAGGGTTAGGGTTTAGGGTTTAGGGTTTAGGGTTTAGGGTTTAGGGTTTAGGGGTTTAGGGTTTAGGGTTTAGGGTTTAGGGTTTAGGGTTTAGGGTTTAGGGTTTAGGGTTTAGGGTTTAGGGTTAGGGTTTAGGGTTTAGGGTTTTAGGGTTTAGGGTTTAGGGTTTAGGGTTTAGGGTTTTGGGGTTTAGGGTTTAGGGTTTAGGGTTTAGGGTTTAGGGTTTAGGGTTTAGGGTTTAGGGTTTAGGGTTTAGGGTTTAGGGTTTAGGGTTTAGGGTTTAGGGTTAGGGTTTAGGGTTTAGGGTTAGGGTTTAGGGTTTAGGGTTTAGGGTTAGGGTTTAGGGTTTAGGGTTAAGGGTTTAGGGTTAGGGTTAGGGTTTAGGGTTTAGGGTTTAGGGTTAGGGTTTAGGGTTTAGGGTTAGGGTTTAGGGTTTAGGGTTTAGGGTTTAGGGTTTAGGGTTAGGGTTAGGGTTTAGGGTTTAGGGTTTGGGGTTTAGGGTTTAGGGTTAGGGTTAGGGTTTAGGGTTTAGGGTTTAGGGTTCAGGGTTTAGGGTTTAGGGTTAGGGTTTAGGGTTAGGGTTAGGGTTAGGGTTTAGGGTTAGGGTTTAGGGTTTAGGGTTTAGGGTTTAGGGTTTAGGGTTTAGGGTTTAGGGTTTAGGGTTTAGGGTTAGGGTTTAGGGTTTAGGGTTAGGGTTTAGGGTTTAGGGTTTAGGGTTTAGGGTTTAGGGTTTAGGGTTTAGGGTTAGGGGTTTAGGGTTTAGGGTTTAGGTTTAGGGTTTAGGGTTTAGGGTTTAGGGTTTAGGGTTTAGGGTTTAGGGTTTAGGGTTAGGGTTAGGGTTTAGGGTTTAGGGTTTAGGGTTTAGGGTTTAGGGTTTAGGGTTTAGGGTTAGGGTTTAGGGTTTAGGGTTAGGGTTTAGGGTTAGGGTTTAGGGTTAGGGTTAGGGTTTAGGGTTTAGGGTTTAGGGTTTAGGGTTTAGGGTTTAGGGTTTAGGGTTTAGGGTTAGGGTTTAGGGTTTAGGGTTAGGGTTTAGGGTTTAGGGTTAGGGTTTAGGGTTAGGGGTTAGGGTTTAGGGTTTAGGGTTTAGGGTTTAGGGTTTAGGGTTTAGGGTTTAGGGTTTTTAGGGTTTAGGGTTTAGGGTTTAGGGTTTAGGGTTTAGGGTTAGGGTTTAGGGTTTAGGGTTTAGGGTTTAGGGTTTAGGGTTTAGGGTTAGGGTTAGGGTTTAGGGTTTAGGGGTTAGGGTTTAGGGTTAGGGTTTAGGGTTTAGGGTTTAGGGTTTAGGGTTTAGGGTTTAGGGTTTAGGGTTTAGGGTTTAGGGTTTAGGGTTTAGGGTTTAGGGTTTAGGGTTTAGGGTTTTTAGGGTTTTAGGGTTAGGGTTTAGGGTTTAGGGTTTAGGGTTTAGGGTTTAGGGTTAGGGTTTAGGGTTTAGGGTTTAGGGTTTAGGGTTTAGGGTTTAGGGTTTAGGGTTTAGGGTTTAGGGTTAGGGTTTAGGGTTTAGGGTTTAGGGTTTGGGGTTTAGGGTTTAGGGTTTAGGGTTTAGGGTTAGGGTTTAGGGTTTAGGGTTTAGGGTTTAGGGTTTAGGGTTTAGGGTTTAGGGTTTAGGGTTTAGGGTTTAGGGTTTAGGGTTTAGGGTTTAGGGTTTAGGGTTTAGGGTTTAGGGTTTAGGGTTTAGGGTTTAGGGTTTAGGGTTTAGGGTTTAGGGTTTAGGGTTTAGGGTTTAGGGTTAGGGTTTAGGGTTTAGGGTTTAGGGTTTAGGGTTTAGGGTTTAGGGTTTAGGGTTTAGGGTTTAGGGTTTAGGGTTTAGGGTTTAGGGTTTAGGGTTAGGGTTAGGGTTTAGGGTTTAGGGTTTAGGTTTAGGGTTTAGGGTTTAGGGTTTAGGGTTTAGGGTTTAGGGTTTAGGGTTTAGGGTTTAGGGTTTAGGGTTTAGGGTTTAGGGTTAGGGTTTAGGGTTTAGGGTTTAGGGTTAGGGTTTAGGGTTTAGGGTTTAGGGTTTAGGGTTTAGGGTTTAGGGTTAGGGTTTAGGGTTTAGGGTTTAGGGTTTAGGGTTTAGGGTTTTGGGTTTAGGGTTTAGGGTTTAGGGTTTAGGGTTTAGGGTTTAGGGTTTAGGGTTTAGGGTTTAGGGTTTAGGGTTTAGGGTTTTTAGGGTTTAGGGTTGTTAGGGTTTAGGGTTTAGGGTTTAGGGTTTAGGGTTAGGGTTTAGGGTTTAGGGTTTAGGGTTTAGGGTTTAGGGTTTAGGGTTTTAGGGTTTTTAGGGTTTTAGGGTTTTAGGGTTTTAGGGTTTTTAGGGTTTAGGGTTTAGGGTTTAGGGTTTAGGGTTTAGGGTTTAGGGTTTAGGGTTTAGGGTTTAGGGTTTAGGGTTTAGGGTTTAGGGTTTAGGGTTTAGGGTTTAGGGTTTAGGGTTTAGGGTTTAGGGTTTAGGGTTTAGGGTTTAGGGTTTAGGGTTTAGGGTTTAGGGTTTAGGGTTTAGGGTTTAGGGTTTAGGGTTTAGGGTTTAGGGTTTAGGGTTAGGGTTTAGGGTTTTAGGGTTAGGGTTTAGGGTTTAGGGTTTAGGGTTTAAGGGTTTAGGGTTTAGGGTTTAGGGTTTAGGGTTTAGGGTTTAGGGTTTAGGGTTTTAGGGTTTAGGGTTTAGGGTTTAGGGTTTAGGGTTAGGGTTTAGGGTTTAGGGTTTAGGGTTTAGGGTTTAGGGTTGGTTTAGGGTTTAGGGTTTAGGGTTTAGGTTTAGGGTTTAGGGTTTAGGGTTTAGGGTTTAGGGTTTAGGGTTTAGGGTTTAGGGTTTAGGGTTTTAGGGTTTAGGGTTAGGGTTTAGGGTTTAGGGTTTAGGGTTTAGGGTTTAGGGTTTAGGGTTTAGGGTTAGGGTTAGGGTTTAGGGTTTAGGGTTTAGGGTTTAGGGTTTAGGGTTTAGGGTTTAGGGTTTAGGGTTTAGGGTTTAGGGTTTAGGGTTTAGGGTTTAGGGTAGGTTTAGGGTTTAGGGTTTAGGGTTTAGGGTTTAGGGTTTAGAGGTTTTAGGGTTTAGGGTTTAGGGTTTAGGGTTAGGGTTTAGGGTTTAGGGTTTTTAGGGTTTAGGGTTTAGGGTTTGGGGTTAGGGTTTAGGGTTTAGGGTTTAGGGTTTAGGGTTTAGGGTTTAGGGTTTAGGGTTTAGGGTTTAGGGTTTAGGGTTAGGGTTTAGGGTTTAGGGTTTAGGGTTTAGGGTTTAGGGTTTAGGGTTTAGGGTTTTAGGGTTTAGGGTTAGGGTTTAGGGTTTAGGGTTTAGGGTTTAGGGTTTAGGGTTTAGGGTTTAGGGTTAGGTTTAGGGTTTAGGGTTTAGGGTTTAGGGTTTAGGGTTTAGGGTTAGGGTTTAGGGTTTAGGGTTTAGGGTTTAGGGTTTAGGGTTAGGGTTTAGGGTTTAGGGTTTAGGGTTTTTAGGGTTTTAGGGTTTAGGGTTTAGGGTTTAGGGTTTAGGTTTAGGGTTTAGGGTTTAGGGTTTAGGGTTTAGGGTTTAGGGTTTAGGTTAGGGTTTAGGGGTTTAGGTTTGGTTAGGGTTTAGGGTTTAGGGTTTAGGGTTTAGGGTTTAGGGTTTAGGGTTTAGGGTTTAGGGTTTAGGGTTTAGGGTTTAGGGTTAGGGTTTAGGGTTTTAGGGTTGGGTTTAGGGTTTAGGGTTAGGGTTTAGGGTTTAGGGTTTAGGGTTTAGGGTTAGGGTTTTTGGTTTAGGGTTAGGGTTTAGGGTTTAGGGTTTAGGGTTTAGGGTTTAGGGTTGTAGGGTTTAGGGTTTAGGGTTTAGGGGTTTAGGGTTTAGGGTTTAGGGTTTAGGGTTTAGGGTTAGGGTTAGGGTTTTTAGGGTTTAGGGTTTAGGGTTTAGGGTTTAGGGTTTAGGGTTTAGGGTTTAGGGTTTAGGGTTAGGGTTTAGGGTTTAGGGTTAGGGTTTAGGGTTTAGGGTTTAGGGTTTAGGGTTTAGGGTTTAGGGTTTAGGTTTAGGGTTTGGGTTTAGGGTTTAGGGTTTAGGGTTTAGGGTTTAGGGTTTAGGTTTAGGGTTTAGGGTTTAGGGTTTAGGGTTAGGGTTTAGGGTTTAGGGTTTAGGGTTAGGGTTTAGGGTTTAGGGTTAGGGTTTAGGGTTAGGGTTTAGGGTTAGGGTTTAGGGTTTAGGGTTTAGGGTTTAGGGTTTAGGGTTTAGGGTTTAGGGTTTAGGGTTTAGGGTTTAGGGTTTAGGGTTTAGGGTTTAGGGTTTAGGGTTTAGGGTTTAGGGTTTAGGGTTTAGGGTTTAGGGTTTAGGGTTTAGGGTTTAGGATTAGGGTTTAGGGTTTAGGGTTAGGGTTTAGGGTTTAGGGTTTAGGGTTTAGGGTTTAGGGTTTAGGGTTTAGGTTTAGGGTTTAGGGTTTAGGGTTTAGGGTTTAGGGTTTAGGGGTTAGGGTTTAGGGTTTAGGGTTAGGGTTTAGGTTTAGGGTTTAGGGTTTAGGGTTTAGGGTTTAGGGTTTAGGGTTTAGGGTTTAGGGTTTAGGGTTTAGGGTTTAGGGTTTAGGGTTTAGGGTTTAGGGTTTAGGGTTTAGGGTTAGGGTTAGGTTTAGGGTTTAGGGTTTAGGGTTTAGGGTTTAGGGTTTAGGGTTTAGGGTTTAGGGTTTAGGGTTTAGGGTTTAGGGTTTAGGGTTTAGGGTTTAGGGGTTTAGGGTTAGGGTTTATGTTTAGGGTTTAGGGTTTAGGGTTTAGGGTTTAGGTTTAGGGTTTAGGGTTTATGGGTTTAGGGTTTAGGGTTTAGGGTTTAGGGTTTAGGGTTAGGGTTTAGGGTTTAGGGTTTAGGGTTTAGGGTTTAGGGTTTAGGGTTTAGGGTTTAGGGTTTAGGGTTTAGGGTTTAGGGGTTTAGGGTTTAGGGTTAGGGTTTAGGGTTAGGGGTTTAGGGTTTAGGGTTAGGGTTTAGGGTTTAGGGTTTAGGGTTTAGGGTTTAGGGTTTAGGGTTTAGGGTTTAGGGTTTAGGGTTAGGGTTTGTTAGGGTTTAGGGTTTAGGGTTTAGGGTTTTAGGGTTTTAGGGTTTAGGGTTTTTAGGGTTTAGGGTTTAGGGTTTAGGGTTTAGGGTTAGGGTTTAGGGTTTAGGGTTTAGGGTTTTAGGGTTTAGGGTTTAGGGTTTAGGGTTTAGGGTTTAGGGTTTAGGGTTTAGGGTTAGGGTTTAGGGTTTAGGGTTTAGGGTTTAGGGTTTAGGGTTTAGGGTTTAGGGTTAGGGTTTAGGGTTTTAGGGTTTAGGGTTAGGGTTTAGGGTTTAGGGTTAGGGTTTAGGGTTTAGGGTTTAGGGTTTAGGGTTAGGGTTTAGGGTTTAGGGTTTAGGGTTTAGGGTTTAGGGTTTAGGGTTTAGGGTTTAGGGTTTAGGGTTTAGGGTTTAGGGTTTAGGGTTTAGGGTTTAGGGTTTAGGGTTAGGGTTTAGGGTTTAGGGTTTAGGGTTAGGGTTTAGGGTTAGGGTTTAGGGTTTAGGGTTTAGGGTTTTAGGGTTTAGGGTTTAGGGTTTAGGGTTTAGGGTTTAGGGTTTAGGGTTTAGGGTTTAGGGTTTAGGGTTTAGGGTTTAGGGTTTAGGGTTTAGGGTTTAGGGTTTAGGGTTTAGGGTTTAGGGTTTAGGGTTTAGGGTTTAGGGTTTAGGGTTTAGGGTTTAGGGTTTTTGGGTTTTAGGGTTTTAGGGTTTTTAGGGTTTAGGGTTTAGGGTTTAGGGTTTAGGGTTAGGGTTTAGGGTTTAGGGTTTAGGGTTTAGGGTTTAGGGTTTAGGGTTTAGGGTTTAGGGTTTAGGGTTTAGGGTTAGGGAGTTTAGGGTTTAGGGTTTAGGGTTTAGGGTTTAGGGTTAGGGTTTAGGGTTTAGGGTTTAGGGTTTAGGGGTTTTAGGTTTTAGGGTTTAGGGTTTAGGGTTTAGGGTTAGGGTTTAGGGTTTAGGGTTTAGGGTTTAGGGTTTAGGGTTTAGGGTTTAGGGTTAGGGTTAGGGTTTAGGGTTTAGGGTTTAGGGTTAGGGTTTTAGGGTTTAGGGTTTAGGGTTTAGGGTTTAGGGTTTAGGGTTGTTTAGGGTTTAGGGTTTAGTGTTTAGGGTTTAGGGTTTAGGGTTAGGGTTAGGGTTTAGGGTTTTTAGGGTTTAGGGTTTAGGGTTTAGGGTTAGGGTTTAGGGTTAGGTTAGGGTTAGGGTTAGGGTTTAGGGTTTAGGGTTTAGGGTTTAGGGTTTAGGGTTTAGGGTTTAGGGTTTAGGGTTTAGGGTTTAGGGTTTAGGGTTAGGGTTTAGGGTTTAGGGTTTAGGGTTTAGGGTTTAGGGTTTAGGGTTTAGGGTTTTAGGGTTTAGGGTTAGGGTTTAGGGTTTAGGGTTTAGGGTTTAGGGTTTTAGGTTTAGGGTTTAGGGTTTAGGGTTTAGGGTTTAGGGTTTAGGGTTTAGGGTTTAGGGTTTAGGGTTTAGGGTTTAGGGTTAGGGTTTGGGGTTAGGGTTTAGGGTTTAGGGTTTAGGGTTTAGGGTTTAGGGTTAGGGTTTAGGGTTTAGGGTTAGGGTTTAGGGTTTAGGGTTTAGGGTTAGGGTTTAGGGTTTAGGGTTTAGGGTTTAGGTTTAGGGTTTAGGGTTTAGGGTTTAGGGTTTAGGGTTTAGGGTAGGGTTTAGGGTTTAGGGTTTAGGGTTTAGGGTTTAGGGTTTAGGGTTTAGGGTTTAGGGTTTAGGGTTTAGGGTTAGGGTTTAGGGTTTAGGGTTTAGGGTTTAGGGTTTAGGGTTTAGGGTTTAGGGTTTAGGGTTTTAGGGTTTAGGGTTTAGGGGTTAGGGTTTAGGGGTTTAGGGTTTAGGGTTTAGGGTTTAGGGTTTAGGGTTTAGGGTTTAGGGTTAGGGTTTAGGGTTTAGGGTTTAGGGTTTAGGGTTTAGGGTTTAGGGTTTAGGGTTTAGGGTTAGGGTTTAGGGTTTAGGGTTTAGGGTTTAGGGTTTAGGGTTTAGGGTTTAGGGTTTAGGGTTTAGGGTTTAGGGTTTAGGGTTTAGGGTTTAGGGTTTAGGGTTTAGGGTTTAGGGTTTAGGGTTTAGGGTTAGGGTTTAGGGTTTAGGGTTTAGGGTTAGGGTTTAGGGTTTAGGGTTTAGGGTTTAGGTTTAGGGTTTAGGGTTTAGGGTTTAGGGTTTAGGGTTTAGGGTTTAGGGTTTAGGGTTTAGGGTTTAGGGTTTAGGGTTTAGGGTTTAGGGTTAGGGTTTTAGGGTTTAGGGTTTAGGGTTTAGGGTTAGGGTTTAGGGTTTAGGGTTTTAGGGTTTAGGGTTTAGGGTTTAGGGTTTAGGGTTTAGGGTTTAGGGTTTAGGGTTTAGGGTTTAGGGTTTAGGGTTTAGGGTTTAGGGTTTTAGGGTTTAGGGTTAGGGTTTAGGGTTTAGGGTTTAGGGTTTAGGGTTTAGGGTTTAGGGTTAGGGTTTAGGGTTAGGGTTTAGGGTTTAGGGTTTAGGGTTTAGGGTTTAGGGTTTAGGGTTTAGGGTTTAGGGTTAGGGTTTAGGGTTTTAGGGTTTAGGGTTAGGGTTTAGGGTTTAGGGTTTAGGGTTTTAGGGTTTAGGGTTTAGGGTTTAGGGTTTAGGGTTTAGGGTTTAGGGTTTAGGGTTTAGGGTTTAGGGTTTTAGGGTTTAGGGTTTAGGGTTTAGGGTTTAGGGTTTAGGGTTTAGGGTTTAGGGTTTAGGGTTGTTTAGGGTTTAGGGTTTAGGGTTTAGGGTTTAGGGTTTAGGGTTTAGGGTTTAGGGTTTAGGGTTTAGGGTTTAGGGTTAGGGTTTAGGGTTTAGGGTTGTTCAGGGTTTAGGGTTTAGGGGTTTAGGGTTTAGGGTTTAGGGTTAGGGTTAGGGTTTAGGGTTTAAGGGTTTAGGGTTTAGGGTTTAGGGTTTAGGGTTTAGGGTTTAGGGTTTAGGGTTTAGGGTTTAGGGTTTAGGGTTTAGGGGGTTTAGGGTTTAGGGTTTAGGGTTTAGGGTTTAGGGTTTAGGGTTAGGGTTTAGGGTTAGGGTTTAGGGTTAGGGTTTAGGGTTTAGGGTTTAGGGTTTAGGGTTTAGGGTTTAGGGTTTAGGGTTTAGGGTTTAGGGTTAGGGTTTAGGGTTTAGGGTTAGGGTTAGGGTTTAGAGTTAGGGTTTAGGGTTTTAGGGTTTAGGGTTTAGGGTTTAGGGTTAGGGTTTAGGGTTTAGGGTTTAGGGTTTAGGGTTTAGGGTTTAGGGTTTAGGGTTTAGGGTTTAGGGTTTAGGGTTTAGGGTTTAGGGTTTAGGGTTTAGGGTTTGGGTTTAGGGTTTAGGGGTTAGGGTTTAGGGTTTAGGGTTTAGGGTTTAGGGTTTAGGGTTTAGGGTTTAGGGTTTAGGGTTTAGGGTTAGGGTTAGGGTTTAGGGTTTAGGGGTTAGGGTTTAGGGTTTAGGGTTTAGGGTTTAGGGTTTAGGGTTTAGGGTTTAGGGTTTAGGGTTTAGGGTTAGGGTTTAGGGTTTAGGGTTTAGGGTTTAGGGTTTAGGGTTTAGGGTTTAGGGTTAGGGTTTAGGGGTTTAGGGTTTAGGGTTAGGGTTTAGGGTTTTAGGGTTTAGGGTTTTAGGGTTTTAGGGGTTTTAGGGTTAGGGTTTAGGGTTTAGGGTTTAGGGTTTAGGGTTTAGGGTTTAGGGTTTAGGGTTTAGGGTTTAGGGTTTAGGGTTAGTGGTTTAGGGTTTAGGGTTTAGGGTTTAGGGTTTAGGGTTTAGGGTTTAGGGTTTAGGGTTTAGGGTTTAGGGTTAGGGTTTAGGGTTTAGGGTTAGGGTTTAGGGTTTAGGGTTTAGGGTTTAGGGTTAGGGTTTAGGGTTTAGGGTTTAGGGTTAGGGTTTAGGGTTTAGGGTTAGGGTTTAGGGTTTAGGGTTTAGGGTTTAGGGTTTAGGGTTTAGGGTTTAGGGTTTAGGGTTTAGGGTTTAGGGTTAGGGTTTAGGGTTTAGGGTTTAGGGTTTAGGGTTTAGGGTTAGGGTTTAGGGTTAGGGTTTAGGGTTAGGGTTTAGGGTTTAGGGTTTTTAGGGTTTAGGGTTTAGGTTTAGGGTTTAGGGTTTAGGGTTTAGGGTTTAGGGTTTAGGGTTTAGGGTTAGGGTTTAGGGTTTAGGGTTTAGGGTTTAGAGGTTTTTAGGGTTTAGGGTTTAGGGTTTAGGGTTTAGGGTTAGGGTTTAGGGTTTAGGGTTTAGGGTTTAGGGTTTAGGGTTTAGGGTTTAGGGTTTAGGGTTTAGGGTTTAGGGTTTAGGGTTTAGGGTTTAGGTGGTTTAGGGTTAGGGTTTAGAGGGTAGGGTTAGGGTTTAGGGTTTAGGGTTTAGGGTTTAGGGTTTAGGGTTTAGGGTTTAGGGTTTAGGGTTTAGGGTTTAGGGTTTAGGGTTTAGGGTTTAGGGTTTAGGGTTTAGGGTTTAGGGTTTTAGGGTTTAGGGTTTAGGGTTTAGGGTTTAGGGTTTAGGGTTTAGGGTTTAGGGTTTAGGGTTTAGGGTTTAGGGTTTAGGGTTTAGGGTTTAGGGTTTAGGGTTTAGGGTTTAGGGTTTAGGGTTTAGGGTTTAGGGTTTAGGGTTTAGGGTTTAGGGTTTAGGGTTTAGGGTTTAGGGTTTAGGTTAGGGTTTAGGGTTTAGGGTTTAGGGTTTAGGGTTTAGGGTTTAGGTTAGGGTTTAGGGTTTAGGGTTAGGTTTAGGGTTTAGGGTTTAGGGTTAGTTAGGGTTTAGGGTTTAGGGTTTAGGGTTTAGGGTTTAGGGTTTAGGGTTAGGGTTTAGGGTTTAGGGTTTAGGGTTTAGGTGGTTTAGGGTTTAGGGTTTAGGGTTTTGGGTTAGGGTTTAGGGTTTAGGGTTTAGGGTTTAGGGTTTAGGGTTAGGGTTTAGGGGTTTAGGGTTTAGGGTTTAGGGTTTAGGGGTTTAGGGTTTAGGGTTTAGGGTTTAGGGTTTAGGTTTAGGGTTTTGGGTTTAGGGTGGTTAGGGTTTTGGGTTTAGGGTTTAGGGTTTAGGGTTTAGGGTTTAGGGTTAGGGTTTAGGGTTTAGGGTTTAGGGTTTAGGGTTTAGGGTTTAGGGGTTTAGGGTTTAGGGTTAGGGTTTGGGTTTAGGGTTTTGGGTTAGGGTTTAGGGTTTAGGGTTTAGGGTTTAGGGTTTAGGGGTTTAGGGTTTAGGGTTTAGGGTTTAGGGTTTAGGGTTAGGGTTAGGGTTTAGGGATTTAGGGTGTTTAGGGTTAGAGGGTTTAGGGTTTAGGGTTTGGTAGGGTTTAGGGTTTAGGTTGGGTTTAGGGTTTAGGGTTTAGGGTTTAGGGTTGGTTAGGGGTTTAGGGTTTTGGGGTTAGGGTTTTGGGTTTTAGGGTTTAGGGTTAGGGTTAGGGTTGAGGGTTTAGGGTTTAGGGTTTGGGTTGTTTAGGGGTTGTAGGGTTTAGGGTGGTTTGAGGGTTTAGGGTTTAGGGTTAGGGGTTTAGGGGTTTAGGGTTTAGGGTTTAGGGTTTAGGGTTTGGGTGTTGGGTTTTGGGTTTGGGTGTAGGGTTGTGGGTGGTTAGGCGGTGGGGTTAGGGGGTGTGGTGGGGTGTTGGGTTTGGGTAGTGGTTGTGGGGTTTAGGGTTTAGGGTGTTGGGGGTTGGGGTTGTGGGGTGAGGGGGTAGGGTTTAGGGTTAGGGGGG

**6AL**

GG**TTTAGGG**TTTAGGGTTTAGGGTTAGGGTTTAGGGTTTAGGGTTTAGGGTTTAGGGTTTAGGGTTTAGGGTTTAGGGTTTAGGGTTTAGGGTTTAGGGTTTAGGGTTTAGGGTTTAGGGTTTAGGGTTTTAGGGTTTAGGGTTTAGGGTTTAGGGTTTAGGGTTTAGGGTTTAGGGTTTAGGGTTTAGGGTTTAGGGTTAGGGTTTAGGGTTTAGGGTTTAGGGTTTAGGGTTAGGGTTTAGGGTTTAGGGTTTAGGGTTTAGGGTTTAGGGTTAGGGTTTAGGGTTTAGGGTTTAGGGTTTAGGGTTTAGGGTTTAGGGTTTAGGGTTAGGGTTTAGGGTTTAGGGTTTAGGGTTTAGGGTTTAGGGTTTAGGGTTTAGGGTTTAGGGTTAGGATTAGGGTTTAGGGTTTAGGGTTTAGGGTTTAGGGTTAGGGTTAGGGTTTAGGGTTTAGGGTTTAGGGTTAGGGTTTAGGGTTAGGGTTTAGGGTTTAGGGTTTAGGGTTAGGGTTTAGGGTTTAGGGTTTAGGGTTTAGGGTTTAGGGTTTAGGGTTTAGGGTTGGTAGGGTTTAGGGTTTAGGGTTTAGGGTTTAGGGTTTAGGGTTTAGGGTTTAGGGTTTAGGGTTTAGGGTTTAGGGTTTAGGGTTTAGGGTTTAGGGTTTAGGGTTTAGGGTTTAGGGTTTAGGGTTTAGGGTTTTAGGGTTTAGGGTTTAGGGTTTAGGGTTTAGGGTTTAGGGTTTAGGGTTTAGGGTTTAGGGGTTTAGGGTTTAGGGGTTTAGGGTTTAGGGTTTAGGGTTTAGGGTTTAGGGTTTAGGGTTTAGGGTTTAGGGTTTAGGGTTTAGGGTTTTAGGGTTTAGGGTTTAGGGTTTAGGGTTTAGGGTTTAGGGTTTAGGGTTTAGGGTTAGGGTTTAGGGTTTAGGGTTTAGGGTTTAGGGTTTAGGGTTTAGGGTTTAGGGTTTAGGGTTAGGGTTAGGGTTTAGGGTTTAGGGTTTAGGGTTTAGGGTTAGGGTTTAGGGTTTAGGGTTTAGGGTTTAGGGTTTAGGGTTTAGGGTTTAGGGTTTAGGGTTTAGGGTTTAGGGTTTAGGGGTTAGGGTTTAGGGTTTAGGGTTTAGGGTTTAGGGTTTAGGGTTTAGGGTTTAGGGTTTAGGGTTTAGGGTTTAGGGTTTAGGGTTTAGGGTTTAGGGTTTAGGGGTTTAGGGTTAGGGTTTAGGGTGGGTTTAGGGTTTAGGGTTAGGGTTTAGGGTTTAGGGTTTAGGGTTTAGGGGTTTAGGGTTTAGGGTTTAGGGTTTAGGGTTAGGGTTTAGGGTTTAGGGTTTAGGGTTTAGGGTTTAGGGTTTAGGGTTTAGGGTTTAGGGTTAGGGTTTAGGGTTTAGGGTTAGGGTTTAGGGTTTAGGGTTTAGGGTTAGGGTTTAGGGTTAGGGTTTAGGGTTTTAGGGTTTAGGGTTTTAGGGTTTTAGGGTTTTAGGGTTTAGGGTTAGGGTTTAGGGTTTAGGGTTTAGGGTTTAGGGTTTAGGGTTTAGGGTTTAGGGTTTAGGGTTTAGGGTTTAGGGTTTAGGGTTTAGGGTTTAGGGTTTAGGGTTTAGGGTTTAGGGTTTAGGGTTTAGGGTTTAGGGTTTAGGGTTTAGGGTTTAGGGTTTAGGGTTTAGGGTTAGGGTTTAGGGTTTAGGGTTTAGGGTTTAGGGTTTAGGGTTTAGGGTTTAGGGTTTAGGGTTTAGGGTTTAGGGTTTAGGGTTTAGGGTTTAGGGTTTAGGGTTTAGGGTTTAGGGTTTAGGGTTTAGGGTTTAGGGTTTAGGGTTTAGGGTTTAGGGTTTAGGGTTTAGGGTTTAGGGTTTAGGGTTTAGGGTTTAGGGTTTAGGGTTTAGGGTTTAGGGTTTAGGGTAGGTTAGGGTTTAGGGTTAGGGTTTAGGGTTTAGGGTTTAGGGTTTAGGGTTTAGGGTTAGGGTTTAGGGTTTAGGGTTTAGGGTTTAGGGTTTAGGGTTTAGGGTTTAGGGTTTAGGGTTTAGGGTTTAGGGTTTAGGGTTTAGGGTTTAGGGTTAGGTTTAGGGTTTAGGGTTTAGGGTTTAGGGTTTAGGGTTAGGGTTTAGGGTTTTAGGGTTTAGGGTTTAGGGTTTAGGGTTTAGGGTTTAGGGTTTAGGGTTCGGGTTAGGGTTAGGGTTTAGGGTTTAGGGTTTAGGGTTTAGGGTTTAGGGTTTAGGGTTTAGGGTTTAGGGTTTAGGGTTAGGGTTTTTAGGGTTTAGGGTTTAGGGTTTAGGGTTTAGGGTTTAGGGTTTAGGGTTTAGGGTTTAGGGTTAGGGTTTAGGGTTTAGGGTTTAGGGTTTAGGGTTTAGGGTTTAGGGTTTAGGGTTAGGGTTTAGGGGTTTAGGGTTAGGGTTTAGGGTTTAGGGTTAGGGTTTAGGGTTTAGGGTTTAGGGTTTAGGGTTTAGGGTTTAGGGTTTAGGGTTTAGGGTTTAGGGTTTAGGGTTTTAGGGTTTAGGGGTTTAGGGTTTAGGGTTAGGGTTAGGGTTTAGGGTTAGGGTTTAGGGTTTAGGGTTTAGGGTTTAGGGTTTAGGGTTTAGGGTTTAGGGTTTAGGGTTTAGGTTTAGGGTTAGGGTTTAGGGTTTAGGGTTTAGGGTTTAGGGTTAGGGTTTAGGGTTTAGGGTTTAGGGTTTAGGGTTAGGGTTTAGGGTTTAGGGTTTAGGGTTTAGGGTTTAGGGTTTAGGGTTTAGGGTTTAGGGTTTAGGGTTTAGGGTTTAGGGTTCTAGGGTTTAGGTTTAGGGTTTAGGGTTTAGGGTTTTGGGGTTTAGGGTTTAGGGTTAGGGTTTAGGGTTTAGGGTTAGGGTTTAGGGTTTAGGGTTTAGGTTTAGGGTTTAGGGTTAGGGTTTAGGGTTTAGGGTTTAGGGTTTAGGGTTTAGGGTTTAGGGGTTTTAGGGTTAGTTAGGGTTTAGGGTTAGGGTTTAGGTTTAGGGTTTAGGGTTTAGGGTTTAGGGTTTAGGGTTTAGGGTTTAGGGTTTAGGGTTTAGGGTTTAGGGTTTAGGGTTTAGGGTTTAGGGTTTAGGGTTTAGGGTTTAGGGTTTAGGGTTTAGGGTTTAGGGTTTAGGGTTTAGGGTTTAGGGTTTAGGGTTAGGGTTTAGGGTTTAGGGTTTAGGGTTTAGGGTTTAGGGTTTAGGGTTTAGGGTTTAGGGTTTAGGGTTTAGGGTTTAGGGTTAGGGTTTAGGGTTTAGGGTTTAGGGTTAGGGTTTAGGGTTTAGGGTTTAGGGTTTAGGGTTTAGGGTTTAGGGTTTAGGGTTTAGGGTTTAGGGTTTAGGGTTTAGGGTTTAGGGTTTAGGGTTTAGGGTTTAGGGTTTAGGGTTTAGGGTTTAGGGTTTAGGGTTTAGGGTTTAGGGTTTAGGGTTTAGGGTTTAGGGTTTAGGGTTTAGGGTTTAGGGTTTAGGGTTTAGGGTTTAGGGTTTAGGGTTTAGGGTTTAGGGTTTGGGTTTAGGGTTTAGGGTTTAGGGTTTAGGGTTTAGGGTTTAGGGTTTAGGGTTAGGTTTAGGGTTTAGGGTTTAGGGTTTAGGGTTTAGGGTTTAGGGTTAGGGTTTAGGGTTTAGGGTTTAGGGTTTAGGGTTTAGGGTTTAGGGTTTAGGGTTTAGGGTTTAGAGGGTTTAGGGTTAGGTTTAGGGTTTAGGGTAGGGTTTAGGGTTTAGGGTTTAGGGTTTAGGGTTTAGGGTTTAGGGTTTAGGGTTTAGGGTTTAGGGTTTAGGGTTTAGGGTTTAGGGTTTAGGGTTTAGGGTTAGGGTTTAGGGTTTAGGGGTTTAGGGTTTAGGGTTTAGGGATTTAGGGTTTAGGGGTTTAGGGTTAGGGTTTAGGGTTTAGGGTAGGGTTTAGGGTTTAGGGTTTAGGGTTTAGGGTTTAGGGTTTAGGGTTTAGGGTTTAGGGTTTAGGGTTGTAGGGTTTAGGGGTTTAGGGTTTAGGGTTTAGGGTTTAGGGTTTAGGGTTTAGGGTTTAGGGTTTAGGGTTTAGGGTTTAGGGTTTAGGGTTAGGGTTAGTGGTTTAGGGTTTAGGGTTTAGGGTTTGTAGGGTTTAGGGTTTAGGGTTAGGGTTAGGGTTTTAGGGTTTAGGGTTTAGGGGTTTAGGGTTTAGGGTTTAGGGTTTAGGGTTTAGGGTTTAGGGTTTAGGGTTTAGGGTTTACGGGTTTAGGGTTAGGGTTTAGAGGGTTTAGGG

**7AS**

TTAAGGGTTAAGGG**TTTAGGG**TTTATGGTTTAGGGTTTAGGGTTTAGGGTTTAGGGTTTAGGTTTAGGTTTAGGGTTTAGGGTTTAGGGTTTAGGGTTTAGGGTTTAGGGTTTAGGGTTTTAGGGTTTAGGGTTTAGGGTTTAGGGTTTAGGGTTTAGGGTTTAGGGTTTAGGGTTTAGGGTTTAGGGTTTAGGGTTAGGTTTAGGGTTTTTAGGGTTTTAGGTTTAGGGTTTAGGGTTTAGGGTTTAGGGTTTAGGGTTTAGGGTTTAGGGTTTAGGGTTTAGGGTTTAGGGTTTTAGGGTTTAGGGTTTAGGGTTTAGGGTTTAGGGTTAGGGTTAGGGTTTAGGGTTTAGGGTTTAGGGTTTAGGGTTTAGGGTTTAGGGTTTAGGTTTTTAGGGTTTAGGGTTAGGGTTAGGGTTTAGGGTTAGGGTTAGGGTTAGGGTTTAGGGTTTAGGGTTAGGGTTTAGGGTTAGGGTTTAGGGTTTAGGGTTTAGGGTTTAGGGTTTAGGGTTTAGGGTTTAGGGTTTAGGGTTAGGGTTTAGGGTTTAGGGTTAGGGTTTAGGGTTTAGGGTTTAGGGTTAGGGTTTAGGGTTTAGGGTTTAGGGTTGGGTTTAGGGTTTTTTAGGGTTTAGGGTTTAGGGTTTAGGGTTTAGGGTTTAGGGTTTAGGGTTTAGGGTTTAGGGTTAGGGTTTAGGGTTTAGGGTTAGGGTTTAGGGTTTAGGGTTTAGGGTTTAGGGTTAGGGTTTAGGGTTTAGGGTTTAGGGTTTAGGGTTTAGGGTTTAGGGTTTAGGGTTTAGGGTTTAGGGTTAGGGTTTAGGGTTTAGGGTTTAGGGTTTAGGGTTTAGGGTTAGGGTTTAGGGTTTAGGGTTTAGGGTTAGGGTTTAGGGTTTAGGGTTTAGGGTTTAGGGTTTAGGGTTTAGGGTTTAGGGTTAGGGTTTAGGGTTTAGGGTTTAGGGTTTAGGGTTTAGGGTTTAGGGTTTAGGGTTTAGGGTTTAGGGTTAGGTTTAGGGTTTAGGGTTTAGGGTTTAGGGTTAGGGTTAGGGTTTAGGGTTTAGGGTTTAGGGTTTAGGGTTTAGGGTTTAGGGTTTAGGGTTTAGGGTTTAGGGTTTAGGGTTAGGGTTTAGGGTTTAGGGTTAGGGTTTAGGGTTTAGGGTTTAGGGTTTAGGGTTTAGGGTTTAGGGTTTAGGGTTTAGGGTTTAGGGTTTAGGGTTTAGGGTTTAGGGTTTAGGGTTTAGGGTTTAGGGGTTTAGGGTTTAGGGTTTAGGGTTTAGGGTTAGGGTTTAGGGTTTAGGGTTTAGGGTTTAGGGTTAGGGTTTAGGGTTAGGGTTTAGGGTTTAGGGGTTTAGGGTTTAGGGTTTAGGGGTTGGGTTTAGGGTTAGGGTTTAGGTTTAGGGTTTAGGGTTTAGGGTTTAGGGTTTAGGGTTTAGGGTTTAGGGTTTAGGGTTTAGGTTTAGGGTTTAGGTTTAGGGTTTAGGGTTTAGGGTTAGGGTTTAGGGTTTAGGGTTAGGGTTTAGGGTTTAGGGTTAGGGTTTAGGGTTTAGGGTTTAGGGTTTAGGGTTTTAGGGTTTAGGGTTTAGGGTTTAGGGTTAGGGTTTAGGGTTAGGGTTAGGGTTTAGGGTTTAGGGTTAGGGTTTAGGGTTTAGGGTTTAGGGTTAGGGTTTAGGGTTAGGGTTTAGGGTTTAGGGTTTAGGGTTTGGGTTTAGGGTTTGTAGGGTTTAGGGTTTAGGGTTTAGGGGTTAGGGGTTAGGGTTTAGGGTTTAGGGTTTAGGGTTTAGGGTTTAGGGTTTAGGGTTTAGGGTTTAGGGTTTAGGGTTTAGGGTTTAGGGTTAGGGTTAGGGTTTAGGGGTAGGGTTTAGGGTTTAGGGTTGTTTAGGGTTTAGGGTTTAGGGTTTAGGGTTTAGGGTTTAGGGTTTAGGGTTAGGGTTAGGGTTTAGGGTTTAGGGTTTAGGTTGGTTTAGGGTTAGGGTTTAGGGTTTAGGATTAGGGTTTAGGGATTAGGGTTTAGGGTTTAGGGTTTAGGTTAGGGTTTAGGGTTTAGGGTTTAGGGTTTAGGTTTAGGGTTTAGGGTTTAGGGTTTGGGTTTAGGGTTTAGGGTTTAGGGTTTAGGGTTAGGGTTTAGGGTTAGGGTTTAGGGTTTAGGGTTAGGGTTAGGTGTTTAGGGTTAGGGTTTAGGGTTGGGTTTAGGGTTAGGGTTTAGGGTTAGGGTTTAGGGTTTAGGTTAGGGTTAGGGTTTAGGGTTTAGGGGTTTAGGGTTTGGGTTAGGGTTGGGTTTAGGGTTTAGGGTTTAGGGTTTAGGGTTTAGGGTTTAGGGTTTAGGGTTTAGGGTTTAGGGTTAGGGTTTAGGGTTAGGGTTTAGGGTTAGGGTTTAGGTTTAGGGTTTAGGGTTAGGGTTTAGGGTAGGGTTTAGGGTTTAGGGTTTAGGGTTAGGGTTTAGGGTTTAGGGTTTAGGGTTTAGGGTTTAGGGTTTAGGGTTTAGGGTTTAGGGTAGGTTTAGGGTTTAGGGTTTAGGGTTTAGGGTTTAGGGTTTAGGGTTTAGGGTTTAGGGTTTAGGGTTTAGGGTTTAGGGTTTAGGGTTTAGGGTTTAGGGTTAGGGTTTAGGGTTTAGGGTTTAGGGTTAGGGTTTAGGGTTTAGGGTTTAGGGTTTAGGGTTTAGGGTTTAGGGTTTAGGGTTAGGGTTTAGGGTTTAGGGTTTTAGGGTGTTAGGGTTTAGGGTTTTAGGGTTTAGGGTTTAGGGTTTAGGGTTTAGGGTTAGGGTTTAGGGTTTAGGGTTTAGGGTTTAGGGTTAGGGTTTAGGGTTTAGGGTTTAGGGTTTAGGGTTTAGGGTTAGGGTTAGGGTTTAGGGTTTAGGGTTTAGGGTGTTTAGGGTTTAGGGTTTAGGGGTTTAGGGTTAGGGTTTAGGGTTTAGGGTTTAGGGTTTAGGGTTTAGGGTTTAGGGTTTTAGGGTTTAGGGTTTGGGTTTAGGGTTTAGGGTTTAGGGTTTAGGGTTTAGGGTTTAGGGTTTAGGGTTTAGGGTTTTAGGGTTTGGGTTGTTTAGGGTTTGGGTTTAGGGTTTGGGTTTAGGGTTAGGGTTTTAGGGTGTGGGTGGGTTTAGGGTTGGGTTTAGGGTTTAGGGTTTAGGGTTTAGGGTAGGTTTAGGGGTTAGGGTTTAGGGTTTAGGGTTTAGGGTTTAGGGTTTAGGGTTTAGGGTTAGGGTTTAGGGTTTTAGGGTTTGGGGTTTTTAGGGTTTAGGGTTTAGGGTTTAGGGTTTAGGGGTTAGGGTTTAGGGTTTAGGGTTTAGGTTTAGGGTTTAGGGTTTAGGGTTTAGGGTTTAGGGTTTAGGGTTAGGGTTTAGGGTTTAGGGTTAGGGTTTAGGGTTTAGGGTTTAGGGTTTTAGGGTTTAGGGTTTAGGGTTTAGGGTTTAGGGTTAGGGTTTAGGGTTTAGGGTTTAGGGTTTAGGGTTAGGGTTTAGGGTTTAGGGTTTAGGGTTAGGGTTTAGGGTTTAGGGTTTAGGGTTAGGGTTTAGGTTTAGGGTTTAGGGTTTAGGGTTTAGGTTTAGGGTTTAGGGTTTAGGGTTTAGGGTTTAGGGTTTAGGGTTAGGGTTAGGGTTTAGGGTTTAGGGTTAGGGTTTAGGGTTTAGGGTTTAGGGTTTAGGGTTTAGGGTTTAGGGTTTAGGGTTTTGGGTTTAGGGTAGGGTTTAGGGTTTAGGGTTTAGGGTTTAGGGTTTAGGGTTAGGGTTAGGGTTTAGGGTTTAGGGTTTAGGGTTAGGGTTAGGGTTTAGGGTTTAGGGTTTAGGGTTTAGGTTAGGGTTTAGGGGTTTAGGGTTTAGGGTTTAGGTTTAGGGTTTAGGGTTTAGGGTTTAGGGTTTAGGTTTAGGGTTTGAGTTAGGGTT

**7AL**

AAGGTAGGG**TTTAGGG**TTTAGGGTTTAGGGTTAGGGTTTTGGTTTAGGATTAGGGTTAGGGTTTAGGTTTTAGGGTTTAGGGTTAGGGTTAGGTTTAGGGTTTAGGGTTTAGGGTTTAGGGTTTAGGGTTTAGGGTTTAGGGTTTAGGGTTTAGGGTTTAGGGTTTAGGGTTAGGGTTTAGGGTTTAGGGTTTAGGGTTTAGGGTTTAGGGTTTAGGGTTTAGGGTTTTTTTAGGGTTTTTTAGGGTTGTTTTGGGTTTAGGGTTTAGGGTTTAGGGTTTAGGGTTTAGGGTTTAGGGTTTAGGGTTTAGGGTTTAGGGTTTAGGGTTTAGGGTTAGGGTTAGGGTTTAGGGTTTAGGGTTTAGGGTTTAGGGTTTAGGGTTTAGGGTTTAGGGTTTAGGGTTTAGGGTTTAGGGTTAGGGTTTAGGGTTAGGGTTAGGGTTTAGGGTTTAGGGTTTAGGGTTTAGGGTTTAGGGTTAGGGTTTAGGGTTTAGGGTTAGGGTTAGGGTTTAGGGTTTAGGGTTAGGGTTAGGGTTTAGGGTTTAGGGTTTAGGGTTTAGGGTTTAGGGTTTAGGGTTAGGGTTTAGGGTTTAGGGTTAGGGTTTAGGGTTTAGGGTTAGGGTTTAGGGTTTAGGGTTTAGGGTTTTAGGGTTTAGGGTTTAGGGTTTAGGGTTAGGGTTTAGGGTTTAGGGTTTAGGGTTTAGGGTTTTAGGGTTTAGGGTTTAGGGTTTAGGGTTAGGGTTTAGGGTTTAGGGTTTAGGGTTTAGGGTTTAGGGTTTAGGGTTTAGGGTTTAGGGTTTAGGGTTTAGGGTTTAGGGTTTGGGGTTTAGGGTTAGGGTTTAGGGTTTAGGGTTTAGGGTTTAGGGTTTAGGGTTTAGGGTTTAGGGTTTAGGGTTTAGGGTTTAGGGTTTAGGGTTTTAGGGTTTTAGGGTTTAGGGTTTAGGGTTTAGGGTTTAGGGTTAGGGTTTAGGGTTTAGGGTTTAGGGTTTAGGGTTTAGGGTTTAGGGTTTAGGGTTTAGGGTTAGGGTTTAGGGTTTTAGGGTTTAGGGTTTAGGGGTTTAGGGTTTAGGGTTTAGGGTTTAGGGTTTAGGGTTTAGGGTTTAGGGTTTAGGGTTTAGGGTTTAGGGTTTAGGGTTTAGGGTTTAGGGTTTAGGGTTTTAGGGTTTAGGGTTTAGGGTTTAGGGTTTAGGGTTAGGGTTTAGGGTTTAGGGTTTAGGGTTTAGGGTTTAGGGTTAGGGTTTAGGGTTAGGGTTTAGGGTTTAGGGTTAGGGTTTAGGGTTTAGGGTTAGGGTTTAGGGTTTAGGGTTTAGGGTTTGGGGTTTAGGGTTTAGGGTTTAGGGTTTAGGGTTTAGGGTTTAGGGTTTAGGGTTAGGGTTTAGGGTTAGGGTTTAGGGTTTAGGGTTTAGGGTTTAGGGTTTAGGGTTTAGGGTTTAGGGTTTAGGGTTTAGGGTTTAGGGTTTAGGGTTTAGGGTTGTTAGGGTTTAGGGTTTAGGGTTTAGGGTTAGGGTTTAGGGTTTTAGGGTTTAGGGTTTTGGGTTTAGGGTTTAGGGTTTAGGGTTAGGGTTTAGGGTTTAGGGTTAGGGTTTAGGGTTTAGGGTTTAGGGTTGTTATGGGGTTTTAGGGTTTAGGGTTTAGGGTTTAGGGTTTAGGGTTTAGGGTTAGGGTTTGGGGTTTAGGGTTTAGGGTTTTAGGGTTTAGGGTTTAGGGTTTAGGGTTTAGGGGTTTAGGGTTTAGGGTTTTAGGGTTTAGGGTTTAGGGTTTAGGGTTAGGGTTAGGGTTTAGGGTTTAGGGTTTAGGGTTTAGGGTTTAGGGTTTAGGGTTTAGGGTTTTACGGGTTTAGGGTTTAGGGTTTTAGGGTTGGGGTTTAGGGTTTAGGGTTTAGGGTTAGGGTTTAGGGTTTAGGGTTAGGGTTTAGGGTTAGGGTTTAGGGTTTAGGGTTTAGGGTTAGGGTTAGGGTTTAGGGTTTAGGGTTTAGGGTTTAGGGTTTAGGGTTTAGGGTTTAGGGTTTAGGGTTTAGGGTTTTTAGGGTTTAGGGTTTAGGGTTAGGGTTTAAGGGTTTAGTAGGGTTTAGGGTTTAGGGTTTAGGGTTAGGGTTTAGGGTTTAGGGTTTAGGGTTTAGGGTTAGGGTTTAGGGTTTAGGGTTGGGGTTAGGGTTTAGGGTTTAGGGTTTAGGGTTTAGGGTTAGGGTTTAGGGTTTAGGGTTTAGGGTTTAGGTTTAGGGTTTAGGGTTTAGGGTTTAGGGTTAGGGTTTAGGGTTTAGGGTTAGGGTTTGTTTAGGGTTTAGGGTTTAGGGTTTAGGGTTTAGGGTTTAGGGTTTAGGGTTTAGGGTTTAGGGTTTAGGGTTTAGGGTTTAGGGTTAGGGTTTAGGGTTTAGGGTTAGGGTTTTAGGGTTTAGGGTTTAGGGTTAGGGTTTAGGGTTTAGGGTTAGGGTTTAGGGTTTAGGGTTTAGGGTTTAGGGTTTAGGGTTTAGGGTTTAGGGTTTAGGGTTTAGGGTTTAGGGTTTAGGGTTTAGGGTTTAGGGTTTAGGGTTTAGGGTTTAGGGTTAGGGTTTAGGGTTAGGGTAGGTTAGGGTTTAGGGTTTAGGGTTTAGGGTTAGGGTTTAGGGTTTAGGGTTTAGGGTTAGGGTTAGGGTTTAGGGTTTAGGGTTTAGGGTTTAGGGTTTAGGGTTAGGGTTTAGGGTTAGGTTTAGGGTTTAGGGTTTAGGGTTTAGGGTTTAGGGTTTAGGGTTTAGGGTTTAGGGTTAGGGTTTAGGGTTTAGGGTTTAGGGTTTAGGGTTTAGGGTTAGGGTTTAGGGTTTAGGGTTAGGGTTTAGGGTTTAGGGTTTAGGGTTAGGGTTTAGGGTTTAGGGTTAGGGTTAGGGTTTAGGGTTTAGGGTTAGGGTTTAGGGTTTAGGGTTAGGGTTAGGGTTAGGGTTTAGGGTTAGGGTTTAGGGTTTAGGGTTTAGGGTTTAGGGTTTAGGGTTTAGGGTTTAGGGTTTAGGGTTGGGGTTTAGGGTTTAGGGTTTAGGGTTTAGGGTTTAGGGTTTAGGGTTTAGGGTTTAGGGTTTAGGGTTTAGGGTTTAGGGTTTTGGGTTTAGGGTTTAGGGTTTAGGGTTAGGGTTAGGTTTAGGGTTAGGGTTTAGGGTTTAGGGTTAGGGTTTAGGGTTTAGGGTTTAGGGATAGTTTAGGGTTTAGGGTTAGGGTTTAGGGGTTTAGGGTTTAGGGTTTGGGTTAGGGTTTAGGGTTTAGGGTTTAGGGTTTAGGGTTTAGGGTTTAGGGTTAGGTTAGGGTTTAGGGTTTAGGGTTTAGGGTTAGGGTTTAGGGTTTAGGGTTAGGGTTTAGGGTTTAGGGTTTTAGGGTTTAGGGTTTAGGGTTTAGGGTTTAGGGTTATAAGGGTTGGGTTGGTTAGGGTTTAGGGTTTAGGGTTAGGGTTTAGGGTTTAGGGTTTTAGGGTTAGGGTTTAGGGGTTTAGGGTTTTAGGGTTTAGGGTTTAGGGGATTAGGGTTTTGGGTTTAGGGTTTTAGGGTTTAGGGTTTAGGGTTAGGGTTTAGGGTTTAGGGTTAGAGGGTTTAGGGTTTAGGGTTTAGGGTTAGGGTTTAGGGTTAGGGTTAGGGTTAGGGTTTTAGGGTTAGGGTTTAGGGTTTTAGGGTTAGGGTTTAGGGGTTTAGGGGTTTAGGGTTTAGGGTTTTAGGGTTTA

**1BS**

AGGG**TTTAGGG**TTTAGGGTTTAAGGGTTTAGGGTTAGGGTTTAGGGTTTAGGGTTTAGGGTTTAGGGTTAGGGTTAGGGTTTAGGGTTTAGGGTTAGGGTTTAGGGTTTAGGGTTTAGGGTTTAGGGTTTAGGGTTTAGGGTTTAGGGTTAGGGTTTAGGGTTTAGGGTTCAGGGTTAGGGTTTAGGGTTTAGGGTTTAGGGTTTAGGGTTTAGGGTTTAGGGTTTAGGGTTTAGGGTTTAGGGTTAGGGTTTAGGGTTTAGGGTTAGGGTTTAGGGTTAGGGTTTAGGGTTTAGGGTTTAGGGTTTAGGGTTTAGGGTTAGGGTTTAGGGTTTAGGGTTTAGGGTTTAGGGTTTTAGGGTTTAGGGTTTAGGGTTTAGGGTTTAGGGTTTAGGGTTTAGGGTTTAGGGTTTAGGGTTTAGGGTTTAGGGTTTAGGGTTAGGGTTTAGGGTTAGGGTTTAGGGTTTAGGGTTTAGGGTTTAGGGTTAGGGTTAGGGTTTAGGGTTTAGGGTTTAGGGTTAGGGTTAGGGTTTAGGGTTAGGGTTTAGGGTTTAGGGTTTAGGGTTTAGGGTTTAGGGTTTAGGGTTTAGGGTTTAGGGTTTAGGGTTTAGGGTTTAGGGTTTAGGGTTAGGGTTTAGGGTTTAGGGTTTAGGGTTAGGGTTTAGGGTTTAGGGTTTAGGGTTTAGGGTTTAGGGTTTAGGGTTTAGGGTTTAGGGTTTAGGGTTTAGGGTTTAGGGTTTAGGGTTTAGGGTTTAGGGTTTAGGGTTTAGGGTTTAGGGTTTAGGGTTTAGGGTTTAGGGTTTAGGGTTTAGGGTTTAGGGTTTAGGGTTTAGGGTTTAGGGTTTAGGGTTTTTGGGTTTAGGGTTTAGGGTTTAGGGTTTAGGGTTTAGGGTTTCTAGGGTTTAGGGTTTAGGGTTTAGGGTTTAGGGTTTAGGGTTTAGGGTTAGGGTTTAGGGTTTAGGGTTTAGGGTTTAGGGTTTAGGGTTTAGGGTTTAGGGTTTAGGGTTTAGGGTTAGGGTTTAGGGTTTAGGGTTTAGGGTTTAGGGTTTAGGGTTTAGGGTTTAGGGTTTAGGGTTTAGGGTTTTTAGGGTTTAGGGTTTAGGGTTTAGGGTTTAGGGTTTAGGGTTTAGGGTTTAGGGTTTAGGGTTTAGGGTTTAGGGTTTAGGGTTTAGGGTTTAGGGTTTAGGGTTTAGGGTTAGGGTTTAGGGTTTAGGGTTAGGGTTTAGGGTTTAGGGTTTAGGGTTTAGGGTTTAGGGTTTAGGGTTAGGGTTTAGGGTTTAGGGTTTTAGGGTTTAGGGTTTTAGGGTTTTTAGGGTTTAGGGTTTAGGGTTTAGGGTTTAGGGTTTAGGGTTTAGGGTTTAGGGTTTAGGGTTTAGGGTTTAGGGTTTAGGGTTTAGGGTTTAGGGTTTAGGGTTTAGGGTTAGGGTTTAGGGTTTAGGGTTTAGGGTTTAGGGTTTAGGGTTTAGGGTTTAGGGTTTAGGGTTTAGGGTTTAGGGTTTAGGGTTTAGGGTTTAGGGTTTAGGGTTTAGGGTTTAGGGTTTAGGGTTTAGGGTTAGGGTTTAGGGTTAGGGTTTAGGGTTTAGGGTTTAGGGTTTAGGGTTTAGGGTTTAGGGTTTAGGGTTTAGGGTTTAGGGTTTAGGGTTTAGGGTTTAGGGTTTAGGGTTTAGGGTTTAGGGTTTAGGGTTTAGGGTTAGGGTTTAGGGTTTAGGGTTTAGGGTTTAGGGTTTAGGGTTTAGGGTTTAGGGTTTAGGGTTTAGGGTT

**3BL**

TGGG**TTTAGGG**TTTAGGGTTTAGGGTTTAGGGTTTAGGGTTTAGGGTTTAGGTTTTAGGGTTTAGGGTTTAGGGTTTAGGGTTTAGGGTTAGGGTTTAGGGTTAGGGTTTAGGGTTTAGGGTTTAGGGTTTAGGGTTAGGGTTTAGGGTTTAGGGTTTAGGGTTTAGGGTTTAGGGTTTAGGGTTTAGGGTTTAGGGTTAGGTTTAGGGTTTAGGGTTTAGGGTTAGGGTTTAGGGTTTAGGGTTTAGGGTTTAGGGTTTAGGGTTTAGGGTTAGGGTTTAGGGTTTAGGGTTTAGGGTTTAGGGTTAGGGTTTAGGGTTTAGGGTTTAGGGTTTAGGGTTTAGGGTTTAGGGTTTAGGGTTTAGGGTTTAGGGTTTTGGGTTTAGGGTTTAGGGTTTAGGGTTTAGGGTTTAGGGTTTAGGGTTTTTTAGGGTTTAGGGTTTAGGGTTTAGGGTTTAGGGTTTAGGGTTTAGGGTTTAGGGTTTAGGGTTTAGGGTTTAGGGTTTAGGGTTTAGGGTTTAGGGTTTAGGGTTTAGGGTTTAGGGTTTAGGGTTTAGGGTTTAGGGTTTAGGGTTTAGGGTTTAGGGTTAGGGTTAGGGTTAGGGTTTAGGGTTTAGGGTTTAGGGTTTAGGGTTTAGGGTTAGGGTTTAGGGTTTAGGGTTTAGGGTTTAGGGTTTAGGGTTTAGGGTTTAGGGTTTAGGGTTTAGGGTTTAGGGTTTAGGGTTTTAGGGTTTAGGGTTTAGGGTTTAGGGTTTAGGGTTTAGGGTTTAGGGTTTAGGGTTTAGGGTTTAGGGTTTAGGGTTTTTAGGGTTTTAGGGTTTAGGGTTTAGGGTTTAGGGTTTAGGGTTTAGGGTTTAGGGTTTAGGGTTTAGGGTTTAGGGTTTAGGGTTTAGGGTTTAGGGTTTAGGGTTTAGGGTTTAGGGTTTAGGGTTTAGGGTTTAGGGTTTAGGGTTTAGGGTTTAGGGTTTAGGGTTTAGGGTTTAGGGTTTAGGGTTTAGGGTTTAGGGTTTAGGGTTTAGGGTTTAGGGTTTAGGGTTTAGGGTTTAGGGTTTAGGGTTTAGGGTTTAGGGTTTAGGGTTTAGGGTTTAGGGTTTAGGGTTTAGGGTTTAGGGTTAGGGTTTAGGGTTTAGGGTTTAGGGTTTAGGGTTTAGGGTTAGGGTTTAGGGTTTAGGGTTTAGGGTTTAGGGTTTAGGGTTAGGGTTTAGGGTTTAGGGTTTAGGGTTTAGGGTTTAGGGTTTAGGGTTTAGGGTTTAGGGTTTAGGGTTTAGGGTTTAGGGTTTAGGGTTTAGGGTTTAGGGTTTAGGGTTTAGGGTTTAGGGTTTAGGGTTTAGGGTTTAGGGTTTAGGGTTTAGGGTTTAGGGGTTAGGGTTTAGGGTTTAGGGTTTAGGGTTTAGGGTTTAGGGTTTAGGGTTTAGGGTTTAGGGTTTTAGGGTTTAGGGTTAGGGTTTAGGGTTTAGGGTTTAGGGTTAGGGTTAGGGTTTAGGGTTTAGGGTTTAGGGTTTAGGGTTTAGGGTTTAGGGTTTAGGGTTTAGGGTTAGGGTTTAGGGTTTAGGGTTTAGGGTTAGGGTTTAGGGTTTAGGGTTTAGGGTTTAGGGTTTAGGGTTTAGGGTTTAGGGTTTAGGGTTTAGGGTTTAGGGTTTAGGGTTTAGGGTTTAGGGTTTAGGGTTTAGGGTTTAGGGTTTAGGGTTTAGGGTTTAGGGTTTAGGGTTTAGGGTTTAGGGTTTAGGGTTTAGGGTTTAGGGTTTAGGGTTTAGGGTTTAGGGTTTAGGGTTTAGGGTTTAGGGTTTAGGGTTTAGGGTTAGGGTTTAGGGTTTAGGGTTTAGGGTTTAGGGTTTAGGGTTTAGGGTTTAGGGTTTTAGGGTTTAGGGTTTAGGTTTAGGGTTTAGGGTTTAGGGTGGTTTAGGGTTTAGGGTTTAGGGTTTAGGGTTTAGGGTTAGGGTTTAGGGTTTAGGGTTTAGGGTTTTTAGGGTTTAGGGTTTAGGGTTTTAGGGTTTAGGGTTTAGGGTTTAGGGTTTAGGGTTAGGGTTTAGGGTTTAGGGTTTAGGGTTTAGGGTTTAGGGGTTTAGGGTTTAGGGTTAGGGGTTTAGGGTTAGGATTAGGGTTTTAGGGTTTTAGGGTTTAGGGTTTAGGGTTTAGGGTTTAGGGTTTAGGGTTTAGGGTTTAGGGTTTAGGGTTTAGGGTTTAGGGTTTAGGGTTTAGGGTTTAGGGGTTTAGGGTTTAGGGTTTAGGGTTTAGGGTTTAGGTTTTAGGGTTTAGGGTTAGGGTTTAGGGTTTAGGGTTTAGGGTTTAGGGTTTAGGGTTTAGGGGTTTAGGGGTTTAGGGTTTTAGGGTTTAGGGTTTAGGGTTTAGGGTTTTTAGGGTTTAGGGTTTAGGGTTTAGGGTTTAGGGTTTAGGGTTTAGGGTTAGGGTTTTAGGGTTTAGGGTTTAGGGTTTAGGGTTTAGGGTTTAGGGTTTAGGGTTTAGGGTTTAGGGTTAGGGTTTAGGGTTTAGGGTTTAGGGTTAGGGTTTAGGGTTTAGGGTTTAGGGTTTAGGGTTTAGGGTTTAGGGTTTAGGGTTTAGGGTTTAGGGTGGTTTAGGGTTTAGTGTTTAGGGTTTAGGGTTTAGGGTTTAGGGTTTAGGGTTTAGGGTTTAGGGTTTAGGGTTTAGGGTTTAGGGTTTAGGGTTTTAGGGTTTTAGGGTTTAGGGTTTAGGGTTTAGGGTTTTAGGGTTTAGGGTTTTAGGGTTTAGGGTTAGGGTTTAGGGTTTAGGGTTAGGGTTTAGGGTTTAGGGTTTAGGGTTTAGGGTTTAGGGTTTAGGGTTTAGGGTTTAGGGTTTTAGGGTTTAGGGTTTAGGGTTTAGGGTTTAGGGTTTAGGGTTTTAGGGTTTAGGGTTTAGGGTTTAGGGTTATAGGGTTTAGGGTTTAGGGTTTAGGGTTTAGGGTTTAGGGTTAGGGTTTAGGGTTTAGGGTTTAGGGTTTAGGGTTTAGGGTTTAGGGTTTAGGGTTTAGGGTTTTAGGGTTAGGGTTAGGGTTTAGGGTTAGGGGTTAGGGTTTAGGGTTTAGGGTTTAGGTTTAGGGTTTAGGGTTTTAGGGTTTAGGGTTTAGGGTTTAGGGTTTAGGGTTTAGGGTTTAGGGTTTAGGGTTTAGGGTTTTAGGGTTTAGGGGTTTAGGGTTAGGGTTTAGGGTTAGGGTTTAGGGTTTAGGGTATAGGGTTTAGGGTTTAGGGTTAGGGTTTTAGGGTTTAGGGTTTAGGGTTTAGGGTTTAGGGTAGGGTTTAGGGTTAGGGTTAGGGTTTAGGGTTAGGGTTAGGGTTAGGGTTTAGGGTTTAGGGTTAGGGTTTAGGGTGTAGGGTTAGGGTTTAGTGGTTTAGGGTTTAGGGTTTAGGGTTTGGGTTTAGGGTTTTAGGGTTTAGGGTTTAGGGTTTAGGGTTTAGGGTTTAGGGGTTAGGGTTAAGGGTTTAGGGTTTAGGGTTAGGGTTTAGGGTTTGGGTTTAGGGTTTAGGGTTTAGGGTTAGGGTTTAGGGTTTAGGGTTTAGGGTTAGGTTAGGGTTTAGGGTTTAGGGTTTAGGGTTTAGGGTTTTAGGGTTTAGGGTTTAGGGTTAGGGTTTAGGGTTTAGGGTGTAGGGTTTAGGGTTTAGGGTTTAGGGTTTGGGTTTAGGGTTTAGGGTTTAGGGTTTAGGGTTTAGGGTTTAGGGTTTAGGGTTAGGGTTTAGGGTTTAGGGTTAGGGTTTAGGTTTTAGGGTTTAGGGTTTAGGGTTTAGGGTGTAGGGTTGTGGGGTGTAGGGTTAGGGTTTTGGGGTTTTGGAGGAGGGTGTAGGGTTTAGGGTTTTGGGTGGTAGGGTTAGGGGTAGGGTGGTTGGGGTTAGGGTTTAGGGTTTAGGGTTAGGGTGAGGGGGTAGGGGTTTAGGGGTTTAGGGTTAGGGTGAGGGTTGAGGGTTTGGGGTTAGGGGTTAGGGTGTAGGGGTGGGTTAGGGTGTAGGGTTGTGGGTGGTAGGGGTGTTGGGTTAGGGGGGTGAGGGTTTTGGGGTAGGGTGGAGGGTTTAGGGTGGTGGGAGTAGAGGGGGGGAGGGTGTAGGGTTGAGTGGGGTGAGGGGGTTGGGGGGTTAGGGTGGGTTGGGGTGAGGGGGGAGGGGGGAGGGGGTAGGGGGGTGGGGGGTGGGGGTGGGAGGGGGGTGGGAGGGGTGGGGTGTGGGGGGTGGGGGGTGGGGGTTGGGGGTGGGGTTGGGGGTGGGGTGGTGGGGGTGGGGGTTGGGGGGTGGGGGTGGGGGTGGGGGTGGGGGGTGGGGTGTGGGGGTAGGGTGGGGTGTGGGGGTGGAGGGGGGTGGGGGGAGGGGGGGTGGGGGAGGGGGGTGGGGGGTGGGTGGAGGGGGAGGGGGGAGGGGGGTGGGGGGTGGGGGGAGGGGGGAGGGGGGGTGAGGGGGAGGGGGTGGGGGGTGGGGGTGGGGGGTGGGGGGTGGGGGGTGGGGGGGTGGGGGGTGGGGGGAGGGGGGTGGGGGGTGGGGGGGG

**1DS**

AAGGG**TTTAGGG**TTTAGGGTTAGGGTTTAGGGTTAGGGTTTAGGGTTTAGGGTTTAGGGTTTAGGGTTTAGGGTTAGGGTTTAGGGTTTAGGGTTAGGGTTTAGGGTTTAGGGTTTAGGGTTTAGGGTTTAGGGTTTAGGGTTTAGGGTTTAGGGTTTAGGGTTTAGGGTTTAGGGTTTAGGGTTTAGGGTTTAGGGTTTAGGGTTTAGGGTTTAGGGTTAGGGTTTAGGGTTTAGGGTTAGGGTTTAGGGTTTAGGGTTAGGGTTTAGGGTTTAGGGTTTAGGGTTTAGGGTTAGGGTTTAGGGTTTAGGGTTAGGGTTTAGGGTTTAGGGTTTAGGGTTTAGGGTTTAGGGTTTAGGGTTTAGGGTTTAGGGTTTAGGGTTTAGGGTTAGGGTTTAGGGTTTAGGGTTTAGGGTTTAGGGTTTAGGGTTTAGGGTTTAGGGTTTAGGGTTTAGGGTTTAGGGTTTAGGGTTTAGGGTTTAGGGTTTAGGGTTTAGGGTTTAGGGTTTAGGGTTTAGGGTTTAGGGTTTAGGGTTTAGGGTTTAGGGTTTAGGGTTTAGGGTTTAGGGTTTAGGGTTTAGGGTTTAGGGTTTAGGGTTTAGGGTTAGGGTTTAGGGTTTTAGGGTTTAGGGTTTAGGGTTTAGGGTTTAGGGTTAGGGTTAGGGTTTAGGGTTTAGGGTTTAGGGTTTAGGGTTAGGGTTTTAGGGTTTAGGGTTAGGGTTTAGGGTTTAGGGTTTAGGGTTAGGGTTTAGGGTTTAGGGTTAGGGTTTAGGGTTAGGGTTAGGGTTTAGGGTTTAGGGTTAGGGTTTAGGGTTTAGGGTTTAGGGTTTAGGGTTTAGGGTTTAGGGTTTAGGGTTTAGGGTTAGGGTTTAGGGTTTAGGGTTTAGGGTTTAGGGTTTAGGGTTTAGGGTTTAGGGTTTAGGGTTTAGGGTTTAGGGTTTAGGGTTAGGGTTTAGGGTTTAGGGTTTAGGGTTAGGGTTTAGGGTTTAGGGTTTAGGGTTAGGGTTTAGGGTTTAGGGTTTAGGGTTTAGGGTTTAGGGTTTAGGGTTTAGGGTTTAGGGTTTAGGGTTTAGGGTTTAGGGTTTAGGGTTTAGGGTTTAGGGTTTAGGGTTTAGGGTTTAGGGTTTAGGGTTTAGGGTTTAGGGTTTAGGGTTTAGGGTTTAGGGTTTAGGGTTTAGGGTTTAGGGTTTAGGGTTTAGGGTTTAGGGTTTAGGGTTTAGGGTTTAGGGTTTAGGGTTTAGGGTTTAGGGTTTAGGGTTTAGGGTTTAGGGTTTAGGGTTTAGGGTTTAGGGTTTAGGGTTTAGGGTTTAGGGTTTAGGGTTTAGGGTTTAGGGTTTAGGGTTTAGGGTTTAGGGTTTAGGGTTTAGGGTTTAGGGTTTAGGGTTAGGGTTTAGGGTTTAGGGTTTAGGGTTTAGGGTTTAGGGTTTAGGGTTTAGGGTTTAGGGTTTAGGGTTTAGGGTTTAGGGTTTAGGGTTTAGGGTTTAGGGTTTAGGGTTTAGGGTTTAGGGTTTAGGGTTTAGGGTTAGGGTTTAGGGTTTAGGGTTTAGGGTTAGGGTTTAGGGTTTAGGGTTTAGGGTTTAGGGTTTAGGGTTTAGGGTTTAGGGTTTAGGGTTTAGGGTTTAGGGTTTAGGGTTTAGGGTTTAGGGTTTAGGGTTAGGTCTTAGGGTTTAGGGTTTAGGGTTTAGGGTTAGGGTTAGGGTTTAGGGTTAGGGTCTTAGGGCTTTAAGGGTTAGGGTTTAGGGTTAGGGTTTAGGGTTTCAGGGTTTAGCGGTTTAGGGTTTAGGGTTTAGGGTTAGGGTTTAGGGTTTAGGGTTTAGGGTTTAGGGTTAGGCGTTAGGGTTAGTTAGGGTTTAGGGTTAGGGTTAGGGTTTAGCGGTTTACAGGGTTTAGGGTTTAGGGTTTAGGGTTAGGGTTTAGGGTTTAGGGTTTAGGGTTAGGGTTTAGGGTTAGGGTTTAGGGTTAGGGTTAGGGTTAGGGTTAGGGTTTAGGGTTTAGGGTTTAGGGTTTAGGGTTAAGGGTTTAGGGTTTTAGGGTTTAGGGTTTAGGGTTTTAGGGTTTAGGGTTTTTAGGGTTAGGGTTTAGGGTTTAGGGTTTAGGGTTAGGGTTAGGGTTTAGGTTTAGGGTTTAGGGTTTAGGGTTTAGGGTTTAGGGTTTGGGTTAGGGTTTAGGGTTAGGGTTTAGGGTTTATGGGTTTAGGGTTTAGGGTTTAGGGTTTAGGCGTTAGGGTTTAGGGTTTAGGGTTTAGGGTTTAGGGTTTAGGGTTTAGGGTTAGGGTTTAGGGTTAGGGTTTAGGGTTTAGGGTTTAGGGTTTAGGGTTAGGGTTTAGGGTTTTAGGGTTTAGGGTTTAGGGTTTAGGGTTTAGGGTTAGGGTTTAGGGTTTAGGGTTTAGGGTTTAGGGTTTAGGTTTAGGGTTTAGGGTTTAGGGTTTAGGGTTTAGGGTTAGGGTTTAGGGTTTAGGGTTTAGGGTTTAGGGTTTAGGGTTTAGGGTTTAGGGTGTCTAGGGTTTAGGGTTAGGGTTAGGGTTTAGGGTTTAGGGTTTAGGGTTTAGGGTAGGGTTTAGGGTTCTAGGGGTTTAGGGTTTAGGGTTTAGGGTTTAGGGTTTAGGGTTTAGGGTTTAGGGTTTAGGGTTTAGGGTTTAGGGTTTAGGGTTTTTAGGGTTAGGGTTTAGGGTTTAGGGTTTAGGGTTAGGGTTTAGGGTTAGGGTTTAGGGTTAGGGTTTAGGGTTAGGGTTTAGGGTTAGGTTAGGGTTTAGGGTTTAGGGTTAGGGTTAGGGTTAGGGTTTAGGGTTTAGGGTTAGGGTTAGGCGTTAGGTTTAGGGTTTAGGGTTTAGGGTTAAGGCGTTTAGGGTTTAGGGTTTAGGGTTTAGGGTTAGGGTTAGGGTTTAGGGTTTAGGGTTTAGGGTTAGGGTTTAGGGTTTAGGGTTTAGGGTTTAGGGTTAGGTTTAGGGTTTAAGGGTTTAGGGTTTAGGGTTTAGGGTTAGGGTTTAGGGTTTAGGTTTAGGGTTTAGGGGTTTAGGGTTTAGGGTTTAGGGTTAGGGTTTAGGGTTTAGGGTTTAGGGTTTAGGGTTTAGGGTTTAGGTTAGGGTTAGGGTTTAGGGTTAGGGTTTAGGTAGGGTTTAGGGTTAGGGTTTAGGGTTAGGGTTTAGGGTTTAGGGTTTAGGGTTTAGGGTTTAGGGTTTAGGGTTTAGGGTTTAGGGTTAGGGTTTAGGGTTTAGGGTTAGGGTTTAGGGTTTAGGGTTTAGGGTTAGGGTTTAGGTTTAGGGTTTAGGTTAGGGTTTAGGGTTAGGGTTTAGGGTTAGGGTGGTAGGGTTTAGGGTTTAGGGTTAGGGTTTAGGGTTTAGGGTTTAGGGTTTAGGGTTTAGGGTTAGGGTTTAGGGTTTAGGGTTAGGGTAGGGTTTAGGGTTTAGGGTTAGGGTTTTAGGGTTTAGGGTTAGGGTTTAGGGTAGGGTTAGGGTTTAGGGTTAGGGTTAGGGTTAGGTTTAGGGTTAGGGTTTAGGGTTAGGGTTTAGGGTTTAGGGTTTAGGGTTTAGGGTTTAGGGTTTAGGGTTTAGGGTTAGGGTTTAGGGTTTAGGCTTTAGGTTAGGGTTTAGGGTTTAGGTTTAGGGTTTAGGGTTTAGGGTTTAGGTTAGGGTTTAGGGTTAGGGTTTAGGGTTTAGGGTTTAGGGTTTAGGGTTTAGGGTTTAGGGTTTAGGGTTGGGGTTTTAGGTTTAGGGTTAGGGTTTCGGGTTTAGGGTTTAGGGTTTAGGGTTAGGGTTTAGGGTTTAGGGTTTAGGGTTTAGGGTTTAGGGTTAGGGTTTAGGGTTAGGGTTTAGGGTTTAGGGTTTAGGGTTTAGGGTTTAGGGTTTAGGGTTTAGGGTTTAGGGTTTAGGGTTAGGGTTTAGGGTTTAGGGTTTAGGTTAGGGTTTAGGGTTAGGGTTTAGGGTTTAGGGTTTAGGGTTTAGGGTTTAGGGTTTAGGGTTTAGGGTTTAGGGTTAGGGTTTAGGGTTTAGGTTTAGGGTTTAGGGTTTAGGGTTAGGGTTAGGGTTTAGGGTTTAGGGTTTAGGTTTAGGGTTTAGGGTTTAGGGTTTAGGGTTTAGGGTTTAGGGTTTAGGGTTTAGGGTTTAGGGTTAGGGTTTAGGGTTTAGGGTTTAGGGTTAGGGGTTTAGGGTTTAGGGTTTAGGGTTTAGGGGTTAGGGTTTAGGGTTTAGGGTTTAGGGTTTAGGTTTAGGGTTTAGGGTTTAGGGTTTAGGGTTAGGTTTAGGGTTTAGGGTTAGGGTTTAGGGTTTAGGGTTTAGGGTTTAGGGTTTTAGGGTTTAGGGTTTAGGTTTAGGATTTAGGGTTTTAGGGTTTAGGGTTTAGGGTTTAGGGTTTAGGGTTTAGGGTTTAGGGTTTAGGGTTTAGGTTTAGGGTTTAGGGTTTAGGGTTTAGGGTTTAGGGTTTAGGGTTTAGGGTTTAGGGTTTAGGGTTTAGGGTTTAGGGTTTAGGGTTAGGGTTTAGGGTTTAGGGTTTAGGTTTAGGGTTTAGGGTTTAGGGTTTAGGGTTTAGGGTTTAGGGTTTAGGGTTTTAGGGTTTAGGGTTTAGGGTTTAGGGTTTAGGGTTAGGTTAGGGTTTAGGGTTTAGGGTTTAGGGTTTAGGGTTTAGGGTTTAGGGTTAGGGTTTAGGGTTTAGGTTTAGGGTTTTAGGGTTTAGGGTTTAGGGTTTAGGGTTTAGGGTTTAGGGTTTAGGGTTTAGGGTTTAGGGTTTAGGGTTTAGGGTTTAGGGTTTAGGGTCTTGGGTTTAGGGTTTAGGGTTTAGGGTTAGGGTTAGGGTTTAGGGTTTAGGGTTATGGTTGGGTTTAGGGTTTAGGGTTTAGGGTTTAGGGTTTAGGGTTAGGGTTTAGGGTTTAGGGTTTAGGGTTTAGGGTTAGGGTTAGGGTTTAGGGTTTTAGGGTTTAGGGTTAGGGTTTAGGGTTAGGGTTTAGGGTTTAGGGTTATTTTTTAGGGTTTAGGGTTTAGGGTTTAGGGTTTAGGGTTTAGGGTTTAGGGTTTAGGGTTAGGGTTTAGGGTTTAGGGTTTAGGGTTTAGGGTTAGGTTAGGGTTTAGGGTTAGGGTTTAGGGTTTAGGGTTTAGGGTTTAGGGTTTAGGGGTTAGGGTTTAGGGGTTTAGGGTTTAGGGTTTAGGGTTTAGGGTTTTAGGGTTTTAGGGGTTAGGGTTTAGGGTTTTAGGGTTAGGTTAGGTAGGGTTTAGGGTTTAGGGTTTAGGGTTTAGGGTTTAGGGTTTAGGGTTTAGGGTTTAGGGTTTAGGGTTTAGGGTTTAGGGTTTAGGGTTTAGGGTTTAGGTTTAAGGGTTTAGGGTTTAGGGTTTAGGGTTTAGGGTTTAGGGTTTAGGGTTTAGGGTTAGGGTTTAGGGTTTAGGGTTTAGGGTTTAGGGTTTAGGGTTTAGGTTAG

**2DL**

TTGGGTTTATGG**TTTAGGG**TTTAGGGTTAGGGTTTATGGATTAGGGTTTAGGGTTAGGGTTTATGGTTTAGGGTTTAGGGTTTAGGGTTAGGGTTTAGGGTTTAGGGTTTAGGGTTTAGGGTTTAGGGTTTAGGGTTTAGGGTTTAGGGTTAGGGTTTTAGGGTTTAGGGTTTAGGGTTTAGGGTTTAGGGTTTAGGGTTTAGGGTTTAGGGTTTAGGGTTTAGGGTTTAGGGTTTAGGGTTAGGGTTAGGGTTTAGGGTTTAGGGTTAGGGTTTAGGGGTTTAGGGTTTAGGGTTAGGGTTTAGGGTTAGGGTTTAGGGTTTAGGGTTTAGGGTTTAGGGTTTAGGGTTTAGGGTTTAGGGTTTAGGGTTAGGGTTAGGGTTTAGGGTTAGGGTTAGGGTTTAGGGTTTAGGGTTTAGGGTTTAGGGTTTAGGGTTTAGGGTTTAGGGTTTAGGGTTAGGGTTAGGGTTTAGGGTTTAGGGTTTAGGGTTTAGGGTTAGGGTTTAGGGTTTAGGGTTTAGGGTTTAGGGTTTAGGGTTTAGGGTTTAGGGTTTAGGGTTTAGGGTTTAGGGTTTAGGGTTTAGGGTTTAGGGTTTAGGGTTTAGGGTTTTTAGGGTTTTTTGGGTTTAGGGTTTAGGGTTTTTAGGGTTAGGGTTTAGGGTTTAGGGTTTAGGGTTTAGGGTTTAGGGTTTAGGGTTTAGGGTTTAGGGTTTAGGGTTTAGGGTTTAGGGTTTAGGGTTTAGGGTTTAGGGTTTAGGGTTTAGGGTTTAGGGTTTAGGGTTTAGGGTTTAGGGTTTAGGGTTTAGGGTTTAGGGTTTAGGGTTTAGGGTTTAGGGTTTTAGGGTTTTTAGGGTTTAGGGTTTAGGGTTTAGGGTTTTAGGGTTTTAGGGTTTTAGGGTTTAGGGTTTTAGGGTTTAGGGTTTAGGGTTTAGGGTTTAGGGTTTCGGGTTTAGGGTTTAGGGTTTAGGGTTTAGGGTTTAGGGTTTAGGGTTAGGGTTTTTAGGGTTTAGGGTTTAGGGTTTAGGGTTTAGGGTTTAGGGTTTAGGGTTTAGGGTTTAGGGTTTAGGGTTTAGGGTTAGGGTTTAGGGTTAGGGTTTAGGGTTTAGGGTTTAGGGGTTTAGGGTTTAGGGTTTAGGGTTTAGGGTTTAGGGTTTAGGGTTTAGGGTTTAGGGTTTAGGGTTTAGGGTTTAGGGTTTAGGGTTTAGGGTTTAGGGTTTAGGGTTTAGGGTTTAGGGTTTAGGGTTTAGGGTTTAGGGTTTAGGGTTTAGGGTTTAGGGTTTAGGGTTTAGGGT

**3DL**

GAGGTGGG**TTTAGGG**TTTAGGGTTAGGGTTTAGGGTTGTTAGGGTTTAGGGTTAGGGTTGGGTTAGGGTTTAGGGTTTAGGGTTTAGGGTTTAGGGTTTAGGGTTTAGGGTTTAGGGTTTAGGGTTAGGGTTTAGGGTTTAGGGTTTAGGGTTAGGGTTTAGGGTTTAGGGTTTAGGGTTTAGGGTTTAGGGTTTAGGGTTTAGGGTTAGGGTTTAGGGTTAGGGTTTAGGGTTTAGGGTTTAGGGTTAGGGTTTAGGGTTAGGGTTTAGGGTTTAGGGTTAGGGTTTAGGGTTTAGGGTTTAGGGTTTAGGGTTTAGGGTTTAGGGTTAGGGTTTAGGGTTAGGGTTTAGGGTTTAGGGTTTAGGGTTAGGGTTTAGGGTTTAGGGTTTAGGGTTTAGGGTTTAGGGTTGGTTTAGGGTTTAGGGTTTAGGGTTGGTTTAGGGTTGGTTTAGGGTTTAGGGTTTAGGGTTGGTTTAGGGTTTAGGGTTTAGGGTTTAGGGTTTAGGGTTTAGGGTTTAGGGTTTAGGGTTTAGGGTTTAGGGTTGTTAGGGTTTAGGGTTTAGGGTTTAGGGTTTAGGGTTTAGGGTTTAGGGTTGGGGTTTAGGGTTTAGGGTTAGGGTTTAGGGTTTAGGGTTTAGGTTTAGGGTTGGGTTGGGTTTAGGGTTTAGGGTTTAGGGTTTAGGGTTTAGGGTTTAGGGTTTAGGGTTTAGGGTTTAGGGTTTAGGGTTTAGGGTTTAGGGTTTAGGGTTTAGGGTTTAGGGTTTAGGGTTTAGGGTTTAGGGTTTAGGGTTTAGGGTTTAGGGTTTAGGGTTTAGGGTTTAGGGTTTAGGGTTTAGGGTTTAGGGTTTAGGGTTTAGGGTTTAGGGTTTAGGGTTTAGGGTTTAGGGTTTAGGGTTTAGGGTTTAGGGTTTAGGGTTTAGGGTTTAGGGTTTAGGGTTAGGGTTTAGGGTTTAGGGTTTAGGGTTTAGGGTTTAGGGTTTAGGGTTTAGGGTTTAGGGTTTAGGGTTTAGGGTTTAGGGTTAGGGTTTAGGGTTTAGGGTTTAGGGTTTAGGGTTTAGGGTTTAGGGTTTAGGGTTTAGGGTTTAGGGTTTAGGGTTTAGGGTTTAGGGTTAGGGTTTAGGGTTTGGGTTTAGGGTTTAGGGTTAGGGTTTAGGGTTTAGGGTTTAGGGTTTAGGGTTTAGGGTTTAGGGTTTAGGGTTTAGGGTTTAGGGTTTAGGGTTTAGGGTTTAGGGTTTAGGGTTTAGGGTTTAGGGTTAGGGTTTAGGGTTTAGGGTTTAGGGTTTAGGGTTTAGGGTTTCGGGTTTAGGGTTTAGGGTTTAGGGTTTAGGGTTTAGGGTTTAGGGTTTAGGGTTAGGGTTTAGGGTTTAGGGTTTAGGGTTTTTAGGGTTTAGGGTTAGGGTTTAGGGTAGGGTTAGGGTTAGGGTTTAGGGTTTAGGGTTAGGGTTAGGGTTTAGGGTTTAGGGTTAGGGTTTAGGGTTAGGGTTAGGGTTACGGTTTAGGGTTTAGGGTTTAGGGTTTAGGGTTTAGGGTTTAGGGTTTAGGGTTTAGGGTTTAGGGTTAGGGTTAGGGTTAGGGTTAGGGTTTAGGGTTTAGGGTTTAGGGTTTAGGGTTTAGGGTTGTTGGGTTTAGGGTTAGGGGTTTAGGGTTTAGGGTTTAGGGTTTAGGGTTTAGGGTTTAGGGTTTAGGGTTTAGGGTTTAGGGTTAGGGTTTAGGGTTTAGGGTTTTTTAGGGTTTAGGGTTTAGGGTTTAGGGTTAGGGTTTAGGGTTTAGGGTTTAGGGTTTAGGGTTTAGGGGTTTAGGGTTTAGGGTTTAGGGTTTAGGGTTTAGGGTTTAGGGTTTAGGGTTTAGGGTTTAGGGTTAGGGTTTAGGGTTTAGGGTTTAAGGGTTTAGGGTTTAGGGTTTAGGGTTAGGGTTTAGGGTTAGGGTTTGTTAGGGTTTAGGGTTTAGGGTTTAGGGTTAGGGTTTAGGGTTTAGGGTTTTAGGGTTTAGGGTTTAGGGTTTAGGGTTTAGGGTTTAGGGTTTAGGGTTAGGGTTAGGGTTTAGGGTTTAGGGTTTAGGGGTTAGGGTTTAGGGTTTAGGGTTTAGGGTTTAGGTAGGGTTTAGGGTTTAGGGTTTAGGGTTTGGTAGGGTTTAGGGTTAGGGTTAGGGTTTAGGGTTTAGGGTTTAGGGTTAGGGTAGGGTTTAGGGTTTAGGGGTTAGGGTTTAGGGTTTAGGGTTTAGGGTTTTAGGGTTTAGGGTTTAGGGTTTAGGGTTTAGGGTTTAGTGGTTTAGGGTTGGGTTAGGGTTTAGGGTTTAGGGTTAGGGTTTAGGGTTTAGGGTTTAGGGTTTAGGGTTTAGGGTTTAGGGTTTGGGTTTAGGGTTTAGGGTTTAGGTGGGTTTAGGGTTTAGGGTTTAGGGTTTAGGGTTTTGGGTTTAGGGTTTAGGGTTTAGGGTTAGGGTTTAGGGTTGGTTAGGGTTTGGTTAGGGTTTAGGGTTTAGGGTTTAGGGTTTAGGGTTTAGGGTTTAGGGTTAGGGTTTAGGGTTTAGGGTTTAGGGTTTAGGGTTTAGGGTTTAGGGTTTTGGGTTTAGGGTTTAGGGTTTAGGGTTTAGGGTTTAGGGTTTAGGGTTTAGGGGTTTAGGGTTTAGGGTTTAGGGTTTAGGGTTTAGGGTTTAGGGTTTAGGGTTTAGGGTTTAGGGTTTAGGGTTTAGGGTTTAGGGTTTAGGGTTTAGGGTTAGGGTTTAGGGTTTTGGGTTTAGGGTTTAGGGTTTAGGGTTTAGGGTTTAGGGTTTAGGGTTTAGGGTATAGGGTTTAGGGTTTAGGGTTTAGGGTTTAGGGTTTAGGGTTAGGGTTTAGGGTTTAGGGTTTAGGGTTTAGGGTTTAGGGTTTAGGGTTTAGGGTTTAGGGTTTAGGGTTAGGGTTTAGGGTTTAGGGTTTAGGGTTAGGGTTTTAGGGGTTTAGGGTTTTAGGGTTTTAGGGTTTAGGGTTTAGGGTTTAGGGTTTAGGTTTTAGGGTTTAGGGTTTAGGGTTTAGGGTTTAGGGTTTAGGGGTTTAGGGTTTAGGGTTTAGGGTTTAGGGTTTAGGGTTTTAGGGTAGGGTTTAGGGTTTAGGGTTTAGGGTTTAGGGTTTAGGGTTTAGGGTTTAGGGTTTAGGGTTTAGGGTTTAGGGTTTAGGGTTTAGGGTTTAGGGTTTAGGGTTTAGGGTTTAGGGTTTAGGGTTTAGGGTTAGGGTTTAGGGTTTAGGGTTTAGGGTTTAGGGTTTAGGGTTTAGGGTTAGGGTTTAGGGTTTAGGGTTTAGGGTTTAGGGTTTAGGGTTTAGGGTTTAGGGTTTAGGGTTTAGGGTTTAGGGTTGGGTTTAGGGTTAGGGTTTAGGGTTTAGGGTTTAGGGTTTAGGGTTTAGGGTTTAGGGTTTAGGGTTTAGGGTTTAGGGTTTAGGGTTTAGGGTTTAGGGTTTAGGGTTTAGGGTTTAGGGTTTAGGGGTTAGGGTTTAGGGTTTAGGGTTTAGGGTTTAGGGTTTAGGGTTAGGGTTTAGGGTTAGGGTTTAGGGTTTAGGGTTTAGGGTTTAGGGTTTGGGTTTAGGGTTTAGGGTTTAGGGTTTAGGGTTTAGGGTTTAGGGTTAGGGTTTAGGGTTTAGGGTTTAGGGTTTAGGGTTTAGGGTTTAGGGTTAGGGTTTAGGGTTTAGGGTTTAGGGTTTAGGGTTTAGGGTTAGGGTTTAGGGTTAGGTTTAGGGTTTAGGGTTTAGGGTTTAGGTTAGGGTTTAGGGTTAGGTTAGGGTTTAGGGTTTAGGGTTTAGGGTTTAGGGTTTAGGGTTTAGGGTTTAGGGTTAGGGTTTAGGGTTTAGGGTTTAGGGTTAGGGTTTAGGGTTAGGGTTTAGGGTTTAGGGTTTAGGGTTAGGGGTTTAGGGTTTAGGGTTTAGGGTTTAGGGTTTAGGGTTTAGGGTTAGGGTTTAGGGTTTAGGGTTTAGGGTTTAGGGTTTAGGGTTTAGGGTTTAGGGTGTTAGGGTTTAGGGTTTAGGGTTTAGGGTTTAGGGTTTAGGGTTTTGGGTTAGGGTTTAGGGTTTGGGTTTAGGGTTAGGGTTTAGGGTTTAGGGTTTAGGGTTAGGGTTTAGGGTTAGGGTTTAGGGTTTAGGGTTAGGGTTTAGGGTTTAGGGTTTAGGGTTTAGGGTTTAGGGTTTAGGGTTTAGGGTTAGGGTTTAGGGTTTAGGGTTTAGGTTTAGGGTTTAGGGTTTAGGGTTTGGGTTTAGGGTTTAGGGTTAGGGTTTAGGGTTAGGGTTTAGGGTTTAGGGTTTAGGGTTAGGTTTAGGGTTTAGGGTTTAGGGTTAGGGTTAGGGTTTGGTTAGGGTTTAGGGTTTAGGTTTAGGGTTTAGGGTTTAGGTTTAGGGTTTAGGGTTTAGGGTAGGTTAGGGTTTAGGGGGTTAGGGTTTAGGGGTTAGGGTTAGGGTTTAGGGTTTAGGGTTTAGGGTTAGGGTTTAGGGTTTAGGGTTTAGGGTTTTGGGTTTAGGTTGTTTTGGGTTAGGGTTTAGGGTTTAGGTTTGGGGTTTAGGGTTAGTTAGGGTTTAGGTTGGTTTAGGGTTAGGGTTTAGGGTTTAGGGTTTAGGGTTAGGGTTTAGGGTTTAGGGTTTAGGGTTTAGGGTTTAGGGTTTAGGGTTTAGGGTTTAGGGTTTAGGGTTTAGGGTTTAGGGTTTAGGGTTTAGGGTTTAGGGTTTAGGGTTTAGGGTTTAGGGTTTAGGGTTAGGGTTTAGGTTTAGGGTTAGGGTTTAGGGTTTAGGGTTTAGGGTGGTTGGGTTTAGGGTTTAGGGTTTAGGGTTTAGGGTTTAGGGTTTAGGGTTTAGGGTTTAGGGTTTAGGGTTTAGGTTAGGGTTTAGGGTTTAGGGTTTAGGGTTAGGGTTTAGGGTTTAGGGTTAGGGTTTAGGTTAGGGTTTAGGGTTTAGGGTTTAGGGTTTAGGGTTTAGGGTTTAGGGTTAGGGTTAGGGTTTGGTTAGGGTTTAGGGGGTTTAAGGTTTAGGGTTTAGGGTTTAGGGTTTAGGGTTTAGGGTTTAGGGTTTAGGGTTTAGGGTTTAGGGTTTAGGGTTTAGGGTTTAGGGTTAGGGTTTAGGGTTTAGGGTTTAGGTTTAGGGTTTAGGGTTTAGGGTTTAGGGTTTAGGGTTAGGGTTTAGGGTTTAGGGTTTAGGGTTTAGGGTTTAGGGTTTAGGGTTTAGGGTTTAGGGTTTAGGGTTTAGGGTTTAGGGTTAGGGTTTAGGGTTTAGGGTTTAGGGGTTTAGGGTTTAGGGTTTAGGGTTTAGGGTTTAGGGTTAGGGTTTAGGGTTTAGGGTTTAGGGTTTAGGGTTTAGGGTTTAGGGTTTAGGGTTTAGGGTTAGGGTTTAGGGTTTAGGGTTTAGGGTTTAGGGTTTAGGGTTTAGGGGTTTAGGGTTTAGGGTTAGGGTTTAGGGTTTAGGGTTTAGGGTTTAGGGTTTAGGGTTTAGGGTTTAGGGTTTAGGGTTAGGGTTTAGGGTTAGGGTTTAGGGTTTAGGGTTTAGGGTTTAGGGTTTAGGGTTTAGGGGTTTAGGGTTTAGGGTTAGGGTTTAGGGTTTAGGGTTTAGGGTTTAGGGTTTAGGGTTTAGGGTTTAGGGTTTAGGGTTTAGGGTTAGGGTTTAGGGTTTAGGGTTTAGGGTTAGGGTTTAGGGTTTAGGGTTTAGGGTTTAGGGTTAGGGTTTAGGGTTTAGGGTTTAGGGTTTTAGGGTTTAGGTGGTGTAGGTTAGGGTTTTAGGGTTTAGGGTTTTAGGGTTTAGGGTTTAGGGTGGTAGGGTTAGGGTTTAGGGTTTAGGGTGGTTTAGGGTTTAGGGTTTAGGGTTTAGGGTTTAGGGTTTAGGGTTAGGGTTTAGGGTTTAGGGTTAGGGTTTAGGGTTTAGGGTTTAGGGTTAGGGTTTAGGGTTTAGGGTTTAGGGTTAGGGTTTAGGGTTTAGGGTTTAGGGTTTAGGGTTTAGGGTTTAGGGTTTAGGGTTTAGGGTTTAGGGTTTAGGGTTTAGGGTTTTAGGGTTTAGGGGTTTAGGGTTTTAGGGTTTTAGGGTTTAGGGTTTAGGGTTTAGGGTTTAGGGTTTAGGGTTTAGGGTTTAGGGGTTTAGGGTTTAGGGTTTAGGGTTTAGGGTTTTAGGGTTTAGGGTTTAGGGTTTAGGGTGGTTAGGGTTTAGGGTTTAGGGTTTAGGGTTAGGGTTTAGGGTTAGGTTTAGGGTTTAGGGTTTAGGGTTTAGGGTTTAGGGTTTAGGGTTTAGGGTTTAGGGTTTAGGGTTAGGGTTTAGGGTTTAGGGTTAGGGTTTAGGGTTTAGGGTTTAGGGTTTAGGGTTTAGGGTTTAGGGTTTAGGGTTTAGGGTTTAGGGGTTTAGGGTTTAGGGTTAGGGTTAGGGTTTAGGGTTTTAGGGGTTTAGGGTTTAGGGTTTAGGGTTTAGGGTTTAGGGTTTAGGGGTTTAGGGTTTAGGGTTTAGGGTTTAGGGTTAGGGTTTAGGGTTAGGGTTTAGGGTTAGGGTTTAGGGTTTAGGGTTTAGGGTTTAGGGGTTTAGGGTTTAGGGTTTAGGGTTAGGGTTTAGGGTTTAGGGTTTAGGGTTTAGGGTTTAGGGTTTAGGTTAGGGTTTAGGGTTTAGGGTTTAGGGTTAGGGTTTAGGGTTTAGGGTTTAGGGTTTAGGGTTTAGGGTTAGGGTTTAGGGGTTTAGGGTTTAGGGTTTAGGGTTTGGGTTTAGGGTTTAGGGTTTAGGGTTTAGGGTTTAGGGTTTAGGGTTTAGGGTTTAGGGTTTAGGGTTTAGGGTTTAGGGTTTAGGGTTTAGGGTTTAGGGTTTAGGGTTAGGGTTTAGGGTTTAGGGTTTAGGGTTTAGGGTTTAGGGTTTAGGGTTTAGGGTTTAGGGTTAGGGTTTAGGGTTTTAGGGTTTAGGGTTTAGGGTTTAGGGTTTAGGGTTTAGGGTTTAGGGTTTAGGGTTTAGGGTTTAGGGTTTAGGGTTTTAGGGTTTAGGGTTTAGGGGTTTAGGGTTTAGGGTTTAGGGTTTAGGGTTAGGGTTTAGGGTTTAGGGTTTAGGGTTTAGGGTTTAGGGTTTAGGGTTAGGGTTTAGGGTTTAGGGTTTAGGGTTTAGGGTTTAGGGTTTAGGGTTAGGGTTTAGGGTTTAGGGTTTAGGGTTTAGGGTTAGGGTTTAGGGTTTAGGGTTTAGGGTTTAGGGTTTAGGGTTTAGGGTTTAGGGTTTAGGGTTTAGGGTTTAGGGTTAGGGTTTAGGGTTTAGGGTTTAGGGTTAGGGTTTAGGGTTTAGGGTTAGGGTTTAGGGTTTAGGGTTTAGGGGTTTAGGGTTTAGGGTTTAGGGTTTAGGGTTTAGGGGTTTAGGGTTTAGGGTTTAGGGTTTAGGGTTTAGGGTTTAGGGTTTAGGGTTAGGGTTTAGGGTTTAGGGTTAGGGTTTAGGGTTTAGGGTTTAGGGTTAGGGTTTAGGGTTTAGGGTTTAGGGTTTAGGGTTAGGGTTTAGGGTTTAGGGTTAGGGTTTAGGGTTTAGGGTTTAGGGTTTAGGGTTTAGGGTTTAGGGTTTAGGGTTTAGGGTTTAGGGTTTAGGGTTTAGGGTTTTGGGTTTAGGGTTTAGGGTTTAGGGTTTAGGGTTTAGGGTTTAGGGTTTAGGGTTTAGGGTTAGGGTTTAGGGTTTAGGGTTTAGGGTTTAGGGTTTAGGGTTTAGGGTTTAGGGTTTAGGGTTTAGGGTTTAGGGTTAGGGTTTAGGGTTTAGGGTTAGGGTTTAGGGTTTAGGGTTTAGGGTTTAGGGTTTAGGGTTTAGGGTTTAGGGTTTAGGGTTTAGGGTTTAGGGTTTAGGGTTTAGGGTTTAGGGTTTAGGGTTAGGGTTAGGGTTTTGGGTTAGGGTTTAGGGTTTTAGGGGTTAGGGTTTAGGGTTTAGGGTTTAGGGTTTAGGGTTAGGGTTAGGGTTAGGGTTTAGGGTTTAGGGTTTAGGGTTTAGGGTTTAGGGTTTAGGGTTTGGGTTTAGGGTTTAGGGTTTAGGGTTTAGGGTTTAGGGTTTAGGGTTTAGGGTTAGGGTTTAGGGTTTAGGGTTTAGGGTTTAGGGTTAGGTTTAGGGTTTAGGGTTTAGGGTTTAGGGGTTTAGGGTTTAGGGTTAGGGTTTAGGGTTTAGGGTTTAGGGTTTAGGGTTTAGGGTTTAGGGTTTAGGGTTTAGGGTTTAGGGTTTAGGGTTTAGGGTTTAGGGTTTAGGGTTTAGGGTTTAGGGTTTAGGGTTTAGGGTTAGGGTTTAGGGTTTAGGGTTTAGGGTTTAGGGTTTAGGTTTAGGGTTAGGGTTTAGGGTTTAGGGTTTAGGGTTTAGGGTTTAGGGTTTAGGGTTAGGGTTTAGGGTTTAGGGTTTAGGGTTTAGGGTTTAGGGTTAGGGTTTAGGGTTAGGGTTTAGGGTTTAGGGTTTAGGGTTTAGGGTTTAGGGTTTAGGGTTTAGGGTTTAGGGTTTAGGGTTTAGGGTTTAGGGTTTAGGGTTTAGGGTTTAGGGTTTAGGGTTTAGGGTTTAGGGTTAGGGTTAGGGTTTAGGGTTTAGGGTTAGGGTTTAGGGTTTAGGGTTTAGGGTTAGGGTTAGGGTTTAGGGTTTAGGGTTTAGGGTTTAGGGTTTAGGGTTTAGGGTTTAGGGTTTAGGGTTTAGGGTTTAGGGTTTAGGGTTTAGGGTTTAGGGTTTAGGGTTAGGGTTTAGGGTTTAGGGGTTTAGGGTTTAGGGTTTAGGGTTTAGGGTTAGGGTTTAGGGTTTAGGGTTTAGGGTTAGGGTTAGGGTTTAGGGTTTAGGGTTTAGGGTTTAGGGTTTAGGGTTAGGGTTTAGGGTTAGGGTTTAGGGTTTAGGGTTTAGGGTTTAGGGTTTAGGGTTTAGGGTTAGGGTTTAGGGTTTAGGGTTTAGGGGTTTAGGGTTTAGGGTTTAGGGTTTAGGGTTAGGGTTTAGGTTTAGGGTTTAGGGTTTAGGGTTTAGGGTTTAGGGTTTAGGGTTTAGGGTTTGGGTTTAGGGTTAGGTTAGGGTTAGGGTTTAGGGTTTAGGGTTTAGGGTTTAGGGTTTAGGGGTTTTAGGGTTTAGGGTTAGGGTTTAGGGTTTAGGGTTTAGGGTTAGGGTTTAGGGTTTAGGGTTTAGGGTTTAGGGTTTAGAGGGTTTAGGGTTTAGGGTTTAGGGTTTAGGGTTTAGGTTAGGTTTAGGGTAGGGTTTAGGGTTTTGGGTTTAGGGTTTAGGGTTTAGGGTTTAGGGTTTAGGGTTTAGGGTTAGGGTTTAGGGTTTAGGGTTTAGGGTTTAGGGTTTAGGGTTAGGGTTTAGGGTTTAGGGTTAGGGTTTAGGGTTTAGGGTTTAGGGTTTTGGGTTTAGGGTTTAGGGTTTAGGGTTAGGGTTTAGGGTTTAGGGTTTAGGTTTAGGGTTTAGGGTTTAGGGGTTAGGGTAGGGTTAGGGTTTGGGTTTAGGGTTAGGGTTTAGGGTAGGTTTAGGGTTTAGGGTTTAGGGTTTAGGGTTTAGGGTTTAGGGTTTAGGGTTTAGGGTTTAGGTTAGGGTTTAGGGTTTAGGGTTTAGGGTTTAGGGTTTAGGGTTTAGGGTTAGGGTTTAGGGTAGGGTTTAGGGTTTAGGGTTTAGGGTTTAGGGTTTAGGGTTTAGGGTTTAGGGTTAGGTTGGGTTAGGGTTTAGGGTTTAGGGTTTAGGGTTTAGGGTTTAGGGTTTAGGTTGGTTTAGGGTTTGGTTTAGGGTTTAGGGTTTAGGTTTTGGTAGGTTTAGGGTTTAGGGTTTAGGGTTTAGGGTTTAGGGTTTAGGGTTTAGGGTTTAGGGTTAGGGTTTAGGGTTTAGGGTTTTGGGTTTAGGGTTTAGGGTTAGGGTTTAGGGTTTAGGGTTTAGGGTTTAGGGTTTAGGGTTAGGGTTTAGGGTTTAGGGTTTAGGGTTTAGGGTTTGGTTAGGGTTTAGGGTTTAGGGTTTGGTTTAGGGTTTAGGGTTTAGGGTTTAGGGTTTAGGGTTTAGGGTTTAGGGTTTAGGGTTTAGGGTTTAGGGGTTAGGGTTAGGGTTTAGGGTTTAGGGTTAGGGTTTAGGGTTTAGGGTTTAGGGTTTAGGGTTAGGGTTTAGGGTTTAGGGTTTAGGGTTAGGTTAGGGTTTAGGGTTTAGGGTTAGGGTTAGGGTTTAGGGTTAGGTTAGGGTTTAGGGTTAGGGTTTAGGGTTTAGGGTTTAGGGTTTAGGGTTTAGGGTTTAGGGTTTAGGGTTTAGGGTTTAGGGTTTAGGGTTTAGGGTTTAGGGTTTAGGGTTTAGGGTTAGGGTTTAGGGTTAGGTTAGGTTTAGGGTTTAGGGTTAGGGTTTAGGGTTTAAGGGTTTAGGTTTAGGGTTTTAGGGTTAGGGTTTAGGGTTTAGGGTTTAGGGTTTAGGTTAGGGTTTAGGGTTTAGGGTTTAGGGTTTAGGTTTAGGGTTTAGGGTTTAGGGTTTAGGGTTTAGGGTTTAGGGTTTAGGGTTAGGGTTTAGGGTTAGGGTTTAGGGTTTAGGGTTTAGGGTTTAGGGTTTAGGGTTTAGGGTTTAGGGTTTAGGGTTTAGGGTTAGGGTTTAGGGTTTAGGTTTAGGGTTTAGGGTTTAGGGTTTAGGGTTTAGGGTTTAAGGGTTTAGGTGGGTTTAGGGTTTAGGGTTTAGGGTTTAGGGTTTAGGGTTTAGGGTTTGGTAAGGGTTTAGGGTTTAGGGTTTAGGGTTTAGGGTTTAGGGTTTAGGGGTTTAGGGTTTAGGGTTTAGGGTTTAGGGTTTTAGGGTTTAGGGTTTAGGGTTTAGGGTTTAGGGTTTAGGGTTTAGGGTTTTTAGGGTTTAGGGTTTAGGGTTTAGGGTTTAGGGTTTAGGGTGGTTTAGGGTTTAGGGTTTAGGGTTTAGGGTTTAGGGTTTTAGGGTTAGGGTTTAGGGTTTAGGGTTTAGGGTTTAGGGTTTAGGGTTTTGGTTTAGGGTTAGGGTTTAGGGTTTAGGGTTAGGGTTTAGGGTTTAGGTTTAGGGTTTAGGGTTTAGGGTTAGGGTTTAGGGTTTAGGGTTAGGGTTTAGGGTTTAGGGTTTAGGTTGTTAGGGTTAGGGTTAGGGTTTAGGGTTTAGGGGTTAGGGTTTAGGGTTTAGGGTTTAGGGTTTAGGGTTTAGGGTTTAGGGTTTAGGGTTTAGGGTTTAGGGTTTAGGGTTTAGGGTTTAGGGTTTAGGGTTTAGGTTTAGGGTTTAGGGTTTAGGGTTTAGGGTTTAGGGTTTAGGGTTTAGGGTTTAGGGTTTAGGGTTAGGGTTTAGGTTTAGGGTTTAGGGTTTAGGGTTAGGGTTAGGGGTTTAGGGTTTAGGTTTAGGGTTTAGGGTTAGGTTAGGGTTTAGGGTTTAGGGTTTAGGGGTTTAGGGTTTAGGGTTTAGGGTTTAGGGTTTAGGGTTTAGGTTTAGGGTTTAGGGTTTAGGGGTTAGGGTTTAGGGTTTAGGTTTAGGGTTTAGGGTTTAGGGTTTAGGGTTTAGGGTTTAGGGTTAGGGTTTAGGGGTTTAGGGTTTAGGGTTTAGGGTTTAGGGTTTAGGGTTTTGGGGTTTAGGGTTTAGGGTTTAGGGTTTAGGGTTTAGGGTTTAGGGTTTAGGGTTTAGGGTTTAGGGTTTAGGGTTTGGGTTAGGGTTTAGGGTTTAGGGTTTAGGGTTTAGGGTTTTAGGGTTTAGGGTTGGTTAGGGTTTAGGGTTTAGGGTTTAGGGTTTTGGGTTTAGGGTTTAGGGTTTAGGTTTAGGGTTAGGGTTTAGGGTTAGGGTTTAGGGTGGTTAGGGTTTAGGGTTTAGGGTTTAGGGTTTAGGGTTTAGGGTTTAGGGTTTTGGGTTTAGGGTTTAGGGTTTAGGGTTTAGGGTTTAGGGTTTAGGGTTTAGGGTTTAGGGTTTAGGGTTAGGGTTTAGGGTTTAGGGTTTAGGGTTTAGGGTTTAGGGTGGTTAGGGTTTAGGGGTTTAGGGTTTAGGGTTTAGGGTTTAGGTTTTAGGGTTTAGGGTTTTAGGGTTTAGGGTTAGGGTTTAGGGATTTAGGGTTTAGGGTTTAGGGTTTAGGTTTAGGGTTTAGGGTTTAGGGTTTAGGGTTTAGGGTTTAGGTTAGGGTTTAGGGTTTAGGGTTTAGGGTTTAGGGTTTAGGGTTTAGGGTTAGGGTTTAGGGTTTAGGGTTTAGGGTTTTAGGGGTTTAGGGTTTAGGGTTTAGGTTTAGGGTTTAGGGTTAGGGTTTAGGGTTGTTAGGGTTTAGGGTTTAGGGTTAGGGTTTAGGGTTTAGGGTTTAGGGTTTAGGTTTAGGGTTTAGGGTTTAGGGTTTAGGGTTTTAGGGTTTAGGGTTTAGGGTTTAGGGTTTAGGGTTTAGGGTTTAGGGTTTAGGGTTTAGGGTTAGGGTTTAGGGTTTAGGGTTTAGGGTTTAGGGTTTAGGGTT

**5DL**

GGTTAGGG**TTTAGGG**TTTAGGGTTAGGGTTTAGGGTTTAGGGTTTAGGGTTTAGGGTTTAGGGTTTAGGGTTTAGGGTTTAGGGTTTAGGGTTTAGGGTTAGGGTTTAGGGTTTAGGGTTTAGGGTTTAGGGTTTAGGGTTTAGGGTTAGGGTTAGGGTTTAGGCTTTAGGGTTTAGGGTTAGGGTTAGGGTTTAGGGTTAGGGTTTAGGGTTAGGGTTTAGGTTTAGGGTTTAGGGTTTAGGGTTGGGTTTAGGGTTTAGGGTTTAGGGTTTAGGGTTAGGGTTTAGGGTTAGGGTTTAGGGTTTAGGGTTTAGGGTTTAGGGTTTAGGGTTAGGGTTTAGGGTTTAGGGTTTAGGGTTTAGGGTTTAGGGTTTAGGGTTTAGGGTTTAGGGTTAGGGTTTAGGGTTTAGGGTTTAGGGTTTAGGGTTTAGGGTTTAGGGTTTAGGGTTTAGGGTTTAGGGTTTAGGGTTTAGGGTTTAGGGTTAGGGTTTAGGGTTTAGGGTTTAGGGTTTAGGGTTTAGGGTTTAGGGTTTAGGGTTTAGGGTTTAGGGTTTAGGGTTTAGGGTTTAGGGTTTAGGGTTTAGGGTTTAGGGTTTAGGGTTTAGGGTTTAGGGTTTAGGGTTTAGGGTTTAGGGTTTAGGGTTTAGGGTTTAGGGTTTAGGGTTTAGGGTTTAGGGTTAGGGTTTAGGGTTTAGGGTTTAGGGTTTAGGGTTTAGGGTTTAGGGTTTAGGGTTTAGGGTTTAGGGTTAGGGTTTAGGGTTTAGGGTTAGGGTTTAGGGTTTAGGGTTTAGGGTTTAGGGTTTAGGGTTTAGGGTTTAGGGTTTAGGGTTTAGGGTTTAGGGTTTAGGGTTTAGGGTTTAGGGTTTAGGGTTTAGGGTTTAGGGTTTAGGGTTTAGGGTTTAGGGTTTAGGGTTTAGGGTTTAGGGTTTAGGGTTTAGGGTTTAGGGTTTAGGGTTTAGGGTTTAGGGTTTAGGGTTTAGGGTTTAGGGTTTAGGGTTTAGGGTTTAGGGTTTAGGGTTTAGGGTTTAGGGTTTAGGGTTTAGGGTTTAGGGTTTAGGGTTTAGGGTTTAGGGTTTAGGGTTTAGGGTTTAGGGTTTAGGGTTTAGGGTTTAGGGTTAGGGTTTAGGGTTTAGGGTTTAGGGTTTAGGGTTTAGGGTTAGGGTTTAGGGTTTAGGGTTTAGGGTTTAGGGTTAGGGTTTAGGGTTTAGGGTTTAGGGTTTAGGGTTAGGGTTAGGGTTAGGGTTAGGGTTTAGGGTTAGGGTTTAGGGTTTAGGGTTTAGGGTTTAGGGTTTAGGGTTTAGGGTTAGGGTTTAGGGTTTAGGGTTTAGGGTTTAGGGTTTAGGGTTTAGGGTTTAGGGTTAGGGTTTAGGGTTTAGGGTTTAGGGTTTAGGGTTTAGGGTTAGGGTTTAGGGTTTAGGGTTTAGGGTTTAGGGTTTAGGGTTTAGGGTTTAGGGTTTAGGGTTTAGGGTTTAGGGTTTAGGGTTTAGGGTTTAGGGTTTAGGGTTTAGGGTTTTTAGGGTTTAGGGTTTAGGGTTTAGGGTTAGGGTTTAGGGTTTAGGGTTTAGGGTTTAGGGTTAGGGTTTAGGGTTTAGGGTTTAGGGTTTAGGGTTTAGGGTTTAGGGTTTAGGGTTTAGGGTTTAGGGTTTAGGGTTTAGGGTTTAGGGTTTAGGGTTTAGGGTTTAGGGTTTAGGGTTTAGGGTTTAGGGTTTAGGGTTTAGGGTTTAGGGTTTAGGGTTTAGGGTTTAGGGTTTAGGGTTTAGGGTTTAGGGTTTAGGGTTTAGGGTTTAGGGTTTAGGGTTTAGGGTTTAGGGTTAGGGTTTAGGGTTTAGGGTTTAGGGTTTAGGGTTTAGGGTTTAGGGTTTAGGGTTTAGGGTTTAGGGTTAGGTTTAGGGTTTAGGGTTTAGGGTTTAGGGTTTAGGGTTTAGGGTTTAGGGTTAGGGTTTAGGGTTTAGGGTTTAGGGTTTAGGGTTAGGGTTTAGGGTTTAGGGTTAGGGTTAGGGTTAGGGTTTAGGGTTTAGGGTTAGGTTAGGGTTAGGGTTTAGGGTTTAGGGTTTAGGGTTTAGGGTTTAGGGTTTAGGGTTTAGGGTTTAGGGTTTAGGGTTTAGGGTTTAGGGTTTAGGGTTAGGGTTTAGGGTTTAGGGGTTTAGGATTAGGGTTTAGGGTTTTTAGGGTTTAGGGTTTAGGGTTAGGGTTTAGGGTTTAGGGTTTAGGGTTTTAGGGTTTAGGGTTTAGGGTTTAGGGTTTAGGGTTTAGGGTTTAGGGTTTAGGGTTTAGGGTTTAGGGTTTAGGGTTTAGGGTTTAGGGTTTAGGGTTTAGGGTTTAGGGTTTAGGGTTTAGGGTTTAGGGTTTAGGGTTTAGGTTTAGGGTTTAGGGTTTAGGGTTTAGGGTTTAGGGTTTAGGGTTTAGGGTTTAGGGTTTAGGGTTTAGGGTTTAGGGTTTAGGGTTGGGTTTAGGGTTTAGGGTTTAGGGTTTAGGGTTTAGGGTTTAGGGTTTAGGGTTTAGGGTTTAGGGTTTAGGGTTAGGGTTTAGGGTTTAGGGTTTAGGGTTTAGGGTTTAGGGTTTAGGGTTTAGGGTTTAGGGTTTAGGGTTTAGGGTTTAGGGTTTAGGGTTTAGGGTTTAGGGTTTAGGGTTAGGGTTTAGGGTTTAGGGTTAGGGTTTAGGGTTTAGGGTTAGGGTTTAGGGTTAGGGTTAGGGTTAGGGTTTAGGGTTTAGGGTTTGGGTTAGGTTTAGGGTTAGGGTTTAGGGTTTAGGGTTAGGGTTTGGGGTTAGGGTTTAGGGTTTAGGGTTTAGGGTTTAGGGTTTAGGGTTTTAGGGTTTAGGGTTTAGGGTTAGGGTTTAGGGTTTAGGGTTTAGGGTTTAGGGTTTAGGGTTTAGGGTTTAGGGTTTAGGGTTTAGGGTTTAGGGTTTAGGGTTTTAGGGTTTAGGGTTTAGGGTTTAGGGTTAGGGGTTTAGGGTTTAGGGTTTAGGGTTAGGGTTTAGGGTTAGGGTTTAGGGTTTAGGGTTAGGGTTTAGGGTTTAGGGTTTAGGGTTTAGGGTTTAGGGTTTAGGGTTTAGGGTTTAGGGTTTAGGGTTTAGGGTTTAGGGTTTAGGGTTTAGGGTTTAGGGTTTAGGGTTTAGGGTTTAGGGTTTAGGGTTTAGGGTTTAGGGTTTAGGGTTTAGGGTTTAGGGTTTAGGGTTTAGGGTTTAGGGTTTAGGGTTTTTTAGGGTTTAGGGTTTAGGGTTTAGGTTAGGGTTTAGGGTTTAGGGTTTAGGGTTTAGGGTTTAGGGTTTAGGGTTTAGGGTTTTAGGGTTTAGGGTTAGGGTTTAGGGTTTAGGGTTTAGGGTTTAGGGTTTAGGGGTTTAGGGTTTAGGGTTTAGGGTTTAGGGTTTAGGGTTTAGGGTTAGGTTTAGGGTTTAGGGTTTAGGGTTTAGGGTTTAGGGTTTAGGGTTTAGGGTTTAGGGTTTAGGGTTTAGGGTTTAGGGTTTAGGGTTTAGGGTTAGGGTTTAGGGTTTAGGGTTTAGGGTTTAGGGTTTAGGGTTTAGGGTTTAGGGTTTAGGGTTTAGGGTTTAGGGTTTAGGGTTTAGGGTTTAGGGTTTAGGGTTTAGGGTTTAGGGTTTAGGGTTTAGGGTTTAGGGTTTAGGGTTAGGGTTTAGGGTTTAGGGTTTAGGGTTTAGGGTTTAGGGTTTAGGGTTTAGGGTTTAGGGTTTAGGGTTTAGGGTTTAGGGTTTAGGGTTTAGGGTTTAGGGTTTAGGGTTTAGGGTTTAGGGTTTAGGGTTTAGGGTTTAGGGTTTTAGGGTTTAGGGTTTAGGGTTTAGGGTTTAGGGTTTAGGGTTTAGGGTTTAGGGTTAGGGTTTAGGGTTTAGGGTTTAGGGTTTAGGGTTTAGGGTTTAGGGTTGGGGTTTAGGGTTTAGGGTTTAGGGTTTAGGGTTTAGGGTTTAGGGTTTAGGGTTTAGGGTTTAGGGTTTAGGGTTTAGGGTTAGGGTTTAGGGTTTAGGGTTAGGGTTTAGGGTTTAGGGTTTAGGGTTTAGGGTTTAGGGTTTAGGGTTTAGGGTTTAGGGTTAGGGTTTAGGGTTGTTAGGGTTTAGGGTTTAGGGTTTAGGGTTTAGGGTTTAGGTTTAGGGTTTAGGGTTTAGGGTTTAGGGTTTAGGGTTTAGGGTTTAGGGTTTAGGGGTTTAGGGTTTAGGGTTGTTTAGGGTTTAGGGTTTAGGGTTTAGGGTTTAGGGTTTAGGGTTTAGGGTTTAGGGTTTAGGGTTTAGGGTTAGGGTTAGGGTTTAGGGTTTAGGGTTTAGGGTTTAGGGTTTAGGGTTTAGGGTTTAGGGTTTAGGGTTAGGGTTTAGGGTTTAGGGTTTAGGGTTTAGGGTTTAGGGTTTAGGGTTAGGGTTTAGGGTTAGGGTTTAGGGTTTAGGGTTTAGGGTTTAGGGTTTAGGGTTTAGGGTTTAGGGTTTAGGGTTTAGGGTTTAGGGTTTAGGGTTTAGGGTTTAGGGTTAGGGTTTAGGGTTTAGGGTTTAGGGTTTAGGGTTTAGGGTTTAGGGTTTAGGGTTTAGGGTTTAGGGTTTAGGGTTTAGGGTTTAGGGTTTAGGGTTTAGGTTTAGGGTTTAGGGTTTAGGGTTTAGGGTTTAGGGTTTAGGGTTTAGGGTTTAGGGTTTAGGGTTTAGGGTTTAGGGTTTAGGGTTAGGGTTTAGGGTTTAGGGTTTAGGGTTTAGGGTTTAGGGTTTAGGGTTTAGGGTTTAGGGTTTAGGGTTTAGGGTTTAGGGTTTAGGGTTTAGGGTTTAGGGTTTAGGGTTTAGGGTTTAGGGTTTAGGGTTTAGGGTTAGGGTTTAGGGTTTAGGGTTAGGGTTTAGGGTTTAGGGTTTAGGGTTTAGGGTTTAGGGTTTAGGGTTTAGGGTTTAGGGTTTAGGGTTTAGGGTTTAGGGTTTAGGGTTTAGGGTTTAGGGTTTAGGGTTTAGGGTTTAGGGTTTAGGGTTTAGGGTTTAGGGTTTAGGGTTTAGGGTTAGGTTAGGGTTTAGGGTTTAGGGTTTAGGGTTTAGGGTTTAGGGTTAGGGTTTAGGGTTTAGGGTTTAGGGTTTTAGGGTTTAGGGTTTAGGGTTTAGGGTTTAGGGTTTAGGGTTTAGGGTTTAGGGTTTAGGGTTTAGGGTTTAGGGTTTAGGGTTTAGGGTTTAGGGTTTAGGGTTTTAGGGTTTAGGGTTTGGTTTAGGGTTTAGGGTTTTAGGGTTTAGGGTTTAGGGTTTAGGGTTTAGGGTTTAGGGTTTAGGGTTTAGGGTTTAGGGTTTAGGGTTTAGGGTTTAGGGTTAGGGTTTAGGGTTTAGGGTTAGGGTTAGGGTTTAGGGTTTAGGGTTAGGGTTTAGGGTTTAGGGTTAGGGTTTAGGGTTTAGGGTTTAGGGTTTAGGGTTTAGGGTTTAGGGTTAGGGTTTAGGGTTTAGGGTTTAGGATTAGGGTTTAGGGTTTAGGGTTTAGGGTTTAGGGTTTAGGGTTTAGGGTTTAGGGTTTAGGGTTTAGGGTTTAGGTTTAGGGTTTAGGGTTTAGGGTTAGGGTTTAGGGTTTAGGGTTTAGGGTTTAGGGTTTAGGGTTTAGGGTTTAGGGTTTAGGGTTTAGGGTTTAGGGTTTAGGGTTTAGGGTTTAGGGTTTAGGGTTTAGGGTTAGGGTTTAGGGTTTAGGGTTTAGGGTTAGGGTTTAGGGTTTAGGGTTTAGGGTTTTAGGGTTAGGGTTTAGGGTTTAGGGTTTAGGGTTTAGGGTTTAGGGTTTAGGGTTTAGGGTTAGGGTTTAGGGTTTAGGGTTTAGGGTTTAGGGTTTAGGGTTTAGGGTTTAGGGTTTTAGGGTTTAGGGTTTAGGGTTTAGGGTTTAGGGTTAGGGTTTAGGGTTTAGGGTTTAGGGTTAGGGTTTAGGGTTTAGGGTTTAGGGTTTAGGGTTTAGGGTTTAGGGTTTAGGGTTATTAGGGTTTAGGGTTTAGGGTTTAGGGTTTAGGGTTTAGGGTTTAGGGTTTAGGGTTTAGGGTTTAGGGTTTAGGGTTTAGGGTTTAGGGTTTAGGGTTTAGGGTTTAGGGTTTAGGGTTTAGGGTTTAGGGTTTAGGGTTAGGGTTTAGGGTTTAGGGTTTAGGGTTTAGGGTTTAGGGTTTAGGGTTTAGGGTTTAGGGTTTAGGGTTTAGGGTTTAGGGTTAGGGTTTAGGGTTTAGGGTTTAGGGTTTAGGGTTTAGGGTTTAGGGTTTAGGGTTTAGGGTTTAGGGTTTAGGGTTAGGGTTTAGGGTTTAGGGTTTAGGGTTTAGGGTTTAGGGTTTAGGGTTTAGGGTTTAGGGTTTAGGGTTTAGGGTTTAGGGTTTAGGGTTTAGGGTTTAGGGTTTAGGGTTTTAGGGTTAGGGTTTAGGGTTTTTAGGGTTTAGGGTTTAGGGTTTAGGGTTTAGGGTTTAGGGTTTAGGGTTTAGGGTTTAGGGTTTAGGGTTTAGGGTTTAGGGTTAGGGTTTAGGGTTTAGGGTTTAGGGTTTAGGGTTTAGGGTTTAGGGTTTAGGGTTTAGGGTTTAGGGTTTAGGGTTTAGGGTTTAGGGTTTAGGGTTTAGGGTTTAGGGTTTAGGGTTTAGGGTTTAGGGTTAGGGTTTAGGGTTTAGGGTTTAGGGTTTAGGGTTTAGGGTTTAGGGTTTAGGGTTTAGGGTTTAGGTTTAGGGTTTAGGGTTTAGGGTTTAGGTTTAGGGTTTAGGGTTTAGGGTTTAGGGTTTAGGGTTTTAGGGTTTAGGGTTTAGGGTTTAGGGTTTAGGGTTTAGGGTTTAGGGTTTAGGGTTTAGGGTTTAGGGTTTAGGGTTTAGGGTTTAGGGTTTAGGGTTTAGGGTTAGGGTTTAGGGTTAGGGTTTAGGGTTTAGGGTTTAGGGTTTAGGGTTAGGGTTTAGGGTTTAGGGTTTAGGGTTTAGGGTTTAGGGTTTAGGGTTTAGGGTTTAGGGTTTAGGGTTTAGGGTTTAGGGTTTAGGGTTTAGGGTTTAGGGTTTTAGGGTTTAGGGTTTAGGGTTTAGGGTTTAGGGTTAGGGTTAGGGTTTAGGGTTTAGGGTTAGGGTTTAGGGTTTAGGGTTAGGGTTTAGGGTTTAGGGTTAGGTTTAGGGTTTAGGGTTTAGGGTTTAGGGTTTAGGGTTTAGGGTTTAGGGTTTAGGGTTTAGGGTTAGGGTTTAGGGTTTGGGGTTTAGGGTTAGGGTTTAGGGTTAGGGTTAGGGTTTAGGGTTTAGGGTTTAGGGTTTAGGGTTTAGGGTTTAGGGTTTAGGGTTTAGGGTTTAGGGTTTAGGGTTTAGGGTTAGGGTTTAGGGGTTTAGGGTTTAGGGTTTAGGGTTTAGGGTTTAGGGTTAGGGTTAGGGTTTAGGGTTTAGGGTTTAGGGTTTAGGGTTAGGGTTTAGGGTTTAGGGTTTAGGGTTAGGGTTTAGGGTTTAGGGTTTAGGGTTTAGGGTTTAGGGTTTAGGGTTTAGGGTTTAGGGTTAGGGTTTAGGGTTAGGGTTTTAGGGTTTAGGGTTTAGGGTTAGGGTTTAGGGTTTAGGGTTTAGGGTTTAGGGTTTAGGGTTTAGGGTTTAGGGTTTAGGGTTTAGGGTTTAGGGTTTAGGGTTTAGGGTTTAGGGGTTTAGGGTTTAGGGTTTAGGGTTTAGGGTTAGGGTTAGGGTTTAGGGTTTAGGGTTTAGGGTTTAGGGGTTAGGGTTTAGGGTTAGGGTTTAGGGTTTAGGGTTAGGGTTTAGGGTTTAGGGTTTAGGGTTAGGGTTAGGGTTAGGGTTTAGGGTTTAGGGTTTAGGGTTTAGGGTTAGGGTTTAGGGTTTAGGGTTAGGGTTTAGGGTTAGGGTTTAGGGTTTTAGGGTTAGGGTTTAGGGTTAGGGTTTAGGGTTTAGGGTTAGGGTTTAGGGTTTAGGGTTTAGGGTTTAGGGTTTAGGGTTTAGGGTTTAGGGTTTAGGGTTTAGGGTTAGGGTTTAGGGTTTAGGGTTTAGGGTTTAGGGTTTAGGGTTTAGGGTTTAGGGTTTAGGGTTTAGGGTTTAGGGTTTAGGGTTTAGGGTTAGGGTTTAGGGTTTAGGGGTTTAGGGTTTAGGGTTTAGGGTTTAGGGTTTAGGGTTTAGGGTTTAGGGTTTAGGGTTAGGGTTTAGGGTTTAGGGTTTAGGGGTTTAGGGTTAGGGTTTAGGGTTTAGGGTTTAGGGTTTAGGGTTTAGGGTTTAGGGTTAGGGTTTAGGGTTTAGGGTTAGGGTTTAGGGTTAGGGTTTAGGGTTTAGGGTTTAGGGTTTAGGGTTTAGGGTTTAGGTTAGGGTTTAGGGTTAGGGTTTAGGGTTTAGGGTTTAGGGTTTAGGGTTTAGGGTTTAGGGTTTTAGGGTTTAGGGTTTAGGGTTTAGGGTTTAGGGTTTAGGGTTAGGGTTTAGGGTTTAGGGTTTAGGGTTTAGGGTTAGGGTTTAGGGTTTAGGGTTTAGGGTTTAGGGTTTAGGGTTTAGGGTTTAGGGTTAGGGTTTAGGGGTTTAGGGTTTAGGGTTTAGGGTTTAGGGTTAGGTTAGGGTTTAGGGTTAGGGTTTAGGGTTTAGGGTTTAGGGTTTAGGGTTTAGGGTTAGGGTTTAGGGTTTAGGTTTAGGGTTTAGGGTTTAGGGTTTAGGGTTTAGGGTTTAGGGTTTAGGGTTTAGGGTTTAGGGTTTTAGGGTTTAGGGTTTAGGGTTTAGGGTTTAGGGTTTAGGGTTTAGGGTTTAGGGTTTAGGGTTTAGGGTTTAGGGTTTAGGGTTTAGGGTTAGGGTTTAGGGTTTAGGGTTTAGGGTTTAGGGTTAGGGTTTAGGGTTTAGGGTTTAGGGTTTAGGGTTTAGGGGTTTAGGGTTTAGGGTTTAGGGTTTAGGGTTAGGGTTTAGGGTTTAGGGTTTAGGGTTTAGGGTTTAGGGTTTAGGGTTTAGGGTTTAGGGTTTAGGGTTAGGGTTAGGGTTTAGGGTTTAGGGTTTAGGGTTTAGGGTTTAGGGTTTAGGGTTTAGGGTTTAGGGTTAGGGTTAGGGTTTAGGGTTTAGGGTTTAGGGTTAGGGTTTAGGGTTTAGGGTTTAGGGTTTAGGGTTTAGGGTTTAGGGTTTAGGGTTTAGGGTTTAGGGTTTAGGGTTTAGGGTTAGGGTTTAGGGTTTAGGGTTTAGGGTTTAGGGTTTAGGGTTTAGGGTTTAGGGTTTAGGGTTTAGGGTTTAGGGTTTAGGGTTTAGGGTTTAGGGTTTAGGGTTTAGGGTTTAGGGTTTAGGGTTTAGGGTTTAGGGTTTAGGGTTTAGGGTTAGGGTTTAGGGTTTAGGGTTTAGGGTTTAGGGTTTAGGGTTTAGGGTTTTAGGGTTTAGGGTTTAGGGTTTAGGGTTTAGGGTTTAGGGTTTAGGGTTAGGGTTTAGGGTTTAGGGTTTAGGGTTTAGGGTTTAGGGTTTAGGGTTAGGGTTTAGGGTTAGGGTTTTAGGGTTTAGGGTTTAGGGTTTAGGGTTAGGGGTTTTTAGGGTTTAGGGTTTAGGGTTTAGGGTTAGGGTTTAGGGTTTAGGGTTTAGGGTTTAGGGTTTAGGGTTTAGGGTTTAGGGTTTAGGGTTTAGGGTTAGGGTTTAGGGTTTAGGGTTTAGGGTTTAGGGTTTAGGGTTTAGGGTTAGGGTTTAGGGTTTAGGGTTAGGGTTTAGGGTTTAGGGTTTAGGGTTTAGGGTTTAGGGTTTAGGGTTTAGGGTTTAGGGTTTAGGGTTTAGGGTTTAGGGTTTAGGGTTAGGGTTTAGGGTTAGGGTTTAGGGTTTAGGGTTTAGGGTTTAGGGTTAGGGTTTAGGGTTTAGGGTTTAGGGTTAGGGTTTAGGGTTAGGGTTAGGGTTTAGGGTTTAGGGTTTAGGGTTTAGGGTTAGGGTTTAGGGTTTAGGGTTTTTAGGGTTTAGGGTTTAGGGTTTAGGGTTTAGGGTTTAGGGTTTAGGGTTAGGGTTTAGGGTTTAGGGTTAGGGTTTAGGGTTTAGGGTTTAGGGTTTAGGGTTTAGGGTTTAGGGTTTTTAGGGTTTAGGGTTTAGGGTTTAGGGTTTAGGGTTTAGGGTTTAGGGTTTAGGGTTTAGGGTTTAGGGTTTAGGGTTTAGGGTTTAAGGGTTTAGGGTTTAGGGTTTAGGGGTTTAGGGGGTTTAGGGTTTAGGGTTTAGGGTTTAGGGTTTAGGGTTTAGGGGTTTAGGGTTAGGGTTTAGGGTTTAGGGTTTAGGGTTTAGGGTTTAGGGTTTAGGGTTTAGGGTTAGGGTTTAGGGTTAGGGTTTAGGGTTTAGGGTTTAGGGTTTAGGGTTTAGGGTTTAGGGTTTAGGGTTTTAGGGTTAGGGTTTAGGGTTTAGGGTTTAGGGTTTAGGGTTTAGGGTTTAGGGTTTAGGGTTTAGGGGTTTAGGGTTTAGGGTTTAGGGTTTAGGGTTTAGGGTTTAGGGTTTAGGGTTTAGGGTTTAGGGTTTAGGGTTTAGGGTTTAGGGTTTAGGGTTTAGGGTTTAGGTTAGGGTTTAGGGTTTAGGGTTTAGGGTTTAGGGTTTAGGGTTTAGGGTTTAGGGTTTAGGGTTTAGGGTTTAGGGTTTAGGGTTAGGGTTTAGGGTTTAGGGTTTTAGGGTTTAGGGTTTAGGGTTTAGGGTTTAGGGTTTAGGGTTTAGGGTTTTAGGGTTTAGGGTTTAGGGTTTAGGGTTTAGGGTTTAGGGTTTAGGGTTTAGGGTTTAGGGTTTAGGGTTTAGGGTTTAGGGTTAGGGTTTAGGGTTTAGGGTTTAGGGTTTAGGGTTTAGGGTTTAGGGTTTAGGGTTTAGGGTTTAGGGTTTAGGGTTTAGGGTTTAGGGTTTAGGGTTTAGGGTTTAGGGTTTAGGGTTTAGGGTTTAGGGTTTAGGGTTTAGGGTTTAGGGTTAGGGTTTAGGGTTTAGGGTTTAGGGTTTAGGGTTTAGGGTGGTTTAGGGTTTAGGGTTTAGGGTTTAGGGTTTAGGGTTTAGGGTTTAGGGTTTAGGGTTTAGGGTTTAGGGTTAGGGTTTAGGGTTAGGGTTTAGGGTTTAGGGTTTAGGGTTTAGGGTTTAGGGTTTAGGGTTTAGGGTTTAGGGTTTAGGGTTTAGGGTTTAGGGTTTAGGGTTTAGGGTTTAGGTTTAGGGTTTAGGGTTTAGGGTTTAGGGTTTAGGGTTTAGGGTTTAGGGTTTAGGGTTTAGGGTTTAGGGTTTAGGGTTTAGGGTTTAGGGTTAGGGTTTTAGGGTTTAGGGTTTAGGGTTTAGGGTTTAGGGTTAGGGTTTAGGGTTTAGGGTTTAGGGTTTAGGGTTAGGGTTTAGGGTTAGGGTTTAGGGTTTAGGGTTTAGGGTTTAGGGTTTAGGGTTTAGGGTTTAGGGTTTAGGGTTTAGGGTTTAGGGTTAGGGTTTAGGGTTTAGGGTTTAGGGTTTAGGGTTTAGGGTTTAGGGTTTAGGGTTTAGGGTTTAGGGTTTAGGGTTTAGGGTTTAGGGTTTAGGGTTTAGGGTTTAGGGTTTAGGGTTTAGGGTTAGGGTTTAGGGTTTAGGGTTTAGGGTTTAGGGTTTTAGGGTTTAGGGTTTAGGGTTTAGGGTTTAGGGTTTAGGGTTAGGGTTTAGGGTTTAGGGTTTAGGGTTTAGGGTTTAGGGTTAGGGTTTAGGGTTTAGGGTTTAGGGTTTAGGGTTTAGGGTTAGGGTTTAGGGTTTAGGGTTTAGGGTTTAGGGTTTAGGGTTTAGGGTTTAGGGTTTAGGGTTTAGGGTTTAGGGTTTAGGGTTTAGGGTTTAGGGGTTTAGGGTTTAGGGTTTAGGGTTTAGGGTTTAGGGTTTAGGGTTTAGGGTTTAGGGTTTAGGGTTTAGGGTTTAGGGTTTAGGTTTAGGGTTTAGGGTTTAGGGTTTAGGGTTTAGGGTTTAGGGTTTAGGGTTTAGGGTTTAGGGTTTAGGGTTTAGGGTTTAGGGTTTAGGGTTTAGGGTTTAGGGTTTAGGGTTTAGGGTTTTAGGGTTTAGGGTTTAGGGTTTAGGGTTAGGGTTTAGGGTTTAGGGTTTAGGGTTTAGGGTTTAGGGTTTAGGGTTTAGGGTTAGGGTTTAGGGTTTAGGGTTTAGGGTTTTAGGGTTTAGGGTTTAGGGTTAGGGTTTAGGGTTTAGGGTTTAGGTTTAGGGTTTAGGGTTTAGGGTTTAGGGTTTAGGGTTTAGGGTTTAGGGTTTAGGGTTTAGGGTTTAGGGTTTAGGGTTTAGGGTTTAGGGTTTAGGGTTTAGGGTTTAGGGTTTAGGGTTTAGGGTTTAGGGTTTAGGGTTTAGGGTTTAGGGTTTTAGGGTTTTAGGGTTTAGGGTTTAGGGTTTAGGGTTTAGGGTTTAGGGTTTAGGGTTTAGGGTTTTAGGGTTTAGGGTTTAGGGTTTAGGGTTAGGGTTTAGGGTTTAGGGTTTAGGGTTTAGGGTTTAGGGTTTAGGGTTTTAGGGTTTAGGGTTTAGGGTTTAGGGTTTAGGGTTTAGGGTTTAGGGTTTAGGGGTTTAGGGTTTAGGGTTTAGGGTTTAGGGTTTAGGGTTTAGGGTTTAGGGTTTAGGGTTTAGGGTTTAGGGTTTAGGGTTTAGGGTTTAGGGTTTAGGGTTTAGGGTTTAGGGTTTAGGGTTTAGGGTTAGGGTTTAGGGTTTAGGGTTTAGGGTTTAGGGTTTAGGGTTTAGGGTTTAGGGTTTAGGGTTTAGGGTTTAGGGTTAGGGTTTAGGGTTTAGGGTTTAGGGTTTAGGGTTTAGGGTTTAGGGTTTTAGGGTTTAGGGTTTAGGGTTTAGGGTTTAGGGTTTAGGGTTTTAGGGTTTAGGGTTTAGGGTTTAGGGTTTAGGGTTTAGGGTTTAGGGTTTAGGGTTTAGGGTTTAGGGTTTAGGGTTTAGGGTTTAGGGTTTAGGGTTTTAGGGTTAGGGTTTAGGGTTTAGGGTTTAGGGTTTAGGGTTTAGGGTTTAGGGTTTAGGGTTTAGGGTTAGGGTTTAGGGTTTAGGGTTTAGGGTTTAGGGTTTAGGGTTTAGGGTTTAGGGTTTAGGGTTTAGGGTTTAGGGTTTAGGGTTTAGGGTTTAGGGTTAGGGTTTAGGGTTTAGGGTTTAGGGTTTAGGGTTTAGGGTTTAGGGTTTAGGGTTTAGGGTTTAGGGTTTAGGGTTAGGGTTTAGGGTTTAGGGTTTAGGGTTTAGGGTTTAGGGTTTAGGGTTTAGGGTTTAGGGTTTAGGGTTTAGGGTTTAGGGTTTAGGGTTTAGGGTTTAGGGTTTAGGGTTTAGGGTTTAGGGTTTAGGGTTTAGGGTTTAGGGTTTAGGGTTTAGGGTTTAGGGTTTAGGGTTTAGGGTTAGGGTTTAGGGTTTAGGGTTTAGGGTTTAGGGTTTAGGGTTTAGGGTTTAGGGTTTTAGGGTTTAGGGTTTAGGGTTTAGGGTTTTAGGGTTTAGGGTTAGGGTTTAGGGTTAGGGTTTAGGGTTTTAGGGTTTAGGGTTTAGGGTTTAGGGTTTAGGGTTTAGGGTTTAGGGTTTAGGGTTTAGGGTTTAGGGTTTTAGGGGTTTAGGGTTTAGGGTTTAGGGTTTAGGGTTTTAGGGTTTAGGGTTTAGGGTTTAGGGTTTAGGTTTAGGGTTAGGGTTTAGGGTTTAGGGTTTAGGGTTTAGGGTTAGGGTTTAGGGTTTAGGGTTTAGGGTTTAGGGTTTAGGGTTTAGGGTTTAGGGTTTAGGGTTTAGGGTTTAGGGTTTAGGGTTTAGGGTTTAGGGTTTAGGGTTTAGGGTTTAGGGTTTAGGGTTTAGGGTTTAGGGTTTAGGGTTTAGGGTTTAGGGTTTAGGGTTTAGGGTTTAGGGTTTAGGGTTTAGGGTTTAGGGTTTAGGGTTTTAGGGTTTAGGGTTTAGGGTTTAGGTTTAGGGTTTAGGGTTTAGGGTTTAGGGTTTAGGGTTTAGGGTTTTAGGGTTTAGGTTTAGGGTTTTAGGGTTTAGGGTTTAGGGTTTTAGGGTTTTAGGGTTTAGGGTTAGGGTTTAGGGTTTAGGGTTTAGGGTTTAGGGTTTAGGGTTTAGGGTTTAGGGTTTAGGGTTTAGGGTTTAGGGTTTAGGGTTAGGGTTTAGGGTTTAGGGTTTAGGGTTTAGGGTTTTAGGGTTTAGGGTTTAGGGTTTAGGGTTTAGGGTTTAGGGTTTAGGGGTTAGGGTTTAGGGTTTAGGGTTTAGGGTTTAGGGTTTAGGGTTTAGGTTTAGGGTTTAGGGTTTAGGGTTTAGGGTTTAGGGTTAGGGTTTAGGGTTTAGGGTTTAGGGTTTAGGGTTTAGGGTTTAGGGTTTAGGGTTAGGGTTTAGGGTTTAGGGTTTAGGGTTTAGGGTTTAGGGGTTTAGGGTTTAGGGTTTAGGGTTTAGGGTTTAGGGTTTAGGGTTTAGGGTTTAGGGTTAGGGTTTAGGGTTTAGGGTTTAGGGTTTAGGGTTTGGTAGGGTTTTAGGGTTTAGGGTTTTAGGGTTTAGGGTTTAGGGTTTAGGTTTAGGGTTTAGGGTTTTAGGGTTTTAGGGTTTAGGGTTAGGGTTTAGGGTTTAGGGTTTAGGGTTTAGGGTTTAGGGTTTTAGGGTTTAGGGTTTAGGGTTTAGGGTTAGGGTTTAGGGTTTAGGGTTTAGGGTTTAGGGTTTAGGGTTTAGGGTTAGGGTTTAGGGTTTAGGGTTTAGGGTTTAGGGTTTAGGTTAGGGTTTAGGGTTTAGGGTTTAGGGTTTAGGGTTAGGGTTTAGGGTTTAGGGTTTAGGGTTTAGGGTTTAGGGTTTAGGGTTTAGGGTTTAGGGTTTTAGGGTTTAGGGTTTAGGGTTTAGGGTTTAGGGTTTAGGGTTTAGGGTTTTAGGGTTAGGGTTTAGGGTTTAGGGTTTAGGGTTTAGGGTTTAGGGTTTAGGTTTAGGGTTTAGGGTTTAGGGTTTAGGGTTTAGGGTTTAGGGTTTAGGGTTTAGGTTTAGGGTTTAGGGTTTAGGGTTTAGGGTTTAGGGTTTAGGGTTGTTTAGGGTTTAGGGTTTAGGGTTTAGGGTTTAGGGTTTAGGGTTAGGGTTTAGGGTTTAGGGTTTAGGGTTTAGGGTTTAGGGTTTAGGGTTTAGGGTTTAGGGTTTAGGGTTTAGGGTTTAGGGTTTAGGGTTTAGGGTTTAGGGTTTAGGGTTTAGGGTTTAGGGTTTAGGGTTTAGGGTTTAGGGTTTAGGGTTAGGGTTTAGGGTTTAGGGGTTTAGGGTTAGGGTTTAGGGTTTAGGGTTAGGGTTTAGGGTTTAGGGTTTAGGGTTTAGGGTTTAGGGTTTAGGGTTTAGGGTTTAGGGTTTAGGGTTAGGGTTTAGGGTTTAGGGTTTAGGGTTTAGGGTTTAGGGTTTAGGGTTTAGGGTTTAGGGTTTAGGGTTAGGGTTTTAGGGTTTAGGGTTTTAGGGTTTAGGGTTTAGGGTTTAGGGTTTAGGGTTTAGGGTTTAGGGTTTAGGGTTAGGGTTTAGGGTTTAGGGTTTAGGGTTTAGGGTTAGGGTTTAGGGTTTAGGGTTTAGGGTTTAGGGTTTAGGGTTAGGGTTTAGGGTTTAGGGTTAGGGTTTAGGGTTTAGGGTTTAGGGTTTAGGGTTAGGGTTTAGGGTTAGGGTTTAGGGTTTAGGGTTTAGGGTTGGTTAGGGTTTAGGGTTTAGGGTTTAGGGTTTAGGGTTTAGGGTTTAGGGTTTAGGGTTTAGGGTTAGGGTTTAGGGTTTAGGGTTTAGGGTTTAGGGTTAGGGTTTAGGGTTAGGGTTTAGGGTTTAGGGTTTAGGGTTTAGGGTTTAGGGTTTTGGGTTTAGGGTTTAGGGTTAGGGTTTAGGGTTTAGGGTTTAGGGTTTAGGGTTTAGGGTTTAGGGTTTAGGGTTAGGGTTTAGGGTTTAGGGTTTAGGGTTAGGGTTTAGGGTTAGGGTTTAGGGTTTAGGGTTTAGGGTTTAGGGTTTAGGGTTTAGGGTTAGGGTTTAGGGTTTAGGGTTTAGGGTTTGGTTAGGGTTTAGGGTTTAGGGTTTAGGGTTTAGGGTTTAGGGTTTAGGGTTTAGGGTTTAGGGTTTTAGGGTTTAGGGTTTAGGGTTTAGGGTTTAGGGTTTAGGGTTTAGGGTTTAGGGTTTAGGGTTTAGGGTTTAGGGTTTAGGGTTTAGGGTTTAGGGTTTAGGGTTTAGGGTTTAGGGTTTAGGGTTTAGGGTTTAGGGTTTAGGGTTTAGGGTTAGGGTTTAGGGTTTAGGGTTTAGGGTTTAGGGTTAGGGTTTAGGGTTTAGGGTTTAGGGTTTAGGGTTTAGGGTTTAGGGTTTAGGGTTTAGGGTTAGGGTTTAGGGTTTAGGGTTTAGGGTTTAGGGTTTAGGGTTTAGGGTTAGGGTTTAGGGTTTAGGGTTTAGGGTTTAGGGTTTAGGGTTAGGGTTTAGGGTTTAGGGTTTAGGGTTTAGGGTTTAGGGTTTAGGGTTTAGGGTTAGGGTTTAGGGTTTAGGGTTTAGGGTTTAGGGTTTAGGGTTTAGGGTTTAGGGTTTTAGGGTTTAGGGTTTAGGGTTTAGGGTTTAGGGTTTAGGGTTAGGGGTTTAGGGTTTAGGGTTTAGGGTTTAGGGTTTAGGGTTTTAGGGTTAGGGTTAGGGGTTTAGGGTTTAGGGGTTTAGGGTTTAGGGTTTATGGGTTTAGGGTTTAGGGTTTAGGGTTTAGGGTTTAGGGTTTAGGGTTAGGGTTTAGGGTTTAGGGTTTAGGGTTTAGGGTTTAGGGTTTAGGGTTAAGGGGTTTAGGGTTTAGGGTTTAGGGTTTAGGGTTTAGGGTTTAGGGTTTAGGGTTTAGGGTTTAAGGTTTAGGGTTTAGGGTTTAGGGTTTTAGGGTTTAGGGTTTAGGGTTAGGGTTTAGGGTTTAGGGTTTAGGGTTTAGGGTTTAGGGTTTAGGGTTTAGGGTTTAGGGTTTAGGGTTTAGGGTTTAGGGTTTAGGGTTTAGGGTTTAGGGTTTAGGGTTAGGGTTTAGGGTTTAGGGTTTAGGTTTAGGGTTTAGGGTTTAGGGTTAGGGGTTTTAGGGTTTAGGGTTTAGGGTTTAGGGGTTTAGGGTTTAGGGTTTAGGGTTTAGGGTTTAGGGTTTAGAGGTTTA

**6DL**

CACTTAGGG**TTTAGGG**TTTAGGGTTTAGGGTTGGGTTTAGGGTTTAGGGTTTATGTTTAGGGTTGTAGGGTTTAGGGTTTAGGTTTAGGGTTTAGGGTTAGGGTTTAGGGTTTAGGGTTTAGGGTTTAGGGTTAGGGTTTAGGGTTTAGGGGGTTTAGGGTTTAGGGTTTAGGGTTTAGGGTTTAGTGGTTTAGGGTTTAGGGGGGTTTAGGTTTAGGGTTTAGGGTTTAGGGTTAGGGTTTAGGGTTTAGGGTTTAGGGTTTAGGGTTTAGGGTTTAGGGTTTAGGGTTAGGGTTAGGGTTTAGGGTTTAGGGTTTAGGGGTTTAGGGTTTAGGGTTTAGGGTTTAGGGTTAGGGTTTAGGGTTTTAGGGTTAGGGTTTAGGGTTAGGGTTAGGGTTTAGGGTTTAGGGTTTAGGGTTTAGGTTTAGGGTTTAGGGTTTAGGGTTTAGGGTTAGAGGTTTAGGTTTAGGGTTTAGGGTTTAGGGTTTAGGGTTTAGGGGTTTAGGGTTAGGGTTTAGGGTTTGGGTTTAGGGTTTAGGGTTAGGTTTAGGGTTTAGGGTTTTAGGGTTTAGGGGTTTAGGGTTTAGGGGTTTAGGGTTTAGGGTTAGGGTTTAGGTTTAGGGGTTTAGGGTTAGGGTTTAGGGTTTAGGTTTAGGGTTTAGGGTTTAGGGTTTAGGGTTTAGGGTTTAGGGTTTTTAGGGTTTAGGGTTTAGGGTTTAGGGTTTAGGGTTTAGGGTTTAGGGTTTTAGGGTTTAGGGTTTAGGGTTTTAGGGTTTAGGGTTTAGGGTTTAGGGTTAGGGTTTAGGGTTTAGGGTTTAGGGTTTAGGGTTTAGGGTTTCTTAGGGTTTAGGGTTTGGGTTTTAGGGTTTAGGGTTAGGGTTTAGGGTTTAGCGTTTGGGTTAGGGTTTAGGGTTTTTAGGGTTTTAGGTTTAGGGTTTAGGGTTTAGGGTTTAGGGTTTAGGGTTAGGGTTTAGGGTTAGGGTTTAGGGTTTAGGGTTTAGGGTTTAGGGTTTAGGGTTTAGGGTTTAGGGTTTAGGGTTTAGGGTTTTAGGGTTTAGGGTTAGGGTTTAGGGATTGTTAGGGTTTAGGGTTTAGGGTTTAGGGTTTAGGGTTTAGGGTTTAGGGTTTAGGGTTAGGGTTTAGGGTTTAGGGTTTAGGGTTTAGGGTTTAGGGTTTAGGGTTTTAGGGTTTAGGGTTTAGGGTTTAGGGTTTAGGGTTTAGGGTTTAGGGTTAGGGTTTAGGGTTTAGGGTTTAGGGTTTAGGGTTTAGGGTTTAGGGTTTAGGGTTTAGGGTTTAGGGTTTAGGGTTTAGGGTTTAGGGTTTAGGTTTAGGGTTTAGGGTTTAGGGTTTAGGGTTTAGGGGTTTAGGGTTTAGGGTTAGGGTTAGGGTTTAGGGTTTAGGGTTTAGGGTTAGGTTTAGGGTTTAGGGTTTAGGGTTTAGGGTTTAGGGTTTAGGGGTTTAGGGTTTAGGGTTTAGGTTAGGGTTTAGGGTTTTAGGGTTTAGGGTTAGGGTTTACGGGTTACGGGTTTAGGGTTTAGGGTTTAGGGTTTAGGGTTAGGTTTAGGGTTTAGGGTTAGGGTTTAGGGTTTAGGGTTTAGGGTTTAGGGTTTAGGGTTTAGGGTTTAGGGTTTAGGGTTTAGGGTTTAGGGGTTAGGGTTTAGGGTTTAGGGTTTAGGGTTTAGGGTTTAGGGTTTAGGGTTTAGGGTTTAGGGTTTAGGGTTAGGGTTAGGGTTTAGGGTTTAGGGTTTAGGGTTTAGGGTTAGGGTTTAGGGTTTAGGGTTTAGGGTTTAGGGTTAGGGTTTAGGGTTTAGGGTTTAGGTTTAGGGTTTAGGGTTTAGGGTTTAGGGTTTAGGGTTTAGGGTTTAGGGTTAGGGTTAGGGTTTAGGGTTTAGGGTTTAGGGTTTAGGGTTTAGGGTTAGGGTTTAGGGTTTAGGGTTTAGGTTAGGGTTTAGGGTTTAGGGTTTAGGGTTTAGGGTTTAGGGTTAGGGTTTAGGGTTTAGGGTTTAGGGTTTAGGGTTTAGGGTTTAGGGTTTAGGGTTTAGGTTTAGGGTTTAGGGTTTAGGGTTTAGGGTTTAGGGTTTAGGGGTTTAGGGGTTTAGGGTTTAGGGTTTAGGGTTTAGGGTTTAGGGTTTAGGGTTTAGGGTTTAGGGTTTAGGGTTTAGGGTTTAGGGTTTAGGGTTTAGGGTTTAGGGTTTAGGTTTAGGGTTTAGGGTTTAGGGTTAGGGTTTAGGGTTTAGGGTTAGGGTTTAGGGTTTAGGGTTAGGGTTTAGGGTTTAGGGTTTAGGGTTTAGGGTTTAGGGTTTAGGGTTTAGGGTTTAGGGTTAGGGTTTAGGGTTTAGGGTTTAGGGTTTAGGGTTAGGGTTTAGGGTTTAGGGTTTAGGGTTTAGGGTTAGGGTTTAGGGTTTAGGGTTAGGGTTTAGGGTTAGGTTGGGTTTAGGGTTTAGGGTTAGGGTTTAGGGTTTAGGGTTTAGGGTTTAGGGTTTAGGGTTTAGGGTTTAGGGTTTAGGGTTTAGGGTTTAGGGTTTAGGGTTTAGGGGTTTAGGGTTTAGGGTTTAGGTTTAGGGTTTAGGGTTTAGGGTTTAGGGTTTAGGGTTTAGGGTTAGGGTTTAGGTTTAGGGTTTAGGGTTAGGGTTTAGGGTTTAGGGTTTAGGGTTTAGGGTTAGGGTTAGGGTTTAGGGTTTAGGGTTTAGGGTTTAGGGTTTAGGGTTTAGGGTTTAGGGTTTAGGTTTAGGGTTTAGGGTTTAGGGTTTAGGGTTTAGGGTTTTAGGGTTTAGGGTTTAGGGTTTAGGGTTTAGGGTTTAGGGTTAGGGTTTAGGGTTTAGGGTTAGGGTTAGGGTTTAGGGTTAGGGTTAGGGTTTAGGGTTTAGGGTTTAGGGTTTAGGGTTTAGGGTTTAGGGTTTAGGGTTTAGGGTTAGGGTTTAGGGTTTAGGGTTTAGGGTTTAGGGTTTAGGGTTTAGGGTTTAGGTTTAGGGTTTAGGGTTAGGGTTAGGGTTTAGGGTTTTAGGGTTTAGGGTTTAGGGTTTAGGGTTTAGGGTTTAGGGTTTAGGGTTTAGGGTTTAGGGTTTAGGGTTTAGGTTTAGGGTTTAGGGTTTAGGGTTTAGGTTTAGGGTTTAGGGTTTTAGGGTTTAGGGTTTAGGGTTTTAGGGTTTAGGGTTAGGGTTTAGGGTTAGGGTTTAGGGTTTAGGGTTTAGGGTTTAGGGTTAGGGTTTAGGGTTTAGGGTTTAGGGTTTAGGGTTTAGGGTTTTAGGGTTTAGGGTTTAGGGTTTTAGGGTTTAGGGTTTAGGGTTTAGGGGTAGGTTTAGGGTTTAGGGTTTAGGGTTTAGGGTTTAGGGTTTAGGGTTTAGGGTTTAGGGTTTAGGGTTTAGGGTTTAGGGTTTAGGGTTTAGGGTTTAGGGTTAGGGTTTAGGGTTTAGGGGTTTAGGGTTTAGGGTTTAGGTTCTTAGGGTTTAGGGTTTAGGGTTTAGGTTTAGGGTTTAGGGTTTAGGGGTTTAGGTTTAGGGTTTAGGGTTTAGGGTTTAAGGGTTTAGGGTTTAGGGTTTAGGGTTTAGGGTTAGGGTTTAGGGTTTAGGGTTTAGGGTTTAGGGGTTTAGGGTTTAGGGTTTAGGGTTTAGGGTTTAGGGTTTAGGGTTTAGGGTTAGGGTTTAGGGTTTAGGGTTAGGGTTTAGGGTTTAGGGTTTAGGGTTTAGGGTTTAGGGTTTAGGGTTTAGGGTTTAGGGTTTAGGGTTTAGGGTTTAGGGTTTAGGGTTTAGGGTTTAGGGTTAGGGTTTAGGGTTTAGGGTTTAGGGTTTAGGGTTAGGTTTAGGGTTAGGGTTAGGGTTAGGGTTTTAGGGTTAGGTTTAGGGGTTTAGGGTTTAGGGTTTAGGGTTTAGGGTTTAGGGTTTAGGGTTTAGGGTTTAGGGTTTAGGGTTTAGTGTTAGGGTTTAGGGTTTAGGGTTTAGGGTTAGGGTTTAGGGTTTAGGGTTTAGGGTTTAGGGTTTAGGGTTTTAGGGTTTAGGGTTTAGGGTTAGGGTTTAGGGTTTAGGGTTTAGGTTTAGGGTTTAGGGTTTAGGGTTTAGGGTTTAGGGTTTAGGGTTTAGGGTTGGTTTAGGGTTAGGGTTTAGGGTTTAGGGTTTAGGGTTAGGGGTTTAGGGTTAGGGTTTAGGGTTAGGGTTAGGGGTTTAGGGTTTAGGGTTTAGGGTTAGGGTTTAGGGTTTAGGGTTTAGGGTTTAGGGTTTAGGGTTTTAGGGTTAGGGTTTAGGGTTTAGGGTTTAGGGTTTAGGGTTTAGGGTTTAGGGTTTAGGGTTTAGGGTTAGGGTTTAGGGTTTAGGGTTTAGGGTTTAGGGTTTAGGGTTTAGGTTTAGGGTTTAGGGTTTAGGGTTTAGGGTTTAGGGTTTAGGGTTTAGGGTTTAGGGTTAGGGTTTAGGGTTTAGGGTTTAGGGTTTAGGGTTTAGGGTTTAGGGTTAGGGTTTAGGGTTTAGGGTTTAGGGTTTAGGGTTTAGGGTTAGGGTTTAGGGTTTAGGGTTTAGGGTTTAAGGGTTTAGGGTTTAGGGGTTAGGGTTTAGGGTTTAGGGTTTAGGGTTTAGGGTTTAGGGTTTAGGGTTTAGGTGGTTTAGGGTTTAGGGTTAGGGTTTAGGGTTTAGGGTTTAGGGTTTAGGGTTAGGGTTTAGGGTTTAGGGTTTAGGGTTTAGGGTTTTAGGGTTTAGGGTTTAGGGTTTAGGGTTTAGGGTTAGGGTTATAGGTGGTTTAGGGTTTAGGGTTTAGGGTTTAGGGTTTAGGGTTTAGGGTAGGGTTTAGGGTTTAGGGTTTAGGGTAGTAGGGTTTAGGGTTTAGGGTTTAGGGGGTTTAGGGTTTAGGGTTAGGTGGTTTTAGGGTTTAGGGTTTAGGGTTTAGGGTTTAGGGGTTTAGGGTTAGGGTTTAGGGTTTAGGGTTTAGGGTTTAGGGTTTAGGGTTTAGGGTTAGGGTTTAGGGTTGTAGGGTTTTAGGGTTTAGGGTTTAGGGTTTAGGGTTTAGGGTTTAGGGTTTAGGGTTTAGGGTTTAGGGTTTAGGGTTAGGGTTTAGGGTTTTAGGGTTTAGGGTTTAGGTTAGGGTTTAGGGTTTAGGGTTAGGGTTTAGGGTTTAGGGTTTAGGGTTTAGGGTTAGGGTTTAGGGTTTAGGGTTAGGGTTAGGTTTAGGGTTTAGGGTTTTAGGGTTTAGGGTTTAGGGTTTAGGGTTTAGGGTTTAGGGTTTAGGGGTTTAGGGTTTAGGGTTTAGGGTTTTAGGGTTTAGGGTTTAGGGTTTGGGTTTAGGGTTTAGGGTTTTAGGGTTTAGGGTTTAGGGTTAGGGTTTAGGGTTTAGGGTTTAGGGTTTAGGGTTTAGGTTAGGGTTTAGGGTTTAGGGGGTTTAGGGTTTAGGGTTTAGGGTTTAGGGTTAGGGTTTAGGGTTTAGGGTTTAGGGTTTAGGGTTTAGGGTTTAGGGTTTAGGGTTTTAGGGTTTAGGGTTTTAGGGTTTAGGGTTTAGGGTTTAGGGTTTAGGGTTTAGGGTTTAGGGTTTTGGGTTAGGGTTTAGGGTTTAGGGTTTAGGGTTTAGGGTTTAGGGGTTTAGGGTTTAGGGTTTAGGGTTTAGGGTTTAGGGTTTTAGGGTTTAGGGTTTAGGGTTTAGGGTTTAGGGTTTAGGGTTTAGGGTTTAGGGTTTAGGGTTTAGGGTTTAGGGTTTAGGGTTTAGGGTTTTAGGGTTTAGGGTTTTAGGGTTTAGGGATTTTAGGGTTTAGGGTTAGGGTTAGGGTTTAGGGTTTAGGGTTTAGGGTTTAGGGTTGGGTTTAGGGTTTAGGGTTTAGGGTTTAGGGTTTAGGTTAGGGTTTGGGTTAGGGTTTAGGGTTTAGGGTTTAGGGGTTTAGGGTTTAGGGTTTAGGGTTTAGGGTTTAGGGTTTAGGGTTTAGGGTTTAGGGTTTAGGGTTTAGGGGTTTAGGGAGGTTAGGGTTTAGGGTTTAGGAGTTTTAGGGTTAGGGTTTAGGGTTTAGGTGGTTTTAGGGTTTAGGGTTTAGGGTTTGTAGGGTTTAGGGTTTAGGGTTTTAGGGTTTAGGGTTTAGAGGGTAGTTTAGGGTTTAGGGTTTAGGGTTTAGGGTTAGGGTTTAGGGTTTTAGGGTTTAGGGTTTAGGGTTTAGGGTTTAGGGTTTAGGGTTTAGGGTTTAGGGTTTAGGGTTTAGGGTTTAGGGTTTAGGGTTAGGGTTTAGGGTTTAGGTTTAGGGTGTTAGGGTTTTAGGGGTTAGGGTTTAGGGTTTAGGGTTTAGGGTTTAGGGTTTAGGGTTTAGGGTTTAGGGCTTTAGGTTTAGGGGTTTAGGGTTTAGGGGTTAGGGTTTAGGGTTTAGGGTTTAGGGTTTAGGGTTTTAGGGTTTAGGGTTTAGGGTTGTAGGGTTTAGGTTTAGGGTTAGGGTTTAGGGTTTAGGTTTAGGGTTTAGGG

***T.aestivum* var.Mace**

**4AS**

GGG**TTTAGGG**TTTAGGGTAAGGGTTTGGGTTTAGGGTTTATGTTTTAGGGTTTAGGGTTTAGGGTTTAG

**4BS**

G**TTTAGGG**TTTAGGGTTTAGGGTTAGGGTTTAGGGTTTAGGGTTTAGGGTTTAGGGTTTAGGGTTTAGGG

**7BS**

GGAATAGG**TTTAGGG**TTTAGGGTTTAGGGTTTTTAGGCTTTAGGGTTTAGGGTTTAGGGTTTAGGGTTTTTAGGGTTTAGGGTTTAGGGTTTAGGGTTTACGGTTTAGGGTTTAGGGTTTAGGG

***T.aestivum* var.Jagger**

**2AS**

AGGG**TTTAGGG**TTTAGGGTTTAGGGTTTAGGGTTTAGGGTTAGGGTTTAGGAGT

***T.aestivum* var.Julius**

**4AS**

GGG**TTTAGGG**TTTAGGGTAAGGGTTTGGGTTTAGGGTTTATGTTTTAGGGT

**7BS**

GAATAGG**TTTAGGG**TTTAGGGTTTAGGGTTTTTAGGCTTTAGGGTTTAGGGTTTAGGGTTTAGGGTTTTTAGGGTT

***T.aestivum* var.Kariega**

**1AS**

GTAGTTTCG**TTTAGGG**TTTAGGGTTTAGGGTTTAGGGTTTAGGGTTTAGGGTTTAGGGTTTAGGGTTTAGGGTTTAGGGTTTAGGGTTTAGGGTTTAGAGTTAGGGTTTAGGGTTTAGGGTTAGGGTTTAGGGTTTAGGGTTTAGGGTTAGGGTTTAGGGTTTAGGGTTTAGGGTTAGGGTTTAGGGTTTAGGGTTTAGGGTTTAGGGTTTAGGGTTTAGGGTTAGGGTTTAGGGTTTAGGGTTTAGGGTTTAGGGTTTAGGGTTTAGGGTTAGGTTAGGGTTTAGGGTTTAGGGTTTAGGGTTTAGGGTTTAGGGTTTAGGGTTTAGGGTTTAGGGTTTAGGGTTAGGGTTTAGGGTTTAGGGTTTAGGGTTTAGGGTTTAGGGTTTAGGGTTTAGGGTTTAGGGTTTAGGGTTTAGGGTTTAGGGTTTAGGGTTTAGGGTTGGGTTTAGGGTTTAAGGGTTTGGGGTTTAGGGTTTTAGGGTTTTAGGGTTTTTAGGGTTTAGGGTTTGGGTTTAGAGTTGGGGTTTGGGTTTTAGGGTTTAGGGTTGGGTTAGGGTTTAGGGTTTAGGGTTTAGGGTTTAGGGTTAGGGTTTAGGGTTTAGGGTTAGGGTTTAGGGTTAGGGTTGTTTTAGGGTTTTTTAGGGTTTAGGGTTAGGGTTTAGGGTTTAGGGTTTAGGGTTAGGGTTAGGGTTAGGGTTAGGGTTTAGGGTTTAGGGTTTAGGGTTTAGGGTTTAGGGTTTAGGGTTTTTTAGGGTTTTTAGGGTTAGGGTTTAGGGTTTTAGGGTTTAGGGTTTAGGGTTTAGGGTTTAGGGTTTAGGGTTAGGGTTTAGGGTTTTGGGGTTGGGTTTAGGGTTTAGGGTTTAGGGTTAGGGTTTAGGGTTTTAGGGTTTAGGGTTAGGGTTTAGGGTTTAGGGTTTAGGGTTTAGGGTTAGGGTTTAGGGTTTTAGGGTTTTAGGGTTTAGGGTTTAGGGTTTAGGGTTAGGGTTTAGGGTTTAGGGTTTTAGGGTTTAGGGTTAGGGTTTAGGGTTTAGGGTTTGGTTAGGGTTTAGGGTTTAGGGTTTAGGGTTTAGGGTTAGGGTTAGGGTTTAGGGTTTAGGGTTGGTTAGGGTTAGGGTTTAGGGTTAGGGTTAGGGTTTAGGGTTTAGGGTTTAGGGTTTAGGGTTTAGGGTTTGGGGTTAGGGTTTAGGGTTTAGGGTTAGGGTTTAGGGTTTAGGGTTTAGGGTTTAGGGTTAGGGTTTAGGGTTTAGGGTTTAGGGTTTAGGGTTAGGGTTTAGGGTTAGGGTTTAGGGTTTAGGGTTTTAGGGTTTTAGGGTTTAGGGTTTAGGGTTTAGGGTTAGGGTTTAGGGTTTTAGGGTTTAGGGTTTTAGGGTTTTAGGGTTTAGGGTTAGGGTTAGGGTTTTTAGGGTTGTTTTAGGGTTTTAGGGTTTAGGGTTTTAGGGTTTTAGGGTTTAGGGTTTAGGGTTTAGGGTTTAGGGTTTAGGGTTTAGGGTTTAGGGTTTAGGGTTTAGGGTTTAGGGTTTAGGGTTTAGGGTTTAGGGTTTAGGGTTTAGGGTTTAGGGTTAGGGTTTAGGGTTAGGGTTTAGGGTTTAGGGTTAGGGTTTAGGGTTTAGGGTTTAGGGTTTTAGGGTTTAGGGTTTAGGGTTTAGGGTTTAGGGTTTAGGGTTTAGGGTTTAGGGTTTAGGGTTTAGGGTTTAGGGTTTTAGGGTTTAGGGTTTAGGGTTTAGGGTTTAGGGTTTAGGGTTTAGGGTTTAGGGTTTAGGGTTTAGGGTTTAGGGTTTAGGGTTTAGGGTTTAGGGTTTAGGGTTAGGGTTGGGTTTAGGGTTTAGGGTTTAGGGTTTAGGGTTTAGGGTTTAGGGTTGGTTTAGGGTTAGGGTTTAGGGTTAGGGTTTAGGGTTTAGGGTTTAGGGTTTAGGGTTTAGGGTTTTGGGTTTAGGGTTTAGGGTTTAGGGTTTAGGGGTTTAGGGTTTAGGGTTTAGGGTTTAGGGTTTAGGGTTTAGGGTTTAGGGTTTAGGGTTAGGGTTTAGGGTTTAGGGTTAGGGTTTAGGGTTTAGGGTTTAGGGTTTAGGGTTAGGTTAGGGTTTAGGGTTTAGGGTTTAGGGTTTAGGGTTTAGGGTTTAGGGTTTAGGGTTTAGGGTTTAGGGTTTAGGGTTTAGGGTTTAGGGTTTAGGGTTAGGGTTTAGGGTTTAGGGTTTAGGGTTAGGGTTTAGGGTTTAGGGTTTAGGGTTTAGGGTTTAGGGTTTAGGGTTTAGGGTTTAGGGTTAGGGTTTAGGGTTTAGGGTTTAGGGTTTAGGGTTTAGGTTTAGGGTTTAGGGTTTAGGGTTTAGGGTTTTAGGGTTTTAGGGTTTAGGGTTTAGGGTTTAGGGTTTAGGGTTTAGGGTTTAGGGTTTAGGGTTTAGGGTTTTAGGGTTTAGGGTTTAGGGTTTAGGGTTTAGGGTTTAGGGTTTAGGGTTTAGGGTTTAGGGTTTAGGGTTTAGGGTTAGGGTTTAGGGTTTAGGGTTTAGGGTTTAGGGTTAGGGTTTAGGGTTTAGGGTTTAGGGTTTAGGGTTTAGGGTTTAGGGTTAGGGTTTAGGGTTGTAGGGTTTAGGGTTTAGGGTTAGGGTTAGGGTTTGGGTTTAGGGTTTAGGGTTTGGGTTTAGGGTTTAGGGTTTAGGGTTTAGGGTTTAGGGTTTAGGGTTTAGGGTTAGGGTTTAGGGTTTAGGGTTTAGGGTTTAGGGTTTAGGGTTTAGGGTTAGGGTTTAGGGTTTAGGGTTTAGGGTTAGGTTTAGGGTTTAGGGTTTAGGGTTTTAGGGTTTAGGGTTTAGGGTTTAGGGTTTTAGGGTTTAGGGTTTAGGGTTTTTAGGGTTTGTTAGGGTTAGGGTTTAGGGGTTTAGGGTTTAGGGTTTAGGGTTGGTAGGGTTTAGGGTTTAGGGTTTAGGGTTAGGTTAGGGTTTAGGGTTTAGGGTTTAGGGTTTAGGGTTTTAGGGTTTAGGGTTTAGGGTTTAGGGTTTAGGGTTTAGGGTTTAGGGTTAGGGTTTAGGGTTTAGGGTTAGGGTTAGGTTTAGGGTTTAGGGTTAGGGTTTAGGGTTTAGGGGTTTAGGGTTTAGGGTTTAGGGTTTAGGGTTTAGGGTTAGGGTTTAGGGTTTAGGGTTTAGGGTTTAGGGTTTAGGGTTTAGGGTTTAGGGTTTAGGGTTAGGGTTTAGGGTTTAGGGTTAGGGTTTAGGGTTTAGGGTTTAGGGTTTAGGGTTTAGGTTGGTTAGGGTTTAGGGTTTAGGGTTTAGGGTTTAGGGTGTTAGGGTTTAGGGTTTAGGGTTTAGGGTTTTAGGGTTTAGGGTTAGGGTTTAGGGTTTAGGGTTAGGGTTTAGGGTTTAGGGTTAGGGTTTAGGGTTTAGGGTTTAGGGTTTAGGGTTTAGGGTTAGGGTTTAGGGTTTAGGGTTTAGGGTTAGGGTTTAGGGTTTAGGGTTTAGGGTTTAGGGTTTAGGGTTAGGGTTTAGGGTTTAGGGTTTAGGGTTTAGGGTTAGGGTTTAGGGTTTAGGGTTTAGGGTTTAGGGTTTAGGGTTTAGGGTTTAGGGTTTAGGGTTTAGGGTTTAGGGTTTAGGGTTTAGGGTTTAGGGTTTAGGGTTTAGGGTTTAGGGTTTAGGGTTTAGGGTTTAGGGTTTAGGGTTTAGGGTTTAGGGTTTAGGGTTTAGGGTTTAGGGTTTAGGGTTTAGGGTTTAGGGTTTAGGGTTTAGGGTTTAGGGTTTAGGGTTTAGGGTTTAGGGTTTAGGGTTTAGGGTTTAGGGTTTAGGGTTTAGGGTTTTAGGGTTAGGTTAGGGTTTAGGGTTTAGGGTTTAGGGTTTAGGGTTTAGGGTTTAGGGTTAGGGTTTAGGGTTGTTAGGGTTTAGGGTTTAGGGTTTAGGGTTTAGGGTTTAGGGTTTAGGGTTTAGGGTTTAGGGTTTAGGGTTAGGGTTTAGGGTTTAGGGTTTTTAGGGTTAGGGTTTAGGGTTTAGGGTTTAGGGTTTAGGGTTTAGGGTTTAGGGTTAGGGTTTAGGGTTTAGGGTTTAGGGTTAGGGTTTAGGGTTTAGGGTTTAGGGTTTAGGGTTAGGGTTTAGGGTTTAGGGTTTAGGGTTTAGGGTTTAGGGTTTAGGGTTAGGGTTTAGGGTTTAGGGTTTAGGGTTTAGGGTTTAGGGTTTAGGGTTTAGGGTTTAGGGTTTAGGGTTTAGGGTTTAGGGGTTTAGGGTTTAGGGTTTAGGGTTTAGGGTTTAGGGTTTAGGGTTTTAGGGTTTAGGGTTTAGGGTTTAGGGTTTAGGGTTTAGGGTTTAGGGTTTAGGGTTTAGGGTTTAGGGTTTAGGGGTTTAGGGTTTAGGGTTTAGGGTTTAGGGTTTAGGGTTTAGGGTTTAGGGTTTAGGGTTTAGGGTTTAGGGTTTAGGGTTTAGGGTTTAGGGTTTAGGGTTTAGGGTTTAGGGTTTAGGGTTAGGGTTTAGGGTTTAGGGTTTAGGGTTTAGGGTTTAGGGTTTAGGGTTAGGGTTTAGGGTTTAGGGTTTAGGGTTTAGGGTTTAGGGGTTTAGGGTTAGGGTTTAGGGTTTAGGGTTTAGGGTTAGGGTTTAGGGTTTAGGGTTTAGGGTTTAGGGTTTAGGGTTTAGGGTTTAGGGTTTAGGGTTTAGGGTTTAGGGTTTAGGGTTTAGGGTTTAGGGTTTAGGGTTTAGGGTTTAGGGTTTAGGGTTTAGGGTTTAGGGTTTAGGGTTTAGGGTTTAGGGTTTAGGGTTTAGGGTTTAGGGTTTAGGGTTTAGGGTTAGGGTTTAGGGTTTAGGGTTTAGGGTTTAGGGTTTAGGGTTTAGGGTTTAGGGTTTAGGGTTTAGGGTTTAGGGTTTAGGGTTTAGGGTTAGGGTTTAGGGTTTAGGGTTTAGGGTTTAGGGTTTAGGGTTAGGGTTAGGGTTTAGGGTTTAGGGTTTAGGGTTAGGGTTTAGGGTTTAGGGTTTAGGGTTTAGGGTTTAGGGTTTAGGGTTTAGGGTTTAGGGTTAGGGTTTAGGGTTTAGGGTTTAGGGTTTAGGGTTTAGGGTTAGGGTTAGGGTTTAGGGTTTAGGGTTAGGGTTAGGGTTTAGGGTTAGGGTTAGGTTTAGGGTTTAGGGTTTAGGGTTTAGGGTTTAGGGTTTAGGGTTTAGGGTTTAGGGTTTAGGGTTTAGGGTTTAGAGGTTAGGGTTTAGGGTTTAGGGTTTAGGGTTTAGGGTTAGGGTTTAGGGTTTAGGGTTTAGGGTTTAGGGTTTAGGGTTTAGGGTTTAGGGTTAGGGTTTAGGGTTTAGGGTTTAGGGTTTAGGGTTAGGGTTTAGGGTTTAGGGTTTAGGGTTTAGGGTTTAGGGTTTAGGGTTTAGGGTTTAGGGTTTAGGGTTTAGGGTTTAGGGTTTAGGGTTTAGGGTTAGGGTTTAGGGTTTAGGGTTTAGGGTTTAGGGTTTAGGGTTTAGGGTTTAGGGTTTAGGTTTAGGGTTTAGGGTTAGGGTTTAGGGTTTAGGGTTTAGGGTTTAGGGTTAGGGTTTAGGGTTTAGGGTTTAGGGTTTAGGGTTTAGGGTTTAGGGTTTAGGGTTTAGGGTTTAGGGTTTAGGGTTTAGGGTTTAGGGTTAGGGTTTAGGGTTTAGGGTTTAGGGTTTAGGGTTTAGGGTTTAGGGGTTTAGGGTTTAGGGTTTAGGGTTTAGGGTTTAGGGTTTAGGGTTTAGGGTTTAGGGTTAGGGTTTAGGGTTTAGGGTTTAGGGTTTAGGGTTAGGGTTTAGGGTTTAGGGTTTAGGGTTTAGGGTTTAGGGTTTAGGGTTAGGGTTTAGGGTTTAGGGTTTAGGGTTTAGGGTTTAGGGTTTAGGGTTAGGGTTTAGGGTTTAGGGTTTAGGGTTTAGGGTTTAGGGTTTAGGGTTTAGGGTTAGGGTTTAGGGTTTAGGGTTAGGGTTTAGGGTTTAGGGTTTAGGGTTTAGGGTTTAGGGTTTAGGTTTAGGGTTTAGGGTTTAGGGTTTAGGGTTAGGGTTAGGGTTTAGGGTTTAGGGTTTAGGGTTTAGGGTTTAGGGTTTAGGGTTTAGGGTTTAGGGTTTAGGGTTTAGGTTTAGGGTTTAGGGTTAGGGTTAGGGTTAGGGTTTAGGGTTTAGGGTTTAGGGTTTAGGGTTTAGGGTTTAGGGTTTAGGGTTAGGGTTAGGGTTTAGGGTTTAGGGTTTAGGGTTTAGGGTTAGGGTTTAGGGTTTAGGGTTTAGGGTTTAGGGTTTAGGGGTTTAGGGTTTAGGGTTTAGGGTTTAGGGTTTAGGGTTTAGGGTTTAGGGTTTAGGGTTTAGGGTTTAGGGTTAGGGTTTAGGGTTTAGGGTTTAGGGTTAGGGTTTAGGGTTTAGGGTTTAGGGTTTAGGGTTTAGGGTTTAGGGTTTAGGGTTAGGGTTAGGGTTAGGGTTTAGGGTTTAGGGTTAGGGTTTAGGGTTTAGGGTTTAGGGTTTAGGGTTTAGGGTTTAGGGTTTAGGGTTAGGGTTTAGGGTTTAGGGTTTAGGGTTTAGGGTTTAGGTTTAGGGTTTAGGGTTTAGGGTTTAGGGTTTAGGGTTTAGGGTTTAGGGTTTAGGGTTTAGGGTTTAGGGTTTAGGGTTTAGGGTTTAGGGTTTAGGGTTTAGGGTTTAGGGTTTAGGGTTTAGGGTTTAGGGTTTAGGGTTTAGGGTTTAGGGTTTAGGGTTAGGGTTTAGGGTTTAGGGTTTAGGGTTTAGGGTTAGGGTTTAGGGTTTAGGGTTAGGGTTTAGGGTTTAGGGTTTAGGGTTTAGGGTTTAGGGTTAGGGTTTAGGGTTTAGGGTTTAGGGTTTAGGGTTTAGGGTTTAGGGTTTAGGGTTTAGGGTTTAGGGTTTAGGGTTAGGGTTTAGGGTTTAGGGTTTAGGGTTTAGGGTTTAGGGTTTAGGGTTAGGGTTTAGGGTTTAGGGTTAGGGTTTAGGGTTTAGGGTTTAGGGTTTAGGGTTTAGGGTTTAGGGTTTAGGGTTTAGGGTTTAGGGTTTAGGGTTTAGGGTTAGGGTTTAGGGTTTAGGGTTTAGGGTTTAGGGTTTAGGGTTTAGGTTTAGGGTTTAGGGTTAGGGTTTAGGGTTTAGGGTTTAGGGTTTAGGGTTTAGGGTTTAGGGTTTAGGGTTTAGGTTTAGGGTTTAGGGTTTAGGGTTTAGGGTTTAGGGTTAGGGTTTAGGGTTTAGGGTTTAGGGTTAGGGTTTAGGGTTTAGGGTTTAGGGTTTAGGGTTTAGGGTTTAGGTTTAGGGTTTAGGTTTAGGGTTTAGGGTTTAGGGTTTAGGGTTTAGGGTTTAGGGTTTAGGGTTTAGGGTTTAGGGTTTAGGGTTTAGGGTTTAGGGTTTAGGGTTTGGTTTAGGGTTTAGGGTTTAGGGTTAGGGGTTAGGGTTTAGGGTTTAGGGTTTAGGGTTTAGGGTTTAGGGTTAGGGTTTAGGGTTTAGGGTTTAGGGTTTAGGGTTTAGGGTTTAGGGTTTAGGGTTTAGGGTTAGGTTTAGGGTTTAGGGTTTAGGGTTTAGGGTTAGGGTTTAGGGTTTAGGGTTTAGGGTTTAGGGTTTAGGGTTTAGGGGTTTAGGTTTAGGGTTTAGGGTTTAGGGTTTAGGGTTTAGGTTTAGGGTTTAGGGTTTAGGGTTTAGGGTTTAGGGTTTAGGGTTTAGGGTTTAGGGTTTAGGGTTAGGGTTAGGGTTTAGGGTTTAGGGTTTAGGGTTTAGGGTTTAGGGTTTAGGGTTTAGGGTTTAGGGTTTAGGGTTTAGGGTTTAGGGTTTAGGGTTTAGGGTTTAGGGTTTAGGGTTAGGTTTAGGGTTTAGGGTTTAGGGTTTAGGGTTTAGGGTTTAGGGTTTAGGGTTTAGGGTTAGGGTTTAGGGTTTAGGGTTTAGGGTTTAGGGTTTAGGGTTTAGGGTTTAGGGTTTAGGGTTTAGGGTTTAGGGTTTAGGGTTTAGGGTTTAGGGTTTAGGGTTAGGTTTAGGGTTTAGGGTTTAGGGTTTAGGGTTTAGGGTTTAGGGTTTAGGGTTTAGGGTTTAGGGTTTAGGGTTTAGGGTTTAGGGTTTAGGGTTTAGGGTTTAGGGTTTAGGTTTAGGGTTTAGGGTTTAGGGTTAGGGTTTAGGGTTTAGGGTTTAGGGTTTAGGGTTTAGGGTTTAGGGTTTAGGGTTTAGGGTTTAGGGTTTAGGGTTTAGGGTTTAGGGTTTAGGGTTTAGGGTTTAGGGTTTAGGGTTAGGGTTTAGGGTTTAGGGTTTAGGGTTTAGGGTTTAGGGTTTAGGGTTTAGGGTTTAGGGTTTAGGGTTTAGGTTTAGGGTTTAGGGTTTAGGGTTAGGGTTTAGGGTTTAGGGTTTAGGGTTTAGGGTTTAGGGTTTAGGGTTTAGGGTTTAGGGTTTAGGGTTTAGGGTTAGGGTTTAGGGTTTAGGGTTTAGGGTTTAGGGTTTAGGGTTTAGGGTTTAGGGTTTAGGGTTTAGGGTTAGGTTTAGGGTTTAGGGTTTAGGGTTTAGGGTTTAGGGTTTAGGGTTTAGGGTTTAGGGTTTAGGGTTTAGGGTTTAGGGTTTAGGGTTAGGGTTTAGGGTTTAGGGTTTAGGGTTTAGGGTTTAGGGTTTAGGGTTTAGGGTTTAGGGTTTAGGTTTAGGGTTTAGGGTTTAGGGTTTAGGGTTTAGGGTTAGGGTTAGGGTTTAGGGTTTAGGGTTTAGGGTTTAGGGTTTAGGGTTTAGGGTTTAGGGTTTAGGGTTTAGGGTTTAGGGTTTAGGGTTTAGGGTTTAGGGTTTAGGGTTTAGGGTTTAGGGTTTAGGGTTTAGGGTTTAGGGTTTAGGGTTTAGGGTTTAGGGTTTAGGGTTTAGGGTTTAGGGTTTAGGGTTTAGGGTTTAGGGTTAGGGTTTAGGGTTTTAGGGTTAGGTTAGGTTTAGGGTTTAGGGTTTAGGGTTTAGGGTTTAGGGTTTAGGGTTAGGGTTTAGGGTTTAGGGTTTAGGGTTTAGGGTTTAGGGTTTAGGGTTTAGGGTTTAGGGTTTAGGGTTTAGGGTTTAGGGTTTAGGGTTTAGGGTTTAGGGTTTAGGGTTTAGGGTTTAGGGTTTAGGGTTTAGGGTTTAGGGTTAGGGTTTAGGGTTTAGGGTTTAGGGTTTAGGGTTTAGGGTTTAGGGTTTAGGGTTTAGGGTTTAGGGTTTAGGGTTTAGGGTTTAGGGTTAGGGTTTAGGGTTTAGGTTAGGGTTTAGGGTTTAGGGTTTAGGGTTTAGGGTTTAGGGTTTAGGGTTTAGGGTTTAGGGTTTAGGGTTTAGGGTTTAGGGTTTAGGGTTTAGGGTTTAGGGTTTAGGGTTTAGGGTTTAGGGTTTAGGGTTTAGGGTTTAGGGTTTAGGGTTTAGGGTTTAGGGTTTAGGGTTTAGGGTTTAGGGTTTAGGGTTTAGGGTTTAGGGTTTAGGGTTTAGGGTTTAGGGTTTAGGGTTTAGGGTTTAGGGTTTAGGGTTTAGGGTTTAGGGTTTTAGGGTTTAGGGTTAGGGTTTAGGGTTTAGGGTTTAGGGTTTAGGGTTTAGGGTTTAGGGTTTAGGGTTTAGGGTTTAGGGTTTAGGTTTAGGGTTTAGGGTTTAGGGTTTAGGGTTTAGGGTTTAGGGTTTAGGGTTTAGGGTTTAGGGTTTAGGGTTTAGGGTTTAGGGTTTAGGGTTTAGGGTTTAGGGTTTAGGGTTTAGGGTTTAGGGTTTAGGGTTTAGGGTTTAGGGTTTAGGGTTTAGGGTTTAGGGTTTAGGGTTTAGGGTTTAGGGTTTAGGGTTTAGGGTTTAGGGTTTAGGGTTTAGGGTTTAGGGTTTAGGGTTTAGGGTTTAGGGTTTAGGGTTTAGGGTTTAGGGTTTAGGGTTTAGGGTTTAGGGTTTAGGGTTTAGGGTTTAGGGTTTAGGTTTAGGGTTTAGGGTTTAGGGTTTAGGGTTTAGGGTTTAGGGTTAGGGTTTAGGGTTTAGGGTTTAGGGTTTAGGTTTAGGGTTTAGGGTTTAGGGTTTAGGGTTTAGGGTTTAGGGTTTAGGGTTTAGGGTTTAGGGTTTAGGGTTTAGGGTTTAGGGTTTAGGGTTTAGGTTTAGGGTTTAGGGTTTAGGGTTTAGGGTTTAGGGTTTAGGTTTAGGGTTTAGGGTTTAGGGTTTAGGGTTTAGGGTTTAGGGTTTAGGGTTTAGGGTTTAGGGTTTAGGGTTTAGGGTTTAGGGTTTAGGGTTTAGGGTTTAGGGTTTAGGGTTTAGGGTTAGGGTTTAGGGTTTAGGGTTTAGGGTTTAGGGTTTAGGGTTAGGGTTTAGGGTTTAGGGTTTAGGGTTTAGGGTTAGGGTTTAGGGTTTAGGGTTTAGGGTTTAGGGTTAGGGTTTAGGGTTTAGGGTTTAGGGTTTAGGGTTTAGGGTTTAGGGTTTAGGGTTTAGGGTTTAGGGTTTAGGGTTTAGGGTTTAGGGTTTAGGGTTTAGGGTTTAGGGTTTAGGGTTTAGGGTTTAGGGTTTAGGGTTTAGGGTTTAGGGTTTAGGGTTTAGGGTTTAGGGTTTAGGGTTTAGGGTTAGGGTTAGGGTTTAGGGTTTAGGGTTTAGGGTTTAGGGTTTAGGGTTTAGGGTTTAGGGTTTAGGGTTAGGGTTTAGGGTTTAGGGTTTAGGGTTTAGGGTTTAGGGTTTAGGGTTTAGGGTTTAGGGTTTAGGGTTTAGGGTTTAGGGTTAGGGTTTAGGGTTTAGGGTTTAGGGTTTAGGGTTTAGGGTTTAGGTTTAGGGTTTAGGGTTTAGGGTTTAGGGTTTAGGGTTTAGGGTTTAGGGTTAGGTTTAGGGTTTAGGGTTTAGGGTTTAGGGTTTAGGGTTTAGGTTTAGGGTTTAGGGTTTAGGGTTTAGGGTTTAGGGTTTAGGGTTAGGGTTTAGGGTTTAGGGTTTAGGGTTTAGGGTTTAGGGTTTAGGGTTTAGGGTTTAGGGTTTAGGGTTTAGGGTTTAGGGTTTAGGGTTTAGGTTTAGGGTTTAGGGTTTAGGGTTTAGGGTTTAGGTTAGGGTTTAGGGTTTAGGGTTTAGGGTTTAGGGTTTAGGGTTTAGGGTTTAGGGTTTAGGGTTAGGGTTTAGGGTTTAGGGTTTAGGGTTTAGGGTTTAGGGTTTAGGTTTAGGGTTTAGGGTTTAGGGTTTAGGGTTAGGGTTTAGGGTTTAGGGTTTAGGGTTTAGGGTTTAGGGTTTAGGGTTTAGGGTTTAGGGTTTAGGGTTTAGGGTTTAGGGTTTAGGGTTTAGGGTTTAGGGTTTAGGGTTTAGGGTTTAGGGTTTAGGGTTTAGGGTTTAGGGTTTAGGGTTTAGGGTTTAGGGTTTAGGGTTTAGGGTTTAGGGTTTAGGGTTAGGGTTTAGGGTTTAGGGTTTAGGGTTTAGGGTTTAGGGTTTAGGGTTTAGGGTTTAGGGTTTAGGGTTTAGGGTTTAGGGTTTAGGGTTTAGGGTTTAGGGTTTAGGGTTTAGGGTTTAGGGTTTAGGGTTTAGGGTTTAGGGTTTAGGGTTTAGGGTTTAGGGTTTAGGGTTTAGGGTTTAGGTTTAGGGTTTAGGGTTTAGGGTTTAGGGTTTAGGGTTTAGGGTTTAGGGTTTAGGGTTTAGGGTTTAGGGTTTAGGGTTTAGGGTTTAGGGTTTAGGGTTTAGGGTTTAGGGTTTAGGGTTTAGGGTTTAGGGTTTAGGGTTTAGGGTTTAGGGTTTAGGGTTTAGGGTTTAGGGTTTAGGGTTTAGGGTTTAGGGTTTAGGGTTTAGGGTTTAGGGTTTAGGGTTTAGGGTTTAGGGTTTAGGGTTTAGGGTTTAGGGTTTTAGGGTTTAGGGTTTAGGGTTTAGGGTTTAGGGTTTAGGGTTTAGGGTTTAGGGTTTAGGGTTAGGGTTTAGGGTTTAGGGTTTAGGGTTTAGGGTTTAGGGTTTAGGGTTTAGGGTTTAGGGTTTAGGGTTTAGGGTTTAGGGTTTAGGGTTTAGGGTTAGGGTTTAGGGTTTAGGGTTTAGGGTTTAGGGTTTAGGGTTTAGGGTTTAGGGTTTAGGGTTTAGGGTTAGGGTTTAGGGTTTAGGGTTTAGGGTTTAGGGTTTAGGGTTAGGGTTTAGGGTTTAGGGTTTAGGGTTTAGGGTTTAGGGTTTAGGGTTTAGGGTTTAGGGTTTAGGGTTTAGGGTTTAGGGTTTAGGGTTTAGGGTTTTAGGGTTTAGGGTTTAGGGTTTAGGGTTTAGGGTTAGGGTTTAGGGTTTAGGGTTTAGGGTTTAGGGTTTAGGGTTTAGGGTTTAGGGTTTAGGGTTTAGGGTT

**1AL**

AGG**TTTAGGG**TTTAGGGTTTAGGGTTAGGGTTTAGGGTTTTCGGGTTTAGGGTTTAGGGTTAGGGTTTAGGGTTTAGGGTTTAGGGTTTAGGGTTTAGGGTTTAGGGTTTAGGGTTAGGGTTTAGGGTTAGGTTAGGGTTTAGGGTTTAGGGTTTAGGGTTTAGGGTTTAGGGTTTAGGGGTTAGGGTTTAGGGTTTAGGGTTTAGGGTTTGGGTTAGGGTTTAGGGTTTAGGGTTTAGGGTTTAGGGTTTAGGGTTTAGGGTTTAGGGTTTAGGGTTTAGGGTTTAGGGTTTAGGGTTTAGGGTTTAGGGTTTAGGGTTTAGGGTTTAGGGTTTAGGGTTTAGGGTTTAGGGTTAGGGTTAGGGTTTAGGGTTAGGGTTTAGGGTTAGGGTTAGGGTTTAGGGTTAGGGTTTAGGGTTTAGGGTTTAGGGTTTAGGGTTTAGGGTTTAGGGTTAGGGTTTAGGGTTTAGGGTTTAGGGTTAGGGTTTAGGGTTTAGGGTTTAGGGTTTAGGGTTAAGGGTTTAGGGTTTAGGGTTAGGGTTTAGGGTTTAGGGTTTAGGGTTTAGGGTTTAGGGTTAGGGTTAGGGTTTAGGGTTTAGGGTTTAGGGTTTAGGGTTAGGGTTTAGGGTTAGGGTTTAGGGTTTAGGGTTTAGAGTTAGGGTTTAGGGTTTAGGGTTAGGGTTTAGGGTTTAGGGTTAGGGTTTAGGGTTTAGGGTTTAGGGTTAGGGTTTAGGGTTTAGGGTTTAGGGTTTAGGGTTTAGGGTTTAGGGTTTAGGGTTTAGGGTTTAGGGTTTAGGGTTTAGGGTTTAGGGTTTAGGGTTTAGGGTTTAGGGTTTAGGGTTTAGGGTTAGGGTTTAGGGTTAGGGTTTAGGGTTTAGGGTTTAGGGTTAGGGTTTAGGGTTTAGGGTTAGGGTTAGGGTTAGGGTTTAGGGTTTAGGGTTTAGGGTTAGGGTTTAGGGTTTAGGGTTTAGGGTTTAGGGTTTAGGGTTAGGGTTTAGGGTTTAGGGTTTAGGGTTTAGAGTTTAGGGTTTAGGGTTTAGGGTTTAGGGTTTAGGGTTTAGGGTTTAGGGTTTAGGGTTTAGGGTTTAGGGTTTAGGGTTTAGGGTTTAGGGTTTAGGGTTTAGGGTTTAGGGTTTAGGGTTTAGGGTTTAGGGTTTAGGGTTTAGGGTTTAGGGTTTAGGGTTTAGGGTTTAGGGTTTAGGGTTTAGGGTTTAGGGTTTAGGGTTTAGGGTTTAGGGTTTAGGGTTTAGGGTTTAGGGTTTAGGGTTTAGGGTTTAGGGTTTAGGGTTTAGGGTTTAGGGTTTAGGGTTTAGGGTTTAGGGTTTAGGGTTTAGGGTTTAGGGTTTAGGGTTTAGGGTTTAGGGTTTAGGGTTTAGGGTTTAGGGTTTAGGTTTAGGGTTTAGGGTTTAGGGTTTAGGGTTTAGGGTTTAGGGTTTAGGGTTTAGGGTTTAGGGTTTAGGGTTTAGGGTTTAGGGTTTAGGGTTTAGGGTTTAGGGTTTAGGGTTTAGGGTTTAGGGTTTAGGGTTTAGGGTTTAGGGTTTAGGGTTAGGGTTTAGGGTTTAGGGTTGGTTAGGGTTTAGGGTTTAGGGTTTAGGGTTTAGGGTTTGGTTAGGGTTAGGGTTTAGGGTTTAGGGTTTAGGGTTTAGGGTTTAGGGTTTAGGGTTAGGGTTTAGGGTTTAGGGTTTAGGGTTTAGGGTTTAGGGTTTAGGGGTTTAGGGTTTAGGGTTTAGGGTTTAGGGTTTAGGGTTTAGGGTTTAGGGTTTAGGGTTTAGGGTTTAGGGTTTAGGGTTAGGGTTTAGGGTTTAGGGTTTAGGGTTTAGGGTTTAGGGGTTTAGGGTTTAGGGTTTAGGGTTTAGGGTTTAGGGTTTGGTTAGGGTTTAGGGTTTAGGGTTTAGGGTTAGGGTTGGGTTAGGGTTTAGGGTTTAGGGTTTTGGGTTTAGGGTTTAGGGTTTAGGGTTTAGGGTTAGGGTTTAGGGTTTAGGGTTTAGGGTTTAGGGTTTAGGGGTTTAGGGTTTAGGGTTTAGGGTTTAGGGTTTAGGGTTTAGGTTTAGGGTTTGGGTTTGGGTTTAGGGTTAGGGTTTAGGGTTGTAGGGTTTAGGGTTTAGGGTTTAGGGTAGGGTTTAGGGTTTAGGGTTTAGGGTTTAGGGTTTTGGTTTAGGGTTTAGGGTTTAGGGGTTTAGGGGTTTAGGGTTTAGGGTTTAGGGTTTAGGGTTTAGGGTTTAGGGTTTAGGGTTTAGGGTTTAGGGTTTAGGGTTTAGGGTTTAGGGTTTAGGGTTTAGGGTTAGGGTTTAGGGTTTAGGGTTAGGGTTTAGGGTTTAGGGTTTAGGGGTTTAGGGTTTAGGGTTGTAGGGTTTAGGGTTTAGGGTTTAGGGTTTAGGGTTTAGGGTTTAGGGTTAGGGTTTAGGGTTTAGGGTTTAGGGTTTAGGGTTTAGGTTTAGGGTTTAGGGTTTAGGGTTAGGGTTTAGGGTTTAGGGTTTAGGGTTTAGGGTTTAGGGTTTAGGTTTAGGGGTTAGGGTTTAGGGTTAGGGTTTAGGGTTTAGGGTTAGGGTTTAGGGTTTAGGGTTTAGGGTTTAGGGTTTAGGGTTTAGGGTTTAGGGTTAGGGTTTAGGGTTTAGGGTTTAGGGTTAGGGTTAGGGTTTAGGGTTAGGGTTTAGGGTTTAGGGTTTGGGTTTAGGGTTTAGGGTTTAGGGTTAGGGTTTAGGGTTTAGGGTTTAGGGTTTAGGGTTTAGGGTTAGGGTTTAGGGGTTTAGGGTTTAGGGTTTAGGGTTTAGGGTTTAGGGTTTAGGGTTTAGGGGTTAGGGTTTAGGGTTTAGGGTTTAGGGTTTAGGGTTAGGGTTTAGGGTTTAGGGTTTAGGGTTTAGGGTTTAGGGTTAGGGTTTAGGGTTTAGGGTTTAGGGTTTAGGGTTTAGGGTTTAGGGTTTAGGGTTTAGGGTTAGGGTTTAGGGTTTAGGGTTTAGGGTTTGGGTTTAGGGTTTAGGGTTAGGGTTGTAGGGTTTAGGGTTTAGGGTTAGGGTTTAGGGTTTAGGGTTTAGGGTTTAGGGTTAGGGTTTAGGGTTTAGGGTTTAGGGTTAGGGTTTAGGGTTTAGGGTTTGGTAGGGTTTAGGGTTTAGGGTTTAGGGTTTAGGGTTTAGGGTTTAGGGTTAGGGTTTAGGGTTTAGGGTTTAGGGTTTAGGTGGTTTAGGGTTTAGGGTTTAGGGTTTAGGGTTTAGGGTTGGTTAGGGTTTAGGGTTTAGGGTTTAGGGTTTAGGGTTTAGGGTTTAGGGTTTAGGGTTTAGGGTTTAGGGTTTAGGGTTTAGGGTTTAGGGTTTTAGGGTTTAGGGTTTAGGGTTTAGGGTTGTTTAGGGTTTAGGGTTTAGGGTTTAGGGTTTTTGGGTTTAGGGTTTAGGGTTTAGGGTTTAGGGTTTAGGGTTTAGGGTTTAGGGTTTAGGGTTTAGGGTTTAGGGTTTAGGGTTTAGGGTTTAGGGTTTAGGGTTTAGGGTTTAGGGTTTAGGGTTTAGGGTTTAGGGTTTAGGGTTTAGGGTTTAGGGTTTAGGGTTTAGGGTTTAGGGTTTAGGGTTTAGGGTTTAGGGTTTAGGGTTTAGGGTTTAGGGTTTAGGGTTTAGGGTTTAGGGTTAGGTTTAGGGTTTAGGGTTTAGGGGTTTAGGGTTTAGGGTTTAGGGT

**2AS**

GG**TTTAGGG**TTTAGGGTTTAGGGTTTAGGGTTTAGGGTTTAGGGTTTAGGGTTTAGGGTTTAGGGTTTTAGGGTTTAGGGTTTAGGGTTAGGGTTTAGGGTTTAGGGTTTAGGGTTTAGGGTTTAGGGTTAGGGTTTAGGGTTTAGGGTTTAGGGTTTAGGGTTTAGGGTTTAGGGTTTAGGGTTTAGGGTTAGGGTTTAGGGTTTAGGGTTTAGGGTTTAGGGTTTAGGGTTTAGGGTTTAGGGTTTAGGGTTTAGGGTTAGGGTTTAGGGTTTAGGGTTTAGGGTTTAGGGTTTAGGGTTTAGGGTTTAGGGTTTAGGGTTTAGGGTTTAGGGTTTAGGGTTTAGGGTTTAGGGTTTAGGGTTTAGGGTTTAGGGTTTAGGGTTAGGGTTTAGGGTTTAGGGTTAGGGTTAGGGTTTAGGGTTTAGGGTTTAGGGTTTAGGGTTTAGGGTTTAGGGTTAGGGTTAGGGTTTAGGGTTTAGGGTTTAGGGTTAGGGTTTAGGGTTTAGGGTTTAGGGTTTAGGGTTTAGGGTTAGGGTTAGGGTTTAGGGTTTAGGGTTAGGGTTTAGGGTTTAGGGTTTAGGGTTTAGGGTTTAGGGTTTAGGGTTTAGGGTTTAGGGTTTAGGGTTTAGGGTTTAGGGTTTAGGGTTTAGGGTTTAGGGTTTAGGGTTTAGGGTTTAGGGTTTAGGGTTTAGGGTTTAGGGTTTAGGGTTTAGGGTTTAGGGTTTAGGGTTTAGGGTTTAGGGTTTAGGGTTTAGGGTTTAGGGTTTAGGGTTAGGGTTTAGGGTTTAGGGTTTAGGGTTTAGGGTTTAGGGTTTAGGGTTTAGGGTTTAGGGTTTAGGGTTTAGGGTTAGGGTTTAGGGTTTAGGGTTTAGGGTTTAGGGTTTAGGGTTTAGGGTTTAGGGTTTAGGGTTTAGGGTTTAGGGTTTAGGGTTTAGGGTTTAGGGTTTAGGGTTTAGGGTTTAGGGTTTAGGGTTTAGGGTTTAGGGTTTAGGGTTTAGGGTTTAGGGTTTAGGGTTTAGGGTTTAGGGTTTAGGGTTTAGGGTTTAGGGTTTAGGGTTTAGGGTTTAGGGTTTAGGGTTTAGGGTTTAGGGTTTAGGGTTTAGGGTTTAGGGTTTAGGTTAGGGTTTAGGGTTTAGGGTTTAGGGTTGGGTTTAGGGTTTAGGGTTTAGGGTTAGGGTTTAGGGTTTAGGGTTTAGGGTTTAGGGTTTAGGGTTTAGGGTTTAGGGTTTAGGGTTTAGGGTTTAGGGTTTAGGGTTTAGGGTTTAGGGTTTAGGGTTTAGGGTTTAGGGTTTAGGGTTTAGGGTTTAGGGTTTAGGGTTAGGGTTTAGGGTTTAGGGTTTAGGGTTTAGGGTTTAGGGTTTAGGGTTTAGGGTTTAGGGTTTAGGGTTTAGGGTTTAGGGTTTAGGGTTTAGGGTTTAGGGTTTAGGGTTTAGGGTTTAGGGTTTAGGGTTTAGGGTTTAGGGTTTAGGGTTTAGGGTTTAGGGTTTTAGGGTTTAGGGTTTAGGGTTTAGGGTTTAGGGTTTAGGGTTTAGGGTTTAGGGTTTAGGGTTTAGGGTTTAGGGTTTAGGGTTTAGGGTTTAGGGTTTAGGGTTTAGGGTTTAGGGTTTAGGGTTTAGGGTTTAGGGTTTAGGGTTTAGGGTTTAGGGTTTAGGGTTTAGGGTTTAGGGTTTAGGGTTTAGGGTTTAGGGTTAGGGTTTAGGGTTTAGGGTTTAGGGTTTAGGGTTTAGGGGTTTAGGGTTTAGGGTTTAGGGTTTAGGGTTTAGGGTTTAGGGTTTAGGGTTTAGGGTTTAGGGTTTAGGGTTTAGGGTTTAGGGTTTAGGGTTTAGGGTTTAGGGTTTAGGGTTTAGGGTTTAGGGTTTAGGGTTTAGGGTTTAGGGTTTAGGGTTTAGGGTTTAGGGTTTAGGGTTTAGGGTTTAGGGTTTAGGTTTAGGGTTTAGGGTTTAGGGTTTAGGGTTTAGGGTTTAGGGTTAGGGTTTAGGGTTTAGGGTTAGGGTTTAGGGTTTAGGGTTTAGGGTTAGGGTTTAGGGTTTAGGGTTTAGGGTTAGGGTTAGGGTTTAGGGTTTAGGGTTTAGGGTTTAGGGTTAGGGTTAGGGTTTAGGGTTTAGGGTTTAGGGTTTAGGGTTAGGGTTTAGGGTTTAGGGTTTAGGGTTTAGGGTTTAGGGTTTAGGGTTTAGGGTTTAGGGTTTAGGGTTTAGGGTTTAGGGTTTAGGGTTAGGGTTTAGGGTTTAGGGTTTAGGGTTTAGGGTTTAGGGTTTAGGGTTTAGGGTTTAGGGTTTAGGGTTTAGGGTTTAGGGTTTAGGGTTTAGGGTTTAGGGTTTAGGGTTTAGGGTTTAGGGTTAGGGTTTAGGGTTTAGGGTTTAGGGTTTAGGGTTTAGGGTTAGGGTTTAGGGTTTAGGGTTTAGGGTTTAGGGTTTAGGGTTTAGGGTTTAGGGTTTAGGGTTTAGGGTTTAGGGTTAGGGTTTAGGGTTTAGGGTTTAGGGTTTAGGGTTTAGGTTTAGGGTTTAGGGTTTAGGGTTTAGGGTTTAGGGTTTAGGGTTTAGGGTTTAGGGTTTAGGGTTAGGGTTTAGGGTTTAGGGTTAGGGTTAGGGTTTAGGGTTTAGGGTTTAGGGTTTAGGGTTTAGGGTTAGGGTTTAGGGTTAGGGTTTAGGGTTTAGGGTTTAGGGTTAGGGTTTAGGGTTTAGGGTTTAGGGTTTAGGGTTAGGGTTTAGGGTTTAGGGTTAGGGTTTAGGGTTTAGGGTTAGGGTTTAGGGGTTTAGGGTTTAGGGTTTAGGGTTTAGGGTTTAGGGTTTGGGTTAGGGTTTAGGGTTAGGGTTTAGGGTTAGGGTTTAGGGTTTAGGGTTTAGGGTTAGGGTTTAGGGTTTAGGGTTTAGGGTTTAGGGTTTAGGGTTTAGGGTTTAGGTTAGGGTTGGTTTAGGGTTAGGGTTTAGGGTTTAGGGTTAGGGTTTAGGGTTTAGGGTTTAGGGTTTAGGGTTTAGGGTTTAGGGTTAGGGTTAGGGTTTAGGGTTTAGGGGTTAGGGTTTAGGGTTTAGGGTTTAGGGTTTAGGGTTTAGGGTTTAGGGTTTAGGGTTTAGGGTTTAGGGTTAGGGTTTAGGGGTTTTAGGGTTTGTTAGGGTTAGGGTTTAGGGTTAGGGTTTAGGGTTAGGGTTTAGGGTTTAGGGTTAGGGGTTAGGGTTTAGGGTTTAGGGTTTAGGGTTTAGGGTTTAGGGTTTAGGGTTTAGGGTTTAGGGTTTAGGGTTAGGGTTTTGGTTAGGGTTTAGGGTTTAGGGTTAGGGTTTAGGGTTTAGGGTTTAGGGTTAGGGTTTAGGGTTAGGGTTTAGGGTTTTAGGGTGTAGGGTTTAGGGTTTGGGTTTAGGGTTAGGGTTTAGGGGTTTGGGTTAGGGTTTAGGGTTTAGGGTTTAGGGTTTTAGGGTTTAGGGTTTAGGGTTTAGGTTTAGGGTTTAGGGTTTAGGGTTTAGGGTTTAGGGTTAGGGTTTAGGGTTTAGGGTTTAGGGTTTAGGGTTAGGGTTTAGGGTTTAGGGTTTAGGGTTTAGGGTTTGGGTTAGGGTTTAGGGTTAGGGTTTAGGGTTTAGGGTTTAGGGTTTAGGGTTTAGGGTTTAGGGTTAGGGTTTAGGGTTTAGGGTTGGGTTAGGGTTTAGGGTTTAGGGTTAGGGTTTAGGTTTAGGGTTTAGGGTTTAGGGTTAGGGTTTAGGGTTTAGGGTTTAGGTTTTAGGGTTTAGGGTTTAGGGTTTAGGGTTTAGGGTTTAGGGTTTAGGGTTAGGGTTTAGGGTTTAGGGTTTAGGTTTAGGGTTTAGGGTTTAGGGTTTAGGGTTTAGGGTTTAGGGGTTAGGGTTTAGGGTTTAGGGTTAGGGTTTAGGGTTTAGGGTTTAGGGTTTAGGGTTTAGGGTTAGGGTTAGGGTTAGGGTTTAGGGTTTAGGGTTTAGGGTTTAGGGTTTAGGGTTAGGGTTTAGGGTTTAGGGTTTAGGTTTAGGGTTAGGGTTTAGGGTTTAGGGTTTAGGGTTTAGGGTTTAGGGTTTAGGGTTTAGGGTTTTAGGTTTAGGGTTTAGGGTTTAGGGTTTAGGGTTTAGGGTTTAGGGTTTAGGTTTAGGGTTTAGGGTTTAGGGTTTAGGGTTTAGGGTTAGGGTTTGGGTTTAGGGTTAGGGTTTAGGGTTTAGGGTTGGTTAGGTTAGGGTTAGGGTTTAGGGTTTAGGGTTAGGGTTTAGGGTTTAGGGTTTAGGGTTTAGGGTTTAGGGTTTAGGGTTTAGGGTTTAGGGTTTAGGGTTTAGGGTTTAGGGTTTAGGGTTTAGGGTTTAGGGTTTAGGGTTTAGGGTTTAGGGTTTAGGGTTTAGGGTTTAGGGTTTAGGGTTTAGGGTTTAGGGTTTAGGGTTTAGGGTTTAGGGTTAGGGTTTAGGGTTTAGGGTTTAGGGTTTAGGGTTTAGGGTTTAGGGTTTAGGGTTTAGGGTTTAGGGTTTAGGGTTTAGGGTTTAGGGTTTAGGGTTAGGGTTTAGGGTTTAGGGTTTAGGGGTTTAGGGTTAGGGTTTAGGGTTAGGGTTAGGGTTTAGGGTTTAGGGTTAGGGTTTAGGGTTAGGGTTTAGGTTAGGTTAGGGTTTAGGGTTTAGGGTTTAGGGTTTAGGGTTTAGGGTTTAGGGTTAGGGTTTAGGGTTTAGGGTTAGGGTTTAGGGTTTAGGGGTTTAGGGTTTAGGGTTTAGGGTTTGGGTTTAGGGTTTAGGGTTTAGGGTTTAGGGTAGGGTTTAGGGTTTAGGGTTTAGGGTTTAGGGTTTAGGGTTTAGGGTTAGGGTTTAGGGTTTAGGGGTTTAGGGTTTAGGGTTTAGGGTTTAGGGTTTAGGGTTTAGGGTTTAGGGTTTAGGGTTTAGGGTTTAGGGTTTAGGGTTTAGGGTTAGGGTTAGGGTTTAGGGTTTAGGGTTTAAGGGTTTAGGGTTTAGGGTTTAGGGTGTTAGGGTTTAGGGTTAGGGTTTAGGGTTTAGGGTTTAGGGTTTAGGGTTTAGGGTTTAGGGTTTAGGGTTAGGGTTTAGGGTTTAGGGTTTAGGGTTTAGGGTTAGGGTTTAGGGTTTAGGGTTTAGGGTTTAGGGTTTAGGGTTTAGGGTTTAGGGTTTTAGGGTTTGTTAGGGTGGTTTAGGGTTTAGGGTTAGGGTTTAGTGGGTTTAGGGTTTAGGGTGTAGGGTTTAGGGGTTTAGGGTTTAGGGTTTAGGGTTTAGGGTTTAGGGTTTAGGGTTTAGGGTTTAGGGTTTGGGTTAGGGTTTTAGGTTTAGGGTTTAGGGTTTAGGGTTTAGGGTTAGGGTTTAGGGTTTAGGGTTTAGGGTTTAGGGTTTAGGGTTTAGGGTTTAGGGTTTAGGGTTTAGGGTTTAGGGTTTAGGGTTAGGGTTAGGGTTTAGGGTTTAGGGTTTAGGGTTTAGGGTTTTAGGGTTTAGGTTTAGGGTTTAGGGTTTAGGGTTTAGGGTTTAGGGTTTAGGGTTTAGGGTTTAGGGTTTAGGGTTAGGGTTTAGGGTTTAGGGTTTAGGGTTTAGGGTTAGGGTTTAGGGTTTAGGGTTTAGGGTTTAGGGTTTAGGGTTTAGGGTTAGGGTTAGGGTTTAGGGTTTAGGGTTAGGGTTTAGGGTTTAGGGTTTAGGGTTTAGGGTTTAGGGTTTAGGGTTTAGGGTTAGGGTTTAGGGTTTAGGGTTTAGGGTTTAGGGTTTAGGGTTTAGGGTTTAGGGTTTAGGGTTTAGGGTTTAGGGTTTAGGGTTAGGGTTAGGGTTTAGGGTTTAGGGTTTAGGTTTAGGGTTTAGGGTTGTAGGGTTTAGGGTTTAGGGGTTTAGGGTTTAGGGTTTAGGGTTTAGGGTTTAGGGTTTAGGGTTTAGGGTTTAGGGTTTAGGGTTAGGGTTTAGGGTTTAGGGTTTAGGGTTTAGGGGTTAGGGTTTAGGGTTTAGGGTTTAGGGTTAGGGTTTAGGGTTTAGGGTTTAGGGTTAGGGTTTAGGGTTTAGGGTTTAGGGTTTAGGTTTAGGGTTTAGGGTTAGGGTTTAGGGTTTAGGGTTTAGGGTTTTAGGGTTTAGGGTTTAGGGTTTAGGGTTTAGGGTTTAGGGTTTAGGGTTTAGGGTTTAGGGTTTAGGGTTAGGGTTTAGGGTTTAGGGTTTAGGGTTTAGGGTTTAGGGTTTAGGGTTTAGGGTTTAGGTTTAGGGTTTAGGGTTTAGGGTTTAGGGTTTAGGGTTTAGGGTTTAGGGTTTAGGGTTTAGGGTTTAGGGTTTAGGGTTTAGGGTTTAGGGTTTAGGGTTTAGGGTTTTAGGGTTTAGGGTTTAGGGTTTAGGGTTTAGGGTTTAGGGTTTAGGGTTTAGGGTTTAGGGTTTAGGGTTTAGGGTTTAGGGTTTAGGGTTTAGGGTTAGGTTTAGGGTTTAGGGGTTTAGGGTTTAGGGTTTAGGGTTTAGGGTTTAGGGTTTAGGGTTTAGGGTTTAGGGTTTAGGGTTTAGGGTTTAGGGTTAGGGTTTAGGGTTTAGGGTTTAGGGTTTAGGGTTTAGGGTTAGGGTTTAGGGTTTAGGGTTTAGGGTTTAGGGTTTAGGGTTTAGGGTTTAGGGTTTAGGGTTTAGGGTTTAGGGTTAGGGTTTAGGGTTTAGGGTTTTAGGGTTTAGGGTTTTAGGGTTTAGGGTTTAGGGTTTAGGGTTTAGGGTTTAGGGTTTAGGGTTTAGGGTTAGGTTTAGGGTTAGGGTTTAGGGTTTAGGGTTTAGGGTTTAGGGTTTAGGTTTAGGTTTAGGGTTTAGGGTTTAGGTTTAGGGTTTAGGGTTTAGGGTTTTAGGGTTTAGGGTTAGGGTTTAGGGTTTAGGGTTTAGGGTTTAGGGTTTAGGGTTTAGGGTTTAGGGTTAGGGTTTAGGGTTTTAGGGTTTAGGGTTTAGGGTTTAGGGTTTAGGGTTTAGGGTTTAGGGTTTAGGGTTTAGGGTTTAGGGTTTAGGGTTTAGGGTTTAGGGTTTAGGGTTTAGGGTTTAGGGTTTAGGGTTTAGGGTTTAGGGTTTAGGTTTAGGGTTTAGGGTTTAGGGTTTAGGGTTTAGGGTTTAGGGTTTAGGGTTTAGGGTTTAGGGTTTAGGGTTTTAGGGTTTAGGGTTTAGGGTTTAGGGTTTAGGGTTTAGGGTTTAGGGTTTAGGGTTTAGGGGTTTAGGGTTTGGTTAGGGTTTAGGGTTTAGGGTTTAGGGTTTAGGGTTTAGGGTTAGGGTTTAGGGTTTAGGGTTTAGGTTTAGGGTTTAGGGTTTAGGGTTTAGGGTTTAGGGTTTAGGGTTTAGGTGGTTTAGGGTTTAGGGTTTAGGTTAGGGTTTAGGGTTTAGGGTTTAGGGTTTAGGGTTTAGGGTTTAGGGTTTAGGGTTTAGGGTTTAGGGTTTAGGGTTTAGGGTTTAGGTTTAGGGTTTAGGGTTTAGGGTTTAGGGTTTAGGGTTTAGGGTTTAGGGTTAGGGTTAGGGTTTAGGGTTTAGGGTTAGGGTTTAGGGTTTAGGGTTTAGGGTTTAGGGTTTAGGGTTTAGGGTTTAGGTTTTAGGGTTTAGGGTTTAGGGTTTAGGGTTTTAGGGGTTTAGGGTTTAGGGTTTAGGTTTAGGGTTGGTTTAGGGTTTAGGGTTAGGGTTAGGGTTTAGGGTTTAGGGTTTAGGGTTTAGGGTTAGGGTTTAGGGTTTAGGGTTTAGGGTTTAGGGTTTAGGGTTTAGGGTTAGGGTTTAGGGTTAGGGTTTAGGGTTTAGGGTTTAGGGTTTAGGGTTAGGGTTTAGGGTTTAGGGTTTAGGGTTTAGGGTTTAGGGTTAGGGTTTAGGGTTTAGGGTTTTAGGGTTAGGGTTTTAGGGTTTAGGGTTTAGGGTTTAGGGTTTTGGGTTTAGGGTTTAGGGTTTAGGGTTTAGGGTGGTTTAGGGTTTAGGGTTTAAGGGTTTAGGGTTTAGGGTTTAGGGTTTAGGGTTTAGGGTTTAGGGTTTAGGGTTTAGGGTTTAGGGTTAGGTTTAGGGTTTAGGGTTTAGGGTTTAGGGTTTAGTGTTAGGGTTTAGGGTTTAGGGTTTAGGGTTTAGGGTTTAGGGTTTAGGGTTTAGGGTTTAGGGTTTAGGGTTTAGGGTTTAGGGTTTAGGGTTTAGGTTTAGGGTTTTGGGTTTAGGGTTTAGGGGTTTAGGTTTAGGGTTTAGGGTTTAGGGTTTAGGGTTTAGGGTTTAGGGTTAGGGTTTAGGGTTTAGGGTTTAGGGGTTTAGGGTTTAGGGTTTAGGGTTTAGGGTTTAGGTTTAGGGTTTAGGGTTTAGGGTTTAGGGTTTAGGGTTTAGGGTTGGTTAGGGTTTAGGGTTTAGGGTTTTAGGGTTTAGGGTTTAGGGTTTAGGGTTTAGGGTTTAGGGTTTAGGGTTTAGGTTTAGGGTTTAGGGTTTAGGGTTTAGGTTTAGGGTTTAGGGTTTAGGGTTAGGGTTTAGGGTTTAGGGTTTAGGGTTTAGGGTTTAGGGTTTAGGGTTTAGGGTTTAGGGTTTAGGTTTAGGGTTTTAGGGTTTAGGGTTTAGGTTTAGGGTTTAGGGTTTAGGGTTTAGGGTTTAGGGTTTAGGGTTTAGGGTTTAGGGTTTAGGGTTTTAGGGTTAGGGTTTAGGGTTTAGGGTTTAGGGTTTAGGGTTTAGGGTTTAGGGTTTAGGTTTAGGGTTTAGGGTTTAGGGTTTAGGTTTAGGGTTTAGGGTTTAGGGTTTAGGGTTTAGGGTTTAGGTTTAGGGTTTTAGGGTTTAGGGTTTAGGGTTTAGGGTTTAGGGTTTAGGGTTTAGGGTTTAGGGTTTAGGGTTTAGGGTTTAGGGGTGTTAGGGTTTAGGGTTTAGGGTTTAGGGTTTAGGGTTTAGGGTTTAGGGTTTAGGGTTTAGGGTTTAGGGTTAGGGTTTAGGGTTTAGGGTTTAGGGGTTTAGGGTTTAGGGTTTAGGGTTTAGGGTTAGGTTAGGGTTAGGTTAGGGTTTAGGGTTTAGGGTTTAGGGTTTAGGGTTAGGGTTTAGGGTTTAGGGTTTAGGTTTAGGGTTGGTTAGGGGTTTAGGGTTTAGGTTTAGGGTTTAGGGGTTTAGGGTTTAGGGTTTAGGGTTTAGGGTTTAGGGTTAGGGTTTTAGGGTTTAGGGTTTAGGGTTTAGGGTTTAGGGTTTAGGGTTTAGGGTTTAGGGTTTAGGTTTAGGGTTTAGGGTTTAGGGTTTAGGGTTTAGGGTTAGGTTTAGGGTTTAGGGTTTAGGGTTTAGGGTTTAGGGTTTAGGGTTTAGGGTTTAGGGTTTAGGGTTTAGGGTTTAGGGTTTAGGGTTTAGGGTTTAGGGTTTAGGGTTTAGGGTTTAGGGTTTAGGGTTTTAGGGTTTAGGGTTTAGGGTTTAGG

**3AS**

ATTTGAGGG**TTTAGGG**TTTAGGGCTTAGGGTTTAGGGTTTAGGGTTAGGGTTAGGGTTAGTGTTAGGGTTTAGGTTTAGGGTTTAGGGTTTAGGGTTTAGGGTTTAGGGTTTAGGGTTTAGGGTTTAGGGTTTAGGGTTTAGGGTTTAGGGTTTAGGGTTTAGGGTTTAGGGTTTAGGGTTTAGGGTTTAGGGTTTAGGGTTTAGGGTTTAGGGTTTAGGGTTAGGGTTTAGGGTTTAGGGTTTAGGGTTTAGGGTTTAGGGTTTAGGGTTTAGGGTTTAGGGTTTAGGGTTTTAGGGTTTTAGGGTTTAGGGTTTAGGGTTTAGGGTTAGGGTTTAGGGTTTAGGGTTTAGGGTTTAGGGTTTAGGGTTTAGGGTTTAGGGTTTAGGGTTTAGGGTTTAGGGTTTTAGGGTTTTAGGGTTTAGGGTTTTAGGGTTTAGGGTTTAGGGTTTAGGGTTTAGGGTTTAGGGTTTAGGGTTAGGGTTTAGGGTTTAGGGTTTAGGGTTTAGGGTTAGGGTTTAGGGTTTAGGGTTTAGGGTTTAGGGTTTAGGGTTTAGGGTTTAGGGTTTAGGGTTTAGGGTTTAGGGTTTAGGGTTTAGGGTTTAGGGTTTAGGGTTTAGGGTTTAGGGTTTTAGGGTTTAGGGTTTAGGGTTTAGGGTTTAGGGTTTAGGGTTTAGGGTTTAGGGTTTAGGGTTTAGGGTTTAGGGTTTAGGGTTTAGGGTTTAGGGTTTAGGGTTTAGGGTTTAGGGTTTAGGGTTTAGGGTTTAGGGTTAGGGTTTAGGGTTTAGGGTTTAGGGTTTAGGGTTTAGGGTTTAGGGTTTAGGGTTTAGGGTTTAGGGTTTAGGGTTTAGGGTTTAGGGTTAGGGTTTAGGGTTTAGGGTTTAGGGTTTAGGGTTTAGGGTTAAGGGTTTAGGGTTTAGGGTTTAGGGTTAGGGTTTAGGGTTTAGGGTTTAGGGTTTAGGGTTAGGGTTTAGGGTTTAGGGTTTAGGGTTTAGGGTTTAGGGTTTAGGGTTTAGGGTTTAGGGTTTAGGGTTTAGGGTTTAGGGTTTAGGGTTTAGGGTTTAGGGTTTAGGGTTTAGGGTTTAGGGTTTAGGGTTTAGGGTTTAGGGTTTAGGGTTTAGGGTTTAGGGTTTAGGGTTTAGGGTTTAGGGTTTAGGGTTTAGGGTTTAGGGTTTAGGGTTTAGGGTTTAGGGTTTAGGGTTTAGGGTTTAGGGTTTAGGGTTTAGGGTTTAGGGTTTAGGGTTTAGGGTTTAGGGTTTAGGGTTTAGGGTTTAGGGTTTAGGGTTTAGGGTTTAGGGTTTAGGGTTTAGGGTTTAGGGTTTAGGGTTTAGGGTTTAGGGTTTAGGGTTTAGGGTTTAGGGTTTAGGGTTTAGGGTTTAGGGTTTAGGGTTTAGGGTTTAGGGTTTAGGGTTTAGGGTTAGGGTTAGGTTTAGGGTTTAGGGTTTAGGGTTTAGGGTTTAGGGTTTAGGGTTTAGGGTTTAGGTTTAGGGTTTAGGGTTAGGGTTTAGGGTTTAGGGTTTAGGGTTTAGGGTTTAGGGTTTAGGGTTTAGGGTTTAGGGTTTAGGGTTTAGGGTTAGGGTTTAGGGTTTAGGGTTTAGGGTTTAGGGTTTAGGGTTTAGGGTTTAGGGTTTAGGGTTAGGGTTTAGGGTTTAGGGTTTAGGGTTGTTAGGGTTAGGGTTAGGGTTTAGGGTTTAGGGTTTAGGGTTTAGGGTTTAGGGTTTAGGGTTTAGGGTTAGGGTTTAGGGTTTAGGGTTTAGGGTTTAGGTTAGGGTTTAGGGTTTAGGGTTTAGGGTTAGGGTTTAGGGTTTAGGGTTTAGGGTTAGGGTTAGGGTTTAGGGTTAGGGTTTAGGGTTTAGGGTTTAGGGTTTAGGGTTTAGGGTTAGGGTTTAGGGTTTAGGGTTTAGGGTTTAGGGTTTAGGGTTTAGGGTTAGGGTTTAGGGTTTAGGGTTAGGGTTTAGGGTTAGGGTTAGGGTTTAGGGTTTAGGGTTTAGGGTTTAGGGTTAGGGTTAGGGTTTAGGGTTTAGGGTTTAGGGTTTAGGGTTTAGGGTTTAGGGTTTAGGGTTTAGGGTTTAGGGTTTAGGGTTTAGGGTTTAGGGTTTAGGGTTTAGGGTTTAGGGTTAGGGTTTAGGGTTTAGGGTTAGGGTTAGGGTTTAGGGTTTAGGGTTAGGGTTTAGGGTTTAGGGTTTAGGGTTTAGGGTTTAGGGTTAGGGTTAGGGTTTAGGGTTTAGGGTTTAGGGTTTAGGGTTTAGGGTTTAGGGTTAGGGTTTAGGGTTTAGGGTTTAGGGTTTAGGGTTTAGGGTTTAGGGTTTAGGTTTAGGGTTTAGGGTTTAGGGTTTAGGGTTTAGGGTTTAGGGTTTAGGGTTTAGGGTTTAGGGTTTAGGGTTTAGGGTTTAGGGTTAGGGTTTTAGGGTTTAGGGTTTAGGGTTTAGGGTTTAGGGTTTAGGGGTTTAGGGTTTAGGGTTTAGGGTTTAGGGTTTAGGGTTAGGGTTTAGGGTTTAGGGTTTAGGGTTTAAGGGTTAGGGTTTAGGGTTTAGGGTTTAGGGTTAGGGTTTAGGGTTTAGGGTTTAGGGTTTAGGGTTTTAGGGTTTAGGGTTTAGGGTTTAGGGTAGGGTTTAGGGTTTAGGGTTTAGGGTTTAGGGTTTAGGGTTTAGGGTTTAGGGTTAGGGTTTAGGGTTTAGGTTTAGGGTTTAGGGTTAGGGTTTAGGGTTTAGGGTTTAGGGTTTAGGGTTTAGGGTTTAGGGTTTAGGGTTTAGGGTTTGGGTTTAGGGTTTAGGGTTTAGGGTTTAGGGTTTAGGGTTTAGGGTTTAGGGTTTAGGGTTTAGGGTTTAAGGGTTTAGGGTTTAGGGTTTAGGGTTTAGGGTTTAGGGTTAGGGTTTAGGGTTTAGGGGTTAGGGTTTAGGGTTTAGGGTTTAGGGTTTAGGGTTTAGGGTTTAGGGTTTAGGGTTTAGGGTTTAGGGTTTAGGGTTTAGGGTTAGGGTTTAGGGTTTAGGGTTTAGGGTTAGGGTTTAGGGTTTAGGGTTTAGGGTTTAGGGTTAGGGTTTAGGGTTTAGGGTTTAGGGTTAGGGTTTAGGGTTTAGGGTTTAGGGTTTAGGGTTTAGGTTTAGGGTTTAGGGTTTAGGGTTTAGGGTTAGGGTTTAGGGTTTAGGGTTTAGGGTTTAGGGGTTTAGGGTTTAGGGTTTAGGGTTTAGGGTTAGGGTTTAGGGTTTAGGGTTTAGGGTTTAGGGTTAGGGTTTAGGGTTTAGGGTTTAGGGTTTAGGGTTTAGGGTTTAGGGTTTAGGGTTTAGGGTTTAGGGTTAGGGTTTAGGGTTAGGGTTTAGGGTTTAGGGTTTAGGGTTTAGGGTTTAGGGTTTAGGGTTTAGGGTTAGGGTTTAGGGTTTAGGGTTAGGGTTTAGGGTTTAGGTTTAGGGTTTAGGGTTAGGGTTTAGGGTTTAGGGTTTAGGGTTTAGGGTTTAGGGTTAGGGTTTAGGGTTTAGGGTTTAGGGTTTAGGTTTAGGGTTAGGGTTAGGGTTTAGGGTTTAGGGTTTAGGGTTTAGGGTTTAGGGTTTAGGGTTTAGGGTTTAGGGTTTAGGGTTAGGGTTTAGGGTTTAGGGTTAGGGTTTAGGGTTTAGGGTTTAGGGTTTAGGGTTTAGGGTTTAGGTTTAGGGTTTAGGGTTTAGGGTTAGGGTTTAGGGTTTAGGGTTTAGGTTTAGGGTTTAGGGTTAGGGTTTAGGGTTTAGGGTTTAGGGTTTAGGGTTTAGGGTTAGGGTTAGGGTTTAGGGTTTAGGGTTTAGGGTTTAGGGTTTAGGGTTTAGGGTTAGGGTTTAGGGTTAGGGTTTAGGGTTTAGGTTAGGGTTTAGGGTTTAGGGTTTAGGGTTTAGGGTTTAGGGTTAGGGTTTAGGGTTTAGGGTTAGGGTTTAGGGTTAGGGTTTAGGGTTTAGGGTTTAGGGTTTAGGGTTTAGGGTTTAGGGTTTAGGGTTTAGGGTTTAGGGTTAGGGTTTAGGGTTTAGGGTTTAGGGTTAGGGTTTAGGGTTTAGGGTTTAGGGTTAGGGTTTAGGGTTTAGGGTTTAGGGTTTAGGGTTTAGGGTTTAGGGTTTAGGGTTTAGGGTTTAGGGTTTAGGGTTAGGGTTTAGGGTTTAGGGTTTAGGGTTTAGGGTTTAGGGTTTAGGGTTTAGGGTTTAGGTGGTTTAGGGTTTAGGGTTTAGGGTTTAGGGTTTAGGGTTTAGGGTAGGGTTAGGGTTAGGGTTTAGGGTTTAGGGTTTTGGGTTTAGGGTTAGGGTTTAGGGTTTAGGGTTTAGGGTTTAGGGTTTAGGGTTTAGGGTTTAGGGTTTAGGGTTTAGGGTTTAGGGTTAGGGTTTAGGGTTTAGGGTTTAGGGTTTAGGGTTTAGGGTTTAGGGTTTAGGGTTTAGGGTTTAGGGTTTAGGGTTTAGGGTTTAGGGTTTAGGGTTTAGGGTTTAGGGTTTAGGGTTTAGGGTTTAGGGTTTAGGGTTTAGGGTTTAGGGTTTAGGGTTAGGGTTTAGGGTTTAGGGTTTAGGGTTTAGGGTTTAGGGTTTAGGGTTTAGGGTTAGGGTTTAGGGTTTAGGGTTTAGGGTTTAGGGTTTAGGGTTTAGGGTTTAGGGTTTAGGGTTAGGGTTTAGGGTTTTAGGGTTTAGGGTTTAGGGTTAGGGTTTAGGGTTTAGGGTTTAGGGTTTAGGGTTTAGGGTTTAGGGTTTAGGGTTTAGGGTTAGGGTTTAGGGTTTAGGGTTTAGGGTTTAGGGGTTTAGGGTTTAGGTTTAGGGTTTAGGGTTAGGGTTTAGGGTTTAGGGTTAGGGTTTAGGGTTTAGGGTTTAGGGTTTAGGGTTTAGGGTTAGGGTTTAGGGTTTAGGGTTTAGGGTTTAGGGTTAGTAGGGTTTAGGGTTTAGGGTTTAGGGTTTAGGGTTTAGGTTTAGGGTTTAGGGTTAGGGTTTAGGGTTAGGGTTAGGGTTTAGGGTTTAGGGGTTAGGGTTTAGGGTTTAGGGTTTAGGGTTTAGGTTTAGGGTTTAGGGTTTAGGGTTTAGGGTTTAGGGTTTAGGGTTTAGGGTTTAGGGTTTAGGGGTTTAGGGTTTAGGGTTTAGGGTTTAGGGTTTAGGGTTTAGGGTTAGGGTTTAGGGTTTAGGGTTTAGGGTTTAGGGTTAGGGTTTAGGGTTTAGGGTTTAGGGTTTAGGGTTTAGGGTTTAGGGTTTAGGGTTTAGGGTTTAGGGTTTAGGGTTAGGGTTTAGGGTTTAGGGTTTAGGGTTTAGGGTTTAGGGTTAGGGTTTAGGGTTTTAGGGTTTAGGGTTAGGGTTTAGGGTTTAGGGTTTAGGGTTTAGGGTTTAGGGTTTAGGGTTTAGGGTTTAGGGTTTAGGGTTAGGGTTTAGGGTTTAGGGTTTAGGGTTTAGGGTTTAGGGTTTAGGGTTTAGGGTTTAGGTTTAGGGTTTAGGGTTTAGGGTTTAGGGTTTAGGGTTTAGGGTTTAGGGTTTAGGTTTAGGGTTTAGGGTTTAGGGTTTAGGGTTTAGGGGTTTAGGGTTTAGGGTTTAGGGTTTAGGGTTAGGGTTAGGGTTTAGGGTTTAGGGTTTAGGGTTTAGGGGTTTAGGGTTTAGGGTTTAGGGTTTAGGGTTTAGGGTTTAGGGTTTAGGGTTTAGGGTTTAGGGTTTAGGGTTAGGGTTTAGGGTTTAGGGTTTAGGGTTTAGGGTTTAGGGTTTAGGGTTTAGGGTTTTAGGGTTTAGGGTTTAGGGTTTAGGGTTTAGGGTTTAGGGTTTAGGGTTTAGGGTTTAGGGTTTAGGGTTTAGGGTTTAGGGTTTAGGGTTTAGGGTTTAGGGTTAGGGTTTAGGGTTTAGGGTTTAGGGTTTAGGGTTTAGGGTTTAGGGTTTAGGGTTTAGGGTTTAGGGTTTAGGGTTTAGGGTTTAGGGTTTAGGGTTTAGGGTTTAGGGTTTAGGGTTTAGGGTTTAGGGTTTAGGGTTTAGGGTTTAGGGTTTAGGGTTTAGGGTTTAGGGTTTAGGTTTAGGGTTTAGGGTTTAGGGTTTAGGGTTTAGGGTTTAGGGTTTAGGGTTTAGGGTTTAGGGTTAGGGTTTAGGGTTTAGGGTTTAGGGTTTAGGGTTTAGGGGTTTAGGGTTTAGGGTTTAGGGTTAGGGTTTAGGGTTTAGGGTTTAGGGTTAGGGTTTAGGGTTTAGGGTTTAGGGTTTAGGGTTTAGGGTTTAGGGTTTAGGGTTTAGGGTTTAGGGTTTAGGGTTTAGGGTTTAGGGTTTAGGGTTTAGGGTTTAGGGTTTAGGGTTTAGGGTTTAGGGTTTAGGGTTTAGGGTTTAGGGTTTAGGGTTTAGGGTTTAGGGTTTAGGGTTTAGGGTTTAGGGTTTAGGGTTTAGGGTTTAGGGTTTAGGGTTTAGGGTTTAGGGTTTAGGGTTTTAGGGTTTAGGGTTTAGGGTTTAGGGTTTAGGGTTTAGGGTTTAGGGTTAGGGTTTAGGGTAGGGTTTAGGGTTTAGGGTTTAGGTTTAGGGTTTAGGGTTAGGGTTTAGGGTTTAGGGTTAGGGTTAGGGTTTAGGGTTTAGGGTTTAGGGTTTAGGGTTTAGGGTTTAGGGTTTAGGGTTTGGTTTAGGTTTAGGGTTTAGGGTTTAGGGTTTAGGGTTTAGGGTTTAGGGTTTAGGGTTAGGGTTTAGGGTTAGGGTTTAGGGTTTAGGGTTTAGGGTTTAGGGTTTAGGGTTTAGGGTTTAGGGTTTAGGGTTTAGGGTTAGGGTTTAGGGTTTAGGGTTTTAGGGTTAGGGTTTAGGGTTTAGGGTTTAGGGTTTAGGGTTTAGGGTTTAGGGTTAGGGTTTAGGGTTTAGGGTTTAGGGTTTAGGGTTTAGGGTTTTAGGGTTAGGGTTTAGGGTTTAGGGTTTAGGGTTAGGGTTTAGGGTTTAGGGTTAGGGTTTAGGGTTTAGGGTTTAGGGTTTAGGGTTTAGGGTTTAGGGTTTAGGGTTAGGGTTTAGGGTTTAGGGTTTAGGGTTTAGGGTTTTAGGGTTTAGGGTTTAGGGTTTAGGGTTTAAGGGTTTAGGGTTTAGGGTTTAGGGTTTAGGGTTTAGGGTTTAGGGTTTAGGGTTTAGGGTTAGGGTTTAGGGTTTAGGGTTAGGGTTTAGGTTTAGGGTTTAGGGTTTAGGGTTTAGGGTTTAGGGGTTTAGGGTTTAGGGTTTAGGGTTTAGGGTTAGGGTTTAGGGTTTAGGGTTTAGGGTTTAGGGTTTAGGGTTTAGGGTTAGGGTTTAGGTTAGGGTTTAGGGTTTAGGGTTTAGGGTTTAGGGTTTTAGGGTTTAGGGTTTAGGGTTAGGGTTAGGGTTTAGGGTTTAGGGTTTAGGGTTTAGGGTTTAGGGTTTAGGGTTTAGGGTTAGGGTTAGGGTTTAGGGTTTAGGGTTTAGGGTTTAGGGTTTAGGGTTTAGGGTTTAGGGTTTAGGGTTTAGGGTTTAGGGTTTAGGGTTTAGGGTTTAGGGTTTAGGGTTTAGGGTTTGGTTAGGGTTTAGGGTTTAGGGGTTTAGGGTTTAGGGTTTAGGGTTTAGGGTTTAGGGTTTAGGGGTTTAGGGTTTAGGGTTTAGGGTTTAGGGTTTAGGGTTTAGGGTTTAGGGTTTAGGGTTTAGGGTTAGGGTAGTTAGGGTTTAGGGTTTAGGGTTTAGGGTTTAGGGTTTAGGGTTTAGGGTTTAGGGTTTAGGGTTTAGGGTTTAGGGTTTAGGGTTTAGGGTTTAGGGTTTAGGGTTTAGGGTTTAGGGTTTAGGGTTTAGGGTTTAGGGTTTAGGGTTTAGGGTTTAGGGTTTAGGGTTTAGGGTTTAGGGTTTAGGGTTAGGTTTAGGGTTAGGGTTAGGGTTTAGGGTTTAGGGTTTAGGGTTTAGGGTTTAGGGGTTAGGGTTTAGGGTTTAGGGTTTAGGGTTTAGGGTTTAGGTTTAGGTTAGGGTTTAGGGTTTAGGGTTTAGGGTTTAGGGTTTAGGGTTTAGGGTTTAGGGTTAGGGTTTAGGGTTAGGGTTTAGGTAGGTTTAGGGTTTAGGGTTTAGGGTTTAGGGTTTAGGGTTTAGGGTTTAGGGTTTAGGGTTTAGGGTTTAGGGTTTAGGGTTTAGGGTTTAGGGTTTAGGGTTTAGGGTTTAGGGTTTAGGGTTTAGGGTTTAGGGTTTAGGTTTAGGGTTAGGGTTTAGGGTTAGGGTTTAGGGTTTAGGGTTTAGGGTTTAGGGTTTAGGGTTTGGTTAGGGTTTAGGGTTTAGGGTTTAGGGTTTAGGGTTTAGGGTTTAGGGTTTAGGGTTTAGGGTTTAGGGTTTAGGGTTTAGGGTTTAGGGTTTAGGGTTTAGGGTTTAGGGTTTAGGGGTTTAGGGTTAGGGTTTAGGGTTTAGGGTTTAGGGTTTAGGGTTTAGGGTTAGGGTTTAGGGTTTAGGGTTAGGGTTAGGGTTTAGGGTTTAGGTTAGGGTTTAGGGTTTAGGGTTAGGGTTTAGGGTTTAGGGTTTAGGGTTTAGGGTTTAGGGTTTAGGGTTAGGGTTTAGGGTTAGGTTTAGGGTTTAGGGTTTAGGGTTTAGGGTTTAGGGTTTAGGGTTTAGGGTTTAGGGTTTAGGGTTAGGGTTTAGGGTTTAGGGTTTAGGGTTAGGGTTAGGTTAGGGTTTAGGGTTTAGGGTTTAGGGTTTAGGGTTTAGGGTTTAGGGTTAGGGTTTAGGGTTTAGGGTTTAGGGTTTAGGGTTTAGGGTTTAGGGTTTAGGGTTAGGGTTTAGGGTTTAGGGTTTAGGGTTTAGGTAGGTTTAGGGTTTAGGGTTTAGGGTTTAGGGTTTAGGGTTTAGGGTTTAGGGTTTAGGGTTTAGGGTTTAGGGTTTAGGGGTTTAGGGTTTAGGGTTTAGGGGTTTAGGGTTTAGGGTTTAGGGTTTAGGTAGGGTTTAGGGTTAGGGTTTAGGGTTTAGGGTTTAGGGTTAGGGTTTAGGGTTTAGGGTTTAGGGTTTAGGGTTAGGGTTTAGGGTTTAGGGTTTAGGGTTTAGGGTTTAGGGTTTAGGGTTTAGGGTTAGGGTTTAGGGTTTAGGGTTTAGGGTTTAGGGTTTAGGGTTTAGGGTTAGGGTTTAGGGTTTAGGGTTTAGGGTTTAGGGTTTAGGGTTAGGGTTTAGGGTTTAGGGTTTAGGGTTTAGGGTTAGGGTTTAGGGTTTAGGGTTTAGGGTTAGGGTTTAGGGTTAGGGTTTAGGGTTAGGGTTAGGGTTTAGGGTTTAGGGTTTAGGGTTTAGGTTTAGGGTTTAGGGTTAGGGTTTAGGGTTTAGGGTTTAGGGTTTAGGGTTTAGGGTTTAGGGTTTAGGGTTTAGGGTTTAGGGTTTAGGGTTTAGGGTTTAGGGTTTAGGGTTAGGGTTTAGGGTTAGGGTTTAGGGTTTAGGGTTTAGGGTTTAGGGTTTAGGGTTTAGGGTTTAGGGTTAGGGTTTAGGGTTTAGGGTTTAGGGTTTAGGGTTTAGGGTTTAGGGTTTAGGGTTTAGGGTTTAGGGGTTTAGGTTTAGGGTTTAGGGTTTAGGGTTTAGGGTTTAGGGTTTAGGGTTTAGGGTTTAGGGTTGGTTAGGGTTTAGGGTTTAGGGTTTAGGGTTTAGGGTTTAGGGTTTAGGGTTTAGGGTTTAGGGTTTAGGGTTTAGGGTTTAGGGTTTAGGGTTTAAGGGTTTAGGGTTTAGGGTTTAGGGTTTAGGGTTAGGGTTTAGGGTTTAGGGTTTAGGGTTTAGGGTTTAGGGTTTAGGGTTTAGGGTTTAGGGTTTAGGGTTAGGGTTTAGGGTTTAGGGTTTAGGGTTTAGGGTTTAGGGTTTAGGGTTTAGGGTTTAGGGTTTAGGGTTTAGGGTTTAGGGTTTAGGGTTTAGGGTTTAGGGTTTAGGGTTTAGGGTTTAGGGTTTAGGGTTTAGGGTTTAGGGTTTAGGGTTTAGGGTTAGGGTTTAGGGTTTAGGGTTAGGGTTTAGGGTTAGGG

**3AL**

TGGTTAGGG**TTTAGGG**TTTAGGGTTTAGGGTTTAGGGTTACGGTTTAGGGTTTAGGGTTTAGGGTTTAGGGTTTAGGGTTTAGGGTTTAGGGTTAGGGTTTAGGGTTTAGGATTAGGGTTTAGGGTTTAGGGTTTAGGGTTTTTAGGGTTTGGGATTTAGGGTTTAGGGTTTAGGGTTAGGGTTTAGGGTTAGGGTTTAGGGTTTAGGGTTTAGGGTTTAGGGTTTAGGGTTTAGGGTTTAGGGTTTAGGGTTAGGGTTTAGGGTTTAGGGTTTAGGGTTAGGGTTTAGGGTTTAGGGTTTAGGGTTTAGGGTTTAGGGTTTAGGGTTTAGGGTTTAGGGTTTAGGGTTTAGGGTTTAGGGTTTAGGGTTTAGGGTTTAGGGTTTAGGGTTTAGGGTTTAGGGTTTAGGGTTTAGGGTTTAGGGTTTAGGGTTTAGGGTTTAGGGTTTAGGGTTTAGGGTTTAGGGTTTAGGGTTTAGGGTTTAGGGTTTAGGGTTTAGGGTTTAGGGTTTAGGGTTTAGGGTTTAGGGTTTAGGGTTTAGGGTTTAGGGTTTAGGGTTTAGGGTTTAGGGTTTAGGGTTTAGGGTTTAGGGTTTAGGGTTTAGGGTTTAGGGTTTAGGGTTTAGGGTTTAGGGTTTAGGGTTTAGGGTTTAGGGTTTAGGGTTTAGGGTTTAGGGTTTAGGGTTTAGGGTTTAGGGTTTAGGGTTTAGGGTTTAGGGTTTAGGGTTTAGGGTTTAGGGTTTAGGGTTTAGGGTTTAGGGTTTAGGGTTTAGGGTTTAGGGTTTAGGGTTTAGGGTTTAGGGTTTAGGGTTTAGGGTTTAGGGTTTAGGGTTTAGGGTTTAGGGTTTAGGGTTTAGGGTTTAGGGTTTAGGGTTTAGGGTTTAGGGTTTAGGGTTTAGGGTTTAGGGTTTAGGGTTTAGGGTTTAGGGTTTAGGGTTTAGGGTTTAGGGTTTAGGGTTTAGGGTTTAGGGTTTAGGGTTTAGGGTTTAGGGTTTAGGGTTTAGGGTTTAGGGTTTAGGGTTTAGGGTTTAGGGTTTAGGGTTTAGGGTTTAGGGTTTAGGGTTTAGGGTTTAGGGTTTAGGGTTTAGGGTTTAGGGTTTAGGGTTTAGGGTTTAGGGTTTAGGGTTTAGGGTTTAGGGTTTAGGGTTTAGGGTTTAGGGTTTAGGGTTTAGGGTTTAGGGTTTAGGGTTTAGGGTTTAGGGTTTAGGGTTTAGGGTTTAGGGTTTAGGGTTTAGGGTTTAGGGTTTAGGGTTTAGGGTTTAGGGTTTAGGGTTTAGGGTTTAGGGTTTAGGGTTTAGGGTTTAGGGTTTAGGGTTTAGGGTTTAGGGTTTAGGGTTTAGGGTTTAGGGTTTAGGGTTTAGGGTTTAGGGTTTAGGGTTTAGGGTTAGGGTTTAGGGTTTAGGGTTTAGGGGTTAGGGGTTAGGGTTTAGGGTTTAGGGGTGTAGGGTTTAGGGTTTAGGGTTAGGGGTTAGGGTTAGGGTAGGGTTTAGGTGTTAGGGGTTTAGGGTTAGGGTGGGTTAGGGTTTGGGTTTAGGGTTTGGGTTTGGGTGGGTTAGGGTGGGTGGGGTTGGGTTGGGTGTTGGGTTGGGTTTGGGGTGGTGGGTTAGGGGTTAGGGTGGGGTTGGGGTTAGGGGTGTGGGTGTGGGTGTGGGGTGGGTTGGGTTGGGGGTGGGTTGGGTGTTGGGGGTGTGGGGTTGGGGGTTGGGGGTGTGGGTTGTGGGTGGGTGGTGGGGTGGGGTGGGTGGGGTTTGGGGGTGGGTGGGGTGGTGGGTGGGGTGGGGGTGGTTGGGGTGTGGGGTGTGGTTTGGGTTGGTGGGTTGGGGGTGGGGTTGGTTGGGTGGTGGGTTGGGGTGTGGGGGTGGGTGTGGGGTTGGGGTTGGGTTGGGGTTTGGGGTGGGTTGGGGGTGGGTGGGGTTGGGGGTGGGGGTGGGGGGTGGGTTGGGGTGTGGGGTGGTGGGTGTGGGTGTGGGGTGGGTGGGTGGGGGTAGGGTGGGGTGGGTTGGGTGGTGGGGTTGGGTTAGGGGTGGTGGGGTTGGGGTGGTGGGTAGGGGGTGTGGGGGTTGGGTGGGGGTAGGGTTTAGGGTTTTAGGGTTTAGGGTTTAGGGTTAGGTTTAGGGTTTAGGGTCTTAGGGTTGGTAGGGTTTAGGGTTTAGGGTATAGGGTTAGGTTTAGGGTTTAGGGTTTAGGGTTTAGGGTTAGGGTTAGGGTTTAGGGTTTAGGGTTTTAGGGTTTAGGGTTTAGGGTTTAGGGGTTTAGGGTTAGGGTTAGGTTTAGGGTTTAGGGTTTAGGGTTAGGGTTTAGGGTTTAGGGTTTAGGGTTTAGGGTTTAGGGTTTAGGGTTTAGGGTTTAGGGTTTAGGGTTTAGGGTTTAGGGTTTAGGGTTTAGGGTTTAGGGTGTTAGGGTTTAGGGTTTAGGGTTTAGGGTTTAGGGTTTAGGGTTAGGGTTTAGGGTTTAGGGTTTAGGGGTTTAGGGTTTAGGGTTTAGGGTTTAGGTTAGGGTTTAGGGTTTAGGGTTTAGGGTTTAGGGTTTAGGGTTTAGGGTTTAGGGTTTAGGGTTTAGGGTTTAGGGTTTAGGGTTTAGGGTTTAGGGTTTAGGGTTTAGGGTTTAGGGTTTAGGGTTTAGGGTTTAGGGTTTAGGGTTTAGGGTTTAGGGTTTAGGGTTTAGGGTTTAGGGTTTAGGGTTAGGGTTTAGGGTTTAGGGGTTTAGGGGTTTGGTTAGGGTTAGGGTTTAGGGTTTAGGGTTTAGGGTTAGGGTTTAGGGTTTAGGGTTTAGGGTTTAGGGTTTAGGTTTAGGGTTAGGGTTTTAGGGTTTAGGGGTTAGGGTTTAGGGTTTAGGGTTTAGGGGTTAGGGTTTAGGGTTTAGGGTTTAGGGTTTAGGGGTTTAGGGTTTAGGGTTTAGGGTTTTAGGGTTTAGGGTTAGGGTTTAGGGTTTAGGGTTTAGGGTTTAGGGTTTAGGTTTAGGGTTTAGGGTTTAGGGTTTAGGGTTTAGGGTTTAGGGTTTAGGGTTTAGGGTTGTAGGGTTTAGGGTTTAGGGTTTAGGGTTTAGGGTTTAGGGTTTAGGGTTAGGGTTTAGGGTTAGGGTTTAGGGTTTAGGGTTTAGGGTTAGGGTTTAGGGTTTAGGGTTTAGGGTTTAGGGTTTAGGTTAGGTTTAGGGTTAGGGTTTAGGGTTTAGGGTTTAGGGTTTAGGGTTAGGGTTTAGGGTTTAGGGTTTAGGGTTTAGGGTTTAGGGTTAGGGTTTAGGGTTTAGGGTTTAGGGTTTAGGGTTTAGGGTTTAGGGTTTAGGGTTTAGGGTTTAGGGTTTAGGGTTTAGGGTTTAGGGTTTAGGGTTTAGGGTTTAGGGTTTAGGGTTTAGGGTTTAGGGTTTAGGGTTTAGGGTTAGGGTTTAGGGTTTAGGGTTAGGGTTTAGGGTTTAGGGTTAGGGTTTAGGGTTTAGGGTTTAGGGTTTAGGGTTTAGGGTTTAGGGTTAGGGTTTAGGGTTAGGGTTTAGGGTTTAGGGTTTAGGGTTTAGGGTTTAGGGTTAGGGTTTAGGGTTTAGGGTTTAGGGTTTAGGGTTTAGGGTTTAGGGTTTAGGGTTTAGGGTTTAGGGTTTAGGGTTTAGGGTTTTGGGTTTAGGGTTTAGGGTTAGGGTTTAGGGTTTAGGGTTTAGGGTTTAGGGTTTAGGGTTTAGGGTTTAGGGTTTAGGGTTTAGGTTTAGGGTTTAGGGTTTTGGTTTAGGGTTAGGTTTAGGGTTTAGGGTTTAGGGTTTAGGGTTTGGGTTTAGGGTTTAGGGTTTAGGGTTAGGGTTTAGGGTTAGGGTTTAGGGTTTAGGGTTTAGGGTTTAGGGTTTAGGGTTTAGGGTTTAGGGTTTAGGGTTTAGGGTTTAGGGTTTAGGGTTTAGGGTTTAGGGTTAGGGTTTAGGGTTTAGGGTTTAGGGTTAGGGTTTAGGGTTTAGGTTAGGGTTAGGGTTTAGGGTTTAGGGTTAGGTTTAGGGTTTAGGGTTTAGGGTTTAGGGTTTAGGGTTTAGGGTTTAGGGTTTAGGGTTTAGGGTTTAGGGTTTAGGGTTTAGGGTTTAGGTTTTAGGGTTTAGGGTTTAGGGTTTAGGTTTAGGGTTTAGGGTTTAGGGTTAGGGTTTAGGGTTTAGGTTTAGGGTTTAGGGTTTAGGGTTAGGGTTTAGGGGTTTAGGGTTTAGGGTTTAGGGTTTAGGGTTTAGGGTTTAGGGTTTAGGGTTTAGGGTTTAGGGTTTAGGGTTTAGGGTTTAGGGTTTAGGGTTTAGGGTTTAGGGTTTAGGGTTTGGTTTAGGGTTAGGGTTTAGGGTTTAGGGTTAGGGTTTAGGGTTTAGGGTTTAGGGTTTAGGGTTTAGGGTTTAGGGTTTAGGGTTTAGGGTTAGGGTTTAGGGTTTAGGGTTTAGGGTTTAGGGTTTAGGGTTTGGTTTAGGGTTTAGGGTTTAGGGTTTAGGGTTTAGGGTTTAGGGTTTAGGGTTTAGGGTTTAGGGTTTAGGGTTTAGGGTTTAGGGTTAGGGTTTAGGTTTAGGGTTTAGGGTTAGGGTTTAGGGTTTAGGGTTTAGGGTTTAGGGTTTAGGGTTTAGGGTTTAGGGTTTAGGGTTTAGGGTTTAGGGTTTAGGGTTTAGGGTTTAGGGTTAGGGTTTAGGGTTAGGGTTTAGGGTTTAGGGTTTAGGGTTTAGGGTTTAGGGTTTAGGGTTTAGGGTTTAGGGTTTAGGGTTTAGGGTTAGGGTTTAGGGTTTAGGGTTTAGGGTTTAGGTTTAGGGTTAGGGTTTAGGGTTAGGGTTAGGGTTTAGGGTTAGGGTTTAGGGTTTAGGGTTTAGGGTTTAGGGTTTAGGGTTTAGGGTTTAGGGTTTAGGGTTTAGGTTTAGGGTTTAGGGTTTAGGGTTTAGGGTTTAGGGTTTAGGGTTTAGGGTAGGTTAGGGTTTAGGGTTTAGGGTTTAGGGTTTAGGTTTAGGGTTAGGGTTTAGGTTAGGGTTAGGGTTTAGGGTTTAGGGTTTAGGGTTTAGGGTTTAGGGTTTAGGGTTTAGGGTTTGGGTTTAGGGTTTAGGGTTTAGGGTTTAGGGTTTAGGGTTTAGGGTTTAGGGTTTAGGGTTTAGGGTTTAGGGTTTAGGGTTTAGGGTTTAGGGTTTAGGGTTAGGGTTTAGGGTTTAGGGTTTAGGGTTTAGGGTTTAGGGTTTAGGGTTTAGGTGGTTAGGGTTTAGGGTTTAGGGTTTAGGGTTTAGGGTTTAGGGTTTAGGGTTAGGGTTTAGGGTTTAGGGTTTAGGGTTTAGGGTTTGGGTTTAGGGTTTAGGGTTAGGGTTTAGGGTTTAGGGTTTAGGGTTTAGGGTTTAGGGTTTAGGGTTAGGGTTTAGGGTTTAGGGTTTAGGGTTTTAGGGTTAGGGTTTAGGGTTTAGGGTTTAGGGTTAGGGTTGTTAGGGTTTAGGGTTTAGGGTTTAGGGTTTAGGGTTTAGGGTTAGGGTTTAGGGTTTAGGGTTTAGGGTTTAGGGTTTAGGGTTTAGGTTTAGGGTTTAGGGTTTAGGGTTTAGGGTTTAGGGTTTAGGGTTTAGGGTTTAGGGTTTAGGGTTTAGGGTTTAGGGTTTAGGGTTTAGGGTTTAGGGTTTAGGGTTTAGGGTTTAGGGTTTAGGGTTTAGGGTTTAGGGTTTAGGGTTTAGGGTTAGGGTTTAGGGTTTAGGGTTTAGGGTTTAGGGTTTAGGGTTTAGGGTTTAGGGTTTAGGGTTTAGGGTTTAGGGTTTAGGGTTTAGGGTTTAGGGTTTAGGGTTTAGGGTTTAGGGTTTAGGGTTTAGGGTTTAGGGTTTAGGTTTAGGGTTTAGGGTTTAGGGTTTAGGGTTTAGGGTTTAGGGTTTAGGGTTTAGGGTTTAGGGTTTAGGGTTTAGGGTTAGGGTTTAGGGTTTAGGGTTTAGGGTTTAGGGTTTAGGGTTTAGGGTTTAGGGTTAGGGTTTAGGGTTTAGGGTTTAGGGTTTAGGGTTTAGGGTTTAGGGTTTAGGGTTTAGGGTTTAGGGTTTAGGGTTTAGGGTTGTTAGGGTTTAGGGTTTAGGGTTTAGGGTTTAGGGTTTAGGGTTTAGGGTTTAGGGTTTAGGGTTTAGGGTTTAGGGTTTAGGGTTTAGGGGTTTAGGGTTTAGGGTTTAGGGTTTAGGGTTTAGGGTTTAGGGTTTAGGGTTTAGGGTTTAGGGTTTAGGGTTTAGGTTTAGGGTTTAGGGTTTAGGGTTTAGGGTTTAGGGTTTAGGGTTTAGGGTTTAGGGTTTAGGGTTTAGGGTTAGGGTTTAGGGTTTAGGGTTTTAGGGTTTAGGGTTTAGGGTTAGGGTTAGGTTTAGGGTTTAGGGTTTAGGGTTTAGGGTTTAGGGTTAGGGTTTAGGGTTTAGGGTTTAGGGTTTAGGGTTTAGGGTTTAGGGTTTAGGGTTTAGGGTTTAGGGTTTAGGGTTTAGGGTTTAGGGTTTAGGGTTTAGGGTTTAGGGTTTAGGGTTTAGGGTTTAGGGTTTAGGGTTTAGGGTTTAGGGTTTAGGGTTTAGGGTTTAGGGTTTAGGGTTTAGGGTTTAGGGTTTAGGGTTTAGGGTTTAGGGTTTAGGGTTTAGGGTTTAGGGTTTAGGGTTTAGGTTTAGGGTTTAGGGTTTAGGGTTTAGGGTTTTAGGGTTTAGGGTTTAGGGTTTAGGGTTTAGGGTTTAGGGTTTAGGGTTTAGGGTTTAGGGTTTAGGGTTTAGGGTTTAGGGTTTAGGGTTTAGGGTTTAGGGTTTAGGGTTTAGGGTTTAGGGTTTAGGGTTTAGGGTTTAGGGTTTAGGGTTTAGGGTTTAGGGTTTAGGGTTTAGGGTTTAGGGTTTAGGGTTTAGGGTTTAGGGTTTAGGGTTTAGGGTTTAGGTTTAGGGTTTAGGGTTTAGGGTTTAGGGTTTAGGGTTTAGGGTTTAGGGTTTAGGGTTTAGGGTTTAGGGTTTAGGGTTTAGGGTTTAGGGTTAGGGTTTAGGGTTTAGGGTTTAGGGTTTAGGGTTTAGGGGTTTAGGGTTTAGGGTTTAGGGTTTAGGGTTTAGGGTTTAGGGTTTAGGGTTTAGGGTTTAGGGTTTAGGTTAGGGTTTAGGGTTTAGGGTTTAGGGTTTAGGGTTTAGGGTTTAGGGTTTAGGGTTTAGGGTTTAGGGTTTAGGGTTTAGGGTTAGGGTTTAGGGTTTAGGGTTTAGGGTTTAGGGTTTAGGGTTTAGGGTTTAGGTTTAGGGTTTAGGGTTTAGGGTTTAGGGTTAGGGTTTAGGGTTTAGGGTTTAGGGTTTAGGGTTAGGGTTTAGGGTTTAGGGTTTAGGGTTTAGGGTTTAGGGTTTAGGGTTTAGGGTTTAGGGTTTAGGGTTTAGGGTTTAGGGTTTAGGGTTTAGGGTTTAGGGTTTAGGGTTTAGGGTTTAGGGTTTAGGGTTTAGGGTTTAGGGTTTAGGTTTAGGGTTTAGGGTTTAGGGTTTAGGGTTTAGGGTTAGGGTTTAGGGTTTAGGGTTTAGGGTTTAGGGTTTAGGGTTTAGGGTTTAGGGTTTAGGGTTTAGGGTTTAGGGTTTAGGGTTTAGGGTTTAGGGTTTAGGGTTTAGGGTTTAGGGTTTAGGGTTTAGGGTTTAGGGTTTAGGGTTTAGGGTTTAGGGTTTAGGGTTTAGGGTTTGGTTAGGGTTTAGGGTTTAGGGTTTAGGGTTTAGGGTTTAGGGTTTAGGGTTTAGGGTTAGGGTTTAGGGTTTAGGGTTTAGGGTTTAGGGTTTAGGGTTTAGGGTTTAGGGTTTAGGGTTTAGGGTTTAGGGTTTAGGGTTAGGGTTTAGGGTTTAGGGTTTAGGGTTTAGGGTTTAGGGTTTAGGGTTTGGGTTTAGGGTTTAGGGTTTAGGGTTTAGGGTTTAGGGTTTAGGGTTTAGGGTTTAGGGTTTAGGGTTTAGGGTTTAGGGTTTAGGGTTTAGGGGTTTAGGGTTTAGGGTTTAGGGTTTAGGGTTTAGGGTTTAGGGTTTAGGGTTAGGTTTAGGGTTTAGGGTTTAGGGTTTAGGGTTAGGGTTTAGGGTTTAGGGTTTAGGGTTAGGGTTTAGGGTTTAGGGTTTAGGGTTTAGGGTTTAGGGTTTAGGGTTTAGGTTAGGTTTAGGGTTTAGGGTTTAGGGTTTAGGGTTTAGGGTTTAGGGTTTAGGGTTTAGGGTTTAGGGTTTAGGGTTTAGGGTTTTAGGGTTTAGGGTTTAGGGTTTAGGGTTTAGGGTTTAGGGTTTAGGGTTTAGGGTTTAGGGTTTAGGGTTTAGGGTTTAGGGTTTAGGGTTTAGGGTTTAGGGTTTAGGGTTTAGGGTTTAGGGTTTAGGGTTTAGGGTTTAGGGGTTTAGGGTTTAGGGTTTAGGGTTTAGGGTTTAGGGTTTAGGGTTTAGGGTTTAGGGTTTAGGGTTTAGGGTTTAGGGTTTAGGGTTTAGGGTTTAGGGTTTAGGGTTTAGGGTTAGGGTTTAGGGTTTAGGGTTTAGGGTTTAGGGTTTAGGTTTAGGGTTTAGGGTTTAGGTTTAGGGTTTTAGGGTTTAGGGTTTAGGGTTTAGGGTTTAGGGTTTAGGGTTTAGGGTTTAGGGTTTAGGTTTAGGGTTAGGGTTTAGGGTTTAGGGTTTAGGGTTTAGGGTTTAGGGTTAGGGTTTAGGGTTTAGGGTTTAGGGTTTAGGGTTTAGGGTTTAGGGTTTAGGGTTTAGGGTTTAGGGTTTAGGGTTTAGGGTTTAGGGTTTAGGGTTTAGGGTTTAGGGTTTAGGTTTAGGGTTTAGGGTTTAGGGTTTAGGGTTTAGGGTTTTAGGGTTTAGGGTTTAGGGTTTAGGGTTTAGGGTTTAGGTTTAGGGTTTAGGGTTTAGGGTTTAGGGTTTAGGGTTTAGGGTTTAGGGTTTAGGGTTTAGGTTTAGGGTTTTAGGGTTTAGGGTTTAGGGTTTAGGGTTTAGGTTTAGGTTTAGGGTTTAGGGTTTAGGGTTTAGGGTTTAGGGTTTAGGGTTTAGGGTTAGGGTTAGGGTTTTTAGGGTTTAGGGTTTAGGTTTAGGGTTTAGGGTTTAGGGTTTAGGGTTTAGGGTTTAGGGTTTAGGTTTAGGGTTTAGGTTTAGGGTTTAGGGTTTTGGGTTAGGTTTAGGGTTTAGGGTTTAGGGTTTAGGTTTAGGGTTTAGGGTTAGGGTTTAGGGTTTAGGGTTTAGGTTTAGGGTTTAGGGTTTAGGGTTTAGGGTTTAGGGTTTAGGGTTTAGGGTTTAGGGTTTAGGGTTTAGGGTTTAGGGTTTAGGGTTTAGGGTTTAGGGTTTAGGGTTTAGGGTTAGGGTTTAGGGTTTAGGGTTAGGGTTTAGGGTTTAGGTTTAGGGTTTAGGGTTTAGGGTTTAGGGTTTAGGGTTTAGGGTTTAGGGTTTAGGGTTTAGGGTTTAGGGTTAGGTTAGGGTTTAGGGGTTTAGGGTTAGGGTTTAGGGTTTAGGGTTTTTAGGGTTTAGGGTTTAGGGTTTAGGGTTTAGGGTTTAGGGTTTAGGGTTTAGGGTTTAGGGTTTAGGGTTTAGGGTTTAGGGTTTAGGGTTTAGGGTTTAGGGTTTAGGGTTTAGGGTTTAGGGTTTAGGGTTTAGGGTTTAGGGTTTAGGGTTTAGGGTTTAGGGTTTAGGGTTTAGGGTTTAGGGTTTAGGTTTAGGGTTTAGGGTTTAGGGTTTAGGGTTTAGGGTTTAGGGTTTAGGTTTAGGGTTTAGGGTTTAGGGTTTAGGGTTTAGGGTTTAGGGTTTAGGGTTTAGGTTTAGGGTTTAGGTTTAGGGTTTAGGGTTTAGGGTTTAGGGTTTAGGGTTTAGGGTTTAGGGTTTAGGTTTAGGGTTTAGGGTTTAGGGTTTAGGGTTTAGGGTTTAGGGTTTAGGGTTTAGGGTTTAGGGTTTAGGGTTAGGGTTTAGGGTTTAGGGTTTAGGGTTTAGGGTTTAGGGTTTAGGGTTTAGGGTTTAGGGTTTAGGGTTTAGGGTTTAGGGTTTAGGGTTTAGGGTTTAGGGTTTAGGGTTTAGGGTTAGGGTTTAGGGTTTAGGGTTTAGGGTTTAGGGTTTAGGGTTTAGGGTTTAGGGTTTAGGTTTAGGGTTTAGGGTTTAGGGTTTAGGGTTTAGGGTTTAGGGTTTAGGGTTTAGGGTTTAGGGTTTAGGGTTTAGGGTTAGGGTTTAGGGTTTAGGGTTTAGGGTTTAGGGTTTAGGGTTTAGGGTTTAGGGTTAGGGTTTAGGGTTTAGGGTTTAGGGTTTAGGGTTTAGGGTTAGGGTTTAGGGTTTAGGGTTTAGGGTTTAGGGTTTAGGGTTTAGGGTTTAGGGTTTAGGGTTTAGGGTTAGGGTTTAGGGTTTAGGTTTAGGGTTTAGGTTTAGGGTTTAGGGTTTAGGGTTTAGGGTTAGGGTTTAGGGTTTAGGGTTTAGGGTTTAGGGTTTAGGGTTTTAGGGTTTAGGGTTTAGGGTTTAGGGTTTAGGGTTTAGGGTTTAGGGTTTAGGGTTTAGGGTTTAGGGTTAGGGTTTAGGGTTTAGGGTTTAGGGTTTAGGGTTTAGGGTTTAGGGTTTAGGGTTTAGGGTTTAGGGTTTAGGTTTAGGGTTTAGGGTTTAGGGTTTAGGGTTTAGGGTTTAGGGTTTAGGGTTTAGGGTTAGGGTTTAGGGTTTAGGGTTTAGGTTTAGGGTTTAGGGTTTAGGGTTTAGGGTTTAGGGTTTAGGGTTAGGTTTAGGTTTAGGGTTTAGGGTTTAGGGTTAGGGTTAGGGTTTAGGGTTTAGGGTTTAGGGTTTAGGTTTAGGGTTTAGGGTTTAGGGTTTAGGGTTTAGGGTTTAGGGTTTAGGGTTTAGGGTTTAGGGTTTAGGGTTAGGTTTAGGGTTTAGGGTTTAGGGTTTAGGGTTTAGGGTTTAGGGTTAGGGTTTAGGGTTTAGGGTTTAGGTTTAGGGTTTAGGTTTAGGGTTTAGGGTTTAGGGTTAGGGTTTAGGGTTTAGGGTTTAGGGTTTAGGGTTTAGGGTTTAGGGTTTAGGGTTAGGGTTTAGGGTTTAGGGTTTAGGGTTTAGGGTTTAGGGTTTAGGGTTTAGGGTTTAGGGTTTAGGGTTTAGGGTTTAGGGTTAGGGTTTAGGGTTTAGGGTTTAGGGTTTAGGGTTTAGGGTTAGGTTTAGGGTTTAGGGTTTAGGGTTTAGGGTTTAGGGTTTAGGGTTTAGGGTTTAGGTTTAGGGTTTAGGGTTTAGGGTTTAGGGTTTAGGGTTTAGGGTTTAGGTTTAGGGTTTAGGGTTTAGGGTTTAGGGTTTAGGGTTTAGGGTTTAGGGTTTAGGGTTTTAGGGTTTAGGGTTTAGAGGGTTTAGGGTTTAGGGTTTAGGGTTTAGGGTTTAGGGTTTAGGGTTTAGGGTTTAGGGTTAGGGTTTAGGGTTTAGGGTTAGGGTTTAGGGTTTAGGGTTTAGGGTTTAGGGTTTAGGGTTTAGGGTTTAGGGTTTAGGGTTTAGGGTTTAGGGTTTAGGGTTAGGGTTTTAGGGTTTAGGGTTTAGGGTTAGGGTTTAGGTTTAGGGTTTAGGGTTTAGGGTTTAGGGTTTAGGGTTAGGGTTTAGGGTTTAGGGTTTAGGGTTTAGGGTTTAGGGTTTAGGGTTTAGGGTTTAGGGTTTAGGGTTTAGGGTTTAGGGTTTAGGGTTTAGGGTTTAGGGTTTAGGGTTTAGGGTTTAGGTTTAGGGTTTAGGGTTTAGGGTTTAGGGTTTAGGGTTTAGGGTTTAGGGTTTAGGGTTTAGGGTTTAGGGTTTAGGGTTTAGGGTTTAGGTTAGGGTTTAGGGTTTAGGGTTTAGGGTTTAGGGTTTAGGGTTTAGGGTTTAGGGTTTAGGGTTTAGGGTTTAGGGTTTTGAGGGTTTAGGGTTTAGGGTTTAGGGTTTAGGGTTTAGGGTTTAGGGTTTAGGGTTTAGGTTTAGGGTTTAGGGTTTAGGGTTTAGGGTTTAGGGTTAGGGTTTAGGGTTAGGTTTAGGGTTTAGGGTTAGGGTTTAGGGTTTAGGGTTTAGGGTTTAGGGTTTAGGGTTTAGGGTTTAGGGTTTAGGGTTTAGGGTTTAGGGTTTAGGGTTTAGGGTTTAGGGTTTAGGGTTTAGGGTTTAGGGTTTAGGGTTAGGGTTTAGGTTTAGGGTTTAGGGTTTAGGGTTTAGGGTTTAGGGTTTAGGGTTTAGGTTTAGGGTTTAGGGTTTAGGGTTTAGGGTTTAGGGTTTAGGGTTTAGGGTTTAGGGTTTAGGGTTTAGGGTTTAGGTTTAGGGTTTAGGGTTTAGGGTTTAGGGTTTAGGGTTTAGGGTTTAGGGTTTAGGGTTTAGGGTTTAGGGTTAGGGTTTAGGGTTTAGGGTTTAGGGTTTAGGGTTTAGGGTTTAGGGTTTTAGGTTTAGGGTTTAGGGTTTAGGGTTTAGGGTTTAGGTTTAGGGTTTAGGGTTTAGGGTTTAGGGTTTAGGGTTTAGGGTTTAGGGTTTAGGGTTTAGGGTTTAGGGTTTAGGGTTTAGGGTTTAGGGTTTAGGGTTTAGGGTTTAGGGTTTAGGGTTAGGGTTTAGGGTTTAGGGTTTAGGGTTTAGGTTTAGGGTTTAGGGTTTAGGGTTTAGGGTTTAGGTTTAGGGTTTAGGGTTTAGGGTTTAGGGTTTAGGGTTTAGGGTTTAGGGTTTAGGGTTTAGGGTTTAGGGTTTAGGGTTTAGGTTTAGGGTTTAGGGTTTAGGGTTTAGGGTTTAGGGTTTAGGGTTTAGGGTTTAGGGTTTAGGGTTTAGGGTTTAGGGTTTAGGGTTTAGGGTTTAGGGTTAGGGTTTAGGGTTTAGGGTTTAGGGTTTAGGGTTTAGGGTTTAGGGTTTAGGGTTTAGGGTTTAGGGTTTAGGGTTTAGGGTTTAGGGTTTAGGTTTAGGTTTAGGGTTTAGGGTTTAGGGTTTAGGGTTTAGGGTTTAGGGTTTAGGGTTTAGGGTTTAGGGTTTAGGGTTTAGGGTTTAGGGTTTAGGGTTTAGGTTTAGGGTTTAGGGTTTAGGGTTTAGGGTTTAGGGTTTAGGGTTTAGGTTTAGGGTTTAGGGTTTAGGGGTTTAGGGTTTAGGGTTTAGGGTTTAGGGTTTAGGGTTTAGGGTTTAGGGTTTAGGGTTTAGGGTTTAGGGTTTAGGGTTTAGGGTTTAGGGTTTAGGGTTTAGGGTTTAGGGTTTAGGGTTAGGGTTTAGGGTTTAGGGTTTAGGGTTTAGGGTTTAGGGTTTTAGGGTTTAGGGTTTAGGGTTTAGGGTTTAGGGTTTAGGGTTTAGGGTTTAGGGTTTAGGGTTTAGGGTTTAGGTTTAGGGTTTAGGGTTTAGGGTTTAGGGTTTAGGGTTTAGGGTTTAGGGTTTAGGGTTTAGGGTTTAGGGTTTAGGGTTTAGGGTTTAGGGTTTAGGGTTTAGGGTTTAGGGTTTAGGGTTTAGGGTTTAGGGTTAGGGTTTAGGGTTTAGGGTTTAGGGTTTAGGTTAGGTTTAGGGTTTAGGGTTTAGGGTTTAGGGTTTAGGGTTTAGGGTTTAGGTTTAGGGTTTAGGGTTTAGGGTTTAGGGTTTAGGGTTTAGGGTTTAGGTTTAGGGTTTAGGGTTTAGGGTTTAGGGTTTAGGTTTAGGGTTTAGGGTTTAGGGTTTAGGGTTTAGGGTTTAGGGTTTAGGTTTAGGGTTTAGGGTTTAGGGTTTAGGGTTTAGGGTTTAGGGTTTAGGGTTTAGGGTTTAGGTTTAGGGTTTAGGGTTTAGGGTTTAGGGTTTAGGGTTTAGGGTTTAGGGTTTAGGTTTAGGGTTTAGGGTTTAGGGTTTAGGGTTAGGGTTTAGGGTTTAGGGTTTAGGGTTTAGGGTTTAGGGTTTAGGGTTTAGGGTTTAGGGTTTAGGGTTTAGGGTTTAGGGTTTAGGGTTTAGGGTTAGGGTTTAGGTTAGGGTTTAGGGTTTAGGGTTTAGTTAGGTTTAGGGTTTAGGGTTTAGGGTTTAGGGTTTAGGGTTTAGGGTTTAGGGTTTAGGGTTTAGGGTTTAGGGTTTAGGGTTTAGGGTTTAGGGTTTAGGGTTTAGGGTTTAGGTTAGGTTTAGGGTTTAGGGTTTAGGGTTTAGGGTTAGGGTTTAGGGTTTAGGGTTTAGGGTTTAGGGTTTAGGTTTAGGGTTAGGGTTTAGGGTTTTAGGGTTTAGGGTTTAGGGTTTAGGGTTTAGGGTTTAGGGTTTAGGGTTTAGGGTTTAGGGTTAGGGTTTAGGGTTTAGGGTTTAGGGTTTAGGGTTTAGGGTTTAGGGTTTTAGGGTTTAGGGTTTAGGGTTTAGGGTTTAGGGGTTTAGGGTTTAGGGTTTAGGGTTTAGGTTTAGGGTTTAGGGTTTAGGGTTTAGGGTTTAGGGTTTAGGGTTTAGGGTTAGGGTTTAGGGTTTAGGGTTTAGGGTTTAGGGTTTAGGGTTTAGGGTTTAGGGTTTAGGGTTTAGGGTTAGGGTTTAGGGTTTAGGGTTTAGGGTTTAGGGTTTAGGGTTTAGGGTTTAGGGTTTAGGGTTAGGGTTTAGGGTTTAGGGTTTAGGGGTTAGGGTTTAGGGTTTAGGGTTTAGGGTTTTAGGGTTTAGGGTTAGGGTTTAGGGTTTAGGGTTTAGGGTTTAGGGTTTAGGGTTAGGGTTTAGGGTTTAGGGTTTAGGGTTTAGGGTTTAGGTTTAGGTTTAGGGTTTAGGGTTTAGGGTTAGGGTTAGGGTTTAGGGTTTAGGGTTTAGGGTTTAGGGTTTAGGGTTTAGGGTTTAGGGTTTAGGGTTTAGGGTTTAGGGTTTAGGGTTTAGGGTTAGGTTAGGGTTTAGGGTTTAGGGTTTAGGGTTTAGGGTTTAGGGTTTAGGGTTTAGGGTTTAGGGTTTAGGGTTAGGGTTTAGGGTTTAGGGTTTAGGGTTTAGGGTTTAGGGTTTAGGGTTTAGGGTTTAGGGTTTAGGGTTTAGGGTTAGGGTTTAGGGTTTAGGGTTTAGGGTTTAGGGTTTAGGGTTTAGGGTTAGGGTTTAGGGTTTAGGGTTTAGGGTTTAGGGTTTAGGGTTTAGGGTTTAGGGTTAGGGTTTAGGGTTTAGGGTTTAGGGTTAGGGTTTAGGGTTTAGGGTTTAGGGTTAGGGTTTAGGGTTTAGGGTTTAGGGTTGTTAGGGTTTAGGGTTTAGGGTTTAGGGTTTAGGGTTTAGGGTTTAGGGTTTAGGGTTAGGGTTTAGGGTTTAGGGTTTAGGGTTTAGGGTTAGGGTTTAGGGTTTAGGGTTTAGGGTTTAGGGTTTAGGGTTTAGGGTTTAGGGTTTAGGGTTTAGGGTTAGGGTTTAGGGTTTAGGGTTTAGGGTTTAGGGTTTAGGGTTTAGGGTTTAGGGTTTAGGGTTAGGGTTTAGGGTTTAGGGTTTAGGGTTAGGTTTAGGGTTAGGGTTTAGGGTTTAGGGTTTAGGGTTTAGGGTTTAGGTTAGGTTTAGGTTTAGGGTTTAGGGTTTAGGGTTTAGGGTTTAGGGTTTAGGGTTTAGGGTTAGGGTTTAGGGTTTAGGGTTTAGGGTTTAGGGTTTAGGGTTTAGGGTTTAGGGTTTAGGGTTTAGGGTTTAGGGTTAGGGTTTAGGGGTTTAGGGTTTAGGTTTAGGGTTTAGGGTTTAGGGTTTAGGGTTTAGGGTTTAGGGTTTAGGGTTTAGGGTTTAGGGTTTAGGGTTTAGGGTTTAGGGTTTTAGGGTTAGGGTTTAGGGTTAGGGTTTAGGGTTTAGGGTTTAGGGTTTAGGGTTTAGGGTTTAGGGTTTAGGGTTAGGGTTTAGGGTTTAGGGTTTAGGGTTAGGGTTTAGGGTTTAGGGTTTAGGGTTTAGGGTTTAGGGTTTAGGGTTTAGGGTTTAGGTTTAGGGTTTAGGGTTTAGGGTTTAGGGTTTAGGGTTTAGGTTTAGGGTTTAGGTTTAGGGTTTAGGGTTTGGTTAGGGTTAGGGTTTAGGGTTTAGGGTTTAGGGTTTAGGGTTTAGGGTTTAGGGTTTAGGGTTTAGGGTTTAGGGTTTAGGGTTTAGGGTTTAGGGTTTAGGGTTTAGGGTTTAGGGTTTAGGGTTAGGGTTTAGGGTTTAGGGTTTAGGTTTAGGGTTTAGGGTTTAGGGTTTAGGGTTTAGGGTTTAGGGTTAGGGTTTAGGGTTTAGGGTTTAGGGTTTAGGGTTTAGGGTTTAGGGTTTAGGGTTTAGGGTTTAGGGTTTTAGGGTTTAGGGTTAGGGTTTAGGGTTTAGGGTTTAGGGTTTAGGGTTTAGGGTTTAGGGTTTAGGGTTTAGGGTTTAGGGTTTAGGTTTAGGGTTTAGGGTTTAGGGTTTAGGGTTTAGGGTTTAGGGTTTAGGGTTTAGGGTTTAGGGTTTAGGGTTTAGGGTTTAGGGTTAGGGTTTAGGGTTTAGGGTTTAGGGTTTAGGGTTTAGGGTTTAGGGTTTAGGGTTTAGGGTTAGGGTTTAGGGTTAGGGTTTAGGGTTTAGGGTTTAGGGTTTAGGGTTTAGGGTTTAGGGTTTAGGGTTTAGGGTTTAGGGTTTAGGGTTTAGGGTTTAGGGTTTAGGGTTAGGGTTTAGGGTTTAGGGTTTAGGGTTTAGGGTTAGGGTTTAGGGTTTAGGGTTTAGGGTTTAGGGTTAGGGTTAGGGTTTAGGGTTTAGGGTTTAGGGTTTAGGGTTTAGGGTTTAGGGTTTAGGGTTTAGGGTTTAGGGTTTAGGGTTTAGGGTTTAGGGTTTAGGGTTTAGGGTTTAGGGTTAGGGTTTAGGGTTTAGGGTTTAGGGTTTAGGGTTTAGGGTTTAGGGTTTAGGGTTTAGGGTTTAGGGTTTAGGGTTTAGGGTTTAGGGTTTAGGGTTTAGGGTTTAGGGTTTAGGGTTTAGGGTTTAGGGTTTAGGGTTTAGGGTTAGGGTTTAGGGTTTAGGGTTTAGGGTTTAGGGTTTAGGGTTTAGGGTTTAGGGTTTAGGGTTTAGGGTTAGGGTTTAGGGTTTAGGGTTTAGGGTTTAGGGTTAGGGTTTAGGGTTTAGGGTTTAGGGTTAGGGTTTAGGGTTTAGGGTTTAGGGTTAGGGTTTAGGGTTTAGGGTTTAGGGTTTAGGGTTAGGGTTTAGGGTTTAGGGTTTAGGGTTTAGGGTTTAGGGTTTAGGGTTTAGGGTTTAGGGTTAGGGTTTAGGGTTTAGGGTTAGGGTTTAGGGTTTAGGGTTTAGGGTTTAGGGTTAGGGTTTAGGGTTTAGGGTTTAGGGTTTAGGGTTTAGGGTTTAGGGTTTAGGGTTTAGGGTTTAGGGTTAGGGTTTAGGGTTTAGGGTTAGGTTAGGGTTTAGGGTTAGGGTTTAGGGTTTAGGGTTTAGGGTTAGGGTTTAGGGTTTAGGGTTTAGGGTTTAGGGTTTAGGGTTTAGGGTTTAGGGTTTAGGGTTTAGGGTTTAGGGTTTAGGGTTTAGGGTTTAGGGTTTAGGGTTTAGGGTTTAGGGTTTAGGGTTAGGGTTTAGGGTTTAGGGTTTAGGGTTAGGGTTTAGGGTTTAGGGTTTAGGGTTTAGGGTTTAGGGTTTAGGGTTTAGGGTTTAGGGTTTAGGGTTAGGGTTTAGGGTTTAGGGTTAGGGTTTAGGGTTTAGGGTTTAGGGTTTAGGGTTTAGGGTTTAGGGTTTAGGGTTTAGGGTTTAGGGTTAGGGTTTAGGGTTTAGGGTTAGGGTTAGGGTTTAGGGTTTAGGGTTTAGGGTTTAGGGTTTAGGGTTTAGGGTTTAGGGTTTAGGGTTAGGGTTTAGGGTTAGGGTTTAGGGTTTAGGGTTTTAGGGTTTAGGGTTTAGGGTTTAGGGTTTAGGGTTTAGGGTTTTAGGGTTTAGGGTTTAGGGTTTAGGGTTTAGGGTTTAGGGTTTAGGGTTTAGGGTTAGGGTTTAGGGTTTAGGGTTTAGGGTTTAGGGTTTAGGGTTTAGGGTTTAGGGTTTAGGGTTTAGGGTTTAGGGTTTAGGGTTTAGGGTTTAGGGTTAGGGTTAGGGTTTAGGGTTAGGTTAGGGTTTAGGGTTTAGGGTTTAGGGTTTAGGGTTTAGGGTTTAGGGTTTAGGTTAGTTAGGTTTAGGGTTTAGGGTTTAGGGTTAGGTTAGGGTTTAGGGTTTAGGGTTTAGGGTTTAGGGTTTAGGGTTAGGGTTTAGGGTTTAGGGTTTAGGGTTTAGGGTTTAGGGTTTAGGGTTAGGGTTTAGGGTTTAGGGTTTAGGTTAGGGTTTAGGGTTTAGGGTTTAGGGTTTAGGGTTTAGGGTTTAGGGTTTTAGGGTTTAGGGTTTAGGGTTTAGGGTTAGGGTTTAGGGTTTAGGGTTTAGGGTTTAGGGTTTAGGGTTTAGGGTTAGGGTTTAGGGTTTAGGGTTTAGGGTTTAGGGTTTAGGGTTTAGGGTTTAGGGTTAGGTTTAGGGTTTAGGGTTAGGGTTTAGGGTTTAGGGTTTAGGGTTTAGGGTTTAGGGTTAGGGTTTAGGGTTTAGGGTTAGGGTTTAGGGTTTAGGGTTTAGGGTTTAGGGTTTAGGGTTTAGGGTTTAGGGTTTAGGTTTAGGGTTTAGGGTTTAGGGTTAGGGTTTAGGGTTTAGGGTTTAGGGTTAGGGTTTAGGGTTTAGGGTTTAGGGTTTAGGGTTTAGGGTTTAGGGTTTAGGGTTTAGGGTTAGGGTTTAGGGTTTAGGGTTTAGGGTTAGGGTTTAGGGTTTAGGGTTAGGGTTTAGGGGTTTAGGGTTTAGGGTTTAGGGTTTAGGGTTTAGGGTTTAGGGTTTAGGGTTTAGGGTTTAGGGTTTAGGGTTTAGGGTTTAGGGTTTAGGGTTTTAGGGTTTAGGGTTAGGGTTTAGGGTTTAGGGTTTAGGGTTTAGGGTTAGGGTTTAGGGTTTAGGGTTAGGGTTAGGGTTTAGGGTTTAGGGTTAGGGTTTAGGGTTTAGGGTTTAGGTTTAGGGTTTAGGGTTTAGGGTTTAGGGTTTAGGGTTTAGGGTTTAGGGTTTAGGGTTTAGGGTTTAGGGTTTAGGGTTTAGGTTTAGGGTTTAGGGGTTTAGGGTTTAGGGTTTAGGGTTTAGGGTTTAGGTTTAGGGTTTAGGGTTAGGGTTTAGGGTTTAGGGTTTAGGGGGTTTAGGGTTTAGGGTTTAGGGTTTA

**5AL**

TTTGG**TTTAGGG**TTTAGGGTTTAGGCTTTAGGTTTAGGGTTTAGGTTTAGGGTTTAGGCTTTAGGTTTAGGGTTTAGGTTTAGGGTTTAGGGTTTAGGGTTTAGGGTTTAGGGTTTAGGGTTTAGGGTTTAGGGTTTAGGGTTTAGGGTTTAGGGTTTAGGGTTTAGGGTTTAGGGTTTAGGGTTTAGGGTTTAGGGTTTAGGGTTTAGGGTTTAGGGTTTAGGGTTTAGGGTTTAGGGTTTAGGGTTTAGGGTTTAGGGTTAGGGTTAGGGTTAGGGTTTAGGGTTTAGGGTTTAGGGTTTAGGGTTAGGGTTTAGGGTTTAGGGTTTAGGGTTTAGGGTTTAGGGTTTAGGGTTTAGGTAGGGTTTAGGGTTAGGGTTAGGGTAGGGTTAGGGTTAGGGTTTAGGGTTAGGGTTAGGGTTTAGGGTTAGGGTTTAGGGTTTAGGGTTAGGGTTTAGGGTTTAGGGTTTAGGGTTTAGGGTTTAGGGGTTTAGGTTTAGGGTTTAGGGTTTTAGGGTTTAGGGTTTAGGGTTTAGGGTTTAGGGTTAGGGTTTAGGGTTTAGGGTTAGGGTTTAGGGTTAGGGTTTAGGGTTTAGGGTTTAGGGTTTAGGGTTTAGGGTTTAGGGTTAGGGTTTAGGGTTTAGGGTTTAGGGTTTAGGGTTTAGGGTTTAGGGTTTAGGGTTTAGGGTTTAGGGTTTAGGGTTTAGGGTTTAGGGTTTAGGGTTTAGGGTTTAGGGTTTAGGGTTTAGGGTTTAGGGTTTAGGTTTAGGGTTTAGGGTTTAGGGTTTAGGGTTAGGGTTTAGGGTTAGGGTTTAGGGTTTAGGGTTTAGGGTTTAGGGTTAGGGTTAGGGTTTAGGGTTTAGGGTTTAGGGTTTAGGGTTTAGGTTTAGGGTTTAGGGTTTAGGGTTTAGGGTTTAGGGTTTAGGGTTAGGGTTTAGGGTTTAGGGTTTAGGGTTTAGGGTTTAGGGTTTAGGGTTTGGGTTTAGGGTTTAGGGTTTAGGGGTTTAGGGTTTAGGGTTTAGGGTTTAGGGTTTAGGGTTTAGGGTTTAGGGTTTAGGGTTTAGGGTTTAGGGTTAGGGTTAGGGTTTAGGGTTTAGGGTTTAGGGTTTAGGGTTTAGGGTTAGGGTTTAGGGTTTAGGGTTTAGGGTTTAGGGTTTAGGGTTAGGGTTTAGGGTTTAGGGTTAGGGTTTAGGGTTTAGGGTTAGGGTTTAGGGTTTAGGGTTAGGGTTTAGGGTTTAGGGTTTAGGGTTAGGGTTTAGGGTTTAGGGTTTAGGGTTTAGGGTGGGTTTAGGGTTTAGGGTTTAGGGTTTAGGGTTTAGGGTTAGGGTTAGGGTTTAGGGTTTAGGGTTTAGGGTTTAGGGTTTAGGGTTTAGGGTTAGGGTTTAGGGTTTAGGGTTTAGGGTTTAGGGTTTAGGGTTTAGGGTTTAGGGTTTAGGGTTAGGGTTTAGGGTTTAGGGTTTAGGGTTTAGGGTTTTAGGGTTTAGGGTTAGGGTTTGGTTTAGGGTTTAGGGTTAGGGTTTAGGGTTTAGGGTTTAGGGTTTAGGGTTTAGGGTTTAGGGTTTAGGGTTTAGGGTTTAGGGTTTAGGGTTTAGGGTTTAGGGTTAGGGTTTAGGGTTTAGGGTTTAGGGTTTTAGGGTTTAGGGTTTAGGGTTTAGGGTTGGTTAGGGTTTAGGGTTTAGGGTTTAGGGTTAGGGGGTTTAGGGTTAGGGTTTAGGGTTTAGGGTTTAGGGTTTTAGGGTTTAGGGTTTAGGGTTTAGGTTAGGGTTTAGGGTTTAGGGTTTAGGGTTTAGGGGTTAGGGTTTAGGGTTTAGGGTTTAGGGTTAGGGTTTAGGGTTAGGGTTTAGGGTTTAGGGTTTAGGGTTTAGGGTTTAGGGTTAGGGTGGTTTAGGGTTTAGGGTTTAGGGGTTTAGGGTTTAGGGTTAGGGTTTAGGGTTTAGGGTTAGGGTTTAGGGTTTAGGGTTTAGGGTTTAGGGTTAGGGTTTAGGGTTTAGGGTTTAGGGTTTAGGGTTAGGGTGGTTTAGGGTGTTAGGGTTTAGGGTTTAGGGTTTAGGGTTAGGGTTAGGGTTTAGGGTTTAGGGTTTAGGGTTTAGGGTTAGGGTTAGGGTTTAGGGTTAGGGTTTAGGGTTTAGGGTTAGGGTTTAGGGTTTAGGGTTAGGGTTTAGGGTTTAGGGTTTAGGGTTTAGGGTTTAGGGTTAGGGTTTAGGGTTTAGGGTTTAGGGTTTAGGGTTAGGGTTTAGGGTTAGGGTTTAGGGTTTAGGGTTTAGGGTTTAGGGTTTAGGGTTTAGGGTTTAGGGTTTAGGGTTAGGGTTTAGTGGGTTTAGGGTTTAGGGTTAGGGTTAGGGTTTAGGGTTTAGGGTTTAGGGTTAGGGTTTAGGGTTTAGGGTTAGGGTTTAGGGTTAGGGTTAGGGTTTAGGGTTAGGTTAGGGTTAGGGTTAGGGTTTAGGGTTAGGGTTAGGGTTTAGGGTTTAGGGTTAGGGTTTAGGGTTTAGGGTTAGGGTTTAGGGTTAGGGTGGGTAGGGTTTAGGGTTTAGGGTTTAGGGTTTAGGGTTTAGGGTTTAGGTTTAGGGTTTAGGGTTAGGTTTAGGGTTAGGGTTTAGGGTTTAGGGTTAGGGTTTAGGGTTTAGGGTTTAGGGTTAGGGTTTAGGGTTTAGGGTTTAGGGTTTAGGGTTTAGGGTTAGGGTTTAGGGTTTAGGGTTTAGGGTTTAGGGTTTAGGGTTTAGGGTTTAGGGTTTGGTTTAGGGTTTAGGGTTAGGGTTTAGGGTTTAGGGTTAGGGTTTAGGGTTTAGGGTTTAGGGTTTAGGGTTTAGGGTTAGGGTTTAGGGTTTAGGGTTTAGGGTTTAGGGTTAGGGTTTAGGGTTAGGGTTTAGGGTTAGGGTTTAGGGTTTAGGGTTTAGGGTTTAGGGTTTAGGGTTAGGGTTTAGGGTTAGGGTTTAGGGTTAGGGTTTAGGGTTTAGGGTTTAGGGTTTAGGGTTTAGGGTTTAGGGTTAGGGTTTAGGGTTTAGGGTTTAGGGTTTAGGGTTTAGGGTTTAGGGTTTAGGGTTTAGGGTTTAGGGTTTAGGGTTAGGGTTTAGGGTTTAGGGTTTAGGGTTTAGGGTTTAGGGTTTAGGGTTTAGGGTTAGGGTTAGGGTTTAGGGTTTAGGGTTTAGGGTTTAGGGTTTAGGGTTTAGGGTTAGGGTTTAGGGTTAGGGTTTAGGGTTTAGGGTTTAGGGTTTAGGGTTTAGGGTTTAGGGTTTAGGGTTTAGGGTTTAGGGTTTAGGGTTTAGGGTTTAGGGTTAGGTTTAGGGTTTAGGGTTTAGGGTTTAGGGTTTAGGGTTTAGGGTTAGGGTTAGGGTTAGGGTTTAGGGTTTAGGGTTTAGGGTTTAGGGTTTAGGGTTTAGGGTTTAGGGTTAGGGTTTAGGGTTTAGGGTTTAGGGGTTTAGGGTTTTAGGGTTTAGGGTTTAGGGTTTAGGGTTTAGGGTTTGGGTTTAGGGTTTAGGGTTTAGGGTTTAGGGTTTAGGGTTTAGGGTTTAGGGTTTAGGGTTTAGGGTTTAGGGTTTAGGGTTTAGGGTTTAGGGTTTAGGGTTTAGGGTTTAGGGTTTAGGGTTAGGGTTTAGGGTTTAGGGTTAGGGTTTAGGGTTTAGGGTTTAGGGTTTAGGGTTAGGGTTTAGGGTTTAGGGTTTAGGGTTTAGGGTTTAGGGTTTAGGGTTTAGGGTTTAGGGTTTAGGGTTAGGGTTTAGGGTTAGGGTTAGGGTTTAGGGTTTAGGGTTTAGGGTTTAGGGTTTAGGGTTTAGGGTTTAGGGTTTAGGGTTTAGGGTTAGGGTTAGGGTTAGGGTTTAGGGTTTAGGGTTAGGGTTTAGGGTTTAGGGTTAGGGTTTAGGGTTTAGGGTTAGGGTTTAGGGTTTAGGGTTTAGGGTTTAGGGTTTAGGGTTTAGGGTTGGGTTTAGGGTTTAGGGTTTAGGGTTTAGGGTTAGGGTTTAGGGTTTAGGGTTTAGGGTTTAGGGTTTAGGTTTAGGGTTTAGGGTTAGGTTTAGGGTTTAGGGTTTAGGGTTTAGGGTTTAGGGTTAGGGTTTAGGGTTTAGGGTTTAGGGTTTAGGGTTTAGGGTTAGGGTTTAGGGTTTAGGGTTTAGGGTTTAGGGTTTAGGGTTTAGGGTTTAGGGTTTAGGGTTTAGGGTTTAGGGTTTAGGGTTTAGGGTTTAGGGTTTAGGGTTTAGGGTTAGGGTTTAGGGTTTAGGGTTTAGGGTTTAGGGTTTAGGGTTTAGGGTTAGGGTTTAGGGTTTAGGGTTTAGGGTTGTAGGGTTAGGGTTTAGGGTTTAGGGTTTAGGGTTTAGGGTTTAGGGTTTAGGGTTAGGGTTTAGGGTTTAGGGTTTAGGGTTTAGGGTTTAGGGTTTAGGGTTAGGGTTTAGGGTTTAGGGTTTTAGGGTTTAGGGTTTAGGGTTTAGGGTTAGGGTTTAGGGTTTAGGGTTTAGGGTTTAGGGTTTAGGGTTTAGGGTTTAGGGTTTAGGGTTTAGGGTTAGGGTTAGGGTTGGTTAGGGTTTAGGGTTTAGGGTTTAGGGTTTAGGGTTTAGGGTTGTAGGGTTTAGGGTTTAGGGGTTAGGGTTTAGGGTTAGGGTTAGGGTTAGGTAGGGTTTAGGGTTAGGGTTTAGGGTTTAGGGGTTTAGGGTTTAGGGTTAGGGTTTAGGGTTTAGGGTTAGGGTTTAGGGTTTAGGGTTTAGGGTTTTAGGGTTTGGTAGGTTTAGGGTTTAGGGTTTAGGGTTTAGGGTTAGGGTTTAGGGTTTAGGGTTTAGGGTTTAGGGTTTAGGGTTTAGGGTTTAGGGTTTGGGTTTAGGGTTTAGGGTTTAGGGTTTAGGGTTTAGGGTTTGGGTTTAGGGTTTAGGGTTTAGGGTTTGGGTTTAGGGTTAGGGTTTAGGGTTTAGGTTGGTTTAGGGTTTAGGGTTTAGGGTTTAGGTAGGGTTTAGGGTTAGGGTTTAGGGTTTAGGGTTGTTAGGGTTAGGGTTTAGGGGTTTAGGGTTTAGGGTTTAGGGTTTAGGGTTTAGGGTTTAGGGTTTAGGGTTTAGGGTTTAGGGTTTGGGTTAGGGTTTAGGGTTTAGGGTTTAGGGTTTAGGGTTTAGGGTTTAGGGGTTTAGGGTTTAGGGTTTAGGGTTTAGGGTTTAGGGTTTAGGGTTTAGGGTTTAGGGTTTAGGGTTTAGGGTTTAGGGTTTAGGGTTAGGGTTTAGGGTTTAGGGTTTAGGGTTTAGGGTTTAGGGTTTAGGGTTTAGGGTTTAGGGTTTAGGGTTTAGGGGTTTAGGGTTTAGGGTTTAGGGTTTAGGGTTTAGGGTTTAGGGTAGGTTTAGGGTTTAGGGTTTAGGGTTTAGGGTTTAGGGTTTAGGGGTTTAGGGTTTAGGGTTTAGGGTTTAAGGGTTTAGGGTTTAGGGTTTAGGGTTTAGGGTTTAGGGTTTAGGGTTAGGGTTTAGGGTTTAGGGTTTAGGGTTTAGGGTTAGGGTTTAGGGTTAGGGTTTAGGGTTTAGGGTTTAGGGTTTAGGGTTTAGGGTTTAGGGTTTAGGGTTTAGGGTTTAGGGTTTAGGGTTTAGGGTTTAGGGTTTAGGGTTTAGGGTTAGGGTTTAGGGTTTAGGGTTTAGGGTTTAGGGTTAGGGTTTAGGGTTTAGGGGTTTAGGGTTTAGGGTTTAGGGTTTAGGGTTTAGGGTTTAGGGTTTAGGGTTAGGGTTTAGGGTTTAGGGTTTAGGGTTTAGGGTTTAGGGTTTAGGGTTTAGGGTTTAGGGTTTAGGGGTTAGGGTTTAGGGTTTAGGGTTTAGGTTTAGGGTAGGTTAGGGTTTAGGGTTAGGGTTTAGGGTTTAGGGTTTAGGGTTTAGGGTTTAGGGTTTAGGGTTTAGGGTTTAGGGTTTAGGGTTTAGGGTTTAGGGTTTAGGGTTTAGGGTTAGGGTTTAGGGGTTTAGGGTTTAGGGTTTAGGTTAGGGTTAGGGTTTAGGGTTTAGGGTTTAGGGTTTAGGGTTTAGGGTTTAGGGTTTAGGGTTAGGGTTTAGGGTTTAGGGTTTAGGGTTTAGGGTTTAGGGTTAGGGTTTAGGGTTTAGGGTTAGGGTTTAGGGTTTAGGGTTTAGGGTTTAGGTTTAGGGTTTAGGGTTTAGGAGGGTTTAGGGTTTAGTAGGGTTTAGGGTTTAGGGTTTAGGGTTTAGGGTTTAGGGTTTAGGGTTTAGGGTTTAGGGTTTAGGGTTTAGGGTTTAGGGTTTAGGTTTAGGGTTTAGGGTTTAGGGTTAGGGTTTAGGGTTTAGGGTTTAGGGTTTAGGGTTTAGGGTTAGGTTTAGGGTTTAGGGTTTAGGGTTTAGGGTTTAGGGTTTAGGGTTTAGGGTTTAGGGTTTAGGGATTAGGGTTTAGGGTTTAGGGTTAGGGTTTAGGGTTTAGGGGTTTAGGGTTAGGGTTTAGGGTTAGGGTTTAGGGTTTAGGGGTTTAGGGTTTAGGGTTTAGGGTTTAGGGTTTAGGTTTAGGGTTTAGGGTTTAGGGTTTAGGGTTAGGGTTTAGGGTTTAGGGTTTAGGGTTTAGGGTTTAGGGTTTAGGGTTTAGGGTTTAGGGTTTAGGGTTTAGGGTTTAGGGTTTAGGGTTTAGGGTTTAGGGTTAGGGTTTAGGGTTTAGGGTTTAGGGTTTTGGGTTTAGGGTTTAGGGTTTAGGGTTTAGGGTTTAGGGTTAGGGTTTAGGGTTTAGGTTTAGGGTTTAGGGTTTAGGGTTTAGGGTTTAGGGTTTAGGGTTAGGGTTTAGGGTTTAGGGTTTAGGGTTTAGGGTTTAGGGTTAGGTTAGGGTTTAGGGTTTAGGGTTTAGGGTTTAGGGTTTAGGGTTAGGGTTTAGGGTTTAGGGTTTGGGTTTAGGGTTTAGGGTTAGGGTTTAGGGTTTAGGGTTTAGGGTTTAGGGTTTAGGGTTTAGGGTTTAGGGTTTAGGGTTTTAGGGTTTAGGGTTTAGGGTTTAGGGTTTAGGGTTTAAGGGTTTAGGGTTTAGGGTTTAGGGTTTAGGGTTTAGGGTTGTTAGGGTTAGGGTTTAGGGTTTAGGGTTTAGGGTTTAGGGTTTAGGGTTAGGGTTTAGGGTTTAGGGTTTAGGGTTTAGGGTTTAGGGTTTAGGGTTTAGGGTTTAGGGTTAGGGTTTAGGGTTTAGGGTTTAGGGTTTAGGGTTTAGGGTTTAGGGTTTAGGGTTTAGGGTTTAGGGTTTAGGGTTTAGGGTTTAGGGTTTAGGGTTTAGGGTTTAGGGTTTAGGGTTTAGGGTTTAGGGTTTAGGGTTTAGGGTTAGGGTTTAGGGTTTAGGGTTTAGGGTTTAGGGTTTAGGGTTTAGGGTTTAGGGTTTAGGGGTTTAGGGTTTAGGGTTTAGGGTTAGGGTTTTAGGGTTAGGGTTTAGGGTTTAGGGTTTAGGGTTTAGGGTTTAGGGTTTAGGGTTTAGGGTTTAGGGTTTAGGGTTAGGGTTTAGGGTTTAGGGTTTAGGGTTTAGGGTTTAGGGTTTAGGGTTTAGGGTTTAGGGTTAGGGTTTAGGGTTTAGGGTTTAGGGTTTAGGTTTAGGGGTTTAGGGTTTAGGGTTTAGGGTTTAGGGTTTAGGGTTTAGGGTTTAGGGTTTAGGGTTTAGGGTTTAGGGTTTAGGGTTTAGGGTTTAGGGTTAGGGTTTAGGGTTTAGGGTTTAGGGTTTAGGGTTTAGGTTTAGGGTTTAGGGTTAGGGTTTAGGGTTTAGGGTTTAGGGTTTAGGGTTGATAGGGTTTAGGGTTTAGGGTTTAGGGTTTAGGGTTTAGGGTTTAGGGTTTTAGGGTTTAGGGTTTAGGGTTTAGGGTTTAGGTTTAGGGTTTAGGGTTTAGGGTTTAGGGTTTAGGGTTTAGGGTTTAGGGTTTAGGGTTTAGGGTTTAGGGTTTAGGGTTTAGGGTTTAGGGTTTAGGGTTTAGGGTTTAGGGTTAGGGTTTAGGGTTTAGGGTTTAGGGTTTAGGGTTTAGGGTTTAGGGTTAGGGTTTAGGGTTTAGGGTTTAGGGTTAGGGTTTAGGGTTTAGGGTTAGGGTTTAGGGTTTAGGGTTAGGGTTTAGGGTTTAGGGTTTAGGGTTTAGGGTTTAGGGTTTAGGGTTTAGGGTTTAGGGTTTAGGGTTTAGGGTTAGGGTTTAGGGTTTAGGGTTTAGGGTTTAGGGTTTAGGGTTTAGGGTTTAGGGTTTAGGGTTTAGGGTTTAGGGTTTAGGGGTTTAGGGGTTAGGGTTTAGGGTTTAGGGTTTAGGGTTAGGGTTTAGGGTTTAGGGTTTAGGGTTAGGGTTTAGGGTTTAGGGTTTAGGGTTTAGGGTTTAGGGTTTAGGGTTTAGGGTTTAGGGTTTAGGTAGGGTTTAGGGTTAGGGTTTAGGGTTTAGGGTTTAGGGTTTAGGGTTTAGGGTTTAGGGTTTAGGGTTTAGGGTTTAGGGTTTAGGGTTTAGGGTTTAGGGTTTAGGGTTTAGGGTTTAGGGTTTAGGGTTTAGGGTTTAGGGTTTAGGGTTTAGGGTTTAGGGTTAGGTTAGGGTTAGGGTTTAGGGTTTAGGGTTTAGGGGTTTAGGGTTTAGGGTTTAGGGTTTAGGGTTTAGGGTTTAGGGTTTAGGGTTTAGGGTTTAGGGTTTAGGGTTTAGGGTTTAGGGTTTAGGGTTTAGGGTTTAGGGTTTAGGGTTAGGGTTTAGGGTTAGGGTTTTAGGGTTTAGGGTTTAGGGTTTAGGGTTAGGGTTTAGGGTTTAGGGTTTAGGGTTTAGGGTTAGGGTTTAGGGTTAGGGTTTAGGGTTTAGGGTTTAGGGTTAGGGTTTAGGGTTTAGGGTTTAGGGTTAGGGTTTAGGGTTTAGGGTTTAGGGTTTAGGGTTTAGGGTTTAGGGTTTAGGGTTAGGGTTTAGGGTTTAGGGTTTAGGGTTTAGGGTTTAGGGTTTAGGGTTTAGGGTTTAGGGTTAGGGTTTAGGGTTAGGGTTTAGGGTTTAGGGTTTAGGGTTTAGGGTTTAGGGTTTAGGGTTTAGGGTTTAGGGTTAGGGTTAGGGTTTAGGGTTTAGGGTTAGGGTTTAGGGTTTAGGGTTTAGGGTTAGGGTTTAGGGTTTAGGGTTTAGGGTTTAGGGTTAGGGTTTAGGGTTTAGGGTTTAGGGTTTAGGGTTTAGGGTTTAGGGTTTAGGGTTTAGGGTTTAGGGTTTAGGGTTAGGGTTTAGGGTTTAGGGTTTAGGGTTTAGGGTTAGGGTTTAGGGTTAGGGTTTAGGGTTTAGGGTTTAGGGTTTAGGGTTTAGGGTTTAGGGTTTAGGGTTTAGGGTTAGGGTTTAGGGTTTAGGGTTTAGGGTTTAGGGTTTAGGGTTTAGGGTTTAGGGTTTAGGGTTTAGGGTTAGGGTTAGGGTTTAGGGTTTAGGGTTAGGGTTTAGGGTTTGGGTTTAGGGTTTAGGGTTAGGGTTTAGGGTTTAGGGTTTAGGGTTTAGGGTTTAGGGTTTAGGGTTTAGGGTTTAGGGTTTAGGGTTTAGGGTTTAGGGTTTAGGGTTAGGGTTTAGGGTTTAGGGTTAGGGTTTAGGGTTTAGGGTTTAGGGTTTAGGGTTTAGGGTTTAGGGTTTAGGGTTTAGGGTTTAGGGTTTAGGGTTAGGGTTTAGGGTTTAGGGTTAGGGTTAGGGTTTAGGGTTTAGGGGTTAGGGTTTAGGGTTTAGGGTTAGGGTTTAGGGGTTTAGGGTTTAGGGTTTAGGGTTTAGGGTTTAGGGTTTAGGGTTTAGGGTTTAGGGTTTAGGGTTTAGGGTTTAGGGTTAGGGTTTAGGGTTAGGGTTTAGGGTTTAGGGTTTAGGGTTTAGGGTTTAGGGTTTAGGGTTTAGGGTTTAGGGTTTAGGGTTTAGGGTTTAGGGTTTAGGGTTTAGGGTTAGTAGGGTTTAGGGTTTAGGGTTTAGGGTTTAGGGTTTAGGGTTTAGGGTTTAGGGTTTAGGGTTTAGGGTTTAGGGTTTAGGGTTTAGGGTTTAGGGTTAGGTTTAGGGTTTAGGGGTTAGGGTTTAGGGTTTAGGGTTTAGGGTTTAGGGTTTAGGGTTTAGGGTTTAGGGTTTAGGGTTTAGGGTTAGGGTTTAGGGTTTAGGGTTTAGGGTTTAGGGTTTAGGGTTTAGGGTTTAGGGTTTAGGGTTTAGGGTTTAGGGTTTAGGGTTTAGGGTTTAGGGTTTAGGGTTTAGGGTTTAGGGTTTAGGGTTTAGGGTTTAGGGTTTAGGGTTTAGGGTTAGGGTTTAGGGTTTAGGGTTTAGGGTTTAGGGTTTAGGGTTTAGGGTTTAGGGTTTAGGGTTTAGGGTTTAGGGTTTAGGGTTTAGGGTTTAGGGTTTAGGGTTTAGGGTTTAGGGTTTAGGGTTTAGGGTTTAGGGTTAGGGTTTAGGGTTTAGGGTTTAGGGTTTAGGGTTTAGGGTTTAGGGTTTAGGGTTTAGGGTTTAGGGTTTAGGGTTTAGGGTTTAGGGTTAGGGTTTAGGGTTTAGGGTTTAGGGTTTAGGGTTTAGGGTTTAGGGTTTAGGGTTTAGGGTTTAGGGTAGGTAGGGTTTAGGGTTTAGGGTTTAGGGTTTAGGGTTAGGGTTTAGGGTTTTAGGGTTTAGGGTTTAGGGTTTAGGGTTTAGGGTTTAGGGTTTAGGGTTTAGGGTTAGGGTTTAGGGTTTAGGGTTTAGGGTTTAGGGTTTAGGGTTAGGGTTTAGGGTTTAGGGTTTAGGGTTTAGGGTTTAGGGTTTAGGGTTTAGGGTTTAGGGTTTAGGGTTTAGGGTTTAGGGTTTAGGGTTTAGGGTTTAGGGTTTAGGGTTTAGGGTTTAGGGTTTAGGGTTTAGGGTTAGGGTTTAGGGTTTAGGGTTTAGGGTTTAGGGTTTAGGGTTTAGGGTTTAGGGTTTAGGGTTTAGGGTTTAGGGTTTAGGGTTGTAGGTTTAGGGTTTAGGGTTAGGGTTTAGGGTTTAGGGTTTAGGGTTTAGGGTTTAGGGTTTAGGGTTTAGGGTTTAGGGTTTAGGGTTAGGGTTTAGGGTTTAGGGTTTAGGGTTTAGGGTTTAGGGTTTAGGGTTTAGGGTTAGGGTTTAGGGTTTAGGGTTTAGGGTTTAGGGTTTAGGGTTTAGGGTTTAGGGTTTAGGGTTTAGGGTTTAGGGTTTAGGGTTTAGGGTTTAGGGTTTAGGGTTTAGGGTTAGGGTTTAGGGTTTAGGGTTTAGGGTTTAGGGTTTAGGGTTTAGGGTTTAGGGTTTAGGGTTTAGGGTTTAGGGTTAGGTTTAGGGTTTAGGGTTTAGGGTTTAGGGTTTAGGGTTTAGGGTTAGGGTTTAGGGTTTAGGGTTTAGGGTTTAGGGTTTAGGGTTAGGGTTTAGGGTTTAGGGTTGGGTTAGGGTTTAGGGTTTAGGGTGTTAGGGTTTAGGGTTAGGGTTTAGGGTTTAGGGTTTAGGGTTTAGGGTTAGGGTTTAGGGTTAGGGTTTAGGGTTTAGGGTTTAGGGTTTAGGGTTAGGGGTTTAGGGTTTAGGGTTTAGGGTTAGGGTTTAGGGTTTAGGGTTTAGGGTTTAGGGTTTAGGGTTTAGGGTTTAGGGTTTAGGGTTTAGGGTTAGGGTTTAGGGTTTAGGGTTTAGGGTTTAGGGTTTAGGGTTTAGGGTTTAGGGTTTGGGTTTAGGGTTAGGGTTTAGGGTTTAGGGTTTAGGGTTTAGGGTTTAGGGTTTAGGGTTTAGGGTTAGGGTTTAGGGTTTAGGGTTAGGGTTTAGGGTTTAGGGTTTAGGGTTTAGGGTTTAGGGTTTAGGGTTAGGGTTTAGGGTTTAGGGTTTAGGGTTTAGGGTTTAGGGTTTAGGGTTTAGGGTTAGGGTTTAGGGTTTAGGGTTTAGGGTTTAGGGTTAGGGTTTAGGGTTTAGGGTTAGGGTTTAGGGTTTAGGGTTTAGGTTAGGGTTTAGGGTTTAGGGTTTAGGGTTTAGGGTTTAGGGTTTAGGGTTTAGGGGTTTAGGGTTTAGGGTTTAGGGTTTAGGGTTTAGGGTTAGGGTTTAGGTTTAGGGTTTAGGGTTTAGGGTTAGGGTTTAGGGTTTAGGGTTAGGGTTTAGGGTTTAGGGTTTAGGGTTTAGGGTTTAGGGTTTAGGGTTTAGGGTTTAGGGGTTAGGGGTTTAGGGTTTAGGGTTTAGGGTTTAGGGTTTAGGGTTTAGGGTTTAGGGTTAGGGTTTAGGGTTTAGGGTTTAGGGGTTTAGGGTTTAGGGGTTTAGGGTTTAGGGTTTAGGGTTTAGGGTTTTGGGGTTTAGGGTTTAGGTTTAGGGGTTAGGGTTTAGGGTTTAGGGTTTAGGGTTTAGGGTTTAGGGTTTAGGGTTAGGGTTTAGGGTTAGGGTTTAGGGTTTAGGGTTTAGGGTTTAGGGTTTAGGGTTTAGGGTTTAGGGTTAGGTTTAGGGTTTAGGGTTTAGGGTTTAGGGTTAGGGTTTAGGGTTTAGGGTTTAGGGTTTAGGGTTTAGGGTTTAGGGTTTAGGGTTTAGGGTGGGTTTAGGGTTGGTTAGGGTTTAGGGTTTAGGGTTTAGGGTTTAGGGTTTTGGGTTTAGGGTTTAGGGTTAGGGTTTAGGGTTTAGGGTTTTGGGTTTTAGGTTTAGGGTTTAGGGTTTAGGGTTTAGGGTTTAGGGTTTAGGGTTTAGGGTTTAGGGTTAGGGTTTAGGATTAGGGTAGGGTTTAGGGTTTAGGTTTAGGGTTTAGGGTTTAGGGTTTAGGGTTTTAGGGTCTAGGGGTTTAAAGTTTAAAGTTTGGGTTTAAAGTG

**6AL**

TAGGG**TTTAGGG**TTTAGGGTTTAGGGTTTAGGGTTAGGGTTTAGGGTTTAGGGTTTAGGGTTTAGGGTTTAGGGTTTAGGGTTTAGGGTTTAAGGTTAGGGTTTAGGGTTTAGGGTTTAGGGTTTAGGGTTAGGGTTTAGGGTTTAGGGTTTAGGGTTTAGGGTTTAGGGTTTAGGGTTTAGGGTTTAGGGTTTAGGGTTTATAGGGTTTAGGGGTTTAGGGTTTAGGGTTTAGGGTTAGGGTTTAGGGTTTAGGGTTGGGTTTTAGGGTTTAGGGTTTAGGGTTTAGGGTTTTAGGGTTTTAGGGTTTTAGGGTTTTAGGGTTGGTTTTAGGGTTTAGGGTTTAGGGTTTAGGGTTAGGGTTTAGGGTTTAGGGTTTAGGGTTAGGGTTTAGGGTTTAGGGTTTAGGGTTTAGGGTTTAGGGTTAGGGTTTAGGGTTTAGGGTTTAGGGTTTTTAGGGTTTTAGGGTTTAGGGTTTAGGGTTTAGGGTTAGGGTTAGGGTTAGGGTTTAGGGTTTAGGGTTTTAGGGTTAGGGTTTAGGGTTAGGGTTTAGGGTTTAGGGTTAGGGTTTAGGGTTTAGGGTTTGGGGTTAGGGTTTAGGGTTAGGGTTTAGGGTTAGGGTTTAGGGTTTAGGGTTAGGGTTTTTAGGGTTTAGGGTTTTAGGGTTTAGGGTTTAGGGTTAGGGTTTAGGGTTTAGGGTTTAGGGTTTTAGGGTTTAGGGTTTAGGGTTTAGGGTTTAGGGTTTAGGGTTAGGGTTTAGGGTTTAGGGTTTAGGGTTTAGGGTTTTAGGGTTTAGGGTTAGGGTTTAGGGTTTAGGGTTTAGGGTTAGGGTTAGGGTTAGGGTTTAGGGTTTAGGGTTTAGGGTTAGGGTTTAGGTTTTTTAGGGTTTAGGGTTAGGGTTTAGGGGTTTAGGGCTTAGGGTTTAGGGTTTAGGGTTAGGGTTTAGGGTTTAGGGTTTAGGGTTTAGGGTTTAGGGTTAGGGTTTAGGGTTAGGGTTTAGGGTTTAGGGTTTAGGGTTTAGGGTTTAGGGTTTAGGGTTAGGGTTTAGGGTTAGGGTTTAGGGTTTAGGGTTTAGGGTTTTTAGGGTTTAGGGTTTAGGGTTAGGGTTTAGGGTTTAGGGTTTAGGGTTTAGGGTTTAGGGTTTAGGGTTTAGGGTTTAGGGTTTAGGGTTTAGGGTTGGGTTTAGGGTTTAGGGTTTAGGGTTTAGGGTTTAGGGTTTAGGGTTTTAGGGTTTAGGGTTTTAGGGTTTAGGGTTTAGGGTTTAGGGTTTAGGGTTTAGGGTTTAGGGTTTAGGGTTTAGGGTTTAGGGTTTAGGGTTTAGGGTTTAGGGTTTAGGGTTTAGGGTTTAGGGTTTAGGGTTTAGGGTTTAGGGTTTAGGGTTTAGGGTTTAGGGTTTAGGGTTTAGGGTTTAGGGTTTAGGGTTTAGGGTTTAGGGTTTAGGGTTTAGGGTTTAGGGTTTAGGGTTTAGGGTTTAGGGTTTAGGGTTTAGGGTTTAGGGTTTAGGGTTTAGGGTTTAGGGTTTAGGGTTTAGGGTTTAGGGTTTAGGGTTTAGGGTTTAGGGTTTAGGGTTTAGGGTTTAGGGTTTAGGGTTTAGGGTTAGGGTTTAGGGTTTAGGGTTTAGGGTTTAGGGTTTAGGGTTTAGGGTTTAGGGTTAGGGTTTAGGGTTTAGGGTTTAGGGTTTAGGGTTTAGGGTTTAGGGTTTAGGGTTTAGGGTTTAGGGTTTAGGGTTTAGGGTTTAGGGTTTAGGGTTAGGGTTTAGGGTTTAGGGTTTAGGGTTTAGGGTTTAGGGTTTAGGGTTTAGGGTTTAGGGTTTAGGGTTTAGGGTTTAGGGTTTAGGGTTTAGGGTTTAGGGTTTAGGGTTTAGGGTTTAGGGTTTAGGGTTTAGGGTTTAGGGTTTAGGGTTTAGGGTTTAGGGTTTAGGGTTTAGGGTTTAGGGTTTAGGGTTAGGGTTTAGGGTTTAGGGTTTAGGGTTTAGGGTTAGGGTTTAGGGTTTAGGGTTTAGGGTTTAGGGTTTAGGGTTTAGGGTTTAGGGTTTAGGGTTTAGGGTTTAGGGTTTAGGGTTTAGGGTTTAGGGTTTAGGGTTTAGGGTTTAGGGTTTAGGGTTTAGGGTTTAGGGTTAGGGTTTAGGGTTTAGGGTTTAGGGTTTAGGGTTTAGGGTTTAGGGTTTAGGGTTTAGGGTTTAGGGTTTAGGGTTTAGGGTTTAGGGTTTAGGGTTTAGGGTTAGGGTTTAGGGTTTAGGGTTTAGGGTTTAGGGTTTAGGGTTTAGGGTTTAGGGTTTAGGGTTTAGGGTTTAGGTTTTAGGGTTTAGGGTTTAGGGTTTAGGGTTTAGGGTTTAGGGTTTAGGGTTTAGGGTTTAGGGTTAGGGTTTAGGGTTAGGGTTTAGGGTTTAGGGTTTAGGGTTTAGGGTTTAGGGTTTAGGGTTTAGGGTTAGGGTTAGGGTTAGGGTTTAGGGTTTAGGGTTTAGGGTTTAGGGTTTAGGGTTTAGGGTTAGGGTTTAGGGTTTAGGGTTTAGGGTTTAGGGTTAGGGTTTAGGGTTTAGGGTTTAGGGTTTAGGGTTAGGGTTTAGGGTTTAGGGTTAGGGTTTAGGGTTTAGGGTTTAGGGTTTAGGGTTTAGGGTTTAGGGTTTAGGGTTTAGGGTTAGGGTTTAGGGTTTAGGGTTTAGGGTTTAGGGTTAGGGTTTAGGGTTAGGGTTTAGGGTTAGGGTTTAGGGTTTAGGGTTTAGGGTTTAGGGTTAGGGTTTAGGGTTAGGGTTTAGGGTTTAGGGTTTAGGGTTTAGGGTTTAGGGTTTAGGGTTTAGGGTTTAGGGTTAGGGTTTAGGGTTTAGGGTTTAGGGTTTAGGGTTTAGGGTTAGGGTTTAGGGTTTAGGGTTTAGGGTTTAGGGTTTAGGGTTTAGGGTTTAGGGTTTAGGGTTTAGGGTTTAGGGTTAGGGTTTAGGGTTTAGGGTTTAGGGTTTAGGGTTTAGGGTTTAGGGTTTAGGGTTTAGGGTTTAGGGTTTAGGGTTTAGGGTTTAGGGTTTAGGGTTTAGGGTTTAGGGTTTAGGGTTTAGGTTAGGGTTTAGGGTTTAGGGTTTAGGGTTTAGGGTTTAGGGTTTAGGGTTAGGGTTTAGGGTTTAGGGTTTAGGGTTTAGGGTTTAGGGTTTAGGGTTTAGGGTTTAGGGTTTAGGGTTTAGGGTTAGGGTTAGGGTTTAGGGTTTAGGGTTTAGGGTTTAGGGTTTAGGGTTTAGGGTTAGGGTTTAGGGTTTAGGGTTTAGGGTTTAGGGTTTAGGGTTTAGGGTTTAGGGTTAGGGTTTAGGGTTTAGGGTTTAGGGTTTAGGGTTAGGGTTTAGGGTTTAGGGTTTAGGGTTTAGGGTTAGGGTTTAGGGTTTAGGGTTAGGGGTTTAGGGTTTAGGGTTTAGGGTTTAGGGTTTAGGGTTTAGGGTTTAGGGTTTAGGGTTAGGGTTTAGGGTTTAGGGTTTAGGGTTTAGGGTTTAGGGTTTAGGGTTAGGGTTTAGGGGTTTAGGGTTTAGGGTTTAGGGTTAGGGTTTAGGGTTTAGGGTTTAGGGTTTAGGGTTAGGGTTTAGGGTTAGGGTTTAGGGTTTAGGGTTTAGGGTTTAGGTTTAGGGTTTAGGGTTAGGGTTAGGGTTTAGGGTTTAGGGTTTAGGGTTTAGGTTTAGGGTTTAGGGTTAGGGTTTAGGGTTTAGGGTTTAGGGTTTAGGGTTTAGGGTTTAGGGTTAGGGTTTAGGGTTTAGGGTTTAGGGTTTAGGGTTTAGGGTTTAGGGTTTAGGGTTTAGGGTTTAGGGTTAGGGTTAGGGTTTAGGGTTTAGGGTTTAGGTTTAGGGTTTAGGGGTTTAGGGTTTAGGGTTTAGGGTTTAGGGTTTAGGGGTTTAGGGTTTAGGGTTTAGGGTTTAGGGTTTAGGGTTAGGGTTTAGGGTTTAGGGTTAGGGTTAGGGTTTAGGGTTTAGGGTTTAGGGTTAGGGTTTAGGGTTTAGGGTTTAGGGTTTAGGGTTTAGGGTTTAGGGTTTAGGTTAGGGTTTAGGGTTTAGGGTTTAGGGTTTAGGGTTTAGGGTTTAGGGTTTAGGGTTTAGGGTTTAGGGTTTAGGGTTTAGGGTTTAGGGTTTAGGGTTAGGGTTTAGGGTTTAGGGTTTAGGGTTTAGGGTTTAGGGTTTAGGGTTAGGGTTTAGGGTTTAGGGTTTAGGGTTTAGGGTTTAGGGTTTAGGGTTTAGGGTTTAGGGTTTTAGGGTTAGGTTAGGGTTTAGGGTTTAGGGTTTAGGGTTTAGGGTTAGGGTTTAGGGTTTAGGGTTTAGGGTTAGGGTTTAGGGTTTAGGGTTTAGGGTTAGGGTTTAGGGTTTAGGGTTTAGGGTTAGGGTTTAGGGTTTAGGGTTTAGGGTTTAGGGTTAGGGTTAGGGTTTAGGGTTTAGGGTTTAGGGTTTAGGGTTTAGGGTTTAGGGTTAGGGTTAGGGTTTAGGGTTTAGGGTTTAGGGTTTAGGGTTTAGGGTTTAGGGTTAGGGTTTAGGGTTTAGGGTTTAGGGTTTAGGGTTAGGGTTTAGGGTTTAGGGTTTAGGGTTTAGGGTTTAGGGTTAGGGTTTAGGGTTTAGGGTTTAGGGTTTAGGGTTTAGGGTTTAGGGTTTAGGGTTTAGGGTTTAGGGTTTAGGGTTTAGGGTTTAGGGTTAGGGTTTAGGGTTTAGGGTTTAGGGTTTAGGGTTTAGGGTTTAGGGTTTAGGGTTTAGGGTTTAGGGTTTAGGGTTTAGGGTTAGGGTTTAGGGTTTAGGGTTTAGGGTTTAGGGTTTAGGGTTTAGGGTTTAGGGTTTAGGTTTAGGGTTAGGGTTTAGGGTTTAGGTTAGGGTTTAGGGTTTAGGGTTAGGGTTTAGGGTTTAGGTTAGGGTTTAGGGTTAGGGTTTAGGGTTTAGGGTTTAGGGTTTAGGGTTTAGGGTTTAGGGTTTAGGGTTTAGGGTTTAGGGTTTAGGGTTTAGGGTTTAGGGTTTAGGGTTTAGGGTTTAGGGTTAGGGTTTAGGGTTTAGGGTTTAGGGTTTAGGGTTTAGGGTTAGGGTTTAGGGTTTAGGGTTTAGGGTTTAGGGTTTAGGGTTTAGGGTTTAGGGTTTAGGGTTTAGGGTTAGGGTTTAGGGTTTAGGGTTTAGGGTTTAGGGGTTAGGGTTTAGGGGTTTAGGGTTTAGGTTTAGGGTTTAGGGTTTAGGGTTTAGGGTTTAGGGTTAGGGTTTAGGGTTTAGGGTTTAGGGTTTAGGGTTTAGGGTTTAGGGTTTAGGGTTTAGGGTTTAGGGTTTAGGGTTTAGGGTTTAGGGTTTAGGGTTTAGGGTTTAGGGTTTAGGGTTTAGGGTTTAGGGTTAGGGTTTGGGTTTAGGGTTTAGGGTTTAGGGTTTAGGGTTTAGGGTTTAGGGTTTAGGGTTTAGGGGTTTAGGGTTTAGGGTTTAGGGTTTAGGGTTTAGGGTTTAGGGTTAGGGTTTAGGGTTTAGGGTTTAGGGTTTAGGGTTTAGGGTTAGGGTTTAGGGTTTAGGGTTTAGGTTTAGGGTTAGGGTTAGGGTTAGGGTTTAGGGTTAGGGTTTAGGGTTTAGGGTTTAGGGTTTAGGGTTTAGGGTTTAGGGTTTAGGGTTTAGGGTTTAGGGTTTAGGGTTAGGGTTTAGGGTTTAGGGTTAGGGTTTAGGGTTTAGGGTTTAGGGTTTAGGGTTAGGGTTTAGGGTTTAGGGTTAGGGTTTAGGGTTTAGGGTTTAGGGTTTAGGGTTTAGGGTTTAGGGTTAGGGTTTAGGGTTTAGGGTTTAGGGTTAGGGTTTAGGGTTTAGGGTTTAGGGTTTAGGGTTTAGGGTTTAGGGTTTAGGGTAGGGTTAGGGTTTAGGGTTTAGGGTTTAGGGTTTAGGGTTAGGGTTTAGGGTTTAGGGTTTAGGGTTAGGGTTAGGGTTTAGGGTTTAGGGTTTAGGGTTAGGGTTTAGGGTTTAGGGTTTAGGGTTTAGGGTTTAGGGTTTAGGGTTTAGGGTTTAGGGTTTAGGGTTTAGGGTTTAGGGTTTAGGGTTTAGGGTTAGGGTTTAGGGTTTAGGGTTTAGGGTTTAGGGTTAGGGTTTAGGGTTTAGGGTTTAGGGTTTAGGGTTTAGGGTTTAGGGTTTAGGGTTTAGGGTTTAGGGTTTAGGGTTTAGGGTTTAGGGTTTAGGGTTTAGGGTTTAGGGTTTAGGGTTTAGGGTTTAGGGTTTAGGGTTTAGGGTTAGGGTTAGGGTTTAGGGTTTAGGGTTTAGGGTTTAGGGTTAGGGTTTAGGGTTTAGGGTTTAGGGTTTAGGGTTTAGGGTTTAGGGTTTAGGGGTTTAGGGTTTAGGGTTTAGGGTTAGGGTTAGGGTTAGGGTTAGGGTTTAGGGTTTAGGGTTTAGGGTTTAGGGTTAGGGTTTAGGGTTTAGGGTTAGGGTTAGGGTTTAGGGTTTAGGGTTTAGGGTTTAGGGTTTAGGGTTTAGGGTTTAGGGTTAGGGTTTAGGGTTTAGGGTTTAGGGTTTAGGGTTTAGGGTTTAGGGTTAGGGTTTAGGGTTAGGGTTAGGGTTTAGGGTTTAGGGTTAGGGTTAGGGTTTAGGGTTTAGGGTTAGGGTTTAGGGTTTAGGTTAGGGTTAGGGTTTAGGGTTAGGGTTTAGGGTTTAGGGTTTAGGGTTTAGGGTTTAGGGTTTAGGGTTTAGGGTTAGGGTTAGGTTTAGGGTTTAGGGTTAGGGTTTAGGGTTTAGGGTTTAGGGTTAGGGTTAGGGTTTAGGGTTAGGGTTAGGGTTTAGGGTTTAGGGTTAGGGTTAGGGTTTAGGGTTTAGGGTTAGGGTTTAGGGTTTAGGGTTTAGGGTTTAGGGTTTAGGGTTTAGGGTTTAGGGTTTAGGGTTTAGGGTTTAGGGTTAGGGTTAGGGTTAGGGTTTAGGGTTAGGGTTTAGGGTTTAGGGTTTAGGGTTAGGGTTTAGGGTTTAGGGTTTAGGGTTTAGGGTTAGGGTTTAGGGTTTAGGGTTAGGGTTTAGGGTTTAGGGTTAGGGTTTAGGGTTTAGGGTTAGGGTTTAGGGTTTAGGGTTTAGGGTTTAGGGTTTAGGGTTTAGGGTTTAGGGTTAGGGTTAGGGTTTAGGGTTTAGGGTTTAGGGTTTAGGGTTTAGGGTTAGGGTTTAGGGTTTAGGGTTTAGGGTTTAGGGTTAGGGTTTAGGGTTTAGGGTTAGGTTTAGGGTTTAGGGTTTAGGGTTTAGGGTTTAGGGTTAGGGTTTAGGGTTTAGGGTTTAGGGTTTAGGGTTTAGGGTTTAGGGTTAGGGTTAGGGTTAGGGTTTAGGGTTAGGGTTTAGGGTTTAGGGTTTAGGGTTTAGGGTTAGGGTTTAGGGTTTAGGGTTAGGGTTTAGGGTTTAGGGTTTAGGGTTTAGGGTTTAGGGTTTAGGGTTTAGGGTTAGGGTTTAGGGTTAGGGTTTAGGGTTAGGGTTTAGGGTTAGGGTTTAGGGTTAGGGTTTAGGGTTTAGGGTTTAGGGTTTAGGGTTTAGGGTTTAGGGTTTAGGGTTTAGGGTTTAGGGTTTAGGGTTTAGGGTTAGGGTTTAGGGTTTAGGGTTTAGGGTTTAGGGTTTAGGTTTAGGGTTTAGGGTTTAGGGTTTAGGGTTTAGGGTTTAGGGTTTAGGGTTTAGGGTTTAGGGTTTAGGTTTAGGGTTTAGGGTTAGGGTTAGGGTTTAGGGTTAGGGTTTAGGGTTTAGGTTTAGGGTTTAGGGTTTAGGGTTTAGGGTTTAGGGTTTAGGGTTTAGGGTTTAGGGTTTAGGGTTTAGGGTTTAGGGTTAGGGTTTAGGGTTAGGGTTAGGGTTTAGGGTTTAGGGTTTAGGGTTTAGGGTTTAGGGTTTAGGTTTAGGGTTTAGGGTTTAGGGTTTAGGGTTTTAGGGTTTAGGGTTTAGGGTTTAGGGTTTAGGGTTTAGGGTTTAGGGTTAGGGTTAGGGTTTAGGGTTTAGGGTTTAGGTTAGGGTTTAGGGTTAGGGTTTAGGTTAGGGTTTAGGGTTAGGGTTTAGGGTTTAGGGTTTAGGGTTTAGGGTTTAGGGTTAGGGTTTAGGGTTAGGGTTTAGGGTTTAGGGTTTAGGGTTAGGGTTTAGGGTTTAGGGTTTAGGGTTTAGGGTTTAGGGTTTAGGGTTAGGGTTAGGGTTTAGGGTTTAGGGTTTAGGGTTTAGGGTTAGGGTTTAGGGTTTAGGGTTAGGGTTTAGGGTTTAGGGTTTAGGGTTAGGGTTTAGGGTTTAGGGTTTAGGGTTTAGGGTTTAGGGTTAGGGTTTAGGGTTTAGGGTTTAGGGTTTAGGGTTTAGGGTTTAGGGTTTAGGGTTTAGGGTTTAGGGTTTAGGGTTTAGGGTTAGGGTTTAGGGTTTAGGGTTTAGGGTTTAGGGGTTAGGGTTTAGGGTTTAGGGTTTAGGGTTTAGGGTTTAGGTTTAGGGTTTAGGGTTTAGGGTTTAGGGTTTAGGGTTTAGGGTTTAGGGTTTAGGTTTAGGGTTTAGGGTTAGGGTTTAGGGTTTAGGGTTTAGGGTTTAGGGTTTAGGGTTTAGGGTTTAGGGTTTAGGGTTTAGGGTTTAGGGTTTAGGTTGGGTTTAGGGTTTAGGGTTTAGGGTTTAGGGTTTAGGGTTTAGGGTTTAGGGTTTAGGGTTTAGGGTTTAGGGTTTAGGGTTTAGGGTTTAGGGTTTAGGGTTTAGGGTTTAGGGTTTAGGGTTTAGGGTTTAGGGTTTAGGGTTTAGGGTTTAGGGTTTAGGGTTTAGGGTTTAGGGTTTAGGGTTTAGGGTTTAGGGTTTAGGGTTTAGGGTTTAGGGTTTAGGGTTTAGGGTTTAGGGTTTAGGGTTTAGGGTTTAGGGTTTAGGGTTTAGGGTTTAGGGTTAGGGTTTAGGGTTTAGGGTTTAGGGTTTAGGGTTTAGGGTTTAGGGTTTAGGGTTTAGGGTTTAGGGTTTAGGGTTTAGGGTTTAGGGTTTAGGGTTTAGGGTTTAGGGTTAGGGTTTAGGGTTTAGGGTTTAGGGTTTAGGGTTTAGGGTTTAGGGTTTAGGGTTTAGGGTTTAGGGTTTAGGGTTTAGGGTTTAGGGTTTAGGGTTTAGGGTTTAGGGTTTAGGGTTTAGGGTTTAG

**7AL**

TTGG**TTTAGGG**TTTAGGGTTTAGGGTTTAGGGTTTAGGGTTAGGGTTTAGGGTTTAGGGTTAGGGTTTAGGGTTTAGGGTTTAGGGTTTAGGGTTTAGGGTTTAGGGTTTAGGGTTTAGGGTTAGGGTTTAGGGTTTAGGGTTTAGGGTTTAGGGTTTAGGGTTTAGGGTTTAGGGTTTAGGGTTTAGGGTTTAGGGTTTAGGGTTTAGGGTTTAGGGTTTAGGGTTTAGGGTTTAGGGTTTAGGGTTTAGGGTTTAGGGTTTAGGGTTTAGGGTTTAGGGTTTAGGGTTTAGGGTTTAGGGTTTAGGGTTTAGGGTTTAGGGTTTAGGGTTTAGGGTTTAGGGTTTAGGGTTTAGGGTTTAGGGTTTAGGGTTTAGGGTTTAGGGTTTAGGGTTTAGGGTTTAGGGTTTAGGGTTTAGGGTTTAGGGTTTAGGGTTTAGGGTTTAGGGTTTAGGGTTAGGGTTTAGGGTTTAGGGTTTAGGGTTTAGGGTTTAGGGTTTAGGGTTTAGGGTTTAGGGTTTAGGGTTTAGGGTTTAGGGTTTAGGGTTTAGGGTTTAGGGTTTAGGGTTTAGGGTTTAGGGTTTAGGGTTTAGGGTTTAGGGTTTAGGGTTTAGGGTTTAGGGTTTAGGGTTTAGGGTTTAGGGTTTAGGGTTTAGGGTTTAGGGTTTAGGGTTTAGGGTTTAGGGTTTAGGGTTTAGGGTTTAGGGTTTAGGGTTTAGGGTTTAGGGTTTAGGGTTTAGGGTTTAGGGTTTAGGGTTTAGGGTTTAGGGTTTAGGGTTTAGGGTTTAGGGTTTAGGGTTTAGGGTTTAGGGTTTAGGGTTTAGGGTTTAGGGTTTAGGGTTTAGGGTTTAGGGTTTAGGGTTTAGGGTTTAGGGTTTAGGGTTTAGGGTTTAGGGTTAGGGTTTAGGGTTAGGGTTTAGGGTTAGGGTTTAGGGTTTAGGGTTAGGGTTTAGGGTTTAGGGTTTAGGGTTTAGGGTTTAGGGTTTAGGGTTTAGGGTTTAGGGTTTAGGGTTTAGGGTTTAGGGTTTAGGGTTTAGGGTTTAGGGTTTAGGGTTTAGGGTTTAGGGTTTAGGGTTTAGGGTTTAGGGTTTAGGGTTTAGGGTTTAGGGTTTAGGGTTTAGGGTTTAGGGTTAGGGTTTAGGGTTTAGGGTTTAGGGTTAGGGTTTAGGGTTTAGGGTTTAGGGTTTAGGGGTAGGGTTTAGGGTTTAGGGTTTAGGGTTTAGGGTTAGGGTTTAGGGTTTAGGGTTTAGGGTTTAGGGTTAGGGTTTAGGGTTTAGGGTTTAGGGTTTAGGGTTAGGGTTTAGGGTTAGGGTTTAGGGTTTAGGGTTTAGGGTTTAGGGTTTAGGGTTTAGGGTTAGGGTTTAGGGTTTAGGGTTTAGGGTTTAGGGTTTAGGGTTTAGGGTTTAGGGTTTAGGGTTTAGGGTTTAGGGTTTAGGGTTAGGGTTAGGGTTTAGGGTTTAGGGTTTAGGGTTAGGGTTTAGGGTTTAGGGTTTAGGGTTTAGGGTTTAGGGTTTAGGGTTTAGGGTTTAGGGTTTAGGGTTTAGGGTTTAGGGTTTAGGTTTAGGGTTTAGGGTTTAGGGTTTAGGGTTTAGGGTTTAGGGTTAGGGTTTAGGGTTGGTTAGGGTTTAGGGTTTAGGGTTTAGGGTTTGTTTAGGGTTAGGGTTTAGGGTTTAGGGTTTAGGGTTTAGGGTTAGGGTTTAGGGTTTAGGGTTTAGGGTTTAGGGTTTAGGGTTTAGGGTTTAGGGTTTAGGGTTTAGGGTTTAGGGTTTAGGGTTTAGGGTTTAGGGTTTAGGGTTTAGGGTTTAGGGTTTAGGGTTTAGGGTTTAGGGTTTAGGGTTTAGGGTTAGGGTTTAGGGTTAGGGTTTAGGGTTAGGGTTAGGGTTTTAGGGTTTAGGGTTTAGGGTTTAGGGTTTAGGGTTTAGGGTTTAGGGTTTAGGGTTTAGGGTTTAGGGTTTAGGGTTTAGGGTTTAGGGTTTAGGGTTTAGGGTTTAGGGTTTAGGGTTTAGGGTTTAGGGTTAGGGTTTAGGGTTTAGGGTTTAGGGTTTAGGGTTTAGGGTTTAGGGTTTAGGGTTTAGGGTTTAGGGTTTAGGGTTAGGGTTTAGGGTTTAGGGTTTAGGGTTTAGGGTTTAGGTTTAGGGTTTAGGGTTTAGGGTTAGGGTTTAGGGTTTAGGGTTTAGGGTTTAGGGTTAGGGGTTTAGGGTTTAGGGTTTAGGGTTAGGGTTTAGGGTTTAGGGTTTAGGGTTTAGGTTTAGGGTTTAGGGTTAGGGTTTAGGGTTTGGGTTTAGGGTTTAGGGTTTAGGGTTAGGGTTTAGGGTTTAGGGTTTAGGGTTAGGGTTTAGGGTTAGGGTTAGGGTTTAGGGTTTAGGGTTTAGGTAGGGTTTAGGGTTTAGGGTTTAGGGTTTAGGGTTAGGGTTAGGGTTTAGGGTTTAGGGTTTAGGGTTTAGGGTTTAGGGTTTTGGGTTAGGGTTTAGGGTTTAGGGTTTAGGGGTTAGGGTTTAGGGTTTAGGGTTTAGGGTTTAGGGTTTAGGGTTTAGGGTTAGGGTAGGGTTTAGGGTTTAGGGTTTAGGGTTTAGGGTTTAGGGTTTAGGGTTTAGGGTTTAGGGTTTAGGGTTAGGGTTTAGGGGTTTAGGGTTAGGGTTAGGGTTTAGGGTTTAGGGTTTAGGGTTTAGGGTTTAGGGGTTTAGGGTTTAGGGTTTAGGGTTTAGGGTTTAGGGTTTAGGGTTGTAGGGTTTAGGGTTTAGGGTTTAGGGTTTAGGGTTTAGGGTTTAGGGTTTAGGGTTTAGGGTTTAGGGTTTAGGGTTAGGGTTTAGGGTTAGGGTTAGGGTTAGGGTTTAGGGGTTTAGGGTTTAGGGTTTAGGGTTAGGGTTTAGGGTTTAGGGTTTAGGGTTTAGGGTTTAGGGTTTAGGGTTAGGGTTTAGGGTTTAGGGTTTAGGGTTTAGGGTTAGGGTTTAGGGTTTAGGGTTTAGGGTTTAGGGTTAGGGTTTAGGGTTTTGGGTTTAGGGTTTAGGGTTTAGGGTTTAGGGTTAGGGTTAGGGTTAGGGTTTAGGGTTTAGGGTTTAGGGGGTTTAGGGTTTAGGGTTTAGGGTTTAGGGTTTAGGGGTTAGGGTTTAGGGTTTAGGGTTAGGGTTTAGGGTTTAGGGTTTAGGGTTTAGGGTTTAGGGTTAGGGTTAGGGTTTAGGGTTTAGGGTTTAGGGTTAGGGTTTAGGGTTTAGGGTTTGGGTTTAGGGTTTAGGGTTTAGGGTTTAGGGTGGTTTAGGGGTTTAGGGTTTAGGGTTTAGGGGTTTAGGGTTTAGGGTTTAGGGTTTAGGGTTTAGGGTTAGGGTTTAGGGTTAGGGTTAGGGTTTAGGGTTTAGGGTTTAGGGGGTTTAGGGTTTAGGGTTTAGGGTTTAGGGTTTAGGGTTTAGGGTTTAGGGTTTAGGGTTTAGGGTTTAGGGTTAGGGTTAGGGTTTAGGGGTTTAGGGTTTAGGGTTTAGGGTTTAGGGTTTAGGGTTTAGGGTTTAGGGTTTAGGGTTTAGGGTTTAGGGTTTAGGGTTTAGGGTTTAGGGTTTAGGGTTTAGGGGTTTAGGGTTTAGGGTGGTTAGGGTTTTAGGGTTTAGGGTTTAGGGTTTAGGGGTTAGGGTTGGGTTTAGGGTTTAGGGTTTAGGGTTTAGGGTTTAGGTTTAGGGTTTAGGGTTTAGGGTTTAGGGTTTAGGGTTTAGGGTTTAGGGTTTAGGGTGTTAGGGTTAGGGTTTAGGGTTTAGGTTTAGGGTTTAGGGTTTAGGGTTTAGGGTTTAGGGTTAGGGTTTTGGGTTTAGGGTTTAGGGTTTAGGGGTTAGGGTTTAGGGTTTAGGGTTTAGGGGTTTAGGGTTTAGGGGTTTAGGGTTTAGGGTTTAGGGTTTAGGGTTTAGGGTTTAGGTTTAGGGTTTAGGGTTTAGGGTTTAGGGTTTAGGGTTTAGGGTTAGGGTTTAGGGTTTAGGGTTTAGGGGTTTAGGGTTTAGGGTTAGGGTTGGGGGTTTAGGGTTTAGGGTTGGGGTTTAGGGTTTAGGGTTAGGGTTTAGGGTTGGGTTAGGGTTTAGGGTTTAGGGTTTAGGGTTTAGGGGTTTAGGGTTTAGGGTTTAGGGTTTAGGGGTTTAGGGTTGGGTTAGGGTTTAGGGTTTAGGGGTTTAGGGGTTAGGGTTTAGGGTTTAGGGGTTTAGGGTTTAGGGTTTAGGGTTAGGGTTTAGGGTTTAGGGTTTTAGGGTTAGGGTTAGGGGTTAGGGTTTAGGGTTTAGGGTTTAGGGGTTTAGGGTTTAGGGTTTAGGGTTTAGGGTTTAGGGTTTAGGGTTGGTTTAGGGTTAGGGGTTTAGGGTTTAGGGGGTTTAGGGTAGGGTTTAGGGTTAAGGGTTTAGGGTTTAGGGGTTTAGGGTTAGGGTTTAGGGTTTAGGGGTTTAGGGTTTTGGGTTTAGGTTTAGTGTTTAGGGTTTAGGTTTTAGGGTTTAGGGTTTAGGTTTTATGAGGTTTAAGGTTATAAGGTTTTAAGGGTTTTAGGTTTTAGGGGGGTTTAG

**1BS**

AGGG**TTTAGGG**TTTAGGGTTTAAGGGTTTAGGGTTAGGGTTAGGGTTAGGGTTTAGGGTTTAGGGTTTAGGGTTAGGGTTTAGGGTTTAGGGTTAGGGTTAGGGTTAGGGTTTAGGGTTTAGGGTTTAGGGTTAGGGTTTAGGGTTTAGGGTTTAGGGTTTAGGGTTTAGGGTTTAGGGTTTAGGGTTTAGGGTTTAGGGTTTAGGGTTTAGGGTTTAGGGTTTAGGGTTTAGGGTTTAGGGTTTAGGGTTTAGGGTTTAGGGTTTAGGGTTTAGGGTTTAGGGTTTAGGGTTTAGGGTTTAGGGTTAGGGTTTAGGGTTTAGGGTTTAGGGTTTAGGGTTTAGGGTTTAGGGTTTAGGGTTTAGGGTTTAGGGTTTAGGGTTAGGGTTTAGGGTTTAGGGTTTAGGGTTAGGGTTTAGGGTTTTGGGTTTTAGGGTTTTAGGGTTTTAGGGTTTTAGGGTTTGTTTAGGGTTTAGGGTTTAGGGTTTAGGGTTTAGGGTTAGGGTTTAGGGTTTAGGGTTTAGGGTTTAGGGTTTAGGGTTTAGGGTTTAGGGTTTAGGGTTTAGGGTTTAGGGTTAGGGTTTAGGGTTTAGGGTTTTAGGGTTTAGGGTTTAGGGTTTAGGGTTTAGGGTTTAGGGTTTAGGGTTTAGGGTTAGGGTTTAGGGTTTAGGGTTTAGGGTTTAGGGTTTAGGGTTTAGGGTTAGGGTTAGGGTTTAGGGTTAGGGTTTAGGGTTTAGGGTTTAGGGTTAGGGTTTAGGGTTTAGGGTTTAGGGTTTAGGGTTTAGGGTTTAGGGTTTAGGGTTTAGGGTTTAGGGTTTAGGGTTTAGGGTTTAGGGTTTAGGGTTTAGGGTTTAGGGTTTAGGGTTTAGGGTTTAGGGTTTTAGGGTTTAGGGTTTAGGGTTTAGGGTTTAGGGTTTAGGGTTTAGGGTTTAGGGTTTAGGGTTAGGGTTAGGGTTTAGGGTTTAGGGTTTAGGGTTTAGGGTTTAGGGTTTAGGGTTAGGTTTAGGGTTTAGGGTTTAGGGTTTAGGTTAGGGTTAGGGTTTAGGGTTTAGGGTTTAGGGTTTAGGGTTTAGGGTTTAGGGTTAGGGTTTAGGGTTTAGGGTTTAGGGTTTAGGGTTTAGGGTTTAGGGTTAGGGTTAGGGTTTAGGGTTTAGGGTTTAGGGTTTAGGTTAGGGTTTAGGGTTTAGGGTTAGGGTTTAGGGTTTAGGGTTTAGGGTTTAGGGTTTAGGGTTAGGGTTAGGGTTAGGGTTAGGGTTAGGGTTTAGGGTTTAGGGTTAGGGTTTAGGGTTTAGGGTTTAGGGTTAGGGTTAGGGTTTAGGGTTTAGGGTTTAGGGTTTAGGGTTTAGGGTTAGGGTTTAGGGTTTAGGGTTAGGGTTTAGGGTTTAGGGTTTAGGGTTTAGGGTTTAGGGTTTAGGGTTTAGGTTAGGGTTAGGGTTTAGGGTTAGGGTTAGGGTTTAGGGTTTAGGGTTAGGGTTAGGGTTTAGGGTTTAGGGGTTAGGGTTTAGGGTTTAGGGTTTAGGGTTTAGGGTTTAGGGTTTAGGGTTTAGGGTTTAGGGTTAGGGTTTAGGGTTTAGGGTTTAGGGTTAGGGTTTAGGGTTTAGGGTTTAGGGTTTAGGGTTAGGGTTTAGGGTTTAGGGTTTAGGGTTTAGGGTTTAGGGTTTAGGGTTTAGGGTTTAGGGTTTAGGGTTAGGGTTTAGGGTTTAGGGTTTAGGGTTTAGGGTTTAGGGTTAGGGTTTAGGGTTAGGGTTAGGGTTAGGGTTAGGGTTTAGGGTTTAGGGTTAGGGTTTAGGGTTTAGGGTTAGGGTTTAGGGTTTAGGGGTTTAGGGTTAGGGTTTAGGGTTTAGGGTTAGGGTTAGGGTTTAGGGTTTAGGGTTTAGGGTTGGTTTAGGGTTTAGGGTTTAGGGTTTAGGGTTTAGGGTTTAGGGTTTAGGGTTTGTTGGGTTTAGGGTTGGGTTAGGGTTTAGGGTTTAGGGTTAGGGGTTTAGGGTTTAGGGTTTAGGGTTAGGGTTTAGGGTTTAGGGTTTAGGGTTTAGGGTTAGGGTTTAGGGTTTAGGGGGTTTAGGGTTAGGGTTTAGGGTTTAGGGTTTAGGGTTTAGGGTTTAGGGTTAGGGTTTAGGGTTTAGGGTTTAGGGTTTGGGGTTGGGTTGTAGGGTTTAGGGTTAGGGTTTAGGGTTTAGGGTTAGGGTTTTGGGTTTAGGGTTTAGGGTTTAGGGTTTAGGGTTTAGGGTTTAGGGTTTAGGGTTTAGGGTTTAGGGTTTAGGGTTTAGGGTTTAGGGTTTAGGGTTTAGGGTTTAGGGTTTAGGGTTTAGGGTTAGGGTTTAGGGTTTGGTTAGGGTTAGGTTAGGGTTTAGGGTTAGGGTTTAGGGTTTAGGGTTTAGGGTTTAGGGTTAGGGTTTGGGTTAGGGTTTAGGGTTTAGGGTTTAGGGTTTAGGGTTTAGGGTTTAGGGTTTAGGGTTTAGGGTTTAGGGTTTTGGGTTAGGGTTTAGGGTTTAGGGTTTAGGGTTTAGGGTTTAGGGTTTAGGGTTAGGGTTTAGGGTTTAGGGTTAGGGTTTAGGGTTTAGGGTTAGGGTTTAGGGTGGGTTAGGGTTTAGGGTTTAGGGTTAGGGTTTAGGGTTAGGGTTTAGGGTTTAGGGTTTAGGGTTTAGGGTTTAGGGTTTAGGGTTTAGGGTTTAGGGTTTAGGGTTGTAGGGGGTGGTTTAGGGGTTAGGTTAGGGTTTAGGGTTTAGGGTTAGGGTTTAGGGTTTAGGGTTTAGGGTTTAGGGTAGGGTTTAGGGGTTTAGGGTTTAGGGTTAGGGTTTAGGGTTTAGGGTTAGGGGTGAGGGTTTAGGGTTTAGGGTTTGGGTTAGGGTTTAGGGTGAGGGGTTTAGGGTTAGGGTTTAGGGTTAGGGTTGGTTTAGGGTTTAGGGTTTGGGTTAGGTTTGAGGGTTGGTTGGGTTTAGGGTTAGGGTTAGGGTTTAGGGTTTAGGGTTTAGGGGTTAGGGTTTAGGGTTTAGGGTTTAGGGTTTAGGGTTTAGGGTTTTGGGGTAGGTTGGGGGTAGGGTTAGGGGTTAGGGTTTAGGGGTAGGGTGTAGGGTTTTGGGTAGGGGTGAGGGTTTAGGGTGTTGGGTGTTGGGTTGTGGGTAGGTTGGGGGTAGGGTTTGGGGTGGGTTAGGGTTAGGTGGTGGGTGGGGTAGGGTTAGGGTTTAGGGTTTAGGTTAGGGGTAGGGTTAGGGGTTTAGGTGTGGGGTTGGGGTAGGGTTTAGGGTGTTAGGGTGTAGGGTGGTTGGGTTTAGGGTTGTAGGGTGTTAGGGTGTTAGGGGGTAGGGTTTTGGGGTGGGTGAGGGGTGGGGGTAGGGTGTAGGGTTGAGGGTTGTAGGGTTAGGGTTTGGGTGTGGGGTAGGTGGGTGGTGTGAGGGTTGGGTTAGGGTTTGGGGTGTGGGGGTTGGGGGTGGTTTGGTTTGGGTGGGTGTGGGGGTAGGGTTTGGGGTAGGGTGTGGGTGTGGGGTTAGGGTGTGGGGGTGGGTGTGGGGTGTGGGGTTTGGGGGGTGGGGTGAGGGTGAGGGGTTGGGTGAGGGGTGGGTGTGGGTGGTGGGTGGTAGGGGGGGGTGGGGGTTGGGGTTAGGGTTTAGGGTTTAGGGTTTAGGGTTTAGGGTTTAGGGTTTAGGGTTTAGGGTTTAGGGTTTAGGGTTTAGGGTTTAGGGTTTAGGTTTAGGGTTTAGGGTTAGGGTTTAGGGTTTAGGGTTTAGGGTTTAGGGTTTAGGGTTTAGGGTTTAGGGTTTAGGGTTAGGGTTTAGGGTTTAGGGTTTAGGTTTAGGGTTTAGGGTTTAGGGTTTAGGGTTTAGGGTTTAGGGTTTAGGGTTTAGGGTTTAGGGTTAGGGTTTAGGGTTAGGGTTTAGGGTTAGGTTAGGGTTTAGGGTTAGGGTTTAGGGTTTAGGGTTTAGGGTTTAGGGTTTAGGGTTTAGGGTTTAGGGTTTAGGGTTAGGGTTTAGGGTTTAGGGTTTAGGGTTTAGGGTTTAGGGTTAGGGTTTAGGGTTTAGGGTTTAGGGTTGGTTTAGGGTTTAGGGTTTAGGGTTTAGGGTTTAGGGTTTAGGGTTAGGGTTTAGGGTTTAGGGTTTAGGGTTTAGGGTTTAGGGTTTAGGGTTAGGGTTTAGGGTTTAGGGTTTAGGGTTAGGGTTTAGGGTTTAGGGTTTAGGGTTTAGGGTTAGGGTTTAGGGGTTAGGGTTTAGGGTTTAGGGTTTAGGGTTTAGGGTTTAGGGTTTAGGGTTTAGGGTTTAGGGTTTAGGGTTTAGGGTTTAGGGTTTAGGGTTTAGGGTTTAGGGTTTAGGGTTTAGGGTTTAGGGTTTAGGGTTTAGGGTTTAGGGTTTAGGGTTAGGGTTTAGGGTTTAGGGTTTAGGGTTTAGGGTTTAGGGTTTAGGGTTTAGGGTTTAGGGTTTAGGGTTAGGGTTTAGGGTTTAGGGTTAGGGTTTAGGGTTTAGGGTTTAGGGTTTAGGGTTTAGGGTTTAGGGTTTAGGGTTTAGGGTTTAGGGTTTAGGGTTTAGGGTTTAGGGTTTAGGGTTAGGGTTTAGGGTTTAGGGTTTAGGGTTTAGGGTTAGGGTTTAGGGTTTAGGGTTTAGGGTTTAGGGTTAGGGTTTAGGGTTTAGGGTTTAGGTAGGGTTTAGGGTTTAGGGTTTAGGGTTTAGGGTTTAGGGGTTTAGGGTTAGGGTTTAGGGTTTAGGGTTTAGGGTTTAGGGTTTAGGGTTTAGGGTTTAGGGTTTAGGGTTTAGGGTTTAGGGTTTAGGGTTTAGGGTTTAGGGTTTAGGGTTAGGGTTTAGGGTTTAGGGTTTAGGGTTTAGGGTTTAGGGTTAGGGTTTAGGGTTTAGGGTTTAGGGTTTAGGGGTTTAGGGTTTAGGGTTAGGGTTTAGGGTTTAGGGTTTAGGGTTTAGGGTTTAGGGTTTAGGGTTTAGGGTTTAGGGTTTAGGGTTTAGGGTTTAGGGTTTAGGGTTTAGGGTTTAGGGTTTAGGGTTTAGGGTTTAGGGTTTAGGGTTTAGGGTTTAGGGTTTAGGGTTTAGGGTTTAGGGTTTAGGGTTTAGGGTTTAGGGTTTAGGGTTAGGGTTTAGGGTTTAGGGTTTAGGGTTTAGGGTTTAGGGTTTAGGGTTTAGGGTTTAGGGTTTAGGGTTTAGGGTTTAGGGTTTAGGGTTTAGGGTTTAGGGTTTAGGGTTTAGGGTTTAGGGTTTAGGGTTTAGGGTTTAGGGTTTAGGGTTTAGGGTTTAGGGTTTAGGGTTAGGTTAGGGTTTAGGGTTTAGGGTTTAGGGTTTAGGGTTTAGGGTTTAGGGTTTAGGGTTAGGGTTTAGGGTTTAGGGTTTAGGGTTTAGGGTTAGGGTTTAGGGTTTAGGGTTTAGGGTTTAGGGTTTAGGGTTTAGGGTTTAGGGTTTAGGGTTTAGGGTTTAGGGTTTAGGGTTAGGGTTTAGGGTTTAGGGTTTAGGGTTTAGGGTTTAGGGTTTAGGGTTAGGGTTAGGGTTTAGGGTTTAGGGTTTAGGGTTTAGGGTTTAGGGTTTAGGTTTAGGGTTTAGGGTTTAGGGTTTAGGGTTTAGGGTTTAGGGTTGGGTTAGGGTTTAGGGTTTAGGGTTTAGGGTTAGGGTTTAGGGTTTAGGGTTAGGGTTTAGGGTTTAGGGTTTAGGGTTTAGGGTTTAGGGTTTAGGGTTAGGGTTTAGGGTTTAGGGTTTAGGGTTTAGGGTTTAGGGTTTAGGGTTTAGGGTTTAGGGTTTAGGGTTAGGGTTTAGGGTTTAGGGTTTAGGGTTTAGGGTTTAGGGTTTAGGGTTTAGGGTTTAGGGTTTAGGGTTTAGGGTTTAGGGTTAGGGTTTAGGGTTTAGGGTTAGGGTTTAGGGTTTAGGGTTTAGGTTTAGGGTTTAGGGTTTAGGGTTAGGGTTTAGGGTTTAGGGTTTAGGGTTTAGGGTTTAGGGTTTAGGGTTTAGGGTTTAGGGTTTAGGGTTTAAGGGTTTAGGGTTTAGGGTTTAGGGTTTAGGGTTTAGGGTTTAGGGTTTAGGGTTTAGGGTTTAGGGTTTAGGGTTTAGGGTTTAGGTTTAGGGTTTAGGGTTTAGGGTTTAGGGTTTAGGGTTTAGGGGTTTAGGGTTTAGGGTTTAGGGTTTAGGGTTTAGGGTTTAGGGTTTAGGTTTAGGGTTTAGGGTTTAGGGTTTAGGGTTTAGGGTTTAGGGTTTAGGGTTTAGGGTTTAGGGTTAGGTTTAGGGTTTAGGGTTTAGGGTTTAGGTTTAGGGTTTAGGGTTTAGGGTTTAGGGTTAGGGTTTAGGGTTTAGGGTTTAGGGTTAGGGTTAGGGTTTAGGGTTTAGGGTTTAGGGTTAGGGTTTAGGGTTTAGGGTTTAGGGTTTAGGGTTTAGGGTTTAGGGTTTAGGGTTTAGGGTTTAGGGTTTAGGGGTTTAGGGTTTAGGGTTTAGGGTTTAGGTTTAGGGTTTAGGGTTTAGGGTTTAGGGTTTAGGGTTTAGGGTTTTAGGGTTTAGGGTTTAGGTTTAGGGTTTAGGGTTTAGGGTTTAGGGTTTAGGGTTTAGGGTTAGGGTTTAGGGTTTAGGGTTTAGGGTTTAGGGTTTAGGGTTTAGGGTTTAGGGTTTAGGGTTTAGGGTTTAGGGTTTAGGGTTAGGGTTTAGGGTTTAGGGTTTAGGGTTAGGGTTTAGGGTTTAGGGTTTAGGGTTTAGGGTTAGGGTTTAGGGTTTAGGGTTTAGGGTTTAGGGTTTAGGGTTTGGTTTAGGGTTAGGGTTTAGGGTTTAGGGTTTAGGGTTTAGGGTTTAGGGTTTAGGGTTTAGGGTTTAGGGTTTAGGGTTTAGGGTTTAGGGTTAGGGTTTAGGGTTTAGGGTTTAGGGTTTAGGTTTAGGGTTTAGGGTTTAGGGTTTAGGGTTTAGGGTTTAGGGTTTAGGGTTTAGGGTTTAGGGTTTAGGGTTAGGGTTTAGGGTTTAGGGTTTAGGGTTTAGGGTTTAGGGTTTAGGGTTTAGGGTTTAGGGTTTAGGGTTTAGGGTTTAGGGTTTAGGGTTTAGGGTTTAGGGTAGGTTTAGGGTTTAGGGTTTAGGGTTTAGGGTTTAGGGTTTAGGGTTTAGGGTTTAGGGTTTAGGGTTTAGGGTTTAGGGTTTAGGGTTTAGGGTTTAGGGTTTAGGGTTTAGGGTTTAGGGTTTAGGGGTTTAGGGTTTAGGGTTTAGGGTTTAGGGTTTAGGGTTTAGGGTTTAGGGTTTAGGGTTTAGGGTTTAGGGTTTAGGGTTTAGGGTTTAGGGTTTAGGGTTTAGGGTTTAGGGTTTAGGGTTTAGGGTTTAGGGTTTAGGGTTTAGGGTTTAGGGTTAGGGTTTAGGGTTTAGGGTTTAGGGTTTAGGGTTAGGGTTTAGGGTTTAGGGTTTAGGGTTTAGGGTTTAGGGTTTAGGGTTTAGGGTTTAGGGTTTAGGGTTTAGGGTTTAGGGTTAGGGTTTAGGGTTTAGGGTTTAGGGTTTAGGGTTTAGGGTTTAGGGTTTAGGGTTTAGGTTTAGGGTTTAGGGTTTAGGGTTTAGGGTTTAGGGTTTAGGGTTTAGGGGTTAGGGTTTAGGGTTTAGGGTTTAGGGTTTAGGGTTTAGGGTTTAGGGTTAGGGTTTAGGGTTTAGGGTTTAGGGTTTAGGGTTAGGGTTTAGGGTTTAGGGTTTAGGGTTTAGGGTTTAGGGTTTAGGGTTTAGGGTTTAGGGTTTAGGGTTTAGGGTTAGGGTTTAGGGTTTAGGGTTTAGGGTTTAGGGTTTAGGGTTTAGGGTTTAGGGTTAGGTTTAGGGTTTAGGGTTTAGGGTTTAGGGTTTAGGGTTTAGGGTTAGGGTTTAGGGTTTAGGGTTTAGGGTTTAGGGTTTAGGGTTTAGGGTTAGGGTTTAGGGTTTAGGGTTTAGGGTTTAGGGTTTAGGGTTTAGGGTTTAGGGTTTAGGGTTTAGGGTTAGGGTTTAGGGTTTAGGGTTTAGGGTTTAGGGTTTAGGGTTAGGGTTTAGGGTTTAGGGTTTAGGGTTAGGGTTTAGGGTTTAGGGTTTAGGGTTTAGGGTTTAGGGTTTAGGGTTTAGGGTTTAGGGTTTAGGGTTTAGGGTTTAGGGTTAGGGTTTAGGGTTTAGGGTTAGGGTTTAGGGTTTAGGGTTTAGGGTTTAGGGTTTAGGGTTTAGGGTTTAGGGTTTAGGGTTAGGGTTTAGGGTTTAGGGTTTAGGTTTAGGGTTAGGGTTTAGGGTTTAGGGTTTAGGGTTTAGGGTTTAGGGTTTAGGGTTTAGGGTTTAGGGTTTAGGGTTTAGGGTTTAGGGTTGTTAGGGTTTAGGGTTTAGGGTTAGGGTTTAGGGTTTAGGGTTTAGGGTTTAGGGTTAGGGTTTAGGGTTTAGGGTTTAGGGTTTAGGGTTTAGGGTTTAGGGTTTAGGGTTTAGGGTTAGGGTTTAGGGTTTAGGGTTTAGGGTTTAGGGTTTAGGGGTTTAGGGTTTAGGGTTAGGGTTAGGGTTTAGGGTTAGGGTTAGGGTTTAGGGTTTAGGGTTAGGGTTTAGGGTTTAGGGTTTAGGGTTTAGGGTTTAGGGTTTAGGGTTTAGGGTTTAGGGTTTAGGGTTTAGGGTTTAGGGTTTAGGGTTTAGGGTTTAGGGTTTAGGGTTTAGGGTTTAGGGTTTAGGGTTTAGGGTTTAGGTTTAGGGTTTTAGGGTTTAGGGTTAGGGTTTAGGGTTTAGGGTTTAGGGTTTAGGGTTTAGGGTTTAGGGTTTAGGGTTTAGGGTTTAGGGTTTAGGGTTTAGGGTTAGGGTTTAGGGTTTAGGGTTTAGGGTTTAGGGTTTAGGGTTTAGGGTTTAGGGTTTAGGGTTTAGGGTTTAGGGTTTAGGGTTTAGGGTTTAGGGTTTAGGGTTTAGGGTTTAGGGTTTAGGTTTAGGGTTTAGGGTTTAGGGTTTAGGGTTTAGGGTTTAGGGTTTAGGGTTTAGGGTTTAGGGTTTAGGGTTTAGGGTTTAGGGTTAGGGTTTAGGGTTTAGGGGTTTAGGGTTTAGGGTTTAGGGTTTAGGGTTTAGGGTTTAGGGTTAGGGTTTAGGTTTAGGGTTTAGGGTTAGGGTTAGGGTTTAGGGTTTAGGGTTTAGGGTTTAGGGTTTAGGGTTTAGGGTTTAGGGTTTAGGGTTAGGGTTTAGGGTTTAGGGTTTAGGGTTTAGGGTTAGGGTTTAGGGGTTAGGGTTTAGGGTTAGGGTTTAGGGTTTAGGGTTTAGGGTTTAGGGTTAGGGTTTAGGGTTTAGGGTTTAGGTTTAGGGTTTAGGGTTTAGGGTTTAGGGTTTAGGGTTTAGGGTTTAGGGTTTAGGGTTTAGGGTTTAGGGTTTAGGGTTTAGGGTTAGGGTTTTAGGGTTTAGGGTTTAGGGTTTAGGGTTTAGGGTTTAGGGTTAGGGTTTAGGGTTTAGGGTTTAGGGTTTAGGGTTAGGGTTTAGGGTTTAGGGTTTAGGGTTTAGGGTTTAGGGTTTAGGGTTTAGGGTTTAGGGTTAGGGTTTAGGGTTTAGGGTTTAGGGTTTAGGGTTAGGGTTTAGGGTTTAGGTTAG

**2BS**

TG**TTTAGGG**TTTAGGGTTGAGGGTTTAGGGTTTAGGGTTTAGGGTTTTTAGGGTTTAGGGTTTAGGGTTTCGGTTTAGGGTTTTAGGGTTTAGGGTTTAGGGTTAGGGTTTAGGGTTTAGGGTTTAGGGTTTAGGGTTTAGGGTTTAGGGTTTAGGTTTAGGGTTAGGGTTTAGGGTTTAGGGTTTAGGGTTTTAGGGTTTAGGGTTTAGGGTTTAGGTTTAGGGTTTAGGGTTTAGGGTTTAGGGTGTTAGGGTTTTAGGGTTTAGGGTTTAGGGTTAGGGTTTAGGGTTTAGGGTTTAGGGTTTAGGGTTTAGGGTTTAGGGTTTAGGGTTTAGGGTTTAGGGTTAGGGTTTAGGGTTTAGGGTTTAGGGTTTTGGGTTAGGGTTTAGGGTTTAGGGTTTAGGGTTTAGGTTTAGGGTTTAGGGTTAGGGTTTAGGGTTTAGGGTTTAGGGTTTAGGGTTTAGGGTTAGGTTTAGGGTTTAGGGTTTAGGGTTTAGGGTTTAGGGTTTAAGGGTTTAGGGTTTAGGGTTTAGGGTTTAGGGTTTAGGGTTTAGGGTTTAGGGTTAGGGTTTAGGGTTTAGGGTTTAGGGTTTAGGGTTAGGTTAGGGTTTAGGGTTTAGGGTTTAGGTTTAGGGTTTAGGGTTTAGGGTTTAGGGTTTAGGGTTTAGGTTTAGGGTTTAGGGTTTAGGGTTTAGGGTTTAGGTGTTTAGGGTTTAGGGTTAGGGTTTAGGGTTTAGGGTTTAGGGTTTAGGGTTTAGGGTTAGGGTTTAGGGTTTAGGGTTTAGGGTTTAGGGTTTAGGGTTAGGTTAGGGTTTAGGGTTTAGGGTTAGGGTTTAGGGTTTAGGGTTAGGGTTTAGGGGTTTAGGGTTAGGGTTAGGGTTTAGGTTTAGGGTTAGGGTTAGGGTTAGGGTTTTAGGGTTTAGGGTTAGGTTAGGGTTTAGGGTTTAGGGTTAGGGTTTAGGTTAGGGTTAGGGTTTAGGGTTTAGGTTTAGGGTTAGGGTTAGGGTTTAGGGTTAGGGTTTAGGGTTTAGGGTTAGGGTTTAGGGTTAGGGTTTAGGGTTTAGGGTTAGGGTTTAGGGTTTAGGGTTTAGGGTTTAGGGTTAGGGTTTAGGGTTAGGGTTTAGGGTTAGGGTTTAGGGTTTAGGTTAGGTTTAGGGTTTAGGGTTTAGGTTAGGGTTAGGGTTTAGGGGTTTAGGGTTTAGGGTTTAGGGTTTAGGGTTTAGGGTTAGGTTAGGGTTTAGGGTTTAGGGTTTAGGGTTTAGGGTTTGGTTAGGGTTAGGGTTTAGGGTTTAGGGTTAGGGTTTAGGGTTTAGGGTTTAGGGTTTAGGGTTTAGGGTTTAGGGTTTAGGGTTTAGGGTTTAGGTTTAGGGTTAGGGGTTTAGGGTTTAGGGTTTAGGGGTGTTAGGGTTTAGGGTTTAGGGTTTAGGGTTTAGGGTTTAGGGTTTAGGGTTTAGGGTTTAGGGTTTAGGGTTTAGGGGTTTAGGGTTTAGGGTTAGGGTTTAGGTTTAGGGTTTAGGTTAGGGTTTAGGGTTTAGGGTTAGGGTTTAGGGTTTAGGTTAGGGTTTAGGGTTTAGGGTTAGGTTAGGGTTTAGGGTTTTAGGGTTTAGGGTTAGGGTTTTAGGGTTTAGGGTTTTAGGGTTAGGGGTTAGGGTTTAGGGTTTAGGGTTTAGGGTTTAGGGTTTAGGGTTTAGGGTTAGGGTTTAGGGTTAGGGTTTAGGGTTTAGGGTTTAGGGTTAGGGGTTTAGGGTTAGGGTTTAGGGTTTAGGGTTTAGGGTTTAGGGTTTAGGGTTTAGGGTTTAGGGTTTAGGTTTAGGGTTAGGGTTTAGGGTTAGGGTTAGGGTTAGGTTTAGGGTTAGGGTTTAGGGTTTAGGGTTTAGGGTTAGGGTTGGGTTAGGGTTTAGGGTTTAGGGTTTAGGTTTAGGGTTTAGGGTTAGGGTTTAGGGTTTAGGGTTTAGGGGTTTAGGGTTAGGGGTTTAGGTTTAGGGTTAGGGTTTAGGGTTTGGTTAGGGTTTAGGGGTTAGGTTTAGGGTTTAGGGGGTTTAGGGGTTAGGGTTTAGGGTTTAGGGTTTAGGGTTTAGGGTTTAGGGTTTAGGGTTGGTTAGGGTTTAGGGTTAGGGTTTAGGGTTTAGGGTTTAGGTTTAGGGTTTAGGGTTTAGGGTTAGGGTTAGGTGGGTTAGGGTTTAGGGTTTAGGGTTTAGGGTTTAGGGTTTAGGGTTAGGGTTTAGGGTTTAGGGTTAGGGTTTAGGGTTTAGGGTTTAGGGTTAGGTTAGGGTTTAGGGTTTAGGGTTTAGGGTTTAGGGTTTAGGGTTTAGGGTTAGGGTTTAGGGTTTAGGGTTTAGGGTTAGGGTTAGGGTTTAGGGTTTAGGTTTAGGGTTTAGGGTTTAGGGTTTAGGGTTTAGGGTTTAGGGTTTAGGTTTAGGGTTTAGGGTTTAGGGTTTAGGGTTTAGGTTAGGGTTTAGGGTTTAGGGTTTAGGGTTTAGGGTTTAGGGTTAGGGTTGGTTAGGGTTTAGGGTTAGGGTTAGGTTGGGTTTAGGGTTTAGGGTTTAGGGTTTAGGGTTTAGGGTTAGGGTTTAGGGGTTTAGGGTTAGGTTAGGGTTTAGGGTTAGGGTTTTAGGGTTTAGGGTTTAGGGGTTTAGGGTTTAGGGTTTAGGGTTTAGGGTTTAGGGTTTAGGGTTTAGGGTTTAGGGTTTAGGGTTTAGGGTTTAGGGTTTAGGTTTAGGGTAGGGTTTAGGGTTAGGTTAGGTTTAGGGTTTAGGGTTTAGGGTTAGGGTTTAGGGTTAGGGTTTAGGGTTTAGGGTTTAGGGTTTAGGGTTTAGGGGTTAGGGTGTTAGGGTTTAGGGTTTAGGGTTTAGGGTTTAGGGTTTAGGGTTAGGGGGTTAGGGGTTTAGGGTTTAGGGGTTTAGGGTTAGGGTTTAGGGTTTAGGGTTTAGGGTTTAGGTTTAGGGTTAGGGTTTAGGGTAGGGTTTAGGGTTTAGGGTTTAGGGGTTTAGGGTTTAGGGTTAGGGTTAGGGTTTAGGGTTTAGGGTTTAGGGTTTAGGGTTAGGGTTTAGGGGTTTAGGGTTTAGGGTTTAGGGTTTAGGGTTTTAGGGTTTAGGGTTTAGGGGTTAGGGTTTAGGGTTTAGGGTTAGGGTTTAGGGTTTAGGGTTAGGGTTTAGGGTTTAGGGTTTAGGTTTAGGGGTTTAGGGTTGTAGGGTTTTAGGGTTAGGGGTTTAGGGTTTAGGGTTTAGGGTTAGGGTTAGGTTTAGGTTAGGGTTTAGGGTTTAGGTTTAGGGTTAGGGTTTTAGGGGTTAGGGTTTAGGGTTTAGGTTTAGGGTTTAGGGTTTAGGTTAGGGTTAGGTTTAGGGTTTAGGGTTTAGGGTTGGTTAGGGTTTAGGGTTTAGGGTTTAGGGGTTAGGTTAGGGTTTAGGGTTTAGGGTTTAGGGTTTAGGGTTTAGGGTTAGGGTTTAGGGTTAGGTTTAGGGTTTAGGGTTTAGGGTTTAGGGTTTAGGGTTAGGGTTTAGGGTTTAGGGTTTAGGGTTAGGGTTTAGGGTTTAGGGTTTAGGGTTGGTTAGGGGTTTAGGGTTTAGGGGTTAGGGTTAGGGTTTAGGGTTTAGGGTTTAGGGTTTAGGGTTTAGGGTTTAGGGTTTAGGGTTAGGGTTAGGGTTTAGGGTTTAGGGTTTGGTAGGGTTTAGGGTTTTAGGGTTTAGGGGTTTAGGGTTTAGGGTTTAGGGTTTTAGGTTTAGGGTTTAGGGGTTTAGGGTTTAGGGTTTAGGGTTAGGGTTTAGGGTTTAGGGTTTAGGGTTTAGGGTTTAGGGTTTAGGGTTTAGGGTTAGGGTTTAGGGGTTTAGGGTTAGGGTTTAGGGTTTAGGGTTTAGGGTTTAGGGTTTAGGTTTAGGGTTTAGGGTTTAGGGTTTAGGGTTTAGGGTTAGGGTTTGAGGGTTTAGGGTTTAGGGTTTAGGGTTTAGGGTGTTAGGGTTAGGGTTTAGGGTTAGGGTTAGGGTTAGGGTTTAGGGTTTAGGGTTTAGGGTTAGGGTTTAGGGTTTAGGGGGGTTTAGGGTTTAGGGTTTAGGGTTAGGGTTTAGGGTTTAGGGTTAGGGTTTAGGTTTAGGGTTTAGGGTTTAGGGTTTAGGGTTTAGGGTTTAGGGTTTAGGGTTTAGGGTTAGGTTAGGGTTTAGGGTTTAGGGTTAGGGTTTAGGGTTTAGGGTTTAGGGTTAGGGTTAGGGTTTAGGGTTTAGGGTTTTGGGTTTAGGGTTAGGGGTAGTTAGGGTTTAGGTTAGGTTTAGGGTTTAGGGTTTAGGGTTTAGGGTTTAGGGTTAGGGTTAGGTTTAGGGTTTAGGGTTTAGGGTTTAGGGTTTAGGGTTAGGGTTAGGGTTAGGGGTTAGGGTTTAGGGTTAGGGTTTAGGGTTTTAGGGTTTTTAGGGTTTGGGTTTAGGGTTTAGGGTTTAGGGTTTAGGGTTTAGGGTTAGGGTTTAGGGTTTAGGGTTTAGGGTTAGGGTTTAGGGTTTAGGTTAGGGTTTAGGGTTTGGGTTTAGGTTTTAGGGTTTAGGGTTTAGGGTTTAGGGTTTAGGGTTTAGGGTTTAGGGTTTAGGGGTTAGGTTAGGGTTTAGGGTTTAGGGTTTAGGGTTTAGGGTTTAGGTTTAGGGTTAGGGTTTAGGGTTTTAGGGTTTAGGGTTTAGGGTTTAGGGTTTAGGGTTTAGGTGTTTAGGGTTTAGGGTTAGGGTTTAGGGTTTAGGGTTTAGGGTTTAGGGTTTAGGGTTTAGGGTTTAGGGTTTAGGGTTTTAGGGTTTAGGGTTTAGGGTTTAGGGTTTAGGGTTTAGGGTTTAGGGTTTAGGGTTATGGGTTTAGGGTTTAGGGTGTTAGGGTTTAGGGTTTAGGGTTTAGGGTTAGGGTTAGGGTTTTTAGGGTTTAGGGTTTAGGGTTTAGGGTTTAGGTTTAGGTTTAGGGTTTAGGGTTTAGGGTTTAGGGTTTAGGTGGTTTAGGGTTTAGGGTTAGGGTTTAGGGTTTAGGGTTTAGGGTTTTAGGGTTTAGGGTTTAGGGTTTAGGGTTTAGGGTTTAGGGTTAGGGTTTAGGGTTTAGGGTTTAGAGGTTTAGGTTAGGGTTAGGGTTAGGGTTAGGTTAGGGTTTAGGGTTTAGGGTTTAGGGTTTAGGGTTTAGGGTTAGGGTTTAGGGTTAGGGTTTAGGGTTTAGGGTTAGGGTAGGGTTTAGGGTTTAGGGTTTAGGGTTTAGGGTTTAGGGTTTAGGGTTTAGGGTTTAGGGTTTAGGGTTTAGGGTTAGGGTTTAGGGTTTAGGGTTTAGGTTTAGGGTTAGGTTAGGGTTTAGGGTTTAGGGTTTAGGGTTTAGGGTTTAGGGTTTAGGGTTAGGGTTAGGGTTTAGGGTTTAGGGTTTAGGGTTTAGGGTTAGGGTTTAGGGTTTAGGGTTTAGGGTTTAGGGGTTTAGGGTTTAGGGTTTAGGGTTAGGGTTTAGGGTTTAGGGTGTTAGGGTTTAGGGTTAGGGTTTAGGGTTTAGGGTTAGGGTTTAGGGTTTAGGGTTTAGGGTTTAGGGTTTAGGGGTTTAGGGTTAGGGTTTTAGGGTTTAGGGTTTAGGGGTTTAGGGTTTAGGGTTTAGGGTTAGGGTTTTAGGGTTAGGGTTTAGGGTTTAGGGTTTAGGGTTAGGGTTTAGGGGTAGGGTTTAGGGTTTAGGGTTTAGGGTTAGGGTTAGGGTTAGGGTTTAGGGTTTAGGGTTAGGGTTTAGGGTTTAGGGTTAGGTTTAGGGTTTAGGGTTTAGGGTTAGGGTTTAGGGTTAGGGTTAGGGTTTAGGGTTAGGTTAGGGTTTAGGGTTTAGGGTTTAGGTTAGGGTTTAGGGTTTAGGGTTTTAGGTTAGGGTTTAGGGTTTAGGGTTTAGGGTTTAGGGTTAGGTAGGTTTAGTGGTTAGGGTTTAGGGTTAGGGTTTAGGGTTTAGGGTTTAGGGTTTTGGTTAGGGTTAGGGTTAGGGTTTAGGGTTTTAGGGTTTAGGTTTAGGGTTTAGGGTTTAGGGTTTAGGGTTTAGGGTTTAGGGTTAGGGTTTAGGTGGGTTAGGGTTTTAGGGTTTAGGGTAGGGTTTAGGGTTAGGGTTTAGGGGTTTAGGGTTTAGGGTAGGGTTTAGGGTTTAGGGTTTGGTTAGGGTTTAGGGTTAGGGTTTAGGGTTAGGGTGTTAGGGTTAGGGTTTAGGGTTTAGGGTGTAGGGTTTTGGGGTTTAGGGGTTTAGGGTTTGGGTTTAGGGTTTAGGGTTTAGGTTTAGGGTTAGGTTAGGGTTAGGGTTTAGGGTTTAGGGTTTAGGGTTTAGGGTTTTGGGTTAGGGTTTAGGGTTTAGGGTTTAGGGTTTAGGGGTTTAGGGTTGGTTTAGGGTTTAGGGTTTGGGTTTAGGGTTTAGGGTTTAGGGTTAGGGTTTAGGGTTAAGGGTTA

**3BL**

TGGG**TTTAGGG**TTTAGGGTTTAGGGTTTAGGGTTTAGGGTTTTAGGGTTTTGGGTTTAGGGTTTGAGGGTTTAGGGTTAGGGTTTAGGGTTTACAGGGTTTAGGGTTTAGGGTTTAGGGTTTAGGGTTTAGGGTTTAGGGTTTAGGGTTTAGGGTTTAGGGTTTAGGGTTTAGGTTTTAGGGTTTAGGGTTTAGGGTTTAGGGTTTAGGGTTAGGGTTTAGGGTTAGGGTTTAGGGTTTAGGGTTTAGGGTTTAGGGTTTAGGGTTAGGGTTTAGGGTTTAGGGTTTAGGGTTTAGGGTTTAGGGTTTAGGGTTTAGGGTTTAGGGTTAGGTTTAGGGTTTAGGGTTTAGGGTTAGGGTTTAGGGTTTAGGGTTTAGGGTTTAGGGTTTAGGGTTTAGGGTTTAGGGTTTAGGGTTTAGGGTTTAGGGTTTAGGGTTTAGGGTTTTTAGGGTTTAGGGTTTAGGGTTTAGGGTTTAGGGTTTAGGGTTTAGGGTTTGGGTTTAGGGTTTAGGGTTTAGGGTTTAGGGTTTAGGGTTAGGGTTTGGGTTTAGGGTTTAGGGTTTAGGGTTTAGGGTTTAGGGTTTAGGGTTTAGGGTTTAGGGTTTAGGGTTTAGGGTTTAGGGTTTAGGGTTTAGGGTTTAGGGTTTAGGGTTTAGGGTTTAGGGTTTAGGGTTTAGGGTTTAGGGTTTAGGGTTTAGGGTTTAGGGTTTAGGGTTTAGGGTTTAGGGTTTAGGGTTTAGGGTTTAGGGTTTAGGGTTTAGGGTTTAGGGTTTAGGGTTTAGGGTTTAGGGTTTAGGGTTTAGGGTTTAGGGTTTAGGGTTTAGGGTTTAGGGTTTAGGGTTTAGGGTTTAGGGTTTAGGGTTTAGGGTTTAGGGTTTAGGGTTTAGGGTTTAGGGTTTAGGGTTTAGGGTTTAGGGTTTAGGGTTTAGGGTTTAGGGTTTAGGGTTTAGGGTTTAGGGTTTAGGGTTTAGGGTTTAGGGTTTAGGGTTTAGGGTTTAGGGTTTAGGGTTTAGGGTTTAGGGTTTAGGGTTTAGGGTTTAGGGTTTAGGGTTTAGGGTTTAGGGTTTAGGGTTTAGGGTTTAGGGTTTAGGGTTTAGGGTTTAGGGTTTAGGGTTTAGGGTTTAGGGTTTAGGGTTTAGGGTTTAGGGTTTAGGGTTTAGGGTTTAGGGTTTAGGGTTTAGGGTTTAGGGTTTAGGGTTTAGGGTTTAGGGTTTAGGGTTTAGGGTTTAGGGTTTAGGGTTTAGGGTTTAGGGTTTAGGGTTTAGGGTTTAGGGTTTAGGGTTTAGGGTTTAGGGTTTAGGGTTTAGGGTTTAGGGTTTAGGGTTTAGGGTTTAGGGTTTAGGGTTTAGGGTTTAGGGTTTAGGGTTTAGGGTTTAGGGTTTAGGGTTTAGGGTTTAGGGTTTAGGGTTTAGGGTTTAGGGTTTAGGGTTTAGGGTTTAGGGTTTAGGGTTTAGGGTTTAGGGTTTAGGGTTTAGGGTTTAGGGTTTAGGGTTTAGGGTTTAGGGTTTAGGGTTTAGGGTTTAGGGTTTAGGGTTTAGGGTTTAGGGTTTAGGGTTTAGGGGTTAGGGTTTAGGGTTTAGGGTTTAGGGTTTAGGGTTGGGTAGGGTTTAGGGTTTAGGGTTTAGGGTTTAGGGTTTAGGGTTTAGGGTTTAGGGTTTAGGGTTTAGGGTTTAGGGTTTAGGGTTTAGGGTTTAGGGTTTAGGGTTTAGGGTTTAGGGTTTAGGGTTTAGGGGTTAGGGTTTAGGGTTTAGGGTTTAGGGTTTAGGGTTTAGGGTTTAGGGTTTAGGGTTTAGGGTTTAGGGTTTAGGGTTAGGGTTTAGGGTTTAGGGTTTAGGGGTAGGTTAGGGTTAGGGTTTAGGGTTAGGGTTTAGGGTTGTAGGGTTTAGGGTTTAGGTTTAGGGTGTAGGGTTTAGGGTTAGGGTATAGGTTTAGGGTTTAGGGTTTAGGGTTTAGGGTTTAGGGTTTAGGGTTTAGGGTTTTAGGGTTTAGGGTTTAGGGTTTAGA

**4BS**

TTAGGG**TTTAGGG**TTTAGGGTTTAGGGTTTAGGGTTTAGGGTTTAGGGTTTTTAGGGTTTAGGGTTTAGGGTTTAGGGTTTAGGGTTAGGGTTAGGGTTAGGGTTTAGGGTTTAGGGTTTAGGGTTTAGGGTTTAGGGTTTAGGGTTTTAGGGTTTAGGGTTTAGGGTTTTTAGGGTTTAGGGTTTAGGGTTTAGGGTTTAGGGTTAGGGTTAGGGTTTAGGGTTTTAGGGTTTTAGGGTTTAGGGTTAGGGTTAGGGTTTAGGGTTAGGGTTAGGGTTTAGGGTTTTAGGGTTTTAGGGTTTAGGGTTAGGGTTAGGGTTTAGGGTTAGGGTTTTAGGGTTTTAGGGTTTAGGGTTAGGGTTTTAGGGTTTAGGGTTTAGGGTTTAGGGTTTAGGGTTTAGGGTTTAGGGTTAGGGTTAGGGTTTAGGGTTTAGGGTTTAGGGTTTAGGGTTTTTAGGGTTTAGGGTTTAGGGTTTAGGGTTTAGGGTTTAGGGTTTAGGGTTTAGGGTTTAGGGTTTAGGGTTTAGGGTTTAGGGTTTAGGGTTTAGGGTTTAGGGTTTAGGGTTTAGGGTTTAGGGTTTAGGGTTTAGGGTTTAGGGTTTAGGGTTTAGGGTTTAGGGTTTAGGGTTTAGGGTTTAGGGTTTAGGGTTTAGGGTTTAGGGTTTAGGGTTTAGGGTTTAGGGTTTAGGGTTTAGGGTTTAGGGTTTAGGGTTTAGGGTTTAGGGTTTAGGGTTTAGGGTTTAGGGTTAGGGTTTAGGGTTTAGGGTTTAGGGTTTAGGGTTAGGGTTTAGGGTTTAGGGTTTAGGGTTTAGGGTTTAGGGTTTAGGGTTTAGGGTTTAGGGTTTAGGGTTTAGGGTTTAGGGTTTAGGGTTTAGGGTTTAGGGTTTAGGGTTTAGGGTTTAGGGTTTAGGGTTTAGGGTTTAGGGTTTAGGGTTTAGGGTTTAGGGTTTAGGGTTTAGGGTTTAGGGTTTAGGGTTAGGGTTTAGGGTTAGGGTTTTTAGGGTTTAGGGTTTAGGGTTTAGGGTTTAGGGTTAGGGTTTAGGGTTTAGGGTTTAGGGTTTAGGGTTAGGGTTTAGGGTTTAGGGTTAGGGTTAGGGTTTAGGGTTTAGGGTTTAGGGTTTAGGGTTTAGGGTTTAGGGTTTAGGGTTTAGGGTTTAGGGTTTAGGGTTTAGGGTTTAGGGTTAGGGTTTAGGGTTAGGGTTAGGGTTAGGGTTTAGGGTTTAGGGTTTAGGGTTAGGGTTTAGGGTTTAGGGTTAGGGTTTAGGGTTTAGGGTTTAGGGTTTAGGGTTTAGGGTTTAGGGTTTAGGGTTAGGGTTTAGGGTTAGGGTTTAGGGTTTAGGGTTTAGGGTTAGGGTTTAGGGTTTAGGGTTTAGGGTTTAGGGTTAGGGTTTAGGGTTTGGGGTTTAGGGTTTAGGGTTTAGGGTTTAGGGTTTAGGGTTAGGGTTAGGGTTAGGGTTAGGGTTTAGGGTTAGGGTTTAGGGTTTAGGGTTAGGGTTAGGGTTTAGGGTTAGGGTTTAGGGTTTAGGGTTTAGGGTTTAGGGTTTAGGGTTTAGGGTTTAGGGTTAGGGTTTAGGGTTTAGGGTTTAGGGTTTAGGGTTTAGGGTTAGGGTTTAGGGTTAGGGTTTAGGGTTAGGGTTAGGGTTTAGGGTTTAGGGTTTAGGGTTTAGGGTTTAGGGTTTAGGGTTTAGGGTTTAGGGTTTAGGGTTAGGGTTTAGGGTTAGGGTTTAGGGTTAGGGTTTAGGGTTAGGGTTTAGGGTTAGGGTTTAGGGTTAGGGTTAGGGTTAGGGTTAGGGTTAGGGTTTAGGGTTTAGGGTTTAGGGTTTAGGGTTTAGGGTTTAGGGTTTAGGGTTTAGGGTTTAGGGTTTAGGGTTTAGGGTTTAGGGTTTAGGGTTTAGGGTTTAGGGTTTAGGGTTAGGGTTAGGGTTTAGGGTTTAGGGTTTAGGGTTAGGGTTAGGGTTAGGGTTAGGGTTTAGGGTTTAGGGTTTAGGGTTTAGGGTTTAGGGTTAGGGTTAGGGTTTAGGGTTTAGGGTTTAGGGGTTTAGGGTTAGGGTTTAGGGTTTAGGGTTTAGGGTTAGGGTTTAGGGTTTTAGGGTTTAGGGTTTAGGGTTTAGGGTTTAGGGTTAGGGTTAGGGTTAGGGTTTAGGGTTTAGGGTTTAGGGTTTAGGGTTTAGGGTTTAGGGTTTAGGTTTAGGGTTTAGGGTTTAGGGTTTAGGGTTTAGGGTTTTAGGGTTTAGGGTTTAGGGTTTTAGGGTTTAGGGTTTAGGGTTTAGGGTTTAGGGTTTAGGGTTTAGGGTTTAGGGTTTAGGGTTTAGGGTTTAGGGTTTAGGGTTTAGGGTTAGGGTTTAGGGTTTAGGGTTTAGGGTTTAGGTTTAGGGTTTAGGGTTTAGGGTTTAGGGTTTAGGGTTTAGGGTTTAGGGTTTAGGGTTTAGGGTTTAGGGTTAGGGTTTAGGGTTAGGGTTAGGGTTAGGGTTTAGGGTTAGGGTTTAGGGTTTAGGGTTAGGGTTTAGGGTTTAGGGTTTAGGGTTAGGGTTTAGGGTTTAGGGTTTTTGGGGTTTAGGGTTTAGGGTTAGGGTTTAGGGTTTAGGGTTTAGGGTTTAGGGTTTAGGGTTTAGGGTTTAGGGTTTAGGGTTTAGGGTTTAGGGTTTAGGGTTTAGGGTTTAGGGTTTAGGGTTTAGGGTTTAGGGTTTAGGGTTAGGGTTTAGGGTTTAGGGTTTAGGGTTTAGGGTTTAGGGTTTAGGGTTTAGGGTTTAGGGTTTAGGGTTTAGGGTTTAGGGTTAGGGTTTAGGGTTTAGGGTTTAGGGTTAGGGTTTAGGGTTTAGGGTTTAGGGTTTAGGGTTTAGGGTTTAGGGTTTAGGGTTTAGGGTTTAGGTTTAGGGTTTAGGGTTTAGGGTTTAGGGTTTAGGGTTTAGGGTTTAGGGTTTAGGGTTTAGGGTTTAGGGTTTAGGGTTTAGGGTTTAGGGTTTGGGGTTTAGGGTTTAGGGTTTAGGGTTTAGGGTTTAGGGTTTAGGGTTTAGGGTTTAGGGTTAGGGTTTAGGGTTTAGGGTTTAGGGTTTAGGGTTTAGGGTTTAGGGTTTAGGGTTTAGGGTTTAGGGTTTAGGGTTTAGGGTTTAGGGTTTAGGGTTTAGGGTTTAGGGTTTAGGGTTTAGGGTTTAGGGTTTAGGGTTTAGGGTTTAGGGTTTAGGGTTTAGGGTTTAGGGTTTAGGGTTAGGGTTTAGGGTTTAGGGTTTAGGGTTTAGGGTTTAGGGTTAGGGTTTAGGGTTTAGGGTTAGGGTTAGGGTTTAGGGTTTAGGGTTTAGGGTTTAGGGTTAGGGTTTAGGGTTTAGGGTTTAGGGTTAGGGTTTAGGGTTTAGGGTTTAGGGTTTAGGGTTTAGGGTTAGGGTTTAGGGTTAGGGTTTAGGGTTTAGGGTAGGTTAGGGTTAGGGTTAGGGTTTAGGGTTTAGGGTTTAGGGTTAGGGTTTAGGGTTTAGGGTTAGGGTTAGGGTTTAGGGGTTAGGGTTTAGGGTTAGGGTTTAGGGTTTAGGGTTTAGGGTTTAGGGTTAGGGTTAGGGTTTAGGGTAGGGTTTAGGGTTTAGGGTTTAGGGTTTAGGGTTTAGGGTTTAGGGTTTAGGGTTTAGGGTTAGGGTTAGGGTTAGGGTTTAGGGTTTAGGGTTTAGGGTTAGGGTTTAGGGTTTAGGGTTTAGGGTTTAGGTTTAGGGTTAGGGTTTAGGGTTTAGGGTTTAGGGTTTAGGGTTTAGGGTTTAGGGTTTAGGGTTTAGGGTTAGGGTTAGGGTTAGGGTTTAGGGTTTAGGGTTTAGGGTTTAGGGTTTAGGGTTTAGGTTAGGGTTTAGGGTTAGGGTTAGGGTTTAGGGTTTAGGGTTTAGGGTTTAGGGTTTAGGGTTTAGGGTTTAGGGTTAGGGTTTAGGGTTTAGGGTTTAGGGTTTAGGGTTTAGGGTTTAGGGTTTAGGGTTTAGGGTTTAGGGTTTAGGGTTAGGGTTTAGGGTTTAGGGTTTAGGTTTAGGGTTTAGGGTTTAGGGTTTAGGGTTAGGGTTTAGGGTTTAGGGTTAGGGTTTAGGTTTAGGGTTTAGGGTTTAGGGTTTAGGGTTTAGGGTTTTAGGGTTTAGGGTTGGTTAGGGTTTAGGGTTTAGGGTTTAGGGTTTAGGGTTTAGGGTTTAGGTTTAGGGTTTAGGGTTTAGGGTTTAGGGTTAGGGTTTAGGGTTTAGGGTTTAGGGTTAGGGTTAGGGTTTAGGGTTTAGGGTTTAGGGTTTAGGGTTTAGGGTTAGGGTTTAGGGTTTAGGGTTTAGGGTTTAGGGTTTAGGGTTTAGGGTTTAAGGGTTTAGGGTTTAGGGTTTAGGGTTTAGGGTTAGGGTTTAGGGTTTAGGGTTTAGGGTTTAGGGTTTAGGGTTAGGGTTAGGGTTAGGGTTAGGGTTTAGGTTTAGGGTTTAGGGTTTAGGGTTTAGGGTTTAGGGTTTAGGGTTTAGGGTTTAGGGTTTAGGGTTTAGGGTTTAGGGTTTAGGGTTTAGGGTTTAGGGTTTAGGGTTTAGGGTTTAGGGTTTTAGGGTTTAGGGTTTAGGGTTTAGGGTTTAGGGTTTAGGGTTAGGGTTTAGGGTTTAGGTTTAGGGTTAGGGTTTAGGGTTTAGGGTTTAGGGTTTAGGGTTTAGGGTTTAGGGTTTAGGGTTTAGGGTTTAGGGTTTAGGGTTTAGGGTTTAGGGTTTAGGGTTTAGGGTTTAGGGTTTAGGGTTTAGGGTTTAGGGTTTAGGGTTTAGGGTTTAGGGTTAGGGTTTAGGGTTTAGGGTTAGGGTTTAGGGTTTAGGGTTTAGGGTTTAGGGTTTAGGGTTTAGGGTTTAGGGTTTAGGGTTTAGGGTTTAGGGTTTAGGGTTTAGGGTTAGGGTTTAGGGTTAGGGTTTAGGGTTTAGGGTTTAGGGTTTAGGGTTTAGGGTTTAGGGTTAGGGTTTAGGGTTTAGGGTTTAGGGTTTAGGGTTTAGGGTTTAGGGTTAGGGTTTAGGGTTTAGGGTTTAGGGTTTAGGGTTTAGGGTTTAGGGTTTAGGGTTTAGGGTTTAGGGTTTAGGGTTTAGGTTTAGGGTTTAGGGTTTAGGGTTTAGGGTTTAGGGTTAGGGTTTAGGGTTTAGGTTGGTTTAGGGTTTAGGGTTTAGGGTTAGGGTTTAGGGTTTAGGGTTTAGGGTTTAGGGTTAGGGTTTAGGGTTTAGGGTTTAGGGTTTAGGGTTTAGGGTTTAGGGTTTAGGGTTTAGGGTTTAGGGTTTAGGGTTTAGGGTTTAGGGTTAGGGTTTAGGGTTTAGGGTTTAGGGTTTAGGGTTTAGGGTTTAGGGTTTAGGGTTTAGGGTTTAGGGTTTAGGGTTTAGGGTTAGGGTTTAGGGTTTAGGGTTTAGGGTTTAGGGTTAGGGTTTAGGGTTTAGGGTTTAGGGTTTAGGGTTTAGGGTTAGGGTTTAGGGTTTAGGGTTTAGGGTTAGGGTTTAGGGTTTAGGGTTTAGGGTTTAGGGTTTAGGTTTAGGGTTAGGGTTAGGGTTTAGGTTTAGGGTTTAGGGTTTTAGGGTTTAGGGTTTAGGGTTTAGGTTTAGGGTTTAGGGTTTAGGGTTTAGGGTTTAGGGTTAGGGTTAGGGTTTAGGGTTTAGGGGTTTAGGGTTTAGGGTTTAGGGTTTAGGGTTTAGGGTTTAGGGTTAGGGTTTAGGGTTTAGGGTTTAGGGTTTAGGGTTTAGGGTTTAGGGTTTAGGGTTTAGGGTTTAGGGTTTAGGGTTTAGGGTTTAGGGTTAGGGTTTAGGGTTTAGGGTTTAGGGTTTAGGGTTTAGGGTTTAGGGTTTAGGGTTAGGGTTTAGGGTTTAGGGTTTAGGGTTTAGGGTTTAGGGTTTAGGGTTTAGGGTTTAGGGTTAGGGTTTAGGGTTAGGGTTTAGGGTTTAGGGTTTAGGGTTTAGGGTTTAGGGTTTAGGGTTTAGGGTTTAGGGTTTAGGGTTTAGGGTTTAGGGTTAGGGTTTAGGGTTTAGGGTTTAGGGTTTAGGGTTTAGGGTTTAGGGTTTAGGGTTTAGGGTTTAGGGTTTAGGTTTAGGGGGTTAGGGTTTAGGGTTTAGGGTTTAGGGTTTAGGGTTTAGGGTTTAGGGTTTAGGGGTTTAGGGTTTAGGGTTTAGGGTTTAGGGTTTAGGGTTTAGGGTTTAGGGTTTAGGGTTAGGGTTTAGGGTTTAGGGTTTAGGGTTTAGGGTTTAGGGTTTAGGGTTTAGGGTTTAGGGTTTAGGGTTTAGGGTTTAGGGTTTAGGGTTTAGGGTTTAGGGTTTAGGGTTAGGGTTTAGGGTTTAGGGTTTAGGGTTTAGGGTTTAGGGTTTAGGGTTTAGGGTTTAGGGTTTAGGGTTTAGGGTTTAGGGTTTAGGGTTTAGGGTTTAGGGTTTAGGGTTTAGGGTTTAGGGTTTAGGGTTTAGGGTTTAGGGTTAGGGTTTAGGGTTTAGGGTTTAGGGTTTAGGGTTTAGGGTTTAGGGTTTAGGGTTTAGGGTTTAGGGTTTAGGGTTAGGGTTTAGGGTTTAGGGTGGTTTAGGGTAGGGTTTAGGGTTAGGGTTTAGGGTTTAGGGTTTAGGGTTTAGGGTTTAGGGTTTAGGGTTTAGGGTTTAGGGTTTAAGGTTTAGGGTTTAGGGTTTAGGGTTTAGGGTTAGGGTTAGGGTTTAGGGTTAGGTTAGGGTTTAGGGTTTAGGGTTAGGGTTAGGGTTTAGGGTTTAGGGTTTAGGGTTTAGGGTTAGGGTTTAGGGTTAGGGTTTAGGTTTAGGGTTAGGGTTAGGGTTGTTAGGGTTTAGGGTTTAGGGTTTAGGGTTAGGGTTTAGGGTTTAGGGTTTAGGGTTAGGGTTTAGGGTTTAGGGTTTAGGGTTAGGGTTTAGGGTTTAGGGTTTAGGGTTTAGGGTTTAGGGTTAGGGTTAGGGTTTAGGGTTAGGGTTTAGGGTTTAGGGTTTAGGGTTTAGGGTTAGGGTTAGGGTTAGGGTTTAGGGTTTAGGGTTTAGGGTTTAGGGTTAGGGTTTAGGGTTTAGGGTTTAGGGTTTAGGGTTTAGGGTTTAGGGTTTAGGGTTTAGGGTTTAGGGTTAGGGTTTAGGGTTAGGGTTAGGGTTTAGGGTTAGGGTTAGGGTTTAGGGTTAGGGTTTAGGGTTAGGGTTAGGGTTTAGGGTTTAGGGTTTAGGGTTTAGGGTTTAGGGTTTAGGGTTTAGGGTTTAGGGTTTTAGGGTTTAGGGTTTGGGTTTAGGGTTTAGGGTTGGTAGGGTTAGGGTTTAGGGTTTAGGGTTAGGATTAGGGTTTAGGGTTAGGGTTTAGGGTTTAGGGTTTAGGGTTTAGGGTTTAGGGTTTAGGGTTTAGGGTTTAGGGTTTAGGGTTTAGGGTTTAGGGTTTAGGGTTTAGGGTTTAGGGTTTTGGGTTTAGGGTTAGGGTTTAGGGTTTAGGGTTTAGGTTTGGGTTAGGGTTTAGGGTTTAGGGTTTAGGGTTTAGGGTTTAGGGTTTAGGGTTTAGGGTTTAGGGTTTAGGGTTTAGGGTTTAGGGTTAGGGTTTAGGGTTTAGGGTTTAGGGTTTAGGGTTTAGGGTTAGGGTTTAGGGTTTAGGGTTTAGGGTTTAGGGTTTAGGGTTTAGGGTTTAGGGTTAGGGTTTAGGGTTTAGGGTTTAGGGTTTAGGGTTTTTAGGGTTTAGGGTTTAGGGTTTAGGGTTTAGGGTTAGGGTTTAGGGTTTAGGGTTTAGGGTTTAGGGTTTAGGGTTTAGGGTTTAGGGTTTAGGGTTTAGGGTTTAGGGTTAGGGTTTAGGGTTTAGGGTTTAGGGTTTAGGGTTTAGGGTTAGGGTTTAGGGTTTAGGGTTTAGGGTTTAGGGTTTAGGGTTTAGGGTTTAGGGTTTAGGGTTTAGGGTTTAGGGTTTAGGGTTTAGGGTTTAGGGTTAGGGTTTAGGGTTAGGGTTTAGGGTTTAGGGTTTAGGGTTTAGGGTTTAGGGTTTAGGGTTAGGGTTTAGGGTTTAGGGTTTAGGGTTTAGGGTTTAGGGTTTAGGGTTTAGGGTTTAGGGTTTAGGGTTTAGGGTTTAGGGTTTAGGGTTTAGGGTTTAGGTTTAGGGTTTAGGGTTAGGGTTTAGGGTTTAGGGTTTAGGGTTTAGGGTTTAGGGTTTAGGGTTTTAGGGTTTGGGGTTTAGGGTTTAGGGTTTAGGGTTAGGGTTTAGGGTTTCAGGGTTTAGGGTTAGGGTTTAGGGTTTAGGGTTTAGGGTTTAGGTTTAGGGTTTAGGGTTTAGGGTTTAGGGTTTAGGGTTTAGGGTTTAGGGTTTAGGGTTTAGGGTTAGGGTTTAGGGTTTAGGGTTTAGGGTTTAGGGTTTAGGGTTTAGGGTTAGGGTTTAGGGTTTAGGGTTTAGGGTTAGGGTTTAGGGTTTAGGGTTTAGGGTTTAGGGTTTAGGGTTTAGGGTTTAGGGTTTAGGGTTTAGGGTTTAGGGTTTAGGGTTTAGGGTTTAGGGTTTAGGGTTTAGGGTTTAGGGTTTAGGGTTTAGGGTTTAGGGGTTAGGGTTTAGGGTTAGGGTTTAGGGTTTAGGGTTTAGGGTTTAGGGTTAGGGTTTAGGGTTTAGGGTTTAGGGTTTAGGGTTTAGGGTTTAGGGTTTTAGGGTTTAGGGTTTAGGGTTTAGGGTTTAGGGTTTAGGGTTTAGGGTTTAGGGTTTAGGGTTTAGGGTTTTAGGGTTTAGGGTTTAGGGTTAGGGTTTAGGGTTTAGGGTTAGGGTTTAGGGTTTGGTTTAGGGTTTAGGGTTTAGGGTTTAGGGTTTAGGGTTTAGGGTTTAGGGTTTAGGGTTTAGGGTTTAGGGTTTAGGGTTTAGGGTTTAGGGTTTAGGGTTTAGGGTTTAGGGTTTAGGGTTTAGGGTTTAGGGTTTAGGGTTTAGGGTTTAGGGTTAGGGTTTTGGGTTTAGGGTTTAGGGTTTAGGGTTTAGGGTTTAGGTTTAGGGTTAGGGTTTAGGGTTTAGGGTTTAGGGTTTAGGGTTTAGGGTTTAGGGTTTAGGGTTTAGGGTTAGGGTTTAGGGTTTAGGGTTTAGGGTTTAGGGTTTAGGGTTAGGGTTTAGGGTTTAGGGTTTAGGGTTTAGGGTTTAGGGTTTAGGGTTTAGGGTTTAGGGTTAGGGTTTAGGGTTTAGGGTTTAGGGTTAGGGTTAGGGTTTAGGGTTAGGGTTAGGGTTTAGGGTTTAGGGTTTAGGTTTAGGGTTTAGGGTTTAGGTTTAGGGTTAGGGTTTAGGGTTTTTAGGGTTTAGGGTTAGGGTTTAGGGTTAGGGTTTAGGGTTTAGGGTTTAGGGTTTAGGGTTTAGGGTTTAGGGTTTAGGGTTTAGGGTTTAGGGTTTAGGGTTAGGGTTTAGGGTTAGGGTTAGGGTTTAGGGTTAGGGTTTAGGGTTTAGGGTTTAGGGGTTTAGGGTTTAGGGTTTAGGGTTTAGGGTTGGTTAGGGTTTAGGGTTTAGGGTTAGGGTTTAGGGTTTAGGGTTTAGGGTTAGGGGTTTAGGGTTTAGGGTTTAGGGTTAGGGTTTAGGGTTTTAGGGTTTTTTAGGGTTTAGGGTTTAGGGTTTAGGGTTTAGGGTTTAGGGTTGGGTTTGGGTTTAGGGTTTAGGGTTTAGGGTTTAGGGTTTAGGGTTTAGGGTTTAGGGTTTAGGGTTTAGGGTTAGGGTTAGGTTTAGGGTTTAGGGTTTAGGGTTTAGGGTTTAGGGTTTAGGGTTAGGGT

**1DS**

AAGGG**TTTAGGG**TTTAGGGTTAGGGTTTAGGGTTAGGGTTTAGGGTTTAGGGTTTAGGGTTTAGGGTTAGGGTTTAGGGTTTAGGGTTAGGGTTTAGGGTTTAGGGTTTAGGGTTTAGGGTTTAGGGTTTAGGGTTTAGGGTTAGGGTTTAGGGTTTAGGGTTTAGGGTTTAGGGTTTAGGGTTTAGGGTTTAGGGTTTAGGGTTTAGGGTTTAGGGTTAGGGTTTAGGGTTTAGGGTTTAGGGTTTAGGGTTTAGGGTTAGGGTTTAGGGTTAGGGTTTAGGGTTTAGGGTTTAGGGTTTAGGGTTTAGGGTTTAGGGTTTAGGGTTTAGGGTTTAGGGTTTAGGGTTTAGGGTTTAGGGTTTAGGGTTTAGGGTTAGGGTTAGGGTTTAGGGTTTAGGGTTTAGGGTTTAGGGTTTAGGGTTTAGGGTTTAGGGTTTAGGGTTTAGGGTTTAGGGTTTAGGGTTTAGGGTTTAGGGTTTAGGGTTTAGGGTTTAGGGTTTAGGGTTTAGGGTTTAGGGTTTAGGGTTTAGGGTTTAGGGTTTAGGGTTTAGGGTTTAGGGTTTAGGGTTTAGGGTTTAGGGTTTAGGGTTTAGGGTTTAGGGTTTAGGGTTTAGGGTTTAGGGTTTAGGGTTTAGGGTTTAGGGTTTAGGGTTTAGGGTTTAGGGTTTAGGGTTTAGGGTTTAGGGTTTAGGGTTTAGGGTTTAGGGTTTAGGGTTTAGGGTTTAGGGTTTAGGGTTTAGGGTTTAGGGTTTAGGGTTTAGGGTTTAGGGTTTAGGGTTTAGGGTTTAGGGTTTAGGGTTTAGGGTTTAGGGTTTAGGGTTTAGGGTTTAGGGTTTAGGGTTTAGGGTTTAGGGTTTAGGGTTTAGGGTTTAGGGTTTAGGGTTTAGGGTTTAGGGTTTAGGGTTTAGGGTTTAGGGTTTAGGGTTTAGGGTTTAGGGTTTAGGGTTTAGGGTTTAGGGTTTAGGGTTTAGGGTTTAGGGTTTAGGGTTTAGGGTTTAGGGTTTAGGGTTTAGGGTTTAGGGTTTAGGGTTTAGGGTTTAGGGTTTAGGGTTTAGGGTTTAGGGTTTAGGGTTTAGGGTTTAGGGTTTAGGGTTTAGGGTTTAGGGTTTAGGGTTTAGGGTTTAGGGTTTAGGGTTTAGGGTTTAGGGTTTAGGGTTTAGGGTTTAGGGTTTAGGGTTTAGGGTTTAGGGTTTAGGGTTTAGGGTTTAGGGTTTAGGGTTTAGGGTTTAGGGTTTAGGGTTTAGGGTTTAGGGTTTAGGGTTTAGGGTTTAGGGTTTAGGGTTTAGGGTTTAGGGTTTAGGGTTTAGGGTTTAGGGTTTAGGGTTTAGGGTTTAGGGTTTAGGGTTTAGGGTTTAGGGTTTAGGGTTTAGGGTTTAGGGTTTAGGGTTTAGGGTTTAGGGTTTAGGGTTTAGGGTTTAGGGTTTAGGGTTTAGGGTTTAGGGTTTAGGGTTTAGGGTTTAGGGTTTAGGGTTTAGGGTTTAGGGTTTAGGGTTTAGGGTTTAGGGTTTAGGGTTTAGGGTTTAGGGTTTAGGGTTTAGGGTTTAGGGTTTAGGGTTTAGGGTTTAGGGTTTAGGGTTTAGGGTTTAGGGTTTAGGGTTTAGGGTTTAGGGTTTAGGGTTTAGGGTTTAGGGTTTAGGGTTTAGGGTTTAGGGTTTAGGGTTTAGGGTTTAGGGTTTAGGGTTTAGGGTTTAGGGTTTAGGGTTTAGGGTTTAGGGTTTAGGGTTTAGGGTTTAGGGTTTAGGGTTTAGGGTTTAGGGTTTAGGGTTTAGGGTTTAGGGTTTAGGGTTTAGGGTTTAGGGTTTAGGGTTTAGGGTTTAGGGTTTAGGGTTTAGGGTTTAGGGTTTAGGGTTTAGGGTTTAGGGTTTAGGGTTTAGGGTTTAGGGTTTAGGGTTTAGGGTTTAGGGTTTAGGGTTTAGGGTTTAGGGTTTAGGGTTTAGGGTTTAGGGTTTAGGGTTTAGGGTTTAGGGTTTAGGGTTTAGGGTTTAGGGTTTAGGGTTTAGGGTTTAGGGTTTAGGGTTTAGGGTTTAGGGTTTAGGGTTTAGGGTTAGGGTTTAGGGTTTAGGGTTTAGGGTTTAGGGTTTAGGGTTTAGGGTTTAGGGTTTAGGGTTTAGGGTTTAGGGTTTAGGGTTTAGGGTTTAGGGTTTAGGGTTTAGGGTTTAGGGTTTAGGGTTTAGGGTTTAGGGTTTAGGGTTTAGGGTTTAGGGTTTAGGGTTTAGGGTTTAGGGTTTAGGGTTTAGGGTTTAGGGTTTAGGGTTTAGGGTTTAGGGTTTAGGGTTTAGGGTTTAGGGTTTAGGGTTTAGGGTTTAGGGTTTAGGGTTTAGGGTTTAGGGTTTAGGGTTTAGGGTTTAGGGTTTAGGGTTTAGGGTTTAGGGTTTAGGGTTTAGGGTTTAGGGTTAGGGTTTAGGGTTTAGGGTTAGGGTTAGGGTTTAGGGTTTAGGGTTTAGGGTTTAGGGTTAGGGTTTAGGGTTAGGGTTTAGGGTTTAGGGTTAGGGTTAGGGTTTAGGGTTTAGGGTTTAGGGTTAGGGTTTAGGGTTTAGGGTTTAGGGTTTAGGGTTTAGGGTTTAGGGTTTAGGGTTTAGGGTTTAGGGTTTAGGGTTTAGGGGTTAGGGTTTAGGGTTTAGGGTTTAGGGTTAGGGTTTAGGGTTTAGGGTTTAGGGTTTAGGGTTTAGGGTTTAGGGTTAGGGTTAGGGTTTAGGGTTTAGGGTTTAGGGTTAGGGTTTAGGGTTTAGGGTTAGGGTTTAGGGTTTAGGGTTTAGGGTTTAGGGTTTAGGGTTTAGGGTTTAGGGTTTAGGGTTTAGGGTTTAGGGTTTAGGGTTTAGGGTTTAGGGTTAGGTTTAGGGTTTAGGGTTTGGTTAGGGTTTAGGGTTTAGGGTTTAGGGTTTAGGGTTTAGGGTTTAGGGTTTAGGGTTTAGGGTTAGGGTTTAGGGTTTAGGGTTTAGGGTTTAGGGTTTAGGGTTTAGGGTTTAGGGTTTAGGGTTAGGGTTTAGGGTTTAGGGTTTAGGGTTTAGGGTTTAGGGTTTAGGGTTAGGGTTTAGGGTTTAGGGTTTAGGGTTAGGGTTTAGGGTTTAGGGTTAGGGTTTAGGGTTTAGGGTTTAGGGTTTAGGGTTAGGGTTTAGGGTTTAGGGTTTAGGGTTTAGGGTTTAGGGTTTAGGGTTTAGGGTTTAGGGTTTAGGGTTTAGGGTTTAGGGTTTAGGGTTAGGGTTAGGGTTTAGGGTTTAGGGTTTAGGGTTTAGGGTTTAGGGTTTAGGGTTTAGGGTTTAGGGTTTAGGGTTTAGGGTTTAGGGTTTAGGGTTAGGGTTTAGGGTTTAGGGTTTAGGGTTTAGGGTTTAGGGTTAGGGTTTAGGGTTTAGGGTTAGGGTTTAGGGTTTAGGGTTTAGGGTTTAGGGTTTAGGGTTTAGGGTTTAGGGTTTAGGGTTTAGGGTTTAGGGTTTAGGGTTTAGGGTTTAGGGTTTAGGGTTTAGGGTTAGGGTTTAGGGTTTAGGGTTTAGGGTTTAGGGTTTAGGGTTTAGGGTTTAGGGTTTAGGGTTTAGGGTTTAGGGTTTAGGGTTTAGGGTTTAGGGTTTAGGGTTTAGGGTTTAGGGTTTAGGGTTTAGGGTTTAGGGTTTAGGGTTTAGGGTTTAGGGTTTAGGGTTTAGGGTTTAGGGTTTAGGGTTTAGGGTTTAGGGTTTAGGGTTTAGGGTTTAGGGTTTAGGGTTTAGGGTTTAGGGTTTAGGTTTAGGGTTTAGGGTTTAGGGTTTAGGGTTTAGGGTTTAGGGTTTAGGGTTTAGGGTTTAGGGTTTAGGGTTTAGGGTTTAGGGTTTAGGGTTTAGGGTTTAGGGTTTAGGGTTTAGGGTTTAGGGTTTAGGGTTTAGGGTTTAGGGTTTAGGGTTTAGGGTTAGGGTTTAGGGTTTAGGGTTTAGGGTTTAGGGTTTAGGGTTTAGGGTTTAGGGTTTAGGGTTTAGGGTTTAGGGTTTAGGGTTAGGGTTAGGGTTTAGGTTTAGGGTTTAGGGTTTAGGGTTTAGGGTTTAGGGTTTAGGGTTTAGGGTTTAGGGTTTAGGGTTTAGGGTTTAGGGTTTAGGGTTTAGGGTTTAGGGTTTAGGGTTAGGGTTTAGGGTTTAGGGTTTAGGGTTTAGGGTTTAGGGTTTAGGGTTTAGGGTTTAGGGTTTAGGGTTTAGGGTTTAGGGTTTAGGGTTTAGGGTTTAGGGTTAGGGTTTAGGGTTTAGGGTTTAGGGTTTAGGGTTTAGGGTTTAGGGTTTAGGGTTTAGGGTTTAGGGTTAGGGTTTAGGGTTTAGGGTTTAGGGTTTAGGGTTTAGGGTTAGGGTTTAGGGTTTAGGGTTTAGGGTTTAGGGTTTAGGGTTTAGGGTTTAGGGTTTAGGGTTTAGGGTTTAGGGTTTAGGGTTTAGGGTTTAGGGTTTAGGGTTTAGGGTTTAGGGTTTAGGGTTTAGGGTTAGGGTTTAGGGTTTAGGGTTTAGGGTTTAGGGTTTAGGGTTTAGGGTTTAGGGTTTAGGGTTAGGGTTTAGGGTTTAGGGTTTAGGGTTAGGGTTTAGGGTTTAGGGTTTAGGGTTTAGGGTTTAGGGTTTAGGGTTTAGGGTTTAGGGTTTAGGGTTTAGGGTTTAGGGTTTAGGGTTTAGGGTTTAGGGTTTAGGGTTTAGGGTTTAGGGTTAGGGTTTAGGGTTTAGGGTTTAGGGTTTAGGGTTTAGGGTTAGGGTTTAGGGTTTAGGGTTTAGGGTTTAGGGTTTAGGGTTAGGGTTTAGGGTTTAGGGTTTAGGGTTTAGGGTTTAGGGTTTAGGGTTAGGGTTTAGGGTTTAGGGTTTAGGGTTTGGTTAGGGTTTAGGGTTTAGGGTTAGGGTTTAGGGTTTAGGGTTTAGGGTTAGGGTTTAGGGTTTAGGGTTTAGGGTTTAGGGTTTAGGGTTTAGGGTTAGGGTTTAGGGTTTAGGGTTTAGGGTTTAGGGTTTAGGGTTTAGGGTTAGGGTTAGGGTTTAGGGTTTAGGGTTTAGGGTTTAGGGTTTAGGGTTTAGGGTTTAGGGTTAGGGTTTAGGGGTTTAGGGTTAGGGTTTAGGGTTAGGGTTTAGGGGTAGGGGTTTAGGGTTAGGGTTAGGTTTTAGGGTTTAGGGTTTAGGGTTAGGGTTTAGGGTTTAGGGTTTAGGGTTAGGGTTTAGGGTTAGGTTAGGGTTTAGGGTGTAGGGGTTTAGGGTTTAGGGTTAGGGTTTGGGTTTAGGGTTTAGGGTTTAGGGTTTAGGGTTTAGGGTTTAGGGGTTAGGGGTTTAGGGTTTAGGGTTTAGGGTTTAGGGTTTAGGGTTAGGGTTTAGGGTTTAGGGTTTAGGGTTTAGGGTTTAGGGTTAGGGTTTAGGGTTTAGGGTTTAGGGTTTAGGGTTTAGGGTTTAGGGTTTAGGGTTTAGGGTTTAGGTTTAGGGTTTAGGGTTTAGGGTTTAGGGTTTAGGGTTAGGGTTTAGGGTTTAGGGTTTAGGGTTTAGGGTTAGGGTTTAGGGTTTAGGGTTTAGGGTTTAGGGTTTAGGGTTTAGGGTTTAGGGTTTAGGGTTTAGGGTTTAGGGTTTAGGGTTTAGGGTTTAGGGTTTAGGGTTTAGGGTTTAGGGTTTAGGGTTTAGGTTTAGGGTTTAGGGTTTAGGGTTTAGGGTTTAGGGTTTAGGGTTTAGGGTTTAGGGTTTAGGGTTTAGGGTTTTAGGGTTTAGGGTTTAGGGTTTAGGGTTTAGGTTTAGGGTTTAGGTTTAGGGTTTAGGGTTTAGGGTTTAGGGTTTAGGGTTTAGGGTTTAGGGTTTAGGGTTTAGGGTTAGGGTTTAGGGTTTAGGGTTTAGGGTTTAGGGTTTAGGGTTTAGGTTTAGGGTTTAGGGTTTAGGGTTAGGGTTTAGGGTTTAGGGTTTAGGGTTTAGGGTTTAGGGTTTAGGGTTTAGGGTTTAGGGTTTAGGGTTTAGGTTTAGGGTTTAGGGTTTAGGGTTTAGGGTTTAGGGTTTAGGGTTTAGGGTTTAGGGTTAGGGTTAGGGTTTAGGGTTTAGGGTTAGGGTTTAGGGTTTAGGGTTTAGGGTTTAGGGTTTAGGGTTTAGGGTTTAGGTTTAGGGTTTAGGGTTTAGGGTTTAGGGTTTAGGGTTTAGGGTTTAGGGTTTAGGGTTTAGGGTTTAGGGTTTAGGGTTTAGGGTTTAGGGTTTAGGGTTTAGGGTTTAGGGTTAGGGTTTAGGGTTTAGGGTTTAGGGTTAGGGTTTAGGGTTTAGGGTTAGGGTTTAGGGTTTAGGGTTTAGGGTTTAGGGTTTAGGGTTTAGGGTTTAGGGTTTAGGGTTTAGGGGTTTAGGGTTTAGGGTTTAGGGTTTAGGGTTTAGGGTTTAGGGTTTAGGGTTTAGGGTTTAGGGTTTAGGGTTTAGGGTTAGGGTTTAGGGTTTAGGGTTTAGGGTTTAGGGTTTAGGGTTTAGGGTTTAGGGTTTAGGGTTTAGGGTTTAGGGTTTAGGGTTTAGGGTTTAGGGTTTAGGGTTTAGGGTTTAGGGTTTAGGGTTTAGGGTTTAGGGTTTAGGGTTTAGGGTTTAGGGTTTAGGGTTTAGGGTTTAGGGTTTAGGGTTTAGGGTTTAGGGTTTAGGGTTTAGGGTTTAGGGTTTAGGGTTTAGGGTTTAGGGTTTAGGGTTTAGGGTTTAGGGTTTAGGGTTAGGGTTTAGGGTTTAGGGTTAGGGTTTAGGGTTTAGGGTTTAGGGTTTAGGGTTTTAGGGTTTAGGGTTTAGGGTTTAGGGTTTAGGGTTTAGGGTTTAGGGTTTAGGGTTTTAGGGTTTAGGGTTTAGGGTTTAGGGTTTAGGGTTTAGGGTTTAGGGTTTAGGTTTAGGGTTTAGGGTTTAGGGTTAGGGTTTAGGGTTTAGGGTTTAGGGTTTAGGGTTTAGGGTTTAGGGTTTAGGGTTAGGGTTTAGGGTTTAGGGTTTAGGGTTTAGGGTTTAGGGTTAGGGTTAGGGTTTAGGGTTTAGGGTTAGGGTTTAGGGTTTAGGGTTTAGGGTTTAGGGTTTAGGGTTTAGGGTTTAGGGTTTGGGTTTAGGGTTTAGGGTTTAGGGTTTAGGGTTTAGGGTTTAGGGTTTAGGGTTTAGGGTTTAGGTTTAGGGTTTAGGGTTTAGGGTTTAGGGTTTAGGGTTTAGGGTTTAGGGTTAGGGTTTAGGGTTTAGGGTTTAGGGTTTAGGTTTAGGGTTAGGGTTTAGGGTTTAGGGTTTAGGGTTTAGGGTTTAGGGTTTAGGGTTTAGGGTTTAGGGTTTAGGGTTAGGGTTTAGGGTTTAGGGTTTAGGGTTTAGGGTTTAGGGTTTAGGGTTTAGGGTTTAGGGTTAGGGTTTAGGGTTTAGGGTTTAGGGTTTAGGGTTTAGGGTTTAGGGTTTAGGGTTTAGGGTTTAGGTTTAGGGTTTAGGGTTTAGGGTTTAGGGTTTAGGGTTTAGGGTTTAGGGTTTAGGGTTTAGGGTTTAGGGTTTAGGGTTTAGGGTTTAGGGTTTAGGGTTTAGGGTTTAGGGTTTAGGGTTTAGGGTTAGGGGTTAGGGTTTAGGGTTTAGGGTTTAGGGTTTAGGGTTTAGGGTTTAGGGTTTAGGGTTTAGGGTTAGGGTTTAGGGTTTAGGGTTTAGGGTTTAGGGTTTAGGGTTTAGGGTTTAGGGTTTAGGGTTTAGGGTTTAGGGTTTAGGGTTAGGGTTTAGGGTTTAGGGTTTAGGGTTAGGGTTTAGGGTTTAGGGTTTAGGGTTTTAGGGTTAGGGTTTAGGGTTAGGGTTTAGGTTAGGGTTTAGGGTTTAGGGTTTAGGGTTTAGGTTTAGGGTTTAGGGTTTAGGGTTTAGGGTTTAGGGTTTAGGGTTAGGGTTTAGGGTTTAGGGTTTAGGGTTAGGGTTTAGGGTTTAGGGTTTAGGGTTTAGGGTTTAGGGTTTAGGGTTTAGGGTTTAGGGTTTAGGGTTTAGGGTTTAGGGTTTAGGTTTAGGGTTTAGGGTTTAGGGTTTAGGGTTTAGGGTTTTAGGGTTTAGGGTTTAGGGTTAGGGTTTAGGGTTTAGGGTTTAGGGTTTAGGGTTTAGGGTTTAGGGTTTAGGGTTTAGGGTTTAGGGTTTAGGGTTTAGGGTTTAGGGTTTAGGGTTAGGGTTTAGGGTTTAGGGTTTAGGGTTAGGGTTTAGGGTTTAGGGTTTAGGGTTTAGGGTTTAGGGTTAGGGTTTAGGGTTAGGGTTTAGGGTTTAGGGTTTAGGGTTTAGGGTTTAGGGTTTAGGGTTTAGGGTTTAGGGTTAGGTTTAGGGTTTAGGGTTTAGGGTTTAGGGTTTAGGTTTAGGGTTTAGGGTTAGGGTTTAGGGTTAGGGTTTAGGGTTTAGGGTTTAGGGTTTAGGGTTTAGGGTTTAGGGTTAGGGTTAGGGTTAGGGTTTAGGGTTTAGGGTTTAGGGTTTAGGGTTTAGGGTTTAGGGTTTAGGGTTAGGGTTAGGGTTTAGGGTTTAGGGTTTAGGGTTTAGGGTTTAGGGTTTAGGGTTAGGGTTAGGGTTTAGGGTTTAGGGTTTAGGGTTTAGGGTTTAGGGTTTAGGGTTTAGGGTTTAGGGTTTAGGGTTTAGGGTTAGGGTTTAGGGTTTAGGGTTAGGTTTAGGGTTTAGGGTTTAGGGTTTAGGGTTAGGGTTTAGGGTTTAGGGTTTAGGGTTAGGGTTTAGGGTTTAGGGTTTAGGGTTTAGGGTTTAGGGTTTAGGGTTTAGGGTTAGGGTTTAGGGTTTAGGGTTAGGGTTAGGGTTTAGGGTTAGGGTTAGGGTTTAGGGTTTAGGGTTTAGGGTTTAGGGTTTAGGGTTAGGGTTTAGGGTTTAGGGTTTAGGGTTTAGGGTTTAGGGTTAGGGTTAGGTTTAGGGTTTGTAGGGTTTAGGGTTTAGGGTTTAGGGTTTAGGGTTAGGGTTTAGGGTTAGGGTTTAGGGTTTAGGGTTTAGGGTTAGGGTTAGGGGGTTTAGGGTTTAGGGTTTAGGGTTTAGGGTTTAGGGTTAGGGTTTAGGGTTAGGGTTTAGGGTTTAGGGTTTAGGGTTTAGGGTTTAGGGTTTAGGGTTAGGGTTTAGGGTTTAGGGTTTAGGGTTTAGGGTTTAGGGTTAGGGTTTAGGGTTTAGGGTTAGGGTTTAGGGTTTAGGGTTAGGGTTTAGGGTTTAGGGTTTAGGGTTTAGGGTTTAGGGTTAGGGTTTAGGGTTAGGGTTTAGGGTTTAGGGTTTAGGGTTTAGGGTTTAGGGTTTAGGGTTTAGGGTTTAGGGTTAGGGTTAGGGTTTAGGGTTAGGGTTAGGGTTTAGGGTTTAGGGTTAGGGTTTAGGGTTTAGGGTTTAGGGTTTAGGGTTTAGGGGTTTAGGGTTTAGGGTTTAGGGTTAGGGTTTAGGGTTAGGGTTTAGGGTTTAGGGTTTAGGGTTTAGGGTTAGGGTTTAGGGTTAGGGTTTAGGGTTAGGGTTAGGGTTTAGGGTTTAGGGTTAGGGTTTAGGGTTTAGGTTTAGGGTTTAGGGTTAGGGTTTAGGGTTTAGGGTTTAGGGTTTAGGGTTTAGGGTTAGGGTTTAGGGTTAGGGTTTAGGGTTAGGGTTTAGGGTTAGGGTTTAGGGTTTAGGGTTAGGGTTTAGGGTTAGGGTTTAGGGTTTAGGGTTTAGGGTTTAGGGTTTAGGGTTTAGGGTTTAGGGTTTAGGGTTTAGGTTTAGGGTTAGGTTAGGGTTTAGGGTTTAGGGTTTAGGGTTTAGGGTTTAGGGTTTAGGGTTAGGGTTTAGGGTTAGGGTTTAGGGTTAGGGTTTAGGGTTAGGGTTTTAGGGTTTAGGGGTTTAGGGTTTAGGGTTTAGGGTTAGGGTTTAGGGTTTAGGGTTTAGGGTTAGGGTTTAGGGTTAGGGTTTAGGGTTTAGGGTTTAGGGTTAGGGTTTAGGGTTAGGGTTTAGGGTTTAGGGTTAGGGTTAGGGTTAGGGTTTAGGGTTTAGGGTTAGGGTTTAGGGTTAGGGTTTAGGGTTTAGGTTT

**2DS**

TTTTAGGG**TTTAGGG**TTTAGGGTTTAGGGTTTAGGGTTTAGGGTTAGGGTTTTAGGGTTTAAGGGTTAGGGTTTAGGTTTAGGGTTTAGGGTTTAGGGTTAGGTTTAGGGTTTAGGGTTTAGGGTTTAGGGTTTAGGGTTAGGGTTTAGGGTTTAGGGTTAGGGTTTTAGGGTTTAGGGTTTAGGGTTTAGGGTTTAGGGTTTAGGGTTTGGGTTTAGGGTTAGGGTTTAGGGTTTGGGGTTTAGGGGTTTAGGGTTTAGGGGTTTAGGGTTTAGGGTTTAGGGTTAGGGTTTAGGGTTTAGGGTTTTAGGGTTTAGGGTTTAGGGTTTAGGGTTAGGGTTTAGGGTTTAGGGTTAGGGTTTAGGGTTTAGGGTTAGGGTTTAGGGTTAGGGTTTAGGGTTTAGGGTTAGGGTTTAGGGTTTAGGGTTTAGGGTTTAGGGTTTAGGGTTAGGGTTTAGGGTTTAGGGTTAGGGTTTAGGGTTTAGGGTTTAGGGTTTAGGGTTTAGGGTTTAGGGTTTAGGGTTTAGGGTTAGGGTTTAGGGTTTAGGGTTTAGGGTTTAGGGTTTTAGGGTTTAGGGTTTAGGGTTTAGGGTTTAGGGTTTAGGGTTTAGGGTTTAGGGTTTAGGGTTTAGGGTTTAGGGTTTAGGGTTTAGGGTTTAGGGTTTAGGGTTAGGGTTTAGGGTTTAGGGTTTAGGGTTAGGGTTTAGGGTTTAGGGTTTAGGGTTTAGGGTTAGGGTTTAGGGTTTGGGTTTAGGGTTTAGGGTTTAGGGTTTAGGGTTTAGGGTTTAGGGTTTTAGGGTTAGGGTTTAGGGTTTAGGGTGGTTTAGGGTTAGGGTTTAGGGTTTAGGGTTTAGGGTTTAGGGTTAGGGTTTAGGGTTTAGGGTTTAGGGTTTAGGGTTTAGGGTTTAGGGTTTAGGGTTTAGGGTTTAGGGTTAGGGTTTAGGGTTAGGGTTTAGGGTTTAGGGTTTAGGGTTTAGGGTTTAGGGTTTAGGGTTTAGGGTTTAGGGTTTAGGGTTAGGGTTTAGGGTTTAGGGTTAGGGTTTAGGGTAGGGTTTAGGGTTTAGGGTTTAGGGTTTAGGGTTAGGGTTTAGGGTTTAGGGTTTAGGGTTTAGGGTTTAGGGTTTAGGGTTTAGGGTGGGTAGGGTTTAGGGTTAGGGTTAGGGTTAGGGTTAGGGTTTAGGGTTTAGGGTTTAGGGGTTTAGGGTTTAGGGTTGGGTTTAGGGTTTAGGGTTTAGGGTTAGGGTTTAGGGTTTAGGGTTTAGGGTTTAGGTTAGGGTTTAGGGTTTAGGGTTTAGGGTTTAGGGTTTAGGGTTTAGGGTTTAGGGTTTAGGGTTTAGGGTTAGGGTAGGGTTAGGGTTAGGGTTTAGGGTTTAGGGTTAGGGGTTTAGGGTTTAGGGTTTAGGGTTTAGGGTTAGGGTTAGGGTTTAGGGTTTAGGGTTTAGGGTTTAGGGTTTAGGGTTTAGGGTTAGGGTTTAGGGTTTAGGGTTTAGGGTTAGGGTTTAGGGTTTAGGGTTTAGGGTTTAGGGTTTAGGGTTTAGGGGTTAGGGTTTAGGGTTTAGGGGTTTAGGGTTTAGGGTTTAGGGTTTAGGGTTTAGGGTTTAGGGTTTAGGGTTTAGGGTTAGGGTTTAGGGTTTAGGGTTTAGGGTTTAGGGTTAGGGTTTAGGGTTTAGGGTTTAGGGTTAGGGTTTAGGGTTAGGGTTAGGGTTAGGGTTTAGGGTTTAGGGTTTAGGGTTTAGGGTTAGGGTTTAGGGGTTTAGGGTTTAGGGTTTAGGGTTAGGGTTTAGGGTTTAGGGTTTAGGGTTTAGGGTTAGGGTTTAGGGTTTAGGGTTAGGGTTTAGGGTTTAGGGGTTTAGGGTTTAGGGTTTAGGGTTAGGGTTTAGGGTTTAGGGTTTAGGGTTAGGGGTTAGGGTTTAGGGTTTAGGGTTAGGGTTTAGGGTTTAGGGTTTAGGGTTAGGGTTTAGGGTTTAGGGTTTAGGGTTTAGGGTTTAGGGGTTTAGGGTTAGGGTTTAGGGTTAGGGTTAGGGTTTAGGGTTTAGGGTTTAGGGTTTAGGGTTTAGGGTTTAGGGTTTAGGGTTTAGGGTTTAGGGTTAGGGTTTAGGGTTTAGGGTTTAGGGTTTAGGGTTTAGGGTTTAGGGTTTAGGGGTTTAGGGTTTAGGGTTTAGGGTTTAGGGTTAGGGTTAGGGTTTAGGGTTAGGGTTAGGGTTTAGGGTTTAGGGTTTAGGGTTTAGGTTTAGGGTTTAGGGTTTAGGGTTAGGGTTTAGGGTTTAGGGTTTAGGGTTTAGGGTTTAGGGTTTAGGGTTTAGGGTTTAGGGTTTAGGGTTTAGGGTTGGGTTTAGGGTTTTTAGGGTTTAGGGTTAGGGTTTAGGGTTTAGGGTTTAGGGTTTAGGGTTAGGGTTTAGGGTTTAGGTTTAGGGTTTAGGGTTTAGGGTTTAGGGTTAGGGTTTAGGGTTTTAGGGTTTAGGGTTTAGGGTTTAGGGTTTAGGGTTTAGGGTTTAGGGTTAGGGTTTAGGGTTAGGGTTTAGGGTTAGGGTTAGGGTTAGGGTTTAGGGTTTAGGGTTTAGGGTTTAGGGTTTAGGGTTAGGGTTTAGGGTTTAGGGTTTAGGGTTTAGGGTTTAGGGTTTAGGGTTTAGGGTTTAGGGTTTAGGGTTTAGGGTTTAGGGTTAGGGTTTAGGGTTTAGGGTTAGGGTTTTAGGGTTTAGGGTTTAGGGTTTAGGGTTTAGGGTTGGTTAGGGTTTAGGGTTTAGGGTTTAGGGTTAGGGTTTAGGGTTTTAGGGTTTAGGGTTTAGGGTTTAGGGTTAGGGTTTAGGGTTTAGGGTTTAGGGTTTAGGGTTTAGGGTTAGGGTTTAGGGTTTAGGGTTGGGTTTAGGGTTTGGGTTTAGGGTTTAGGGTTTAGGGTGTTAGGGTTTAGGGTTTTTAGGGTTAGGGTTTAGGGTTTAGGGTTAGGGTTAGGGTTAGGGTTAGGGGTTTAGGGTTAGGGGTTAGGGTTTAGGGTTTAGGGTTTAGGGTTTAGGGTTAGGGTTTAGGGTTAGGGTTTAGGGTTAGGGTTTAGGGTTTAGGGTTTAGGGTTTAGGGTTAGGGTTTAGGGTTTAGGGTTTAGGGTTAGGGTTTAGGGTTTAGGGTTTAGGGTTTAGGGTTTAGGGTTAGGGTTTAGGGTTTAGGGTTTAGGGTTTAGGGTTTAGGGTTAGGGTTTAGGGTTGTTAGGGTTTGTAGGGTTTAGGGGTTTAGGGTTAGGGTTTAGGGTTTAGGGTTTAGGGTTTAGGGTTTAGGGTTTAGGGTTAGGGTTTAGGGTTTAGGGTTTAGGGTTTAGGGTTAGGTTTAGGGTTTAGGGTTTAGGGTTTAGGGTAGGGTTTAGGGTTTAGGGTTTAGGGTTAGGGTTTAGGGTTTAGGGTTTAGGTTTAGGGTTTAGGGTTTAGGGTTTAGGGTTTAGGGTTTAGGGTTAGGGTTTAGGGTTAGGGTTTAGGGTTTAGGGTTTAGGGTTTAGGGTTTAGGGTTTAGGGTTTAGGGTTTAGGTTTAGGTTAGGGTTTAGGGTTTAGGGTTAGGGTTTAGGGTTTAGGGTTTAGGGTTAGGGTTTAGGGTTTAGGGTTTAGGGTTTAGGGTTTAGGGTTTTAGGGTTTAGGGTTTAGGGTTAGGGTTTAGGGTTTAGGGTTAGGGTTTAGGGTTTAGGGTTTAGGGTTTAGGGTTAGGGTTTAGGGTTTAGGGTTTAGGGTTTAGGGTTTAGGGTTTAGGGTTTAGGGTTTAGGGTTTAGGGTTTAGGGTTAGGGTTTAGGGTTAGGGTTAGGGTTTAGGGTTTAGGGTTAGGGTTTAGGGTTTAGGGTTTAGGGTTTTAGGGTTTAGGGTTTAGGGTTTAGGGTTTAGGGTTTAGGTTAGGGTTTAGGGTTTAGGGTTTAGGGTTTAGGGTTTAGGGTTTAGGGTTAGGGTTTAGGGTTTAGGGTTTAGGGTTTAGGGTTAGGGTTTAGGGTTTAGGGTTTAGGGTTTAGGGTTAGGGTTTAGGGTTTAGGGTTTAGGGTTTAGGGTTTAGGTTTAGGGTTTAGGGTTAGGGTTTAGGGTTTAGGGTTTAGGGTTTAGGGTTTAGGGTTTAGGGTTTAGGGTTTAGGGTTTAGGGTTTAGGGTTTAGGGTTTAGGGTTAGGGTTTAGGGTTTAGGGTTTAGGTTTAGGGTTTAGGGTTTAGGGTTTAGGGTTTAGGGTTTAGGGTTTAGGGTTTAGGGTTGGTTAGGGTTTAGGGTTAGGGTTTTAGGGTTTAGGGTTTAGGGTTTAGGGTTTAGGGTTTAGGGTTTTAGGGTTTAGGGTTTAGGGTTTAGGGTTAGGGTTTAGGGTTTAGGGTTAGGGTTTAGGGTTTAGGGTTTAGGGTTTAGGTTTAGGGTTTAGGGTTAGGGTTTAGGGTTTAGGGTTTAGGGTTTAGGGTTTAGGGTTTAGGGTTTAGGGTTTAGGGTTTAGGGTTTAGGGTTTAGGGTTAGGGTTTAGGTTTAGGGTTAGGGTTTAGGGTTAGGGTTTAGGGTTTAGGGTTAGGGTTTAGGGTTTAGGGTTTAGGGTTTAGGGTTTAGGGTTTAGGGTTTAGGGTTTAGGTTAGGTTTAGGGTTTAGGGTTAGGGTTTAGGGTTTAGGGTTAGGGTTTAGGGTTTAGGGTTTAGGGTTTAGGGTTTAGGGTTTAGGGTTTAGGGTTAGGGTTTAGGGTTAGGGTTTAGGGTTTAGGGTTTAGGGTTTAGGGTTTAGGGTTTAGGGTTTAGGTTTAGGGTTTAGGGTTTAGGGTTTAGGGTTTAGGGTTTAGGGTTTAGGGTTTAGGGTTTAGGGTTTAGGGTTAGGGTTAGGGTTTAGGGTTTAGGGTTTAGGGTTTAGGGTTTAGGGTTTAGGGTTTAGGGTTTAGGGTTTAGGGTTTAGGGTTAGGGTTTAGGGTTTAGGGTTTAGGTTTAGGGTTTAGGGTTTAGGGTTAGGGTTTAGGGTTTAGGGTTTAGGGTTTAGGGTTAGGGTTAGGGTTTTAGGGTTTAGGGTTAGGGTTTAGGGTTTAGGGTTTAGGGTTTAGGGTTTAGGGTTTAGGGTTAGGGTTTAGGGTTTAGGGTTTAGGGTTTAGGGTTTAGGGTTTAGGGTTAGGGTTAGGGTTTAGGGTTTAGGGTTTAGGGTTTAGGGTTTAGGGTTAGGGTTTAGGGTTTAGGGTTTAGGGTTTAGGGTTTAGGGTTTAGGGTTTAGGGTTTAGGGTTTTAGGGTTTAGGGTTAGGGTTTAGGGTTTAGGGTTTAGGGTTTAGGGTTTAGGGTTTAGGGTTAGGGTTAGGGTTTAGGGTTTAGGGTTTTAGGGTTTAGGGTTTAGGGTTAGGGTTTAGGGTTTAGGGTTTAGGGTTTAGGGTTTAGGGTTTAGGGTTTAGGGTTTAGGGTTTAGGGTTAGGGTTTTGGGTTTAGGGTTTAGGGTTTAGGGTTTAGGGTTTAGGGTTTAGGGTTTAGGGTTTAGGGTTTAGGGTTTAGGGTTAGGGTTTAGGGTTTAGGGTTTAGGGTTTAGGGTTTAGGGTTTAGGGTTTAGGGTTTAGGGTTTAGGGTTTAGGGTTTAGGGTTTAGGGTTTAGGGTTTAGGGTTAGGGTTTAGGGTTAGGGTTTAGGGTTAGGGTTTAGGTTTAGGGTTTAGGGTTAGGGTTAGGGTTTAGGGTTTAGGGTTTAGGGTTTAGGGTTTAGGGTTTAGGGTTAGGGTTTAGGGTTTAGGGTTTAGGGTTTAGGGTTTAGGGTTTAGGGTTTAGGGTTTAGGGTTTAGGGTTTAGGGTTTAGGGTTTAGGTTAGGGTTTAGGGTTTAGGGTTTAGGGTTAGGGTTTAGGGTTAGGGTTTAGGGTTTAGGGTTTAGGGTTTAGGGTTTAGGGTTTAGGGTTTAGGGTTAGGGTTGGTTTAGGGTTTAGGGTTTAGGGTTTAGGGTTTAGGGTTTAGGGTTTAGGGTTTAGGGTTAGGGTTAGGGTTTAGGGTTTAGGGTTGTAGGGTTTAGGGTTTAGGGTTTAGGGTTTAGGGTTTAGGGTTTAGGGTTTAGGGTTTAGGGTTTAGGGTTTAGGGTTTAGGGTTTTAGGGTTTAGGGTTTAGGGTTAGGGTTTAGGGTTTAGGGTTTAGGGTTTAGGGTTTAGGGTTTAGGGTTTAGGGTTAGGGTTAGGGTTTAGGGTTTAGGGTTAGGGTTTAGGGTTTAGGGTTTAGGGTTTAGGGTTTAGGGTTTAGGGTTTAGGGTTTAGGGTTTAGGGTTTAGGGTTTAGGGTTTAGGGTTTAGGGTTTAGGGTTTAGGGTTTAGGGTTTAGGGGTTTAGGTTTAGGGTTTAGGGTTTAGGGTTTAGGGTTTAGGGTTTAGGGTTTAGGGTTTAGGGTTTAGGGTTTAGGGTTTAGGGTTTAGGGTTTAGGGTTTAGGGTTTAGGTTAGGGTTTAGGGTTTAGGGTTAGGGTTAGGGTTAGGGTTTAGGGTTTAGGGTTTAGGGTTTAGGGTTTAGGGTTTTAGGGTTTAGGGTTTAGGGTTTAGGGTTTAGGGTTTAGGGTTTAGGGTTTAGGGTTTAGGGTTAGGGTTTAGGGTTAGGGTTTAGGTTTAGGGGTTTAGGGTTTAGGGTTTAGGGTTAGGGTTTAGGGTTTAGGGTTTAGGGTTTAGGGTTAGGGTTTTAGGGTTTAGGGTTTTAGGGTTTTAGGGTTTAGGGTTTAGGGTTTTAGGGTTTTAGGGTTTAGGGTTTAGGGTTTAGGGTTTTAGGGTTTTAGGGTTTAGGGTTTAGGTTTAGGGTTTAGGGTTTAGGTTTAGGGTTTAGGGTTTAGGGTTGTTAGGGTTTAGGGTTAGGGTTTAGGGTTTAGGGTTAGGGTTTTAGGGTTTAGGGTTTTGGGTTTTAGGGTTTTAGGGTTTAGGGTTAGGGTTAGGGTTTAGGGTTTAGGGTTAGGGTTTTAGGGTTTAGGGTTTAGGGTTAGGGTTTAGGGTTTAGGGTTTAGGGTTTAAGGGTTAGGGTTAGGGTTAGGGTTAGGGTTTAGGGTTTAGGGTTTAGGGTTTAGGGTTTAGGGTTTAGGGTTTAGGGTTTAGGGTTTAGGGTTTAGGGTTTAGGGTTTAGGGTTTAGGGTTTAGGGTTAGGGTTAGGGTTTAGGGTTTAGGGTTTAGGGTTTAGGGGTTTAGGGTTTAGGGTTTAGGTTTAGGGTTTAGGGTTTAGGGTTAGGGTTTAGGGTTAGGGTTTAGGGTTAGGGTTAGGGTTTAGGGTTAGGGTTTAGGGTTTAGGGTTAGGGTTAGGGTTTAGGGTTAGGGTTTTAGGGTTTAGGGTTTAGGGTTTAGGGTTTAGGGTTTAGGGTTTAGGGTTTAGGGTTTAGGGTTAGGTTAGGGTTTAGGGTTAGGGTTTAGGGTTAGGGTTTAGGGTTTAGGGTTTAGGGTTTAGGGTTTAGGGTTTAGGGTTTAGGGTTTAGGGTTTAGGGTTTAGGGTTAGGGTTTAGGGTTTAGGGTTTAGGGTTAGGGTTTAGGGTTTAGGGTTAGGGTTTAGGGTTAGGGTTAGGGTTTAGGGTTTAGGGTTTAGGGTTTAGGGTTTTAGGGTTTAGGGTTTAGGGTTTAGGGTTAGGGTTTAGGGTTTAGGGTTTAGGGTTTAGGGTTTAGGGTTTAGGGTTTAGGGTTTAGGGTTTAGGGTTTAGGGTTTAGGGTTTAGGGTTAGGGTTTAGGGTTTAGGGTTTAGGGTTTAGGGTTTAGGGTTAGGGTTTAGGGTTAGGGTTTAGGGTTTAGGGTTTAGGGTTTAGGGTTTAGGGTTTTAGGGTTTAGGGTTTAGGGTTTAGGGTTTAGGGTTAGGGTTTAGGGTTTAGGGTTTAGGGTTTAGGGTTTAGGGTTAGGGTTTAGGGTTTAGGGTTTAGGGTTTAGGGTTTAGGGTTTAGGGTTTAGGGTTAGGGTTAGGGTTAGGGTTTAGGGTTTAGGGTTTAGGGTTTAGGGTTTTAGGGTTTAGGGTTTAGGGTTTAGGGTTAGGGTTTAGGGTTTAGGGTTTAGGGTTTAGGGTTTAGGGTTTAGGGTTTAGGGTTTAGGGTTTAGGGTTTAGGGTTTAGGGTTTAGGGTTGTTTAGGGTTTAGGGTTTAGGGTTTAGGGTTAGGGTTTAGGGTTTAGGGTTTAGGGTTAGGGTTAGGGTTTAGGGTTTAGGGTTTAGGGTTTAGGGTTTAGGGTTAGGGTTAGGGTTTAGGGTTTAGGGTTTAGGGTTTAGGGTTTAGGGTTTAGGGTTTAGGGGTTTAGGGTTTAGGGTTTAGGGTTTAGGGTTAGGGTTTAGGGTTTAGGGGTTTAGGGTTTAGGGGTTAGGGTTTAGGGTTTAGGGTTTAGGGTTTAGGGTTAGGGTTTAGGGTTTAGGGTTTAGGGTTTAGGGTTTAGGGTTTAGGGTTTAGGGTTAGGGTTTAGGGTTTAGGGTTTAGGGTTTAGGGTTTAGGGTTAGGGTTTAGGGTTAGGGTTTAGGGTTAGGTTTAGGGTTTAGGGTTTAGGGTTTAGGGTTTAGGGTTTAGGGTTTAGGGTTAGGGTTTAGGGTTTAGGGTTTAGGGTTTAGGGTTTAGGGTTTAGGGTTTAGGGTTTAGGGTTAGGGTTTAGGGTTTAGGGTTTAGGGTTTAGGGGTTTAGGGTTTAGGGTTTAGGGTAGGTTTAGGGTTAGGGTTTAGGGTTTAGGGTTTAGGGTTAGGGTTTAGGGTTTAGGGTTTAGGGTTTAGGGTTAGGGTTTAGGGTTTAGGGTTTAGGGTTTAGGGTTTAGGGTTTAGGGTTAGGGTTAGGGTTTAGGGTTTAGGGTTAGGGTTAGGGTTTAGGGTTTAGGGTTTAGGGTTAGGGTTTAGGGTTTAGGGTTTAGGGTTTAGGGTTTAGGGTTTAGGGTTAGGGTTTAGGGTTTAGGGTTTAGGGTTTAGGGTTTAGGGTTAGGGTTTAGGGTTTAGGGTTTAGGGTTTAGGGTTTAGGGTTTAGGGTTTAGGGTTAGGGTTTAGGGTTTAGGGTTTAGGGTTAGGGTTAGGGTTTAGGGTTTAGGGTTTAGGGTTTAGGGTTTAGGGTTTAGGGTTAGGGTTTTAGGGTTTAGGGTTTAGGGTTTAGGGTTTAGGGTTTAGGGTTTAGGGTTTAGGGTTTAGGGTTAGGGTTTAGGGTTTAGGGTTTAGGGTGTAGGGTTAGGGTTTAGGGTTTAGGGTTTAGGGTTTAGGGTTTAGGGTTTGGGTTTAGGGTTTAGGGTTTAGGGTTTAGGGTTTAGGGTTTAGGGTTAGGGTTTAGGGTTTAGGGTTTAGGGTTTAGGGTTTAGGGTTTAGGGTTTAGGGTTTAGGGTTAGGGTTTAGGGTTTAGGGTTTAGGGTTTAGGGTTTTAGGGTTTAGGGTTTAGGGTTTAGGGTTTAGGGTTAGGGTTTGGTAGGGTTTAGGGTTGGTTAGGGTTTAGGGTTTAGGTTTAGGGTTTAGGGTTTAGGGTTTAGGGTTTAGGGTTTAGGGTTTAGGGTTTAGGGTTTAGGGTTAGGGTTTAGGGTTTAGGGTTTAGGGTTTAGGGTTTAGGGTTTAGGGTTTAGGGTTTTAGGGTTTAGGGTTTAGGGTTTAGGGTTTAGGGTTTAGGGTTTAGGGTTAGGGTTTAGGGTTTAGGGGTTAGGGTTTAGGGTTAGGGTTTAGGGTTTAGGGTTTAGGGTTTAGGGTTTAGGGTTTAGGGTTAGGGTTTAGGGTTTAGGGTTTAGGGTTTAGGGTTGTTTAGGGTTTAGGGTTAGGGTTAGGGTTTAGGGTTTAGGGTTTAGGGTTTAGGGTTTAGGGTTTAGGGTTTAGGGTTTAGGGTTTAGGGTTTAGGGTTTAGGGTTTAGGGTTTAGGGTTAGGGTTTAGGGTTTAGGGTTTAGGGTTAGGGTTTAGGGTTTAGGGTTAGGGTTTAGGGTTAGGGTTTAGGGTTTAGGGTTTAGGGTTTAGGGTTTAGGGTTTAGGGTTTAGGTGGTTTAGGGTTTAGGGTTTAGGGTTTAGGGTTAGGGTTTAGGGTTTAGGGTTAGGGTTTAGGGTTAGGTTAGGGTTTAGGGTTTAGGGTTTAGGGTTTAGGGTTTAGGGTTTAGGGTTTAGGGTTTAGGTTTAGGGTTTAGGGTTTAGGGTTTAGGGTTTAGGGTTTTAGGGTTAGGGTTTAGGGTTAGGGTTTAGGGTTTAGGGTTTAGGGTTTAGGGTTTAGGGTTTAGGGTTTAGGGTTTAGGGTTTAGGGTTTAGGGTTAGGGTTTAGGGTTTAGGGTTTAGGGGTTTAGGGTTTAGGGTTTAGGGTTTAGGGGTTTAGGGGTTTTAGGGTTTAGGGTTAGGGTTTAGGGTTTAGGGTTTAGGGTTTAGGGTTAGGGTTTAGGGTTTAGGGTTAGGGTTTAGGGTTTAGGGTTTAGGGTTTAGGGTTAGGGTTTAGGGTTTAGGGTTAGGTTAGGGTTTAGGGTTAGGGTTTAGGGTTAGGGTTTAGGGTTTAGGGTTTAGGGTTTAGGGTTTAGGGTTTAGGGTTTAGGGTTTAGGGTTTAGGGTTAGGGTTTAGGGTTTAGGTTTAGGGTTTAGGGTTTAGGGTTTAGGGTTTAGGGTTTAGGGTTTAGGGTTTAGGGTTTAGGGTTTAGGGTTTAGGGTTTAGGGTTTAGGGTTTAGGGTTTAGGGTTTAGGGTTTAGGGTTTAGGGTTAGGGTTTAGGGTTTAGGGTTTAGGGTTTAGGGTTAGGGTTTAGGGTTTAGGGTTTAGGGGTTTAGGGTTTAGGTTTAGGGTTTAGGGTTTAGGGTTTAGGGTTTAGGGTTTAGGGTTTAGGGTTTAGGGTTTAGGGTTTAGGGTTTAGGGTTTAGGGTTTAGGGTTAGGGTTTAGGGTTAGGGTTTAGGGTTTAGGGTTAGGGTTTAGGGTTTAGGGTTTAGGGTTTAGGGTTTAGGGTTTAGGGTTTAGGGTTTAGGGTTTAGGGTTTAGGGTTTAGGGTTTAGGGTTAGGGTTTAGGGTTTAGGGTTTAGGGTTTAGGGTTTAGGGGTTTAGGTTTTAGGGGTTTAGGGTTTAGGGTTTAGGGTTTAAGGGTTGGGTTTAGGGTTTAGGGTTTAGGGTTTAGGGTTTAGGGTTTAGGGTTTAGGGTTTAGGGTTTAGGGTTAGGGTTTAGGTTTTGGGTTAGGGTTTAGGGTTTAGGGTTTAGGGTTTAGGGTTTAGGGTTTAGGGTTAGGGTTTAGGGTTTAGGGTTTAGGGTTTAGGGTTTAGGGTTTAGGGTTTAGGGTTTAGGGTTAGGGTTTAGGGTTTAGGGTTTAGGGTTTTAGGGTTTAGGGTTTAGGGTTTAGGGTTTAGGGTTTAGGGTTTAGGTTTAGGGTTTAGGGTTAGGGTTTAGGGTTTAGGGTTTAGGGTTTAGGGTTTAGGGTTTAGGGTTTAGGGTTTAGGGTTAGGGTTTAGGGTTTAGGGTTTAGGGTTTAGGGTTTAGGGTTTAGGGTTTAGGGTTAGGGTTTAGGGTTTAGGGTTTAGGGTTTAGGGTTTAGGGTTTAGGGTTAGGGTTAGGGTTTAGGGTTTAGGGTTTAGGGTTAGGGTTTAGGGTTTAGGGTTTAGGGTTTAGGGTTTAGGGTTTAGGGTTTAGGGTTTAGGGTTAGGGTTTAGGGTTTAGGGTTTAGGGTTAGGGTTTAGGGTTTAGGGTTTAGGGTTTAGGGTTTAGGGTTTAGGGTTTAGGTTTAGGGTTTAGGGTTTAGGGTTTAGGGTTAGGGTTTAGGGTTTAGGGTTTAGGGTTTAGGGTTTAGGGTTAGGGTTTAGGGTTTAGGGTTTAGGGTTAGGGTTAGGGTTAGGGTTTAGGGTTTAGGGTTTAGGGTTAGGGTTTAGGGTTTAGGGTTTAGGGTTTAGGGTTTAGGGTTTAGGGTTTAGGGTTTAGGGTTTAGGGTTTAGGGTTTAGGGTTTAGGGTTTAGGGTTTAGGGTTTAGGGTTTAGGGTTTAGGGTTTAGGGTTTAGGGTTAGGGTTAGGGTTTTAGGGTTTAGGGTTTAGGGTTTAGGGTTTAGGGTTTAGGGTTAGGTTTAGGGTTAGGTTTAGGGTTTAGGGTTTTGGGTTAGGGTTAGGGTTTAGGGTTTAGGGTTTAGGGTTTAGGGTTTTAGGGTTTAGGGTTGGTTTAGGGTTTAAGGGTTTAGGGTTTAGGGTTTAGGGTTTAGGGTTTAGGGTTAGGGTTTAGGGTTTAGGGTTAGGGTTTAGGGTTTAGGGTTTAGGGTTTAGGGTTTAGGGTTTAGGGTTTAGGGTTTAGGGTTAGGGGTTTAGGGTTTTAGGGTTTAGGGTTAGGGTTTAGGGTTTAGGGTTAGGGTTTAGGGTTTAGGGTTAGGGTTTAGGGTTTAGGGTTTAGGGTTTAGGGTTAGGGTTTAGGGTTTAGGGTTTAGGGTTTAGGGTTTTAGGGTTGGGTTTAGGGTTTAGGGTTTAGGGTTTAGGGTTTAGGGTTTAGGGTTTAGGGTTTAGGGTTTAGGGTTAGGGTTTAGGGTTTTAGGGTTAGGGTTTAGGGTTTAGGGTTTAGGGTTTAGGGTTTAGGGTTTAGGGTTTAGGGTTTAGGGTTTAGGGTTTAGGGTTTAGGGTTTAGGGTTTTAGGGTTTAGGGTTTAGGGTTTAGGGTTTAGGGTTTAGGGTTTAGGGTTTAGGGTTAGGGTTAGGGTTTAGGGTTTAGGGTTTAGGGTTTAGGGTTTAGGGTTTAGGTAGGGTTTAGGGTTAGGTTTAGGGTTTAGGGTTTAGGGTTAGGGTTTAGGGTTTAGGGTTTAGGGTTAGGGGTTTAGGGTTTAGGGTTTAGGGTTGGTTTAGGGTTTAGGGTTTAGGGTTTAGGGTTAGGGTTTAGGGTTTAGGGTTAGGGTTTAGGGTTTAGGGTTTAGGGTTTAGGGTTAGGGTTTAGGGTTTAGGGTTTAGGGTTTAGGTTTTAGGGTTAGGGTTTAGGGTTTAAGGGTTTAGGGTTAGGGTTTAGGGTTAGGGTTTAGGGTTAGGGTTTAGGGTTTAGGGTTTAGGGTTTAGGGTTTAGGGTTTAGGGTTTAGGGTTTAGGGTTTAGGGTTTAGGGTTTAGGGTTTAGGGTTTAGGGTTTAGGGTTAGGGTTTAGGGTTTAGGGTTTAGGGTTAGGGTTTAGGGTTTAGGGTTTTAGGGTTTAGGGTTTTGGGTTTAGGGGTTTAGGGTTTAGGGTTTAGGGTTTAGGGTTTAGGGTTTAGGGTTTAGGGTTTAGGGTTAGGGTTAGGGTTTAGGGTTTAGGGTTTAGGGTTTAGGGTTTAGGGTTAGGGTTTAGGGTTTAGGGTTTAGGGTTTAGGGTTTAGGGTTTAGGGTTTAGGGTTTAGGGTTTAGGGTTTAGGGTTAGGGTTAGGGTTTAGGGTTTAGGGTTTAGGGTTTAGGGTTTAGGGTTAGGGTTTAGGGTTTAGGGTTTAGGGTTTAGGGTTAGGGTTAGGGTTTAGGGTTTAGGGTTTAGGGTTTAGGGTTTAGGGTTAGGGTTTAGGGTTAGGGTTTAGGGTTTAGGGTTTAGGGTTTAGGGTTAGGGTTTAGGGTTTAGGGTTTAGGGTTTAGGGTTTAGGGTTTAGGGTTTAGGGTTTAGGGTTTAGGGTTTAGGGTTTAGGGTTTAGGGTTTAGGGTTTAGGGTTTAGGGTTTAGGGTTTAGGGTTTTAGGGTTTAGGGTTTAGGGTTTAGGGTTTAGGGTTTAGGGTTTTAGGGTTTAGGGTTTAGGGTTAGGGTTTAGGGTTTAGGGTTTAGGGTTTAGGGTTTAGGGTTTAGGGTTTAGGGTTAGGGTTTAGGGTTTAGGGTTTAGGGTTTAGGGTTTTAGGGTTTAGGGTTTAGGGTTTAGGGTTTAGGGTTTAGGGTTTAGGGTTAGGGTTTAGGGTTTAGGGTTTAGGGTTTAGGGTTTAGGGTTTAGGGTTTAGGGTTAGGGTTTAGGGTTTAGGGTTTAGGGTTTAGGGTTTAGGGTTTAGGGTTTAGGGTTTTAGGGTTTAGGGTTTAGGTTTAGGGTTTAGGGTTTAGGGTTTAGGGTTTAGGGTTTAGGGTTAGGGTTTAGGGTTTAGGGTTTAGGGTTTAGGGTTTAGGGTTTAGGGTTTAGGGTTTAGGGTTTAGGGTTAGGGTTAGGGTTAGGGTTTAGGGTTTAGGGTTTAGGGTTTAGGGTTTAGGGTTTAGGGTTTAGGGTTTAGGGTTTAGGTTTAGGGTTTAGGGTTTAGGGTTAGGGTTTAGGGTTAGGGTTTAGGGTTTAGGGTTTAGGGTTAGGGTTTAGGGTTAGGGTTTTAGGGTTTAGGGTTAGGGTTTAGGGTTTAGGGTTTAGGGTTTAGGGTTTTAGGGTTTTAGGGTTTAGGGTTTAGGTTTTAGGGTTTAGGGTTAGGGTTAGGGTTTAGGGTTTAGGGTTAGGGTTTAGGGTTTAGGGTTTAGGGTTTTAGGGTTTAGGGTTTAGGTTTAGGGTTTAGGGTTTAGGGTTTAGGGTTTAGGGTTTAGGGTTTAGGGTTTAGGGTTTAGGGTTTAGGGTTTAGGGTTTAGGGTTTAGGGTTTAGGGTTTAGGGTTTAGGTTTAGGGTTTAGGGTTTAGGGTTAGGGTTTAGGGTTAGGGTTTAGGTTTAGGGTTTAGGGTTTAGGGTTTAGGGTTTAGGGTTTAGGGTTTAGGGTTTAGGGTTTAGGGTTTAGGGTTAGGGTTTAGGGTTTAGGGTTTAGGGTTAGGGTTTAGGGTTTAGGGTTTAGGGTTTGGTTTAGGGTTTAGGGTTTAGGGTTTAGGTTTAGGGTTTAGGGTTTAGGGTTTAGGGTTTAGGGTTTAGGGTTTAGGGTTTAGGGGTTTAGGGTTTAGGGTTTAGGGTTTAGGGTTTAGGGTTTAGGGTTTAGGGTTAGGTTTAGGGTTTAGGTTTAGGGTTAGGGTTTAGGGTTTAGGGTTTAGGGTTTAGGGTTTAGGGTTTAGGGTTTAGGGTTTAGGGTTTAGGGTTTAGGGTTTTAGGGTTTAGGGTTTAGGGTTAGGGTTTAGGGTTAGGGTTTAGGGTTTAGGGTTTAGGGTTTAGGGTTTAGGGTTTAGGGTTTAGGGTTTAGGGTTTAGGGTTTAGGGTTTAGGGTTTAGGGTTTAGGGTTTTAGGGTTTTAGGGTTTAGGGTTTAGGGTTTAGGGTTTAGGGTTTAGGGTTTAGGGTTTAGGGTTTAGGGTTTAGGGTTTAGGGTTTAGGGTTTAGGGTTTAGGGTTTAGGGTTTAGGGTTTAGGGTTTAGGGTTTAGGGTTTAGGGTTTAGGGTTAGGGTTTAGGGTTTAGGGGTTTAGGGTTTAGGGTTTAGGGTTTAGGGTTTAGGGTTTAGGGTTTAGGGTTTAGGGTTTAGGGTTTAGGGTTTAGGGTTTAGGGTTTAGGGTTTAGGGTTTAGGGTTTAGGGTTTAGGGTTAGGGTTTAGGTTTAGGTTTAGGGTTTAGGGTTTAGGGTTTAGGGTTAGGGTTTAGGGTTTAGGGTTTAGGGTTTAGGGTTTAGGGTTTTAGGGTTTAGGGTTTAGGGTTTAGGGTTTAGGGTTTAGGGTTTAGGGTTTAGGGTTTAGGGTTTAGGGTTTAGGGTTTAGGGTTTAGGGTTTAGGGTTTAGGGTTTAGGGTTTAGGGTTTAGGGTTTAGGGTTTAGGGTTTAGGGTTTAGGGTTAGGGTTTAGGGTTTAGGGTTAGGGTTTAGGGTTTAGGGTTTAGGGTTTTAGGGTTAGGGTTTAGGGTTTAGGGTTTAGGGTTTAGGGTTAGGGTTTAGGGTTTAGGGTTTAGGGTTTTAGGGTTTAGGGTTAGGTTTAGGGTTTAGGGTTAGGGTTTAGGGTTTAGGGTTTAGGGTTTAGGGTTTAGGGTTTAGGGTTTAGGGTTTAGGGTTTAGGGTTTTAGGGTTTTAGGGTTTAGGTTTAGGGTTTTAGGGTTTTAGGGTTTAGGGTTTAGGGTTTAGGGTTTAGGGTTTAGGGTTTAGGGTTTAGGGTTTAGGGTTTAGGGTTTAGGGTTTAGGGTTTAGGGTTTAGGGTTTAGGGTTTAGGGTTTAGGGTTTAGGGTTTAGGGTTTAGGGTTTAGGGTTAGGGTTTAGGGTTTAGGGTTTAGGGTTAGGGTTTAGGGTTTAGGGTTTAGGGTTTAGGGGTTTAGGGTTTAGGGTTTAGGGTTTAGGGTTTAGGGTTTAGGGTTTAGGGTTTAGGGTTAGGGTTTAGGGTTTAGGGTTTAGGGTTTAGGGTTTAGGGGTTTAGGGTTTAGGGTTTAGGGTTTAGGGTTTAGGGTTTAGGGTTTAGGGTTTTAGGGTTTAGGGTTTAGGGTTAGGGTTTAGGGTTTAGGGTTTAGGGTTAGGGTTTTTAGGGTTTAGGGTTTAGGGTTTAGGGTTTAGGGTTTAGGGTTTAGGGTTTTAGGGTTTAGGGTTTAGGGTTTAGGGTTTAGGGTTTAGGGTTTAGGGTTTAGGGTTTAGGGTTTAGGGTTTAGGGTTTAGGGTTTAGGGTTAGGGTTTAGGGTTTAGGGTTTAGGGTTTAGGGTTTAGGGTTTAGGGTTAGGGTTAGGGTTTAGGGTTTAGGGTTTAGGGTTTAGGGTTTAGGGTTTAGGGTTAGGGTTTAGGGGTTTAGGGTTTAGGGTTTAGGGTTTAGGGTTGTAGGGTTTAGGGTTTAGGGTTTAGGGTTTAGGGTTTAGGGTTTAGGGTTTAGGGTTTAGGGTTTAGGGTTTAGGGTTTAGGGTTTAGGGTTTAGGGTTTAGGGTTTAGGGTTTAGGGTTTAGGGTTTAGGGTTAGGGTTTAGGGTTTAGGGTTTAGGGTTTAGGGTTTAGGGTTTAGGGTTTAGGGTTTAGGGTTTAGGGTTTAGGGTTTAGGGTTTAGGGTTTAGGGTTAGGGTTTAGGGTTTAGGGTTTAGGGTTTAGGGTTTAGGGTTTAGGGTTTAGGGTTTTAGGGTTTAGGGTTTAGGGTTTAGGGTTTAGGGTTTAGGGTTTAGGGTTTAGGGTTTAGGGTTTAGGGTTTAGGGTTTAGGGTTTTAGGGTTTAGGGTTTAGGGTTAGGGTTTAGGGTTTTAGGGTTTAGGGTTTAGGGTTTAGGGTTTAGGGTTTAGGGTTTAGGGTTTAGGGTTTAGGGTTTAGGGTTAGGGTTTAGGGTTTAGGGTTTAGGGTTTAGGGTTTTAGGGTTTAGGGTTTAGGGTTTAGGGTTTAGGGTTTAGGGTTTAGGGTTTAGGGTTTAGGGTTTAGGGTTTAGGGTTTAGGGTTTAGGGTTTAGGGTTTGGGTTTAGGGTTTAGGGTTTAGGGTTTAGGGTTAGGGTTTAGGGTTTAGGGTTTAGGGTTTAGGGTTTAGGGGTTAGGGTTTAGGGTTTAGGTTTAGGTTTAGGGTTTAGGGTTTAGGGTTTAGGGTTTAGGGTTTAGGGTTTAGGGTTTAGGGTTTAGGGTTTAGGGTTTAGGGTTTAGGGTTTAGGGTTTAGGGTTTAGGGTTTAGGGTTTAGGGTTTAGGGTTTAGGGTTAGGGTTTAGGGTTTAGGGTTTAGGGTTAGGGTTTAGGTTTAGGGTTTAGGGTTAGGGTTTAGGGTTTTAGGGTTTAGGGTTTAGGGTTTAGGGTTTAGGGTTTAGGGTTTAGGGTTTAGGGTTTAGGGTTTAGGGTTTAGGGTTAGGGTTTAGGGTTTAGGGTTTAGGGTTTAGGGTTTAGGGTTTAGGGTTAGGGTTTAGGGTTTAGGGTTTAGGGTTTAGGGTTTAGGGTTTAGGGTTTTAGGGTTTAGGGTTTAGGGTTTAGGGTTTAGGGTTAGGGTTTAGGGTTTAGGGTTTAGGGTTAGGGTTTAGGGTTTAGGGTTTAGGGTTTAGGGTTTAGGGTTAGGGTTTTAGGGTTTAGGGTTTAGGGTTTAGGGTTTAGGGTTTAGGGTTTAGGGTTAGGGTTTAGGGTTTAGGGTTTAGGGTTTAGGGTTTAGGGTTTAGGGTTTAGGGTTTAGGGTTTAGGGTTTAGGGTTTAGGGTTAGGGTTTAGGGTTTAGGGTTTAGGGGTTTAGGGTTTAGGGTTTAGGGTTTAGGGTTTAGGGTTTAGGGTTTAGGGTTAGGGTTAGGGTTTAGGGTTAGGGTTTAGGGTTTAGGGTTTAGGGTTTAGGGTTTAGGGTTTAGGGTTTAGGGTTTAGGGTTAGGGTTTAGGGTTTAGGGTTTAGGGTTTAGGGTTTAGGGTTTAGGGTTTAGGGTTTAGGGTTTAGGGTTTAGGGTTAGGGTTTAGGGTTTAGGGTTTAGGGTTTAGGGTTAGGGTTTAGGGTTTAGGGTTTAGGGTTAGGGTTTAGGGTTTAGGGTTTAGGGTTTAGGGTTAGGGTTTAGGGTTTTAGGGTTTAGGGTTTAGGGTTAGGGGTTTAGGGTTTAGGGTTTAGGGTTAGGGTTTAGGGTTTAGGGTTTAGGGTTAGGGTTTAGGGTTTAGGGTTTAGGGTTTAGGGTTTAGGGGTTAGGGTTTAGGGTTTAGGGTTTAGGGTTTAGGGTTAGGGTTTAGGGTTTAGGGTTAGGGTTAGGGTTTAGGGTTTAGGGTTTAGGGTTTAGGGTTTAGGGTTTAGGGTTTTAGGGTTTAGGGTTTAGGGTTAGGGTTTAGGGTTTAGGGTTTAGGGTTTAGGGTTTAGGGTTTAGGGTTTAGGGTTTAGGGTTAGGGTTTAGGGTTTAGGGTTTAGGGTTTAGGGTTTAGGGTTTAGGGTTTAGGGTTTAGGGTTTAGGGTTTAGGGTTTAGGGTTTAGGGTTAGGTGGTTTAGGGTTTAGGGTTTTAGGTTTAGGGTTTAGGGTTTAGGGTTTAGGGTTTAGGGGTTTAGGGGTTAGGGTTTAGGGTTTAGGGTTAGGTTTAGGGTTTAGGGTTTAGGGTTTAGGGGTTAGGGTTTAGGGTTTAGGGTTTAGGGTTAGGGTTTAGGGTTTAGGGTTGGTTAGGGTTTAGGGTTTAGGGTTTAGGGTTAGGGGTTTAGGGTTTGGGTTTAGGGTTTAGGGTTTAGGGTTTAGGGTTTAGGGTTTAGGGTTTAGGGTTAGGGTTTAGGGTTTAGGGTTTAGGGTTTAGGGTTTAGGGTTAGGGTTAGGGTTTAGGGTTTAGGGTTTAGGGTTTAGGGTTTAGGTTTAGGGTTTAGGGTTTAGGGTTTAGGGTTTAGGGT

**2DL**

TTGGGTTTATGG**TTTAGGG**TTTAGGGTTAGGGTTTATGGATTAGGGTTTAGGGTTAGGGTTTATGGTTTAGGGTTTAGGGTTTAGGGTTAGGGTTTAGGGTTTAGGGTTTAGGGTTTAGGGTTTAGGGTTTAGGGTTTAGGGTTTAGGGTTAGGGTTTTAGGGTTTAGGGTTTAGGGTTTAGGGTTTAGGGTTTAGGGTTTAGGGTTTAGGGTTTAGGGTTTAGGGTTTAGGGTTTAGGGTTAGGGTTAGGGTTTAGGGTTTAGGGTTAGGGTTTAGGGGTTTAGGGTTTAGGGTTAGGGTTTAGGGTTAGGGTTTAGGGTTTAGGGTTTAGGGTTTAGGGTTTAGGGTTTAGGGTTTAGGGTTTAGGGTTAGGGTTAGGGTTTAGGGTTAGGGTTAGGGTTTAGGGTTTAGGGTTTAGGGTTTAGGGTTTAGGGTTTAGGGTTTAGGGTTTAGGGTTTAGGGTTTAGGGTTTAGGGTTTAGGGTTTAGGGTTTAGGGTTTAGGGTTTAGGGTTTAGGGTTTAGGGTTTAGGGTTTAGGGTTTAGGGTTTAGGGTTTAGGGTTTAGGGTTTAGGGTTTAGGGTTTAGGGTTTAGGGTTTAGGGTTTAGGGTTTAGGGTTTAGGGTTTAGGGTTTAGGGTTTAGGGTTTAGGGTTTAGGGTTTAGGGTTTAGGGTTTAGGGTTTAGGGTTTAGGGTTTAGGGTTTAGGGTTTAGGGTTTAGGGTTTAGGGTTTAGGGTTTAGGGTTTAGGGTTTAGGGTTTAGGGTTTAGGGTTTAGGGTTTAGGGTTTAGGGTTTAGGGTTTAGGGTTTAGGGTTTAGGGTTTAGGGTTTAGGGTTTAGGGTTTAGGGTTTAGGGTTTAGGGTTTAGGGTTTAGGGTTTAGGGTTTAGGGTTTAGGGTTTAGGGTTTAGGGTTTAGGGTTTAGGGTTTAGGGTTTAGGGTTTAGGGTTTAGGGTTTAGGGTTTAGGGTTTAGGGTTTAGGGTTTAGGGTTTAGGGTTTAGGGTTTAGGGTTTAGGGTTTAGGGTTTAGGGTTTAGGGTTTAGGGTTTAGGGTTTAGGGTTTAGGGTTTAGGGTTTAGGGTTTAGGGTTTAGGGTTTAGGGTTTAGGGTTTAGGGTTTAGGGTTTAGGGTTTAGGGTTTAGGGTTTAGGGTTTAGGGTTTAGGGTTTAGGGTTTAGGGTTTAGGGTTTAGGGTTTAGGGTTTAGGGTTTAGGGTTTAGGGTTTAGGGTTTAGGGTTTAGGGTTTAGGGTTTAGGGTTTAGGGTTTAGGGTTTAGGGTTTAGGGTTTAGGGTTTAGGGTTTAGGGTTTAGGGTTTAGGGTTTAGGGTTTAGGGTTTAGGGTTTAGGGTTTAGGGTTTAGGGTTTAGGGTTTAGGGTTTAGGGTTTAGGGTTTAGGGTTTAGGGTTTAGGGTTTAGGGTTTAGGGTTTAGGGTTTAGGGTTTAGGGTTTAGGGTTTAGGGTTTAGGGTTTAGGGTTTAGGGTTTAGGGTTTAGGGTTTAGGGTTTAGGGTTTAGGGTTTAGGGTTTAGGGTTTAGGGTTTAGGGTTTAGGGTTTAGGGTTTAGGGTTTAGGGTTTAGGGTTTAGGGTTTAGGGTTTAGGGTTTAGGGTTTAGGGTTTAGGGTTTAGGGTTTAGGGTTTAGGGTTTAGGGTTTAGGGTTTAGGGTTTAGGGTTTAGGGTTTAGGGTTTAGGGTTTAGGGTTTAGGGTTTAGGGTTTAGGGTTTAGGGTTTAGGGTTTAGGGTTTAGGGTTTAGGGTTTAGGGTTTAGGGTTTAGGGTTTAGGGTTTAGGGTTTAGGGTTTAGGGTTTAGGGTTTAGGGTTTAGGGTTTAGGGTTTAGGGTTTAGGGTTTAGGGTTTAGGGTTTAGGGTTTAGGGTTTAGGGTTTAGGGTTTAGGGTTTAGGGTTTAGGGTTTAGGGTTTAGGGTTTAGGGTTTAGGGTTTAGGGTTTAGGGTTTAGGGTTAGGGTTTAGGGTTAGGGTTTAGGGTTTAGGGTTAGGGTTTAGGGTTTAGGGTTTAGGGTTTAGTTTAGGGTTTAGGGTTTAGGGTTTAGGGTTTAGGGTTTAGGGTTTAGGGTTTAGGGTTTAGGGTTTAGGGTTTAGGGTTTAGGGTTTAGGGTTTAGGGTTTAGGGTTTAGGGTTTAGGGTTTAGGGTTTAGGGTTTAGGGTTTAGGGTTTAGGGTTAGGGTTTAGGGTTTAGGGTTTAGGGTTTAGGGTTTAGGGTTTAGGGTTTAGGGTTTAGGGTTTAGGGTTTAGGGTTTAGGGTTTAGGGTTGGTTAGGGTTTAGGGTTTAGGGTTTAGGGTTTAGGGTTTAGGGTTTAGGGTTTAGGGTTTAGGGTTTAGGGTTTAGGGTTTAGGGTTTAGGGTTTAGGGTTTAGGGTTTAGGGTTTAGGTAGGTTTAGGGTTTAGGGTTTAGGGTTTAGGGTTTAGGGTTAGGGTTTAGGGTTTTAGGGTTTAGGGTTTAGGTGGTTTAGGGTTTAGGGTTTAGGGTTAGGGTTGGTTTAGGGTTAGGGTTTAGGGTTTTAGGGTTTAGGGTTAGGGTTTAGGGTTTAGGGTTTAGGGTTAGGGTTTAGGGTTTAGGGTTAGGGTTTAGGGTTAGGGTTTAGGGTTTAGGGTTTAGGGTTTAGGGTTTTGGGTTTTTAGGGTTTAGGGTAGGTTAGGGTTAGGGTTAGGGTTTAGGGTTTAGGGTTTAGGGTTAGGGTTTAGGGTTTAGGGTTTAGGGTTTAGGGTTTAGGGTTTAGGGTTTAGGGTTTAGGGTTAGGGTTTAGGGTTTAGGGTTAGGGTTTAGGGTTTAGGGTTTAGGGTTTAGGGTTTAGGGTTTAGGGTTTAGGGTTTAGGGTTTAGGGTTTAGGGTTTAGGTTAGGGTTTAGGGTTTAGGGTTAGGGTTGTAGGGTTTAGGGTTAGGGTTAGGGTTTAGGGTTTAGGGTTTAGGGTTTAGGGTTAGGGTTTAGGGTTTAGGGTTTAGGGTTAGGGTTTAGGGTTTAGGGTTTAGGGTTAGGGTTTAGGGTTTAGGGTTTAGGGTTAGGTTTAGGGTTTAGGGTTTTAGGTTTAGGGTTTAGGGTTTAGGGTTTAGGGGTTAGGGTTTAGGGTTTAGGGTTTAGGGTTTAGGGTTTAGGGTTTAGGTTAGGGTTTAGGGTTTAGGGTTTAGGGTTAGGGTTTAGGGTTTAGGGTTTAGGTTTAGGGTTTAGGGTTAGGTTAGGGTTTAGGGTTTAGGGTTTAGGGTTTAGGGTTTAGGGTTTAGGGTGTTAGGGTTTAGGGTTTAGGGTTTAGGTTTAGGGTTTAGGGTTTAGGGTTTAGGGTTTAGGGTTTAGGGTTTAGGGTTTAGGGTTTAGGGTTTAGGGTTTAGGGTTTAGGGTTTAGGGTTTAGGGTTTAGGGTTTAGGGTTTAGGGTAGTTTAGGGTTAGGGTTAGGGTTTAGGTTTAGGGTTTAGGGTTTAGGGTTTAGGGTTTAGGGTTATAGGGTTTAGGGTTTAGGGTTTAGGGTTTAAGGGTTTAGGGTTTAGGGGTTTAGGGTTTAGGGTTTAGGGTTAGGTTTTAGGGTTTAGGGTTTAGGGTTTAGGGTTTAGGGTTTTAGGTTTTAGGTTTTAGGGTTTAGGGTTTAGGGTTTAGGGTTTAGGGTTTAGGGTTTAGGTTTAGGGTTTAGGGTTTAGGGTTTAGGGTTAGGGTTTAGGGTTTAGGGTTTAGGGTTTAGGGTTTAGGGTTTAGGGTTTAGGTTTAGGGTTTAGGGTTAGGGTTTAGGGTTTAGGGTTTAGGGTTAGGGTTTAGGGTTTAGGGTTTAGGGTTTAGGGTTTAGGGTTTAGGGTTTAGGGTTTAGGGTTTAGGTTTAGGGTTTAGGGTTTAGGGTTTAGGGTTTAGGGTTTAGGTTTAGGGTTTAGGGTTTAGGGTTTAGGGTTTAGGGTTTAGGGTTAGGGTTTAGGGTTAGGGTTTAGGGTTTAGGGTTTAGGGTTTAGGGTTTAGGGTTTAGGGTTTAGGGTTTAGGGTTTAGGGTTTAGGGTTTAGGGTTTAGGGTTTAGGGTTTAGGGTTTAGGGTTTAGGGTTTAGGGTTTAGGGTTTAGGGTTTAGGGTTTAGGGTTTAGGGTTTAGGGTTTTAGGGTTTAGGGTTAGGGTTTAGGGTTTAGGGTTGGTTAGGGTTTAGGGTTTAGGGTTTAGGGTTTAGGGTTTAGGGTTTAGGGTTTAGGGTTTAGGGTTTAGGGTTTAGGGTTTAGGGTTTAGGGTTTAGGGTTTAGGGTTTAGGGTTTAGGGTTTAGGGTTTAGGGTTTAGGGTTTAGGGTTTAGGGTTAGGGTTTAGGGTTTAGGGTTTAGGGTTTAGGGTTTAGGGTTTAGGGTTTAGGGTTTAGGGTTTAGGTTTAGGGTTTAGGGTTTAGGGTTTAGGGTTTAGGGTTAGGTTTAGGGTTTAGGGTTTAGGGTTTAGGGTTTAGGGTTTAGGGTTTAGGGTTTAGGGTTTAGGGTTTAGGGTTTAGGGTTTAGGGTTTAGGGTTTAGGGTTTAGGGTTTAGGGTTTAGGGTTTAGGGTTTAGGGTTTAGGGTTTAGGGTTTAGGGTTAGGGTTTAGGGTTTAGGGTTTAGGGTTTAGGGTTTAGGGTTTAGGGTTTAGGGTTTAGGGTTTAGGGTTTAGGGTTTTAGGGTTTAGGGTTTAGGGTTTAGGGTTAGGGTTTAGGGTTAGGGTTTAGGGTTTAGGGTTTAGGGTTTAGGGTTTAGGGTTTAGGGTTTAGGGTTTAGGGTTTAGGGTTTAGGTTTAGGGTTTAGGGTTTAGGGTTTAGGGTTTAGGGTTTAGGGTTTAGGGTTTAGGGTTTAGGGTTTAGGGTTTAGGGTTTAGGGTTTAGGGTTTAGGGTTTAGGGTTTAGGGTTTAGGGTTTAGGGTTTAGGGTTTAGGGTTTAGGGTTTAGGGTTTAGGGTTTAGGGTTTAGGGTTTAGGGTTTAGGGTTTAGGGTTTAGGGTTTAGGGTTTAGGGTTTAGGGTTTAGGGTTTAGGGTTAGGGTTTAGGGTTTAGGGTTTAGGGTTTAGGGTTTAGGGTTTAGGGTTTAGGGTTTAGGGTTTAGGGTTTAGGGTTTAGGGTTTAGGGTTTAGGGTTTAGGGTTTAGGGTTTAGGGTTTAGGGTTTAGGGTTTAGGGTTTAGGGTTTAGGGTTTAGGGTTTAGGGTTTAGGGTTTAGGGTTTAGGGTTTAGGTTTAGGGTTTAGGGTTTAGGGTTTAGGGTTAGGGTTTAGGGTTTAGGGTTTAGGGTTTAGGGTTTAGGGTTTAGGTTTAGGGTTTAGGGTTTAGGGTTTAGGGTTTAGGGTTTAGGGTTTAGGTTTAGGGTTTAGGGTTTAGGGTTTAGGGTTTAGGGTTTAGGGTTTAGGGTTTAGGGTTTAGGTTTAGGGTTTAGGGTTTAGGGTTTAGGGTTTAGGGTTTAGGGTTTAGGGTTTAGGGTTTAGGGTTTAGGGTTTAGGGTTTAGGGTTTAGGGTTTAGGGTTTAGGGTTTAGGGTTTAGGGTTTAGGGTTTAGGGTTTAGGGTTTAGGGTTTAGGGTTTAGGGTTTAGGGTTTAGGGTTTAGGGTTTAGGGTTTAGGGTTTAGGGTTTAGGGTTTAGGGTTTAGGGTTTAGGGTTTAGGGTTTAGGGTTTAGGGTTTAGGGTTTAGGGTTTTAGGGTTTAGGGTTTAGGGTTTAGGGTTTAGGGTTTAGGGTTTAGGGTTTAGGGTTTAGGGTTTAGGGTTTAGGGTTTAGGGTTAGGGTTTAGGGTTTAGGGTTTAGGGTTTAGGGTTTAGGGTTTAGGGTTTAGGGTTTAGGGTTTAGGGTTTAGGGTTTAGGGTTTAGGGTTTAGGGTTTAGGGTTTAGGGTTTAGGGTTTAGGGTTTAGGGTTTAGGGTTTAGGGTTTAGGGTTTAGGGTTTAGGGTTTAGGGTTTAGGGTTTAGGGTTTAGGTTTAGGGTTTAGGGTTTAGGGTTTAGGGTTTAGGGTTTAGGGTTTAGGGTTTAGGGTTTAGGGTTTAGGGTTTAGGGTTTAGGGTTTAGGGTTTAGGGTTAGGGTTTAGGGTTTAGGGTTTAGGGTTTAGGGTTTAGGGTTTAGGGTTTAGGGTTTAGGGTTTAGGGTTAGGGTTTAGGGTTTAGGGTTTAGGGTTTAGGTTTAGGGTTTTAGGGTTTAGGGTTTAGGGTTTAGGGTTTAGGGTTTAGGGTTTAGGGTTTAGGGTTTAGGGTTAGGGTTTAGGGTTTAGGGTTTAGGGTTTAGGGTTTAGGGTTTAGGGTTTAGGTTTAGGGTTTAGGGTTTAGGGTTTAGGGTTTAGGGTTTAGGGTTTAGGGTTTAGGGTTTAGGGTTTAGGGTTTAGGGTTTAGGGTTTAGGGTTTAGGGTTTAGGGTTTAGGGTTTAGGGTTTAGGGTTTAGGGTTTAGGGTTTAGGGTTTAGGTTTAGGGTTTAGGGTTTAGGGTTTAGGGTTTAGGGTTTAGGGTTTAGGGTTTAGGGTTTAGGGTTTAGGGTTTAGGGTTTAGGGTTTAGGGTTTAGGGTTTAGGTTAGGGTTAGGGTTTAGGGTTTAGGGTTTAGGGTTTAGGGTTTAGGGTTTAGGGTTAGGGTTTAGGGTTTAGGGTTTAGGGTTTTAGGGTTTAGGGTTTAGGGTTTAGGGTTTAGGGTTTAGGGTTTAGGGTTTAGGGTTTAGGGTTAGGGTTTAGGGTTTAGGGTTTAGGGTTTAGGGTTTAGGGTTTAGGGTTTAGGGTTTAGGGTTTAGGGTTTAGGGTTTAGGGTTTAGGGTTTAGGGTTTAGGGTTTAGGGTTAGGGTTTAGGGTTTAGGGTTTAGGGTTTAGGGTTAGGGTTTAGGGTTAGGGTTTAGGGTTTAGGGTTTAGGGTTTAGGGTTAGGGTTTAGGGTTTAGGGTTTAGGTGGTTTAGGGTTTAGGGTTTAGGGTTTAGGGTTTAGGGTTTAGGGTTTAGGGTTTAGGGTTAGGGTTTAGGGTTTAGGGTTTAGGGTTTAGGGTTTAGGTTTAGGGTTTAGGGTTTAGGGTTTAGGGTTTAGGGTTTAGGGGTTTAGGGTTTAGGGTTTAGGTTTAGGGTTTAGGGTTTAGGGTTTAGGGTTTAGGGTTTAGGTTTAGGGTTTAGGGTTTAGGGTTTAGGGTTTAGGGTTTAGGGTTTAGGGTTTAGGGTTTAGGGTTGGTTAGGGTTTAGGGTTTAGGGTTTAGGGTTTAGGGTTTAGGGTTTAGGTGGGTTTAGGGTTTAGGGTTTAGGGTTTAGGGTTTAGGGTTTAGGGTTTAGGTTTAGGGTTTAGGGTTTAGGGTTTAGGGTTTAGGGTTTAGGGTTTAGGGTTTAGGGTTTAGGGTTTAGGGTTTAGGGTTTAGGGTTTAGGGTTTAGGGTTTAAGGGTTTAGGGTTTAGGGTTTTAGGGTTTAGGGTTTAGGGTTTAGGGTTTAGGGTTTAGGGTTTAGGGTTTAGGGTTTAGGGTTTAGGGTTTAGGGTTTAGGGTTTAGGGTTTAGGGTTTAGGGTTTAGGGTTTAGGGTTAGGGTTTAGGGTTTAGGGTTTAGGGTTTAGGGTTTAGGTTTAGGGTTTAGGGTTAGGGTTTAGGGTTTAGGGTTTAGGGTTTAGGGTTTAGGTTTAGGGTTTAGGGTTTAGGGTTAGGTTTAGGGTTTTAGGGTTTAGGGTTTAGGGTTTAGGGTTTAGGGTTAGGGTTTAGGGTTTAGGGTTTAGGGTTTAGGGTTTAGGGTTAGGGTTTAGGGTTTAGGGTTAGGGTTTAGGGTTAGGGTTTAGGGTTTAGGTTAGGGTTTAGGGTTTAGGGTTAGGGTTTAGGTTTAGGGTTAGGGTTTAGGGTTTAGGGTTTAGGGTTTAGGGTTTAGGGTTTAGGGTTTAGGGTTTAGGGTTTAGGGTTTAGGGTTTAGGGTTTAGGGTTTAGGGTTTAGGGTTAGGTTTAGGGTTAGGGTTTAGGGTTTAGGGTTTAGGGTTTAGGGTTTAGGGTTTAGGGTTTAGGGTTTAGGGTTTAGGGTTTAGGGTTAGGGTTAGGGTTTAGGGTTTAGGGTTAGGGTTAGGGTTTAGGGTTTAGGGTTTAGGGTTTAGGTTTAGGGTTAGGGTTTAGGGTTTAGGGTTTAGGGTTTAGGGTTTAGGGTTAGGGTTTAGGGTTTAGGGTTTAGGGTTTAGGGTTAGGGTTTAGGGTTTAGGGTTTAGGGTTAGGGTTTAGGGTTTAGGGTTTAGGGTTTAGGTTTAGGGTTTAGGGTTTAGGGTTTAGGGTTTAGGGTTTAGGGTTAGGGTTTAGGGTTTAGGGTTTAGGGTTTAGGGTTTAGGGTTTTAGGGTTTAGGGTTTAGGGTTTAGGGTTTAGGGTTTAGGGTTTAGGGTTTAGGGTTTAGGGTTTAGGGTTTAGGGTTTAGGGTTTAGGTTTAGGGTTTAGGGTTTAGGGTTTAGGGTTTAGGGTTTAGGGTTTAGGGTTTAGGGTTTAGGGTTTAGGGTTTAGGGTTTAGGGTTTAGGGTTTAGGGTTTAGGGTTTAGGGTTTAGGGTTTAGGGTTTAGGGTTTAGGGTTTTAGGGTTAGGGTTTAGGGTTTAGGGTTTAGGGTTTAGGGTTTAGGGTTAGGGTTTAGGGTTTAGGGTTTAGGGTTTAGGGTTTAGGGTTTAGGGGTTTAGGGTTTAGGGTTAGGGTTTAGGGTTAGGGTTAGGGTTTAGGGTTTAGGGTTTAGGGTTTAGGGTTTAGGGTTTAGGGTTTAGGGTTTAGGGTTAGGGTTTAGGGTTTAGGGTTTAGGGTTTAGGGTTTAGGGTTTAGGGGTTTAGGGTTTAGGGTTTAGGGTTTAGGGTTTAGGGTTTAGGGTTAGGTTTAGGGTTTAGGGTTTAGGGTTTAGGGTTAGGGTTTAGGGTTTAGGGTTTAGGGTTAGGGTTTAGGGTTTAGGGTTTAGGGTTTAGGGTTTAGGGTTTAGGGTTTAGGGTTTAGGGTTTAGGGTTTAGGGTTTAGGGTTAGGGTTTAGGGTTTAGGGTTTAGGTTTAGGGTTTGGGTTTAGGGTTTAGGGTTTAGGGTTTAGGGTTAGGGTTTAGGGTTTAGGGTTTAGGGTTTAGGGTTTAGGGTTTAGGTTTAGGGTTTAGGGTTTAGGGTTTAGGGTTTAGGGTTTAGGGTTTAGGGTTTAGGGTTAGGGTTAGGGTTTAGGGTTTAGGGTTTAGGGTTTAGGTTTAGGGTTTAGGGTTTAGGGTTTAGGGTTTAGGGTTTAGGGTTAGGGTTTAGGGTTTAGGGTTTAGGGTTAGGGTTTAGGGTTTAGGGTTTAGGGTTAGGGTTTAGGGTTTAGGGTTTAGGGGTTTAGGGTTTAGGGTTTAGGGTTTAGGGTTTAGGGTTTAGGGTTTAGGGTTAGGGTTTAGGTTAGGGTTTAGGGTTTAGGTTAGGGTTTAGGGTTTAGGGTTTAGGGTTTAGGGTTTAGGGTTTAGGGTTTAGGGTTTAGGGTTTAGGGTTAGGGTTTAGGGTTTAGGGTTTAGGGTTTAGGGTTAGGGTTTGGGTTTAGGGTTTAGGGTTTAGGGTTAGGGTTTAGGGTTTAGGGTTTAGGGTTAGGTTTAGGGTTAGGGTTTAGGGTTTAGGGTTTAGGGTTAGGGTTTAGGGTTTAGGGTTTAGGGTTTAGGGTTTAGGGTTTAGGGTTTAGGGTTTAGGGTTTAGGTTTAGGGTTTAGGGTTTAGGGTTAGGGTTTAGGGTTTAGGGTTTAGGGTTTAGGGTTTAGGGTTTAGGGTTTAGGGTTTAGGGTTTAGGGTTAGGGTTAGGGTTTAGGGTTTAGGGTTTAGGGTTTAGGGTTTAGGGTTAGGGTTTAGGGTTTAGGGGTTTAGGGTTAGGTTTAGGGTTTAGGGTTTGGGTTTAGGGTTTAGGGTTTAGGGTTTAGGGTTTAGGGTTTAGGGTTTAGGGTTTAGGGTTTAGGGTTTAGGGTTAGGGTTTAGGGTTTAGGGTTTAGGGTTTAGGGTTTAGGGTTTAGGGTTTAGGGTTTAGGGTTTAGGGTTTAGGGTTTAGGGTTTAGGGTTTAGGGTTTAGGGTTTAGGGTTTAGGGTTAGGTAGGGTTAGGGTTTAGGGTTTAGGGTTTAGGGTTTTAGGGTTTAGGGGTTTAGGGTTTAGGGGTTTAGGGTTTAGGGTTTAGGGTTAGGGTTTAGGGTTTAGGGTTTAGGGTTTAGGGTTTAGGGTTTAGGGTTTAGGGTTTAGGGTTTAGGGTTTAGGGTTTAGGGTTTAGGGTTTAGGGTTTAGGGTTAGGGTTTAGGGTTTAGGGTTTAGGGTTTAGGGTTTAGGGTTTTAGGGTTTAGGGTTTAGGGTTAGGGTTTAGGGTTTAGGGTTTAGGGTTAGGGTTTAGGGTTAGGGTTTAGGGTTTAGGGTTTAGGGTTTAGGGTTTAGGGTTTAGGGTTAGGGTTTAGGGTTTAGGGTTTAGGGTTAGGGTTTAGGGTTTAGGGTTTAGGGTTTAGGGTTTAGGGTTTAGGGTTAGGGTTTAGGGTTTAGGGTTTAGGGTTAGGTTAGGGTTTAGGGTTTAGGGTTAGGGTTTAGGGTTTAGGGTTTAGGGTTTAGGGTTTAGGGTTTAGGGTTTAGGGTTTAGGGTTTAGGGTTTAGGGTTTAGGGTTTAGGGTTTAGGGTTTAGGGTTTAGGGTTTAGGGTTTAGGGTTAGGGTTTAGGGTTTAGGGTTTAGGGTTAGGGTTAGGGTTTAGGGTTTAGGGTTTAGGGTTAGGGTTTAGGGTTTAGGGTTTAGGGTTTAGGTTTAGGGTTTAGGGTTTAGGGTTTAGGGTTTAGGGTTTAGGGTTTAGGGTTTAGGGTTTAGGGTTTAGGGTTTAGGGTTTAGGGTTTAGGGTTTAGGGTTTAGGGTTTAGGGTTTAGGGTTTAGGGGTTTAGGGTTAGGGTTTAGGGTTTAGGGTTGGTTAGGGTTTAGGGTTTAGGGTTTAGGGTTTAGGGTTTAGGGTTTAGGGTTTAGGGTTTAGGGTTTAGGGTTTAGGGTTTAGGGTTTAGGTTAGGGTTTAGGGTTTAGGGTTTAGGGTTAGGGTTTAGGGTTTAGGGTTTAGGGTTTAGGGTTTAGGGTTTAGGGTTAGGGTTTAGGGTTTAGGGTTTAGGGTTTAGGGTTTAGGGTTTAGGGTTAGGGTTAGGGTTTAGGGTTTAGGGTTTAGGGTTTAGGGTTTAGGTTTAGGGTAGGGTTTAGGGTTTAGGGTTTAGGGTTTAGGGTTAGGGTTAGGGTTTAGGGTTTAGGGTTAGGGTTTAGGGTTTAGGGTTTAGGGTTTAGGGTTTTAGGTTTAGGGTTTAGGGTTTAGGGTTTAGGGTTTAGGTTTAGGGTTTAGGGTTTAGGGTTTAGGGTTTAGGGTTTAGGGTTAGGGTTTAGGGTTTAGGGTTTAGGGTTTAGGGTTTAGGGTTTAGGGTTTAGGGTTTAGGGTTTAGGGTTTAGGGTTAGGGTTTAGGGTTTAGGGTTTAGGGTTTAGGGTTTAGGGTTAGGGTTTAGGGTTTAGGGTTTAGGGTTTAGGGTTTAGGGTTTAGGGTTTAGGGTTTAGGGTTAGGGTTTAGGGTTTAGGGTTAGGGTTTAGGGTTTAGGGTTTAGGGTTAGGGTTTAGGGTTTAGGGTTTAGGTTTAGGGTTTAGGGTTTAGGGTTTAGGGTTTAGGGTTTAGGGTTTAGGGTTTAGGGTTTAGGGTTTAGGGTTAGGGTTTAGGGTTTAGGGTTAGGGTTTAGGTTTAGGGTTAGGGTTTAGGGTTTAGGGTTTAGGGTTTAGGGTTTAGGGTTTAGGGTTTAGGTTTAGGGTTTAGGGTTTAGGGTTTAGGGTTTAGGGTTTAGGGTTTAGGGTTTAGGGTTAGGGTTTAGGGTTTAGGGTTAGGGTTTAGGGTTTAGGGTTTAGGGTTTAGGGTTTAGGGTTTAGGGTTTAGGGTTTAGGGTTTAGGGTTTAGGGTTTAGGGTTTAGGGTTTAGGGTTTAGGGTTTAGGGTTTAGGGTTTAGGGTTTAGGGTTTAGGGTTTAGGGTTAGGGTTTAGGGTTTAGGGTTTAGGGTTTAGGTTTAGGGTTAGGGGTTTAGGGTTTAGGGTTTAGGGTTTAGGGTTAGGGTTTAGGGTTTAGGGTTTAGGGTTTAGGGTTTAGGTTTAGGGTTTAGGGTTTAGGGTTTAGGTTAGGGTTTAGGGTTTAGGGTTAGGGTTTAGGGTTAGGGTTTAGGGTTTAGGGTTTAGGGTTTAGGGTTTAGGTTTAGGGTTTAGGGTTAGGGTTAGGGTTTAGGTTAGGGTTTAGGGTTTAGGGTTTAGGGTTTAGGGTTTAGGGTTTAGGGTTTAGGGTTTAGGGTTTAGGGTTTAGGGTTTAGGGTTTAGGGTTTAGGGTTTAGGGTTTAGGGTTTAGGGTTTAGGGTTTAGGGTTAGGGTTTAGGGTTTAGGGTTTAGGGTTTAGGGTTTAGGGTTTAGGGTTTAGGGTTTAGGGTTAGGGTTTAGGGTTTAGGGTTTAGGGTTAGGGTTTAGGTTTAGGGTTTAGGGTTTAGGTTTAGGGTTTAGGGTTTAGGGTTTAGGGTTTAGGGTTTAGGGTTTAGGGTTTAGGGTTTAGGGTTTAGGGTTTAGGGTTTAGGGTTAGGGTTAGGGTTTAGGTTTAGGGTTAAGGGTTTAGGGTTTAGGGTTTAGGGTTAGGGTTTAGGGTTTAGGGTTTAGGGTTAGGGTTAGGGTTTAGGGTTTAGGGTTAGGGTTTAGGGTTTAGGGTTTAGGGTTTAGGGTTTAGGTTTAGGGTTAGGGTTTAGGGTTTAGGGTTTAGGGTTTAGGTTTAGGGTTTAGGGTTTAGGGTTTAGGGTTTAGGGTTTAGGGTTTAGGGTTTAGGGTTTAGGGTTTAGGGTTTAGGGTTAGGGTTTAGGGTTTAGGGTTTAGGGTTAGGGTTTAGGGTTTAGGTTTAGGGTTTAGGGTTTAGGGTTTAGGGTTTAGGGTTTAGGGTTAGGGTTTAGGGTTTAGGGTTAGGGTTTAGGGTTTAGGGTTTAGGGTTTAGGGTTTAGGGTTTAGGGTTTAGGGTTTAGGGTTTAGGGTTTAGGGTTTAGGGTTTAGGGTTTAGGGTTTAGGGTTTAGGGTTTAGGGTTAGGGTTTAGGGTTTAGGGTTTTAGGGTTTAGGGTTTAGGGTTAGGGTTTAGGGTTTAGGGTTTAGGGTTTAGGGTTTAGGGTTTAGGGTTTAGGGTTTAGGGTTTAGGGTTTAGGGTTTAGGGTTTAGGGTTTAGGGTTTAGGGTTTAGGGTTTAGGGTTTAGGGTTTAGGGTTAGGGTTTAGGGTTTAGGGTTAGGGTTAGGGTTTAGGGTTTAGGGTTTAGGGTTTAGGGTTTAGGGTTTAGGGTTTAGGGTTTAGGGTTTAGGGTTTAGGGTTTAGGGTTTAGGGTTTAGGGTTTAGGGTTTAGGGTTTAGGGTTTAGGGTTTAGGGTTAGGGTTTAGGGTTAGGGTTAGGGTTTAGGGTTTAGGGTTAGGGTTTAGGGTTTAGGGTTTAGGGTTTAGGGTTAGGGTTTAGGGTTTAGGGTTTAGGGTTTAGGGTTTAGGTTTAGGGTTTAGGGTTTAGGGTTTAGGGTTTAGGGTTTAGGGTTTAGGGTTTAGGGTTAGGGTTTAGGGTTTAGGGTTTAGGGTTTAGGGTTTAGGTTTAGGGTTTAGGGTTTAGGGTTTAGGGTTTAGGGTTTAGGGTTTAGGGTTTAGGGTTTAGGGTTTAGGGTTGGTTAGGGTTTAGGGTTAGGGTTTAGGGTTTAGGGTTAGGGTTTAGGGTTTAGGGTTTAGGGTTTAGGGTTTAGGGTTAGGGTTTAGGGTTTAGGGTTTAGGGTTAGGGTTTAGGGGTTAGGGTTTAGGGTTTAGGGTTTAGGGTTAGGGTTTAGGGTTTAGGGTTTAGGGTTAGGGTTTAGGGTTTAGGGTTTAGGGTTTAGGGTTTAGGGTTTAGGGTTTTAGGGTTTAGGGTTAGGGTTTAGGGTTTAGGGTTTAGGGTTTAGGGTTTAGGGTTTAGGGTTTAGGGTTTAGGGTTTAGGGTTTAGGGTTTAGGGTTTAGGGTTTAGGGTTAGGGTTTAGGGTTAGGGTGTTAGGGTTTAGGGTTTAGGGTTTTAGGGTTAGGGTTTAGGGTTTAGGGTTTAGGGTTAGGGTTTTAGGGGTTTAGGGTTTAGGGTTTAGGGTTTAGGGTTAGGTTTAGGGTTTAGGGGTTTAGGGTTTAGGGTTTAGGGTTTAGGGTTTAGGGTTTAG

**3DL**

GTGGG**TTTAGGG**TTTAGGGTTAGGGTTAGGGTTGTTAGGGTTTTAGGGTAGGGTTGGGTTAGGGTTTAGGGTTAGGGTTTAGGGTTTAGGGTTTAGGGTTTAGGGTTTAGGTTTGGGTTAGGGTTTAGGGTTTAGGGTTTAGGTTTAGGGTTTAGGGTGGTTAGGGTTTAGGGTTTAGGGTTAGGGTTTAGGGTTAGGGTTTAGGGTTTAGGGTTAGGGTTTAGGGTTTAGGGTTTAGGGTTTAGGGGTTAGGGTTTAGGGTTTAGGGTTTAGGGTTTAGGGTTTAGGGTTTAGGGTTAGGGTTTAGGGTGTTTAGGGTTTAGGGTTTAGGGTTTAGGGTTAGGGTTTAGGGTTTAGGGTTTAGGGTTTAGGGTTTTAGGGTTTAGGGTTTAGGGTTTAGGGTTTAGGGTTTAGGGTTTAGGGTTTAGGGTTAGGGTTTTTTAGGGTTTAGGGTTTAGGGTTTAGGGTTTTAGGGTTTAGGGTTTAGGGTTTAGGGTTAGGGTTTAGGGTTTTAGGGTTTAGGGTTAGGGTTTAGGGTTTAGGGTTTAGGGTTTAGGGTTTTAGGGTTTAGGGTTTAGGGTTTAGGGTTTAGGGTTAGGGTTTAGGGTTTAGGGTTTAGGGTTTAGGGTTTAGGGTTTAGGGTTAGGGTTTAGGGTTAGGGTTTAGGGTTTAGGGTTAGGGTTTAGGGTTTAGGGTTTAGGGTTTAGGGTTTAGGGTTTAGGGTTTAGGGTTTAGGGTTTAGGGTTTAGGGTTAGGGGTTTAGGGTTAGGGTTTAGGGTTTAGGGTTTAGGGTTTAGGGTTTAGGGTTTAGGGTTTAGGGTTTAGGGTTTAGGGTTTAGGGTTTAGGGTTTTAGGGTTAGGGTTTAGGGTTAGGGTTTAGGGTTTAGGGTTTAGGGTTTAGGGTTTAGGGTTTAGGGTTTAGGGTTTAGGGGTTTAGGGTTTAGGGTTTAGGGTTTAGGGTTTAGGGTTTAGGGTTTAGGGTTTAGGGTTTAGGGTTTAGGGTTTAGGGTTTAGGGTTTAGGGTTAGGGTTTAGGGTTTAGGGTTTAGGGTTTAGGGTTTAGGGTTTAGGTTTAGGGTTTAGGGTTAGGGTTTAGGGTTTAGGGTTTAGGGTTTAGGGTTTAGGGTTTAGGGTTTAGGGTTTAGGGTTTAGGGTTTAGGGTTTAGGGTTTAGGGTTTAGGGTTTAGGGTTTAGGTTAGGGTTTAGGGTTTAGGGTTTAGGGTTTAGGGTTTAGGGTTTAGGGTTTTAGGGTTTAGGGTTAGGGTTTAGGGTTTAGGGTTTAGGGTTTAGGGTTTAGGGTTTAGGGTTTAGGGTTTAGGGTTTAGGGTTTAGGGTTTAGGGTTAGGGTTTAGGGTTTAGGGTTTAGGGTTTAGGGTTTCGTTAGGGTTTAGGGTTTAGGGTTTAGGGTTTAGGGTTAGGGTTTAGGGTTTAGGGTTTAGGGTTTAGGGTTTAGGGTTTAGGGTTTAGGGTTTAGGGTTTAGGGTTTAGGGTTTAGGGTTTAGGGTTTAGGGTTAGGGTTTAGGGTTTAGGGTTTAGGGTTTAGGGTTTAGGGTTTAGGGTTTAGGGTTTAGGGTTTAGGGTTTAGGGTTTAGGGTTTAGGGTTTAGGGTTTAGGGTTTAGGGTTTAGGGTTAGGGTTTAGGGTTTAGGGTTTAGGGTTTAGGGTTTTAGGGTTTAGGGTTTAGGGTTTAGGGTTTTAGGGTTTAGGGTTTAGGGTTTAGGGTTTAGGGTTTAGGTTAGGGTTTAGGGTTAGGGTTTAGGGTTTAGGGTTTAGGGTTTAGGGTTTAGGGTTTAGGGTTTAGGGTTTAGGGTTTAGGGTTTAGGGTTTAGGGTTTAGGGTTTAGGGGTTTAGGGTTTAGGGTTTAGGGTTTAGGGTTTAGGGTTTAGGGTTTAGGGTTTAGGGTTTAGGGTTTAGGGTTTAGGGTTAGGGTTTAGGGTTTAGGGTTTAGGGTTTAGGGTTTAGGGTTTAGGGTTTTAGGGTTTAGGGTTTAGGGGTTTTAGGGTTTAGGGTTAGGGTTTTAGGGTTAGGGTTTAGGGTTTAGGGTTTAGGGTTTAGGGTTAGGGTTTAGGGTTAGGGTTTTAGGGTTAGGGTTTAGGGTTTAGGGTTTAGGGTTTAGGGTTAGGGTTTAGGGTTTAGGGTTTAGGGTTTAGGGTTTAGGTTTAGGGTTTTAGGGTTTAGGGTTTAGGGTTTAGGGTTTTAGGGTTTAGGGTTTAGGGTTTAGGGTTTAGGGTTTCGGGTTTAGGGTTTAGGGTTAGGTTTAGGGTTTAGGGTTAGGGTTTAGGGTTTAGGGAGTTAGGGTTTAGGGTTTAGGGTAGGGTTTAGGGTTTAGGGTTAGGGTTTAGGGTTTTAGGGTTAGGGTTTAGGGTTAGGGTTTAGGGTTTAGGGTTTAGGGTTTAGGGTTTAGGGTTTAGGGTTTTAGGGTTTAGGGTTTAGGGTTAGGTTAGGGTTTAGGGTTTAGGGTATAGGGTTTAGGGTTTAGGGTTTAGGGTTTAGGGTTTAGGGTTAGGGTTTAGGGTTTAGGGTTTAGGGTTAGGGTTTAGGGTTTAGGTTAGGTTTAGGGTTTAGGGTTAGGGTTAGGGTTTAGGGTTTAGGGTTTAGGGTTTAGGGTTTAGGGTTTAGGGTTTAGGGTTTAGGGTTTAGGGTTTAGGGTTTAGGGTTAGGGTTTAGGGGTTTAGGGTTAGGGTTAGGGTTTAGGGTTTAGGGTTTAGGGTTTAGGGTTTGGGTTTAGGGTTTAGGGTTTAGGGTTTAGGGTTTAGGGTTTAGGGTTTAGGGTTTAGGGTTTAGGGTTTAGGGTTTAGGGTTTAGGGTTTAGGGTTGGGTTTAGGGTTTAGGGTTTAGGGTTTAGGGTTTAGGGTTTAGGGTTTAGGGTTTAGGGTTTAGGGTTTAGGGTTAGGGTTTTAGGGTTAGGGTTTAGGGTTTAGGGTTTAGGGTTTAGGGTTTAGGGTTTAGGGTTTAGGGTTTAGGGTTTAGGGTTTAGGGTTTAGGGTTTAGGGGTTTAGGGTTTAGGGTTTAGGGTTTAGGGTTTAGGGTTTAGGGTTTAGGGTTTAGGGTTTAGGGTTTAGGGTTTAGGGTTTAGGGTTTAGGGTTTAGGGTTAGGTAGGGTTAGGGTTTAGGGTTTAGGGTTTAGGGTTTAGGTTAGGGTTTAGGGTTTAGGGTTTAGGGTTTAGGTTTAGGGTTAGGGTTTAGGGTTAGGGTTTAGGGTTTAGGTTTAGGTTTAGGGTTTAGGGTTTAGGGTTTAGGGTTATAGGGTTTAGGGTTTAGGGTTTAGGGTTTTAGGGTTTAGGGTTTAGGGTTAGGATTAGGGTTTAGGGTTTAGGGTTTAGGGTTTAGGGTTTAGGGTTTAGGGTTTAGGGTTTTTAGGGTTTAGGGTTTAGGGTTATGGGTTTAGGGTTTAGGGTTTTAGGGTTAGGGTTTAGGGTTTTAGGGTTTAGGGTTTAGGGTTTAGGGGTTTAGGGTTTAGGGTTTAGGGTTTAGGGTTTGGGTTAGGGTTTAGGGTTTAGGGTTTAGGGTTTAGGGTTTAGGTTGGGTTTAGGGTTTAGGGTTTAGGGTTTAGGGGTTTAGGGTTTAGGGTTTAGGGTTTAGGTTTAGGGTTTAGGGTTTAGGGTTTAGGGTTTAGGGGTTAGGGTTTTAGGGTTTTAGGGTTAGGGTTTAGGGTTTAGGGTTAGGGTTTAGGGTTTAGGGTTAGGGTTTAGGGTTTAGGGTTTAGGGTTTTAGGGTTTAGGGTTTAGGGTTTTAGGGTTTAGGGTTTAGGGTTTAGGGTTTAGGGTTTAGGGTTTAGGGTTTAGGGTTTAGGGTTTAGGGTTTAGGGTTTAGGGTTTAGGGTTTAGGGTTTTAGGGTTAGGGTTTAGGGGTTAGGGTTTAGGGTTTAGGGTTTAGGGTTTAGGGTTTAGGGTTAGGGTTTAGGGGTTAGGGGTTTAGGGTTTTAGGTTTAGGGTTTAGGGTTTAGGGTTTAGGGTTTAGGGTTAGGGTTAGGGTTTAGGGTTTAGGGTTTAGGGTTTAGGGTTTAGGGTTTTAGGGTTTAGGGTTTAGGGTTAGGGTTTAGGGGTTTAGGGTTTAGGGTTTAGGGTTTAGGGTTTAGGGTTTAGGGTTAGGGTTTAGGGTTTAGGGTTTAGGGTTTAGGGTTTAGGGTTTAGGGTTTAGGGTTAGGGTTTAGGGTTTAGGGTTTAGGGTTTAGGGTTTAGGGTTTAGGGTTTAGGGTTAGGGTTTTAGGGTTTAGGGTTTAGGGTTTAGGGTTTAGGGTTTAGGGTTTAGGGTTTAGGGTTTAGGGTTAGGGTTAGGTTAGGGTTTAGGGTTTAGGGGTTTAGGGTTTAGGGTTTAGGGTTTAGGGTTTAGGGTTTAGGGTTTTAGGGTTTAGGGTTTAGAGGGTTTAGGGTTTAGGGTTTAGGGTTTAGGGTTTAGGGTTTAGGGTTTAGGGTTTAGGGTTTAGGGTTTTAGGGTTAGGGTTTAGGTTTAGGGTTTAGGGTTTTGGGTTAGGGTTTAGGGTTTAGGGTTTTAGGGTTTAGGGTTAGGGTTTTAGGGTTTAGGGTTTAGGGTTAGGGTTTAGGGTTTAGGGTTTAGGGTTTAGGGTTAGGGTTTAGGGTTTAGGGTTTAGGGTTTAGGGTTTAGGGTTTAGGTTTAGGGGTTTAGGGTTAGGGTTAGGGTTTAGGGTTAGAGGTTTGGGTTTAGGGTTTAGGGTTTAGGGTTTTAGGGGTTAGGGTTTAGGGTTTAGGGTTTAGGGTTTTAGGGTTTAGGGGTTTAGGGTTTAGGGTTTAGGGTTTAGGGTTTTAGGGGTTTAGGGTTTAGGGTTTAGGGTTGAGGGTTTAGGGTTTAGGGTTTAGGGTTGTAGGGTTTAGGGTTTAGGGTTTAGGGTTTAGGGTTTTAGGGTTTTTGGTAGGGTTTAGGGTTTAGGGTTTAGGGTTTAGGGTTTAGGGTTAGGGTTTAGGGTTTAGGGTTTAGGGTTTAGGGTTTAGGGTTTAGGGTTTAGGGTTTAGGTTAGGGTTTAGGGTTTAGGGTTTAGGGTTTAGGGTTTAGGGTTTAGGGTGTTTAGGGTTTAGGGTTTAGGGTTTAGGGTTTGTTAGGGTAGGTTTAGGGTTTAGGGTGTTAGGGTTTAGGGTTAGGGTTTTAGGGTTTAGGGTTTTAGGGTTTAGGGTTTAGGGTTTAGGGTTTAGGGTTTAGGGTTTAGGGTTTAGGGTTTAGGGTTTAGGGTTTAGGGTTTAGGGTTAGGGTTTAGGTTTAGGGTTGTTGGGTTTAGGGTTTAGGGTTTAGGGGTTTAGGGTTTAGGGGTTTAGGGTTTAGGGTTTAGGGTTTAGGGGTTTAGGGTTTAGGGTTTAGGGTTTAGGGTTTAGGGTTTAGGGTTTAGGGTTTTAGGGTTTAGGGTTTAGGGTTAGGGTTTTAGGGTTAGGGTTTAGGGTTTAGGGTTTAGGGTTTAGGGTTTAGGGTTAGGGTTTAGGGTTTAGGGTTAGGGGTTTAGGGTTTAGGGTTTAGGGTAGGGTTTAGGGTTTTGGGTTTAGGGGTTTAGGGTTTAGGGTTTAGGGTTTAGGGTTTAGGGTTAGGGTTTAGGGTTTAGGGTTTAGGGTTAGGGGTTTAGGGTTTAGGGGTTTGGGTTTTAGGGTTTAGGGGTTTAGGGTTTAGGGTTTAGGGTTTAGGGTTTAGGGTTTAGGGTTTAGGGTTTTGGGTTTAGGGTTTAGGGTTTAGGGTTTAGGGTTTAGGGTTTAGGGGTTAGGGTTTAGGTGGGTTTTAGGGGTTTGGGTTTTGGGTTTAGGGTTAGGGTTTAGGGTTTAGGGTTTAGGGTTTAGGGGTTTAGGGTTTAGGGGTTAGGGTTAGGGTTTAGGGTTTAGGGTTTAGGGTTTAGGGTTTAGGGTTTAGGGTTTAGGGTTTAGGGTTTAGGGTGTGGGTTTAGGGTTTAGGGTTTTGGGGTTTAGGGTTTAGGGTTTAGGGTTGGGTTAGGGTTTAGGGTTTTGGGTTAGGGTTTAGGGTTTAGGGTTTAGGGTTAGGGTTTAGGGGTTTAGGGTTAGGGTTTAGGGTTTAGGGTTTTAGGGTTTAGGGTTGAGGGTTTTGGGTTAGGGTTTTAGGGTTTAGGGTTTAGGGTTTTGGGTTAGGGGTTAGGGTTTAGGGTTTAGGGTTTGGGTTAGGGGTTAGGGTTTTGGGTGGGTTAGGGTTAGGGTTTTAGGGTTTAGGGTTTTGGGTTTAGGGTTTAGGGTTTAGGGGTTTAGGGTTTTAGGGTTAGGGGTTAGGGTTTAGGGTTTTAGGGTTTTGGGTTTAGG

**5DS**

TTTTAGGG**TTTAGGG**TTTAGGGTTTAGGGTTTAGGGTTTAGGGTTTAGGGTTAGGGTTTAGGGTTTAGGGTTAGGGTTTAGGGTTTAGGGTTTAGGGTTTAGGGTTTAGGGTTTAGGGTTTAGGGTTAGTAGGGTTTAGGGTTTAGGGTTAGGGTTTAGGGTTTAGGGTTTAGGGTTTAGGGTTAGGGTTTAGGGTTAGGGTTTAGGGTTAGGGTTAGGGTTTAGGGTTAGGGTTTAGGGTTAGGGTTTAGGGTTTAGGGTTTAGGGTTTAGGGTTTAGGTTTAGGGTTTTGGGTTTAGGGTTTAGGGTTAGGGTTTAGGGTTAGGGTTTAGGGTTTAGGGTTTAGGGTTTAGGTTTAGGGTTAGGGTTTAGGGTTTAGGGTTAGGGTTTAGGGTTTAGGGTTTAGGGTTTAGGGTTTAGGGTTTAGGGTTAGGGTTTAGGGTTTAGGGTTTAGGGTTTAGGGTTTAGGGTTTAGGGTTTAGGGTTTAGGGTTTAGGGTTAGGGTTTAGGGTTAGGGTTAGGGTTTAGGGTTAGGGTTTAGGGTTTAGGGTTTAGGGTTTAGGGTTAGGGTTTAGGGTTTAGGGTTTAGGGTTTAGGGTTTAGGGTTTAGGGTTTAGGGTTTAGGGTTTAGGGTTTAGGGTAGGGTTTAGGGGTTAGGGTTTAGGGTTTAGGTTTAGGGTTAGGGTTTAGGGTTAGGGTTTAGGTTTAGGGTTTAGGGGTTAGGGTTTAGGGTTTAGGGTTTAGGGTTTAGGGTTAGGGTTTAGGGTTTAGGGTTTAGGGTTAGGGTTTAGGGTTTAGGGGTTTAGGGTTTAGGGGTTTAGGGTTAGGGTTTAGGGTTAGGGTTTAGGGGTTAGGGTTAGGGTTTAGGGTTTAGGGGTTTAGGTTTAGGGTTTAGGGTTTTAGGGTTTAGGGTTTAGGGTTTAGGGTTTAGGGTTTAGGGTTTAGGGTTTAGGGGTTTAGGGTTTAGGGTTAGGGTTTAGGGTTTAGGGTTAGGGTTAGGGTTTAGGGTTTAGGGTTAGGGTAGGTTTAGGGTTTAGGGTTTAGGGTTTAGGGTTTTAGGTTTAGGGTTTAGGGTTTAGGGTTTAGGGTTTAGGGTTTAGGGTTTAGGGTTTAGGGGTTTAGGGTTTAGGGTTTAGGGTTTAGGGTTTAGGGTTTAGGGTTTAGGTTAGGGTTTAGGGTTTAGGGTTTAGGGTTTAGGGTTTAGGGGTTAGGGTTTAGGGTTAGGGTTTAGGGTTAGGGTTTAGGGTTTAGGGTTTAGGGTTAGGGTTTAGGGTTTAGGGTTTAGGGTTTAGGTGTTAGGGTTTAGGGTTTAGGGTTTAGGGTTTAGGGTTTAGGGTTAGGGTTTAGGGTTAGGGTTTAGGGTTAGGGTTTAGGGTTTAGGGTTTAGGGTTTAGGGTTAGGGTTTAGGGTTTAGGGTTAGGGTTAGGGTTTAGGGTTGGTTTAGGGTTTAGGGTTTAGGGTTTAGGGTTGTTAGGGTTTAGGGTTTAGGGTTTAGGGTTTAGGGTTGTTAGGTTTAGGGTTAGGGTTTAGGGTTTTAGGGTTTAGGGTTTAGGGTTTAGGGTGTTAGGGTTTAGGGGTTTAGGGTTAGGGTTTAGGGTTGGGTAGGGTTTAGGGTTTAGGGTTAGGGTTAGGGTTTAGGGTTTAGGGGTTTAGGGTTAGGGTTTAGGGTTTAGGGTTAGGGGTTTAGGGTTTAGGGTTTAGGGTTTAGGTAGGGTTAGGGTTTAGGGTTTAGGGTTTAGGGTTTAGGGTTTAGGGTTTAGGGTTTAGGGTTTAGGGTTTAGGGTTTAGGGTTTAGGGTTTAGGGTTTAGGGTTTAGGGTTGTAGGGTTTAGGGTTAGGGTTTAGGGTTTAGGGTTTAGGGTTTAGGGTTTAGGGTTTAGGGTTTAGGGTTAGGGTTTAGGGTTTAGGGTTTAGGGTTAGGGTTTAGGGTTTAGGGTTAGGGTTTAGGGTTTAGGGTTTAGGGTTTAGGGTTTAGGGTTTAGGGTTAGGGTTTAGGGTTTAGGGTTAGGGGTTTAGGGTTTAGGGTTTAGGGTTTAGGGTTTAGGGTTAGGGTTTAGGGTTAGGGTTTAGGGTTAGGGTTTAGGGTTTAGGGTTTAGGGGTTTAGGGTTTAGGGTTAGGGTTTAGGGTTTAGGGTTTAGGGTTAGGGTTTAGGGTTAGGGTTTAGGGTTTAGGGTTAGGGTTTAGGGTTTAGGGTTAGGGTTTAGGGTTTAGGTTTAGGGTTAGGGTTAGGGTTTAGGGTTTAGGGTTTAGGGTTTAGGGTTTAGGGTTAGGGTTTAGGGTTAGGGTTAGGGTTTAGGGTTAGGGTTTAGGGTTTAGGGTTTAGGGTTTAGGGTTTAGGGTTTAGGGTTAGGGTTTTAGGGTTTAGGGTTTAGGGTTTAGGGTTTTAGGGTTTAGGGTTTAGGTTTAGGGTTTAGGGGTTAGGGTTTAGGGTTTAGGGTTAGGGTTTAGGGTTTAGGGTTTAGGGGGTTTAGGGTTAGGGTTTAGGGTTTAGGGTTTAGGTTAGGGTTAGGGTTTAGGGGTTAGGGTTTAGGGTTAGGGTTTAGGGGTTTAGGGTTTAGGGTTTAGGGTTAGGGTTTAGGGTTTAGGGTTTAGGGGTTTAGGGTTAGGGTTTAGGGTTTAGGGTTTAGGGTTTAGGGTTTAGGGTTTAGGGTTAGGTTTAGGGTTAGGGTTGGGTTTAGGGTTAGGGTTAGGGTTAGGGTTTAGGGTTTAGGGTTTAGGGTTTAGGGTTTAGGGTTAGGGTTTAGGGTTTAGGGTTTAGGGTTTAGGGTTTAGGGTTTAGGGTTTAGGGTTTAGGGTTTAGGGTTTTGTTAGGGTTTAGGGTTTAGGGTTTAGGGTTAGGGTTTAGGGTTAGGGTTAGGGTTTAGGGTTTAGGGTTTAGGGTTAGGGTTAGGTTAGGGTTTAGGGTTTAGGTTTAGGGTTTAGGGTTTAGGGTTTAGGGTTTAGGGTTTAGGGTTTAGGGTTTAGGGTTTAGGGTTTAGGGTTTAGGGTTTAGGGTTTAGGGTTTAGGGTTTAGGGTTTAGGGTTTAGGTTTAGGGTTTAGGGTTTAGGGTTTAGGGTTTAGGGTTAGGGTTTAGGTTTAGGGTTTAGGGTTGTAGGGTTAGGGTTTAGGTTTAGGGTTTAGGGTTTAGGGTTTAGGGTTTAGGGTTTAGGGTTTAGGGTTTAGGGTTTAGGGTTTAGGGTTTAGGGTTTAGGGTTTAGGGTTTAGGGTGTTAGGGTTAGGGTTTAGGGTTTAGGGTTTAGGGTTGTAGGGTTTAGGGTTTTAGGGTTTAGGGTTTAGGGTTAGGGTTTAGGGTTTAGGGTTTAGGGTTTAGGGTTAGGGTTTTAGGGTTTAGGGTTTAGGGTTAGGGTTTGTAGGGTTTAGGGTTTAGGGTTTAGGGTTTAGGGTTTAGGGGTTTAGGGGTTTAGGGTTTAGGGTTGTTAGGGTTTAGGGTTTAGGGGTTAGGGGTTAGGGGTTAGGGTTTAGGGTTAGGGGTTAGGGTTAGGGTTTAGGGTTTAGGGGTTGTTAGGGTTAGGGTTAGGGTTTAGGGTTTAGGGGTTTAGGGTTTAGGTTAGGGTTTAGGGGTTTTAGGGTTTAGGGTTAGGGTTAGGGTTTAGGGTTTAGGGTTTAGGGTTTAGGGTTTAGGGGTTTAGGGGTTAGGGTTTAGGTTTAGGGTTTTAGGGGTTTAGGGTTTAGGTTTAGGGTTAGGGTTTAGGGTTTTAGGGTTAGGGTTTAGGGTTTTAGG

**5DL**

TAGGGTTAGGG**TTTAGGG**TTTAGGGTTTAGGGTTTAGGGTTTAGGGTTTAGGGTTTAGGGTTTAGGGTTTAGGTTTAGGGTTTAGGGTTTAGGGTTAGGGTTTAGGGTTTAGGGTTTAGGATTTAGGGTTAGGGTTTAGGGTTTAGGGTTAGGGTTTAGGGTTAGGGTTTAGGGTTTAGGGTTTAGGGTTTAGGGTTAGGGTTAGGGTTTAGGGTTAGGGTTTAGGGTTTAGGGTTTAGGGTTAGGGTTTAGGGTTTAGGGTTAGGGTTTAGGGTTAGGGTTTAGGGTTTAGGGTTTAGGGTTAGGGTTTAGGGTTTAGGGTTTAGGGTTTAGGGTTTAGGGTTTAGGGTTTAGGGTTTAGGGTTTAGGGTTTAGGGTTTAGGGTTTAGGGTTTAGGGTTTAGGGTTTAGGGTTTAGGGTTTAGGGTTTAGGGTTTAGGGTTTAGGGTTTAGGGTTTAGGGTTTAGGGTTTAGGGTTTAGGGTTTAGGGTTAGGGTTTAGGGTTTAGGGTTTAGGGTTTAGGGTTAGGGTTTAGGGTTTAGGGTTTAGGGTTTAGGGTTTTAGGGTTTAGGGTTAGGGTTTAGGGTTTAGGGTTTAGGGTTTAGGGTTTAGGGTTTAGGGTTTAGGGTTTAGGGTTTAGGGTTTAGGGTTTAGGGTTTAGGGTTTAGGGTTTAGGGTTTAGGGTTTAGGGTTTAGGGTTTAGGGTTTAGGGTTTAGGGTTTAGGGTTTAGGGTTTAGGGTTTAGGGTTTAGGGTTTAGGGTTTAGGGTTTAGGGTTTAGGGTTAGGGTTTAGGGTTTAGGGTTTAGGGTTTAGGGTTTAGGGTTAGGGTTTAGGGTTTAGGGTTAGGGTTTAGGGTTTAGGGTTTAGGGTTTAGGGTTTAGGGTTTAGGGTTTAGGGTTTAGGGTTAGGGTTAGGGTTTAGGGTTTAGGGTTAGGGTTTAGGGTTAGGGTTTAGGGTTTAGGGTTTAGGGTTTAGGGTTTAGGGTTTAGGGTTTAGGGTTTAGGGTTAGGGTTTAGGGTTAGGGTTTAGGGTTTAGGGTTTAGGGTTTAGGGTTTAGGGTTTAGGGTTGGGTTTAGGGTTAGGGTTTAGGGTTTTAGGGTTAGGGTTTAGGGTTTAGGGTTAGGGTTAGGGTTTAGGGTTTAGGGTTAGGGTTAGGGGTTTAGGGTTTAGGGTTAGGGTTTAGGGTTTAGGGTTTAGGGTTTAGGGTTTAGGGTTAGGGTTTGGGGTTAGGGTTTAGGGTTTAGGGTTTAGGGTTAGGGTTAGGGTTTAGGGTTTAGGGTTTAGGGTTTAGGGTTAGGGTTTAGGGTTTAGGGTTAGGGTTAGGGTTTAGGGTTAGGGTTTAGGGTTTAGGGTTTAGGGTTTAGGGTTTAGGGTTTAGGGTTTAGGGTTAGGGTTAGGGTTAGGGTTTAGGGTTAGGGTTTAGGGTTAGGGTTTAGGGTTTAGGGTTTAGGGTTAGGGTTTAGGGTTAGGGTTAGGGTTTAGGGTTTAGGGTTTAGGGTTTAGGGTTAGGGTTTAGGGTTTAGGGTTTAGGGTTTAGGGTTTAGGGTTAGGGTTTAGGGTTAGGGTTTAGGGTTTAGGGTTTAGGGTTAGGGTTAGGGTTTAGGGTTTAGGGTTTAGGGTTTGGGTTTAGGGTTAGGGTTTAGGGTTTAGGGTTAGGGTTAGGGTTTAGGGTTTAGGGTTAGGGTTTAGGGTTAGGGTTTAGGGTTTAGGGTTTAGGGTTTAGGGTTTAGGGTTAGGGTTTAGGGTTTAGGGTTTAGGGTTTAGGGTTTAGGGTTTAGGGTTTAGGGTTTAGGGTTTAGGGTTTAGGGTTTAGGGTTTAGGGTTAGGGTTTAGGGTTTAGGGTTTAGGGTTTAGGGTTTAGGGTTTAGGGTTTAGGGTTTAGGGTTTAGGGTTTAGGGTTTAGGGTTTAGGGTTTAGGGTTAGGGTTTAGGTTTAGGGTTTAGGGTTTAGGGTTTAGGGTTTAGGGTTTAGGGTTTAGGGTTTAGGGTTTAGGGTTAGGGTTTAGGGTTTAGGGTTTAGGGTTTAGGGTTTAGGGTTTAGGGTTTAGGGTTTAGGGTTTAGGGTTTAGGGTTAGGGTTTAGGGTTTAGGGTTTAGGGTTTAGGGTTTAGGGTTAGGGTTTAGGGTTTAGGGTTTAGGGTTTAGGGTTTAGGGTTTAGGGTTAGGGTTTAGGGTTTAGGGTTTAGGGTTTAGGGTTTAGGGTTAGGGTTTAGGGTTTAGGGTTTAGGGTTTAGGGTTTAGGGTTAGGGTTTAGGGTTAGGGTTTAGGGTTAGGGTTTAGGGTTTAGGGTTTAGGGTTTAGGGTTTAGGGTTTAGGGTTTAGGGTTTAGGGTTTAGGGTTTAGGGTTTAGGGTTTAGGGTTTAGGGTTTAGGGTTTAGGGTTAGGGTTTTAGGGTTTAGGGTTTAGGGTTTAGGGTTTAGGGTTTAGGGTTTAGGGTTTAGGGTTTAGGGTTTAGGGTTTAGGGTTTAGGGTTTAGGGTTAGGGTTTAGGGTTTAGGGTTAGGGTTTAGGGTTTAGGGTTTAGGGTTTAGGGTTTAGGGTTTAGGGTTTAGGGTTTAGGGTTTAGGGTTTAGGGTTTAGGGTTTAGGGTTTAGGGTTAGGGTTTAGGGTTTAGGGTTTAGGGTTTAGGGTTTAGGGTTTAGGGTTTAGGGTTAGGGTTTAGGGTTTAGGTTTAGGGTTTAGGGTTAGGGTTTAGGGTTAGGGTTTAGGGTTTAGGGTTTAGGGTTTAGGGTTTAGGGTTTGGGTTTAGGGTTAGGGTTTAGGGTTTAGGGTTTAGGGTTTAGGGTTTAGGGGTTTAGGGTTTAGGGTTTAGGGTTTAGGGTTAGGGTTAGGGTTTAGGGTTTAGGGTTTAGGGTTTAGGGTTTAGGGTTTAGGGTTTAGGGTTTAGGGTTTAGGGTTTAGGGTTTAGGGTTTAGGGTTTAGGGTTTAGGGTTTAGGGTTTAGGGTTTAGGGTTGGTTTAGGGTTTAGGGTTTAGGGTTTAGGGTTTAGGGTTTAGGGTTAGGGTTTAGGGTTTAGGGTTTAGGGTTTAGGGTTAGGGTTTAGGGTTTAGGGTTTAGGGTTTAGGGTTTAGGGTTTAGGGTTTAGGGTTAGGGTTTAGGGTTTAGGGTTTAGGGTTTAGGGTTTAGGGTTAGGGTTAGGGTTTAGGGTTTAGGGTTTAGGGTTTAGGGTTTAGGGTTTAGGGTTTAGGGTTTAGGGTTAGGGTTTAGGGTTTAGGGTTTAGGGTTTAGGGTTTAGGGTTAGGGTTTAGGGGTTAGGGTTTAGGTTTAGGGTTTAGGGTTTAGGGTTTAGGGTTTAGGGTTAGGGTTTAGGGTTTAGGGTTAGGGTTTAGGTTAGGGTTTAGGGTTTAGGGTTAGGGTTAGGGTTTAGGGTTTAGGGTTAGGGTTTAGGGTTTAGGGTTTAGGGTTTAGGGTTGGTTAGGGTTAGGGTTTAGGGTTTAGGGTTAGGGTTTAGGGTTTAGGGTTTAGGGTTTAGGGTTTAGGGTTAGGGTTTAGGGTTTAGGGTTTAGGGTTTAGGGTTTAGGGTTTAGGGTTTAGGGTTTAGGGTTTAGGGTTAGGGTTTAGGGTTTAGGGTTAGGGTTTAGGGTTTAGGGTTTAGGGTTTAGGGTTTAGGGTTTAGGGTTTAGGGTTTAGGGTTTAGGGTTTAGGGTTAGGGTTTAGGGTTTAGGGTTTAGGGTTTAGGGTTTAGGGTTTAGGGTTTAGGGTTAGGGTTTAGGGTTTAGGGTTTAGGGTTAGGGTTTAGGGTTTAGGGTTTAGGGTTTAGGGTTTAGGGTTTAGGGTTTAGGGTTAGGGTTTAGGGTTTAGGGTTTAGGGTTTAGGGTTAGGGTTTAGGGTTTAGGGTTTAGGGTTTAGGGTTTAGGGTTTAGGGTTTAGGGTTTAGGGTTTAGGGTTTAGGGTTTAGGGTTAGGGTTTAGGGTTTAGGGTTTAGGGTTTAGGGTTTAGGGTTAGGGTTTAGGGTTTAGGGTTTAGGGTTTAGGGTTTAGGGTTTAGGTTTAGGGTTTAGGGTTTAGGGTTTAGGGTTTAGGGTTTAGGGTTTAGGGTTTAGGGTTTAGGGTTTAGGGTTAGGGTTTAGGGTTTAGGGTTTAGGGTTTAGGGTTAGGGTTTAGGGTTTAGGGTTTAGGGTTTAGGGTTTAGGGTTTAGGGTTTAGGGTTTAGGGTTTAGGGTTTAGGGTTTAGGGTTTAGGGTTTAGGGTTTTGGGTTTAGGGTTTAGGGTTTAGGGTTTAGGGTTTAGGGTTTAGGGTTTAGGGTTTAGGGTTTGGGTTTAGGGTTTAGGGTTTAGGGTTTAGGGTTTAGGGTTTAGGGTTTAGGGTTAGGGTTAGGGTTTAGGGTTTAGGGTTTAGGGTTTAGGGTTTAGGGTTTAGGGTTTAGGGTTTAGGGTTTAGGGTTTAGGGTTTAGGGTTAGGGTTTAGGGTTTAGGGTTTAGGGTTTAGGGTTTAGGGTTTAGGGTTTAGGGTTTAGGGTTTAGGGTTTAGGGTTTAGGGTTTAGGGTTTAGGGTTTAGGGTTTAGGGTTAGGGTTTAGGGTTTAGGGTTTAGGGTTTAGGGTTTAGGGTTTAGGGTTTAGGGTTTAGGGTTTAGGGTTAGGGTTAGGGTTAGGGTTTAGGGTTTAGGGTTTAGGGTTTAGGGTTTAGGGTTTAGGGTTTAGGGTTTAGGGTTTAGGGTTAGGGTTTAGGGTTTAGGGTTTAGGGTTTAGGGTTTAGGGTTTAGGGTTTAGGGTTTAGGGTTTAGGGTTTAGGGTTTAGGGTTTAGGGTTTAGGGTTAGGGTTTAGGGTTTAGGGTTTAGGGTTTAGGGTTTGGGTTTAGGGTTTAGGGTTTAGGGTTTAGGGTTTAGGGTTAGGGTTTAGGGTTTAGGGTTTAGGGTTTAGGGTTTAGGGTTAGGGTTTAGGGTTTAGGGTTTAGGGTTTAGGGTTTAGGGTTTAGGGTTTAGGGTTTAGGGTTTAGGGTTTAGGGTTTAGGGTTTAGGGTTTAGGGTTAGGGTTTAGGGTTTAGGGTTTAGGTTTAGGGTTTAGGGTTTAGGGTTTAGGGTTTAGGGTTTAGGGTTAGGGTTTAGGGTTTAGGGTTTAGGGGTTTAGGGTTTAGGGTTTAGGGTTTAGGGTTTAGGGTTTAGGGTTTGGGTTTAGGGTTTAGGGTTTAGGGTTTAGGGTTTAGGGTTTAGGGTTTAGGGTTAGGGTTTAGGGTTTAGGGTTTAGGGTTTAGGGTTTAGGGTTTAGGGTTTAGGGTTTAGGGTTTAGGGTTTAGGGTTTAGGGTTTAGGGTTTAGGTAGGGTTTAGGGTTTAGGGTTTAGGGTTTAGGGTTTAGGGTTTAGGGTTAGGGTTTAGGGTTTAGGGTTTAGGTTTAGGGTTTAGGGTTTAGGGTTTAGGGTTTAGGGTTTAGGGTTAGGGTTTAGGGTTTAGGGTTTAGGGTTTAGGGTTTAGGGTTTAGGGTTTAGGGTTTAGGGTTTAGGGTTTAGGGTTTAGGGTTTAGGGTTTAGGGTTAGGGTTTAGGGTTTAGGGTTTAGGGTTAGGGTTTAGGGTTTAGGGTTTAGGGTTTAGGGTTTAGGGTTAGGGTTTAGGGTTTAGGGTTTAGGGTTTAGGGTTTAGGGTTTAGGGTTTAGGGTTAGGGTTTAGGGTTTAGGGTTTAGGGTTTAGGGTTTAGGGTTTAGGGTTTAGGGTTTAGGGTTTAGGGTTAGGGTTAGGGTTTAGGGTTTAGGGTTTAGGGTTTAGGGTTAGGGTTTAGGGTTTAGGGTTTAGGGTTTAGGGTTTAGGGTTTAGGGTTTAGGGTTTAGGGTTAGGGTTTAGGGTTTAGGGTTTAGGGTTTAGGGTTTAGGGTTTAGGGTTTAGGGTTTAGGGTTTAGGGTTAGGGTTTAGGGTTTAGGGTTTAGGGTTTGGTTAGGGTTTAGGGTTTAGGGTTTAGGGTTTAGGGTTAGGGTTTAGGGTTTAGGGTTTAGGGTTTAGGGTTAGGGTTTAGGGTTTAGGGTTTAGGGTTAGGGTTTAGGGTTTAGGGTTTAGGGTTTAGGGTTTAGGGTTTAGGGTTTAGGGTTTAGGGTTAGGGTTTAGGGTTTAGGGTTAGGGTTTAGGGTTAGGGTTTAGGGTTTAGGGTTTAGGGTTTAGGGTTTAGGGTTTAGGGTTTAGGGTTAGGGTTTAGGGTTTAGGGTTTAGGGTTTAGGGTTTAGGGTTTGGGTTAGGGTTTAGGGTTTAGGGTTTAGGGTTTAGGGTTTAGGGTTTAGGGTTTAGGGTTTAGGGTTTAGGGTTTAGGGTTTAGGGTTTAGGGTTTAGGGTTTAGGGTTGGGTTAGGGTTTAGGGTTTAGGGTTTAGGGTTTAGGGTTTAGGGTTTAGGGTTAGGGTTTAGGGTTTAGGGTTTAGGGTTTAGGGTTTAGGGTTTAGGGTTTAGGGTTAGGGTTTAGGGTTAGGGTTTAGGGTTTAGGGTTGGTAGGGTTTAGGGTTTAGGGTTTAGGGTTTAGGGTTTAGGGTTTAGGGTTTAGGGTTTAGGGTTTAGGGTTTAGGGTTTAGGGTTTAGGGTTTAGGGTTTAGGGTTTAGGGTTAGGGTTTAGGGTTTAGGGTTTAGGGTTTAGGGTTAGGGTTTAGGGTTTAGGGTTTAGGGTTTAGGGTTTAGGGTTAGGGTTTAGGGTTAGGGTTTAGGGTTTAGGGTTTAGGGTTTAGGGTTTAGGGTTTAGGGTTTAGGGTTTAGGGTTTAGGGTTTAGGGTTTAGGGTTTAGGGTTTAGGGTTTAGGGTTTAGGGTTTAGGGTTTAGGGTTAGGGTTTAGGGTTTAGGGTTTAGGGTTTAGGGTTTAGGGTTTAGGGTTTAGGGTTAGGGTTTAGGGTTTAGGGTTTAGGGTTTAGGGTTTAGGGTTTAGGGTTTAGGGTTTAGGGTTTAGGGTTTAGGGTTTAGGGTTTAGGGTTTAGGGTTAGGGTTTAGGGTTTAGGGTTTAGGGTTAGGGTTTAGGGTTAGGGTTAGGGTTTGGGTTTAGGGTTTAGGGTTTAGGGTTTAGGGTTTAGGGTTTAGGGTTTAGGGTTTAGGGTTTAGGGTTTAGGGTTTAGGGTTTAGGGTTTAGGGTTTAGGGTTTAGGGTTTAGGGTTTAGGGTTTAGGGTTTAGGGTTTAGGGTTAGGGTTTAGGGTTTAGGGTTTAGGGTTTAGGGTTTAGGGTTTAGGGTTTAGGGTTTAGGGTTTAGGGTTTAGGGTTTAGGGTTTGGGTTTAGGGTTTAGGGTTAGGGTTTAGGGTTTAGGGTTTAGGGTTTAGGGTTTAGGGTTTAGGGTTTAGGGTTTAGGGTTTAGGGTTTAGGGTTTAGGGTTAGGGTTTAGGGTTTAGGGTTTAGGGTTTAGGGTTTAGGGGTTTAGGGTTTAGGGTTTAGGGTTTAGGTTTAGGGTTTAGGGTTTAGGGTTTAGGGTTTAGGGTTTAGGGTTTAGGGTTTAGGGTTTAGGGTTTAGGGTTTAGGGTTTAGGGTTTAGGGTTTAGGGTTTAGGGTTAGGGTTTAGGGTTTAGGGTTTAGGGTTTAGGGTTTAGGGTTTAGGGTTTAGGGTTAGGGTTTAGGGTTTAGGGTTTAGGGTTTAGGGTTTAGGGTTTAGGGTTTAGGGTTTAGGGTTTAGGGTTTAGGGTTAGGGTTAGGGTTTAGGGTTTAGGGTTTAGGGTTTAGGGTTTAGGGTTTAGGGTTTAGGGTTTAGGGTTAGGGTTTAGGGTTTAGGGTTTAGGGTTTAGGGTTTAGGGTTTAGGGTTAGGTTTAGGGTTTTAGGGTTTAGGGTTTAGGGTTTAGGGTTTAGGGTTTAGGGTTTAGGGTTTAGGGTTTAGGGTTAGGGTTTAGGGTTTAGGGTTTAGGGTTAGGGTTTAGGGTTTAGGGTTTAGGGTTTAGGGTTTAGGGTTTAGGGTTAGGGTTTAGGGTTTAGGGTTTAGGGTTTAGGGTTTAGGGTTAGGGTTTAGGGTTAGGGTTTAGGGTTTAGGGTTTAGGGTTTAGGGTTTAGGGTTTAGGGTTTAGGGTTTAGGGTTTAGGGTTTAGGGTTTAGGGTTTAGGGTTTAGGGTTTAGGGTTAGGGTTTAGGGTTTAGGGTTTAGGGTTTAGGGTTTAGGGTTTAGGGTTTAGGGTTTAGGGTTTAGGGTTTAGGGTTTAGGGTTTAGGGTTTAGGGTTTAGGGTTTAGGGTTTAGGGTTTAGGGTTTAGGGTTTAGGGTTAGGGTTTAGGGTTAGGGTTTAGGGTTTAGGGTTTAGGGTTTAGGGTTTAGGGTTTAGGGTTTAGGGTTAGGGTTTAGGGTTTAGGGTTTAGGGTTTAGGGTTTAGGGTTTAGGGTTTAGGGTTTAGGGTTTAGGGTTTAGGGTTTAGGGTTTAGGGTTAGGGTTTAGGGTTTAGGGTTTAGGTTTAGGGTTTAGGGTTTAGGGTTAGGGTTTAGGGTTTAGGGTTTAGGGTTTAGGGTTAGGGTTTAGGGTTTAGGGTTAGGGTTTAGGGTTTAGGGTTTAGGGTTTAGGGTTTAGGGTTTAGGGTTTAGGGTTTAGGGTTTAGGGTTGGTAGGGTTTAGGGTTTAGGGTTTAGGGTTTAGGGTTTAGGGTTTAGGGTTTAGGGTTTAGGGTTTAGGGTTTAGGGTTTAGGGTTTAGGGTGGTTAGGGTTTAGGGTTTAGGGTTTAGGGTTTAGGGTTTAGGGTTAGGGTTTAGGGTTTAGGGTTTAGGGTTTAGGGTTAGGTTAGGGTTTAGGGTTTAGGGTTTAGGGTTTAGGGTTTAGGGTTAGGGTTTTAGGGGTTTAGGGTTTAGGGTTTAGGGTTTAGGGTTTAGGGTTTAGGGTTAGGGTTTAGGGTTTAGGGTTTAGGGTTAGGGTTTAGGGTTTAGGGTTAGGTTTAGGGTTTAGGGTTTAGGGTTTAGGGTTTAGGGTTTAGGGTTTAGGGTTAGGGTTTAGGGTTTAGGGTTTAGGGTTTAGGGTTTAGGGTTTAGGGTTTAGGGTTTAGGGTTAGGGTTTAGGGTTTAGGGTTTAGGGTTTAGGGTTAGGGTTTAGGGTTTAGGGTTTAGGGTTAGGTTAGGGTTAGGGTTTAGGGTTTAGGGTTTAGGGTTTAGGGTTTAGGGTTTAGGGTTTAGGGTTTAGGGTTTAGGGTTTAGGGTTTAGGGTTTAGGGTTTAGGGTTAGGGTTTAGGGTTTAGGGTTAGGGTTTAGGGTTTAGGGTTTAGGGTTAGGGTTTAGGGTTTAGGGTTTAGGGGTTTAGGGTTTAGGGTTTAGGGTTTAGGGTTTAGGGTTTAGGGTTTAGGGTTTAGGTTAGGGTTTAGGGTTTAGGGTTGGGTTTAGGGTTTAGGGTTTAGGTTTAGGGTTTAGGGTTTAGGGTTTAGGGTTTAGGGTTAGGGTTTAGGGTTTAGGGTTTAGGGTTTAGGGTTTAGGGTTTAGGGTTAGGGTTTAGGGTTTAGGGTTTAGGGTTTAGGGTTTAGGGTTTAGGGTTTAGGGTTTAGGGTTTAGGGTTTAGGGTTTAGGGTTTAGGGTTTAGGGTTTAGGGTTTAGGGTTTAGGGTTTAGGGTTTAGGGTTTAGGGTTAGGTTAGGGTTTAGGGGTTTAGGGTTTAGGGTTTAGGGTTTAGGGTTTAGGGTTTAGGGTTTAGGGTTAGGGTTTAGGGTTTAGGGTTTAGGGTTTAGGGTTAGGGTTTAGGGTTTAGGGTTAGGGTTTAGGGTTTAGGGTTTAGGGTTTAGGGTTTAGGGTTTAGGGTTTAGGGTTTAGGGTTTAGGGTTAGGGTTTAGGGTTTAGGGTTAGGGTTTAGGGTTTAGGGTTAGGGTTAGGGTTTAGGGTTTAGGGTTTAGGGTTTAGGGTTTAGGGTTTAGGGTTTAGGGTTTAGGGTTTAGGGTTTAGGGTTTAGGGTTTAGGGTTAGGGTTTAGGGTTTAGGGTTTAGGGTTTAGGGTTTAGGGTTTAGGGTTAGGGTTTAGGGTTTAGGGTTTAGGGTTAGGGTTTAGGGTTTAGGGTTTAGGGTTTAGGGTTTAGGGTTTAGGGTTAGGGTTTAGGGTTTAGGGTTTAGGGTTTAGGGTTTAGGTTTAGGGTTAGGGTTAGGGTTTAGGGTTTAGGGTTTAGGGTTTAGGGTTAGGTTAGGGTTTAGGGTTTAGGGTTTAGGGTTTAGGGTTAGGGTTTAGGGTTTAGGGTTAGGGTTTAGGGTTTAGGGTTAGGGTTTAGGGTTTAGGGTTTAGGGTTTAGGGTTTAGGGTTTAGGGTTAGGGTTTAGGGTTTAGGGTTTAGGGTTTAGGGTTTAGGGTTAGGGTTTAGGGTTTAGGGTTTAGGGTTTAGGGTTTAGGGTTTAGGGTTTAGGGTTTAGGGTTTTAGGGTTTAGGGTTTAGGGTTAGGGTTTAGGGTTTAGGGTTTAGGGTTTAGGGTTTAGGGTTAGGGTTTAGGGTTTAGGGTTTAGGGTTTAGGGTTTAGGGTTTAGGGTTTAGGGTTTAGGGTTTAGGGTTTAGGGTTTAGGGTTTAGGGTTTAGGGTTTAGGGTTTAGGGTTTAGGGTTTAGGGTTAGGGTTTAGGGTTTAGGGTTTAGGGTTTAGGGTTTAGGGTTTAGGGTTTAGGGTTTAGGGTTTAGGGTTAGGGTTTAGGGTTTAGGGTTTAGGGTTTAGGGTTTAGGGTTTAGGGTTTAGGGTTTAGGGTTAGGGTTTAGGGTTAGGGTTTAGGGTTTAGGGTTAGGGTTTAGGGTTAGGGTTTAGGGTTAGGGTTTAGGGTTTAGGGTTTAGGGTTAGGGTTTAGGGTTAGGGTTTAGGGTTTAGGGTTTAGGGTTTAGGGTTTAGGGTTTAGGGTTTAGGGTTTAGGGTTTAGGGTTTAGGGTTTAGGGTTAGGGTTTAGGGTTTAGGGTTAGGGTTTAGGGTTTAGGGTTAGGGTTTAGGGTTTAGGGTTTAGGGTTTAGGGTTTAGGGTTTAGGGTTTAGGGTTTAGGGTTTAGGGTTTAGGGTTTAGGGTTTAGGGTTTAGGGTTTAGGGTTTAGGGTTTAGGGTTTGGGTTTAGGGTTTAGGGTTTAGGGTTTTGGGTTTAGGGTTTAGGGGTTTAGGGTTTAGGGTTTAGGGTTTAGGGTTTAGGGTTTAGGGTTTAGGGTTTAGGGTTAGGGTTTAGGGTTAGGGTTTAGGGTTAGGGTTTAGGGGTTTAGGGTTAGGGTTTTAGGGTTTAGGGTTTAGGGTAGGGTTTAGGGTTTAGGGTTTAGGGTTTAGGGTTTAGGGTTTAGGGTTGTGGGTTTAGGGTTTTAGGGTTTTAGGGTTTAGGGGTTTAGGGTTTAGGGGTTTAGGGTTAGGGTTTAGGGTTTAGGGTTTGGGTTAGGGTTTAGGGTTTAGGGTTAGGGTTTAGGGTTAGGGTTTAGGGTTTAGGGTTTAGGGTTTTTAGGGTTTAGGTTTAGGGTTTAGGGTTTGGGATTAGGGATTAGGGTTTAGGGTTTAGGGTTTAGGGTTTAGGGTTTAGGGTTA

**6DL**

CACTTAGGG**TTTAGGG**TTTAGGGTTTAGGGTTGGGGTTTAGGGTTTAGGGTTTATGGGTTTAGGGTTGTAGGGTTTAGGGTTTAGGGTTTAGGGTTTAGGGTTTAGGGTTAGGGTTAGGGTTTAGGGTTTAGGGTTTAGGGTTTAGGGTTTAGGGTTTAGGGTTTAGGGTTTAGGGTTTAGGGTTTAGGGTTAGGGTTTAGGGTTTAGGGTTTAGGGTTTAGGGTTTAGGGGTTAGGGTTTAGGGTTTAGGGTTTAGGGTTTAGGGTTTAGGGTTTAGGGTTTAGGGTTTAGGGTTTAGGGTTTAGGGTTTAGGGTTTAGGGTTTAGGGTTTAGGGTTTAGGGTTTAGGGTTAGGGTTTAGGGTTAGGGTTTAGGGTTTAGGGTTTAGGGTTTAGGGGTTTAGGGTTTAGGGTTTAGGGTTAGGGTTTAGGGTTAGGGTTAGGGTTTAGGGTTTAGGGTTTAGGGTTAGGGTTTAGGGTTTAGGGTTTAGGGTTTAGGGTTTAGGGTTTAGGGTTGGGTTTAGGGTTTAGGGTTTAGGGTTTAGGGTTTAGGGTTTAGGGTTAGGGTTGTTAGGGTTTAGGGTTAGGGTTTAGGGTTAGGGTTTAGGGTTTAGGGTTTAGGGTTTAGGGTTAGGGGTTAGGGTTAGGGTTAGGGTTAGGGTTAGGGTTTAGGGTTTTTAGGGTTTAGGGTTAGGGTTTAGGGTTTAGGGTTAGGGTTAGGGTTTAGGGTTTAGGGTTAGGGTTTAGGGTTAGGGTTTAGGGTTTAGGGTTTAGGATTAGGGTTTAGGGTTAGGGTTTAGGGTTAGGGTTAGGGTTTAGGGTTTAGGGTTAGGGTTTAGGGTTTAGGGTTTAGGGTTTAGGGTTTAGGGTTTAGGGTTTAGGGTTAGGGTTTAGGGTTTAGGGTTTAGGGTTTAGGGTTTAGGGTTTAGGGTTTAGGGTTTAGGGTTTTAGGGTTTAGGGTTTAGGGTTTAGGGTTTAGGGTTTAGGGTTTAGGGTTTAGGGTTAGGGTTAGAGGGTTAGGGTTAGGGTTTAGGGTTTAGGGTTTAGGGTTAGGGTTTAGGGTTTAGGGTTTAGGGTTAGGGTTTAGGGTTTAGGGTTTAGGGTTAGGGTTTAGGGTTAGGGTTTAGGGTTTAGGGTTAGGGTTAGGGTTTAGGGTTTAGGGTTTAGGGTTTAGGGTTTAGGGTTTAGGGTTTAGGGTTTAGGGTTTAGGGTTTAGGGTTTTAGGGTTTAGGGTTTAGGGTTAGGGTTAGGGTTTAGGGTTTAGGGTTTAGGGTTAGGGTTAGGGTTTAGGGTTTAGGGTTTAGGGTTAGGGTTTAGGGTTTAGGGTTTAGGGTTTAGGGTTTAGGGTTAGGGTTTGGGTTAAGGGTTTAGGGTTTAGGGTTTAGGGTTTAGGGTTTAGGGTTTAGGGGTTTAGGGTTAGGTTAGGGTTTAGGGGTTTAGGGTTTAGGGTTTAGGGTTTAGGGTTAGGGTTTAGGGTTTAGGGTTAGGGTTTAGGGTTTAGGGTTTAGGGTTTAGGGTTTAGGGTTTAGGGTTTAGGGTTTAGGGTTAGGGTTTAGGGTTTAGGGTTTAGGGTTTAGGGTTTAGGGTTAGGGTTTAGGGTGTAGGGTTTAGGGTTTAGGGTTTAGGGTTTAGGGTTTAGGGTTTAGGGTTTAGGGTTTAGGGTTTAGGGTTAGGGTTTAGGGTTTAGGGTTTAGGGTTTAGGGTTTAGGGTTTAGGGTTTAGGGTTTAGGGTTAGGGTTTAGGGTTTAGGGTTTAGGGTTTAGGGTTAGGGTTAGGGTTTAGGGTTTAGGGTTTAGGGTTAGGGTTTAGGGTTTAGGGTTAGGGTTAGGGTTTAGGGTTTAGGGTTTAGGGTTTAGGGTTTAGGGTTAGGGTTTAGGGTTTAGGGTTAGGGTTAGGGTTTAGGGTTTAGGGTTAGGGTTTAGGGTTTAGGGGTTTAGGGTTTAGGGTTAGGGTTTAGGGTTTAGGGTTTAGGGTTAGGGTTTAGGGTTTAGGGTTTAGGGTTTAGGGTTTAGGGTTTAGGGTTAGGGTTTAGGGTTTAGGGTTTAGGGTTAGGGTTAGGGTTTGTTAGGTTTAGGGTTTAGGGTTTGGGTTTAGGGTTTAGGGTTTAGGGTTTAGGGTTTAGGGTTTGGGTTTAGGGTTTAGGGTTTAGGGTTTAGGGTTTAGGGTTTAGGGTTTAGGGTTTAGGGTTAGGGTTTAGGGTTTAGGGTTTAGGGTTTAGGGTTTAGGGTTAGGGTTTAGGGTTAGGGTTAGGGTTTAGGGTTTAGGGTTTAGGGTTTAGGGTTTAGGGTTTAGGGTTTAGGGTTTAGGGTTTAGGGTTTAGGGTTTAGGGTTTGGGTTTAGGGTTTAGGGTTTAGTGGTTTAGGGTTTAGGGTTTAGGGTTTAGGGTTTAGGGTTTAGGGTTAGGGTTTAGGGTTTAGGGTTTAGGGTTAGGGTTAGGGTTTAGGGTTTTAGGGTTTAGGGTTTAGGGTTTAGGGTTTAGGGTTTAGGGTTTTAGGGTTTAGGGGTTTAGGGTTTAGGGTTTAGGGTTTAGGGTTTAGGGTTTAGGGTTTAGGGTTTAGGGTTTAGGGTTAGGGTTTAGGGTTTAGGGTTTAGGGTTTAGGGTTTAGGGTTTAGGGTTTAGGGTTTAGGGTTTAGGGTTTAGGGTTTAGGGTTTAGGGTTAGGGTTTAGGGTTTAGGGTTTAGGGTTAGGGTTTAGGGTTTAGGGTTTAGGGTTTAGGGTTTAGGGTTAGGGTTAGGGTTTAGGGTTTAGGGTTTAGGGTTTAGGGTTTAGGGTTTAGGGTTTAGGGTTTAGGGTTTAGGGTTAGGGTTTAGGGTTTAGGGTTTAGGGTTTAGGGTTTTAGGGTTTAGGGTTTAGGGTTTTAGGGTTTAGGGTTAGGGTTTAGGGTTTAGGGTTTAGGGTTTAGGGTTTAGGGTTTAGGGTTAGGGTTTAGGGTTTAGGGTTTAGGGTTTAGGGTTTAGGGTTTAGGGTTTAGGGTTTAGGGTTTAGGGTTTAGGGTTAGGGTTTAGGGTTTAGGGTTTAGGGTTTAGGGTTTAGGGTTTAGGGTTTAGGGTTAGGGTTAGGGTTTAGGGTTTAGGGTTTAGGGTTTAGGGTTTAGGGTTTAGGGTTTATTTAGGGTTTAGGGTTTAGGGTTTAGGGTTTGGGGTTTGGGTTTTTAGGGTTTAGGGTTTAGGGTTTAGGGTTTAGGGTTTAGGGTTTAGGGTTTAGGGTTTAGGGTTTAGGGTTTAGGGTTAGGGTTTAGGGTTTAGGGTTTAGGGTTTAGGGTTTAGGGTTAGGGTTTAGGGTTTAGGGTTTAGGGTTTAGGGTTTAGGGTTTAGGGTTTAGGGTTTAGGGTTTAGGGTTTAGGGTTTAGGGTTTAGGGTTTAGGGTTTAGGGTTAGGGTTAGGGTTTAGGGTTAGGTTAGGGTTTAGGGTTTAGGGTTTAGGGTTTAGGGTTTAGGGTTTAGGGTTTAGGGTTTAGGGTTTAGGGTTAGGGTTTAGGGTTTAGGGTTTAGGTTTAGGGTTTAGGGTTTAGGGTTAGGGTTTAGGGTTTAGGGTTTAGGGTTTAGGGTTAGGGTTTAGGGTTTAGGGTTTAGGGTTTAGGGTTTAGGGTTTAGGGTTTAGGGTTTAGGGTTTAGGGTTTAGGGTTTAGGGTTAGGGTTTAGGGTTAGGGTTTAGGGTTTAGGGTTTAGGGTTTAGGGTTAGGGTTTAGGGTTTAGGGTTTAGGGTTTAGGGTTTAGGGTTTAGGGTTTAGGGTTTAGGGTTTAGGGTTTAGGGTTTAGGTTTAGGGTTTAGGGTTTAGGGTTTAGGGTTTAGGGTTAGGGTTTAGGGTTTAGGGTTTAGGGTTTAGGGTTTAGGGTTAGGGTTTAGGGTTTAGGGTTTAGGGTTTAGGGTTTAGGGTTTAGGGTTTAGGGTTTAGGGTTTAGGGTTTGGGGTTTAGGGTTTAGGGGTTTAGGGTTAGGGTTTAGGGTTTAGGGTTTAGGGTTTAGGGTTTAGGGTTTAGGGTTTTAGGGTTTAGGGTTTAGGGTTTAGGGTTTAGGGTTAGGGTTTAGGGTTTAGGGTTTAGGGTTTAGGGTTTAGGGTTTAGGGTTTAGGGTTTAGGGTTTAGGGTTTAGGGTTTAGGGTTTAGGGTTTAGGGTTTAGGGTTTAGGGTTTAGGGTTAGGGTTAGGGTTTAGGGTTTAGGGTTAGGGTTTAGGGTTTAGGGTTTGGGTTTAGGGTTAGGGTTTAGGGTTTAGGGTTTTAGGGTTTAGGGTTTAGGGTTTAGGGTTTAGGGTTTAGGGTTTAGGGTTTAGGGTTTAGGGTTTAGGGTTTTAGGGTTTAGGGTTTAGGGTTTAGGTTTAGGGTTTAGGGTTTAGGGTTTAGGGTTTAGGGTTTAGGGTTTAGGGTTTAGGGTTTAGGGTTTAGGTTAGGGTTTAGGGTTTAGGGTTTAGGGTTTAGGGTTTAGGGTTTAGGGTTAGGGTTTAGGGTTTAGGGTTTAGGGTTTAGGGTTAGGGGTTTAGGGTTTAGGGTTTAGGGTTTAGGGTTTAGGGTTTAGGGTTTAGGGGTTTAGGGTTTAGGGTTTAGGGTTTAGGGTTTAGGGTTTAGGGTTTAGGGTTTAGGGTTTAGGGTTTAGGGTTTAGGGTTTAGGGTTTAGGGTTTAGGGGTTTAGGGTTTAGGGTTTAGGGTTTAGGGTTTAGGGTTTAGGGTTTAGGGTTTAGGGTTTAGGGTTTAGGGTTTAGGGTTTAGGTTTAGGGTTTAGGGTTTAGGGTTAGGGTTTAGGGTTTAGGGTTTAGGGTTTAGGGTTTAGGGTTTAGGGTTTAGGGTTTAGGGTTTAGGGTTTAGGGTTTAGGGTTAGGGTTTAGGGTTTAGGGTTTAGGGTTTAGGGTTTAGGGTTTAGGGTTTAGGGTTAGGGTTAGGGTTAGGGGTTTAGGGTTTAGGGTTTAGGGTTTAGGGTTTAGGGTTTTAGGGTTTAGGGTTAGGGTTAGGGTTTAGGGTTTAGGGGTTTAGGGTTGGTTTAGGGTTAGGGTTTAGGGTTTAGGGTTAGGGTTTAGGGTTTAGGGTTGGTTAGGGGTTTAGGGTTTAGGGTTTAGGGTTTAGGGTTTAGGGTTTAGGGTTTAGGGTTTAGGGTTTAGGGTTTAGGGTTAGGGTTTAGGGTTTAGGGTTAGGGTTTAGGGTTTAGGGTTTAGGGTTTAGGTTGGTTTAGGGTTTAGGGTTTAGGGTTTAGGGTTTAGGGTTTAGGGGTTTAGGGTTTAGGGTTTAGGGTTAGGTTTAGGGTTTAGGGGTTTAGGGTTAGGGTTTAGGGTTAGGGTTTAGGGTTTAGGGTTTAGGGTTTAGGGTTTAGGGTTTAGGGTTTAGGGTTTAGGGTTTAGGGTTTAGGGTTTAGGGTTTAGGGTTAGGGTTTAGGGTTTAGGGTTTAGGGGTTTAGGGTTTAGGGTTTTAGGGTTAGGGTTTAGGGTTAGGGTTTAGGGTTTAGGGTTTAGGGTTAGGGTTTAGGGTTTAGGGTTTAGGGTTTAGGGTTAGGGTTAGGGTTAGGGTTTAGGGTTTAGGGTTTAGGGTTTAGGGTTTAGGGTTTAGGGTTTAGGGTTTAGGTTTAGGGTTTAGGGTTTAGGGTTTAGGGTTTAGGGTTTAGGGTTAGGGTTTAGGGTTAGGGTTAGGGTTTAGGGTTTAGGGTTAGGGTTTAGGGTTTAGGGTTTAGGGTTAGGTTTAGGGTTTAGGGTTTTAGGGTTAGGGTTTAGGGTTTAAGGGTTTAGGGTTTAGGGTTTAGGGGTTAGGGTTTAGGGTTTAGGGTTTAGGGTTATAGGGTTTAGGGTTAGGTTTTAGGGTTTAGGGTTTAGGGTTTAGGTTAGGGTTTAGGGTTTAGGGTTTAGGGTTTAGGGTTTAGGGTTTAGGGTTTAGGGTAGGTTAGGGTTTAGGGTTTAGGGTTTAGGGTTTAGGGTTTAGGTTTTAGGGTTTAGGGTTTAGGGTTTAGGGTTAGGGTTTAGGGTTTAGGGTTAGGGTTTAGGGTTTAGGGTTTAGGGTTTAGGGTTTAGGGTTTAGGGTTTAGGGTTAGGGTTTAGGGTTTAGGGTTTAGGGTTTAGGGTTTAGGGTTTAGGGTTTAGGGTTTAGGGTTTAGGGTTTAGGGTTTAGGGTTTAGGGTTTAGGGTTAGGGTTTAGGGTTTTAGGGTTTAGGGTTTAGGGTTTAGGGTTTAGGGTTTAGGGTTAGGGTTTAGGGTTTAGGGTTTAGGGTTTAGGGTTTAGGGTTTAGGGTTTAGGGTTTAGGGTTTAGGGTTTAGGGTTTAGGGTTTAGGGTTAGGGTTTAGGGTTTAGGGTTTAGGGTTTAGGGTTTAGGGTTTAGGGTTTAGGGTTTAGGGTTTAGGGTTTAGGGTTAGGGTTTAGGGTTTAGGGTTTAGGGTTTAGGGTTTAGGGTTTAGGGTTTAGGGTTTAGGGTTAGGGTTTTTTAGGGTTTAGGGTTTAGGGTTTAGGGTTTAGGGTTAGGTTTAGGGTTTAGGGTTTAGGGTTTAGGGTTAGGTTAGGGTTTAGGGTTTAGGGTTTAGGGTTTAGGGTTTAGGGTTTTAGGGTTAGGGTTTAGGGTTTTAGGGTTTAGGGTTAGGGTTTAGGGTTTAGGGTTTAGGGTTTTAAGGGTTAGGGTTTAGGGGTTTAGGGTTTAGGGTTTAGGGTTTAGGGTTTAGGGTTTAGGGTTTAGGGTTTAGGGTTAGGGTTTTTAGGGTTTAGGGTTTAGGGTTTAGGGTTAGGGTTTAGGGTTTAGGGTTTAGGGTTTAGGGTTTAGGGTTTAGGGTTTAGGGTTTGGGTTTAGGGTTAGGGTTTAGGGTTTAGGGTTAGGGTTTAGGGTTTAGGGTTTAGGGTTTAGGGTTTAGGGTTTAGGGTTTAGGGTTAGGGTTTAGGGTTTAGGGGTTAGGGTTTAGGGTTTAGGGTTAGGGTTAGGGTTTAGGGTTTAGGGTTTAGGGTTTAGGGTTTAGGGTTTAGGGTTAGGGTTTAGGGTTTTGGGTTTAGGGTTTAGGGTTTAGGGTTTAGGGTTAGGGTTAGGGTTTAGGGTTAGGGTTTAGGGTTTAGGGTTTTAGGGTTAGGGTTTAGGGTTTAGGGTTTAGGTTTAGGGTTTAGGGTTTAGGGTTTAGGGTTTAGGGTTTAGGGTTTAGGGTTAGGGTTAGGTTAGGGTTTAGGGTTAGGGTTTAGGGTTAGGGTTTTTTTTGGTAGGGTTAGGGTTTAGGGTTTAGGGGTTTAGGGTTTAGGGTTTAGGGTTTAGGGTTAGGGTTAGGGTTAGGTTTAGGGTTTAGGGTTTAGGGTTTAGGGTTAGGTTAGGGTTTAGGGTTTAGGGTTTAGGGTTTAGGGTTTAGGGTTTAGGGTTTAGGGTTTTAGGGTTTAGGGTTAGGGTTTAGTAGGGTTAGGGTTTAGGGTTTAGGGTTAGGGTTTAGGGTTTAGGGTTTAGGGTTAGGGTTTAGGGTTTAGGGTTTAGGGTTTAGGGTTAGGGTTTAGGGTTTAGGGTTAGGGGTTTAGGGTTAGGGTTTAGGGTTAGGGTTTTAGGGTTTAGGGTTAGTAGGGTTTAGGGTTTAGGGTTTAGGGTTAGGGTTTAGGGTTAGGGTTTAGGGTTTAGGGTTAGGGTTTAGGGTTTAGGGTTTAGGGTTAGGGTTTAGGGTTTAGGGTTTAGGGTTTAGGGTTTAGGGTTAGGGTTAGGGTTTGGTTAGGGTTTAGGGTTTAGGGTTTAGGGTTTAGGGTTTTAGGGTTTAGGGTTAGGGTTTAGGGTTTAGGGTTTAGGGTTTAGGGTTTAGGGTTTAGGGTTTAGGGTTTAGGGTTTAGGGTTTAGGGTTTAGGGTTTAGGGTTTAGGGTTTAGGGTTTAGGTTAGGGTTTAGGGTTTAGGGTTTAGGGTTTAGGGTTTGGGTTTAGGGGTTTAGGGTTTAGGGTTTAGGGTTTAGGGTTTAGGGTTTAGGGTTTAGGGTTTAGGGTTTAGGGTTTAGGGTTTAGGGTTTAGGGTTTAGGGTTTAGGGTTTAGGGTTTGGGTTAGGGTTTAGGGTTTAGGTAGGTTTAGGGTTAGGGTTTAGGGTTTAGGGTGGTTTAGGGTTTAGGGTTTAGGGTTTAGGGTTTAGGGTTTAGGGTTAGGGTTTAGGGTTTAGGGTTTAGGGTTTAGGGTTTAGGGTTTAGGGTTTAGGGTTTAGGGTTTAGGGTTTAGGGTTTAGGGTTTAGGGTTTAGGGTTAGGGTTTAGGGTTTAGGGTTTAGGGTTTAGGGTTTAGGGTTTTAGGGGTTTAGGGTTTAGGGTTTAGGGTAGGTTTAGGGTTTAGGGTTTAGGGTTTAGGGTTTAGGGTTAGGGTTTAGGGTTTAGGGTTTAGGGTTTAGGGTTTAGGGTTTAGGGTTTAGGGTTTAGGGTTAGGGTTTAGGGTTTAGGGTTTAGGGTTTAGGGTTTAGGGTTTAGGTTTAGGGTTTAGGGTTTAGGGTTAGGGTTTTAGGGTTTAGGGTTTAGGGTTTAGGGTTAGGGTTTAGGGTTTAGGGTTTAGGGTTAGGGTTTAGGGTTTAGGGTTAGGGTTTAGGGTTTAGGGTTTAGGGTTAGGGTTTAGGGTTTAGGGTTTAGGGTTTAGGGTTTAGGGTTTAGGGTTTAGGGTTTAGGGTTTAGGGTTAGGGTTTAGGGTTTAGGGTTTAGGGTTTAGGGTTTAGGGTTAGGGTTAGGGTTTAGGGTTTAGGGTTTAGGGTTTAGGGTTTAGGGTTTAGGGTTAGGGTTTAGGGTTTAGGGTTAGGGTTTAGGGTTAGGGTTTAGGGTTAGGGTTAGGGTTTAGGGTTTAGGGTTTAGGGTTTAGGGTTTAGGGTTTAGGGGTTTAGGGTTTAGGGTTTAGGGTTAGGGTTTAGGGTTTAGGGTTTAGGGTTTAGGGTTAGGGTTTAGGGTTAGGGTTTAGGGTTTAGGGTTTAGGGTTTAGGGTTTAGGGTTTAGGGTTTAGGGTTTAGGTTTAGGGTTTAGGGTTTTAGGGTTTAGGGTTTAGGGTTAGGGTTTAGGGGTTAGGGTTTAGGGTTTAGGGTTTAGGGTTTAGGGTTAGGGTTAGGGTTTAGGGTTTAGGGTTTAGGGTTTTTAGGGTTTAGGGTTAGGGTTTAGGGTTTAGGGTTTAGGGTTAGGGTTTAGGGTTTTAGGGTTTAGGGTTTGGTTTAGGGTTTTAGGGTTTAGGGTTTAGGTTTAGGGTTTAGGGTTTAGGTTTAGGGTTTTTAGGGTTTAGGGTTTAGGGTTTAGGGTTTAGGGTTTAGGGTTTAGGGTTTAGGGTTTAGGGTTAGGGTTTAGGGTTTAGGGTTTAGGGTTAGGGTTTAGGGTTTAGGGTTTAGGGTTTAGGGTTTAGGGTTAGGGTTTAGGGTTTAGGGTTAGGGTTTAGGGTTTTTAGGGTTTAGGGTTTAGGGTTTAGGGTTTAGGGTTTAGGGTTTAGGGTTTAGGGTTTTAGGGTTAGGGTTTAGGGTTTAGGGTTTAGGGTTTAGGGTTTAGGGTTTAGGGTTTAGGGTTTAGGGTTTAGGGTTTAGGGTTTAGGGTTAGGGTTTAGGGTTTAGGGTTTAGGGTTTAGGGTTTAGGGTTTAGGGTTAGGGTTTAGGGTTTAGGGTTTTTAGGGTTTAGGGTTAGGGTTTAGGGTTTAGGGTTTAGGGTTTAGGGGTTAGGGTTTAGGGTTAGGGTTTAGGGTTTAGGGTTTAGGGTTTAGGGTTTAGGGTTTAGGGTTTAGGGTTTAGGGTTTAGGGTTTAGGGTTTAGGGTTTAGGGTTTAGGGTTAGGGTTTAGGGTTTAGGGTTTAGGGTTTAGGTTTAGGGGTTTAGGGTTTAGGGTTTAGGGTTTAGGGTTTAGGGTTTAGGGTTTAGGGTTTAGGGTTTAGGGTTTAGGGTTTAGGGTTTAGGGTTAGGGTTTAGGGTTTAGGGTTTAGGGTTAGGGTTTAGGGTTTAGGGTTTAGGGTTTAGGGTTTAGGGTTTAGGGTTTAGGGTTTAGGGTTTAGGGTTTAGGGTTTAGGGTTAGGGTTTAGGGTTTAGGGTTTAGGGTTTAGGGTTTAGGGTTAGGGTTAGGGGTTTAGGGTTTAGGGTTTAGGGTTTAGGGTTTAGGGTTTAGGGTTTAGGGTTTAGGGTTTAGGGTTAGGGTTTAGGGTTTTAGGGTTTAGGGTTTAGGGTTTAGGGTTAGGGTTTAGGGTTTAGGGTTTAGGGTTAGGGTTAGGGTTAGGGTTAGGGTTTAGGGTTTAGGTTTAGGGTTTAGGGTTTAGGGTTTAGGGTTTAGGGTTTAGGGTTTAGGGTTTAGGGTTTAGGGTTTAGGGTTTTAGGGGGTTTAGGGTTTAGGGTTTAGGGTTAGGGTTTAGGGTTTAGGGTTAGGGTTTAGGGTTTTGGGTTTAGGGTTTAGGGTTTAGGGTTAGGGTTTAGGGTTTAGGGTTTAGGGGTTTAGGGTTTAGGGTTTAGGGTTAGGGTTAGGGTTTAGGGTTTAGGGTTTAGGGTTTAGGGTTTTAGGGTTTAGGGTTTAGGGTTTAGGGTTTAGGGTTTAGGGTTTAGGGTTTAGGGTTTAGGGTTTAGGGTTTAGGGTTTAGGGTTTAGGGTTTAGGGTTTAGGGTTTAGGGTTTAGGGTTTAGGGTTTAGGGTTTAGGGTTTAGGGTTAGGGTTTAGGGTTTAGGGTTTAGGGTTTAGGGTTTAGGGTTTAGGGTTTAGGGTTTAGGGTTTAGGGTTTAGGGTTTAGGTAGGGTTTAGGGTTTAGGGTTTAGGGTTTAGGGTTTAGGGTTTAGGGGTTTAGGGTTTAGGGTTTAGGGTTTAGGGTTAGGGTTTAGGGTTTAGGGTTTAGGGTTAGGGTTTAGGGTTTAGGGTTTAGGGTTTAGGGTTTAGGGTTTAGGGTTTAGGGTTTAGGGTTTAGGGTTTAGGGTTTAGGGTTTAGGGTTAGGGTTTAGGGTTTAGGGTTTAGGGTTTAGGGTTAGGGTTTAGGGTTTAGGGTTAGGGTTTAGGGGTTTAGGGTTTAGGGTTTAGGGTGTTAGGGTTAGGGGTTTAGGGTTTAGGGTTTAGGGTGTGGGGTTAGGGGTTTAGGGTTTAGGGTTTAGGGTTTAGGGTTTAGGGTTTAGGGTTTAGGGTTAGGGGTTAGGGTTTAGGGTTTAGGGTTTAGGGTTTAGGGTTTTAGGGTTTAGGGTTTAGGGTTTAGGGTTTAGGGTTTAGGGTTTAGGGTTAGTAGGGTTTAGGGTTTAGGGTTTAGGGT

**7DS**

CCCGGG**TTTAGGG**TTTAGGGTTTAGGGTTTAGGGTTTAGGGTTTAGGGTTAGGGTTTAGGGTTTAGGGTTTAGGGTTTAGGGTTTAGGGTTTAGGGTTTAGGGTTTAGGGTTTAGGGTTTAGGGTTTAGGGTTTAGGGTTAGGGTTAGGGTTTAGGGTTTAGGGTTTAGGGTTTAGGGTTTAGGGTTTGGGGTTTAGGGTTTAGGGTTTAGGGTTTAGGGTTAGGGTTTAGGGTTTAGGGTTAGGGTTTAGGGTTTAGGGTTTAGGGTTTAGGGTTTAGGGTTTAGGGTTTAGGGTTTAGGGTTTAGGGTTTAGGGTTTAGGGTTTAGGGTTTAGGGTTTAGGGTTTAGGGTTTAGGGTTTAGGGTTTAGGGTTTAGGGTTTAGGGTTTAGGGTTTAGGGTTTAGGGTTTAGGGTTTAGGGTTTAGGGTTTAGGGTTTAGGGTTAGGGTTTAGGGTTTAGGGTTTAGGGTTTTAGGGTTTAGGGTTAGGGTTAGGGTTAGGGTTAGGGTTTAGGGTTTAGGGTTTTAGGGTTTAGGGTTAGGGTTAGGGTTAGGGTTAGGGTTTAGGGTTTAGGGTTTAGGGTTAGGGTTTAGGGTTTAGGGTTTAGGGTTTAGGGTTTTAGGGTTTAGGGTTTAGGGTTTTAGGGTTTAGGGTTTTAGGGTTTTAGGGGGGGGTTTAGGGTTTAGGGTTAGGGTTTAGGGTTTAGGGTTAGGGTTTAGGGTTTTAGGGTTTTAGGGTTTTTTAGGGTTTAGGGTTTAGGGTTTAGGGTTTAGGGTTAGGGTTTAGGGTTAGGGTTTAGGGTTTAGGGTTTAGGGTTTAGGGTTAGGGTTTAGGGTTTAGGGTTTTTTAGGGTTTAGGGTTTAGGGTTAGGGTTTAGGGTTTAGGGTTAGGGTTTAGGGTTTAGGGTTAGGGTTTAGGGTTTAGGGTTTAGGGTTAGGGTTTAGGGTTTAGGGTTAGGGTTTAGGGTTTAGGGTTTAGGGTTTAGGGTTTAGGGTTTTTAGGGTTTAGGGTTTAGGGTTTAGGGTTTAGGGTTTAGGGTTTAGGGTTAGGGTTAGGGTTTAGGGTTAGGGTTAGGGTTTAGGGTTAGGGTTTAGGGTTTAGGGTTTAGGGTTTAGGGTTTAGGGTTTAGGGTTAGGGTTAGGGTGTTTAGGGTTTAGGGTTTAGGGTTTAGGGTTTGGGGTTAGGGTTAGGGTTAGGGTTTAGGGTTTAGGGTTAGGGTTTAGGGTTTAGGGTTAGGGTTAGGGTTTAGGGTTTAGGGTTTAGGGTTTAGGGTTAGGGTTTTAGGGTTTAGGGTTTAGGGTTAGGGTTTAGGGTTTAGGGTTTAGGGTTAGGGTTTAGGGTTTAGGGTTAGGGTTAGGGTTTAGGGTTGGTTTAGGGTTAGGGTTTAGGGTTTAGGGTTTAGGGGTTTAGGGTTTAGGGTTAGGGGTTAGGGTTTAGGGTTTAGGGTTTAGGGTTTAGGGTTAGGGTTTAGGGTTTAGGGTTTAGGGTTTAGGGTTTAGGGTTTAGGGTTAGGGTTTAGGGTTTAGGGTTTAGGGTTAGGGTTTAGGGTTTGGGTTAGGGTTTAGGGTTTAGGGTTTAGGGTTTAGGGTTTAGGGTTTAGGGTTAGGGTTTAGGGTTTAGGGTTTAGGGTTTAGGGTTTAGGGTTAGGGTTTAGGGTTTAGGGTTTAGGGTTTAGGGTTTAGGGTTTAGGGTTAGGGTTAGGGTTTAGGGTTTAGGGTTTAGGGTTTAGGGTTTAGGGTTAGGGTTTAGGGTTTAGGGTTAGGGTTAGGGTTTAGGGTTTAGGGTTTAGGGTTTAGGGTTAGGGTTAGGGTTTAGGGTTAGGGTTTAGGGTTTAGGGTTTAGGGTTTAGGGTTGTTAGGGTTTAGGGTTTAGGGTTAGGGTTAGGGTTTAGGGTTTAGGGTTTAGGGTTTAGGGTTTAGGGTTTAGGGTTTAGGGTTTAGGGTTTTAGGGTTTAGGGTTTAGGGTTTAGGGTTTAGGGTTTTAGGGTTTAGGGTTTAGGGGTTTAGGGTTAGGGTTTAGGGTTTAGGGTTTAGGGTTTTAGGGTTTAGGGGTTTAGGGTTAGGGTTAGGGTTTAGGGTTTAGGGTTTAGGGTTTAGGGTTTAGGGTTAGGGTTTAGGGTTTAGGGTTTAGGGTTTAGGGTTTAGGGTTTAGGGTTTAGGGTTAGGGTTGGTAGGGTTTAGGGTTTGGTTAGGGTTTAGGGTTTAGGGTTTAGGGTTAGGGTTTAGGGTTTAGGGGTTTAGGGTTTAGGGTTTAGGGTTTAGGGTTGGTTTAGGGGTTTAGGGTTTAGGGTTTAGGGTTTAGGGTTAGGGTTTAGGGGTTTAGGGTTTAGGGTTTAGGGTTTAGGGTTTAGGGTTTAGGGTTTAGGGTTTAGGGTTTAGGGTTTAGGGTTTAGGGGTTTAGGGTTTAGGGTTTAGGGTTTAGGGTTTAGGGTTTAGGGTTTAGGGTTTAGGGTTAGGGTTTAGGGTTTAGGGTTTAGGGTTTAGGGTTTAGGGTTTAGGGTTTAGGGTTTAGGGTTTAGGGTTTAGGGTTTAGGGTTTAGGGTTTAGGGTTTAGGGTTAGGGTTAGGGTTTAGGGTTTAGGGTTTAGGGTTTAGGGTTAGGGTTTAGGGTTTAGGGGTTTAGGGTTTAGGGGTTTAGGGTTTAGGGTTTAGGGTTTTAGGGTTTAGGGTTAGGGTTTAGGGTTTGGTAGGGTTTAGGGTTTAGGGTTTAGGGTTTAGGGTTAGGGTTAGGGTTTAGGGTTTAGGGTTTAGGGTTTAGGGTTTAGGGTTTAGGGTTTAGGGTTTAGGGTTTAGGGTTTAGGGTTTAGGGTTTAGGGTTTAGGGTTTAGGGTTTAGGGTTTAGGGTTTAGGGTTTAGGGTTTAGGGTTTAGGGTTTAGGGTTAGGGTTTAGGGTTTAGGGTTTAGGGTTTAGGGTTTAGGGTTTAGGGTTTAGGGTTAGGGTTTAGGGTTTAGGGTTTAGGGTTTAGGGTTTAGGGTTTAGGGTTTAGGGGTTTAGGGTTTAGGGTTTAGGGTTTAGGGTTTAGGGTTTAGGGTTTAGGGTTTAGGGTTTAGGGTTTAGGGTTTAGGGTTAGGGTTTAGGGTTTAGGGTTTAGGGTTTAGGGTTTAGGGTTTAGGGTTTAGGGTTTAGGGTTTAGGGTTTAGGGTTAGGGTTTAGGGTTTAGGGTTTAGGGTTTAGGGTTTAGGGTTTAGGGTTAGGGTTTAGGGTTTAGGGTTAGGGTTTAGGGTTTAGGGTTTAGGGTTTAGGGTTTAGGGTTTAGGGTTTAGGGTTTAGGGTTTAGGGTTTAGGGTTAGGGTTTAGGGTTTAGGGTTTTAGGGTTTAGGGTTTAGGGTTTAGGGTTTAGGGTTTAGGGTTAGGGTTTAGGGTTTAGGGTTTAGGGTTTAGGGTTTAGGGTTTAGGGTTTAGGGTTTAGGGTTAGGGTTTAGGGTTTAGGGTTTAGGGTTTAGGGTTTAGGGTTAGGGTTTAGGGTTTAGGGTTTAGGGTTTAGGGTTTAGGGTTTAGGGTTTAGGGTTTAGGTTTAGGGTTTAGGGTTTAGGGTTTAGGGTTTAGGGTTTAGGGTTTAGGGTTTAGGGTTTTAGGGTTTAGGGTTTAGGGTTTAGGGTTTAGGGTTTAGGGTTAGGGTTTAGGGTTTAGGGTTTAGGGTTTAGGGTTTAGGGTTTAGGGTTAGGGTTTAGGGTTTAGGGTTTAGGGTTTAGGGTTTAGGGTTTAGGGTTTAGGGTTAGGGTTTAGGGTTTAGGGTTTAGGGTTTAGGGTTTAGGGTTTAGGGTTTAGGGTTTAGGGTTTAGGGTTTAGGGTTTAGGGTTTAGGGTTTAGGGTTTAGGGTTTAGGGTTTAGGGTTTAGGGTTTAGGGTTTAGGGTTTAGGGTTTAGGGTTTAGGGTTAGGGTTTAGGGTTTAGGGTTTAGGGGTTTAGGGTTTAGGGTTTAGGGTTTAGGGTTTAGGGTTTAGGGTTTAGGGGTTAGGGTTTAGGGTTTAGGGTTTAGGGTTTAGGGTTTAGGGTTTAGGGTTTAGGGTTTAGGGTTTAGGGTTTAGGGTTTAGGGTTTAGGGTTTAGGGTTTAGGGTTTAGGGTTTAGGGTTTAGGGTTAGGGTTTAGGGTTTAGGGTTTAGGGTTTAGGGTTTAGGGTTTAGGGTTTAGGGTTTAGGGTTTTAGGGTTTAGGGTTTAGGGTTTAGGGTTTAGGGTTAGGGTTTAGGGTTTAGGGTTTAGGGTTTAGGGTTTAGGGTTTAGGGTTTAGGGTTTAGGGTTTAGGGTTAGGGTTTAGGGTTTAGGGTTTAGGGTTAGGGTTTAGGGTTTAGGGTTTAGGGTTTAGGGTTTAGGGTTTAGGGTTTAGGGTTTAGGGTTTAGGGTTAGGGTTTAGGGTTTAGGGTTTAGGGTTTAGGGTTTAGGGTTTAGGGTTTAGGGTTTAGGGTTTAGGGTTTAGGGTTAGGGTTTAGGGTTTAGGGTTTAGGGTTTAGGGTTTAGGGTTTAGGGTTTAGGGTTTAGGGTTTAGGGTTTAGGGTTTAGGGTTTAGGGTTTAGGGTTTAGGGTTTAGGGTTTAGGGTTTAGGGTTAGGGTTAGGGTTTAGGGTTTAGGGTAGGGTTTAGGGTTTAGGGTTTAGGGTTTAGGGTTTAGGGTTTAGGGTTTAGGGTTTAGGGTTTAGGGTTTAGGGTTTAGGTTAGGGTTTAGGGTTTAGGGTTTAGGGTTTAGGGTTTAGGGTTTAGGGTTTAGGGTTTAGGGTTTAGGGTTTAGGGTTTAGGGGTTTAGGGTTTAGGGTTAGGGTTTAGGGTTTAGGGTTTAGGGGTTTAGGGTTTAGGGTTTAGGGTTTAGGGTTTAGGGTTTAGGGTTTTAGGGTTTAGGGTTTAGGGTTTAGGGGTTTAGGGTTTAGGGTTTAGGGTTTAGGGTTTAGGGTTAGGGTTTAGGGTTTAGGGTTTAGGGTTTAGGGTTAGGGTTTAGGGTTTAGGGTTTAGGGTTTAGGTTTAGGGTTTAGGGTTTAGGGTTTAGGGTTTAGGGTTAGGGTTTAGGGTTTAGGGTTTAGGGTTTTAGGGTTTAGGTTAGGGTTTAGGGTTTAGGGTTTAGGGTTTAGGGTTTAGGGTTAGGGTTTAGGGTTTAGGGTTTAGGGTTTAGGGTTTAGGGTTTAGGGTTTAGGGTTTAGGGTTTAGGGTTTAGGGGTTTAGGGTTTAGGGTTTAGGGTTAGGGTTTAGGGTTAGGGTTTAGGGTTTAGGGTTAGGGTTTAGGGTTTAGGGTTTAGGGTTTAGGGTTAGGGTTTAGGGTTTAGGGTTTAGGGTTAGGGTTTAGGGTTTAGGGTTTAGGGTTTAGGGTTTAGGGTTTAGGGTTTAGGGTTAGGGTTTTAGGGTTTAGGGTTTAGGGTTTAGGGTTTAGGGTTTAGGGTTTAGGGTTTAGGGTTTAGGGTTAGGGTTTAGGGTTTAGGGTTAGGGTTTAGGGTTTAGGGTTTAGGGTTAGGGTTAGGGTTTAGGGTTTAGGGTTTAGGGTTTAGGGTTTAGGGTTTAGGGTTTAGGGTTTAGGGTTTAGGGTTTAGGGTTTAGGGTTTAGGGTTTAGGGTTAGGTTTAGGGTTTAGGGTTTAGGGTTTAGGGTTTAGGGTTTAGGGTTTAGGGTTTAGGGTTTAGGGTTAGGGTTTAGGGTTAGGGTTTAGGGTTTAGGGTTTAGGGTTTAGGGTTAGGGTTTAGGGTTAGGGTTTAGGGTTTAGGGTTTAGGGTTTAGGGTTTAGGGTTTAGGGTTTAGGGTTAGGGTTTAGGGTTTAGGGTTTAGGGTTTAGGGTTTAGGGTTTAGGGTTTAGGGTTAGGGTTTAGGGTTTAGGGTTTAGGGTTAGGGTTTAGGGTTTAGGGTTTAGGGTTTAGGGTTAGGGTTTAGGGTTTAGGGTTTAGGGTTTAGGGTTTAGGGTTTAGGGTTTAGGGTTTAGGGTTTAGGGTTTAGGGTTTAGGGTTTAGGGTTTAGGGTTAGGGTTTAGGGTTTAGGGTTTAGGGTTTAGGGTTTTAGGGGTTTAGGGTTTAGGGTTTAGGGTTTAGGGTTTAGGGTTTAGGGTTTAGGGTTTAGGGTTTAGGGTTAGGGTTTAGGGTTTAGGGTTTAGGGTTTAGGGTTTAGGGTTTAGGGTTTAGGGTTTAGGGTTTAGGGTTTAGGGTTTAGGGTTTAGGGTTTAGGGTTTAGGGTTTAGGGTTTAGGGTTAGGGTTTAGGGTTTAGGGTTTAGGGTTTAGGGGTTTAGGGTTTAGGGTTTAGGGTTTAGGGTTAGGGTTTAGGGTTTAGGGTTTAGGGTTAGGGTTAGGGTTTAGGGTTTAGGGTTAGGGTTTAGGGTTTAGGGTTTAGGGTTAGGGTTTAGGGTTTAGGGTTTAGGGTTTAGGGTTTAGGGTTTAGGGTTTAGGGTTTAGGGTTTAGGGTTAGGGTTTAGGGTTTAGGGTTTAGGGTTTAGGGTTTAGGGTTTAGGGTTTAGGGTTTAGGGTTTAGGGTTAGGGTTTAGGGTTTAGGGTTTAGGGTTTAGGTTTAGGGTTTAGGGTTTAGGGTTAGGGTTTAGGGTTTAGGGTTTAGGGTTTAGGGTTAGGTTAGGGTTTAGGGTTTAGGGTTAGGGTTTAGGGTTTAGGGTTTAGGGTTTAGGGTTTAGGGTTTAGGGTTAGGGTTTAGGGTTTAGGGTTTAGGGTTAGGGTTTAGGGTTTAGGGTTTAGGGTTAGGGTTTAGGGTTTAGGGTTTTTAGGGTTTTAGGGTTTAGGGTTTAGGGTTTAGGGTTAGGGTTTAGGGTTTAGGGTTAGGGTTTAGGGTTTAGGGTTTAGGGTTTTAGGGTTTTAGGGTTTAGGGTTTAGGGTTTAGGGTTAGGGTTTAGGGTTAGGGTTTAGGGTTTAGGGTTTAGGGTTAGGGTTTAGGGTTTAGGGTTTAGGGTTTAGGGTTTAGGGTTTAGGGTTTAGGGTTTAGGGTTTAGGGTTTAGGGTTTAGGGTTTAGGGTTTAGGGTTTAGGGTTAGGGTTTAGGGTTTAGGGTTTAGGGTTTAGGGTTTAGGGTTTAGGGTTTAGGGTTTAGGGTTTAGGGTTTAGGGTTTAGGGTTTAGGGTTTAGGGTTTAGGGTTTAGGGTTTAGGGTTTAGGGTTTAGGGTTTAGGGTTTAGGGTTTAGGGTTAGGGTTTAGGGTTTAGGGTTTAGGGTTTTAGGGTTTAGGGTTTAGGGTTAGGGTTTAGGGTTTAGGGTTTAGGGTTTAGGGTTTAGGGTTTAGGGTTTAGGGTTTAGGGTTAAGGGTTTAGGGTTTAGGGTTTAGGGTTAGGGTTTAGGGTTTAGGGTTTAGGGTTTAGGGTTTAGGGTTTAGGGTTTAGGGTTTAGGGTTTAGGGTTTAGGGTTTAGGGTTTAGGGTTTAGGGTTTAGGGTTTAGGGTTAGGGTTTAGGGTTTAGGGTTTAGGGTTAGGTTTAGGGTTTAGGGTTTAGGGTTTAGGGTTTAGGGTTTAGGGTTTAGGGTTAGGGTTTAGGGTTTAGGGTTTAGGGTTAGGGTTTAGGGTTAGGGTTTAGGGTTTAGGGTTTAGGGTTTAGGGTTTAGGTTAGGGTTTAGGGTTTAGGGTTAGGGTTTAGGGTTTAGGGTTTAGGGTTTAGGGTTTAGGGTTTAGGGTTAGGGTTAGGGTTTAGGTTGGTTTAGGGTTTAGGGTTTAGGGTTTAGGGTTTAGGGTTTAGGGTTTAGGGTTTAGGGTTAGGGTTAGGGTTAGGGTTTAGGGTTTAGGGTTTAGGGTTAGGGTTTAGGGTTTAGGGTTTAGGGTTTTAGGGTTTAGGGTTTAGGGTTAGGGTTAGGGTTTAGGGTTTAGGGTTAGGGTTTAGGGTTTAGGGTTAGGGTTTAGGGTTTAGGGTTTAGGGTTTAGGGTTTAGGGGTTAGGGTTTAGGGTTTAGGGTTTAGGGTTTAGGGTTTAGGGTTAGGGTTTAGGGTTTAGGGTTTAGGGTTTAGGGTTTAGGGTTTAGGGTTTAGGGTTTAGGGTTTAGGGTTTAGGGTTAGGGTTTAGGGTTAGGGTTTAGGGTTTAGGGGTTAGGGTTTAGGGTTTAGGGTTTAGGGTTTAGGGTTTAGGGTTTAGGGTTAGGTTAGGGTTGGGTTTAGGGTTTAGGTTTAGGGTTTAGGGTTTAGGGTTTAGGGTTTAGGGTTTTAGGGTTAGGGTTTTAGGTGGGTTTAGGGTTAGGGTTTAGGGTTTAGGGTTTAGGGTTTAGGGTTTAGGGTTAGGGTTTAGGGTTTAGGGTTAGGTTAGGGTTTAGGGTTTAGGGTTTAGGGTTTAGGGTTAGGGTTAGGGTTTAGGGTTAGGGTTTAGGGTTTAGGGTTTAGGGTTAGGGTTTGGTTTAGGGTTTAGGGTTAGGGTTTAGGGTTTAGGGTTAGGGTTTAGGGTTTAGGGTTTAGGGTTTAGGGTTTGGGTTTAGGGTTAGGGTTTAGGGTTTAGGGTTTAGGGTTTAGGGTTTAGGGTTTAGGGTTTTAGGGTTTAGGGTTTAGGGTTTGGGGTTTAGGGTTAGGGTTTAGGGTTAGGGTTTAGGGGGTTTAGGGTTTAGGGTTTAGGGTTTAGGGTTTAGGGTTTAGGGTTTAGGGTTTAGGGTTTAGGGTTTAGGGTTTAGGGTTTAGGTTTAGGGTTTAGGGTTTAGGGTTTAGGTTTAGGGTTTAGGGTTTAGGGTTTAGGGTTTAGGGTTTTAGGGTTTAGGGTTTAGGGTTGGGTTTAGGGTTTAGGGTTTAGGGTTAGGGTTTAGGGTTTAGGGTTTAGGGTTTAGGTTTAGGGTTTAGGGTTTAGGGTTTAGGGTTTAGGGTTTAGGGTTTAGGGTTTAGGGTTTAGGGGTTTAGGGTTTAGGGTTAGGGTTTAGGGTTAGGGTTTAGGGTTTAGGGGTTTAGGGTTTAGGGTTTAGGGTTTAGGGTTTAGGGTTTAGGGTTTAGGGTTTAGGGTTTAGGGTTTAGGGTTTAGGGTTTAGGGTTTAGGGTTTAGGGTTTAGGGTTTAGGGTTTAGGGTTTAGGGTTTAGGGTTTAGGGTTTAGGGGTTTAGGGTTAGGGTTTAGGGTTTAGGGTTTAGGGTTTAGGGTTTAGGGTTTGGGTTTAGGGTTAGGGTTTAGGGTTTAGGGTTTAGGGTTTAGGGTTTAGGGTTTAGGGTTTAGGGTTTAGGGTTTTAGGGTTTAGGGTTTAGGGTTTAGGGTTTAGGGTTTAGGGTTTAGGGTTTAGGGTTTAGGGTTAGGGTTTAGGGTTTAGGGTTTAGGGTTTAGGGTTTAGGGTTTAGGGTTTAGGGTTTAGGGTTTAGGGTTTAGGGTTTAGGGTTTAGGGTTTAGGGTTGGGTTTAGGGTTTAGGGTTTAGGGTTAGGGTTTGGGTTTAGGGTTAGGGTTTAGGGTTTAGGGGTTTAGGGTTTAGGTTAGGGTTTAGGGTTTAGGGTTTAGGGTTTAGGGTTTAGGGTTAGGGTAGGTTAGGGTTTAGGGTTTTAGGGTTTAGGGTTTTAGGGTTTTAGGGTTTAGGGTTTAGGGTTTTAGGGTTTAGGGTTTAGGGTTTAGGGTTTAGGGTTTAGGGTTTAGGGTTAGGGTTAGGGTTTAGGGTTTTAGGGTTTAGGGTTTAGGGTTAGGGTTTAGGGTTTAGGGTTTAGGGTTAGGGTTTAGGGTTTAGGGTTTGGTTAGGGTTTAGGGTTTAGGGTTTAGGGTTTAGGTTTAGGGTTTAGGGTAGGGTTTAGGGTTTAGGGTTAGGGTTTAGGGTTTAGGGTTTAGGGTTTAGGGTTTAGGGTTTAGGGTTTAGGGTTTAGGGTTAGGGTTTAGGGTTTAGGGTTTAGGGTTTAGGGTTTAGGGTTTAGGGTTGGTTTTAGGGTTTAGGGTTTAGGGTTTAGGGTTTTGGGGTTTAGGGTTTAGGGTTTAGGGTTTAGGGTTAGGGTTTAGGGTTTAGGGTTTAGGGTTTAGGGTTTAGGTGGTTTAGGGTTTAGGGTTTAGGGTTTAGGGTTTAGGGTTTAGGGTTTAGGGTTAGGGTTTAGGGTTTAGGGTTAGGGTTAGGGTTTAGGGTTTAGGGTTTAGGGTTTAGGGTTTAGGGGTTTAGGTGGTTTAGGGTTTAGGTTTAGGTTTAGGGTTTAGGGTTTAGGGTTTAGGGTTTAGGGTTAGGGTTTAGGGTTTAGGGTTTAGGGTTTAGGGTTTAGGGTTTAGGTTAGGGTTAGGGTTTAGGGTTTAGGGTTTAGGGTTTAGGGTTTAGGGTTAGGGTTTAGGGTTTAGGGTTTAGGGTTTAGGGTTTAGGGTTTAGGGTTTAGGGTTAGGGTTTAGGGTTTAGGGTTTAGGGTTTAGGGTTTAGGGTTTAGGGTTTAGGGTTTAGGGTTTAGGGTTTAGGGTTAGGGTTTAGGGTTTAGGGTTTAGGGTTTAGGGTTTAGGGTTAGGGTTTAGGGTTTAGGGTTTAGGGTTAGGGTTAGGGTTTAGGGTTTAGGGTTTAGGGTTAGGGTTTAGGGTTTAGGGTTTAGGGTTAGGTTAGGGTTTAGGGTTTAGGGTTTAGGGTTTAGGGTTTAGGGTTTAGGGTTTAGGGTTAGGGTTTAGGGGTTTAGGGTTTAGGGTTTAGGGTTTAGGGTTTAGGGTTTAGGGTTTAGGGTTTAGGGTTTAGGGTTTAGGGTTGTTAGGGTTTAGGGTTTAGGGTTTAGGGTTTTAGGGTTTAGGGTTTAGGGTTTAGGGTTTAGGGTTTAGGGTTTAGGGTTTAGGGTTTAGGGTTTAGGGTTAGGGTTTAGGGTTTAGGGTTTAGGGTTAGGGTTAGGGTTTAGGGTTAGGGTTTAGGGTTTAGGGTTTAGGGTTTAGGGTTTAGGGTTTAGGGTTTAGGGTTTAGGGTTTAGGGTTAGGGTTAGGGTTTAGGGGTTTAGGGTTTAGGGTTTAGGGTTTAGGGTTTAGGGTTTTAGGGTTTTAGGGTTTAGGGTTTAGGGTTTAGGGTTTAGGGTTTAGGGTTTAGGGTTTAGGGTTTAGGGTTTAGGGTTTTAGGGTTTAGGGTTTAGGGTTAGGGTTTAGGGTTTAGGGTTTAGGGTTAGGGTTTAGGGTTTAGGGTTTAGGGTTTAGGGTTTAGGGTTTAGGGTTTAGGGTTTAGGGTTTAGGGTTTAGGGTTTAGGGGTTTAGGGTTTAGGGTTAGGGTTTAGGGTTTAGGGTTTAGGGTTTAGGGTTAGGGTTTAGGGTTTAGGGTTTAGGGTTTAGGGTTTAGGGTTTAGGGTTTAGGGTTTAGGGTTTAGGGTTAGGGTTTAGGGTTTAGGGTTTAGGGTTTAGGGTTTAGGGGTTTAGGGGTTTAGGGTTTAGGGTTAGGGTTTAGGGTTTAGGGTTTAGGGTTAGGGTTAGGGTTTAGGGTTTAGGGTTTAGGGTTTAGGGTTTAGGGTTTAGGGTTAGGGTTTAGGGTTTTAGGGTTTAGGGTTTAGGGTTTAGGGTTTAGGGTTTAGGTTTAGGGTTTAGGGTTTAGGGTTTAGGGTTTAGGGTTTAGGTTTAGGGTTAGGGTTTAGGGTTTAGGGTTTAGGGTTTAGGGTTTAGGGTTTAGGGTTTAGGGTTTAGGGGTTTAGGGTTTTAGGGTTTAGGGTTTAGGGTTAGGGTTTAGGTTTAGGGTTTAGGGTTTAGGGTTTAGGCTGTTTAGGGTTAGGGTTTTAGGGTTTGTTTTAGGGTTTTAGGGTTTTAGGGTTTT

**7DL**

GTTTAGG**TTTAGGG**TTTAGGGTTTAGGGTTTAGGGTTTAGGGTTTAGGGTTAGGGTTTAGGGTTTAGGGTTTAGGGTTAGGGTTTAGGGTTTAGGGTTTAGGGTTAGGTTTAGGGTTAGGGTTTAGGGTTTAGGGTTTAGGGTTTAGGGTTTAGGGTTTAGGGTTTAGGGTTTAGGGTTTAGGGTTTAGGGTTTAGGGTTTAGGGTTTAGGGTTTAGGGTTTAGGGTTTAGGGTTTAGGGTTTAGGGTTTAGGGTTTAGGGTTTTAGGGTTTAGGGTTTAGGGTTTAGGGTTTGGGTTTAGGGTTTAGGGTTTAGGGTTTAGGGTTTAGGGTTTAGGGTTTAGGGTTTAGGGTTTAGGGTTAGGGTTAGGGTTAGGGTTTAGGGTTTAGGGTTTAGGGTTTAGGGTTTAGGGTTAGGGTTTAGGGTTTAGGGTTTAGGGTTAGGGTTTAGGGTTAGGGTTTAGGGTTTGGGTTAGGGTTTAGGGTTTAGGGTTTAGGGTTTAGGGTTTAGGGTTTGGGGTTTAGGGGGTTTAGGGTTTAGGGTTTAGGGTTTAGGGTTTAGGGTTTAGGGTTTAGGGTTTTAGGGTTTAGGGTTTAGGGTTAGGGTTAGGGTTTTAGGGTTTAGGGTTTAGGGTTTAGGGTTAGGGTTTAGGGTTTAGGGTTTAGGGTTAGGGTTAGGGTTAGGGTTTTAGGGTTTAGGGTTTAGGGTTTAGGGTTTAGGGTTTAGGGTTAGAGTTAGGGTTTAGGGTTTAGGGTTAGGGTTTAGGGTTTAGGGTTAGGGTTTAGGGTTTAGGGTTTAGGGTTTAGGGTTAGGGTTTAGGGTTAGGGTTTAGGGTTTAGGGTTAGGGTTAGGGTTTAGGGTTTAGGGTTTAGGGTTAGGGTTTAGGGTTTAGGGTTTAGGGTTGGGTTGTGGGTTTGGGGTTTAGGGTTTAGGGTTAGGGTTTAGGGTTAGGGTTAGGGTTTAGGGTTTAGGGTTTAGGGTTAGGGTTTAGGGTTTAGGGTTTAGGGTTTAGGGTTTAGGGTTTAGGGTTTAGGGTTAGGGTTTAGGGTTTAGGGTTTAGGGTTTAGGGTTTAGGGTTTAGGGTTAGGGTTAGGGTTAGGGTTTAGGGTTTAGGGTTTAGGGTTTAGGGTTTAGGGTTTAGGGTTTAGGGTTTAGGGTTTAGGGTTTAGGGTTAGGGTTAGGGTTTAGGGTTTAGGGTTTAGGGTTAGGGTTTAGGGTTTAGGGTTTAGGGTTTAGGGTTAGGGTTTAGGGTTTAGGGTTTAGGGTTTAGGGTTTAGGGTTAGGGTTTAGGGTTAGGGTTTAGGGTTTAGGGTTAGGGTTAGGGTTTAGGGTTTAGGGTTTAGGGTTAGGGTTTAGGGTTTAGGGTTTAGGGTTAGGGTTTAGGGTTTAGGGTTTAGGGTTTAGGGTTTAGGGTTTAGGGTTTAGGGTTTAGGGTTTAGGGTTAGGGTTTAGGGTTTAGGGTTTAGGGTTTAGGGTTTAGGGTTTAGGGTTTAGGGTTTAGGGTTTAGGGTTTAGGGTTTAGGGTTTAGGGTTTAGGGTTTAGGGTTTAGGGTTTAGGGTTTAGGGTTTAGGGTTAGGGTTTAGGGTTTAGGGTTTAGGGTTTAGGGTTTAGGGTTTAGGGTTTAGGGTTTAGGGTTAGGGTTTAGGGTTTAGGGTTTAGGGTTTAGGGTTTAGGGTTTAGGGTTTAGGGTTTAGGGTTTAGGGTTTAGGGTTTAGGGTTTAGGGTTTAGGGTTTAGGGTTTAGGGTTTAGGGTTTAGGGTTTAGGGTTTAGGGTTTAGGGTTTAGGGTTTAGGGTTTAGGGTTTAGGGTTTAGGGTTTAGGGTTTAGGGTTTAGGGTTTAGGGTTAGGGTTAGGGTTTAGGGTGTTAGGGTTTAGGGTTTAGGGTTTAGGGTTTAGGGTTTAGGGTTTAGGGTTTAGGGTTTAGGGTTTAGGGTTTAGGGTTTAGGGTTTAGGGTTTAGGGTTTAGGGTTTAGGGTTTAGGGTTTAGGGTTTAGGGTTTAGGGTTTAGGGTTTAGGGTTTAGGGTTTAGGGTTTAGGGTTTAGGGTTTAGGGTTTAGGGTTTAGGGTTTAGGGTTTAGGGTTTAGGGTTTAGGGTTTAGGGTTTAGGGTTTAGGGTTGGTTTAGGGTTTAGGGTTTAGGGTTTAGGGTTTAGGGTTTAGGGTTTAGGGTTTAGGGTTAGGGTTTAGGGTTTAGGGTTTAGGGTTTAGGGTTTAGGGTTTAGGGTTTAGGGTTTAGGGTTTAGGGTTTAGGGTTTAGGGTTTAGGGTTTAGGGTTTAGGGTTTAGGGTTTAGGGTTTAGGGTTTAGGGTTTAGGGTTTAGGGTTTAGGGTTTAGGGTTTAGGGTTTAGGGTTTAGGGTTTAGGGTTTAGGGTTTAGGGTTTAGGGTTTAGGGTTTAGGGTTTAGGGTTTAGGGTTTAGGGTTTAGGGTTTAGGGTTTAGGGTTAGGTTTAGGGTTTAGGGTTTAGGGTTTAGGGTTAGGGTTTAGGGTTTAGGGTTTAGGGTTTAGGGTTTAGGGTTTAGGGTTTAGGGTTAGGGTTTAGGGTTTAGGGTTTAGGGTTAGGGTTTAGGGTTTAGGGTTTAGGGTTTAGGGTTTAGGGTTTAGGGTTAGGGTTTAGGGTTTAGGGTTTAGGGTTTAGGGTTTAGGGTTTAGGGTTTAGGGTTTAGGGTTAGGGTTTAGGGTTTAGGGTTTAGGGTTTAGGGTTTAGGGTTTAGGGTTTAGGGTTAGGGTTTAGGGTTTAGGGTTTAGGGTTTAGGGTTAGGGTTTAGGGTTTAGGGTTTAGGGTTTAGGGTTTAGGGTTTAGGGTTTAGGGTTTAGGGTTTAGGGTTTAGGGTTTAGGGTTTAGGGTTTAGGGTTTAGGGTTTAGGGTTTAGGGTTTAGGGTTTAGGTTTAGGGTTAGGGTTTAGGGTTTAGGGTTTAGGGTTTAGGGTTTAGGGTTTTAGGGTTAGGGTTTAGGGTTAGGGTTTAGGGTTTTGGGTTTAGGGTTAGGGTTAGGTTAGGGTTTAGGGTTAGGGTTTAGGGTTAGGGTTAGGGTTAGGGTTTAGGGTTTAGGGTTTAGGGTTTTAGGGTTTAGGGTTTAGGGTTAGGGTTTAGGGGTTAGGGTTTAGGGTTTAGGGTTTAGGGTTTAGGGTTTAGGGTTTAGGGTTTAGGGTTGGTTAGGGTTTAGGGTTAGGGTTTAGGGTTTAGGGTTTAGGGTTTAGGGTTTAGGGTTTAGGGTTTAGGGTTTAGGGTTTAGGGTTTAGGGTTAGGGTTTAGGGTTTAGGGTTTAGGGTAGGGTTTAGGGTTTAGGGTTTAGGGTTTAGGGTTTAGGGTTTAGGGTTTAGGGTTTAGGGTTTAGGGTTTAGGGTTTAGGGTTTAGGGTTTAGGGTTTAGGGTTTAGGGTTTAGGGTTAGGGTTTAGGGTTAGGGTTTAGGGTTTAGGGTTTAGGGTTTAGGGTTAGGGTTTAGGGTTAGGGTTTAGGGTTTAGGGTTTAGGTTTAGGGTTTAGGGTTTAGGGTTTAGGGTTTAGGGTTTAGGGTTTAGGGTTAGGGTTTAGGGTTTAGGGTTTAGGGTTTAGGGTTTAGGGTTTAGGGTTTAGGGTTTAGGGTTTAGGGTTAGGGTTTAGGGTTTAGGGTTTAGGGTTTAGGGTTTAGGGGTTTAGGGTTAGGGTTTAGGGTTTAGGGTTTAGGGTTTAGGGTGGTTAGGGTTTAGGGTTTAGGGTTTAGGGTTTAGGGTTAGGGTTTAGGGTTTAGGGTTTAGGGTTTAGGTTTAGGGGTTTAGGGTTAGGGTTTAGGGTTTAGGGTTTAGGGTTTAGGGTTTAGGGTTTAGGGTTTAGGGTTTAGGGTTTAGGGTTTAGGGTTTAGGGTTTAGGGTTTAGGGTTTAGGGTTTAGGGTTTAGGGTTTAGGGTTTAGGGTTTAGGGTTTAGGGTTAGGGTTTAGGGTTTAGGGTTAGGGTTTAGGGTTTAGGGTTTAGGGTTGTAGGGTTTAGGGTTAGGGTTTAGGGTTTAGGGTTTAGGTTAGGGTTTAGGGTTTAGGGTTAGGGTTTAGGGTTTAGGGTTTAGGGTTAGGGTTAGGTTAGGGTTTAGGGTTTAGGGTTTAGGGTTTAGGGTTTAGGGTTTAGGGTTTAGGGTTTAGGGTTTAGGGTTAGGGTTTAGGGTTTAGGGTTTAGGGTTTAGGGTTAGGGTTTAGGGTTTAGGGTTTAGGGTTTAGGGTTTAGGGTTTAGGGTTTAGGGTTTAGGGTTTAGGGTTTAGGGTTTAGGGTTAGGGTTTAGGGTTTAGGGTTTAGGGTTTAGGGTTTAGGGTTTAGGGTTTAGGGTTTAGGGTTTAGGGTTTAGGGTTTAGGGTTTAGGGTTAGGGTTAGGGTTTTAGGGTTTAGGGTTTAGGGTTTAGGGTTTAGGGTTTAGGGTTTAGGGTTTAGGGTTAGGGTTTAGGGTTTAGGGTTTAGGGTTTAGGGTTTAGGGTTTAGGGTTTAGGGTTTAGGGTTTAGGGTTTAGGGTTTAGGGTTAGGGTTTAGGGTTTAGGGTTTAGGGTTTAGGGGTTTAGGGTTTAGGGTTTAGGGTTTAGGGTTAGGGTTTAGGGTTTAGGGTTTAGGTTTAGGGTTTAGGGTTAGGGTTTAGGGTTAGGGTTTAGGGTTTAGGGTTTAGGGTTTAGGGTTTAGGGTTTAGGGTTAGGGTTAGGGTTAGGGTTTAGGGTTTAGGGTTTAGGGTTTAGGGTTAGGGTTTAGGGTTTAGGGTTAGGGTTAGGGTTTAGGGTTTAGGGTTTAGGGTTAGGGTTAGGTTTAGGGTTTAGGGTTTAGGGTTTAGGGTTTAGGGTTTAGGGTTTAGGGTTTAGGGTTTAGGGTTTAGGGTTTAGGGTTTAGGGTTTAGGGTTAGGGTTTAGGGTTTAGGGTTAGGGTTTAGGGTTTAGGGTTTAGGGTTGTTAGGGTTTAGGGTTTAGGGTTTAGGGTTTAGGGTTAGGGTTTAGGGTTTAGGGTTTAGGGTTTAGGGTTTAGGGTTAGGGTTTAGGGTTTAGGGTTTAGGGTTTAGGGTTTAGGGTTTAGGGTTAGGGTTTAGGGTTTAGGGTTTAGGGTTTAGGGTTTAGGGTTTAGGGTTAGGGTTTAGGGTTTAGGGTTTAGGGTTTAGGGTTTAGGGTTTAGGGTTTAGGGTTTTAGGGTTTAGGGTTTAGGGTTTAGGGTTTAGGGTTTAGGGTTTAGGTTTAGGGTTTAGGTTTAGGGTTTAGGGTTTAGGGTTTAGGGTTTAGGGTTTAGGGTTTAGGGTTTAGGTTTAGGGTTTAGGGTTTAGGGTTTAGGGTTTAGGGTTTAGGGTTTAGGTTTAGGGTTAGGGTTAGGGTTAGGGTTTAGGGTTTAGGGTTTAGGGTTTAGGGTTTAGGGTTTAAGGGTTTAGGGTTTAGGGTTTAGGGGTTTAGGGTTTAGGGTTTAGGGTTTAGGGTTTAGGGTTTAGGGTTTAGGGTTTAGGGTTTAGGGTTTTAGGGTTTAGGGTTTAGGGTTTAGGGTTTAGGGTTAGGGTTTAGGGTTTAGGGTTAGGGTTTAGGGTTTAGGGTTTAGGGTTAGGGTTTAGGGTTTAGGGTTTAGGGTTTAGGGTTTAGGGTTTAGGGTTAGGGTTTAGGGTTTAGGGTTTAGGGTTTAGGGTTTAGGGTTTAGGGTTTAGGTTAGGGTTTAGGGTTTAGGGTTTAGGGTTTAGGGTTTAGGGTTTAGGGTTTAGGGTTAGGGTTTAGGGTTTAGGGTTTAGGGTTTAGGGTTTAGGGTTAGGGTTTAGGGTTTAGGGTTTAGGGTTTAGGGTTTAGGGTTTAGGGTTTAGGGTTTAGGTTAGGGTTTAGGGTTTAGGGTTTAGGGTTTAGGGTTTAGGGTTTAGGGTTTAGGTTAGGGTTAGGGTTTAGGGTTTAGGGTTTAGGGTTTAGGGTTTAGGGTTTAGGTTTAGGGTTTAGGGTTTAGGGTTTAGGGTTTAGGGTTTAGGGTTTAGGGTTAGGGTTTAGGGTTGGGTTTAGGGTTTAGGGTTTAGGGTTTAGGGGTTAGGGTTAGGGTTTAGGGTTTAGGGTTTAGGGTTTAGGGTTTAGGGTTTAGGGTTTAGGGTTTAGGGGTTTAGGGTTTAGGGTTTAGGGTTTAGGGTTAGGTTAGGGTTTAGGGTTTAGGGTTAGGGTTTAGGGTTTTGGGTTTAGGGTTTAGGGTTTAGGGTTTAGGGTTTAGGGTTTTAGGGTTTAGGGTTTAGGGTTTAGGGTTAGGGTTTAGGGTTTAGGGTTTTAGGGTTTAGGGTTTAGGGTTTAGGGTTTAGGGTTTTAGGGTTTAGGGTTTAGGGTTAGGGTTTAGGGTTAGGTTTGGGTTTAGGTTTAGGGTTTAGGGTTTAGGGTTTAGGGTTTAGGGTTAGGGTTTAGGGTTTTAGGTGGTTTAGGGTTTAGGGTTTAGGGTTTAGGTTTTGGTTTAGGGTTTAGGGTTTAGGGTTTAGGGTTTAGGGTTTAGGGTTTAGGGTTTAGGGTTTAGGGTTTAGGGTTTAGGGTTTAGGGTTTAGGGTTTAGGGTTTAGGGTTTAGGGTTTAGGTTTAGGGTTTAGGGTTTAGGGTTTAGGGTTTAGGGTTTAGGGTTTAGGGTTTAGGGTTTAGGGGTTTTGGGTTAGGGTTTAGGGTTAGGGTTGGTAGGGTTTAGGGTTTAGGGTTTAGGGTTTAGGGTTTAGGGTTAGGGTTTAGGTTTAGGGGTTTAGGGTTTAGGGTTTAGGGTTTAGGGTTAGGGTTTAGGGTTAGGGTTTAGGGTTTAGGGTGGGTTTAGGGTTTAGGTTTAGGGTTTAGGGTTAGGGTTTAGGGTTAGGGTTTAGGGTTTAGGGTTAGGGTTTAGGGTTTAGGGTTTAGGGTTTAGGGTTTAGGGTTTAGGGTTTAGGGTTAGGGTTTAGGGTTTAGGGTTTAGGTTTAGGGTAGGTTAGGGTTGTTAGGGTTTGGTAGGGTTTAGGGTTTAGGGTTTAGGGTTTAGGGTTTAGGGTTAGGGTTTAGGGTTAGGGTTAGGGTTTAAGGGTTTAGGGTTTAGGTTTAGGGTTTAGGGTTAGGGTTTAGGGTTTAGGGTTGGGTTAGGGTTTAGGGTTTAGGTTAGGGTTTAGGGTTTAGGTTTAGGGTTTAGGGTTTAGGTTAGGGTTTAGGGTTTAGGGTTAGGGTTTAGGGTTTAGGGTAGGTTTAGGGTTTAGGGGTTAGGGTTTAGGGTTTAGGGTTTAGGGTTTAGGGTTTAGGGTTTAGGGTTTAGGGTTTAGGGTTAAGGGTTAGGGTTTAGGGTTTAGGGTTTAGGGTTAGGGTTAGGGTTAGGGTTTAGGGTTTAGGGTTTAGGGTTTTAGGGTTTAGGTTTAGGGTTTAGGGTTTTGGGTTTAGGTTTAGGGTTAGGGTTTAGGGGTTTAGGGTTAGGGTTTAGGGTTTAGGGTTTAGGGTTAGGGTTTAGGGTTAGGGTTTAGGGTTTAGGGTTAGGGTTTAGGGTTAGGGTTTAGGGTTTAGGGTTTAGGGTTTAGGGTTTAGGGTTAGGGTTTAGGGGTTTAGGGTTTAGGGTTAGGGTTTAGGGGTTAGGGGGTGGTTTTGGGTTAGGGTTTAGGGTTGGGTTAGGGTTTAGGGTTTAGGGTTTAGGGTTTAGGGTTTAGGGTAGGGTTTAGGGGTTTAGGGTTTAGGGTTTAGGGTTAGGGTTTAGGGTTTAGGGGTTTAGGGTTAGGGTTTAGGGTAGGGTTTAGGGTTTTAGGGTTTAGGGTAGGTTTAGGGTTTAGGGTTTAGGGTTTAGGGTTTGTAGGGTTTAGGGTTTAGGGTTTAGGGTTTAGGGTTTAGGGTTTAGGGTTAGGGTTTAGGGTTTAGGGTTTAGGGTTT

***T.dicoccoides***

**3AS**

GAGGG**TTTAGGG**TTTAGGGTTTAGGGTTTAGGGTTTAGGGTTTAGGGTTTAGGGTTTTTAGGGTTTAGGGTTTA

**4AS**

TTG**TTTAGGG**TTTAGGGTTTAGGGTTTAGGGTTAGGGTTTAGGGTTTAGGGTTTAGGGTTTAGGGTTTAGGGTTTAGGGTTTAGGGTTTAGGGTTTAG

**6AS**

G**TTTAGGG**TTTAGGGTTTAGGGTTTAGGGTTTAGGGTTTAGGGTTTAGGGTTTAGGGTTTAGGGTTTAGGGTTTAGGGTTTAGGGTTTAGGGTTTAGGGTTTAGGGTTTAGGGTTTAGGGTTAAGGGTTTAGG

**7AS**

GTTAAGGGTTAAGGG**TTTAGGG**TTTAGGGTTTAGGGTTTAGGGTTTAGGTTTAGGGTTTAGGTTTAGGGTTTAGGGTTTAGGGTTTTAGGGTTTAGGGTTT

**4BS**

GTG**TTTAGGG**TTAGGTTTAGGGTTTAGGGTTTAGGGTTTAGGGTTTAGGTTTAGGGTTTAGGGTTTAGGGTTTAGGGTTTAGGGTTTAGGGTTTAGG

**7BS**

GGTTAGGG**TTTAGGG**TTAGGGTTTAGGGTTTAGGGTTTAGGGTTTAGGGTTTTTAGGGTTTAGGGTTTAGGGTTTAGGGTTTAGGGTTTAGGGTTAGGGTTTAGGGTTTAGGGTTTAGGGTTTA

***T.turgidum***

**7BS**

GGAATAGG**TTTAGGG**TTTAGGGTTTAGGGTTTTTAGGCTTTAGGGTTTAGGGTTTAGGGTTTAGGGTTTTTAGGGTTTAGGGTTTAGGGTTTAGGGTTTACGGTTTAGGGTTTAGGGTTTAGGGTTAGTTAGGGTTAGG

***A.tauschii* subsp.*strangulata***

**1DS**

CCAGGG**TTTAGGG**TTTAGGGTTTAGGGTTTAGGGTTTTAGGGTTTAGGGTTTAGGGTTTAGGGTTTAGGGTTTAGGGTTAGGGTTTAGGGTTAGGGTTTAGGGTTTAGGGTT

**5DL**

TTGG**TTTAGGG**TTTAGGGTTTAGGGTTTAGGGTTTAGGGTTAGGGTTTAGGGTTAGGGTTTAG

**6DL**

CACTTAGGG**TTTAGGG**TTGAGGGTTTAGGGTTTAGGGTTGGGGTTTAGGGTTTAGGGTTTAGGGTTTAGGGTTTAGGGTTTAGGGTTTAGGGTTT
