# Supplementary material for: Role of chromosome ends in meiotic stability, recombination and wheat evolution in the context of breeding
Source: BMC Plant Biol. 2025 Dec 29;26:187. doi: 10.1186/s12870-025-08020-5 (PMC12859859; doi:10.1186/s12870-025-08020-5)
Supplement: Supplementary file 2 — Supplementary Material 2 [file 12870_2025_8020_MOESM2_ESM.docx]

| **Additional file 2.** Mutations of the telomere sequence repeat. | | | | | | | | | | |
| --- | --- | --- | --- | --- | --- | --- | --- | --- | --- | --- |
| **Chromosome** | **Species/Cultivar** | **Telomere**  **(bp)** | **Substitutions** | | **Additions** | | **Deletions** | | **All mutations** | |
|  |  |  | **Number** | **%** | **Number** | **%** | **Number** | **%** | **Number** | **%** |
| 1AS | LongReach Lancer | 108 | 1 | 0.93 | 7 | 6.48 | 4 | 3.70 | 12 | 11.11 |
|  | CDC Landmark | 121 | 1 | 0.83 | 6 | 4.96 | 4 | 3.31 | 11 | 9.09 |
|  | Chinese Spring | 88 | 1 | 1.14 | 2 | 2.27 | 5 | 5.68 | 8 | 9.09 |
|  | Spelt | 107 | 1 | 0.93 | 6 | 5.61 | 4 | 3.74 | 11 | 10.28 |
|  | Fielder | 1390 | 8 | 0.58 | 20 | 1.44 | 41 | 2.95 | 69 | 4.96 |
|  | Kariega | 12143 | 10 | 0.08 | 77 | 0.63 | 254 | 2.09 | 341 | 2.81 |
| 1AL | Spelt | 82 | 0 | 0.00 | 1 | 1.22 | 3 | 3.66 | 4 | 4.88 |
|  | Kariega | 3657 | 10 | 0.27 | 26 | 0.71 | 80 | 2.19 | 116 | 3.17 |
| 2AS | CDC Landmark | 89 | 0 | 0.00 | 0 | 0.00 | 2 | 2.25 | 2 | 2.25 |
|  | SY Mattis | 106 | 0 | 0.00 | 3 | 2.83 | 2 | 1.89 | 5 | 4.72 |
|  | CDC Stanley | 109 | 0 | 0.00 | 1 | 0.92 | 4 | 3.67 | 5 | 4.59 |
|  | Jagger | 50 | 0 | 0.00 | 2 | 4.00 | 1 | 2.00 | 3 | 6.00 |
|  | Fielder | 2684 | 73 | 2.72 | 97 | 3.61 | 100 | 3.73 | 270 | 10.06 |
|  | Attraktion | 1015 | 1 | 0.10 | 7 | 0.69 | 27 | 2.66 | 35 | 3.45 |
|  | Kariega | 9292 | 12 | 0.13 | 67 | 0.72 | 227 | 2.44 | 306 | 3.29 |
| 3AS | SY Mattis | 97 | 1 | 1.03 | 1 | 1.03 | 9 | 9.28 | 11 | 11.34 |
|  | Spelt | 82 | 0 | 0.00 | 0 | 0.00 | 2 | 2.44 | 2 | 2.44 |
|  | Attraktion | 11495 | 27 | 0.23 | 99 | 0.86 | 304 | 2.64 | 430 | 3.74 |
|  | Kariega | 9870 | 6 | 0.06 | 43 | 0.44 | 232 | 2.35 | 281 | 2.85 |
|  | *T. dicoccoides* | 73 | 0 | 0.00 | 2 | 2.74 | 3 | 4.11 | 5 | 6.85 |
| 3AL | Kariega | 18882 | 25 | 0.13 | 481 | 2.55 | 418 | 2.21 | 924 | 4.89 |
| 4AS | ArinaLrFor | 160 | 4 | 2.50 | 0 | 0.00 | 8 | 5.00 | 12 | 7.50 |
|  | Chinese Spring | 85 | 0 | 0.00 | 2 | 2.35 | 1 | 1.18 | 3 | 3.53 |
|  | Norin-61 | 33 | 0 | 0.00 | 0 | 0.00 | 2 | 6.06 | 2 | 6.06 |
|  | Spelt | 77 | 0 | 0.00 | 2 | 2.60 | 2 | 2.60 | 4 | 5.19 |
|  | Attraktion | 4958 | 6 | 0.12 | 20 | 0.40 | 81 | 1.63 | 107 | 2.16 |
|  | Mace | 66 | 3 | 4.55 | 0 | 0.00 | 4 | 6.06 | 7 | 10.61 |
|  | Julius | 48 | 3 | 6.25 | 1 | 2.08 | 2 | 4.17 | 6 | 12.50 |
|  | *T. dicoccoides* | 98 | 0 | 0.00 | 0 | 0.00 | 3 | 3.06 | 3 | 3.06 |
| 5AL | Attraktion | 11488 | 46 | 0.40 | 162 | 1.41 | 264 | 2.30 | 472 | 4.11 |
|  | Kariega | 12639 | 22 | 0.17 | 72 | 0.57 | 366 | 2.90 | 460 | 3.64 |
| 6AS | Chinese Spring | 64 | 0 | 0.00 | 2 | 3.13 | 1 | 1.56 | 3 | 4.69 |
|  | *T. dicoccoides* | 133 | 1 | 0.75 | 0 | 0.00 | 1 | 0.75 | 2 | 1.50 |
| 6AL | Aikang58 | 364 | 8 | 2.20 | 32 | 8.79 | 25 | 6.87 | 65 | 17.86 |
|  | Spelt | 132 | 1 | 0.76 | 3 | 2.27 | 4 | 3.03 | 8 | 6.06 |
|  | Attraktion | 4176 | 4 | 0.10 | 34 | 0.81 | 84 | 2.01 | 122 | 2.92 |
|  | Kariega | 9035 | 6 | 0.07 | 35 | 0.39 | 249 | 2.76 | 290 | 3.21 |
| 7AS | SY Mattis | 124 | 1 | 0.81 | 1 | 0.81 | 3 | 2.42 | 5 | 4.03 |
|  | Aikang58 | 3256 | 28 | 0.86 | 120 | 3.69 | 171 | 5.25 | 319 | 9.80 |
|  | Chinese Spring | 133 | 1 | 0.75 | 1 | 0.75 | 8 | 6.02 | 10 | 7.52 |
|  | Spelt | 68 | 0 | 0.00 | 0 | 0.00 | 2 | 2.94 | 2 | 2.94 |
|  | Attraktion | 3854 | 13 | 0.34 | 39 | 1.01 | 151 | 3.92 | 203 | 5.27 |
|  | Renan | 2109 | 1 | 0.05 | 12 | 0.57 | 3 | 0.14 | 16 | 0.76 |
|  | *T. dicoccoides* | 101 | 0 | 0.00 | 4 | 3.96 | 2 | 1.98 | 6 | 5.94 |
| 7AL | Alchemy | 33 | 0 | 0.00 | 0 | 0.00 | 2 | 6.06 | 2 | 6.06 |
|  | Aikang58 | 135 | 3 | 2.22 | 2 | 1.48 | 7 | 5.19 | 12 | 8.89 |
|  | Attraktion | 3665 | 16 | 0.44 | 50 | 1.36 | 124 | 3.38 | 190 | 5.18 |
|  | Kariega | 4519 | 18 | 0.40 | 41 | 0.91 | 122 | 2.70 | 181 | 4.01 |
|  | Renan | 594 | 1 | 0.17 | 10 | 1.68 | 11 | 1.85 | 22 | 3.70 |
| 1BS | LongReach Lancer | 100 | 4 | 4.00 | 0 | 0.00 | 5 | 5.00 | 9 | 9.00 |
|  | SY Mattis | 129 | 0 | 0.00 | 1 | 0.78 | 5 | 3.88 | 6 | 4.65 |
|  | Spelt | 90 | 0 | 0.00 | 2 | 2.22 | 3 | 3.33 | 5 | 5.56 |
|  | Attraktion | 1756 | 2 | 0.11 | 13 | 0.74 | 28 | 1.59 | 43 | 2.45 |
|  | Kariega | 9484 | 60 | 0.63 | 168 | 1.77 | 317 | 3.34 | 545 | 5.75 |
| 2BS | Kariega | 6397 | 19 | 0.30 | 98 | 1.53 | 321 | 5.02 | 438 | 6.85 |
| 3BS | SY Mattis | 76 | 0 | 0.00 | 2 | 2.63 | 3 | 3.95 | 5 | 6.58 |
|  | Spelt | 48 | 0 | 0.00 | 0 | 0.00 | 1 | 2.08 | 1 | 2.08 |
| 3BL | Attraktion | 3735 | 12 | 0.32 | 45 | 1.20 | 62 | 1.66 | 119 | 3.19 |
|  | Kariega | 2000 | 8 | 0.40 | 8 | 0.40 | 24 | 1.20 | 40 | 2.00 |
| 4BS | LongReach Lancer | 91 | 2 | 2.20 | 2 | 2.20 | 2 | 2.20 | 6 | 6.59 |
|  | CDC Stanley | 82 | 1 | 1.22 | 0 | 0.00 | 2 | 2.44 | 3 | 3.66 |
|  | Mace | 69 | 0 | 0.00 | 0 | 0.00 | 1 | 1.45 | 1 | 1.45 |
|  | Kariega | 9657 | 11 | 0.11 | 52 | 0.54 | 367 | 3.80 | 430 | 4.45 |
|  | *T. dicoccoides* | 95 | 0 | 0.00 | 0 | 0.00 | 4 | 4.21 | 4 | 4.21 |
| 6BS | Chinese Spring | 147 | 1 | 0.68 | 3 | 2.04 | 3 | 2.04 | 7 | 4.76 |
| 7BS | Alchemy | 178 | 2 | 1.12 | 8 | 4.49 | 5 | 2.81 | 15 | 8.43 |
|  | Chinese Spring | 111 | 2 | 1.80 | 6 | 5.41 | 0 | 0.00 | 8 | 7.21 |
|  | Spelt | 118 | 1 | 0.85 | 0 | 0.00 | 8 | 6.78 | 9 | 7.63 |
|  | Mace | 116 | 2 | 1.72 | 4 | 3.45 | 0 | 0.00 | 6 | 5.17 |
|  | Julius | 69 | 1 | 1.45 | 6 | 8.70 | 0 | 0.00 | 7 | 10.14 |
|  | *T. turgidum* | 139 | 2 | 1.44 | 4 | 2.88 | 6 | 4.32 | 12 | 8.63 |
|  | *T. dicoccoides* | 124 | 0 | 0.00 | 2 | 1.61 | 5 | 4.03 | 7 | 5.65 |
| 1DS | Spelt | 82 | 0 | 0.00 | 1 | 1.22 | 3 | 3.66 | 4 | 4.88 |
|  | Attraktion | 5398 | 14 | 0.26 | 49 | 0.91 | 187 | 3.46 | 250 | 4.63 |
|  | Kariega | 9906 | 7 | 0.07 | 19 | 0.19 | 207 | 2.09 | 233 | 2.35 |
|  | *A. tauschii* | 110 | 0 | 0.00 | 3 | 2.73 | 2 | 1.82 | 5 | 4.55 |
| 1DL | Alchemy | 90 | 0 | 0.00 | 0 | 0.00 | 1 | 1.11 | 1 | 1.11 |
| 2DS | Kariega | 18858 | 17 | 0.09 | 137 | 0.73 | 531 | 2.82 | 685 | 3.63 |
| 2DL | Attraktion | 1280 | 4 | 0.31 | 19 | 1.48 | 20 | 1.56 | 43 | 3.36 |
|  | Kariega | 14043 | 9 | 0.06 | 47 | 0.33 | 332 | 2.36 | 388 | 2.76 |
| 3DL | Attraktion | 12322 | 33 | 0.27 | 113 | 0.92 | 381 | 3.09 | 527 | 4.28 |
|  | Kariega | 6235 | 33 | 0.53 | 106 | 1.70 | 163 | 2.61 | 302 | 4.84 |
| 4DL | Aikang58 | 5430 | 40 | 0.74 | 173 | 3.19 | 310 | 5.71 | 523 | 9.63 |
|  | Spelt | 88 | 0 | 0.00 | 0 | 0.00 | 3 | 3.41 | 3 | 3.41 |
| 5DS | Kariega | 3714 | 6 | 0.16 | 55 | 1.48 | 150 | 4.04 | 211 | 5.68 |
| 5DL | Attraktion | 16898 | 10 | 0.06 | 117 | 0.69 | 301 | 1.78 | 428 | 2.53 |
|  | Kariega | 11335 | 9 | 0.08 | 24 | 0.21 | 302 | 2.66 | 335 | 2.96 |
|  | A. tauschii | 63 | 0 | 0.00 | 0 | 0.00 | 4 | 6.35 | 4 | 6.35 |
| 6DS | ArinaLrFor | 67 | 0 | 0.00 | 0 | 0.00 | 3 | 4.48 | 3 | 4.48 |
| 6DL | Attraktion | 6405 | 11 | 0.17 | 109 | 1.70 | 171 | 2.67 | 291 | 4.54 |
|  | Kariega | 11108 | 18 | 0.16 | 104 | 0.94 | 339 | 3.05 | 461 | 4.15 |
|  | A. tauschii | 92 | 2 | 2.17 | 3 | 3.26 | 1 | 1.09 | 6 | 6.52 |
| 7DS | LongReach Lancer | 51 | 0 | 0.00 | 3 | 5.88 | 1 | 1.96 | 4 | 7.84 |
|  | Chinese Spring | 85 | 0 | 0.00 | 2 | 2.35 | 1 | 1.18 | 3 | 3.53 |
|  | Kariega | 11560 | 11 | 0.10 | 105 | 0.91 | 318 | 2.75 | 434 | 3.75 |
| 7DL | Kariega | 7783 | 15 | 0.19 | 53 | 0.68 | 244 | 3.14 | 312 | 4.01 |
