# Supplementary material for: Role of chromosome ends in meiotic stability, recombination and wheat evolution in the context of breeding
Source: BMC Plant Biol. 2025 Dec 29;26:187. doi: 10.1186/s12870-025-08020-5 (PMC12859859; doi:10.1186/s12870-025-08020-5)
Supplement: Supplementary file 3 — Supplementary Material 3 [file 12870_2025_8020_MOESM3_ESM.docx]

**Additional file 3.** Number and frequency of G4 quadruplexes in the telomeric sequence of diploid, tetraploid and hexaploid wheat chromosomes arms.

| **Chromosome** | **Species/Cultivar** | **Telomere size (bp)** | **Number** | **G4 (Nr./Kb)** |
| --- | --- | --- | --- | --- |
| 1AS | LongReach Lancer | 135 | 8 | 59.3 |
|  | CDC Landmark | 148 | 9 | 60.8 |
|  | Chinese Spring | 112 | 5 | 44.6 |
|  | Spelt | 129 | 8 | 62 |
|  | Fielder | 1414 | 112 | 79.2 |
|  | Kariega | 12152 | 1158 | 95.3 |
| 1AL | Spelt | 85 | 6 | 70.6 |
|  | Kariega | 3660 | 288 | 78.7 |
| 2AS | CDC Landmark | 91 | 10 | 109.9 |
|  | SY Mattis | 112 | 12 | 107.1 |
|  | CDC Stanley | 115 | 12 | 104.3 |
|  | Jagger | 54 | 4 | 74.1 |
|  | Fielder | 2686 | 214 | 79.7 |
|  | Attraktion | 1025 | 68 | 66.3 |
|  | Kariega | 9292 | 858 | 92.3 |
| 3AS | SY Mattis | 105 | 8 | 76.2 |
|  | Spelt | 83 | 3 | 36.1 |
|  | Attraktion | 11505 | 944 | 82.1 |
|  | Kariega | 9879 | 876 | 88.7 |
|  | T. dicoccoides | 73 | 7 | 95.9 |
| 3AL | Kariega | 18891 | 1889 | 100 |
| 4AS | ArinaLrFor | 163 | 9 | 55.2 |
|  | Chinese Spring | 92 | 6 | 65.2 |
|  | Norin-61 | 40 | 2 | 50 |
|  | Spelt | 81 | 4 | 49.4 |
|  | Attraktion | 4961 | 491 | 99 |
|  | Mace | 69 | 1 | 14.5 |
|  | Julius | 51 | 1 | 19.6 |
|  | T. dicoccoides | 98 | 6 | 61.2 |
| 5AL | Attraktion | 11488 | 964 | 83.9 |
|  | Kariega | 12639 | 943 | 74.6 |
| 6AS | Chinese Spring | 65 | 6 | 92.3 |
|  | T. dicoccoides | 133 | 16 | 120.3 |
| 6AL | Aikang58 | 369 | 15 | 40.7 |
|  | Spelt | 136 | 7 | 51.5 |
|  | Attraktion | 4178 | 394 | 94.3 |
|  | Kariega | 9040 | 690 | 76.3 |
| 7AS | SY Mattis | 131 | 3 | 22.9 |
|  | Aikang58 | 3270 | 231 | 70.6 |
|  | Chinese Spring | 141 | 9 | 63.8 |
|  | Spelt | 77 | 2 | 26 |
|  | Attraktion | 3868 | 277 | 71.6 |
|  | Renan | 2113 | 286 | 135.4 |
|  | T. dicoccoides | 101 | 9 | 89.1 |
| 7AL | Alchemy | 39 | 2 | 51.3 |
|  | Aikang58 | 138 | 11 | 79.7 |
|  | Attraktion | 3674 | 247 | 67.2 |
|  | Kariega | 4523 | 328 | 72.5 |
|  | Renan | 594 | 64 | 107.7 |
| 1BS | LongReach Lancer | 103 | 10 | 97.1 |
|  | SY Mattis | 133 | 7 | 52.6 |
|  | Spelt | 94 | 6 | 63.8 |
|  | Attraktion | 1760 | 166 | 94.3 |
|  | Kariega | 9488 | 701 | 73.9 |
| 2BS | Kariega | 6399 | 414 | 64.7 |
| 3BS | SY Mattis | 79 | 8 | 101.3 |
|  | Spelt | 52 | 5 | 96.2 |
| 3BL | Attraktion | 4482 | 364 | 81.2 |
|  | Kariega | 2004 | 238 | 118.8 |
| 4BS | LongReach Lancer | 91 | 6 | 65.9 |
|  | CDC Stanley | 84 | 5 | 59.5 |
|  | Mace | 70 | 4 | 57.1 |
|  | Kariega | 9663 | 766 | 79.3 |
|  | T. dicoccoides | 95 | 11 | 115.8 |
| 6BS | Chinese Spring | 148 | 6 | 40.5 |
| 7BS | Alchemy | 186 | 19 | 102.2 |
|  | Chinese Spring | 119 | 11 | 92.4 |
|  | Spelt | 121 | 4 | 33.1 |
|  | Mace | 124 | 12 | 96.8 |
|  | Julius | 76 | 5 | 65.8 |
|  | T. turgidum | 139 | 14 | 100.7 |
|  | T. dicoccoides | 124 | 8 | 64.5 |
| 1DS | Spelt | 86 | 3 | 34.9 |
|  | Attraktion | 5403 | 437 | 80.9 |
|  | Kariega | 9911 | 906 | 91.4 |
|  | A. tauschii | 110 | 8 | 72.7 |
| 1DL | Alchemy | 111 | 12 | 108.1 |
| 2DS | Kariega | 18859 | 1468 | 77.8 |
| 2DL | Attraktion | 1292 | 134 | 103.7 |
|  | Kariega | 14055 | 1411 | 100.4 |
| 3DL | Attraktion | 12330 | 952 | 77.2 |
|  | Kariega | 6240 | 533 | 85.4 |
| 4DL | Aikang58 | 5430 | 416 | 76.6 |
|  | Spelt | 88 | 1 | 11.4 |
| 5DS | Kariega | 3722 | 236 | 63.4 |
| 5DL | Attraktion | 16906 | 1605 | 94.9 |
|  | Kariega | 11346 | 846 | 74.6 |
|  | A. tauschii | 63 | 3 | 47.6 |
| 6DS | ArinaLrFor | 82 | 7 | 85.4 |
| 6DL | Attraktion | 6411 | 549 | 85.6 |
|  | Kariega | 11114 | 828 | 74.5 |
|  | A. tauschii | 92 | 6 | 65.2 |
| 7DS | LongReach Lancer | 54 | 4 | 74.1 |
|  | Chinese Spring | 99 | 6 | 60.6 |
|  | Kariega | 11563 | 853 | 73.8 |
| 7DL | Kariega | 7790 | 596 | 76.5 |
