# Supplementary figures and images for: Role of chromosome ends in meiotic stability, recombination and wheat evolution in the context of breeding
Source: BMC Plant Biol. 2025 Dec 29;26:187. doi: 10.1186/s12870-025-08020-5 (PMC12859859; doi:10.1186/s12870-025-08020-5)

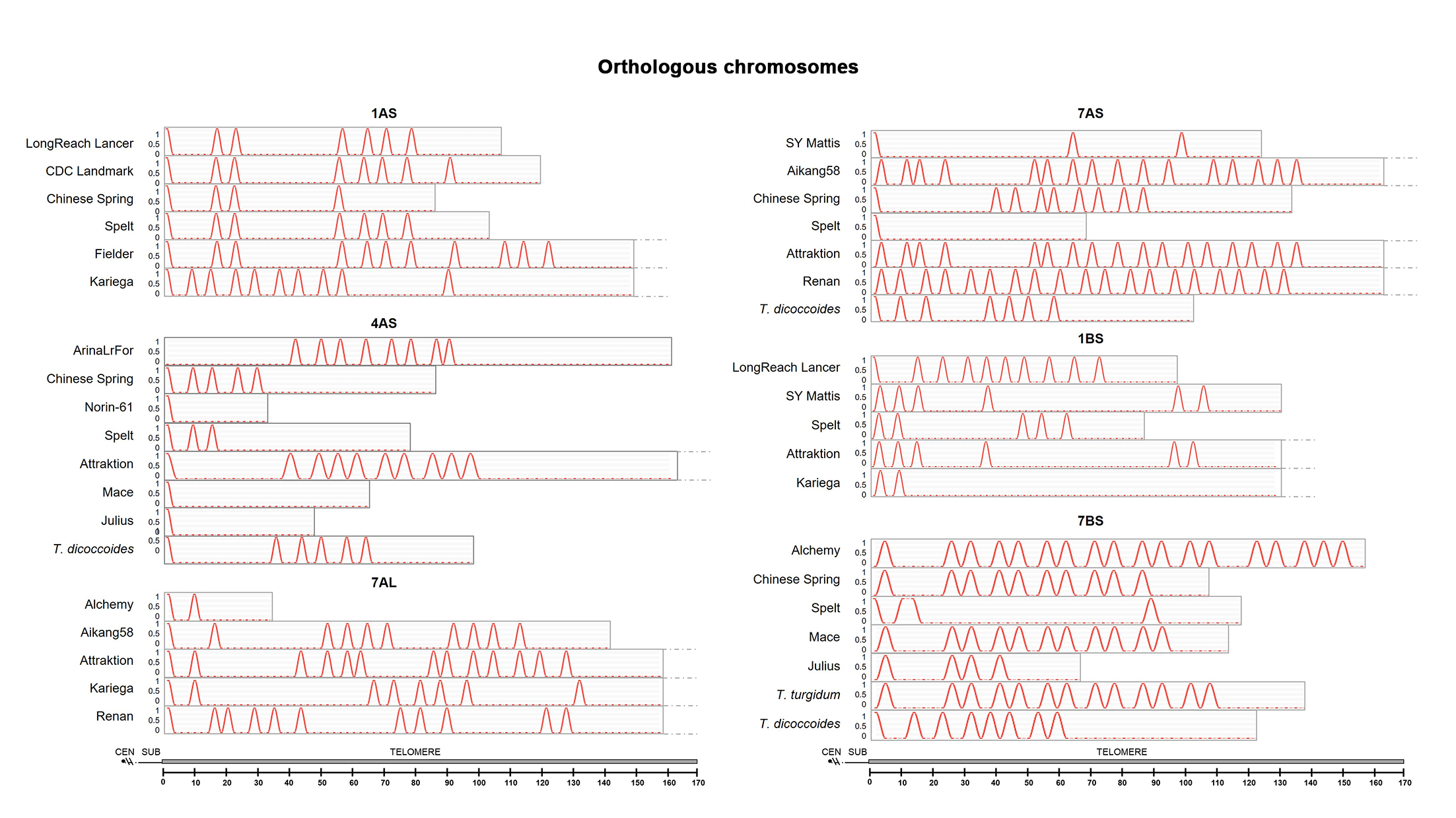

Supplement: Supplementary file 4 — Supplementary Material 4 [file 12870_2025_8020_MOESM4_ESM.tif]

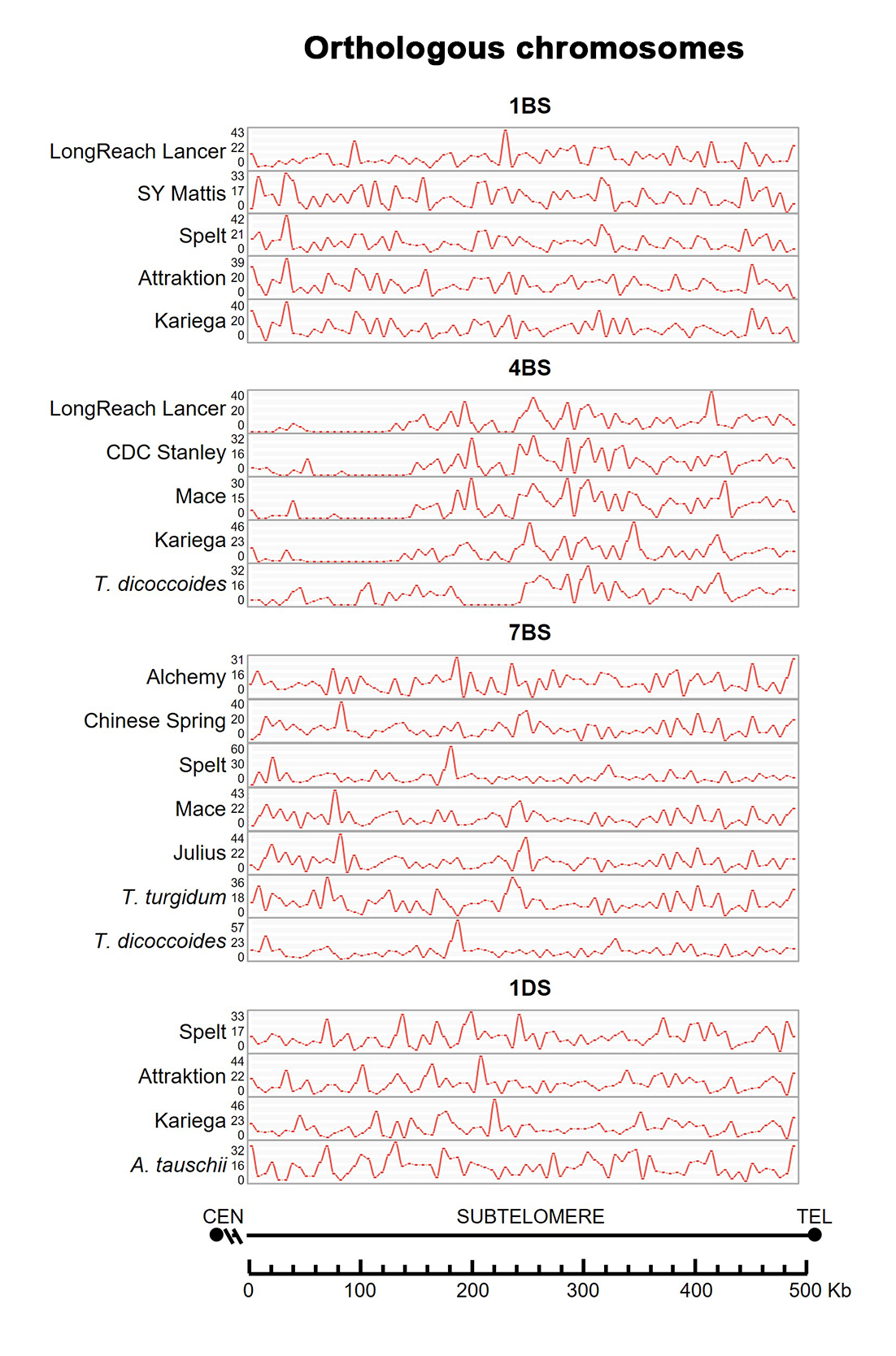

Supplement: Supplementary file 6 — Supplementary Material 6 [file 12870_2025_8020_MOESM6_ESM.tif]

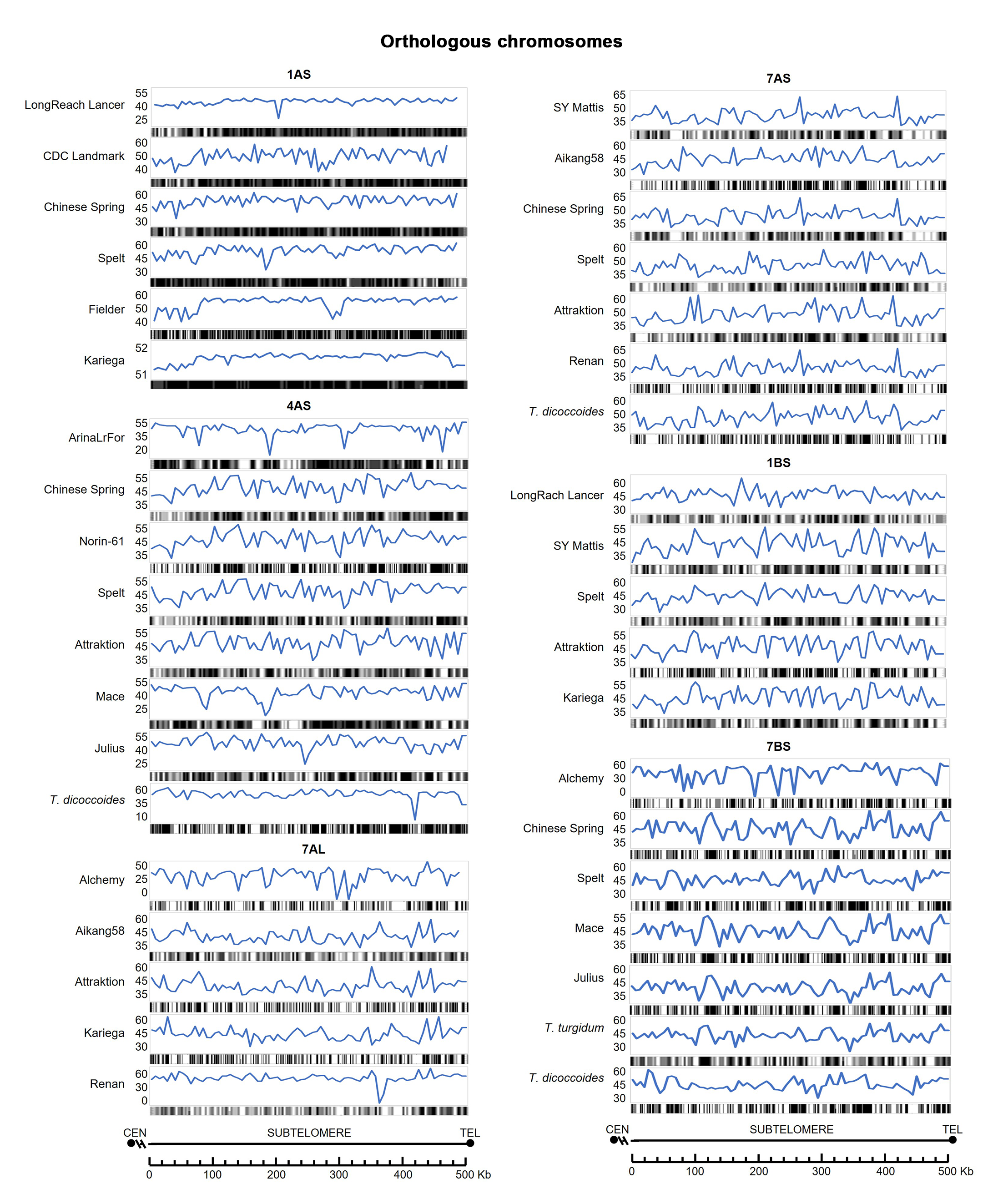

Supplement: Supplementary file 9 — Supplementary Material 9 [file 12870_2025_8020_MOESM9_ESM.tif]

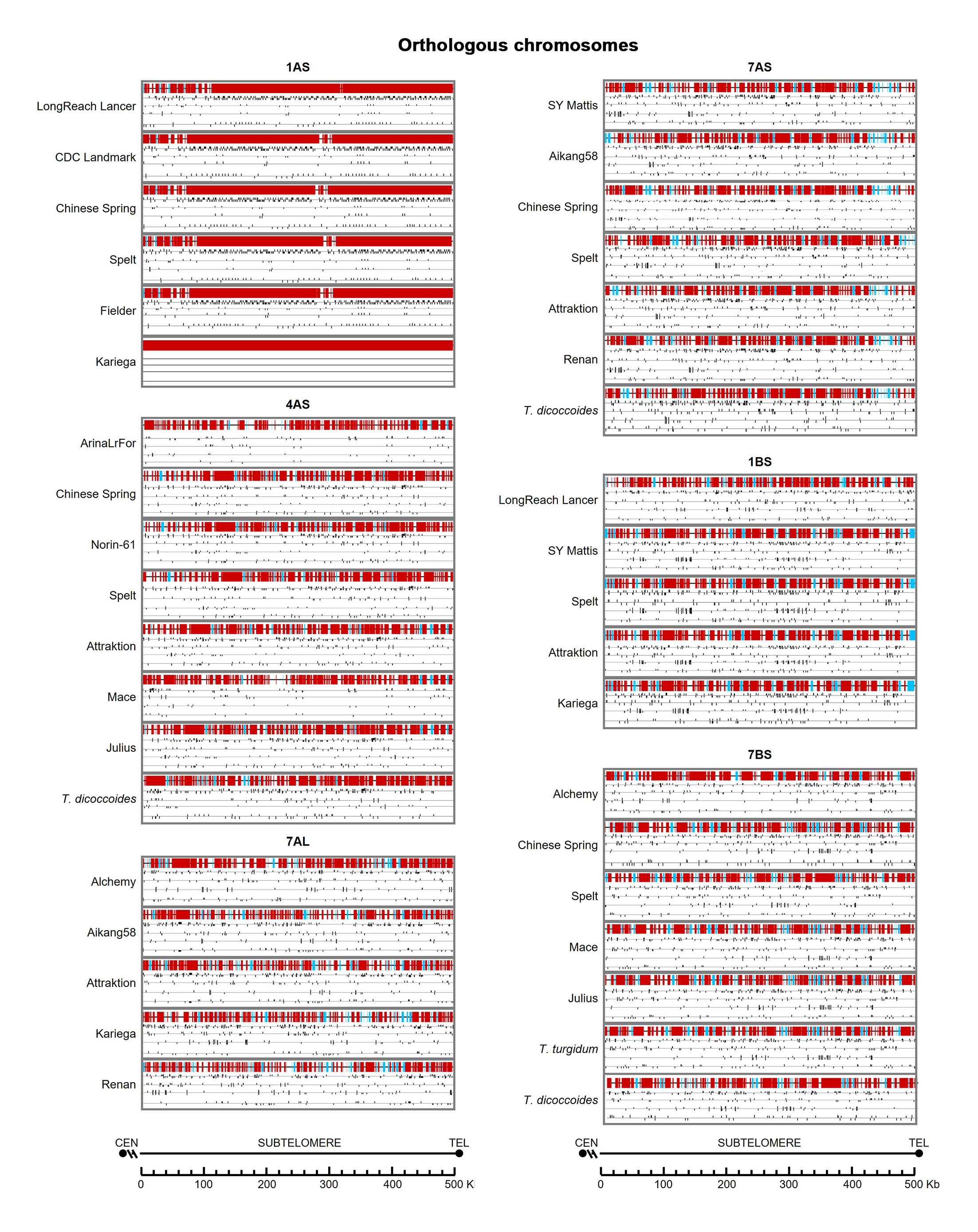

Supplement: Supplementary file 10 — Supplementary Material 10 [file 12870_2025_8020_MOESM10_ESM.tif]
