# Supplementary material for: Role of chromosome ends in meiotic stability, recombination and wheat evolution in the context of breeding
Source: BMC Plant Biol. 2025 Dec 29;26:187. doi: 10.1186/s12870-025-08020-5 (PMC12859859; doi:10.1186/s12870-025-08020-5)
Supplement: Supplementary file 5 — Supplementary Material 5 [file 12870_2025_8020_MOESM5_ESM.docx]

**Additional file 5.** Number and frequency of G4 quadruplexes in the distal 500 kb from the subtelomeric region adjacent to the telomere.

| **Chromosome** | **Species/Cultivar** | **Nº** | **G4 (nº/Kb)** |
| --- | --- | --- | --- |
| 1AS | LongReach Lancer | 1125 | 2.3 |
|  | CDC Landmark | 1148 | 2.3 |
|  | Chinese Spring | 1158 | 2.3 |
|  | Spelt | 1142 | 2.3 |
|  | Fielder | 1240 | 2.5 |
|  | Kariega | 0 | 0 |
| 1AL | Spelt | 860 | 1.7 |
|  | Kariega | 1042 | 2.1 |
| 2AS | CDC Landmark | 711 | 1.4 |
|  | SY Mattis | 906 | 1.8 |
|  | CDC Stanley | 841 | 1.7 |
|  | Jagger | 843 | 1.7 |
|  | Fielder | 768 | 1.5 |
|  | Attraktion | 707 | 1.4 |
|  | Kariega | 707 | 1.4 |
| 3AS | SY Mattis | 660 | 1.3 |
|  | Spelt | 636 | 1.3 |
|  | Attraktion | 643 | 1.3 |
|  | Kariega | 675 | 1.3 |
|  | *T. dicoccoides* | 905 | 1.8 |
| 3AL | Kariega | 983 | 2 |
| 4AS | ArinaLrFor | 1365 | 2.7 |
|  | Chinese Spring | 1269 | 2.5 |
|  | Norin-61 | 1244 | 2.5 |
|  | Spelt | 1252 | 2.5 |
|  | Attraktion | 1296 | 2.6 |
|  | Mace | 1356 | 2.7 |
|  | Julius | 1173 | 2.3 |
|  | *T. dicoccoides* | 1361 | 2.7 |
| 5AL | Attraktion | 901 | 1.8 |
|  | Kariega | 910 | 1.8 |
| 6AS | Chinese Spring | 983 | 2 |
|  | T. dicoccoides | 848 | 1.7 |
| 6AL | Aikang58 | 939 | 1.9 |
|  | Spelt | 947 | 1.9 |
|  | Attraktion | 26 | 0.1 |
|  | Kariega | 973 | 1.9 |
| 7AS | SY Mattis | 991 | 2 |
|  | Aikang58 | 890 | 1.8 |
|  | Chinese Spring | 1012 | 2 |
|  | Spelt | 971 | 1.9 |
|  | Attraktion | 910 | 1.8 |
|  | Renan | 1035 | 2.1 |
|  | T. dicoccoides | 860 | 1.7 |
| 7AL | Alchemy | 787 | 1.6 |
|  | Aikang58 | 1107 | 2.2 |
|  | Attraktion | 1101 | 2.2 |
|  | Kariega | 1071 | 2.1 |
|  | Renan | 1041 | 2.1 |
| 1BS | LongReach Lancer | 1015 | 2 |
|  | SY Mattis | 1021 | 2 |
|  | Spelt | 1014 | 2 |
|  | Attraktion | 1052 | 2.1 |
|  | Kariega | 1053 | 2.1 |
| 2BS | Kariega | 241 | 0.5 |
| 3BS | SY Mattis | 975 | 1.9 |
|  | Spelt | 974 | 1.9 |
| 3BL | Attraktion | 1085 | 2.2 |
|  | Kariega | 1082 | 2.2 |
| 4BS | LongReach Lancer | 715 | 1.4 |
|  | CDC Stanley | 717 | 1.4 |
|  | Mace | 705 | 1.4 |
|  | Kariega | 760 | 1.5 |
|  | *T. dicoccoides* | 703 | 1.4 |
| 6BS | Chinese Spring | 1033 | 2.1 |
| 7BS | Alchemy | 888 | 1.8 |
|  | Chinese Spring | 1001 | 2 |
|  | Spelt | 1001 | 2 |
|  | Mace | 1012 | 2 |
|  | Julius | 1008 | 2 |
|  | *T. turgidum* | 1024 | 2 |
|  | *T. dicoccoides* | 964 | 1.9 |
| 1DS | Spelt | 998 | 2 |
|  | Attraktion | 1025 | 2 |
|  | Kariega | 1006 | 2 |
|  | *A. tauschii* | 1006 | 2 |
| 1DL | Alchemy | 813 | 1.6 |
| 2DS | Kariega | 0 | 0 |
| 2DL | Attraktion | 1008 | 2 |
|  | Kariega | 1009 | 2 |
| 3DL | Attraktion | 957 | 1.9 |
|  | Kariega | 953 | 1.9 |
| 4DL | Aikang58 | 964 | 1.9 |
|  | Spelt | 947 | 1.9 |
| 5DS | Kariega | 321 | 0.6 |
| 5DL | Attraktion | 829 | 1.7 |
|  | Kariega | 938 | 1.9 |
|  | A. tauschii | 924 | 1.8 |
| 6DS | ArinaLrFor | 879 | 1.8 |
| 6DL | Attraktion | 1137 | 2.3 |
|  | Kariega | 1136 | 2.3 |
|  | A. tauschii | 1085 | 2.2 |
| 7DS | LongReach Lancer | 721 | 1.4 |
|  | Chinese Spring | 792 | 1.6 |
|  | Kariega | 690 | 1.4 |
| 7DL | Kariega | 1067 | 2.1 |
