# Supplementary material for: Role of chromosome ends in meiotic stability, recombination and wheat evolution in the context of breeding
Source: BMC Plant Biol. 2025 Dec 29;26:187. doi: 10.1186/s12870-025-08020-5 (PMC12859859; doi:10.1186/s12870-025-08020-5)
Supplement: Supplementary file 7 — Supplementary Material 7 [file 12870_2025_8020_MOESM7_ESM.docx]

**Additional file 7.** Number and density of the genes identified in the distal 500 Kb and 5 M of the subtelomeric region of diploid, tetraploid and hexaploid wheat chromosomes.

| **Chromosome** | **Species/Cultivar** | **Nr. Genes** | | **Gene density** | |
| --- | --- | --- | --- | --- | --- |
|  |  | **500 Kb** | **5Mb** | **500 Kb** | **5 Mb** |
| 1AS | LongReach Lancer | 35 | 150 | 1 per 14.3 Kb | 1 per 33.3 Kb |
|  | CDC Landmark | 32 | 129 | 1 per 15.6 Kb | 1 per 38.8 Kb |
|  | Chinese Spring | 17 | 82 | 1 per 29.4 Kb | 1 per 61.0 Kb |
|  | Spelt |  |  |  |  |
|  | Fielder |  |  |  |  |
|  | Kariega | 0 | 70 | 1 per 0.0 Kb | 1 per 71.4 Kb |
| 1AL | Spelt |  |  |  |  |
|  | Kariega | 1 | 65 | 1 per 500.0 Kb | 1 per 76.9 Kb |
| 2AS | CDC Landmark | 8 | 123 | 1 per 62.5 Kb | 1 per 40.7 Kb |
|  | SY Mattis | 11 | 107 | 1 per 45.5 Kb | 1 per 46.7 Kb |
|  | CDC Stanley | 0 | 1 | 1 per 0.0 Kb | 1 per 5000.0 Kb |
|  | Jagger | 11 | 103 | 1 per 45.5 Kb | 1 per 48.5 Kb |
|  | Fielder |  |  |  |  |
|  | Attraktion |  |  |  |  |
|  | Kariega | 7 | 92 | 1 per 71.4 Kb | 1 per 54.3 Kb |
| 3AS | SY Mattis | 7 | 118 | 1 per 71.4 Kb | 1 per 42.4 Kb |
|  | Spelt |  |  |  |  |
|  | Attraktion |  |  |  |  |
|  | Kariega | 4 | 85 | 1 per 125.0 Kb | 1 per 58.8 Kb |
|  | *T. dicoccoides* | 5 | 97 | 1 per 100.0 Kb | 1 per 51.5 Kb |
| 3AL | Kariega | 2 | 73 | 1 per 250.0 Kb | 1 per 68.5 Kb |
| 4AS | ArinaLrFor | 17 | 100 | 1 per 29.4 Kb | 1 per 50.0 Kb |
|  | Chinese Spring | 6 | 81 | 1 per 83.3 Kb | 1 per 61.7 Kb |
|  | Norin-61 | 5 | 97 | 1 per 100.0 Kb | 1 per 51.5 Kb |
|  | Spelt |  |  |  |  |
|  | Attraktion |  |  |  |  |
|  | Mace | 17 | 101 | 1 per 29.4 Kb | 1 per 49.5 Kb |
|  | Julius | 17 | 110 | 1 per 29.4 Kb | 1 per 45.5 Kb |
|  | *T. dicoccoides* | 0 | 63 | 1 per 0.0 Kb | 1 per 79.4 Kb |
| 5AL | Attraktion |  |  |  |  |
|  | Kariega | 5 | 82 | 1 per 100.0 Kb | 1 per 61.0 Kb |
| 6AS | Chinese Spring | 4 | 95 | 1 per 125.0 Kb | 1 per 52.6 Kb |
|  | T. dicoccoides | 5 | 83 | 1 per 100.0 Kb | 1 per 60.2 Kb |
| 6AL | Aikang58 |  |  |  |  |
|  | Spelt |  |  |  |  |
|  | Attraktion |  |  |  |  |
|  | Kariega | 4 | 79 | 1 per 125.0 Kb | 1 per 63.3 Kb |
| 7AS | SY Mattis | 10 | 144 | 1 per 50.0 Kb | 1 per 34.7 Kb |
|  | Aikang58 |  |  |  |  |
|  | Chinese Spring | 6 | 104 | 1 per 83.3 Kb | 1 per 48.1 Kb |
|  | Spelt |  |  |  |  |
|  | Attraktion |  |  |  |  |
|  | Renan | 5 | 74 | 1 per 100.0 Kb | 1 per 67.6 Kb |
|  | T. dicoccoides | 8 | 95 | 1 per 62.5 Kb | 1 per 52.6 Kb |
| 7AL | Alchemy |  |  |  |  |
|  | Aikang58 |  |  |  |  |
|  | Attraktion |  |  |  |  |
|  | Kariega | 15 | 80 | 1 per 33.3 Kb | 1 per 62.5 Kb |
|  | Renan | 5 | 78 | 1 per 100.0 Kb | 1 per 64.1 Kb |
| 1BS | LongReach Lancer | 5 | 79 | 1 per 100.0 Kb | 1 per 63.3 Kb |
|  | SY Mattis | 2 | 108 | 1 per 250.0 Kb | 1 per 46.3 Kb |
|  | Spelt |  |  |  |  |
|  | Attraktion |  |  |  |  |
|  | Kariega | 1 | 84 | 1 per 500.0 Kb | 1 per 59.5 Kb |
| 2BS | Kariega | 4 | 56 | 1 per 125.0 Kb | 1 per 89.3 Kb |
| 3BS | SY Mattis | 10 | 132 | 1 per 50.0 Kb | 1 per 37.9 Kb |
|  | Spelt |  |  |  |  |
| 3BL | Attraktion |  |  |  |  |
|  | Kariega | 16 | 56 | 1 per 31.3 Kb | 1 per 89.3 Kb |
| 4BS | LongReach Lancer | 4 | 103 | 1 per 125.0 Kb | 1 per 48.5 Kb |
|  | CDC Stanley | 4 | 92 | 1 per 125.0 Kb | 1 per 54.3 Kb |
|  | Mace | 4 | 91 | 1 per 125.0 Kb | 1 per 54.9 Kb |
|  | Kariega | 3 | 57 | 1 per 166.7 Kb | 1 per 87.7 Kb |
|  | *T. dicoccoides* | 9 | 87 | 1 per 55.6 Kb | 1 per 57.5 Kb |
| 6BS | Chinese Spring | 6 | 67 | 1 per 83.3 Kb | 1 per 74.6 Kb |
| 7BS | Alchemy |  |  |  |  |
|  | Chinese Spring | 15 | 77 | 1 per 33.3 Kb | 1 per 64.9 Kb |
|  | Spelt |  |  |  |  |
|  | Mace | 15 | 109 | 1 per 33.3 Kb | 1 per 45.9 Kb |
|  | Julius | 17 | 118 | 1 per 29.4 Kb | 1 per 42.4 Kb |
|  | *T. turgidum* | 15 | 84 | 1 per 33.3 Kb | 1       per 59.5 Kb |
|  | *T. dicoccoides* | 11 | 58 | 1 per 45.5 Kb | 1 per 86.2 Kb |
| 1DS | Spelt |  |  |  |  |
|  | Attraktion |  |  |  |  |
|  | Kariega | 13 | 81 | 1 per 38.5 Kb | 1 per 61.7 Kb |
|  | *A. tauschii* | 18 | 100 | 1 per 27.8 Kb | 1 per 50.0 Kb |
| 1DL | Alchemy |  |  |  |  |
| 2DS | Kariega | 0 | 38 | 1 per 0.0 Kb | 1 per 131.6 Kb |
| 2DL | Attraktion |  |  |  |  |
|  | Kariega | 24 | 110 | 1 per 20.8 Kb | 1 per 45.5 Kb |
| 3DL | Attraktion |  |  |  |  |
|  | Kariega | 9 | 63 | 1 per 55.6 Kb | 1 per 79.4 Kb |
| 4DL | Aikang58 |  |  |  |  |
|  | Spelt |  |  |  |  |
| 5DS | Kariega | 1 | 63 | 1 per 500.0 Kb | 1 per 79.4 Kb |
| 5DL | Attraktion |  |  |  |  |
|  | Kariega | 6 | 92 | 1 per 83.3 Kb | 1 per 54.3 Kb |
|  | A. tauschii | 6 | 116 | 1 per 83.3 Kb | 1 per 43.1 Kb |
| 6DS | ArinaLrFor | 8 | 154 | 1 per 62.5 Kb | 1 per 32.5 Kb |
| 6DL | Attraktion |  |  |  |  |
|  | Kariega | 9 | 82 | 1 per 55.6 Kb | 1 per 61.0 Kb |
|  | A. tauschii | 7 | 104 | 1 per 71.4 Kb | 1 per 48.1 Kb |
| 7DS | LongReach Lancer | 7 | 125 | 1 per 71.4 Kb | 1 per 40.0 Kb |
|  | Chinese Spring | 6 | 93 | 1 per 83.3 Kb | 1 per 53.8 Kb |
|  | Kariega | 5 | 103 | 1 per 100.0 Kb | 1 per 48.5 Kb |
| 7DL | Kariega | 28 | 104 | 1 per 17.9 Kb | 1 per 48.1 Kb |
