# Supplementary material for: Role of chromosome ends in meiotic stability, recombination and wheat evolution in the context of breeding
Source: BMC Plant Biol. 2025 Dec 29;26:187. doi: 10.1186/s12870-025-08020-5 (PMC12859859; doi:10.1186/s12870-025-08020-5)
Supplement: Supplementary file 8 — Supplementary Material 8 [file 12870_2025_8020_MOESM8_ESM.docx]

**Additional file 9.** Repeat DNA sequences identified in the 500 Kb distal subtelomere region adjacent to telomeric repeats of diploid, tetraploid and hexaploid wheat chromosomes arms. *Satellites*: multiple copies of the same DNA sequence, the repeated pattern can vary in length for a single base to several thousand bases long; *SSRs*: duplications of simple sets of DNA bases (2–5 bp) such as A, CA, CGG etc.; *Low complexity*: Poly-purine or poly-pyrimidine stretches and regions of extremely high AT or CG content.

| **Chromosome** | **Species/Cultivar** | **Element** | **Number** | **Length (bp)** | **%** |
| --- | --- | --- | --- | --- | --- |
| 1AS | LongReach Lancer | Satellites  SSRs  Low complexity  Total | 0  28  3  31 | 0  1370  122  1492 | 0  0.27  0.02  0.29 |
|  | CDC Landmark | Satellites  SSRs  Low complexity  Total | 0  17  3  20 | 0  809  150  959 | 0  0.16  0.03  0.19 |
|  | Chinese Spring | Satellites  SSRs  Low complexity  Total | 0  20  3  23 | 0  1206  139  1345 | 0  0.24  0.03  0.27 |
|  | Spelt | Satellites  SSRs  Low complexity  Total | 0  25  2  27 | 0  1378  79  1457 | 0  0.28  0.02  0.3 |
|  | Fielder | Satellites  SSRs  Low complexity  Total | 0  17  2  19 | 0  1068  86  1154 | 0  0.21  0.02  0.23 |
|  | Kariega | Satellites  SSRs  Low complexity  Total | 1958  0  0  1958 | 494918  0  0  494918 | 98.98  0  0  98.98 |
| 1AL | Spelt | Satellites  SSRs  Low complexity  Total | 0  139  20  159 | 0  6266  964  7230 | 0  1.25  0.19  1.44 |
|  | Kariega | Satellites  SSRs  Low complexity  Total | 0  113  28  141 | 0  5253  1418  6671 | 0  1.05  0.28  1.33 |
| 2AS | CDC Landmark | Satellites  SSRs  Low complexity  Total | 0  128  14  142 | 0  5262  578  5840 | 0  1.05  0.12  1.17 |
|  | SY Mattis | Satellites  SSRs  Low complexity  Total | 0  162  31  193 | 0  7660  2207  9867 | 0  1.53  0.44  1.97 |
|  | CDC Stanley | Satellites  SSRs  Low complexity  Total | 0  150  29  179 | 0  6272  1941  8213 | 0  1.25  0.39  1.64 |
|  | Jagger | Satellites  SSRs  Low complexity  Total | 0  154  30  184 | 0  7037  2180  9217 | 0  1.41  0.44  1.85 |
|  | Fielder | Satellites  SSRs  Low complexity  Total | 0  135  18  153 | 0  5933  852  6785 | 0  1.19  0.17  1.36 |
|  | Attraktion | Satellites  SSRs  Low complexity  Total | 0  130  17  147 | 0  5308  782  6090 | 0  1.06  0.16  1.22 |
|  | Kariega | Satellites  SSRs  Low complexity  Total | 0  130  17  147 | 0  5427  781  6208 | 0  1.09  0.16  1.25 |
| 3AS | SY Mattis | Satellites  SSRs  Low complexity  Total | 0  89  31  120 | 0  3995  1380  5375 | 0  0.80  0.28  1.08 |
|  | Spelt | Satellites  SSRs  Low complexity  Total | 0  96  17  113 | 0  4668  805  5473 | 0  0.93  0.16  1.09 |
|  | Attraktion | Satellites  SSRs  Low complexity  Total | 0  97  18  115 | 0  4398  844  5242 | 0  0.88  0.17  1.05 |
|  | Kariega | Satellites  SSRs  Low complexity  Total | 0  101  28  129 | 0  4343  1323  5666 | 0  0.87  0.26  1.13 |
|  | *T. dicoccoides* | Satellites  SSRs  Low complexity  Total | 0  113  32  145 | 0  5268  1605  6873 | 0  1.05  0.32  1.37 |
| 3AL | Kariega | Satellites  SSRs  Low complexity  Total | 0  109  19  128 | 0  6401  979  7380 | 0  1.28  0.20  1.48 |
| 4AS | ArinaLrFor | Satellites  SSRs  Low complexity  Total | 916  46  12  974 | 207994  2378  642  211014 | 41.61  0.48  0.13  42.22 |
|  | Chinese Spring | Satellites  SSRs  Low complexity  Total | 130  117  26  273 | 29990  5674  1501  37165 | 6.0  1.13  0.30  7.43 |
|  | Norin-61 | Satellites  SSRs  Low complexity  Total | 121  117  26  264 | 27421  5740  1484  34645 | 5.48  1.15  0.30  6.93 |
|  | Spelt | Satellites  SSRs  Low complexity  Total | 86  118  25  229 | 20362  5987  1381  27730 | 4.07  1.2  0.28  5.55 |
|  | Attraktion | Satellites  SSRs  Low complexity  Total | 75  130  25  115 | 17358  6284  1344  24986 | 3.47  1.26  0.27  5 |
|  | Mace | Satellites  SSRs  Low complexity  Total | 860  54  15  929 | 194431  2841  959  198231 | 38.39  0.57  0.19  39.15 |
|  | Julius | Satellites  SSRs  Low complexity  Total | 66  120  24  210 | 14837  5940  1311  22088 | 2.97  1.19  0.26  4.42 |
|  | *T. dicoccoides* | Satellites  SSRs  Low complexity  Total | 215  120  26  361 | 48595  6101  1514  56210 | 9.72  1.22  0.30  11.24 |
| 5AL | Attraktion | Satellites  SSRs  Low complexity  Total | 0  85  18  103 | 0  4287  783  5070 | 0  0.86  0.16  1.02 |
|  | Kariega | Satellites  SSRs  Low complexity  Total | 0  90  18  108 | 0  4310  783  5093 | 0  0.86  0.16  1.02 |
| 6AS | Chinese Spring | Satellites  SSRs  Low complexity  Total | 9  120  31  160 | 4386  6336  1486  12208 | 0.88  1.27  0.30  2.45 |
|  | T. dicoccoides | Satellites  SSRs  Low complexity  Total | 14  110  27  151 | 6330  7003  1595  14928 | 1.27  1.40  0.32  2.99 |
| 6AL | Aikang58 | Satellites  SSRs  Low complexity  Total | 0  135  31  166 | 0  5621  1745  7366 | 0  1.12  0.35  1.47 |
|  | Spelt | Satellites  SSRs  Low complexity  Total | 0  136  31  167 | 0  5786  1705  7491 | 0  1.16  0.34  1.5 |
|  | Attraktion | Satellites  SSRs  Low complexity  Total | 0  1  0  1 | 0  50  0  50 | 0  0.01  0  0.01 |
|  | Kariega | Satellites  SSRs  Low complexity  Total | 0  154  32  186 | 0  6212  2091  8303 | 0  1.24  0.42  1.66 |
| 7AS | SY Mattis | Satellites  SSRs  Low complexity  Total | 18  136  35  189 | 3689  6456  2178  12323 | 0.74  1.29  0.44  2.47 |
|  | Aikang58 | Satellites  SSRs  Low complexity  Total | 17  127  33  177 | 3449  6092  1779  11320 | 0.69  1.22  0.36  2.27 |
|  | Chinese Spring | Satellites  SSRs  Low complexity  Total | 18  141  40  199 | 3688  7221  2887  13796 | 0.74  1.44  0.58  2.76 |
|  | Spelt | Satellites  SSRs  Low complexity  Total | 5  146  36  187 | 1312  6658  2155  10125 | 0.26  1.33  0.43  2.02 |
|  | Attraktion | Satellites  SSRs  Low complexity  Total | 16  137  31  184 | 3453  6274  1945  11672 | 0.69  1.25  0.39  2.33 |
|  | Renan | Satellites  SSRs  Low complexity  Total | 15  145  39  189 | 2854  7313  2702  12869 | 0.57  1.46  0.54  2.57 |
|  | T. dicoccoides | Satellites  SSRs  Low complexity  Total | 21  135  27  183 | 3970  6341  1562  11873 | 0.79  1.27  0.31  2.37 |
| 7AL | Alchemy | Satellites  SSRs  Low complexity  Total | 0  137  31  168 | 0  6274  1945  8219 | 0  1.25  0.39  1.64 |
|  | Aikang58 | Satellites  SSRs  Low complexity  Total | 0  204  43  247 | 0  9668  2574  12242 | 0  1.93  0.51  2.44 |
|  | Attraktion | Satellites  SSRs  Low complexity  Total | 0  209  45  254 | 0  9998  2747  12745 | 0  2  0.55  2.55 |
|  | Kariega | Satellites  SSRs  Low complexity  Total | 0  213  44  257 | 0  9443  2853  12296 | 0  1.89  0.57  2.46 |
|  | Renan | Satellites  SSRs  Low complexity  Total | 1  197  35  233 | 62  10159  2562  12783 | 0.01  2.03  0.51  2.55 |
| 1BS | LongReach Lancer | Satellites  SSRs  Low complexity  Total | 0  141  27  168 | 0  7259  1691  8950 | 0  1.45  0.34  1.79 |
|  | SY Mattis | Satellites  SSRs  Low complexity  Total | 0  115  28  143 | 0  6234  1795  8029 | 0  1.25  0.36  1.61 |
|  | Spelt | Satellites  SSRs  Low complexity  Total | 185  117  27  329 | 41082  6114  1580  48776 | 8.22  1.22  0.32  9.76 |
|  | Attraktion | Satellites  SSRs  Low complexity  Total | 0  122  28  150 | 0  6721  1957  8678 | 0  1.34  0.39  1.73 |
|  | Kariega | Satellites  SSRs  Low complexity  Total | 0  125  29  154 | 0  6641  2011  8652 | 0  1.33  0.40  1.73 |
| 2BS | Kariega | Satellites  SSRs  Low complexity  Total | 1597  39  5  1641 | 409666  1471  349  411486 | 81.93  0.29  6.98x10^-4^  82.3 |
| 3BS | SY Mattis | Satellites  SSRs  Low complexity  Total | 1  131  25  157 | 82  7130  1907  9119 | 0.02  1.43  0.38  1.83 |
|  | Spelt | Satellites  SSRs  Low complexity  Total | 0  134  25  159 | 0  6941  2238  9179 | 0  1.39  0.45  1.84 |
| 3BL | Attraktion | Satellites  SSRs  Low complexity  Total | 0  168  33  201 | 0  7005  1635  8640 | 0  1.40  0.33  1.73 |
|  | Kariega | Satellites  SSRs  Low complexity  Total | 0  173  32  205 | 0  7143  1593  8736 | 0  1.43  0.32  1.75 |
| 4BS | LongReach Lancer | Satellites  SSRs  Low complexity  Total | 603  65  14  682 | 137729  2893  976  141598 | 27.55  0.58  0.20  28.33 |
|  | CDC Stanley | Satellites  SSRs  Low complexity  Total | 560  70  13  643 | 129662  3550  808  134020 | 25.94  0.71  0.16  26.81 |
|  | Mace | Satellites  SSRs  Low complexity  Total | 598  68  13  679 | 136923  3251  774  140948 | 27.39  0.65  0.15  28.19 |
|  | Kariega | Satellites  SSRs  Low complexity  Total | 614  66  13  693 | 145023  3135  1081  149239 | 29  0.63  0.22  29.83 |
|  | *T. dicoccoides* | Satellites  SSRs  Low complexity  Total | 479  74  12  565 | 111883  3394  774  116051 | 22.38  0.68  0.15  23.21 |
| 6BS | Chinese Spring | Satellites  SSRs  Low complexity  Total | 7  119  26  152 | 3284  6444  1395  11123 | 0.66  1.29  0.28  2.23 |
| 7BS | Alchemy | Satellites  SSRs  Low complexity  Total | 1  172  21  194 | 34  7614  993  8641 | 6.8x10^-3^  1.52  0.2  0.73 |
|  | Chinese Spring | Satellites  SSRs  Low complexity  Total | 1  162  25  188 | 34  6818  1473  8325 | 0.01  1.36  0.29  1.66 |
|  | Spelt | Satellites  SSRs  Low complexity  Total | 1  163  26  190 | 32  7074  1455  8561 | 6.4x10^-3^  1.41  0.29  1.71 |
|  | Mace | Satellites  SSRs  Low complexity  Total | 1  160  21  182 | 34  7009  1233  8276 | 0.01  1.40  0.25  1.66 |
|  | Julius | Satellites  SSRs  Low complexity  Total | 1  162  22  185 | 34  7196  1250  8480 | 0.01  1.44  0.25  1.70 |
|  | *T. turgidum* | Satellites  SSRs  Low complexity  Total | 1  158  23  182 | 34  7229  1389  8652 | 0.01  1.45  0.28  1.74 |
|  | *T. dicoccoides* | Satellites  SSRs  Low complexity  Total | 0  159  26  185 | 0  6756  1544  8300 | 0  1.35  0.31  1.66 |
| 1DS | Spelt | Satellites  SSRs  Low complexity  Total | 0  136  26  162 | 0  6457  1798  8255 | 0  1.29  0.36  1.65 |
|  | Attraktion | Satellites  SSRs  Low complexity  Total | 0  144  26  170 | 0  6476  1780  8256 | 0  1.30  0.36  1.66 |
|  | Kariega | Satellites  SSRs  Low complexity  Total | 0  136  26  162 | 0  6210  1780  7990 | 0  1.24  0.36  1.60 |
|  | *A. tauschii* | Satellites  SSRs  Low complexity  Total | 8  136  24  168 | 1260  6486  1778  9524 | 0.25  1.30  0.36  1.91 |
| 1DL | Alchemy | Satellites  SSRs  Low complexity  Total | 18  138  18  174 | 3959  6488  1539  11986 | 0.79  1.3  0.31  2.4 |
| 2DS | Kariega | Satellites  SSRs  Low complexity  Total | 1820  0  0  1820 | 495277  0  0  495277 | 99.06  0  0  99.06 |
| 2DL | Attraktion | Satellites  SSRs  Low complexity  Total | 9  180  39  228 | 1971  9497  2666  14134 | 0.39  1.90  0.53  2.82 |
|  | Kariega | Satellites  SSRs  Low complexity  Total | 9  180  39  228 | 1971  9559  2665  14195 | 0.39  1.91  0.53  2.84 |
| 3DL | Attraktion | Satellites  SSRs  Low complexity  Total | 0  131  25  156 | 0  6984  1836  8820 | 0  1.40  0.37  1.77 |
|  | Kariega | Satellites  SSRs  Low complexity  Total | 0  127  25  152 | 0  6806  1846  8652 | 0  1.36  0.37  1.73 |
| 4DL | Aikang58 | Satellites  SSRs  Low complexity  Total | 1  115  30  146 | 70  11780  2092  13942 | 0.01  2.36  0.42  2.79 |
|  | Spelt | Satellites  SSRs  Low complexity  Total | 1  118  30  149 | 73  6888  2094  9055 | 0.01  1.38  0.42  1.81 |
| 5DS | Kariega | Satellites  SSRs  Low complexity  Total | 694  30  6  730 | 165709  1435  238  167382 | 33.14  0.29  0.05  33.48 |
| 5DL | Attraktion | Satellites  SSRs  Low complexity  Total | 0  147  19  166 | 0  6317  1000  7317 | 0  1.26  0.2  1.46 |
|  | Kariega | Satellites  SSRs  Low complexity  Total | 0  142  18  160 | 0  6205  1004  7209 | 0  1.24  0.20  1.44 |
|  | A. tauschii | Satellites  SSRs  Low complexity  Total | 0  144  21  165 | 0  6650  1192  7842 | 0  1.33  0.24  1.57 |
| 6DS | ArinaLrFor | Satellites  SSRs  Low complexity  Total | 115  155  33  303 | 25342  7453  1701  34496 | 5.07  1.49  0.34  6.9 |
| 6DL | Attraktion | Satellites  SSRs  Low complexity  Total | 63  155  24  242 | 15108  8241  1580  24929 | 3.02  1.65  0.32  4.99 |
|  | Kariega | Satellites  SSRs  Low complexity  Total | 65  153  24  242 | 15104  8112  1584  24800 | 3.02  1.62  0.32  4.96 |
|  | A. tauschii | Satellites  SSRs  Low complexity  Total | 38  160  28  226 | 8875  7796  1548  18219 | 1.78  1.56  0.31  3.65 |
| 7DS | LongReach Lancer | Satellites  SSRs  Low complexity  Total | 1  115  21  137 | 177  5889  1552  7618 | 0.04  1.18  0.31  1.53 |
|  | Chinese Spring | Satellites  SSRs  Low complexity  Total | 1  115  21  137 | 177  5797  1517  7491 | 0.04  1.16  0.30  1.5 |
|  | Kariega | Satellites  SSRs  Low complexity  Total | 0  109  22  131 | 0  5806  1744  7550 | 0  1.16  0.35  1.51 |
| 7DL | Kariega | Satellites  SSRs  Low complexity  Total | 0  191  35  226 | 0  9428  2437  11865 | 0  1.89  0.49  2.37 |
