# Supplementary material for: Role of chromosome ends in meiotic stability, recombination and wheat evolution in the context of breeding
Source: BMC Plant Biol. 2025 Dec 29;26:187. doi: 10.1186/s12870-025-08020-5 (PMC12859859; doi:10.1186/s12870-025-08020-5)
Supplement: Supplementary file 11 — Supplementary Material 11 [file 12870_2025_8020_MOESM11_ESM.docx]

**Additional file 8.** Transposable elements (TEs) identified in the distal 500 Kb subtelomeric sequence of diploid, tetraploid and hexaploid wheat chromosome arms. The different types of TEs are quantified: DNA transposons; Retroelements (SINEs, LINEs and LTR elements (including Copia and Gypsy).

| **Chromosome** | **Species/cultivar** | **Element** | **Number** | **Length (bp)** | **%** |
| --- | --- | --- | --- | --- | --- |
| 1AS | LongReach Lancer | Retroelements  SINEs  LINEs  LTR elements  *Copia*  *Gypsy*  DNA transposons  Total | 84  0  18  66  24  42  9  93 | 53838  0  10917  42921  10890  32031  10050  64755 | 10.77  0  2.18  8.59  2.18  6.41  2.01  12.95 |
|  | CDC Landmark | Retroelements  SINEs  LINEs  LTR elements  *Copia*  *Gypsy*  DNA transposons  Total | 67  0  1  66  14  52  7  74 | 47309  0  89  47220  7312  39908  9290  56599 | 9.46  0  0.02  9.45  1.46  7.98  1.86  11.32 |
|  | Chinese Spring | Retroelements  SINEs  LINEs  LTR elements  *Copia*  *Gypsy*  DNA transposons  Total | 66  0  1  65  15  50  7  73 | 45609  0  89  45520  7521  37999  9290  54899 | 9.12  0  0.02  9.10  1.50  7.60  1.86  10.98 |
|  | Spelt | Retroelements  SINEs  LINEs  LTR elements  *Copia*  *Gypsy*  DNA transposons  Total | 79  0  0  79  28  51  8  87 | 33791  0  0  33791  7568  26223  8707  42498 | 6.76  0  0  6.76  1.51  5.24  1.74  8.50 |
|  | Fielder | Retroelements  SINEs  LINEs  LTR elements  *Copia*  *Gypsy*  DNA transposons  Total | 68  0  1  67  14  53  7  75 | 47416  0  89  47327  7312  40015  9290  56706 | 9.48  0  0.02  9.47  1.46  8.00  1.86  11.34 |
|  | Kariega | Retroelements  SINEs  LINEs  LTR elements  *Copia*  *Gypsy*  *DIRS*  DNA transposons  Total | 2997  0  584  2413  321  2079  13  664  3661 | 150957  0  49648  101309  16740  83304  1265  40022  190979 | 30.19  0  9.93  20.26  0.03  16.66  0.25  8  38.20 |
| 1AL | Spelt | Retroelements  SINEs  LINEs  LTR elements  *Copia*  *Gypsy*  DNA transposons  Total | 210  0  0  210  83  127  27  237 | 139635  0  0  139635  44980  94655  23937  163572 | 27.93  0  0  27.93  9.00  18.93  4.79  32.71 |
|  | Kariega | Retroelements  SINEs  LINEs  LTR elements  *Copia*  *Gypsy*  DNA transposons  Total | 209  0  6  203  125  78  14  223 | 134707  0  3732  130975  59500  71475  15535  150242 | 26.94  0  0.75  26.20  11.90  14.30  3.11  30.05 |
| 2AS | CDC Landmark | Retroelements  SINEs  LINEs  LTR elements  *Copia*  *Gypsy*  DNA transposons  Total | 204  0  8  196  117  79  26  230 | 141411  0  5671  135740  73472  62288  16828  158239 | 8.29  0  1.13  27.15  14.69  12.46  3.37  31.65 |
|  | SY Mattis | Retroelements  SINEs  LINEs  LTR elements  *Copia*  *Gypsy*  DNA transposons  Total | 262  3  26  233  47  186  30  292 | 198057  241  14746  183070  37939  145131  30066  228123 | 39.61  0.05  2.95  36.61  7.59  29.03  6.01  45.63 |
|  | CDC Stanley | Retroelements  SINEs  LINEs  LTR elements  *Copia*  *Gypsy*  DNA transposons  Total | 262  3  26  233  47  186  29  291 | 198036  241  14746  183049  37939  145110  28690  226726 | 39.62  0.05  2.95  36.62  7.59  29.03  5.74  45.36 |
|  | Jagger | Retroelements  SINEs  LINEs  LTR elements  *Copia*  *Gypsy*  DNA transposons  Total | 261  2  26  233  47  186  29  290 | 197961  151  14746  183064  37939  145125  28630  226591 | 39.6  0.03  2.95  36.62  7.59  29.03  5.73  45.33 |
|  | Fielder | Retroelements  SINEs  LINEs  LTR elements  *Copia*  *Gypsy*  DNA transposons  Total | 205  0  8  197  116  81  29  234 | 145790  0  5671  140119  76300  63819  17992  163782 | 29.16  0  1.13  28.02  15.26  12.76  3.60  32.76 |
|  | Attraktion | Retroelements  SINEs  LINEs  LTR elements  *Copia*  *Gypsy*  DNA transposons  Total | 167  0  3  164  121  43  11  178 | 84175  0  2049  82126  48536  33590  9019  93194 | 16.84  0  0.41  16.43  9.71  6.72  1.80  18.64 |
|  | Kariega | Retroelements  SINEs  LINEs  LTR elements  *Copia*  *Gypsy*  DNA transposons  Total | 167  0  3  164  121  43  11  178 | 84175  0  2049  82126  48536  33590  9019  93194 | 16.84  0  0.41  16.43  9.71  6.72  1.80  18.64 |
| 3AS | SY Mattis | Retroelements  SINEs  LINEs  LTR elements  *Copia*  *Gypsy*  DNA transposons  Total | 201  0  43  158  113  45  39  240 | 128118  0  16643  111475  74284  37191  28716  156834 | 25.62  0  3.33  22.30  14.86  7.44  5.74  31.37 |
|  | Spelt | Retroelements  SINEs  LINEs  LTR elements  *Copia*  *Gypsy*  DNA transposons  Total | 119  0  0  119  90  29  26  145 | 70414  0  0  70414  46110  24304  23833  94247 | 14.08  0  0  14.08  9.22  4.86  4.77  18.85 |
|  | Attraktion | Retroelements  SINEs  LINEs  LTR elements  *Copia*  *Gypsy*  DNA transposons  Total | 93  0  0  93  70  23  51  144 | 36780  0  0  36780  26798  9982  44196  80976 | 7.36  0  0  7.36  5.36  2.00  8.84  16.20 |
|  | Kariega | Retroelements  SINEs  LINEs  LTR elements  *Copia*  *Gypsy*  DNA transposons  Total | 126  0  0  126  110  16  22  148 | 60972  0  0  60972  46312  14660  19052  80024 | 12.19  0  0  12.19  9.26  2.93  3.81  16.01 |
|  | *T. dicoccoides* | Retroelements  SINEs  LINEs  LTR elements  *Copia*  *Gypsy*  DNA transposons  Total | 323  0  69  254  171  83  33  356 | 198463  0  33783  164680  106683  57997  24699  223162 | 39.69  0  6.76  32.94  21.34  11.60  4.94  44.63 |
| 3AL | Kariega | Retroelements  SINEs  LINEs  LTR elements  *Copia*  *Gypsy*  DNA transposons  Total | 149  0  0  149  79  70  15  164 | 127687  0  0  127687  41006  86681  12978  140665 | 25.54  0  0  25.54  8.20  17.34  2.60  28.13 |
| 4AS | ArinaLrFor | Retroelements  SINEs  LINEs  LTR elements  *Copia*  *Gypsy*  DNA transposons  Total | 94  0  16  78  12  66  0  94 | 89870  0  5582  42144  7443  34701  0  89870 | 17.98  0  1.12  8.43  1.49  6.94  0  17.98 |
|  | Chinese Spring | Retroelements  SINEs  LINEs  LTR elements  *Copia*  *Gypsy*  DNA transposons  Total | 225  0  27  198  95  103  15  240 | 165273  0  8619  156654  58312  98342  11088  176361 | 33.5  0  1.72  31.33  11.66  19.67  2.22  35.27 |
|  | Norin-61 | Retroelements  SINEs  LINEs  LTR elements  *Copia*  *Gypsy*  DNA transposons  Total | 222  0  27  195  91  104  16  238 | 166701  0  8619  158082  57050  101032  11555  178256 | 33.34  0  1.72  31.62  11.41  20.21  2.31  35.65 |
|  | Spelt | Retroelements  SINEs  LINEs  LTR elements  *Copia*  *Gypsy*  DNA transposons  Total | 157  0  0  157  80  77  3  160 | 118268  0  0  118268  43432  74836  3570  121838 | 23.65  0  0  23.65  8.69  14.97  0.71  24.37 |
|  | Attraktion | Retroelements  SINEs  LINEs  LTR elements  *Copia*  *Gypsy*  DNA transposons  Total | 145  0  7  138  72  66  2  147 | 106661  0  4442  102219  39243  62976  3565  110226 | 21.33  0  0.89  20.44  7.85  12.60  0.71  22.05 |
|  | Mace | Retroelements  SINEs  LINEs  LTR elements  *Copia*  *Gypsy*  DNA transposons  Total | 61  0  2  59  2  57  6  67 | 45122  0  3046  41074  4048  37026  3096  48218 | 9.03  0  0.61  8.22  0.81  7.41  0.62  9.64 |
|  | Julius | Retroelements  SINEs  LINEs  LTR elements  *Copia*  *Gypsy*  DNA transposons  Total | 199  0  18  191  80  101  15  214 | 155591  0  8931  146660  51409  95251  10584  166175 | 31.12  0  1.79  29.33  10.28  19.05  2.12  33.24 |
|  | *T. dicoccoides* | Retroelements  SINEs  LINEs  LTR elements  *Copia*  *Gypsy*  DNA transposons  Total | 134  0  3  131  53  78  21  155 | 121393  0  1650  119743  41272  78471  13530  134923 | 24.28  0  0.33  23.95  8.25  15.69  2.71  26.98 |
| 5AL | Attraktion | Retroelements  SINEs  LINEs  LTR elements  *Copia*  *Gypsy*  DNA transposons  Total | 159  0  16  143  77  66  14  173 | 90681  0  11582  79099  33772  45327  10363  101044 | 18.14  0  2.32  15.82  6.75  9.07  2.07  20.21 |
|  | Kariega | Retroelements  SINEs  LINEs  LTR elements  *Copia*  *Gypsy*  DNA transposons  Total | 148  0  9  139  73  66  14  162 | 86669  0  7428  79241  34937  44304  10315  96984 | 17.33  0  1.49  15.85  6.99  8.86  2.06  19.40 |
| 6AS | Chinese Spring | Retroelements  SINEs  LINEs  LTR elements  *Copia*  *Gypsy*  DNA transposons  Total | 222  1  28  193  56  137  91  313 | 146065  72  18167  127826  25921  101905  59114  205179 | 29.21  0.01  3.63  25.57  5.18  20.38  11.82  41.03 |
|  | T. dicoccoides | Retroelements  SINEs  LINEs  LTR elements  *Copia*  *Gypsy*  DNA transposons  Total | 231  1  24  206  97  109  109  340 | 141286  72  17133  124081  46940  77141  83189  22 | 28.26  0.01  3.43  24.82  9.39  15.43  16.54  44.90 |
| 6AL | Aikang58 | Retroelements  SINEs  LINEs  LTR elements  *Copia*  *Gypsy*  DNA transposons  Total | 222  0  19  203  116  87  18  240 | 125574  0  11579  113995  55441  58554  12411  137985 | 25.11  0  2.32  22.80  11.09  11.71  2.48  27.60 |
|  | Spelt | Retroelements  SINEs  LINEs  LTR elements  *Copia*  *Gypsy*  DNA transposons  Total | 219  0  18  201  109  92  16  235 | 125793  0  12252  113541  54266  59275  11846  137639 | 25.16  0  2.45  22.71  10.85  11.86  2.37  27.53 |
|  | Attraktion | Retroelements  SINEs  LINEs  LTR elements  *Copia*  *Gypsy*  DNA transposons  Total | 441  0  77  364  121  243  21  462 | 34323  0  5947  28376  10028  18348  1311  35634 | 6.86  0  1.19  5.68  2.01  3.67  0.26  7.13 |
|  | Kariega | Retroelements  SINEs  LINEs  LTR elements  *Copia*  *Gypsy*  DNA transposons  Total | 214  0  16  198  109  89  16  230 | 122819  0  10583  112236  53663  58573  11873  134692 | 24.56  0  2.12  22.45  10.73  11.71  2.37  26.94 |
| 7AS | SY Mattis | Retroelements  SINEs  LINEs  LTR elements  *Copia*  *Gypsy*  DNA transposons  Total | 275  1  64  210  126  84  95  370 | 191114  180  47183  143751  80868  62883  43216  234330 | 38.22  0.04  9.44  28.75  16.17  12.58  8.64  46.87 |
|  | Aikang58 | Retroelements  SINEs  LINEs  LTR elements  *Copia*  *Gypsy*  DNA transposons  Total | 165  0  24  141  89  52  20  185 | 115686  0  17763  97923  51010  46913  14656  130342 | 23.14  0  3.55  19.58  10.20  9.38  2.93  26.07 |
|  | Chinese Spring | Retroelements  SINEs  LINEs  LTR elements  *Copia*  *Gypsy*  DNA transposons  Total | 285  1  74  210  126  84  79  364 | 192970  180  50401  142389  80562  61827  41489  234459 | 38.59  0.04  10.08  28.48  16.11  12.37  8.30  46.89 |
|  | Spelt | Retroelements  SINEs  LINEs  LTR elements  *Copia*  *Gypsy*  DNA transposons  Total | 150  0  26  124  84  40  27  177 | 88851  0  20257  68594  38343  30251  18078  106929 | 17.77  0  4.05  13.72  7.67  6.05  3.62  21.39 |
|  | Attraktion | Retroelements  SINEs  LINEs  LTR elements  *Copia*  *Gypsy*  DNA transposons  Total | 96  0  8  88  44  44  15  111 | 53703  0  7684  46019  20134  25885  15069  68772 | 10.74  0  1.54  9.20  4.03  5.18  3.01  13.75 |
|  | Renan | Retroelements  SINEs  LINEs  LTR elements  Copia  Gypsy  DNA transposons  Total | 159  0  19  140  96  44  31  190 | 107115  0  17414  89701  54591  35110  21455  128570 | 21.42  0  3.48  17.94  10.92  7.02  4.29  25.71 |
|  | T. dicoccoides | Retroelements  SINEs  LINEs  LTR elements  *Copia*  *Gypsy*  DNA transposons  Total | 266  1  61  204  121  83  80  346 | 204722  180  45659  158883  87422  71461  34453  239175 | 40.94  0.04  9.13  31.78  17.48  14.29  6.89  47.84 |
| 7AL | Alchemy | Retroelements  SINEs  LINEs  LTR elements  *Copia*  *Gypsy*  DNA transposons  Total | 96  0  8  88  44  44  15  111 | 53703  0  7684  46019  20134  25885  15069  68772 | 10.74  0  1.54  9.20  4.03  5.18  3.01  13.75 |
|  | Aikang58 | Retroelements  SINEs  LINEs  LTR elements  *Copia*  *Gypsy*  DNA transposons  Total | 144  0  0  144  107  37  15  159 | 75675  0  0  75675  60289  15386  13680  89355 | 15.14  0  0  15.14  12.06  3.08  2.74  17.87 |
|  | Attraktion | Retroelements  SINEs  LINEs  LTR elements  *Copia*  *Gypsy*  DNA transposons  Total | 159  0  3  156  114  42  20  179 | 73590  0  3891  69699  52971  16728  18076  91666 | 14.72  0  0.78  13.94  10.59  3.35  3.62  18.33 |
|  | Kariega | Retroelements  SINEs  LINEs  LTR elements  *Copia*  *Gypsy*  DNA transposons  Total | 99  0  0  99  70  29  13  112 | 49626  0  0  49626  31154  18472  13136  62762 | 9.93  0  0  9.93  6.23  3.69  2.63  12.55 |
|  | Renan | Retroelements  SINEs  LINEs  LTR elements  Copia  Gypsy  DNA transposons  Total | 149  0  0  149  121  28  17  166 | 75103  0  0  75103  54353  20750  15316  90419 | 15.02  0  0  15.02  10.87  4.15  3.06  18.08 |
| 1BS | LongReach Lancer | Retroelements  SINEs  LINEs  LTR elements  *Copia*  *Gypsy*  DNA transposons  Total | 199  0  28  171  126  45  100  299 | 152891  0  26348  126543  91149  35394  74441  227332 | 30.59  0  5.27  25.31  18.23  7.08  14.89  45.48 |
|  | SY Mattis | Retroelements  SINEs  LINEs  LTR elements  *Copia*  *Gypsy*  DNA transposons  Total | 262  0  0  262  86  176  91  353 | 182096  0  0  182096  53701  128395  66227  248323 | 36.42  0  0  36.42  10.74  25.68  13.25  49.66 |
|  | Spelt | Retroelements  SINEs  LINEs  LTR elements  *Copia*  *Gypsy*  DNA transposons  Total | 196  0  0  196  97  99  69  265 | 112469  0  0  112469  44369  68100  52959  165428 | 22.49  0  0  22.49  8.87  13.62  10.59  33.09 |
|  | Attraktion | Retroelements  SINEs  LINEs  LTR elements  *Copia*  *Gypsy*  DNA transposons  Total | 197  0  0  197  97  100  67  264 | 110311  0  0  110311  44174  66137  52577  162888 | 22.06  0  0  22.06  8.83  13.23  10.52  32.58 |
|  | Kariega | Retroelements  SINEs  LINEs  LTR elements  *Copia*  *Gypsy*  DNA transposons  Total | 185  0  0  185  89  96  65  250 | 107431  0  0  107431  43096  64335  52633  160064 | 21.49  0  0  21.49  8.62  12.87  10.53  32.01 |
| 2BS | Kariega | Retroelements  SINEs  LINEs  LTR elements  *Copia*  *Gypsy*  DNA transposons  Total | 70  0  0  70  51  19  0  70 | 27458  0  0  27458  18045  9413  0  27458 | 5.49  0  0  5.49  3.61  1.88  0  5.49 |
| 3BS | SY Mattis | Retroelements  SINEs  LINEs  LTR elements  *Copia*  *Gypsy*  DNA transposons  Total | 249  1  61  187  81  106  56  305 | 200570  37  35534  164999  65454  99545  31927  232497 | 40.11  0.01  7.11  33.00  13.09  19.91  6.39  46.50 |
|  | Spelt | Retroelements  SINEs  LINEs  LTR elements  *Copia*  *Gypsy*  DNA transposons  Total | 159  0  24  135  78  57  26  185 | 120966  0  15471  105495  47517  57978  19033  139999 | 24.19  0  3.09  21.10  9.50  11.60  3.81  28.00 |
| 3BL | Attraktion | Retroelements  SINEs  LINEs  LTR elements  *Copia*  *Gypsy*  DNA transposons  Total | 137  0  5  132  109  23  14  151 | 73387  0  6406  66981  51530  15451  10432  83819 | 14.68  0  1.28  13.40  10.31  3.09  2.09  16.76 |
|  | Kariega | Retroelements  SINEs  LINEs  LTR elements  *Copia*  *Gypsy*  DNA transposons  Total | 136  0  5  131  113  18  15  151 | 70310  0  6406  63904  51535  12369  10620  80930 | 14.06  0  1.28  12.78  10.31  2.47  2.12  16.19 |
| 4BS | LongReach Lancer | Retroelements  SINEs  LINEs  LTR elements  *Copia*  *Gypsy*  DNA transposons  Total | 211  0  0  211  43  168  15  226 | 166988  0  0  166988  31913  135075  5295  172283 | 33.40  0  0  33.40  6.38  27.02  1.06  34.46 |
|  | CDC Stanley | Retroelements  SINEs  LINEs  LTR elements  *Copia*  *Gypsy*  DNA transposons  Total | 230  0  0  230  60  170  13  243 | 170227  0  0  170227  35445  134782  4727  174954 | 34.05  0  0  34.05  7.09  26.96  0.95  35.00 |
|  | Mace | Retroelements  SINEs  LINEs  LTR elements  *Copia*  *Gypsy*  DNA transposons  Total | 222  0  0  222  59  163  13  235 | 166953  0  0  166953  35215  131738  4727  171680 | 33.40  0  0  33.40  7.04  26.35  0.95  34.34 |
|  | Kariega | Retroelements  SINEs  LINEs  LTR elements  *Copia*  *Gypsy*  DNA transposons  Total | 149  0  0  149  7  142  7  156 | 88477  0  0  88477  7391  81086  2627  91104 | 17.70  0  0  17.70  1.48  16.22  0.53  18.22 |
|  | *T. dicoccoides* | Retroelements  SINEs  LINEs  LTR elements  *Copia*  *Gypsy*  DNA transposons  Total | 221  0  0  221  64  157  21  242 | 161542  0  0  161542  37928  123614  11217  172759 | 32.31  0  0  32.31  7.59  24.72  2.24  34.55 |
| 6BS | Chinese Spring | Retroelements  SINEs  LINEs  LTR elements  *Copia*  *Gypsy*  DNA transposons  Total | 238  0  57  181  102  79  56  294 | 191621  0  33900  157721  76428  81293  38497  230118 | 38.32  0  6.78  31.54  15.29  16.26  7.60  46.02 |
| 7BS | Alchemy | Retroelements  SINEs  LINEs  LTR elements  *Copia*  *Gypsy*  DNA transposons  Total | 154  0  15  139  100  39  12  166 | 83342  0  12003  71339  40199  31140  6420  89762 | 16.67  0  2.40  14.27  8.04  6.23  1.28  17.95 |
|  | Chinese Spring | Retroelements  SINEs  LINEs  LTR elements  *Copia*  *Gypsy*  DNA transposons  Total | 290  0  31  259  154  105  33  323 | 214042  0  22403  191639  106115  85524  22410  236452 | 42.81  0  4.48  38.33  21.22  17.10  4.48  47.29 |
|  | Spelt | Retroelements  SINEs  LINEs  LTR elements  *Copia*  *Gypsy*  DNA transposons  Total | 206  0  0  206  145  61  12  218 | 132316  0  0  132316  74273  58043  11649  143965 | 26.46  0  0  26.46  14.85  11.61  2.33  28.79 |
|  | Mace | Retroelements  SINEs  LINEs  LTR elements  *Copia*  *Gypsy*  DNA transposons  Total | 298  0  35  263  146  117  33  331 | 221312  0  23233  198079  101851  96228  22398  243710 | 44.27  0  4.65  39.63  20.38  19.25  4.48  48.75 |
|  | Julius | Retroelements  SINEs  LINEs  LTR elements  *Copia*  *Gypsy*  DNA transposons  Total | 284  0  32  252  149  103  32  316 | 210250  0  20323  189927  104347  85580  22351  232601 | 42.06  0  4.07  37.99  20.87  17.12  4.47  46.53 |
|  | *T. turgidum* | Retroelements  SINEs  LINEs  LTR elements  *Copia*  *Gypsy*  DNA transposons  Total | 295  0  34  261  144  117  33  328 | 219986  0  23153  196833  100623  96210  22398  242384 | 44.01  0  4.63  39.38  20.13  19.25  4.48  48.49 |
|  | *T. dicoccoides* | Retroelements  SINEs  LINEs  LTR elements  *Copia*  *Gypsy*  DNA transposons  Total | 321  0  33  288  135  153  46  367 | 234543  0  18645  215898  99654  116244  29384  263927 | 46.91  0  3.73  43.18  19.93  23.25  5.88  52.79 |
| 1DS | Spelt | Retroelements  SINEs  LINEs  LTR elements  *Copia*  *Gypsy*  DNA transposons  Total | 163  0  14  146  126  23  12  175 | 84473  0  9610  74863  57048  17815  14370  98843 | 16.89  0  1.92  14.97  11.41  3.56  2.87  19.77 |
|  | Attraktion | Retroelements  SINEs  LINEs  LTR elements  *Copia*  *Gypsy*  DNA transposons  Total | 169  0  13  156  132  24  13  182 | 90848  0  8675  82173  62568  19605  14669  105517 | 18.17  0  1.74  16.43  12.51  3.92  2.93  21.10 |
|  | Kariega | Retroelements  SINEs  LINEs  LTR elements  *Copia*  *Gypsy*  DNA transposons  Total | 168  0  13  155  135  20  14  182 | 86091  0  8675  77416  58272  19144  14737  100828 | 17.22  0  1.74  15.48  11.65  3.83  2.95  20.17 |
|  | *A. tauschii* | Retroelements  SINEs  LINEs  LTR elements  *Copia*  *Gypsy*  DNA transposons  Total | 169  0  0  169  150  19  24  194 | 87497  0  0  87497  70719  16778  16689  106348 | 17.50  0  0  17.50  14.14  3.36  3.34  21.27 |
| 1DL | Alchemy | Retroelements  SINEs  LINEs  LTR elements  *Copia*  *Gypsy*  DNA transposons  Total | 184  0  17  167  109  58  8  192 | 89084  0  13774  75310  48731  26579  8221  97305 | 17.82  0  2.75  15.06  9.75  5.32  1.64  19.46 |
| 2DS | Kariega | Retroelements  SINEs  LINEs  LTR elements  *Copia*  *Gypsy*  DNA transposons  Total | 1609  0  616  993  686  307  395  2004 | 122315  0  43643  78672  58618  20054  28132  150447 | 24.46  0  8.73  15.73  11.72  4.01  5.63  30.09 |
| 2DL | Attraktion | Retroelements  SINEs  LINEs  LTR elements  *Copia*  *Gypsy*  DNA transposons  Total | 115  0  0  115  21  94  33  148 | 57431  0  0  57431  11754  45677  28839  86270 | 11.49  0  0  11.49  2.35  9.14  5.77  17.25 |
|  | Kariega | Retroelements  SINEs  LINEs  LTR elements  *Copia*  *Gypsy*  DNA transposons  Total | 114  0  0  114  21  93  33  147 | 57351  0  0  57351  11754  45597  28839  86190 | 11.47  0  0  11.47  2.35  9.12  5.77  17.24 |
| 3DL | Attraktion | Retroelements  SINEs  LINEs  LTR elements  *Copia*  *Gypsy*  DNA transposons  Total | 177  0  43  134  59  75  9  186 | 103716  0  23564  80152  35911  44241  6534  110250 | 20.74  0  4.71  16.03  7.18  8.85  1.31  22.05 |
|  | Kariega | Retroelements  SINEs  LINEs  LTR elements  *Copia*  *Gypsy*  DNA transposons  Total | 174  0  43  131  58  73  9  183 | 103539  0  23480  80059  35849  44210  6636  110175 | 20.71  0  4.70  16.01  7.17  8.84  1.33  22.04 |
| 4DL | Aikang58 | Retroelements  SINEs  LINEs  LTR elements  *Copia*  *Gypsy*  DNA transposons  Total | 174  0  0  174  72  102  24  198 | 91254  0  0  91254  37761  53493  21111  112365 | 18.25  0  0  18.25  7.55  10.70  4.22  22.47 |
|  | Spelt | Retroelements  SINEs  LINEs  LTR elements  *Copia*  *Gypsy*  DNA transposons  Total | 179  0  0  179  78  101  23  202 | 92689  0  0  92689  39462  53227  20200  112889 | 18.54  0  0  18.54  7.89  10.65  4.04  22.58 |
| 5DS | Kariega | Retroelements  SINEs  LINEs  LTR elements  *Copia*  *Gypsy*  DNA transposons  Total | 96  0  0  96  60  36  0  96 | 58161  0  0  58161  25704  32457  0  58161 | 11.63  0  0  11.63  5.14  6.49  0  11.63 |
| 5DL | Attraktion | Retroelements  SINEs  LINEs  LTR elements  *Copia*  *Gypsy*  DNA transposons  Total | 222  0  7  215  43  172  0  222 | 123501  0  10796  112705  18128  94577  0  123501 | 24.70  0  2.16  22.54  3.63  18.92  0  24.70 |
|  | Kariega | Retroelements  SINEs  LINEs  LTR elements  *Copia*  *Gypsy*  DNA transposons  Total | 179  0  6  173  81  92  6  185 | 118363  0  6372  111991  44670  67321  6726  125089 | 23.67  0  1.27  22.40  8.93  13.46  1.35  25.02 |
|  | A. tauschii | Retroelements  SINEs  LINEs  LTR elements  *Copia*  *Gypsy*  DNA transposons  Total | 128  0  8  120  48  72  6  248 | 86515  0  7285  79230  20811  58419  4905  165745 | 17.30  0  1.46  15.85  4.16  11.68  0.98  33.15 |
| 6DS | ArinaLrFor | Retroelements  SINEs  LINEs  LTR elements  *Copia*  *Gypsy*  DNA transposons  Total | 186  0  27  159  140  19  60  246 | 136464  0  15888  120576  107194  13382  37803  174267 | 27.29  0  3.18  24.12  21.44  2.68  7.56  34.85 |
| 6DL | Attraktion | Retroelements  SINEs  LINEs  LTR elements  *Copia*  *Gypsy*  DNA transposons  Total | 205  0  0  205  156  49  21  226 | 108356  0  0  108356  75153  33203  16455  124811 | 21.67  0  0  21.67  15.03  6.64  3.29  24.96 |
|  | Kariega | Retroelements  SINEs  LINEs  LTR elements  *Copia*  *Gypsy*  DNA transposons  Total | 205  0  0  205  156  49  20  225 | 108356  0  0  108356  75153  33203  16492  124848 | 21.67  0  0  21.67  15.03  6.64  3.30  24.97 |
|  | A. tauschii | Retroelements  SINEs  LINEs  LTR elements  *Copia*  *Gypsy*  DNA transposons  Total | 227  0  0  227  168  59  14  241 | 124240  0  0  124240  85890  38350  10597  134837 | 24.85  0  0  24.85  17.19  7.67  2.12  26.97 |
| 7DS | LongReach Lancer | Retroelements  SINEs  LINEs  LTR elements  *Copia*  *Gypsy*  DNA transposons  Total | 267  0  1  266  201  65  35  302 | 159774  0  386  159388  113310  46078  22888  182662 | 31.96  0  0.08  31.88  22.66  9.22  4.58  36.54 |
|  | Chinese Spring | Retroelements  SINEs  LINEs  LTR elements  *Copia*  *Gypsy*  DNA transposons  Total | 184  0  13  171  111  60  45  229 | 117905  0  6128  111777  75804  35973  29871  147776 | 23.58  0  1.23  22.36  15.16  7.19  5.97  29.56 |
|  | Kariega | Retroelements  SINEs  LINEs  LTR elements  *Copia*  *Gypsy*  DNA transposons  Total | 162  0  0  162  138  24  12  174 | 77382  0  0  77382  62133  15249  11064  88446 | 15.48  0  0  15.48  12.43  3.05  2.21  17.69 |
| 7DL | Kariega | Retroelements  SINEs  LINEs  LTR elements  *Copia*  *Gypsy*  DNA transposons  Total | 154  0  9  145  145  0  24  178 | 54824  0  8256  46568  46568  0  16818  71642 | 10.96  0  1.65  9.31  9.31  0  3.36  14.33 |
